# Pharmacological and dietary-supplement treatments for autism spectrum disorder: a systematic review and network meta-analysis

**DOI:** 10.1186/s13229-022-00488-4

**Published:** 2022-03-04

**Authors:** Spyridon Siafis, Oğulcan Çıray, Hui Wu, Johannes Schneider-Thoma, Irene Bighelli, Marc Krause, Alessandro Rodolico, Anna Ceraso, Giacomo Deste, Maximilian Huhn, David Fraguas, Antonia San José Cáceres, Dimitris Mavridis, Tony Charman, Declan G. Murphy, Mara Parellada, Celso Arango, Stefan Leucht

**Affiliations:** 1 Department of Psychiatry and Psychotherapy, School of Medicine, Technical University of Munich, Ismaningerstr. 22, 81675 Munich, Germany; 2 Department of Child and Adolescent Psychiatry, Mardin State Hospital, Artuklu, Mardin Turkey; 3 Department of Psychiatry and Psychotherapy, School of Medicine, Technical University of Munich, Munich, Germany; 4 Department of Experimental and Clinical Medicine, Psychiatric Clinic University Hospital ‘Gaspare Rodolico’, University of Catania, Catania, Italy; 5 Department of Psychiatry, Spedali Civili Hospital, Brescia, Italy; 6 Department of Psychiatry, Psychosomatic Medicine and Psychotherapy, Social Foundation Bamberg, Teaching Hospital of the University of Erlangen, Bamberg, Germany; 7 Institute of Psychiatry and Mental Health, Hospital Clínico San Carlos, IdISSC CIBERSAM, School of Medicine, Universidad Complutense, Madrid, Spain; 8 Department of Child and Adolescent Psychiatry, Institute of Psychiatry and Mental Health, Hospital General Universitario Gregorio Marañón, Madrid, Spain; 9 Department of Primary Education, University of Ioannina, Ioannina, Greece; 10 Faculté de Médecine, Université Paris Descartes, Paris, France; 11 Department of Psychology, Institute of Psychiatry, Psychology and Neuroscience, King’s College London, London, UK; 12 Department of Forensic and Neurodevelopmental Sciences, Institute of Psychiatry, Psychology and Neuroscience, King’s College London, London, UK; 13 School of Medicine, Universidad Complutense, Madrid, Spain; 14 Instituto de Investigación Sanitaria Gregorio Marañón, Madrid, Spain; 15 Centro Investigación Biomédica en Red Salud Mental (CIBERSAM), Madrid, Spain

**Keywords:** Autism, Meta-analysis, Treatment, Response, Social communication, Restricted and repetitive behaviors, Irritability, ADHD, Anxiety, Caregiver stress

## Abstract

**Background:**

There is still no approved medication for the core symptoms of autism spectrum disorder (ASD). This network meta-analysis investigated pharmacological and dietary-supplement treatments for ASD.

**Methods:**

We searched for randomized-controlled-trials (RCTs) with a minimum duration of seven days in ClinicalTrials.gov, EMBASE, MEDLINE, PsycINFO, WHO-ICTRP (from inception up to July 8, 2018), CENTRAL and PubMed (up to November 3, 2021). The co-primary outcomes were core symptoms (social-communication difficulties-SCD, repetitive behaviors-RB, overall core symptoms-OCS) measured by validated scales and standardized-mean-differences (SMDs). Associated symptoms, e.g., irritability/aggression and attention-deficit/hyperactivity disorder (ADHD) symptoms, dropouts and important side-effects, were investigated as secondary outcomes. Studies in children/adolescents and adults were analyzed separately in random-effects pairwise and network meta-analyses.

**Results:**

We analyzed data for 41 drugs and 17 dietary-supplements, from 125 RCTs (*n* = 7450 participants) in children/adolescents and 18 RCTs (*n* = 1104) in adults. The following medications could improve at least one core symptom domain in comparison with placebo: aripiprazole (*k* = 6 studies in analysis, SCD: SMD = 0.27 95% CI [0.09, 0.44], RB: 0.48 [0.26, 0.70]), atomoxetine (*k* = 3, RB:0.49 [0.18, 0.80]), bumetanide (*k* = 4, RB: 0.35 [0.09, 0.62], OCS: 0.61 [0.31, 0.91]), and risperidone (*k* = 4, SCM: 0.31 [0.06, 0.55], RB: 0.60 [0.29, 0.90]; *k* = 3, OCS: 1.18 [0.75, 1.61]) in children/adolescents; fluoxetine (*k* = 1, RB: 1.20 [0.45, 1.96]), fluvoxamine (*k* = 1, RB: 1.04 [0.27, 1.81]), oxytocin (*k* = 6, RB:0.41 [0.16, 0.66]) and risperidone (*k* = 1, RB: 0.97 [0.21,1.74]) in adults. There were some indications of improvement by carnosine, haloperidol, folinic acid, guanfacine, omega-3-fatty-acids, probiotics, sulforaphane, tideglusib and valproate, yet imprecise and not robust. Confidence in these estimates was very low or low, except moderate for oxytocin. Medications differed substantially in improving associated symptoms, and in their side-effect profiles.

**Limitations:**

Most of the studies were inadequately powered (sample sizes of 20–80 participants), with short duration (8–13 weeks), and about a third focused on associated symptoms. Networks were mainly star-shaped, and there were indications of reporting bias. There was no optimal rating scale measuring change in core symptoms.

**Conclusions:**

Some medications could improve core symptoms, although this could be likely secondary to the improvement of associated symptoms. Evidence on their efficacy and safety is preliminary; therefore, routine prescription of medications for the core symptoms cannot be recommended.

*Trial registration* PROSPERO-ID CRD42019125317.

**Supplementary Information:**

The online version contains supplementary material available at 10.1186/s13229-022-00488-4.

## Background

Autism spectrum disorder (ASD) consists of heterogeneous conditions, which are characterized by social-communication difficulties, restricted interests/repetitive behaviors and sensory abnormalities [1]. Behavioral interventions are the mainstay treatment [2]. Medications with different mechanisms of action have been examined in randomized controlled trials (RCTs) [3–5], and some of them have been found efficacious for associated symptoms, such as aripiprazole, risperidone and haloperidol for irritability, methylphenidate, atomoxetine, clonidine and guanfacine for attention-deficit/hyperactivity disorder (ADHD) symptoms and melatonin for sleep disorders [2, 6]. However, prior late-stage clinical trials failed to identify efficacious treatments for the core symptoms of neurodevelopmental disorders [5, 7]. Despite lack of clear evidence in efficacy, about half of the individuals with ASD receive psychotropic drugs [8]. The current synthesis of literature is restricted to medication classes or target symptoms [9–19], hence failing to combine the huge amount of recently conducted RCTs [3–5]. In order to better inform clinical practice and identify medications potentially efficacious for ASD, we combined evidence from pharmacological and dietary-supplement ASD trials in a network meta-analysis.

## Methods

### Search strategy and selection criteria

This network meta-analysis analyzed placebo-controlled and head-to-head RCTs on pharmacological/dietary-supplement interventions for ASD according to the PRISMA-NMA (Additional file 1: eAppendix-1) [20], and with PROSPERO-ID: CRD42019125317 (Additional file 1: eAppendix-2).

We searched ClinicalTrials.gov, EMBASE, MEDLINE, PsycINFO, WHO-ICTRP (from inception to July 8, 2018), CENTRAL and PubMed (last update on November 3, 2021), without restrictions in terms of language, document type, date/time and publication status (Additional file 1: eAppendix-3). Reference lists of included studies and reviews [2, 9–17, 19, 21–24] were inspected.

Participants should have a diagnosis of ASD according to standardized diagnostic criteria (e.g., DSM-III or newer versions) and/or validated diagnostic tools (e.g., ADI-R) [2], without restrictions in terms of age, sex, baseline severity and presence of genetic syndromes or other associated conditions (e.g., irritability, ADHD symptoms).

Any drug, dietary-supplement or placebo was eligible. We excluded augmentation and multimodal interventions (e.g., medications combined with risperidone or behavioral interventions) as well as other types of interventions (e.g., behavioral, elimination diets). The minimum duration of treatment was seven days, and there was no restriction in terms of dosing-schedule and route of administration. Multiple doses of the same intervention were combined [25] (Additional file 1: eAppendix-2.2).

Blinded and open RCTs were eligible. RCTs with a low or unclear risk of bias in random sequence generation and allocation concealment were eligible, yet we excluded trials with a high risk of bias in these domains [26]. Trials stated to be randomized but did not report the exact randomization methods (unclear risk of bias) were eligible, since poor reporting does not necessarily reflect the actual conducted methods [27–30] (Additional file 1: eAppendix-2.2). However, such trials were excluded in a *post hoc* sensitivity analysis. We included data only from the first phase of crossover studies in order to avoid carry-over effects [31], and we excluded discontinuation studies, studies published before 1980, or with a randomized sample smaller than ten participants [32].

At least two independent reviewers/contributors selected relevant records (SS, OC, HW, IB, MK, YZ, AC, GD and TF), extracted data from eligible studies into an Access database as well as evaluated risk of bias using the Cochrane risk-of-bias tool (SS, OC, AR, HW) [26]. Studies were rated as having a low, moderate or high overall risk of bias [33]. Differences were resolved with discussion, and if needed, a third reviewer was involved (SL, JST). Study authors were contacted for additional data by e-mail (with a reminder in case of no response) (Additional file 1: eAppendix-4).

### Outcomes

The co-primary outcomes were the change in core symptoms measured with validated rating scales: (1) social-communication difficulties (SCD, e.g., ABC-L/SW [34] or VABS-Socialization [35]), (2) repetitive behaviors (RB, e.g., ABC-S [34] or CYBOCS-PDD [36]), and (3) overall core symptoms (OCS, e.g., SRS [37] or CARS [38]). There is yet no optimal outcome measure [39], and we accepted a wide range of validated scales, giving preference to clinician-ratings and to the commonly used scales mentioned above, similar to our previous review [4] (Additional file 1: eAppendix-5.3).

Secondary outcomes were premature discontinuation (dropout) due to any reason and due to adverse events, number of participants with a positive response (preferably defined with a CGI-Improvement score ≤ 2 or at least “much improved” [40]), change in irritability/aggression, ADHD symptoms and anxiety/depression, quality of life, global functioning and caregiver stress (Additional file 1: eAppendix-5.3). We also examined the number of participants with adverse events, sedation, weight gain (preferably defined as ≥ 7% increase) and extrapyramidal symptoms.

### Data analysis

Random-effects pairwise and network meta-analyses were conducted within a frequentist framework using meta v4.15-1 [41] and netmeta v1.2-1 [42] in R statistical software v4.0.3 [43]. The certainty of evidence of comparisons with placebo for the co-primary outcomes was evaluated using CINeMA (Confidence in Network Meta-Analysis) [44, 45] (Additional file 1: eAppendix-6.9).

The effect-sizes for continuous outcomes were standardized mean differences (SMD, Hedge’s g) and for dichotomous outcomes were odds ratios (OR), presented with their 95% confidence intervals (95% CI). We post hoc used ORs instead of relative risks, due to their preferred mathematical properties in meta-analysis [46, 47]. In order to present both continuous and dichotomous outcomes in figures, ORs were also converted to SMDs [25]. Treatments were ranked with *P*-scores [48]. Intention-to-treat data were used, whenever available, and methods that handle missing data were preferred to completers’ data, giving preference to mixed-models for repeated measures (MMRM) and multiple imputation over last-observation carried forward (LOCF). For dichotomous outcomes, we assumed that participants lost to follow-up did not have a response. The number of participants with a positive response (CGI-Improvement ≤ 2) [40] and weight gain (≥ 7% increase) was imputed from means and standard deviations (SD) using a validated method, when dichotomous data were not reported [49, 50]. Missing SDs were calculated from available statistics [25], pooling subscales (e.g., SRS subscales, assuming a correlation of 0.5) [51] or using the mean SD of included studies [25]. Change scores were preferred to follow-up scores, and the former were estimated post hoc when both baseline and follow-up scores were available using a correlation of 0.5 [25], since baseline imbalances could have inflated treatment effects (Additional file 1: eAppendix-6.1).

RCTs in children/adolescents and adults (or mixed populations) were analyzed separately, since extrapolation between age groups is discouraged [52]. Transitivity was further assessed by comparing the distribution of clinical and methodological variables (i.e., study duration, type of rater, associated conditions at baseline, baseline scores of CGI-Severity (ranging 1–7) [40], ABC-Irritability (ranging 0–45) [34] and mean age). Trials focused on subgroups, i.e., intellectual disability/high-functioning, genetic syndrome or another associated condition, were classified in CINeMA with moderate indirectness [44].

A common heterogeneity variance (*τ*^2^) was assumed for all comparisons per network, and heterogeneity was quantified as low, moderate or high by comparing *τ*^2^ with its empirical distributions [53, 54]. Incoherence was examined globally with a design-by-treatment interaction test and locally with separating indirect from direct evidence [55].

We aimed to include unpublished trials (e.g., contacting authors, using data reported in trial registries, abstracts and reviews), and eligible studies with no usable data were considered in the assessment of reporting bias [44]. Additionally, small-study effects were examined with comparison-adjusted [56] (assuming the direction of bias towards newer medications) and contour-enhanced funnel plots, when there were more than ten studies per comparison [25].

The robustness of the results was investigated in sensitivity analyses using (a) fixed-effects models, excluding studies with (b) implied randomization, (c) genetic syndrome or (d) associated symptoms as inclusion criteria, (e) using only diagnostic evaluation tools, (f) with non-clinician-ratings, (g) from less developed countries (post hoc) [57], (h) with imputed SDs, (i) overall high risk of bias, (j) unclear risk of bias in random sequence generation or allocation concealment (post hoc), (k) open or single-blind, (l) shorter than four weeks, (m) presenting only completers’ data, (n) using a correlation of 0.25 and 0.75 to calculate the SD of change scores, and (o) using ABC-L/SW or ABC-S (post hoc). In a post hoc sensitivity analysis, relative risks were used for dichotomous outcomes. Baseline severity could not be assessed in a subgroup or sensitivity analysis, due to inconsistent reporting and diversity of scales (Additional file 1: eAppendix-2.2).

Alpha was set at two-sided 0.05, except for heterogeneity, incoherence and funnel plot tests at 0.1 due to their small statistical power.

## Results

### Description of included studies

Study selection is presented with a PRISMA flow diagram (Additional file 1: eAppendix-4.1), and the list of included/excluded full-texts in Additional file 1: eAppendix-4.2/4.3. From 203 eligible trials, 125 trials in children/adolescents (*n* = 7450 participants) and 18 in adults (*n* = 1104) were included in the quantitative analysis.

Study characteristics are presented in Additional file 1: eAppendix-5.1 and the distribution of potential effect-modifiers in Additional file 1: eAppendix-6.1. The majority of trials were double-blind (*k* = 138 studies), placebo-controlled (*k* = 137) with a parallel-design (*k* = 110) and two-arms (*k* = 125). They were recently published (median publication year of 2015, interquartile range [2008–2019]), had a short duration (12 [8–13] weeks), small sample sizes (40 [23–76]) and few sites (1 [1–3]), which were mainly academic (*k* = 102 trials had only academic sites).

The median age of participants was 8.2 [6.3–9.5] years in children/adolescents and 24.6 [21.9–27.9] years in adults. The overall male-to-female ratio was 5.3 [3.9–8.2]. Standardized diagnostic criteria were used in most of the studies (95%), and seven studies used only diagnostic evaluation tools. Associated symptoms were required as an inclusion criterion in about a third of the studies, mainly irritability and ADHD symptoms (in 30 trials), and a genetic syndrome (neurofibromatosis-type-I) in one trial [58]. At baseline, the sample was moderately to markedly ill with a CGI-S score of 4.8 [4.4–5.1], and ABC-Irritability of 16.9 [13.3–22.3], and about half of the participants had intellectual disability (50% [0–73.5%]). Nevertheless, reporting of participant characteristics was poor in about two thirds of the studies.

Risk of bias assessment is presented in Additional file 1: eAppendix-5.2. About 25% of the studies had an overall low risk of bias, 55% had moderate and 17% high. About half adequately reported methods of random sequence and allocation concealment, and blinding was adequately addressed in about 65%. High risk of bias was assigned in about 26% studies for incomplete outcome data, 36% for selective reporting and about 12% for other biases, mainly due to baseline imbalance or early trial termination. Finally, about 30% of the studies were funded by industry or their investigators applied for a patent.

**Fig. 1:**
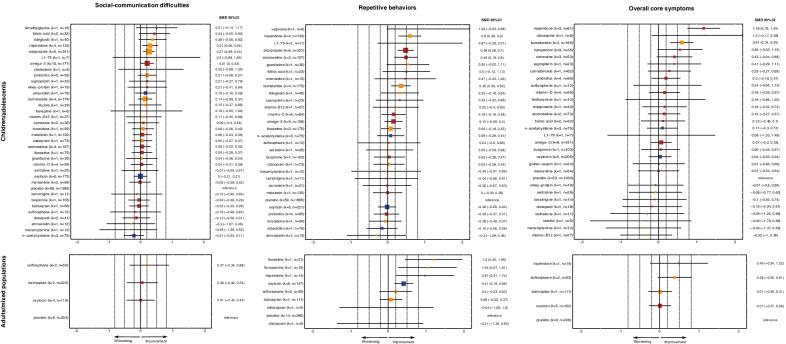
Forest plots of network meta-analysis for the primary outcomes, i.e., social-communication difficulties (SCD), repetitive behaviors (RB), and overall core symptoms (OCS), in children/adolescents and adults. Placebo was used as reference. The squares and bars represent the effect-sizes (standardized mean differences-SMD) along with their 95% confidence intervals. The size of the square is proportional to the inverse standard error of the effect size. The color represents confidence in the estimates as evaluated with the CINeMA framework, i.e., blue = moderate, yellow = low, and red = very low. SMDs > 0 indicate more improvement with the medication in comparison with placebo, SMDs = 0 indicate no difference between medication and placebo, and SMDs < 0 indicate less improvement with the medication in comparison with placebo. SMDs could be interpreted as small (SMD =|0.2|), medium (SMD =|0.5|) and large (SMD =|0.8|), and these thresholds are presented with dashed lines. *k* = total number of studies for the intervention; *n* = total number of participants on the intervention

Forty-one drugs were investigated in 100 trials (antipsychotics and antidepressants in about a third) and 17 dietary-supplements in 43 trials (Additional file 1: eAppendix-5.1). Interventions were connected in mainly star-shaped networks with placebo as the main node (Additional file 2: Fig. S1). Therefore, we focused on comparisons with placebo (Fig. 1, Additional file 3: Fig. S2), and league tables with all comparisons are presented in Additional file 4: Table S1. The results of pairwise meta-analyses and individual studies are presented in Additional file 5: Fig. S3. In addition, incoherence could not be evaluated when there were no closed loops (i.e., networks for anxiety/depression, quality of life, caregiver stress and all networks in adults). There was no clear indication of incoherence for the rest of the networks, except for irritability, response, weight gain and sedation in children/adolescents for which pairwise meta-analyses were conducted (Additional file 1: eAppendix-6.8).

### Primary outcomes

#### Social-communication difficulties (SCD)

Social-communication difficulties were measured mainly with ABC-L/SW (55%) and VABS-S (18%).

In children/adolescents, social-communication difficulties were improved by risperidone (*k* = 4 studies in the analysis, *n* = 133 participants treated with risperidone; SMD = 0.31 95%CI [0.06, 0.55]; *low* quality of evidence) and aripiprazole (*k* = 6, *n* = 341; SMD = 0.27 [0.09, 0.44]; *low*). Some trends of improvement were noted for folinic acid (*k* = 2, *n* = 32, SMD = 0.44 [− 0.05, 0.93]; *very low*), tideglusib (*k* = 1, *n* = 40; SMD = 0.38 [− 0.06, 0.82]; *low*), omega-3-fatty-acids (*k* = 10, *n* = 171; SMD = 0.21 [0.00, 0.43], *very low*), probiotics (*k* = 5, *n* = 92; SMD = 0.21 [− 0.08, 0.51]; *low*) and bumetanide (*k* = 4, *n* = 174; SMD = 0.14 [− 0.08, 0.37]; *low*). There were no clear differences between other medications and placebo with very low-to-moderate confidence. Heterogeneity was low (*τ*^2^ = 0).

In adults, none of the investigated medications (sulforaphane, balovaptan, oxytocin) improved social-communication difficulties with very-low- or low-quality evidence. There were high levels of heterogeneity (*τ*^2^ = 0.096).

#### Repetitive behaviors (RB)

Repetitive behaviors were measured mainly with ABC-S (47%) and YBOCS-versions (27%).

In children/adolescents, repetitive behaviors were improved by risperidone (*k* = 4, n = 133; SMD = 0.60 [0.29, 0.90]; *low*), aripiprazole (*k* = 6, *n* = 322; SMD = 0.48 [0.26, 0.70]; *very low*), atomoxetine (*k* = 3, *n* = 107; SMD = 0.49 [0.18, 0.80]; *very low*) and bumetanide (*k* = 4, *n* = 175; SMD = 0.35 [0.09, 0.62], *low*). There were trends for valproate (*k* = 1, *n* = 9; SMD = 1.33 [− 0.03, 2.68]; *very low*) and guanfacine (*k* = 1, *n* = 30; SMD = 0.55 [− 0.02, 1.11]; * low*), and no clear differences for other medications with very low-to-moderate confidence. Heterogeneity was low-to-moderate (*τ*^2^ = 0.017).

In adults, repetitive behaviors were improved by fluoxetine (*k* = 1, *n* = 21; SMD = 1.20 [0.45, 1.96]; *low*), fluvoxamine (*k* = 1, *n* = 15; SMD = 1.04 [0.27, 1.81]; *low*), risperidone (*k* = 1, *n* = 14; SMD = 0.97 [0.21, 1.74]; *very low*), and oxytocin (*k* = 6, *n* = 147; SMD = 0.41 [0.16, 0.66]; *moderate*). Sulforaphane, balovaptan, milnacipran and citalopram were not found efficacious with very low or low confidence. Heterogeneity was low (*τ*^2^ = 0).

#### Overall core symptoms (OCS)

Overall core symptoms were measured mainly with SRS (47%) and CARS (22%).

In children/adolescents, overall core symptoms were improved by risperidone (*k* = 3, *n* = 81; SMD = 1.18 [0.75, 1.61]; *very low*), and bumetanide (*k* = 4, *n* = 189; SMD = 0.61 [0.31, 0.91]; *low*). There were some trends for haloperidol (*k* = 3, *n* = 36; SMD = 0.56 [− 0.03, 1.15]; *very low*) and carnosine (*k* = 3, *n* = 53; SMD = 0.42 [− 0.04, 0.88]; *very low*), and no clear differences for other medications with very low-to-moderate confidence. There were moderate levels of heterogeneity (*τ*^2^ = 0.038) and no indication of incoherence. Nevertheless, a small study (*n* = 30) [59] that found no difference between risperidone and memantine (SMD = 0.00 [− 0.71, 0.72]) introduced incoherence and was excluded from the primary analysis of this outcome (Additional file 1: eAppendix-6.8), and the results were robust after inclusion of this study (Additional file 6: Fig. S4).

In adults, none of the investigated medications (risperidone, sulforaphane, balovaptan and oxytocin) found to be more efficacious than placebo in reducing overall core symptoms, though a trend was noted for sulforaphane (*k* = 2, *n* = 53; SMD = 0.38 [− 0.05, 0.81]; *low*). Confidence in evidence was very low or low. Heterogeneity was low (*τ*^2^ = 0).

#### Sensitivity analysis

The results did not materially change in sensitivity analyses (Additional file 1: eAppendix-6.6, Additional file 6: Fig. S4). There were some potential differences in omega-3-fatty-acids. Omega-3-fatty-acids did not reduce social-communication difficulties in children/adolescents when studies on associated symptoms were excluded (*k* = 6, *n* = 112, SMD = 0.05 [− 0.21, 0.32]) or when clinician-ratings were used (*k* = 3, *n* = 53, SMD = 0.03 [− 0.36, 0.42]). Yet, their effect-size was larger when ABC-L/SW was used (*k* = 6, *n* = 79, SMD = 0.45 [0.13, 0.77]). In addition, the results for some interventions, i.e., folinic acid, carnosine, vitamin-D, were not robust in sensitivity analyses, which were based on one or two small trials with potentially inflated effect-sizes.

#### Small-study effects and publications

There was asymmetry in funnel plots for social-communication difficulties in children/adolescents, indicating small-study effects (Additional file 1: eAppendix-6.8). Funnel plots for the other co-primary outcomes were inconclusive. Reporting bias was suspected for some medications, and quality of evidence was downgraded accordingly (Additional file 1: eAppendix-6.9).

### Secondary outcomes

#### Irritability

Irritability was measured mainly with ABC-I (83%).

In children/adolescents, there was evidence of incoherence (none of the closed loops were incoherent, but *p*-_design-by-treatment_ = 0.014) and pairwise meta-analysis were conducted. Irritability was improved by risperidone (*k* = 4 studies in the analysis, *n* = 138 participants treated with risperidone; SMD = 1.05 [0.76, 1.33], *τ*^2^ = 0.02), sulforaphane (*k* = 1, *n* = 12; SMD = 0.97 [0.12, 1.83]), aripiprazole (*k* = 5, *n* = 312; SMD = 0.63 [0.44, 0.82], *τ*^2^ = 0), and citalopram (*k* = 1, *n* = 73; SMD = 0.37 [0.04, 0.69]), as well as there was a trend for guanfacine (*k* = 1, *n* = 30; SMD = 0.50 [0.00, 1.01]) and riluzole (*k* = 1, *n* = 29; SMD = 0.43 [− 0.09, 0.95]). On the other hand, irritability was worsened by vitamin-B12 (*k* = 1, *n* = 27; SMD = − 0.62 [− 1.19, − 0.05]) and levetiracetam (*k* = 1, *n* = 10; SMD = -1.47 [− 2.48, − 0.46]).

In adults, risperidone was found efficacious (*k* = 1, *n* = 14; SMD = 1.19 [0.34, 2.04]), and heterogeneity was moderate (*τ*^2^ = 0.028).

#### ADHD symptoms

ADHD symptoms were measured in the majority of the studies with ABC-H (79%).

In children/adolescents, ADHD symptoms were improved by olanzapine (*k* = 1, *n* = 6; SMD = 2.08 [0.48, 3.68], *based only on indirect evidence*), guanfacine (*k* = 1, *n* = 30; SMD = 1.39 [0.73, 2.05]), aripiprazole (*k* = 7, *n* = 363; SMD = 0.82 [0.59, 1.05]), risperidone (*k* = 5, *n* = 155; SMD = 0.79 [0.47, 1.11]), naltrexone (*k* = 1, *n* = 23; SMD = 0.85 [0.12, 1.59]), and atomoxetine (*k* = 3, *n* = 107; SMD = 0.64 [0.30, 0.99]), as well as a trend was noted for sulforaphane (*k* = 1, *n* = 12; SMD = 0.88 [− 0.03, 1.80]). Heterogeneity was moderate (*τ*^2^ = 0.032).

In adults, none of the investigated medications were found efficacious for ADHD symptoms, and heterogeneity was low (*τ*^2^ = 0).

#### Anxiety/depressive symptoms

Different scales measured anxiety/depression in children/adolescents (e.g., CBCL-I, BASC-I, CASI, DBC-Anxiety), and STAI-state was used in half of the studies in adults. None of the investigated medications found to improve anxiety or depressive symptoms, except for a trend about risperidone in adults (*n* = 1, *k* = 14; SMD = 0.67 [− 0.07, 1.41]). There were moderate-to-high levels of heterogeneity in children/adolescents (*τ*^2^ = 0.041) and low in adults (*τ*^2^ = 0).

#### Caregiver stress

Caregiver stress was measured mainly with PSI (36%), CSQ (22%) and CGSQ (14%) in children/adolescents, and with PedsQL-Family Impact in adults. In children/adolescents, it was reduced by melatonin (*k* = 1, *n* = 54; SMD = 0.51 [0.12, 0.91]), and there were trends of small improvements by cannabinoids (*k* = 1, *n* = 80; SMD = 0.32 [− 0.06, 0.69]) and atomoxetine (*k* = 3, *n* = 104; SMD = 0.21 [− 0.06, 0.48]). There were no clear differences between other medications and placebo in both age groups, and heterogeneity was low (*τ*^2^ = 0).

#### Global functioning

Global functioning was measured with GAF or CGAS. In children/adolescents, it was improved by risperidone (*k* = 3, *n* = 62, SMD = 0.83 [0.40, 1.26]) and aripiprazole (*k* = 2, *n* = 69, SMD = 0.75 [0.33, 1.17]). No clear differences between other investigated medications and placebo were found in both age groups. Heterogeneity was moderate in children/adolescents (*τ*^2^ = 0.016) and low in adults (*τ*^2^ = 0).

#### Quality of life

Quality of life was measured with PedsQL in children/adolescents, and with PedsQL (40%) and WHO-QOL (60%) in adults. There were no clear differences between medications and placebo in children/adolescents. In adults, quality of life was improved by balovaptan (*k* = 2, *n* = 217; SMD = 0.22 [0.02, 0.43]), and potentially by oxytocin (*k* = 3, *n* = 41; SMD = 0.44 [− 0.02, 0.90]). Heterogeneity was low in both age groups (*τ*^2^ = 0).

#### Response

Pairwise meta-analyses were conducted in children/adolescents due to incoherence (50% of the closed loops were incoherent; *p*-_design-by-treatment_ = 0.068). In comparison with placebo, more participants responded with risperidone (*k* = 5, *n* = 161; OR = 11.33 [4.99, 25.70]; *τ*^2^ = 0.294), guanfacine (*k* = 1, *n* = 30; OR = 9.67 [2.41, 38.71]), whey-protein (*k* = 1, *n* = 22; OR = 4.56 [1.25, 16.63]), aripiprazole (*k* = 5, *n* = 317; OR = 4.26 [2.32, 7.83]; *τ*^2^ = 0.212), vitamin-B12 (*k* = 1, *n* = 28; OR = 3.83 [1.20, 12.28]), atomoxetine (*k* = 3, *n* = 109; OR = 3.18 [1.56, 6.48]; *τ*^2^ = 0), melatonin (*k* = 1, *n* = 60; OR = 3.06 [1.38, 6.77]), bumetanide (*k* = 3, *n* = 155; OR = 2.78 [1.48, 5.21]; *τ*^2^ = 0), and cannabinoids (*k* = 1, *n* = 100; OR = 2.56 [1.15, 5.70]), while fewer with oral human immunoglobulins (IGOH) (*k* = 1, *n* = 94; OR = 0.40 [0.16, 0.99]). There were no clear differences for other medications.

In adults, there were more responders with risperidone (*k* = 1, *n* = 15; OR = 37.40 [1.62, 865.22]) and fluvoxamine (*k* = 1, *n* = 15; OR = 35.13 [1.52, 814.72]. There were high levels of heterogeneity (*τ*^2^ = 0.257).

#### Dropouts due to any cause

In children/adolescents, fewer overall dropouts were noted with risperidone (*k* = 10, *n* = 274; OR = 0.38 [0.22, 0.65]), lurasidone (*k* = 1, *n* = 100; OR = 0.35 [0.14, 0.88]) and aripiprazole (*k* = 8, *n* = 399; OR = 0.46 [0.29, 0.75]), as well as potentially with melatonin (*k* = 4, *n* = 239; OR = 0.52 [0.26, 1.03]). More dropouts were observed with arbaclofen (*k* = 1, *n* = 76; OR = 3.39 [1.16, 9.88]), and a trend was noted for fluoxetine (*k* = 3, *n* = 161; OR = 1.59 [0.97, 2.58]). There were no clear differences for other medications, and there were some indications of incoherence (12.5% of the loops were incoherent; *p*-_design-by-treatment_ = 0.334). In adults, there were no clear differences for the investigated medications. Heterogeneity was low in both age groups (*τ*^2^ = 0.006 and *τ*^2^ = 0).

#### Dropouts due to adverse events

There were no clear differences between investigated medications and placebo in both age groups, and heterogeneity was low (*τ*^2^ = 0).

#### Any adverse event

In children/adolescents, more participants had adverse events with risperidone (*k* = 4, *n* = 123; OR = 4.74 [2.24, 10.04]), citalopram (*k* = 1, *n* = 73; OR = 5.38 [1.14, 25.46]), fluvoxamine (*k* = 1, *n* = 18; OR = 4.50 [1.02, 19.90]) and aripiprazole (*k* = 6, *n* = 348; OR = 2.62 [1.65, 4.15]), as well as potentially with guanfacine (*k* = 1, *n* = 30; OR = 17.94 [0.98, 329.56]) and lurasidone (*k* = 1, *n* = 100; OR = 1.92 [0.95, 3.90]). In adults, more participants had adverse events with risperidone (*k* = 1, *n* = 15; OR = 14.30 [2.19, 93.37]). There were no clear differences between other medications and placebo. Heterogeneity was low in children/adolescents (*τ*^2^ = 0) and moderate in adults (*τ*^2^ = 0.049).

#### Sedation

In children/adolescents, pairwise meta-analyses were conducted due to incoherence (75% of the closed loops were incoherent; *p*-_design-by-treatment_ = 0.051). More participants had sedation with guanfacine (*n* = 1, *k* = 30; OR = 62.83 [12.84, 307.45]), haloperidol (*n* = 1, *k* = 20; OR = 44.33 [4.78, 410.96]), risperidone (*n* = 4; *k* = 142, OR = 11.95 [5.86, 24.36], *τ*^2^ = 0), aripiprazole (*n* = 5, *k* = 317; OR = 3.56 [1.62, 7.86]; *τ*^2^ = 0) and melatonin (*n* = 1, *k* = 60; OR = 3.28 [1.25, 8.59]).

In adults, there were no clear differences, and heterogeneity was low (*τ*^2^ = 0).

#### Weight gain

In children/adolescents, there was evidence of incoherence (50% of the closed loops were incoherent; *p*-_design-by-treatment_ = 0.032) and pairwise meta-analyses were conducted. More participants had weight gain with aripiprazole (*n* = 5, *k* = 317; OR = 3.78 [2.09, 6.84], *τ*^2^ = 0) and risperidone (*n* = 5, *k* = 161; OR = 3.39 [1.80, 6.38], *τ*^2^ = 0) in comparison with placebo, while aripiprazole caused less weight gain in comparison with risperidone (*n* = 2, *k* = 104; OR = 0.22 [0.09, 0.55], *τ*^2^ = 0.045). There were no clear differences between other medications.

In adults, none of the investigated medications (sulforaphane, oxytocin and balovaptan) was associated with weight gain, and heterogeneity was low (*τ*^2^ = 0).

#### Extrapyramidal symptoms

The network of children/adolescents was disconnected; therefore, pairwise meta-analyses were conducted. In comparison with placebo, more participants had extrapyramidal symptoms with risperidone (*n* = 4, *k* = 142; OR = 3.02 [1.22, 7.48]; *τ*^2^ = 0) and aripiprazole (*n* = 4, *k* = 300; OR = 2.38 [1.18, 4.77]; *τ*^2^ = 0).

There were no data available for adults.

## Discussion

This is the first comprehensive network meta-analysis on pharmacological and dietary-supplement interventions for ASD. Pediatric and adult populations were analyzed separately, in order to avoid misleading extrapolations [52]. Core symptom domains (SCD and RB) were also examined separately as co-primary outcomes, since differential treatment responses can be expected [52]. In addition, scales that measure overall core symptoms (OCS) in single scores were considered as a distinct outcome. Associated symptoms and side-effects were also investigated as secondary outcomes. Therefore, our analysis provides a more comprehensive synthesis of evidence in comparison with previous reviews that were mainly focused on pediatric populations, certain symptoms or specific medications, or did not utilize a network meta-analysis [9–17, 19, 21, 23, 24].

Our review identified the following medications that could improve at least one core symptom domain: aripiprazole (SCD, RB), atomoxetine (RB), bumetanide (RB, OCS) and risperidone (SCD, RB, OCS) in children/adolescents; fluoxetine (RB), fluvoxamine (RB), oxytocin (RB) and risperidone (RB) in adults. In addition, there were some indications of improvement by carnosine, haloperidol, folinic acid, guanfacine, omega-3-fatty-acids, probiotics, sulforaphane, tideglusib and valproate, yet they were imprecise based on limited data and not formally statistically significant, as well as not robust in sensitivity analysis.

### Summary of evidence

#### Commonly used medications

**Fig. 2:**
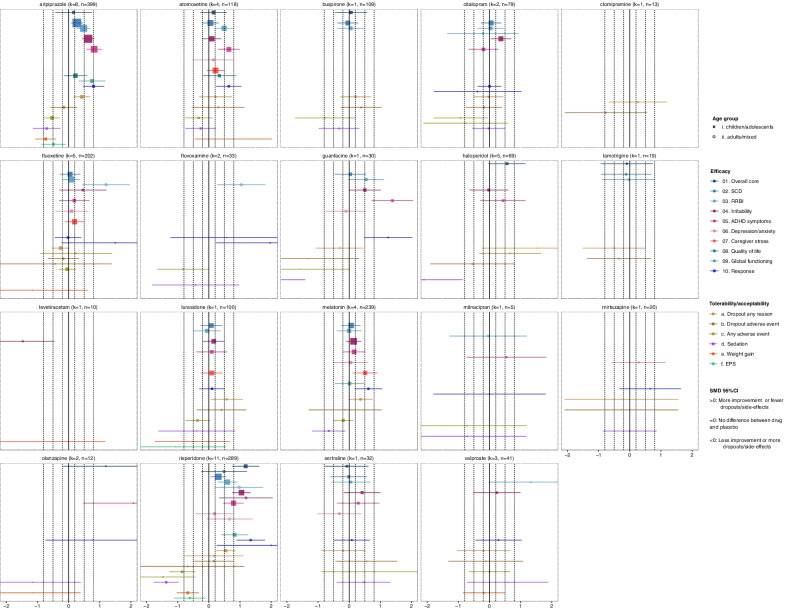
Summary forest plots for commonly used medications, i.e., antipsychotics, ADHD medications, antiepileptic/mood-stabilizers, sleep medications. Effect-sizes (standardized mean differences—SMDs and their 95% confidence intervals) of comparisons with placebo are presented for each medication, outcome and age group. SMDs are presented with squares in children/adolescents and circles in adults, and their size is proportional to the inverse standard error of the effect size. For dichotomous outcomes (response, dropouts due to any cause or adverse event, any adverse event, sedation, weight gain, extrapyramidal symptoms), odds ratios were converted to SMDs. The results are based on network meta-analysis, except for irritability, response, sedation, weight gain and extrapyramidal symptoms (EPS) in children/adolescents, since pairwise meta-analyses were conducted due to incoherence or disconnected networks. SMDs > 0 indicate more improvement or fewer dropouts/adverse events with the medication in comparison with placebo, SMDs = 0 indicate no difference between medication and placebo, and SMDs < 0 indicate less improvement or more dropouts/adverse events with the medication in comparison with placebo. SMDs could be interpreted as small (SMD =|0.2|), medium (SMD =|0.5|) and large (SMD =|0.8|), and these thresholds are presented with dashed lines. There were no usable data for methylphenidate, and effect-sizes for this drug are not presented. *k* = total number of studies for the intervention with data for at least an outcome and age group, *n* = total number of participants on the intervention with data for at least an outcome and age group. *EPS* extrapyramidal symptoms, *RB* repetitive behaviors, *SCD* social-communication difficulties

Currently, no medication is approved for the core symptoms of ASD [39]. However, about half of the individuals with ASD receive psychotropic drugs, mainly for associated symptoms, such as antipsychotics (median prevalence of 18.1%), ADHD medications (16.6%), antidepressants (17.2%), antiepileptics/mood-stabilizers and sleep medication [8]. Findings of our analysis on these medications are summarized in Fig. 2, facilitating intuitive understanding of the current evidence.

Among antipsychotics, aripiprazole and risperidone demonstrated medium-to-large effect-sizes in reducing irritability and ADHD symptoms, while smaller improvements were found in social-communication difficulties and repetitive behaviors. On the other hand, lurasidone was in general not efficacious, and there were only a few data available for olanzapine and haloperidol, and for adults. Antipsychotics were also associated with more adverse events, sedation, weight gain and extrapyramidal symptoms. Nevertheless, reporting bias was suspected (Additional file 1: eAppendix-6.8), e.g., two pediatric studies found that risperidone did not improve social-communication difficulties as measured with ABC-L/SW, yet there were no usable data for this analysis [60, 61]. In addition, trials on antipsychotics were conducted mainly in participants with irritability. As a result, improvements in core symptoms could be collateral to the reduction in interfering challenging behaviors that can subsequently allow participation in social interactions [62]. In other words, antipsychotics may not have direct effects on core symptoms, but rather secondary to the reduction in irritability. Trials focusing on core symptoms are sparse, and data from a small trial (*n* = 41) investigating risperidone for repetitive behaviors are not yet reported [63]. Therefore, evidence was downrated due to indirectness and reporting bias (Additional file 1: eAppendix-6.9).

Among ADHD medications, atomoxetine and guanfacine improved ADHD symptoms and potentially repetitive behaviors, but not social-communication difficulties. Guanfacine was also associated with more adverse events and sedation. A causal-mediation analysis suggested a causal link from hyperactivity to repetitive behaviors and from impulsivity/inattention to social-communication difficulties in ASD [64]. Therefore, and since these drugs were investigated in participants with ADHD symptoms, improvements in repetitive behaviors could be indirect and subsequent to the reduction in hyperactivity. Of note, there were no usable data for methylphenidate, since none of the five crossover trials reported usable data from the first phase (Additional file 1: eAppendix-6.8), and none of the ADHD medications were investigated in adults.

Antidepressants and buspirone were not found efficacious for core or associated symptoms in children/adolescents, except citalopram that improved irritability with a small-to-medium effect-size. Citalopram, fluvoxamine and fluoxetine were also associated with more adverse events or dropouts. In adults, however, fluoxetine and fluvoxamine improved repetitive behaviors with large effect-sizes, yet based on single small (*n* = 30–37) studies [65, 66]. Apart from the limited data for adults, such differences might be explained by different study designs, participant characteristics and age-dependent variability in treatment response [67, 68].

Antiepileptics/mood-stabilizers were in general not efficacious based on limited and very low-quality data. A single small study (*n* = 13) suggested efficacy for valproate [69], yet there was reporting bias and two additional studies did not report appropriate data [70, 71] (Additional file 1: eAppendix-6.8). Of note, levetiracetam worsened irritability with a large effect-size in a small study (*n* = 12) [72], in accordance with the well-documented behavioral side-effects of this drug [73]. Last, melatonin was not efficacious for core or associated symptoms, yet it decreased caregiver stress and increased the number of responders. Such beneficial effects could be collateral to the reduction in sleep problems [2, 74, 75]. Sleep outcomes were not investigated in this review, but our findings support its sedative effects.

#### Experimental medications

**Fig. 3:**
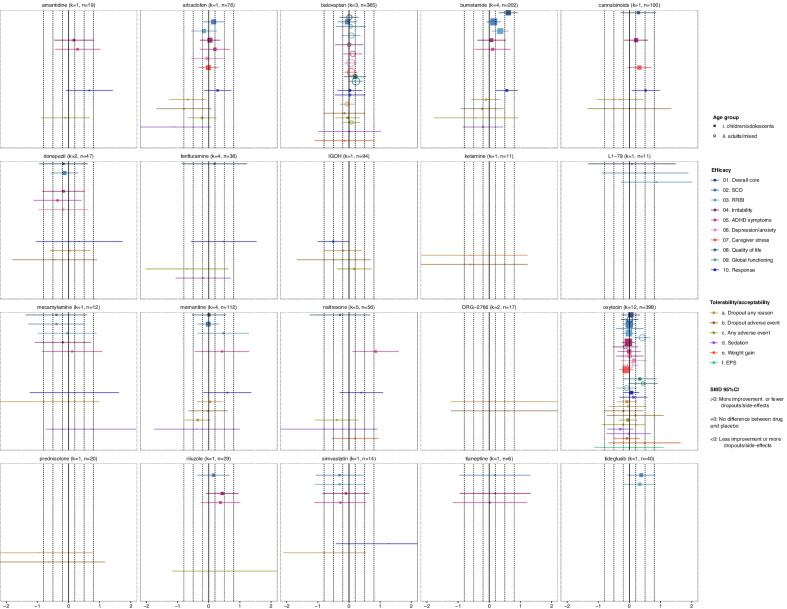
Summary forest plots for experimental medications. Effect-sizes (standardized mean differences—SMDs and their 95% confidence intervals) of comparisons with placebo are presented for each medication, outcome and age group. SMDs are presented with squares in children/adolescents and circles in adults, and their size is proportional to the inverse standard error of the effect size. For dichotomous outcomes (response, dropouts due to any cause or adverse event, any adverse event, sedation, weight gain, extrapyramidal symptoms), odds ratios were converted to SMDs. The results are based on network meta-analysis, except for irritability, response, sedation, weight gain and extrapyramidal symptoms (EPS) in children/adolescents, since pairwise meta-analyses were conducted due to incoherence or disconnected networks. SMDs > 0 indicate more improvement or fewer dropouts/adverse events with the medication in comparison with placebo, SMDs = 0 indicate no difference between medication and placebo, and SMDs < 0 indicate less improvement or more dropouts/adverse events with the medication in comparison with placebo. SMDs could be interpreted as small (SMD =|0.2|), medium (SMD =|0.5|) and large (SMD =|0.8|), and these thresholds are presented with dashed lines. There were no usable data for dextromethorphan/quinidine and effect-sizes for this drug are not presented. *k* = total number of studies for the intervention with data for at least an outcome and age group; *n* = total number of participants on the intervention with data for at least an outcome and age group; EPS: extrapyramidal symptoms, IGOH: oral human immunoglobulin; RB: repetitive behaviors; SCD: social-communication difficulties

Our review identified a considerable number of experimental medications (Fig. 3) with diverse mechanisms of action, which discussion is out of the scope of this review (e.g., see [39, 76–79]). The majority of them were investigated exclusively in children/adolescents, except for oxytocin and balovaptan.

Oxytocin and balovaptan (vasopressin-V_1A_ receptor antagonist) were not efficacious in children/adolescents, based on substantial evidence from large trials, e.g., (*n* = 290–339) [80, 81]. In adults, however, oxytocin improved repetitive behaviors with small-to-medium effect-sizes and moderate-quality evidence. This finding needs replication, since studies were mainly focused on high-functioning participants and variability in treatment response due to age-dependent differences in the oxytocin system cannot be excluded [82, 83]. Balovaptan was not found efficacious in adults based on two large studies (*n* = 223–322) [84, 85], yet small improvements in quality of life were noted. Of note, intranasal vasopressin was efficacious in a small trial (*n* = 30) [86], which was, however, excluded from our analysis due to unconcealed allocation (Additional file 1: eAppendix-4.2.).

Bumetanide (loop-diuretic that may enhance GABAergic inhibition) was found to improve repetitive behaviors and overall core symptoms with small-to-medium effect-sizes, but not social-communication difficulties. However, two large phase-III trials (*n* = 422 in total) [87] were negative and prematurely terminated [88], yet they did not report usable data, and therefore, evidence was downrated due to reporting bias. Other experimental medications were not found efficacious based on current data. There were some indications for cannabinoids (more participants had a positive response), and naltrexone (improvement of ADHD symptoms), yet they were based on single studies [89, 90] and there was also reporting bias for naltrexone (Additional file 1: eAppendix-6.8). On the other hand, arbaclofen (GABA_B_ agonist) was associated with more dropouts and IGOH (oral human immunoglobulin) with fewer responders. Nevertheless, several trials are ongoing, e.g., for arbaclofen [91, 92], memantine [93] and cannabinoids [94, 95]. In addition, the findings on tideglusib (GSK-3β inhibitor), L1-79 (tyrosine hydroxylase inhibitor) and riluzole could be imprecise, since data from abstracts were used [96–98].

#### Dietary-supplements

**Fig. 4:**
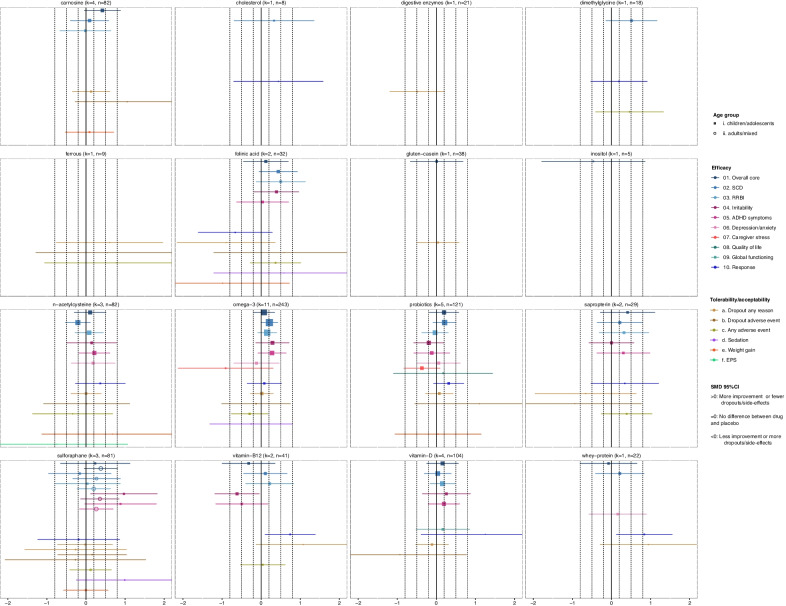
Summary plots for dietary-supplements. Effect-sizes (standardized mean differences—SMDs and their 95% confidence intervals) of comparisons with placebo are presented for each medication, outcome and age group. SMDs are presented with squares in children/adolescents and circles in adults, and their size is proportional to the inverse standard error of the effect size. For dichotomous outcomes (response, dropouts due to any cause or adverse event, any adverse event, sedation, weight gain, extrapyramidal symptoms), odds ratios were converted to SMDs. The results are based on network meta-analysis, except for irritability, response, sedation, weight gain and extrapyramidal symptoms (EPS) in children/adolescents, since pairwise meta-analyses were conducted due to incoherence or disconnected networks. SMDs > 0 indicate more improvement or fewer dropouts/adverse events with the medication in comparison with placebo, SMDs = 0 indicate no difference between medication and placebo, and SMDs < 0 indicate less improvement or more dropouts/adverse events with the medication in comparison with placebo. SMDs could be interpreted as small (SMD =|0.2|), medium (SMD =|0.5|) and large (SMD =|0.8|), and these thresholds are presented with dashed lines. There were no usable data for pyridoxine, and effect-sizes for this dietary-supplement are not presented. *k* = total number of studies for the intervention with data for at least an outcome and age group; *n* = total number of participants on the intervention with data for at least an outcome and age group. *EPS* extrapyramidal symptoms, *IGOH* oral human immunoglobulin, *RB* repetitive behaviors, *SCD* social-communication difficulties

The efficacy of dietary-supplements was inconclusive (Fig. 4). Omega-3-fatty-acids could potentially improve social-communication difficulties with small effect-sizes, based on very low-quality evidence from ten studies in children/adolescents. Similarly, there were some trends for carnosine, folinic acid and probiotics, based on fewer data. Nevertheless, these findings were highly heterogeneous (for carnosine and folinic acid), imprecise and not statistically significant (at two-sided alpha 0.05), and not robust in sensitivity analyses. Therefore, results from larger trials are warranted, e.g. [99, 100]. There was also mixed evidence about sulforaphane (broccoli sprout extract), since findings were based on one inconclusive trial (*n* = 45) in children/adolescents [101], and two contradicting trials (*n* = 44–48) in adults [102, 103], while usable data from a larger trial (*n* = 110) are not yet reported [104]. In addition, there were some indications from single studies for cysteine-rich whey-protein [105] and vitamin-B12 [106], since both increased the number of responders but were not found to be efficacious for core or associated symptoms. On the contrary, vitamin-B12 worsened irritability with a medium effect-size, which is in line with a meta-analysis of prevalence that identified its potential behavioral side-effects [107]. Therefore, the safety of dietary-supplements should not be overlooked.

## Limitations

There are certain limitations. First, and in contrast with other fields of psychopharmacology, evidence base of ASD is flooded by small trials focusing on associated symptoms and investigating a plethora of medication classes, for which adequate dosing or duration of treatment is still unclear, and some of them have not yet investigated in RCTs. This reflects the two main approaches that guide psychopharmacology in ASD, by re-purposing psychotropics for associated symptoms or by targeting neurobiological processes [2]. Nevertheless, ASD may not be a unitary diagnosis in terms of neurobiology, according to its heterogeneity and lack of biomarkers. Therefore, it is likely that there is substantial interpersonal variability of treatment response across medications. Individual-participant-data meta-analyses could further explore this issue and investigate the potential impact of participant-level covariates [108], e.g., age, sex, baseline severity of core and associated symptoms. In that direction, there are also efforts to disentangle the neurobiology of subgroups within ASD in order to facilitate biomarker stratification and more targeted treatments [39, 77].

Second, clinical trials in ASD could be prone to substantial placebo responses and a lower ability to detect efficacy, which may be increased with adequately powered trials, rigorous selection of participants and careful selection of outcome measures [4]. In line with this, there is lack of consensus on outcome measures [39], and different scales are often used. We accepted a wide range of validated scales in order to incorporate more evidence, yet we preferred recommended and commonly used scales in order to obtain comparable measures (Additional file 1: eAppendix-5.3). As a result, data for most of the outcomes were derived mainly from one or two scales (Additional file 1: eAppendix-5.1), which treatment effects might agree in most cases, e.g., as suggested between CYBOCS and ABC-S [39]. The results were also generally robust in sensitivity analyses when clinician-ratings or when ABC subscales were used, except for some potential differences in omega-3-fatty-acids (Additional file 1: eAppendix-6.6, Additional file 6: Fig. S4). Nevertheless, further research is needed, since scales with different psychometric properties, e.g., sensitivity to change or susceptibility to placebo effects, could demonstrate discordant treatment effects. For example, a trial found low-dose buspirone to improve repetitive behaviors as measured with ADOS-RRB and RBS, but not with CYBOCS [109], which was preferred in our analysis according to our hierarchy (Additional file 1: eAppendix-5.3).

Third, there were limited data for adults, some medications, e.g., methylphenidate, and secondary outcomes, e.g., anxiety/depressive symptoms, which are, however, considered one of the top research priorities [110, 111]. Fourth, our analysis was mainly based on star-shaped networks of placebo-controlled comparisons and only a few medications were investigated in more than one or two trials, often with small sample sizes. Therefore, heterogeneity and incoherence could be masked, due to the low statistical power of their tests. Small-study effects could also be masked, since comparison-adjusted funnel plots should be interpreted with great caution when there are a few trials per comparison. Fifth, transitivity assumption could not be adequately assessed, since effect-modifiers are still unclear and insufficiently reported in clinical trials. Therefore, and despite of ordering treatments by their ranking in forest plots, indirect evidence, treatment hierarchies and league tables should be interpreted with great caution. There was also evidence of incoherence in irritability, response, sedation and weight gain in children/adolescents; therefore, pairwise meta-analyses were conducted for these outcomes. In addition, about half of the studies stated to be randomized without an exact description of the randomization method, yet the results did not materially change in sensitivity analyses when studies with an unclear risk of bias in random sequence generation or allocation concealment were excluded (Additional file 1: eAppendix-6.6, Additional file 6: Fig. S4).

Last, a comprehensive review of tolerability was beyond the scope of the manuscript, yet we examined dropouts and important side-effects that overlap among drug classes, i.e., sedation, weight gain and extrapyramidal symptoms, and our findings are in line with the literature [112]. Nevertheless, medications with different mechanisms of action can have unique side-effect profiles, e.g., bumetanide as a loop-diuretic can cause diuresis and hypokalemia [113]. Individuals with ASD may also be more sensitive to side-effects in comparison with neurotypical individuals [2]. Therefore, medications should be used after careful consideration and monitoring of their safety [2], as well as at low doses, since a therapeutic window could be expected, e.g., for risperidone [114].

## Conclusions

In conclusion, there was evidence that some medications could improve social-communication difficulties and/or repetitive behaviors in children/adolescents: aripiprazole, atomoxetine, bumetanide, and risperidone; while some medications could improve repetitive behaviors in adults: fluoxetine, fluvoxamine, oxytocin and risperidone. A large part of the evidence consisted of small RCTs (median 40 participants) with a short duration (median 12 weeks) and limited generalizability. Therefore, current commonly used medications, i.e., antipsychotics and ADHD medications, can be used for associated symptoms as indicated, and smaller improvements in core symptoms could also be expected, at least collaterally to the improvement of challenging behaviors. These medications are associated with side-effects, and therefore, they should be prescribed only after careful consideration and monitoring of their benefit-risk ratio. Evidence on the efficacy and safety for other medications, including bumetanide, oxytocin and some dietary-supplements, is at best preliminary and warrants further investigation. In line with the limitations of our review, there are current efforts to advance clinical psychopharmacology in ASD (e.g., within the AIMS-2-Trials consortium or the ISCTM/ECNP ASD working group), first with the elucidation of its neurobiology and the development of more targeted medications, second with the use of appropriate scales for measuring core symptoms, and third with well-designed and adequately powered clinical trials [39, 77].

## Supplementary Information

**Additional file 1.** eAppendix.

**Additional file 2. Fig. S1.** Network plots.

**Additional file 3. Fig. S2.** Forest plots for secondary outcomes.

**Additional file 4. Table S1.** League tables.

**Additional file 5. Fig. S3.** Forest plots for pairwise meta-analysis and individual studies.

**Additional file 6. Fig. S4.** Sensitivity analyses.

**Additional file 7.** Dataset.

## Data Availability

The dataset used in the current study is available in this published article and its Additional file 7: Dataset.

## Abbreviations

95% CI: 95% Confidence intervals
ASD: Autism spectrum disorder
ABC-H: Aberrant Behavior Checklist—Hyperactivity/noncompliance
ABC-I: Aberrant Behavior Checklist—Irritability
ABC-L/SW: Aberrant Behavior Checklist—Lethargy/Social-Withdrawal
ABC-S: Aberrant Behavior Checklist—Stereotypic behavior
ADHD: Attention Deficit Hyperactivity Disorder
ADI-R: Autism Diagnostic Interview-Revised
ADOS-RRB: Autism Diagnostic Observation Schedule—Restricted and Repetitive Behaviors
BASC-I: Behavior Assessment System for Children—Internalizing
CARS: Childhood Autism Rating Scale
CASI: Childhood Anxiety Sensitivity Index
CBCL-I: Child Behavior Checklist—Internalizing
CGAS: Children Global Assessment Scale
CGI: Clinical Global Impression
CGSQ: Caregiver Strain Questionnaire
CINeMA: Confidence in Network Meta-Analysis
CSQ: Client Satisfaction Questionnaire
CYBOCS(-PDD): Children's Yale-Brown Obsessive Compulsive Scale (Modified for Pervasive Developmental Disorders)
DBC-Anxiety: Developmental Behavior Checklist—Anxiety
DSM: Diagnostic and Statistical Manual of Mental Disorders
GAF: Global Assessment of Functioning
FDA: Food and Drug Administration
ICD: International Classification of Diseases
IGOH: Oral human immunoglobulin
IQR: Interquartile range
OCS: Overall core symptoms
OR: Odds ratio
LOCF: Last-observation carried forward
MMRM: Mixed-models for repeated measures
PedsQL: Pediatric Quality of Life Inventory
PSI: Parental Stress Index
RB: Repetitive Behaviors
RBS: Repetitive Behavior Scale
RCT: Randomized Controlled Trials
SCD: Social Communication difficulties
SD: Standard deviation
SMD: Standardized Mean Differences
SRS: Social Responsiveness Scale
STAI: State-Trait Anxiety Inventory
VABS: Vineland Adaptive Behavior Scale
WHO-QoL: World Health Organization-Quality of Life
