# Supplementary material for: Pharmacological and dietary-supplement treatments for autism spectrum disorder: a systematic review and network meta-analysis
Source: Mol Autism. 2022 Mar 4;13:10. doi: 10.1186/s13229-022-00488-4 (PMC8896153; doi:10.1186/s13229-022-00488-4)
Supplement: Supplementary file 1 — Additional file 1. eAppendix. [file 13229_2022_488_MOESM1_ESM.pdf]

Appendix:
Pharmacological and dietary supplement treatments for autism spectrum disorder: a systematic review and network meta-analysis

Spyridon Siafis, MD;[1] Oğulcan Çıray, MD;[2] Hui Wu, MD;[1] Johannes Schneider-Thoma, MD;[1] Irene Bighelli, PhD;[1] Marc Krause, Dr. rer. biol. hum.;[3] Alessandro Rodolico, MD;[4] Anna Ceraso, MD;[5] Giacomo Deste, MD;[5] Maximilian Huhn, MD;[1,6] David Fraguas, MD, PhD;[7] Antonia San José Cáceres, PhD;[8] Dimitris Mavridis, PhD;[9, 10] Tony Charman, PhD;[11] Declan G Murphy, MD, PhD;[12] Mara Parellada, MD, PhD;[9,13] Celso Arango, MD, PhD;[9,13] Stefan Leucht, MD[1]

[1]Department of Psychiatry and Psychotherapy, School of Medicine, Technical University of Munich, Munich, Germany; [2]Mardin State Hospital, Department of Child And Adolescent Psychiatry, Artuklu, Mardin, Turkey; [3]Department of Psychiatry and Psychotherapy, School of Medicine, Technical University of Munich, Munich, Germany (affiliated up to March 2019); [4]Department of Experimental and Clinical Medicine, Psychiatric Clinic University Hospital 'Gaspare Rodolico', University of Catania, Catania, Italy; [5]Department of Psychiatry, Spedali Civili Hospital, Brescia, Italy; [6]Department of Psychiatry, Psychosomatic Medicine and Psychotherapy Social Foundation Bamberg, Teaching Hospital of the University of Erlangen, Germany; [7]Institute of Psychiatry and Mental Health, Hospital Clínico San Carlos, IdISSC CIBERSAM, School of Medicine, Universidad Complutense, Madrid, Spain; [8]Department of Child and Adolescent Psychiatry, Institute of Psychiatry and Mental Health, Hospital General Universitario Gregorio Marañón, Madrid, Spain;[9]Department of Primary Education, University of Ioannina, Ioannina, Greece; [10]Faculté de Médecine, Université Paris Descartes, Paris, France; [11]Department of Psychology, Institute of Psychiatry, Psychology & Neuroscience, King's College London, London, UK; [12]Department of Forensic and Neurodevelopmental Sciences, Institute of Psychiatry, Psychology & Neuroscience, King's College London, London, UK; [13]School of Medicine, Universidad Complutense, Madrid, Spain

Corresponding author:
Spyridon Siafis,
Department of Psychiatry and Psychotherapy, School of Medicine, Technical University of Munich, Ismaningerstr. 22, 81675 Munich, Germany
Tel: +498941406415, Fax: +498941404888, e-mail: spyridon.siafis@tum.de

Contents:

eAppendix-1 PRISMA NMA checklist
eAppendix-2 Protocol and methods
eAppendix-3 Database search strategy
eAppendix-4 Study selection
eAppendix-5 Study characteristics
eAppendix-6 Results

eAppendix-1 PRISMA NMA checklist

1.1 PRISMA NMA checklist 3

1.2 References 7

1.1 PRISMA NMA checklist

PISMA NMA checklist according to Hutton et al 2015 [1].

Section/Topic	Item #	Checklist Item	Reported on Page #
**TITLE**			
Title	1	Identify the report as a systematic review *incorporating a network meta-analysis (or related form of meta-analysis).*	1
**ABSTRACT**			
Structured summary	2	Provide a structured summary including, as applicable:<br>**Background:** main objectives<br>**Methods:** data sources; study eligibility criteria, participants, and interventions; study appraisal; and *synthesis methods, such as network meta-analysis.*<br>**Results:** number of studies and participants identified; summary estimates with corresponding confidence/credible intervals; *treatment rankings may also be discussed. Authors may choose to summarize pairwise comparisons against a chosen treatment included in their analyses for brevity.*<br>**Discussion/Conclusions:** limitations; conclusions and implications of findings.<br>**Other:** primary source of funding; systematic review registration number with registry name.	3-4
**INTRODUCTION**			
Rationale	3	Describe the rationale for the review in the context of what is already known*, including mention of why a network meta-analysis has been conducted.*	5
Objectives	4	Provide an explicit statement of questions being addressed, with reference to participants, interventions, comparisons, outcomes, and study design (PICOS).	5
**METHODS**			
Protocol and registration	5	Indicate whether a review protocol exists and if and where it can be accessed (e.g., Web address); and, if available, provide registration information, including registration number.	6, eAppendix-2
Eligibility criteria	6	Specify study characteristics (e.g., PICOS, length of follow-up) and report characteristics (e.g., years considered, language, publication status) used as criteria for eligibility, giving rationale. *Clearly describe eligible treatments included in the*	6-7, eAppendix-2

		treatment network, and note whether any have been clustered or merged into the same node (with justification).	
Information sources	7	Describe all information sources (e.g., databases with dates of coverage, contact with study authors to identify additional studies) in the search and date last searched.	6-7, eAppendix-3
Search	8	Present full electronic search strategy for at least one database, including any limits used, such that it could be repeated.	eAppendix-3
Study selection	9	State the process for selecting studies (i.e., screening, eligibility, included in systematic review, and, if applicable, included in the meta-analysis).	6-7, eAppendix-2
Data collection process	10	Describe method of data extraction from reports (e.g., piloted forms, independently, in duplicate) and any processes for obtaining and confirming data from investigators.	6-7, eAppendix-2, eAppendix-4
Data items	11	List and define all variables for which data were sought (e.g., PICOS, funding sources) and any assumptions and simplifications made.	6-8, eAppendix-2
**Geometry of the network**	**S1**	Describe methods used to explore the geometry of the treatment network under study and potential biases related to it. This should include how the evidence base has been graphically summarized for presentation, and what characteristics were compiled and used to describe the evidence base to readers.	n.i.
Risk of bias within individual studies	12	Describe methods used for assessing risk of bias of individual studies (including specification of whether this was done at the study or outcome level), and how this information is to be used in any data synthesis.	6-7, 9
Summary measures	13	State the principal summary measures (e.g., risk ratio, difference in means). *Also describe the use of additional summary measures assessed, such as treatment rankings and surface under the cumulative ranking curve (SUCRA) values, as well as modified approaches used to present summary findings from meta-analyses.*	8
Planned methods of analysis	14	Describe the methods of handling data and combining results of studies for each network meta-analysis. This should include, but not be limited to:<br>• *Handling of multi-arm trials;*<br>• *Selection of variance structure;*<br>• *Selection of prior distributions in Bayesian analyses; and*<br>• *Assessment of model fit.*	6, 8-9
**Assessment of Inconsistency**	**S2**	Describe the statistical methods used to evaluate the agreement of direct and indirect evidence in the treatment network(s) studied. Describe efforts taken to address its presence when found.	9

Risk of bias across studies	15	Specify any assessment of risk of bias that may affect the cumulative evidence (e.g., publication bias, selective reporting within studies).	9, eAppendix-9
Additional analyses	16	Describe methods of additional analyses if done, indicating which were pre-specified. This may include, but not be limited to, the following:<br>• Sensitivity or subgroup analyses;<br>• Meta-regression analyses;<br>• *Alternative formulations of the treatment network; and*<br>• *Use of alternative prior distributions for Bayesian analyses (if applicable).*	9
**RESULTS†**			
Study selection	17	Give numbers of studies screened, assessed for eligibility, and included in the review, with reasons for exclusions at each stage, ideally with a flow diagram.	eAppendix-4
**Presentation of network structure**	**S3**	Provide a network graph of the included studies to enable visualization of the geometry of the treatment network.	Figure-S1
**Summary of network geometry**	**S4**	Provide a brief overview of characteristics of the treatment network. This may include commentary on the abundance of trials and randomized patients for the different interventions and pairwise comparisons in the network, gaps of evidence in the treatment network, and potential biases reflected by the network structure.	11
Study characteristics	18	For each study, present characteristics for which data were extracted (e.g., study size, PICOS, follow-up period) and provide the citations.	10-1, eAppendix-5, eAppendix-4.3
Risk of bias within studies	19	Present data on risk of bias of each study and, if available, any outcome level assessment.	eAppendix-5.2
Results of individual studies	20	For all outcomes considered (benefits or harms), present, for each study: 1) simple summary data for each intervention group, and 2) effect estimates and confidence intervals. *Modified approaches may be needed to deal with information from larger networks.*	Figure-S3
Synthesis of results	21	Present results of each meta-analysis done, including confidence/credible intervals. *In larger networks, authors may focus on comparisons versus a particular comparator (e.g. placebo or standard care), with full findings presented in an appendix. League tables and forest plots may be considered to summarize pairwise comparisons.* If	11-18, Figure-1, Figure-S2, Figure-2/3/4, Table-S1

		additional summary measures were explored (such as treatment rankings), these should also be presented.	
**Exploration for inconsistency**	**S5**	Describe results from investigations of inconsistency. This may include such information as measures of model fit to compare consistency and inconsistency models, *P* values from statistical tests, or summary of inconsistency estimates from different parts of the treatment network.	11, 13, 16, eAppendix-6.5
Risk of bias across studies	22	Present results of any assessment of risk of bias across studies for the evidence base being studied.	11-13, eAppendix-6.8/9
Results of additional analyses	23	Give results of additional analyses, if done (e.g., sensitivity or subgroup analyses, meta-regression analyses*, alternative network geometries studied, alternative choice of prior distributions for Bayesian analyses,* and so forth).	eAppendix-6.6, Figure-S4
**DISCUSSION**			
Summary of evidence	24	Summarize the main findings, including the strength of evidence for each main outcome; consider their relevance to key groups (e.g., healthcare providers, users, and policy-makers).	19-23
Limitations	25	Discuss limitations at study and outcome level (e.g., risk of bias), and at review level (e.g., incomplete retrieval of identified research, reporting bias). *Comment on the validity of the assumptions, such as transitivity and consistency. Comment on any concerns regarding network geometry (e.g., avoidance of certain comparisons).*	24-25
Conclusions	26	Provide a general interpretation of the results in the context of other evidence, and implications for future research.	26
**FUNDING**			
Funding	27	Describe sources of funding for the systematic review and other support (e.g., supply of data); role of funders for the systematic review. This should also include information regarding whether funding has been received from manufacturers of treatments in the network and/or whether some of the authors are content experts with professional conflicts of interest that could affect use of treatments in the network.	36

PICOS = population, intervention, comparators, outcomes, study design.
* Text in italics indicates wording specific to reporting of network meta-analyses that has been added to guidance from the PRISMA statement.
† Authors may wish to plan for use of appendices to present all relevant information in full detail for items in this section.
n.i.: not indicated

1.2 References

1. Hutton B, Catala-Lopez F, Moher D. The PRISMA statement extension for systematic reviews incorporating network meta-analysis: PRISMA-NMA. Med Clin (Barc). 2016;147(6):262-6.

eAppendix-2 Protocol and methods

2.1. PROSPERO protocol 9

2.1.1 Second version of the protocol (submitted to PROSPERO on 31.03.2020, online on 29.10.2020) 9

2.1.2 First version of the protocol (15.03.2019) 15

2.2 Methods clarifications and post-hoc decisions 23

2.3 References 26

2.1. PROSPERO protocol

This manuscript refers to the second objective of the PROSPERO protocol below, i.e. network meta-analysis to investigate the comparative efficacy and tolerability of pharmacological and dietary supplement interventions for ASD. The analysis of the first objective, i.e. placebo response, is reported elsewhere [1].

2.1.1 Second version of the protocol (submitted to PROSPERO on 31.03.2020, online on 29.10.2020)

Pharmacological and dietary supplement interventions for autism spectrum disorders (ASD): a systematic review, network meta-analysis, and meta-regression-analysis of placebo response
Spyridon Siafis, Oğulcan Çıray, Irene Bighelli, Johannes Schneider-Thoma, Stefan Leucht

Citation
Spyridon Siafis, Oğulcan Çıray, Irene Bighelli, Johannes Schneider-Thoma, Stefan Leucht. Pharmacological and dietary supplement interventions for autism spectrum disorders (ASD): a systematic review, network meta-analysis, and meta-regression-analysis of placebo response. PROSPERO CRD42019125317 Available from: https://www.crd.york.ac.uk/prospero/display_record.php?ID=CRD42019125317

Changes from previous version
1) Review questions are clearly presented. 2) A network meta-analysis will be conducted, if possible, to investigate the efficacy and tolerability of pharmacological and dietary supplement interventions. For this reason, head-to-head trials will also be eligible. 3) The outcome 'important side effects' is specified.

Review question
1) The first objective is to investigate predictors of placebo response and efficacy (drug-placebo differences) in placebo-controlled pharmacological and dietary supplement trials in ASD. Single-group and pairwise meta-analysis as well as meta-regression-analysis are planned.
2) The second objective is to investigate the comparative efficacy and tolerability of pharmacological and dietary supplement interventions for ASD. Pairwise and network meta-analysis are planned.

Searches
1) Electronic databases: Comprehensive searches will be conducted in ClinicalTrials.gov, Cochrane Central Register of Controlled Trials (CENTRAL), EMBASE, MEDLINE, PsycINFO, PubMed, World Health Organization International Clinical Trials Registry Platform (WHO ICTRP). There will be no date/time, language, document type, and publication status limitations.
2) Reference searching: Reference lists of included records will be hand-searched for potentially relevant studies.
3) Previous reviews: Relevant reviews on pharmacological and dietary supplement treatments for ASD will be hand-searched for potentially relevant studies.
4) Personal contact: In addition, we will contact the first and/or corresponding author of each included study published in the last 30 years for missing information.
At least two reviewers will independently inspect the titles and abstracts of non-duplicated references identified through the search and will exclude those not pertinent. Discrepancies between the two reviewers will be resolved by discussion reaching consensus. If doubts still remain, the full-text will be obtained. In a second step, full-texts will be independently assessed by two reviewers for eligibility. Again, disagreements will be resolved by discussion and, if needed,

a third senior author will be involved. When required, further information will be requested from study authors.

Search strategy
https://www.crd.york.ac.uk/PROSPEROFILES/125317_STRATEGY_20190213.pdf

Types of study to be included
Randomized controlled trials (RCT) in which participants with ASD received pharmacological treatments or dietary supplements compared to each other or placebo will be eligible.
Inclusion:
- Both open and blinded RCTs will be eligible.
- Randomization will be implied if not explicitly reported, when the study is stated as double-blind.
- In case of cross-over studies only data from the first phase before the crossover will be eligible, in order to avoid carry-over effects.
- No restriction in terms of language or country of origin.

Exclusion:
- Quasi-randomized trials and studies with high risk of bias in randomization as described in the Cochrane Handbook [2].
- Cluster randomized trials.
- Long-term studies with maintenance design, studies with placebo-controlled discontinuation or withdrawal design.
- Studies published before 1980 (see participants/population).
- Studies with less than 10 participants.

Condition or domain being studied
Autism spectrum disorders (ASD), including autistic disorder, Asperger's syndrome and pervasive developmental disorder-not otherwise specified, as they were previously classified as independent categorical entities in DSM-IV.

Participants/population
Inclusion:
- ASD as diagnosed by standardized diagnostic criteria (such as DSM-III, DSM-III-R, DSM-IV, DSM-IV-TR, DSM-5, ICD-10) and/or validated diagnostic tools.
- Studies including participants with ASD and associated conditions (e.g. ADHD symptoms, irritability) will be accepted.
- Studies with all or some of the participants having a genetic syndrome (such as Fragile X syndrome) will be accepted, when all participants had also ASD (defined by inclusion criteria).
- Trials in which less than 20% of participants had a developmental or psychiatric disorder other than ASD will be eligible.
- There will be no restriction in terms of age, sex, ethnicity, setting, initial severity of ASD symptoms.

Exclusion:
- Participants characterized as 'autistic' or with 'autistic behavior' and 'autistic traits' without using standardized diagnostic criteria or validated diagnostic tools for ASD. Studies published before 1980 will also be excluded, because ASD was clearly separated from childhood schizophrenia after the introduction of DSM-III (published in 1980). In addition, DSM-II and ICD-9 did not have standardized criteria.
- Studies requiring all participants to have a genetic syndrome, but not all of the participants had ASD defined by inclusion criteria.

- Studies focused on stable patients (controlled-discontinuation or withdrawal studies).

Intervention(s), exposure(s)
Any pharmacological treatment and dietary supplement will be eligible.
Inclusion:
- Any application form or route of administration (e.g. oral, intramuscular, intravenous, intranasal)
- Both fixed- and flexible-dose designs.
- The minimum duration of treatment will be seven days.

Exclusion:
- Other interventions, such as psychological/behavioral, traditional medicine, homeopathic, dietary interventions, such as elimination diets (gluten/casein-free, ketogenic diets) or milk formulations.
- Augmentation treatments.
- Studies using single doses.

Comparator(s)/control
Placebo or any eligible intervention can be a comparator in a network meta-analysis. Placebo will be used as reference for presentation.

Context

Main outcome(s)
The co-primary outcomes will be:
1) Overall ASD core symptoms.
2) Social communication/interaction deficits.
3) Repetitive behaviors/restricted interests.

* Measures of effect
Outcomes should be measured by published and validated scales. Scales could be filled by different raters, and in the primary analyses, we will prefer clinicians' to parents/caregivers' to teachers' ratings. Subgroup analysis of the co-primary outcomes will be conducted by pooling separately rating scales filled by parent/caregivers, teachers and clinicians. Outcomes will be measured at endpoint and studies will be classified in subgroup analyses as shorter-term (less or equal to three months) and medium-to-longer-term.
The effect sizes for continuous outcomes will be the standardized mean change (SMC, single-group) and the standardized mean difference as Hedges' g (SMD, treatment contrasts). SMCs will be preferably standardized to baseline standard deviations, and a pre-post correlation of 0.5 will be assumed for the primary analysis (correlations of 0.25 and 0.75 will be used in sensitivity analyses). The effect sizes for dichotomous outcomes will be response rates (single-group, logit transformed in the meta-analysis) and relative risks (treatment contrasts). Effect sizes will be accompanied by their 95% confidence intervals.

Additional outcome(s)
1) Internalizing associated symptoms (such as anxiety) as measured by appropriate scales.
2) Externalizing associated symptoms as well as ADHD symptoms and irritability, as measured by appropriate scales.
3) Number of participants with response to treatment, preferably defined by CGI-I at least much improved, but any study definition will be eligible for treatment comparisons.
4) Quality of life of participants as measured by appropriate scales (e.g. PedsQL).
5) Global functioning of participants as measured by appropriate scales (e.g. CGAS).

6) Parental stress as measured by appropriate scales (e.g. PSI).
7) Number of participants who prematurely discontinued due to any cause (as a measure of overall acceptability) and adverse events (as a measure of overall tolerability).
8) Important side effects defined as a) at least one adverse event, b) sedation, c) weight gain, d) extrapyramidal symptoms
* Measures of effect
As defined for the primary outcomes.

<u>Data extraction (selection and coding)</u>
Two authors will independently extract data from all selected trials in a Microsoft Access database. When disagreement arises we will resolve it by discussion and, if needed, involving a third senior author. Where this is not sufficient we will contact the study authors.

Data extraction will include characteristics of study (study citation, registration number to trials registries, year of publication, location, setting, number of centers, sample size, and funding/sponsor such as industry or academic), methodology (study design, number of arms and risk of bias), participants (age, sex, IQ, diagnosis, sample size), intervention (name, dose, application form) and outcome measure (including information on whether an intention-to-treat approach has been used and how it was defined)

For continuous outcomes, change scores will be preferred, but endpoint scores will also be eligible. Missing standard deviations (SD) will be calculated from the following options and following order by the 1) standard error, 2) CIs, t-value or p-value, 3) contacting original authors, 4) median-ranges, 5) pooling subscales with an assumed correlation of 0.5, 6) by SDs from other studies using a validated imputation method as described in the Cochrane Handbook. Intention-to-treat data will be used whenever possible and when imputation methods were used to handle missing data, they will be preferred to completers' data, giving preference to mixed-models of repeated measurement (MMRM) and multiple imputation over last-observation carried forward (LOCF). For dichotomous outcomes, if the original authors presented only the results of completer population, we will assume that those participants lost to follow-up would not have changed for a given outcome. When the number of responders was not reported, it will be imputed from mean and standard deviation (SD) of CGI-I using a validated method and studies with imputed responders will be excluded in a sensitivity analysis.

<u>Risk of bias (quality) assessment</u>
Two independent review authors will assess the risk of bias in the selected studies using the Cochrane Collaboration 'risk of bias' tool. When disagreement arises we will resolve it by discussion and, if needed, involving a third senior author. The following domains will be considered (classified as low, moderate or high): sequence generation, allocation concealment, blinding, completeness of outcome data, selective reporting and other biases. Similar to the GRISELDA NMA [3, 4], studies will be classified as having an overall low (no domain with high risk of bias and three or less with unclear risk), moderate (one with high risk of bias or none with high risk of bias but four or more with unclear risk) and high risk of bias (all other cases). Confidence in the results of the co-primary outcomes will be assessed using the approach of CINeMA [5] [6].

<u>Strategy for data synthesis</u>
1) For the first objective, single-group (placebo/drug response) and pairwise meta-analysis (drug-placebo differences) will be conducting for the co-primary outcomes by pooling placebo-controlled trials using random-effects models.
Heterogeneity will be investigated by visual inspection of forest plots, chi-squared and I-squared statistics.

2) For the second objective, as a first step of a network-meta-analysis (NMA), pairwise meta-analyses will be conducted using random-effects models by pooling each treatment comparison, separately in children/adolescents and adults.
We assume that participants eligible to our review have an equal likelihood to be randomized to any of the intervention of the NMA (transitivity assumption). The distribution of potential effect-modifiers will also be investigated, i.e. study duration, type of rater, associated conditions at inclusion, baseline severity.

In a second step and if the requirements of NMA are fulfilled, we will conduct NMA in a frequentist framework using a random-effects model and assuming a common variance (tau-squared) for each network. We will present relative effects and 95% confidence intervals for all pairwise comparisons.

Heterogeneity will be quantified with the tau-squared and we will compare it to empirical distributions. If the estimation of treatment effects is precise, we aim to obtain an hierarchy of treatments [7].

Incoherence will be evaluated both locally (with the 'separating indirect from direct evidence' approach) and globally (with a design-by-treatment interaction test) [8]. Tests for inconsistency are expected to have small statistical power, and therefore, sources of inconsistency will be explored even when evidence of inconsistency is lacking.

3) We will attempt to include unpublished studies. Small study and publication bias will be explored for both objectives with funnel plot analyses if at least 10 studies are available per comparison.

4) Statistical software: R (meta [9], netmeta [10], metafor [11] packages)

Analysis of subgroups or subsets

1) Meta-regressions of predictors of placebo/drug response or efficacy will be conducted similarly to our previous analyses in acute schizophrenia [12]. A priori, we plan exploratory univariable meta-regressions; multivariable meta-regressions will also be conducted, if there are enough data. Dependent variables will be SMC of placebo/drug response or SMD of efficacy in core symptoms. Independent variables will be the following potential predictors:

- Participants: a) associated symptoms, b) age c) sex (post-hoc), d) ethnicity (post-hoc), e) intellectual disability, f) baseline severity.
- Intervention: a) route of administration (oral versus other), b) type of intervention (pharmacological versus dietary supplements), c) fixed- versus flexible-designs.
- Study-design: a) study-duration (weeks), b) publication year, c) wash-out, c) lead-in with exclusion of placebo responders, d) rater (clinician versus caregiver), e) sample size, f) number of sites and academic sites, g) number of arms and medications, h) proportion of participants on placebo, i) country of origin (US vs not only US), j) sponsorship (industry-funded/patent application vs industry-independent), k) risk-of-bias domains

2) Sensitivity analysis of the co-primary outcomes will be conducted by excluding studies with a) implied randomization, b) genetic syndrome at inclusion, c) diagnosis using only diagnostic evaluation tools, d) open- or single-blind, e) duration of less than 4 weeks, f) presenting only completers data, g) imputed SDs, h) overall high or moderate risk-of-bias, i) by using fixed-effects model.

3) In NMA, subgroup analyses of the co-primary outcomes will investigate potential effect-modifiers, i.e. a) study-duration, b) rater, c) associated condition and d) baseline severity

Contact details for further information
Stefan Leucht
stefan.leucht@tum.de

Organizational affiliation of the review
Department of Psychiatry and Psychotherapy, School of Medicine, Technical University of Munich, Klinikum rechts der Isar http://www.psykl.mri.tum.de/

Review team members and their organisational affiliations

- Mr Spyridon Siafis. Department of Psychiatry and Psychotherapy, School of Medicine, Technische Universität München, Klinikum rechts der Isar
- Dr. Oğulcan Çıray, Dokuz Eylul University, School of Medicine, Department of Child and Adolescent Psychiatry Balçova, İzmir, Turkey
- Dr Irene Bighelli. Department of Psychiatry and Psychotherapy, School of Medicine, Technische Universität München, Klinikum rechts der Isar
- Dr Johannes Schneider-Thoma. Department of Psychiatry and Psychotherapy, School of Medicine, Technische Universität München, Klinikum rechts der Isar
- Professor Stefan Leucht. Department of Psychiatry and Psychotherapy, School of Medicine, Technische Universität München, Klinikum rechts der Isar

Collaborators

- Professor Celso Arango. Department of Child and Adolescent Psychiatry, Hospital General Universitario Gregorio Marañón, Madrid, Spain; IiSGM, CIBERSAM, Spain; School of Medicine, Universidad Complutense, Madrid, Spain.
- Professor Mara Parellada. Department of Child and Adolescent Psychiatry, Hospital General Universitario Gregorio Marañón, Madrid, Spain; IiSGM, CIBERSAM, Spain; School of Medicine, Universidad Complutense, Madrid, Spain
- Professor Dimitris Mavridis, Department of Primary Education, University of Ioannina, Greece
- Mr Farhad Shokraneh. Cochrane Schizophrenia Group, The Institute of Mental Health, the University of Nottingham and Nottinghamshire Healthcare NHS Trust, Nottingham, UK

Type and method of review
Intervention, Meta-analysis, Systematic review

Anticipated or actual start date
01 June 2018

Anticipated completion date
31 May 2020

Funding sources/sponsors
This project has received funding from the Innovative Medicines Initiative 2 Joint Undertaking under grant agreement No 777394. This Joint Undertaking receives support from the European

Union's Horizon 2020 research and innovation programme and EFPIA and AUTISM SPEAKS, Autistica, SFARI

Conflicts of interest

- In the last 3 years, Stefan Leucht has received honoraria for consulting or lectures from LB Pharma, Lundbeck, Otsuka, TEVA, LTS Lohmann, Geodon Richter, Recordati, Boehringer Ingelheim, Sandoz, Janssen, Lilly, SanofiAventis, Servier and Sunovion.
- David Fraguas has been a consultant and/or has received fees from Angelini, Eisai, IE4Lab, Janssen, Lundbeck, and Otsuka. He has also received grant support from Instituto de Salud Carlos III (Spanish Ministry of Science, Innovation and Universities) and from Fundación Alicia Koplowitz.
- Mara Parellada has received educational honoraria from Otsuka, research grants from FAK and Fundación Mutua Madrileña (FMM), Instituto de Salud Carlos III (Spanish Ministry of Science, Innovation and Universities) and European ERANET and H2020 calls, travel grants from Otsuka and Janssen. Consultant for Exeltis and Servier.

Language
English

Country
Germany
Stage of review
Review Ongoing

Subject index terms status
Subject indexing assigned by CRD

Subject index terms
Autism Spectrum Disorder; Dietary Supplements; Humans

Date of registration in PROSPERO
15 March 2019

Date of publication of this version
15 March 2019

Stage of review at time of this submission
Preliminary searches: Started Yes Completed Yes
Piloting of the study selection process: Started Yes Completed Yes
Formal screening of search results against eligibility criteria: Started Yes Completed No
Data extraction: Started Yes Completed No
Risk of bias (quality) assessment: Started Yes Completed No
Data analysis: Started Yes Completed No

2.1.2 First version of the protocol (15.03.2019)

Placebo-controlled pharmacological and dietary supplement trials in autism spectrum disorders (ASD): systematic review, meta-analysis and meta-regression
Spyridon Siafis, Irene Bighelli, Johannes Schneider-Thoma, Stefan Leucht

Citation
Spyridon Siafis, Irene Bighelli, Johannes Schneider-Thoma, Stefan Leucht. Placebo-controlled pharmacological and dietary supplement trials in autism spectrum disorders (ASD): systematic review, meta-analysis and meta-regression. PROSPERO 2019 CRD42019125317 Available from: https://www.crd.york.ac.uk/prospero/display_record.php?ID=CRD42019125317

Review question
To investigate predictors of efficacy and placebo response in placebo-controlled pharmacological and dietary supplement trials in ASD.

Searches
- Electronic databases: Comprehensive searches will be conducted in ClinicalTrials.gov, Cochrane Central Register of Controlled Trials (CENTRAL), EMBASE, MEDLINE, PsycINFO, PubMed, World Health Organization International Clinical Trials Registry Platform (WHO ICTRP). There will be no date/time, language, document type, and publication status limitations.
- Reference searching: Reference lists of included records will be hand-searched for potentially relevant studies.
- Previous reviews: Relevant reviews on pharmacological and dietary supplement treatments for ASD will be hand-searched for potentially relevant studies.
- Personal contact: In addition, we will contact the first and/or corresponding author of each included study published in the last 30 years for missing information.

Search strategy
https://www.crd.york.ac.uk/PROSPEROFILES/125317_STRATEGY_20190213.pdf

Types of study to be included
Randomized controlled trials (RCT) in which participants with ASD received pharmacological treatments or dietary supplements compared to placebo will be eligible.
Inclusion:
- Both open and blinded RCTs.
- Randomization will be implied if not explicitly reported, when the study is stated as double-blind.
- In case of cross-over studies only data from the first phase before the crossover will be eligible, in order to avoid carry-over effects.
- No restriction in terms of language or country of origin.

Exclusion:
- Quasi-randomized trials and studies with high risk of bias in randomization as described in the Cochrane Handbook [13].
- Cluster randomized trials.
- Long-term studies with maintenance design, studies with placebo-controlled discontinuation or withdrawal design.
- Studies published before 1980 (see participants/population).
- Studies with less than 10 participants.

Condition or domain being studied

Autism spectrum disorders (ASD), including autistic disorder, Asperger's syndrome and pervasive developmental disorder-not otherwise specified, as they were previously classified as independent categorical entities in DSM-IV.

Participants/population

Inclusion:

- ASD as diagnosed by standardized diagnostic criteria (such as DSM-III, DSM-III-R, DSM-IV, DSM-IV-TR,DSM-5, ICD-10) and/or validated diagnostic tools.
- Studies including participants with ASD and associated symptoms (e.g. ADHD symptoms, irritability) will be accepted.
- Studies with all or some of the participants having a genetic syndrome (such as Fragile X syndrome) will be accepted, when all participants had also ASD (defined by inclusion criteria).
- Trials in which less than 20% of participants had a developmental or psychiatric disorder other than ASD will be eligible.
- There will be no restriction in terms of age, sex, ethnicity, setting, initial severity of ASD symptoms.

Exclusion:

- Participants characterized as 'autistic' or with 'autistic behavior' and 'autistic traits' without using standardized diagnostic criteria or validated diagnostic tools for ASD. Studies published before 1980 will also be excluded, because ASD was clearly separated from childhood schizophrenia after the introduction of DSM-III (published in 1980). In addition, DSM-II and ICD-9 did not have standardized criteria.
- Studies requiring all participants to have a genetic syndrome, but not all of the participants had ASD defined by inclusion criteria.
- Studies focused on stable patients (controlled-discontinuation or withdrawal studies).

Intervention(s), exposure(s)

Any pharmacological treatment and dietary supplement will be eligible.

Inclusion:

- Any application form or route of administration (e.g. oral, intramuscular, intravenous, intranasal) - Both fixed- and flexible-dose designs.
- The minimum duration of treatment will be seven days.

Exclusion:

- Other interventions, such as psychological/behavioral, traditional medicine, homeopathic, dietary interventions, such as elimination diets (gluten/casein-free, ketogenic diets) or milk formulations.
- Augmentation treatments.
- Studies using single doses.

Comparator(s)/control

Placebo.

Context

Main outcome(s)

- Overall ASD core symptoms.
- Social communication/interaction deficits.
- Repetitive behaviors/restricted interests.

Timing and effect measures

Published and validated scales will be used. When scales filled by multiple informants are available, we will use the hierarchy: clinicians', parents/caregivers' and teachers' rating. Regarding core symptoms, separate analyses will be conducted for rating scales filled by parent/caregivers, teachers and clinicians as secondary outcomes. Change scores will be preferred, but we will also use endpoint scores if the former are not available.
We will take the endpoint results and pool all studies. In addition, studies will be classified as shorter-term (1-12 weeks) and longer-term (13 or more weeks).

<u>Additional outcome(s)</u>

- CGI-improvement and CGI-Severity.
- Overall ASD core symptoms as measured by rating scales filled by clinicians, caregivers or teachers.
- Social communication/interaction deficits as measured by rating scales filled by clinicians, caregivers or teachers.
- Repetitive behaviors/restricted interests as measured by rating scales filled by clinicians, caregivers or teachers.
- Internalizing associated symptoms (such as anxiety) as measured by appropriate scales.
- Externalizing associated symptoms as well as ADHD symptoms and irritability, as measured by appropriate scales.
- Number of participants with response to treatment, any study definition is eligible.
- Quality of life of participants as measured by appropriate scales (e.g. PedsQL)
- Global functioning of participants as measured by appropriate scales (e.g. CGAS)
- Parental stress as measured by appropriate scales (e.g. PSI)
- Number of participants who prematurely discontinued due to any cause (as a measure of overall acceptability), inefficacy (as a measure of global efficacy), adverse events (as a measure of overall tolerability).
- Important side effects.

Timing and effect measures

We will take the endpoint results and pool all studies. In addition, studies will be classified as shorter-term (1-12 weeks) and longer-term (13 or more weeks).

<u>Data extraction (selection and coding)</u>

Selection of trials: At least two reviewers will independently inspect the titles and abstracts of non-duplicated references identified through the search and will exclude those not pertinent. Discrepancies between the two reviewers will be resolved by discussion reaching consensus. If doubts still remain, the full-text will be obtained and eligibility will be assessed. Full-texts of included references will be obtained and independently assessed by two reviewers for eligibility. Again, disagreements will be resolved by discussion and, if needed, a third senior author will be involved. When required, further information will be requested from study authors.

Data extraction: Two authors will independently extract data from all selected trials in a Microsoft Access database. When disagreement arises we will resolve it by discussion and, if needed, involving a third senior author. Where this is not sufficient we will contact the study authors.
Data extraction will include:

- Study citation, registration number to trials registries, year of publication, location, setting, number of centers, sample size, and funding/sponsor (industry or academic).
- Methodology (study design, number of arms and risk of bias).
- Characteristics of study participants (age, sex, IQ, diagnosis, sample size).
- Characteristics of intervention (name, dose, application form).
- Outcome measures, including information on whether an intention-to-treat approach has been used and how it was defined.

Risk of bias (quality) assessment

Two independent review authors will assess the risk of bias in the selected studies using the Cochrane Collaboration 'risk of bias' tool. When disagreement arises we will resolve it by discussion and, if needed, involving a third senior author. The following domains will be considered (classified as low, moderate or high): sequence generation, allocation concealment, blinding, completeness of outcome data, selective reporting and other biases. Similar to the GRISELDA NMA [3, 4] studies will be classified as having an overall low (no domain with high risk of bias and three or less with unclear risk), moderate (one with high risk of bias or none with high risk of bias but four or more with unclear risk) and high risk of bias (all other cases). Quality of evidence for the primary outcome will be assessed by GRADE approach [14].

Strategy for data synthesis

The effect size for continuous outcomes will be the standardized mean difference as Hedges' g, and for dichotomous outcomes the relative risk, accompanied by their 95% confidence intervals. Intention-to-treat data will be used whenever possible. For dichotomous outcomes, if the original authors presented only the results of completer population, we will assume that those participants lost to follow-up would not have changed for a given outcome. Missing standard deviations (SD) will be calculated from the following options and following order by the 1) standard error, 2) CIs, t-value or p-value, 3) contacting original authors 4) by SDs from other studies using a validated imputation method as described in the Cochrane Handbook.

Pairwise meta-analyses of studies, separately for children/adolescents and adults, that compared the same intervention with placebo will be conducted using random effects models [15]. Meta-regressions of predictors of efficacy and placebo response will be conducted by using all studies. Meta-regressions for individual interventions will not be conducted due to small statistical power. Heterogeneity will be investigated by visual inspection of the forest plots, χ^2 test of homogeneity and I^2.

We will attempt to include unpublished studies. Small study and publication bias will be explored with funnel plot analyses if at least 10 studies are available for a comparison.

Sensitivity analyses of the primary outcomes will be performed: A) Exclusion of studies with implied randomization. B) Exclusion of studies including participants with associated symptoms or genetic syndrome. C) Exclusion of studies with a diagnosis based only on diagnostic evaluation tools. D) Exclusion of open and single-blinded trials. E) Exclusion of studies lasting less than 4 weeks. F) Using a fixed effects model. G) Exclusion of studies presenting only completers data. H) Exclusion of studies with imputed missing SD. I) Exclusion of studies with an overall high or unclear risk of bias.

Analysis of subgroups or subsets

Meta-regressions of predictors of efficacy and placebo response will be conducted for the primary outcomes using a similar approach to our previous meta-regressions in acute schizophrenia [16-18]. The dependent variables in the analyses will be 1) placebo response, 2) drug response and 3) effect sizes for the comparisons of interventions with placebo. The independent variables will be the following potential moderators. A priori, we plan exploratory univariate meta-regressions. Multivariable meta-regression models will be conducted, if there are enough available data, because a higher statistical power is required.

Potential moderators will be assessed:
1. Drug-related factors: A) Route of administration (oral versus other). B) Type of intervention: pharmacological versus dietary supplements. C) Fixed versus flexible designs.
2. Design-related factors: A.) Study duration (in weeks). B) Target symptom of the study: associated symptoms versus not. C) Publication year. D) Duration of wash-out (in days). E) Use of placebo-lead in phase with exclusion of placebo responders. F) Type of informant: parent/caregiver versus clinician or teacher. G) Sample size. H) Number of sites and proportion of academic sites. I) Number of arms and medications. J) Percentage of participants on placebo. K) Sponsorship (at least one site industry funded, no donation alone) versus not industry funded. L) Risk of bias for each domain.
3. Participant-related factors: A. Degree of placebo response (when efficacy or drug response are the dependent variables). B. Mean age. C. US population versus not US or mixed populations. D. Baseline severity. E. Intellectual impairment

Contact details for further information

Stefan Leucht
stefan.leucht@tum.de

Organisational affiliation of the Review

Department of Psychiatry and Psychotherapy, Technische Universität München, Klinikum rechts der Isar http://www.cfdm.de/

Review team members and their organisational affiliations

- Mr Spyridon Siafis. Department of Psychiatry and Psychotherapy, Technische Universität München, Klinikum rechts der Isar
- Dr Irene Bighelli. Department of Psychiatry and Psychotherapy, Technische Universität München, Klinikum rechts der Isar
- Dr Johannes Schneider-Thoma. Department of Psychiatry and Psychotherapy, Technische Universität München, Klinikum rechts der Isar
- Professor Stefan Leucht. Department of Psychiatry and Psychotherapy, Technische Universität München, Klinikum rechts der Isar

Collaborators

- Professor Celso Arango. Department of Child and Adolescent Psychiatry, Hospital General Universitario Gregorio Marañón, Madrid, Spain; IiSGM, CIBERSAM, Spain; School of Medicine, Universidad Complutense, Madrid, Spain.
- Professor Mara Parellada. Department of Child and Adolescent Psychiatry, Hospital General Universitario
- Gregorio Marañón, Madrid, Spain; IiSGM, CIBERSAM, Spain; School of Medicine, Universidad

- Complutense, Madrid, Spain
- Mr Farhad Shokraneh. Cochrane Schizophrenia Group, The Institute of Mental Health, the University of Nottingham and Nottinghamshire Healthcare NHS Trust, Nottingham, UK

Type and method of review
Intervention, Meta-analysis, Systematic review

Anticipated or actual start date
01 June 2018

Anticipated completion date
31 May 2020

Funding sources/sponsors
This project has received funding from the Innovative Medicines Initiative 2 Joint Undertaking under grant agreement No 777394. This Joint Undertaking receives support from the European Union's Horizon 2020 research and innovation programme and EFPIA and AUTISM SPEAKS, Autistica, SFARI

Conflicts of interest

- In the last 3 years, Stefan Leucht has received honoraria for consulting or lectures from LB Pharma, Lundbeck, Otsuka, TEVA, LTS Lohmann, Geodon Richter, Recordati, Boehringer Ingelheim, Sandoz, Janssen, Lilly, SanofiAventis, Servier and Sunovion.
- David Fraguas has been a consultant and/or has received fees from Angelini, Eisai, IE4Lab, Janssen, Lundbeck, and Otsuka. He has also received grant support from Instituto de Salud Carlos III (Spanish Ministry of Science, Innovation and Universities) and from Fundación Alicia Koplowitz.
- Mara Parellada has received educational honoraria from Otsuka, research grants from FAK and Fundación Mutua Madrileña (FMM), Instituto de Salud Carlos III (Spanish Ministry of Science, Innovation and Universities) and European ERANET and H2020 calls, travel grants from Otsuka and Janssen. Consultant for Exeltis and Servier.

Language
English

Country
Germany

Stage of review
Review Ongoing

Subject index terms status
Subject indexing assigned by CRD

Subject index terms
Autism Spectrum Disorder; Dietary Supplements; Humans

Date of registration in PROSPERO
15 March 2019

Date of publication of this version
15 March 2019

2.2 Methods clarifications and post-hoc decisions

- Exclusion of combination, augmentation or multimodal treatments: We excluded interventions aiming to idenrtify treatment effects of augmentation, combination or multimodal treatments, e.g., risperidone + memantine, atomoxetine + parental training, vitamin-D + omega-3, vitamin-D + perceptual-motor exercises.
- Exclusion of low fixed-doses: In case of fixed-doses, we excluded a low dose arm of risperidone (≤0.175mg/day; smaller than 0.25mg/day initial recommended dose by FDA) [19] and of balovaptan (1.5mg/day; a dose with limited pharmacodynamic effects that was used to provide initial safety) [20]. Multiple dose arms of the same intervention were combined according to the Cochrane Handbook [2].
- Exclusion of studies with high risk of bias in the randomization process: According to our protocol, we included studies with a low or unclear risk of bias in the randomization process, and we excluded studies with a high risk of bias in randomization, in accordance to the Cochrane Handbook [13, 21]. Sequence generation and allocation concealment are both components of the randomization process and the associated selection bias [22]. Therefore, we excluded studies with a high risk of bias in sequence generation (e.g., allocation by the day of the week, clinical judgment, patient preferences, laboratory values, or any other method without a random component of allocation etc.) or allocation concealment (e.g. allocation by alternation, non-opaque envelopes, open table of random tables, allocation by an unblinded investigator) according to the risk of bias tool [21]. Studies with high risk of bias in one of these two domains could inflate effect-sizes in favor of the interventions, e.g. [23].

  We a priori included studies with unclear risk of bias in random sequence generation or allocation concealment. This decision is according to the standards of the Cochrane Handbook [21], and supported by evidence from meta-epidemiological studies, e.g., [24-27]. These meta-epidemiological studies suggested that poor reporting does not necessarily reflect the actual methods conducted in the trials. One large meta-epidemiological study of 429 RCTs (and their protocols) found that from the trials with adequate random sequence generation and acllocation concealment, only 59% and 25% of them reported the exact methods in their publications [27]. Similar results were found in an older meta-epidemiological study of 56 RCTs in radiation oncology, which found that despite all trials had adequate allocation concealment, only 42% of them reported the exact method in their papers [26]. Another meta-epidemiological study of 98 RCTs (published in 29 medical journals before 2002) found that about 96% of RCTs with unclear reporting of allocation concealment had indeed concealed allocation [24]. In a similar vein, another meta-epidemiological study of 40 RCTs (also published before 2002) in rheumatology, which found that 76% and 78% of the studies with unclear reporting in random sequence generation and allocation concealment had indeed adequate methods [25]. This meta-epidemiological study included also non-drug trials (32.5% of the sample) that accounted for most of the trials with inadequately performed randomization methods [25].

  Most the included trials in our meta-analysis were very recently published (median publication year of 2015, interquartile range [2008-2019]) and only a few studies were published before the CONSORT statement (1996). A meta-epidemiological study found that the methodology of RCTs in psychopharmacology has improved in the last sixty years, yet there may be a disconnection between improvement in methodology and adequate reporting of the methodology [28]. Nevertheless, we acknowledge that there could be a few studies with unclear risk of bias in random sequence generation or allocation concealment, which could have had inadequate randomization methods. Therefore, we excluded these studies in a post-

hoc sensitivity analysis, and the results did not materially change (see eAppendix-6.6., Figure-S4).

- Including data from unpublished studies: We aimed to include studies with unpublished data, e.g., by contacting authors or using data from trial registries, conference abstracts, presentations or reviews. When more than one sources of data were available, preference was given to data and clarifications provided by authors, clinical study reports, published manuscripts, trial registries or conference abstracts, presentations and other reviews.
- Extraction of descriptive characteristics: A) About descriptive characteristics (e.g., age, sex), we preferred as randomized data per arm, but we also used completers' data per arm, or data from the total sample. B) Baseline severity was inconsistently assessed and reported in trials. CGI-S and ABC-Irritability were used as measures of global severity and serious problem behaviors, as well as they were used in other meta-analyses as measures of baseline severity [29-31]. C) Transitivity assumption was investigated with the distribution of potential effect-modifiers across interventions. In the predefined list of potential effect-modifiers, mean age was added post-hoc. D) We classified studies as industry sponsored or linked to a patent application, when 1. the study was funded by the industry (irrespective if the analysis was conducted centrally by the sponsor or independently), 2. at least an author of the manuscript was an industry employee, 3. at least an author of the manuscript had applied for a patent on the use of the intervention (e.g., as reported in the conflicts of interests). A study that was funded by the state or academia was not considered as industry-sponsored. We did not consider conflicts of interest in the forms of consultation of speaker fees or if the industry provided or donated the medications for the study.
- Imputation of responder rates: We analysed responder rates as a secondary outcome, since they are easily interpretable in comparison to standardized mean changes. We used response as defined by at least much improvement in the CGI-I (CGI-I=1 or 2), which is a rather homogenous definition (despite the use of different anchors, as well as CGI-I was frequently used in clinical trials of autism [32, 33]. We imputed the number of responders from mean and standard deviations of CGI-I, when they were not reported, using and validated method [34, 35]. In the imputation, a threshold of 2.5 was used instead of 2 to impute responders from an assumed normal distribution of the continuous CGI-I. This artificial threshold wa used, since CGI-I is a categorical scale with 7-points, so that we assumed that a participant with a value of 2.5 or less in the assumed underlying normal distribution would have been considered as at least much improved [36].
- Extraction of mean and standard deviations from median/ranges and from pooling subscales: When only medians and ranges were reported, we estimated means and standard deviations as reported in Shi et al 2020 [37] . When subscale scores were reported instead of eligible total scores, we pooled means and standard deviations of the subscales assuming a correlation of 0.5 [38]. In a predefined sensitivity analysis, we excluded studies with imputed standard deviations [13, 39], including studies with estimated means and standard deviations from medians/ranges or studies in which subscales scores were pooled.
- Extraction of change scores: In our protocol, we preferred change scores to endpoint scores, yet endpoint scores were used when the former were not reported. Since we found large baseline imbalances in some studies, which could have inflated effect sizes when endpoint scores were used, we estimated change scores when both endpoint and baseline scores were provided using a correlation of 0.5 [40] (see eAppendix-6.1.2 for further explanation). Therefore, we used the following hierarchy: 1) reported change scores, 2) estimated change scores when endpoint and baseline scores were reported, 3) endpoint scores (when baseline scores were not reported). In a sensitivity analysis, we used a correlation of 0.25 and 0.75.

- Using odds ratios instead of relative risks: Odds ratios (OR) were post-hoc used instead of relative risks (RR), since there is more recent evidence that ORs have better mathematical properties and should be preferred in meta-analysis [41, 42]. Nevertheless, we conducted a sensitivity analysis using relative risks, and the results did not materially change (Figure-S4). In addition, Figures 2-4 present the effect sizes of each medication in comparison to placebo for the continuous (measured with SMDs) and dichotomous outcomes (measured with ORs). In order to increase interpretability, we converted ORs to SMDs using the formula in the Cochrane Handbook (SMD=ln(OR)/1.81)[2].
- Changes in sensitivity analysis and subgroup analysis: A) Due to the disproportionate number of studies to interventions, we investigated a priori defined subgroup analyses as sensitivity analyses, i.e., 1. excluding studies with associated symptoms as inclusion criteria, 2. excluding studies shorter than four weeks, 3. excluding studies with non-clinician ratings. Baseline severity could not be assessed due to inconsistent reporting and large diversity of scales. B) In a sensitivity analysis, we post-hoc excluded studies with an overall high risk of bias, instead of the predefined sensitivity analysis of excluding studies with a high or moderate risk of bias. Since an important number of studies were rated with a moderate risk of bias, their exclusion would have led to a not meaningful sensitivity analysis. Nevertheless, we discussed the presence of risk of bias and risk of bias was incorporated in CINeMA approach for the primary outcomes. C) In a post-hoc sensitivity analysis, we excluded studies from less developed countries or countries with less tradition in clinical research (i.e. Azerbaijan, China, Egypt, India, Indonesia, Iran, Turkey, Ukrania), since there is evidence that treatment effects are on average more favourable in less developed countries [43]. D) In a post-hoc sensitivity analysis suggested by a reviewer, we used ABC-L/SW for social-communication difficulties and ABC-S for repetitive behaviors. E) In a post-hoc sensitivity analysis, we excluded studies with an unclear description of random sequence generation or allocation concealment.

2.3 References

1. Siafis S, Çıray O, Schneider-Thoma J, Bighelli I, Krause M, Rodolico A, et al. Placebo response in pharmacological and dietary supplement trials of autism spectrum disorder (ASD): systematic review and meta-regression analysis. Mol Autism. 2020;11(1):66. doi: 10.1186/s13229-020-00372-z.
2. Higgins JP, Thomas J, Chandler J, Cumpston M, Page MJ, Welch VA. Cochrane Handbook for Systematic Reviews of Interventions version 6.0. 2019.
3. Furukawa TA, Salanti G, Atkinson LZ, Leucht S, Ruhe HG, Turner EH, et al. Comparative efficacy and acceptability of first-generation and second-generation antidepressants in the acute treatment of major depression: protocol for a network meta-analysis. BMJ Open. 2016;6(7):e010919. doi: 10.1136/bmjopen-2015-010919.
4. Cipriani A, Furukawa TA, Salanti G, Chaimani A, Atkinson LZ, Ogawa Y, et al. Comparative efficacy and acceptability of 21 antidepressant drugs for the acute treatment of adults with major depressive disorder: a systematic review and network meta-analysis. Lancet. 2018;391(10128):1357-66. doi: 10.1016/s0140-6736(17)32802-7.
5. Papakonstantinou T, Nikolakopoulou A, Higgins JPT, Egger M, Salanti G. CINeMA: Software for semiautomated assessment of the confidence in the results of network meta-analysis. Campbell Systematic Reviews. 2020;16(1):e1080. doi: 10.1002/cl2.1080.
6. Nikolakopoulou A, Higgins JPT, Papakonstantinou T, Chaimani A, Del Giovane C, Egger M, et al. CINeMA: An approach for assessing confidence in the results of a network meta-analysis. PLOS Medicine. 2020;17(4):e1003082. doi: 10.1371/journal.pmed.1003082.
7. Rücker G, Schwarzer G. Ranking treatments in frequentist network meta-analysis works without resampling methods. BMC Medical Research Methodology. 2015;15(1):58. doi: 10.1186/s12874-015-0060-8.
8. Efthimiou O, Debray TPA, van Valkenhoef G, Trelle S, Panayidou K, Moons KGM, et al. GetReal in network meta-analysis: a review of the methodology. Research Synthesis Methods. 2016;7(3):236-63. doi: 10.1002/jrsm.1195.
9. Schwarzer G. meta: An R package for meta-analysis. R news. 2007;7(3):40-5.
10. Rücker G, Schwarzer G, Krahn U, König J, Schwarzer MG. Package ‘netmeta’. Network Meta-Analysis using Frequentist Methods (Version 07-0). 2015.
11. Viechtbauer W. Conducting meta-analyses in R with the metafor package. Journal of statistical software. 2010;36(3):1-48.
12. Leucht S, Chaimani A, Mavridis D, Leucht C, Huhn M, Helfer B, et al. Disconnection of drug-response and placebo-response in acute-phase antipsychotic drug trials on schizophrenia? Meta-regression analysis. Neuropsychopharmacology. 2019;44(11):1955-66. doi: 10.1038/s41386-019-0440-6.
13. Higgins JP, Green S. Cochrane handbook for systematic reviews of interventions. John Wiley & Sons; 2011.
14. Guyatt G, Oxman AD, Akl EA, Kunz R, Vist G, Brozek J, et al. GRADE guidelines: 1. Introduction-GRADE evidence profiles and summary of findings tables. J Clin Epidemiol. 2011;64(4):383-94. doi: 10.1016/j.jclinepi.2010.04.026.
15. DerSimonian R, Laird N. Meta-analysis in clinical trials. Controlled clinical trials. 1986;7(3):177-88.
16. Leucht S, Chaimani A, Leucht C, Huhn M, Mavridis D, Helfer B, et al. 60years of placebo-controlled antipsychotic drug trials in acute schizophrenia: Meta-regression of predictors of placebo response. Schizophr Res. 2018;201:315-23. doi: 10.1016/j.schres.2018.05.009.
17. Leucht S, Chaimani A, Mavridis D, Leucht C, Huhn M, Helfer B, et al. Disconnection of drug-response and placebo-response in acute-phase antipsychotic drug trials on schizophrenia? Meta-regression analysis. Neuropsychopharmacology. 2019. doi: 10.1038/s41386-019-0440-6.
18. Leucht S, Leucht C, Huhn M, Chaimani A, Mavridis D, Helfer B, et al. Sixty Years of Placebo-Controlled Antipsychotic Drug Trials in Acute Schizophrenia: Systematic Review, Bayesian Meta-

Analysis, and Meta-Regression of Efficacy Predictors. Am J Psychiatry. 2017;174(10):927-42. doi: 10.1176/appi.ajp.2017.16121358.
19. Kent JM, Kushner S, Ning X, Karcher K, Ness S, Aman M, et al. Risperidone dosing in children and adolescents with autistic disorder: a double-blind, placebo-controlled study. Journal of autism and developmental disorders. 2013;43(8):1773-83.
20. Bolognani F, del Valle Rubido M, Squassante L, Wandel C, Derks M, Murtagh L, et al. A phase 2 clinical trial of a vasopressin V1a receptor antagonist shows improved adaptive behaviors in men with autism spectrum disorder. Science translational medicine. 2019;11(491).
21. Higgins JP, Altman DG, Gotzsche PC, Juni P, Moher D, Oxman AD, et al. The Cochrane Collaboration's tool for assessing risk of bias in randomised trials. Bmj. 2011;343:d5928. doi: 10.1136/bmj.d5928.
22. Odgaard-Jensen J, Vist GE, Timmer A, Kunz R, Akl EA, Schunemann H, et al. Randomisation to protect against selection bias in healthcare trials. Cochrane Database Syst Rev. 2011(4):Mr000012. doi: 10.1002/14651858.MR000012.pub3.
23. Pildal J, Hróbjartsson A, Jørgensen KJ, Hilden J, Altman DG, Gøtzsche PC. Impact of allocation concealment on conclusions drawn from meta-analyses of randomized trials. Int J Epidemiol. 2007;36(4):847-57. doi: 10.1093/ije/dym087.
24. Devereaux PJ, Choi PT, El-Dika S, Bhandari M, Montori VM, Schünemann HJ, et al. An observational study found that authors of randomized controlled trials frequently use concealment of randomization and blinding, despite the failure to report these methods. J Clin Epidemiol. 2004;57(12):1232-6. doi: 10.1016/j.jclinepi.2004.03.017.
25. Hill CL, LaValley MP, Felson DT. Discrepancy between published report and actual conduct of randomized clinical trials. J Clin Epidemiol. 2002;55(8):783-6. doi: 10.1016/s0895-4356(02)00440-7.
26. Soares HP, Daniels S, Kumar A, Clarke M, Scott C, Swann S, et al. Bad reporting does not mean bad methods for randomised trials: observational study of randomised controlled trials performed by the Radiation Therapy Oncology Group. Bmj. 2004;328(7430):22-4. doi: 10.1136/bmj.328.7430.22.
27. Mhaskar R, Djulbegovic B, Magazin A, Soares HP, Kumar A. Published methodological quality of randomized controlled trials does not reflect the actual quality assessed in protocols. J Clin Epidemiol. 2012;65(6):602-9. doi: 10.1016/j.jclinepi.2011.10.016.
28. Brunoni AR, Tadini L, Fregni F. Changes in clinical trials methodology over time: a systematic review of six decades of research in psychopharmacology. PLoS One. 2010;5(3):e9479. doi: 10.1371/journal.pone.0009479.
29. Masi A, Lampit A, DeMayo MM, Glozier N, Hickie IB, Guastella AJ. A comprehensive systematic review and meta-analysis of pharmacological and dietary supplement interventions in paediatric autism: moderators of treatment response and recommendations for future research. Psychol Med. 2017;47(7):1323-34. doi: 10.1017/s0033291716003457.
30. Masi A, Lampit A, Glozier N, Hickie IB, Guastella AJ. Predictors of placebo response in pharmacological and dietary supplement treatment trials in pediatric autism spectrum disorder: a meta-analysis. Translational psychiatry. 2015;5:e640. doi: 10.1038/tp.2015.143.
31. King BH, Dukes K, Donnelly CL, Sikich L, McCracken JT, Scahill L, et al. Baseline factors predicting placebo response to treatment in children and adolescents with autism spectrum disorders: a multisite randomized clinical trial. JAMA pediatrics. 2013;167(11):1045–52.
32. Aman MG, Novotny S, Samango-Sprouse C, Lecavalier L, Leonard E, Gadow KD, et al. Outcome measures for clinical drug trials in autism. CNS Spectr. 2004;9(1):36-47.
33. Scahill L, Aman MG, Lecavalier L, Halladay AK, Bishop SL, Bodfish JW, et al. Measuring repetitive behaviors as a treatment endpoint in youth with autism spectrum disorder. Autism. 2013;19(1):38-52. doi: 10.1177/1362361313510069.
34. Furukawa TA, Cipriani A, Barbui C, Brambilla P, Watanabe N. Imputing response rates from means and standard deviations in meta-analyses. Int Clin Psychopharmacol. 2005;20(1):49-52.

35. Samara MT, Spineli LM, Furukawa TA, Engel RR, Davis JM, Salanti G, et al. Imputation of response rates from means and standard deviations in schizophrenia. Schizophr Res. 2013;151(1-3):209-14. doi: 10.1016/j.schres.2013.10.029.
36. Siafis S, Rodolico A, Çıray O, Murphy DG, Parellada M, Arango C, et al. Imputing the Number of Responders from the Mean and Standard Deviation of CGI-Improvement in Clinical Trials Investigating Medications for Autism Spectrum Disorder. Brain Sci. 2021;11(7). doi: 10.3390/brainsci11070908.
37. Shi J, Luo D, Weng H, Zeng XT, Lin L, Chu H, et al. Optimally estimating the sample standard deviation from the five-number summary. Research Synthesis Methods. 2020.
38. Morris SB, DeShon RP. Combining effect size estimates in meta-analysis with repeated measures and independent-groups designs. Psychological methods. 2002;7(1):105-25.
39. Furukawa TA, Barbui C, Cipriani A, Brambilla P, Watanabe N. Imputing missing standard deviations in meta-analyses can provide accurate results. J Clin Epidemiol. 2006;59(1):7-10. doi: 10.1016/j.jclinepi.2005.06.006.
40. Balk EM, Earley A, Patel K, Trikalinos TA, Dahabreh IJ. Empirical assessment of within-arm correlation imputation in trials of continuous outcomes. 2012.
41. Doi SA, Furuya-Kanamori L, Xu C, Lin L, Chivese T, Thalib L. Questionable utility of the relative risk in clinical research: a call for change to practice. J Clin Epidemiol. 2020. doi: 10.1016/j.jclinepi.2020.08.019.
42. Bakbergenuly I, Hoaglin DC, Kulinskaya E. Pitfalls of using the risk ratio in meta-analysis. Research Synthesis Methods. 2019;10(3):398-419. doi: https://doi.org/10.1002/jrsm.1347.
43. Panagiotou OA, Contopoulos-Ioannidis DG, Ioannidis JP. Comparative effect sizes in randomised trials from less developed and more developed countries: meta-epidemiological assessment. Bmj. 2013;346:f707. doi: 10.1136/bmj.f707.

eAppendix-3 Database search strategy

3.1 Electronic search 30
3.1.1 Multiple electronic database search on the 8th July 2018 30
3.1.2 PubMed update search on the 4th July 2019 30
3.1.3 PubMed update search on the 4th July 2020 30
3.1.4 PubMed and CENTRAL update search on the 31st August 2020 30
3.1.5 PubMed and CENTRAL update on the 17th September 2021 31
3.1.6 PubMed and CENTRAL update on the 3rd November 2021 31
3.2. Search Strategies 32
3.2.1 ClinicalTrials.Gov 32
3.2.2 Cochrane Central Register of Controlled Trials (CENTRAL) 32
3.2.3 EMBASE 34
3.2.4 MEDLINE 37
3.2.5 PsycINFO 40
3.2.6 PubMed 42
3.2.7 World Health Organization International Clinical Trials Registry Platform (WHO ICTRP) 47
3.3 References 48

3.1 Electronic search

3.1.1 Multiple electronic database search on the 8th July 2018

We searched the following resources on the 8th July 2018 with no date/time, language, document type, and publication status limitations:

- ClinicalTrials.Gov (Until search date)
- Cochrane Central Register of Controlled Trials (CENTRAL) (Until search date)
- EMBASE (1974 to 2018 week 28)
- MEDLINE (1946 – search date)
- PsycINFO (1806 to July week 1 2018)
- PubMed (1946 – search date)
- World Health Organization International Clinical Trials Registry Platform (WHO ICTRP) (Until search date)

We followed the Cochrane Handbook [1] and MECIR [2] for conducting the search, PRISMA guideline [3] for reporting the search, and PRESS checklist for peer-reviewing the search strategies [4]. Keywords and search concepts were collected through experts' opinion, existing recent survey[5], controlled vocabulary (APA Thesaurus, Medical Subject Headings = MeSH, and Excerpta Medica Tree = EMTREE), text mining using the Yale MeSH Analyzer [6], and reviewing the primary search results. In addition, we utilized the report from Canadian Agency for Drugs and Technologies in Health (CADTH) to focus the search employing EMTREE [7]. We used existing search filters such as Eisinga's animal search filter to remove the non-human search results in EMBASE [8] and Cochrane's Randomized Controlled Trials search filters for EMBASE, MEDLINE, and PubMed [1]. Because of poor reporting of outcomes in medical research [9-13], we did not limit the search adding specific outcomes so that we could have all the outcomes. Search strategies, developed by assistance of a medical information specialist, were reported in eAppendix-3.2. The duplicate search results were detected based on title match and deleted after approval through manual check in EndNote X8.

The search found 13,803 references. Removing 6,455 duplicate records, we screened 7,348 records.

3.1.2 PubMed update search on the 4th July 2019

An update search on PubMed was conducted on 4th July 2019. The search found 191 new records, which were screened.

3.1.3 PubMed update search on the 4th July 2020

An update search on PubMed was conducted on the 30th April 2020. The search found 158 records, which were screened.

3.1.4 PubMed and CENTRAL update search on the 31st August 2020

An update search on PubMed and CENTRAL was conducted on 31st August 2020. The search found 70 records in PubMed and 599 in CENTRAL, which were screened.

3.1.5 PubMed and CENTRAL update on the 17th September 2021

An update search on PubMed and CENTRAL was conducted on the 17th September 2021. The search found 315 records in PubMed and 126 in CENTRAL, which were screened.

3.1.6 PubMed and CENTRAL update on the 3rd November 2021

An update search on PubMed and CENTRAL was conducted on the 3rd November 2021. The search found 32 records in PubMed and 28 in CENTRAL, which were screened.

3.2. Search Strategies

3.2.1 ClinicalTrials.Gov

Advanced Search

Condition or Disease: Autism Spectrum Disorder OR Autistic Disorder OR Asperger Syndrome OR Rett Syndrome OR "Child Development Disorders, Pervasive" OR Childhood Disintegrative Disorder OR Hyperammonemia

Study Type: Interventional Studies (Clinical Trials)

3.2.2 Cochrane Central Register of Controlled Trials (CENTRAL)

([mh "Autistic Disorder"] OR [mh "Autism Spectrum Disorder"] OR [mh "Asperger Syndrome"] OR [mh "Rett Syndrome"] OR [mh "Child Development Disorders, Pervasive"] OR (Autis* OR Kanner* OR Asperger* OR "Pervasive Child Development Disorder" OR "Pervasive Child Development Disorders" OR "Pervasive Developmental Disorder" OR "Pervasive Developmental Disorders" OR "Pervasive Development Disorder" OR "Pervasive Development Disorders" OR "Childhood Disintegrative Disorder" OR "Childhood Disintegrative Disorders" OR Rett* OR "Cerebroatrophic Hyperammonemia" OR "Cerebroatrophic Hyperammonemias"):ti,ab) AND ([mh ^"Pharmaceutical Preparations"] OR [mh "Drug Combinations"] OR [mh "Drugs, Chinese Herbal"] OR [mh "Drugs, Essential"] OR [mh "Drugs, Generic"] OR [mh "Drugs, Investigational"] OR [mh "Nonprescription Drugs"] OR [mh "Plant Extracts"] OR [mh "Prescription Drugs"] OR [mh Prodrugs] OR [mh "Pharmacologic Actions"] OR [mh "Drug Therapy"] OR [mh "Therapeutic Uses"] OR [mh "Physiological Effects of Drugs"] OR [mh Aripiprazole] OR [mh Clozapine] OR [mh Haloperidol] OR [mh Loxapine] OR [mh "Lurasidone Hydrochloride"] OR [mh "Paliperidone Palmitate"] OR [mh "Quetiapine Fumarate"] OR [mh Risperidone] OR [mh Sulpiride] OR [mh Citalopram] OR [mh Clomipramine] OR [mh Desipramine] OR [mh Fluoxetine] OR [mh Fluvoxamine] OR [mh Imipramine] OR [mh Mianserin] OR [mh Nortriptyline] OR [mh Paroxetine] OR [mh Sertraline] OR [mh "Venlafaxine Hydrochloride"] OR [mh "Atomoxetine Hydrochloride"] OR [mh Dextromethorphan] OR [mh Fenfluramine] OR [mh "Lisdexamfetamine Dimesylate"] OR [mh "Lithium Carbonate"] OR [mh Methylphenidate] OR [mh "N-Methyl-3,4-methylenedioxyamphetamine"] OR [mh "Valproic Acid"] OR [mh Betahistine] OR [mh Bromocriptine] OR [mh Buspirone] OR [mh Cyproheptadine] OR [mh Famotidine] OR [mh Levodopa] OR [mh Sumatriptan] OR [mh Clonidine] OR [mh Guanfacine] OR [mh Dexmedetomidine] OR [mh Propranolol] OR [mh Acetylcysteine] OR [mh Amantadine] OR [mh Ketamine] OR [mh Memantine] OR [mh Riluzole] OR [mh Baclofen] OR [mh Bumetanide] OR [mh Flumazenil] OR [mh Galantamine] OR [mh Mecamylamine] OR [mh Pregnenolone] OR [mh Rivastigmine] OR [mh Varenicline] OR [mh Cannabidiol] OR [mh Celecoxib] OR [mh Everolimus] OR [mh "Fingolimod Hydrochloride"] OR [mh Fluconazole] OR [mh "Glatiramer Acetate"] OR ([mh Immunoglobulins] AND [mh "Administration, Oral"]) OR [mh Minocycline] OR [mh Naltrexone] OR [mh Pentoxifylline] OR [mh Sirolimus] OR [mh Staurosporine] OR [mh Suramin] OR [mh Tacrolimus] OR [mh "Insulin-Like Growth Factor I"] OR [mh "Adrenal Cortex Hormones"] OR [mh "Adrenocorticotropic Hormone"] OR [mh Angiotensins] OR [mh Carnitine] OR [mh "Diet Therapy"] OR [mh "Dietary Supplements"] OR [mh "Fatty Acids, Omega-3"] OR [mh "Gastrin-Releasing Peptide"] OR [mh Ghrelin] OR [mh Hydrocortisone] OR [mh Lovastatin] OR [mh Melanocortins] OR [mh Melatonin] OR [mh Metformin] OR [mh Minerals] OR [mh "Nutrition Therapy"] OR [mh Oligosaccharides] OR [mh Oxytocin] OR [mh Prednisone] OR [mh Probiotics] OR [mh Pyridoxine] OR [mh Secretin] OR [mh "Thyroid Hormones"] OR [mh Thyroxine] OR [mh Triiodothyronine] OR [mh Vasopressins] OR [mh "Vitamin E"] OR [mh "Arachidonic Acid"] OR [mh "Ascorbic Acid"] OR

[mh Carnosine] OR [mh "Docosahexaenoic Acids"] OR [mh "Folic Acid"] OR [mh "Ginkgo biloba"] OR [mh Glutathione] OR [mh Glutens] OR [mh Inositol] OR [mh Leucovorin] OR [mh Magnesium] OR [mh "Magnesium Oxide"] OR [mh Milk] OR [mh Papain] OR [mh Succimer] OR [mh "Vitamin B 12"] OR [mh "Vitamin B 6"] OR [mh "Vitamin D"] OR [mh "Adrenergic alpha-2 Receptor Antagonists"] OR [mh "Antidepressive Agents, Second-Generation"] OR [mh "Anti-Dyskinesia Agents"] OR [mh Antiemetics] OR [mh "Antipsychotic Agents"] OR [mh "Dopamine Agonists"] OR [mh "Dopamine Antagonists"] OR [mh "Dopamine D2 Receptor Antagonists"] OR [mh "GABA Antagonists"] OR [mh "Serotonin 5-HT2 Receptor Antagonists"] OR [mh "Serotonin Agents"] OR [mh "Serotonin Antagonists"] OR [mh "Serotonin Uptake Inhibitors"] OR [mh "Adrenergic alpha-Antagonists"] OR [mh "Adrenergic Uptake Inhibitors"] OR [mh "Cytochrome P-450 CYP1A2 Inhibitors"] OR [mh "Cytochrome P-450 CYP2C19 Inhibitors"] OR [mh "Cytochrome P-450 CYP2D6 Inhibitors"] OR [mh "Enzyme Inhibitors"] OR [mh "Histamine H1 Antagonists"] OR [mh "Serotonin and Noradrenaline Reuptake Inhibitors"] OR [mh "Anti-Anxiety Agents"] OR [mh "Antidepressive Agents"] OR [mh "Antidepressive Agents, Tricyclic"] OR [mh "Psychotropic Drugs"] OR [mh Anticonvulsants] OR [mh "Antimanic Agents"] OR [mh "Calcium Channel Blockers"] OR [mh "Central Nervous System Stimulants"] OR [mh "Cytochrome P-450 CYP3A Inducers"] OR [mh "Dopamine Uptake Inhibitors"] OR [mh "Excitatory Amino Acid Antagonists"] OR [mh "GABA Agents"] OR [mh Hallucinogens] OR [mh "Neuroprotective Agents"] OR [mh "Nootropic Agents"] OR [mh "Serotonin Receptor Agonists"] OR [mh "Voltage-Gated Sodium Channel Blockers"] OR [mh "Antiparkinson Agents"] OR [mh "Dopamine Agents"] OR [mh "Histamine Agonists"] OR [mh "Histamine H2 Antagonists"] OR [mh "Hormone Antagonists"] OR [mh "Serotonin 5-HT1 Receptor Agonists"] OR [mh "Adrenergic alpha-2 Receptor Agonists"] OR [mh "Adrenergic beta-Antagonists"] OR [mh Sympatholytics] OR [mh "Excitatory Amino Acid Agonists"] OR [mh "Cholinesterase Inhibitors"] OR [mh "GABA Modulators"] OR [mh "GABA-B Receptor Agonists"] OR [mh "Ganglionic Blockers"] OR [mh "Nicotinic Agonists"] OR [mh "Nicotinic Antagonists"] OR [mh Parasympathomimetics] OR [mh "Sodium Potassium Chloride Symporter Inhibitors"] OR [mh "14-alpha Demethylase Inhibitors"] OR [mh "Adjuvants, Immunologic"] OR [mh "Anti-Bacterial Agents"] OR [mh "Antifungal Agents"] OR [mh "Cannabinoid Receptor Agonists"] OR [mh "Cannabinoid Receptor Antagonists"] OR [mh "Cannabinoid Receptor Modulators"] OR [mh "Central Nervous System Agents"] OR [mh "Cyclooxygenase 2 Inhibitors"] OR [mh "Cytochrome P-450 CYP2C9 Inhibitors"] OR [mh "Immunologic Factors"] OR [mh "Immunosuppressive Agents"] OR [mh "Narcotic Antagonists"] OR [mh "Neurotransmitter Agents"] OR [mh "Purinergic Agents"] OR [mh "Anti-Inflammatory Agents"] OR [mh Antioxidants] OR [mh "Central Nervous System Depressants"] OR [mh "Chelating Agents"] OR [mh Hormones] OR [mh Oxytocics] OR [mh "Vitamin B Complex"] OR [mh Vitamins] OR (Anticonvuls* OR Antiepilep* OR Antipsychotic* OR Psychotropic* OR "Anti-Anxiety" OR Anxiolytic* OR Antidepress* OR "Pharmaco-Therapy" OR "Pharmaco-Therapies" OR Chemotherapy OR Chemotherapies OR Pharmacotherapy OR Pharmacotherapies OR "Pharmacological Interventions" OR "Pharmacological Intervention" OR "Pharmacological Treatment" OR "Pharmacological Treatments" OR "Drug Therapy" OR "Drug Therapies" OR Amisulpride OR Aripiprazol* OR Abilify OR Brexpiprazole OR Clozapine OR Clozaril OR Leponex OR Haloperidol OR Haldol OR Loxapine OR Lurasidone OR Latuda OR Olanzapine OR Zyprexa OR Paliperidone OR Invega OR Quetiapine OR Seroquel OR Risperidone OR Risperdal OR Risperidal OR Sertindole OR Sulpiride OR Dogmatil OR Ziprasidone OR Geodon OR Ziprazidone OR Agomelatine OR Citalopram OR Clomipramine OR Desipramine OR Escitalopram OR Fluoxetine OR Prozac OR Fluvoxamine OR Imipramine OR Mianserin OR Milnacipran OR Mirtazapine OR Nortriptyline OR Paroxetine OR Sertraline OR Tianeptine OR Tianeptine OR Venlafaxine OR "m-chlorophenylpiperazine" OR "m-CPP" OR "1-(3-chlorophenyl)piperazine" OR Atomoxetine OR Strattera OR Dextromethorphan OR Fenfluramine OR Lamotrigine OR Levetiracetam OR Lisdexamfetamine OR Lithium OR MDMA OR "N-Methyl-3,4-methylenedioxyamphetamine" OR Ecstasy OR Methylenedioxymethamphetamine OR Methylphenidate OR Ritalin* OR Oxcarbazepine OR Topiramate OR Valproic Acid OR Divalproex OR Valproate OR Divalproate OR "(+)-5-FPT" OR

"PRX-07034" OR Betahistin* OR Bromocriptine OR Buspirone OR Cyproheptadine OR Famotidine OR Levodopa OR "L-Dopa" OR "LP-211" OR "N-(4-cyanophenylmethyl)-4-(2-diphenyl)-1-piperazinehexanamide" OR Sumatriptan OR Volinanserin OR "M100907" OR Clonidine OR Guanfacine OR Dexmedetomidine OR Propranolol OR Acetylcysteine OR "ADX71149" OR "JNJ-40411813" OR "1-butyl-3-chloro-4-(4-phenyl-1-piperidinyl)-(1H)-pyridone" OR Amantadine OR "AZD8529" OR Basimglurant OR "2-chloro-4-(1-(4-fluorophenyl)-2,5-dimethyl-1H-imidazol-4-ylethynyl)pyridine" OR "CDPPB" OR "3-cyano-N-(1,3-diphenyl-1H-pyrazol-5-yl)benzamide" OR "CX516" OR "BDP 12" OR "1-(quinoxalin-6-ylcarbonyl)piperidine" OR "D-Cycloserine" OR Eglumetad OR Fenobam OR "GRN-529" OR Ketamine OR "LY 341495" OR "LY341495" OR "LY 379268" OR "LY379268" OR "LY 487379" OR "LY487379" OR Mavoglurant OR Memantine OR "MGS0039" OR "MPEP" OR "6-methyl-2-(phenylethynyl)pyridine" OR "MPX-004" OR "MPX-007" OR "MTEP" OR "NCFP" OR Riluzole OR "RO4491533" OR "TASP0433864" OR "A 867744" OR "4-(5-(4-chlorophenyl)-2-methyl-3-propionyl-1H-pyrrol-1-yl)benzenesulfonamide" OR Acamprosate OR "ADX71441" OR Arbaclofen OR "AZD7325" OR Baclofen OR Bumetanide OR "DMXB A" OR "DMXBA" OR "GTS 21" OR "3-(2,4-dimethoxybenzylidene)anabaseine" OR Donepezil OR "EVP-6124" OR "7-chloro-N-quinuclidin-3-yl-benzo(b)thiophene-2-carboxamide" OR Flumazenil OR Galantamin* OR Mecamylamine OR "PNU 120596" OR "PNU120596" OR "1-(5-chloro-2,4-dimethoxyphenyl)-3-(5-methylisoxazol-3-yl)urea" OR Pregnenolone OR Rivastigmine OR "SSR180711" OR Varenicline OR "AF38469" OR "AR-A014418" OR "N-(4-methoxybenzyl)-N'-(5-nitro-1,3-thiazol-2-yl)urea" OR Cannabidiol OR Cannabidivarin OR Celecoxib OR Everolimus OR Fingolimod OR Fluconazole OR Glatiramer OR Hydroxyfasudil OR Lenalidomide OR Minocycline OR Naltrexone OR "NVP-BKM120" OR Buparlisib OR "Oral Human Immunoglobulin" OR Pentoxifylline OR "SB 216763" OR "SB216763" OR Sirolimus OR Rapamycin OR Staurosporine OR Suramin OR Tacrolimus OR "TAK-242" OR "TAK242" OR Temsirolimus OR Tideglusib OR "NP031112" OR Amastatin OR Angiotensin* OR Carnitine OR Levocarnitine OR "CM-AT" OR "Diet Therapy" OR "Diet Therapies" OR "Dietary Supplements" OR "Dietary Supplement" OR Dimethylglycine OR "EPI-743" OR "alpha-Tocotrienol Quinone" OR "Food Supplement" OR "Food Supplements" OR "Gastrin-Releasing Peptide" OR Ghrelin OR "Herbal Supplement" OR "Herbal Supplements" OR Hormone* OR Corticosteroid* OR Corticoid* OR Hydrocortisone OR "IGF-1" OR "Insulin-Like Growth Factor I" OR Lovastatin OR Melanocortin* OR Melatonin OR Metformin OR Mineral* OR "NNZ-2566" OR "NNZ2566" OR "Nutrition Therapy" OR "Nutritional Therapy" OR Oligosaccharide* OR "Omega-3" OR "Omega3" OR "n-3 Fatty" OR "n-3 Polyunsaturated Fatty" OR "n-3 PUFA" OR "n3 PUFA" OR "n 3 Oils" OR "n 3 Oil" OR "n3 Fatty" OR "ORG-2766" OR Oxytocin OR Syntocinon OR Pioglitazone OR Prednisone OR Probiotic* OR Bifidobacter* OR Pyridoxine OR "RG7713" OR "Ro27 3225" OR Secretin OR Sulforaphane OR Sulforafan OR Tetrahydrobiopterin OR Sapropterin OR Thyroxine OR Triiodothyronine OR "T3" OR Trofinetide OR Vasopressin* OR Vitamin* OR "WAY-267464" OR "WAY267464" OR Arachidonic OR Arachidonate OR Ascorbic OR Ascorbate OR Carnosine OR Cyanocobalamin OR Cobalamin* OR Cobamide* OR Hydroxocobalamin OR Docosahexaenoic OR Docosahexaenoate OR Ferrous OR Folic OR Folate OR Ginkgo* OR Gingko* OR Ginko* OR Maidenhair OR Glutathione OR Gluten OR Inositol OR Leucovorin OR Folinic OR Magnesium OR Milk OR Papain OR Pepsin OR Pyridoxal OR Pyridoxamine OR Succimer OR Dimercaptosuccinic Acid OR DMSA OR "Trichuris Suis" OR Ubiquinol):ti,ab)
In Trials

3.2.3 EMBASE

1. Exp Autism/ OR "Asperger Syndrome"/ OR "Childhood Disintegrative Disorder"/ OR "Pervasive Developmental Disorder Not Otherwise Specified"/ OR "Rett Syndrome"/ OR (Autis* OR Kanner* OR Asperger* OR "Pervasive Child Development Disorder" OR "Pervasive Child Development Disorders" OR "Pervasive Developmental Disorder" OR "Pervasive

Developmental Disorders" OR "Pervasive Development Disorder" OR "Pervasive Development Disorders" OR "Childhood Disintegrative Disorder" OR "Childhood Disintegrative Disorders" OR Rett* OR "Cerebroatrophic Hyperammonemia" OR "Cerebroatrophic Hyperammonemias").ti,ab.

2. Exp "Chemicals And Drugs"/ OR Exp *Drug/ OR *Behind the Counter Drug/ OR *Chinese Drug/ OR *Essential Drug/ OR *Generic Drug/ OR *Long Acting Drug/ OR *New Drug/ OR *Non Prescription Drug/ OR *Orphan Drug/ OR *Prescription Drug/ OR *Prodrug/ OR *Short Acting Drug/ OR *Unclassified Drug/ OR *Unindexed Drug/ OR Exp *Medicinal Plant/ OR Exp *Plant Extract/ OR Exp "Drug Combination"/ OR Exp *Drug Therapy/ OR Exp *Drug Effect/ OR Aripiprazole/ OR Clozapine/ OR Haloperidol/ OR Loxapine/ OR Lurasidone/ OR Paliperidone/ OR Quetiapine/ OR Risperidone/ OR Sulpiride/ OR Citalopram/ OR Clomipramine/ OR Desipramine/ OR Fluoxetine/ OR Fluvoxamine/ OR Imipramine/ OR Mianserin/ OR Nortriptyline/ OR Paroxetine/ OR Sertraline/ OR Venlafaxine/ OR Atomoxetine/ OR Dextromethorphan/ OR Fenfluramine/ OR Lisdexamfetamine/ OR "Lithium Carbonate"/ OR Methylphenidate/ OR Midomafetamine/ OR "Valproic Acid"/ OR Betahistine/ OR Bromocriptine/ OR Buspirone/ OR Cyproheptadine/ OR Famotidine/ OR Levodopa/ OR Sumatriptan/ OR Clonidine/ OR Guanfacine/ OR Dexmedetomidine/ OR Propranolol/ OR Acetylcysteine/ OR Amantadine/ OR Ketamine/ OR Memantine/ OR Riluzole/ OR Baclofen/ OR Bumetanide/ OR Flumazenil/ OR Galantamine/ OR Mecamylamine/ OR Pregnenolone/ OR Rivastigmine/ OR Varenicline/ OR Cannabidiol/ OR Celecoxib/ OR Everolimus/ OR Fingolimod/ OR Fluconazole/ OR Glatiramer/ OR (Immunoglobulin/ AND "Oral Drug Administration"/) OR Minocycline/ OR Naltrexone/ OR Pentoxifylline/ OR Rapamycin/ OR Staurosporine/ OR Suramin/ OR Tacrolimus/ OR "Somatomedin C"/ OR Exp *Corticosteroid/ OR Corticotropin/ OR Exp *Angiotensin Derivative/ OR Carnitine/ OR Exp *Diet Therapy/ OR *Diet Supplementation/ OR "Omega 3 Fatty Acid"/ OR "Gastrin Releasing Peptide"/ OR Ghrelin/ OR Hydrocortisone/ OR Mevinolin/ OR Melanocortin/ OR Melatonin/ OR Metformin/ OR Mineral/ OR Exp *Oligosaccharide/ OR Oxytocin/ OR Prednisone/ OR Probiotic Agent/ OR Pyridoxine/ OR Secretin/ OR Thyroid Hormone/ OR Thyroxine/ OR Liothyronine/ OR Exp *Vasopressin Derivative/ OR "alpha Tocopherol"/ OR "Arachidonic Acid"/ OR "Ascorbic Acid"/ OR Carnosine/ OR "Docosahexaenoic Acid"/ OR "Folic Acid"/ OR "Ginkgo biloba"/ OR Glutathione/ OR Gluten/ OR Inositol/ OR Folinic Acid/ OR Magnesium/ OR "Magnesium Oxide"/ OR Milk/ OR Papain/ OR Succimer/ OR Cyanocobalamin/ OR Pyridoxine/ OR "Vitamin D"/ OR Exp "alpha 2 Adrenergic Receptor Blocking Agent"/ OR Exp "Antidepressant Agent"/ OR Exp "Antiparkinson Agent"/ OR Exp "Antiemetic Agent"/ OR Exp "Neuroleptic Agent"/ OR Exp "Dopamine Receptor Stimulating Agent"/ OR Exp "Dopamine Receptor Blocking Agent"/ OR Exp "Dopamine 2 Receptor Blocking Agent"/ OR Exp "4 Aminobutyric Acid Receptor Blocking Agent"/ OR Exp "Serotonin 2 Antagonist"/ OR Exp "Serotonin Receptor Affecting Agent"/ OR Exp "Serotonin Antagonist"/ OR Exp "Serotonin Uptake Inhibitor"/ OR Exp "alpha Adrenergic Receptor Blocking Agent"/ OR Exp "Adrenergic Receptor Affecting Agent"/ OR Exp "Cytochrome P450 1A2 Inhibitor"/ OR Exp "Cytochrome P450 2C19 Inhibitor"/ OR Exp "Cytochrome P450 2D6 Inhibitor"/ OR Exp "Enzyme Inhibitor"/ OR Exp "Histamine H1 Receptor Antagonist"/ OR Exp Noradrenalin Uptake Inhibitor/ OR Exp "Serotonin Uptake Inhibitor"/ OR Exp "Anxiolytic Agent"/ OR Exp "Tricyclic Antidepressant Agent"/ OR Exp "Psychotropic Agent"/ OR Exp "Anticonvulsive Agent"/ OR Exp Tranquilizer/ OR Exp "Calcium Channel Blocking Agent"/ OR Exp "Central Stimulant Agent"/ OR Exp "Cytochrome P450 3A Inducer"/ OR Exp "Dopamine Uptake Inhibitor"/ OR Exp "Amino Acid Receptor Blocking Agent"/ OR Exp "GABAergic Receptor Affecting Agent"/ OR Exp "Psychedelic Agent"/ OR Exp "Neuroprotective Agent"/ OR Exp "Nootropic Agent"/ OR Exp "Serotonin Agonist"/ OR Exp "Voltage Gated Sodium Channel Blocking Agent"/ OR Exp "Histamine Agonist"/ OR Exp "Histamine H2 Receptor Antagonist"/ OR Exp "Hormone Antagonist"/ OR Exp "Serotonin 1 Agonist"/ OR Exp "alpha 2 Adrenergic Receptor Stimulating Agent"/ OR Exp "beta Adrenergic Receptor Blocking Agent"/ OR Exp "Adrenergic Receptor Blocking Agent"/ OR Exp "Amino

Acid Receptor Stimulating Agent"/ OR Exp "Cholinesterase Inhibitor"/ OR Exp "Benzodiazepine Receptor Affecting Agent"/ OR Exp "4 Aminobutyric Acid B Receptor Stimulating Agent"/ OR Exp "Ganglion Blocking Agent"/ OR Exp "Nicotinic Agent"/ OR Exp "Nicotinic Receptor Blocking Agent"/ OR Exp "Cholinergic Receptor Stimulating Agent"/ OR Exp "Loop Diuretic Agent"/ OR Exp "Sterol 14alpha Demethylase Inhibitor"/ OR Exp "Immunological Adjuvant"/ OR Exp "Antiinfective Agent"/ OR Exp "Antifungal Agent"/ OR Exp "Cannabinoid Receptor Agonist"/ OR Exp "Cannabinoid Receptor Antagonist"/ OR Exp "Cannabinoid Receptor Affecting Agent"/ OR Exp "Central Nervous System Agents"/ OR Exp "Cyclooxygenase 2 Inhibitor"/ OR Exp "Cytochrome P450 2C9 Inhibitor"/ OR Exp "Immunologic Factor"/ OR Exp "Immunosuppressive Agent"/ OR Exp "Narcotic Antagonist"/ OR Exp "Agents Interacting With Transmitter, Hormone OR Drug Receptors"/ OR Exp "Purinergic Receptor Affecting Agent"/ OR Exp "Antiinflammatory Agent"/ OR Exp Antioxidant/ OR Exp "Central Depressant Agent"/ OR Exp "Chelating Agent"/ OR Exp Hormone/ OR Exp "Oxytocic Agent"/ OR Exp "Vitamin B Complex"/ OR Exp Vitamin/ OR (Anticonvuls* OR Antiepilep* OR Antipsychotic* OR Psychotropic* OR "Anti-Anxiety" OR Anxiolytic* OR Antidepress* OR "Pharmaco-Therapy" OR "Pharmaco-Therapies" OR Chemotherapy OR Chemotherapies OR Pharmacotherapy OR Pharmacotherapies OR "Pharmacological Interventions" OR "Pharmacological Intervention" OR "Pharmacological Treatment" OR "Pharmacological Treatments" OR "Drug Therapy" OR "Drug Therapies" OR Amisulpride OR Aripiprazol* OR Abilify OR Brexpiprazole OR Clozapine OR Clozaril OR Leponex OR Haloperidol OR Haldol OR Loxapine OR Lurasidone OR Latuda OR Olanzapine OR Zyprexa OR Paliperidone OR Invega OR Quetiapine OR Seroquel OR Risperidone OR Risperdal OR Risperidal OR Sertindole OR Sulpiride OR Dogmatil OR Ziprasidone OR Geodon OR Ziprazidone OR Agomelatine OR Citalopram OR Clomipramine OR Desipramine OR Escitalopram OR Fluoxetine OR Prozac OR Fluvoxamine OR Imipramine OR Mianserin OR Milnacipran OR Mirtazapine OR Nortriptyline OR Paroxetine OR Sertraline OR Tianeptine OR Tianeptine OR Venlafaxine OR "m-chlorophenylpiperazine" OR "m-CPP" OR "1-(3-chlorophenyl)piperazine" OR Atomoxetine OR Strattera OR Dextromethorphan OR Fenfluramine OR Lamotrigine OR Levetiracetam OR Lisdexamfetamine OR Lithium OR MDMA OR "N-Methyl-3,4-methylenedioxyamphetamine" OR Ecstasy OR Methylenedioxymethamphetamine OR Methylphenidate OR Ritalin* OR Oxcarbazepine OR Topiramate OR Valproic Acid OR Divalproex OR Valproate OR Divalproate OR "(+)-5-FPT" OR "PRX-07034" OR Betahistin* OR Bromocriptine OR Buspirone OR Cyproheptadine OR Famotidine OR Levodopa OR "L-Dopa" OR "LP-211" OR "N-(4-cyanophenylmethyl)-4-(2-diphenyl)-1-piperazinehexanamide" OR Sumatriptan OR Volinanserin OR "M100907" OR Clonidine OR Guanfacine OR Dexmedetomidine OR Propanolol OR Acetylcysteine OR "ADX71149" OR "JNJ-40411813" OR "1-butyl-3-chloro-4-(4-phenyl-1-piperidinyl)-(1H)-pyridone" OR Amantadine OR "AZD8529" OR Basimglurant OR "2-chloro-4-(1-(4-fluorophenyl)-2,5-dimethyl-1H-imidazol-4-ylethynyl)pyridine" OR "CDPPB" OR "3-cyano-N-(1,3-diphenyl-1H-pyrazol-5-yl)benzamide" OR "CX516" OR "BDP 12" OR "1-(quinoxalin-6-ylcarbonyl)piperidine" OR "D-Cycloserine" OR Eglumetad OR Fenobam OR "GRN-529" OR Ketamine OR "LY 341495" OR "LY341495" OR "LY 379268" OR "LY379268" OR "LY 487379" OR "LY487379" OR Mavoglurant OR Memantine OR "MGS0039" OR "MPEP" OR "6-methyl-2-(phenylethynyl)pyridine" OR "MPX-004" OR "MPX-007" OR "MTEP" OR "NCFP" OR Riluzole OR "RO4491533" OR "TASP0433864" OR "A 867744" OR "4-(5-(4-chlorophenyl)-2-methyl-3-propionyl-1H-pyrrol-1-yl)benzenesulfonamide" OR Acamprosate OR "ADX71441" OR Arbaclofen OR "AZD7325" OR Baclofen OR Bumetanide OR "DMXB A" OR "DMXBA" OR "GTS 21" OR "3-(2,4-dimethoxybenzylidene)anabaseine" OR Donepezil OR "EVP-6124" OR "7-chloro-N-quinuclidin-3-yl-benzo(b)thiophene-2-carboxamide" OR Flumazenil OR Galantamin* OR Mecamylamine OR "PNU 120596" OR "PNU120596" OR "1-(5-chloro-2,4-dimethoxyphenyl)-3-(5-methylisoxazol-3-yl)urea" OR Pregnenolone OR Rivastigmine OR "SSR180711" OR Varenicline OR "AF38469" OR "AR-A014418" OR "N-(4-methoxybenzyl)-

N'-(5-nitro-1,3-thiazol-2-yl)urea" OR Cannabidiol OR Cannabidivarin OR Celecoxib OR Everolimus OR Fingolimod OR Fluconazole OR Glatiramer OR Hydroxyfasudil OR Lenalidomide OR Minocycline OR Naltrexone OR "NVP-BKM120" OR Buparlisib OR "Oral Human Immunoglobulin" OR Pentoxifylline OR "SB 216763" OR "SB216763" OR Sirolimus OR Rapamycin OR Staurosporine OR Suramin OR Tacrolimus OR "TAK-242" OR "TAK242" OR Temsirolimus OR Tideglusib OR "NP031112" OR Amastatin OR Angiotensin* OR Carnitine OR Levocarnitine OR "CM-AT" OR "Diet Therapy" OR "Diet Therapies" OR "Dietary Supplements" OR "Dietary Supplement" OR Dimethylglycine OR "EPI-743" OR "alpha-Tocotrienol Quinone" OR "Food Supplement" OR "Food Supplements" OR "Gastrin-Releasing Peptide" OR Ghrelin OR "Herbal Supplement" OR "Herbal Supplements" OR Hormone* OR Corticosteroid* OR Corticoid* OR Hydrocortisone OR "IGF-1" OR "Insulin-Like Growth Factor I" OR Lovastatin OR Melanocortin* OR Melatonin OR Metformin OR Mineral* OR "NNZ-2566" OR "NNZ2566" OR "Nutrition Therapy" OR "Nutritional Therapy" OR Oligosaccharide* OR "Omega-3" OR "Omega3" OR "n-3 Fatty" OR "n-3 Polyunsaturated Fatty" OR "n-3 PUFA" OR "n3 PUFA" OR "n 3 Oils" OR "n 3 Oil" OR "n3 Fatty" OR "ORG-2766" OR Oxytocin OR Syntocinon OR Pioglitazone OR Prednisone OR Probiotic* OR Bifidobacter* OR Pyridoxine OR "RG7713" OR "Ro27 3225" OR Secretin OR Sulforaphane OR Sulforafan OR Tetrahydrobiopterin OR Sapropterin OR Thyroxine OR Triiodothyronine OR "T3" OR Trofinetide OR Vasopressin* OR Vitamin* OR "WAY-267464" OR "WAY267464" OR Arachidonic OR Arachidonate OR Ascorbic OR Ascorbate OR Carnosine OR Cyanocobalamin OR Cobalamin* OR Cobamide* OR Hydroxocobalamin OR Docosahexaenoic OR Docosahexaenoate OR Ferrous OR Folic OR Folate OR Ginkgo* OR Gingko* OR Ginko* OR Maidenhair OR Glutathione OR Gluten OR Inositol OR Leucovorin OR Folinic OR Magnesium OR Milk OR Papain OR Pepsin OR Pyridoxal OR Pyridoxamine OR Succimer OR Dimercaptosuccinic Acid OR DMSA OR "Trichuris Suis" OR Ubiquinol).ti,ab.

3. Crossover Procedure/ OR Double Blind Procedure/ OR Randomized Controlled Trial/ OR Single Blind Procedure/ OR (Random* OR Factorial* OR Crossover* OR "Cross Over*" OR Placebo* OR (Doubl* adj Blind*) OR (Singl* adj Blind*) OR Assign* OR Allocat* OR Volunteer*).ti,ab.
4. 1 AND 2 AND 3
5. Exp Animals/ OR Exp Invertebrate/ OR Animal Experiment/ OR Animal Model/ OR Animal Tissue/ OR Animal Cell/ OR Nonhuman/
6. Human/ OR Normal Human/ OR Human Cell/
7. 5 AND 6
8. 5 NOT 7
9. 4 NOT 8

3.2.4 MEDLINE

1. "Autistic Disorder"/ OR "Autism Spectrum Disorder"/ OR "Asperger Syndrome"/ OR "Rett Syndrome"/ OR "Child Development Disorders, Pervasive"/ OR (Autis* OR Kanner* OR Asperger* OR "Pervasive Child Development Disorder" OR "Pervasive Child Development Disorders" OR "Pervasive Developmental Disorder" OR "Pervasive Developmental Disorders" OR "Pervasive Development Disorder" OR "Pervasive Development Disorders" OR "Childhood Disintegrative Disorder" OR "Childhood Disintegrative Disorders" OR Rett* OR "Cerebroatrophic Hyperammonemia" OR "Cerebroatrophic Hyperammonemias").ti,ab.
2. "Pharmaceutical Preparations"/ OR Exp "Drug Combinations"/ OR "Drugs, Chinese Herbal"/ OR "Drugs, Essential"/ OR "Drugs, Generic"/ OR "Drugs, Investigational"/ OR Exp "Nonprescription Drugs"/ OR Exp "Plant Extracts"/ OR "Prescription Drugs"/ OR Prodrugs/ OR "Pharmacologic Actions"/ OR Exp "Pharmacological Actions (Non MeSH)"/ OR Exp "Drug Therapy"/ OR Exp "Therapeutic Uses"/ OR Exp "Physiological Effects of Drugs"/ OR Aripiprazole/ OR Clozapine/ OR Haloperidol/ OR Loxapine/ OR "Lurasidone Hydrochloride"/ OR "Paliperidone Palmitate"/ OR "Quetiapine Fumarate"/ OR Risperidone/ OR Sulpiride/ OR

Citalopram/ OR Clomipramine/ OR Desipramine/ OR Fluoxetine/ OR Fluvoxamine/ OR Imipramine/ OR Mianserin/ OR Nortriptyline/ OR Paroxetine/ OR Sertraline/ OR "Venlafaxine Hydrochloride"/ OR "Atomoxetine Hydrochloride"/ OR Dextromethorphan/ OR Fenfluramine/ OR "Lisdexamfetamine Dimesylate"/ OR "Lithium Carbonate"/ OR Methylphenidate/ OR "N-Methyl-3,4-methylenedioxyamphetamine"/ OR "Valproic Acid"/ OR Betahistine/ OR Bromocriptine/ OR Buspirone/ OR Cyproheptadine/ OR Famotidine/ OR Levodopa/ OR Sumatriptan/ OR Clonidine/ OR Guanfacine/ OR Dexmedetomidine/ OR Propranolol/ OR Acetylcysteine/ OR Amantadine/ OR Ketamine/ OR Memantine/ OR Riluzole/ OR Baclofen/ OR Bumetanide/ OR Flumazenil/ OR Galantamine/ OR Mecamylamine/ OR Pregnenolone/ OR Rivastigmine/ OR Varenicline/ OR Cannabidiol/ OR Celecoxib/ OR Everolimus/ OR "Fingolimod Hydrochloride"/ OR Fluconazole/ OR "Glatiramer Acetate"/ OR (Immunoglobulins/ AND "Administration, Oral"/) OR Minocycline/ OR Naltrexone/ OR Pentoxifylline/ OR Sirolimus/ OR Staurosporine/ OR Suramin/ OR Tacrolimus/ OR "Insulin-Like Growth Factor I"/ OR "Adrenal Cortex Hormones"/ OR "Adrenocorticotropic Hormone"/ OR Angiotensins/ OR Carnitine/ OR "Diet Therapy"/ OR "Dietary Supplements"/ OR "Fatty Acids, Omega-3"/ OR "Gastrin-Releasing Peptide"/ OR Ghrelin/ OR Hydrocortisone/ OR Lovastatin/ OR Melanocortins/ OR Melatonin/ OR Metformin/ OR Minerals/ OR "Nutrition Therapy"/ OR Oligosaccharides/ OR Oxytocin/ OR Prednisone/ OR Probiotics/ OR Pyridoxine/ OR Secretin/ OR "Thyroid Hormones"/ OR Thyroxine/ OR Triiodothyronine/ OR Vasopressins/ OR "Vitamin E"/ OR "Arachidonic Acid"/ OR "Ascorbic Acid"/ OR Carnosine/ OR "Docosahexaenoic Acids"/ OR "Folic Acid"/ OR "Ginkgo biloba"/ OR Glutathione/ OR Glutens/ OR Inositol/ OR Leucovorin/ OR Magnesium/ OR "Magnesium Oxide"/ OR Milk/ OR Papain/ OR Succimer/ OR "Vitamin B 12"/ OR "Vitamin B 6"/ OR "Vitamin D"/ OR "Adrenergic alpha-2 Receptor Antagonists"/ OR "Antidepressive Agents, Second-Generation"/ OR "Anti-Dyskinesia Agents"/ OR Antiemetics/ OR "Antipsychotic Agents"/ OR "Dopamine Agonists"/ OR "Dopamine Antagonists"/ OR "Dopamine D2 Receptor Antagonists"/ OR "GABA Antagonists"/ OR "Serotonin 5-HT2 Receptor Antagonists"/ OR "Serotonin Agents"/ OR "Serotonin Antagonists"/ OR "Serotonin Uptake Inhibitors"/ OR "Adrenergic alpha-Antagonists"/ OR "Adrenergic Uptake Inhibitors"/ OR "Cytochrome P-450 CYP1A2 Inhibitors"/ OR "Cytochrome P-450 CYP2C19 Inhibitors"/ OR "Cytochrome P-450 CYP2D6 Inhibitors"/ OR "Enzyme Inhibitors"/ OR "Histamine H1 Antagonists"/ OR "Serotonin and Noradrenaline Reuptake Inhibitors"/ OR "Anti-Anxiety Agents"/ OR "Antidepressive Agents"/ OR "Antidepressive Agents, Tricyclic"/ OR "Psychotropic Drugs"/ OR Anticonvulsants/ OR "Antimanic Agents"/ OR "Calcium Channel Blockers"/ OR "Central Nervous System Stimulants"/ OR "Cytochrome P-450 CYP3A Inducers"/ OR "Dopamine Uptake Inhibitors"/ OR "Excitatory Amino Acid Antagonists"/ OR "GABA Agents"/ OR Hallucinogens/ OR "Neuroprotective Agents"/ OR "Nootropic Agents"/ OR "Serotonin Receptor Agonists"/ OR "Voltage-Gated Sodium Channel Blockers"/ OR "Antiparkinson Agents"/ OR "Dopamine Agents"/ OR "Histamine Agonists"/ OR "Histamine H2 Antagonists"/ OR "Hormone Antagonists"/ OR "Serotonin 5-HT1 Receptor Agonists"/ OR "Adrenergic alpha-2 Receptor Agonists"/ OR "Adrenergic beta-Antagonists"/ OR Sympatholytics/ OR "Excitatory Amino Acid Agonists"/ OR "Cholinesterase Inhibitors"/ OR "GABA Modulators"/ OR "GABA-B Receptor Agonists"/ OR "Ganglionic Blockers"/ OR "Nicotinic Agonists"/ OR "Nicotinic Antagonists"/ OR Parasympathomimetics/ OR "Sodium Potassium Chloride Symporter Inhibitors"/ OR "14-alpha Demethylase Inhibitors"/ OR "Adjuvants, Immunologic"/ OR "Anti-Bacterial Agents"/ OR "Antifungal Agents"/ OR "Cannabinoid Receptor Agonists"/ OR "Cannabinoid Receptor Antagonists"/ OR "Cannabinoid Receptor Modulators"/ OR "Central Nervous System Agents"/ OR "Cyclooxygenase 2 Inhibitors"/ OR "Cytochrome P-450 CYP2C9 Inhibitors"/ OR "Immunologic Factors"/ OR "Immunosuppressive Agents"/ OR "Narcotic Antagonists"/ OR "Neurotransmitter Agents"/ OR "Purinergic Agents"/ OR "Anti-Inflammatory Agents"/ OR Antioxidants/ OR "Central Nervous System Depressants"/ OR "Chelating Agents"/ OR Hormones/ OR Oxytocics/ OR "Vitamin B Complex"/ OR Vitamins/ OR "Diet Therapy".sh. OR (Brexpiprazole OR Olanzapine OR

Sertindole OR Ziprasidone OR Agomelatine OR Milnacipran OR Mirtazapine OR Tianeptine OR Tianeptine OR "1-(3-chlorophenyl)piperazine" OR Lamotrigine OR Levetiracetam OR Oxcarbazepine OR Topiramate OR "N-(4-cyanophenylmethyl)-4-(2-diphenyl)-1-piperazinehexanamide" OR Volinanserin OR "1-(quinoxalin-6-ylcarbonyl)piperidine" OR "1-butyl-3-chloro-4-(4-phenyl-1-piperidinyl)-(1H)-pyridone" OR "2-((4-tert-butylphenoxy)methyl)-5-methyl-2,3-dihydroimidazo(2,1-b)(1,3)oxazole-6-carboxamide" OR "2-amino-3-(3,4-dichlorobenzyloxy)-6-fluorobicyclo(3.1.0)hexane-2,6-dicarboxylic acid" OR "2-chloro-4-(1-(4-fluorophenyl)-2,5-dimethyl-1H-imidazol-4-ylethynyl)pyridine" OR "3-cyano-N-(1,3-diphenyl-1H-pyrazol-5-yl)benzamide" OR "4-(3-(2,6-dimethylpyridin-4-yl)phenyl)-7-methyl-8-trifluoromethyl-1,3-dihydrobenzo(b)(1,4)diazepin-2-one" OR "6-methyl-2-(phenylethynyl)pyridine" OR "AZD8529" OR Eglumetad OR Fenobam OR "GRN-529" OR "LY 341495" OR "LY 379268" OR Mavoglurant OR "MPX-004" OR "MPX-007" OR "N-(4-(2-methoxyphenoxy)phenyl)-N-(2,2,2-trifluoroethylsulfonyl)pyrid-3-ylmethylamine" OR "1-(5-chloro-2,4-dimethoxyphenyl)-3-(5-methylisoxazol-3-yl)urea" OR "3-(2,4-dimethoxybenzylidene)anabaseine" OR "4-(5-(4-chlorophenyl)-2-methyl-3-propionyl-1H-pyrrol-1-yl)benzenesulfonamide" OR "4-amino-8-(2-fluoro-6-methoxy-phenyl)-N-propylcinnoline-3-carboxamide" OR "7-chloro-N-quinuclidin-3-yl-benzo(b)thiophene-2-carboxamide" OR Acamprosate OR "ADX71441" OR "Arbaclofen Placarbil" OR Donepezil OR "SSR180711" OR "ethyl 6-(N-(2-chloro-4-fluorophenyl)sulfamoyl)cyclohex-1-ene-1-carboxylate" OR "N-(4-methoxybenzyl)-N'-(5-nitro-1,3-thiazol-2-yl)urea" OR "AF38469" OR Cannabidivarin OR Hydroxyfasudil OR Lenalidomide OR "NVP-BKM120" OR "SB 216763" OR Temsirolimus OR "NP 031112" OR "(6-chloro-1-(2-(dimethylamino)ethyl)indol-3-yl)-spiro(1H-isobenzofuran-3,4'-piperidine)-1'-yl-methanone" OR "4-(3,5-dihydroxybenzyl)-N-(2-methyl-4-((1-methyl-4,10-dihydropyrazolo(3,4-b)(1,5)benzodiazepin-5(1H)-yl)carbonyl)benzyl)piperazine-1-carboxamide" OR "alpha-tocotrienol quinone" OR "butir-His-Phe-Arg-Trp-Sar-NH2" OR Amastatin OR Dimethylglycine OR "NNZ 2566" OR "ORG 2766" OR Pioglitazone OR Sapropterin OR Sulforafan OR "Ferrous Sulfate" OR Ubiquinol).rn. OR (Anticonvuls* OR Antiepilep* OR Antipsychotic* OR Psychotropic* OR "Anti-Anxiety" OR Anxiolytic* OR Antidepress* OR "Pharmaco-Therapy" OR "Pharmaco-Therapies" OR Chemotherapy OR Chemotherapies OR Pharmacotherapy OR Pharmacotherapies OR "Pharmacological Interventions" OR "Pharmacological Intervention" OR "Pharmacological Treatment" OR "Pharmacological Treatments" OR "Drug Therapy" OR "Drug Therapies" OR Amisulpride OR Aripiprazol* OR Abilify OR Brexpiprazole OR Clozapine OR Clozaril OR Leponex OR Haloperidol OR Haldol OR Loxapine OR Lurasidone OR Latuda OR Olanzapine OR Zyprexa OR Paliperidone OR Invega OR Quetiapine OR Seroquel OR Risperidone OR Risperdal OR Risperidal OR Sertindole OR Sulpiride OR Dogmatil OR Ziprasidone OR Geodon OR Ziprazidone OR Agomelatine OR Citalopram OR Clomipramine OR Desipramine OR Escitalopram OR Fluoxetine OR Prozac OR Fluvoxamine OR Imipramine OR Mianserin OR Milnacipran OR Mirtazapine OR Nortriptyline OR Paroxetine OR Sertraline OR Tianeptine OR Tianeptine OR Venlafaxine OR "m-chlorophenylpiperazine" OR "m-CPP" OR "1-(3-chlorophenyl)piperazine" OR Atomoxetine OR Strattera OR Dextromethorphan OR Fenfluramine OR Lamotrigine OR Levetiracetam OR Lisdexamfetamine OR Lithium OR MDMA OR "N-Methyl-3,4-methylenedioxyamphetamine" OR Ecstasy OR Methylenedioxymethamphetamine OR Methylphenidate OR Ritalin* OR Oxcarbazepine OR Topiramate OR Valproic Acid OR Divalproex OR Valproate OR Divalproate OR "(+)-5-FPT" OR "PRX-07034" OR Betahistin* OR Bromocriptine OR Buspirone OR Cyproheptadine OR Famotidine OR Levodopa OR "L-Dopa" OR "LP-211" OR "N-(4-cyanophenylmethyl)-4-(2-diphenyl)-1-piperazinehexanamide" OR Sumatriptan OR Volinanserin OR "M100907" OR Clonidine OR Guanfacine OR Dexmedetomidine OR Propranolol OR Acetylcysteine OR "ADX71149" OR "JNJ-40411813" OR "1-butyl-3-chloro-4-(4-phenyl-1-piperidinyl)-(1H)-pyridone" OR Amantadine OR "AZD8529" OR Basimglurant OR "2-chloro-4-(1-(4-fluorophenyl)-2,5-dimethyl-1H-imidazol-4-ylethynyl)pyridine" OR "CDPPB" OR "3-cyano-N-

(1,3-diphenyl-1H-pyrazol-5-yl)benzamide" OR "CX516" OR "BDP 12" OR "1-(quinoxalin-6-ylcarbonyl)piperidine" OR "D-Cycloserine" OR Eglumetad OR Fenobam OR "GRN-529" OR Ketamine OR "LY 341495" OR "LY341495" OR "LY 379268" OR "LY379268" OR "LY 487379" OR "LY487379" OR Mavoglurant OR Memantine OR "MGS0039" OR "MPEP" OR "6-methyl-2-(phenylethynyl)pyridine" OR "MPX-004" OR "MPX-007" OR "MTEP" OR "NCFP" OR Riluzole OR "RO4491533" OR "TASP0433864" OR "A 867744" OR "4-(5-(4-chlorophenyl)-2-methyl-3-propionyl-1H-pyrrol-1-yl)benzenesulfonamide" OR Acamprosate OR "ADX71441" OR Arbaclofen OR "AZD7325" OR Baclofen OR Bumetanide OR "DMXB A" OR "DMXBA" OR "GTS 21" OR "3-(2,4-dimethoxybenzylidene)anabaseine" OR Donepezil OR "EVP-6124" OR "7-chloro-N-quinuclidin-3-yl-benzo(b)thiophene-2-carboxamide" OR Flumazenil OR Galantamin* OR Mecamylamine OR "PNU 120596" OR "PNU120596" OR "1-(5-chloro-2,4-dimethoxyphenyl)-3-(5-methylisoxazol-3-yl)urea" OR Pregnenolone OR Rivastigmine OR "SSR180711" OR Varenicline OR "AF38469" OR "AR-A014418" OR "N-(4-methoxybenzyl)-N'-(5-nitro-1,3-thiazol-2-yl)urea" OR Cannabidiol OR Cannabidivarin OR Celecoxib OR Everolimus OR Fingolimod OR Fluconazole OR Glatiramer OR Hydroxyfasudil OR Lenalidomide OR Minocycline OR Naltrexone OR "NVP-BKM120" OR Buparlisib OR "Oral Human Immunoglobulin" OR Pentoxifylline OR "SB 216763" OR "SB216763" OR Sirolimus OR Rapamycin OR Staurosporine OR Suramin OR Tacrolimus OR "TAK-242" OR "TAK242" OR Temsirolimus OR Tideglusib OR "NP031112" OR Amastatin OR Angiotensin* OR Carnitine OR Levocarnitine OR "CM-AT" OR "Diet Therapy" OR "Diet Therapies" OR "Dietary Supplements" OR "Dietary Supplement" OR Dimethylglycine OR "EPI-743" OR "alpha-Tocotrienol Quinone" OR "Food Supplement" OR "Food Supplements" OR "Gastrin-Releasing Peptide" OR Ghrelin OR "Herbal Supplement" OR "Herbal Supplements" OR Hormone* OR Corticosteroid* OR Corticoid* OR Hydrocortisone OR "IGF-1" OR "Insulin-Like Growth Factor I" OR Lovastatin OR Melanocortin* OR Melatonin OR Metformin OR Mineral* OR "NNZ-2566" OR "NNZ2566" OR "Nutrition Therapy" OR "Nutritional Therapy" OR Oligosaccharide* OR "Omega-3" OR "Omega3" OR "n-3 Fatty" OR "n-3 Polyunsaturated Fatty" OR "n-3 PUFA" OR "n3 PUFA" OR "n 3 Oils" OR "n 3 Oil" OR "n3 Fatty" OR "ORG-2766" OR Oxytocin OR Syntocinon OR Pioglitazone OR Prednisone OR Probiotic* OR Bifidobacter* OR Pyridoxine OR "RG7713" OR "Ro27 3225" OR Secretin OR Sulforaphane OR Sulforafan OR Tetrahydrobiopterin OR Sapropterin OR Thyroxine OR Triiodothyronine OR "T3" OR Trofinetide OR Vasopressin* OR Vitamin* OR "WAY-267464" OR "WAY267464" OR Arachidonic OR Arachidonate OR Ascorbic OR Ascorbate OR Carnosine OR Cyanocobalamin OR Cobalamin* OR Cobamide* OR Hydroxocobalamin OR Docosahexaenoic OR Docosahexaenoate OR Ferrous OR Folic OR Folate OR Ginkgo* OR Gingko* OR Ginko* OR Maidenhair OR Glutathione OR Gluten OR Inositol OR Leucovorin OR Folinic OR Magnesium OR Milk OR Papain OR Pepsin OR Pyridoxal OR Pyridoxamine OR Succimer OR Dimercaptosuccinic Acid OR DMSA OR "Trichuris Suis" OR Ubiquinol).ti,ab.

3. Randomized Controlled Trial.pt. OR Controlled Clinical Trial.pt. OR (Randomi?ed OR Placebo OR Randomly OR Trial OR Groups).ti,ab. OR Drug Therapy.fs. NOT (Exp Animals/ NOT Humans.sh.)
4. 1 AND 2 AND 3

3.2.5 PsycINFO

1. Autism Spectrum Disorders/ OR Rett Syndrome/ OR (Autis* OR Kanner* OR Asperger* OR "Pervasive Child Development Disorder" OR "Pervasive Child Development Disorders" OR "Pervasive Developmental Disorder" OR "Pervasive Developmental Disorders" OR "Pervasive Development Disorder" OR "Pervasive Development Disorders" OR "Childhood Disintegrative Disorder" OR "Childhood Disintegrative Disorders" OR Rett* OR "Cerebroatrophic Hyperammonemia" OR "Cerebroatrophic Hyperammonemias").ti,ab.
2. Exp Drugs/ OR Exp Drug Therapy/ OR Exp Adrenergic Blocking Drugs/ OR Exp Adrenergic Drugs/ OR Exp Anti Inflammatory Drugs/ OR Antiandrogens/ OR Exp Antibiotics/ OR Exp

Anticonvulsive Drugs/ OR Exp Antidepressant Drugs/ OR Exp Antiemetic Drugs/ OR Exp Antihistaminic Drugs/ OR Exp Antitremor Drugs/ OR Channel Blockers/ OR Exp Cholinergic Blocking Drugs/ OR Exp Cholinergic Drugs/ OR Exp Cholinomimetic Drugs/ OR Exp CNS Affecting Drugs/ OR Exp Dopamine Agonists/ OR Exp Enzyme Inhibitors/ OR Exp Ganglion Blocking Drugs/ OR Generic Drugs/ OR Exp Hallucinogenic Drugs/ OR Exp Narcotic Antagonists/ OR Exp Neurotransmitter Uptake Inhibitors/ OR Nonprescription Drugs/ OR Exp Nootropic Drugs/ OR Prescription Drugs/ OR Exp Psychotomimetic Drugs/ OR Exp Serotonin Agonists/ OR Exp Serotonin Antagonists/ OR Exp Sympatholytic Drugs/ OR Exp Sympathomimetic Drugs/ OR Exp Vitamins/ OR Exp Hormones/ OR Exp "Medicinal Herbs and Plants"/ OR Tricyclic Antidepressant Drugs/ OR Exp Neuroleptic Drugs/ OR Exp Dopamine Antagonists/ OR Exp Gamma Aminobutyric Acid Antagonists/ OR Exp Serotonin Reuptake Inhibitors/ OR Exp Serotonin Norepinephrine Reuptake Inhibitors/ OR Exp CNS Stimulating Drugs/ OR Exp Gamma Aminobutyric Acid Agonists/ OR Exp Cholinesterase Inhibitors/ OR Exp Immunologic Factors/ OR Exp Neurotransmitters/ OR Exp Antioxidants/ OR Exp CNS Depressant Drugs/ OR Aripiprazole/ OR Clozapine/ OR Haloperidol/ OR Loxapine/ OR Quetiapine/ OR Risperidone/ OR Sulpiride/ OR Citalopram/ OR Chlorimipramine/ OR Desipramine/ OR Fluoxetine/ OR Fluvoxamine/ OR Imipramine/ OR Mianserin/ OR Nortriptyline/ OR Paroxetine/ OR Sertraline/ OR Venlafaxine/ OR Atomoxetine/ OR Fenfluramine/ OR "Lithium Carbonate"/ OR Methylphenidate/ OR Methylenedioxymethamphetamine/ OR "Valproic Acid"/ OR Bromocriptine/ OR Buspirone/ OR Clonidine/ OR Propranolol/ OR Amantadine/ OR Ketamine/ OR Baclofen/ OR Galanthamine/ OR Mecamylamine/ OR (Immunoglobulins/ AND Oral*.ti,ab.) OR Naltrexone/ OR "Insulin-Like Growth Factor"/ OR "Adrenal Cortex Hormones"/ OR Corticotropin/ OR Angiotensin/ OR Diets/ OR "Dietary Supplements"/ OR Fatty Acids/ OR Ghrelin/ OR Hydrocortisone/ OR Melatonin/ OR Nutrition/ OR Oxytocin/ OR "Thyroid Hormones"/ OR Thyroxine/ OR Triiodothyronine/ OR Vasopressin/ OR Vitamin Therapy/ OR "Arachidonic Acid"/ OR "Ascorbic Acid"/ OR "Folic Acid"/ OR Magnesium/ OR (Anticonvuls* OR Antiepilep* OR Antipsychotic* OR Psychotropic* OR "Anti-Anxiety" OR Anxiolytic* OR Antidepress* OR "Pharmaco-Therapy" OR "Pharmaco-Therapies" OR Chemotherapy OR Chemotherapies OR Pharmacotherapy OR Pharmacotherapies OR "Pharmacological Interventions" OR "Pharmacological Intervention" OR "Pharmacological Treatment" OR "Pharmacological Treatments" OR "Drug Therapy" OR "Drug Therapies" OR Amisulpride OR Aripiprazol* OR Abilify OR Brexpiprazole OR Clozapine OR Clozaril OR Leponex OR Haloperidol OR Haldol OR Loxapine OR Lurasidone OR Latuda OR Olanzapine OR Zyprexa OR Paliperidone OR Invega OR Quetiapine OR Seroquel OR Risperidone OR Risperdal OR Risperidal OR Sertindole OR Sulpiride OR Dogmatil OR Ziprasidone OR Geodon OR Ziprazidone OR Agomelatine OR Citalopram OR Clomipramine OR Desipramine OR Escitalopram OR Fluoxetine OR Prozac OR Fluvoxamine OR Imipramine OR Mianserin OR Milnacipran OR Mirtazapine OR Nortriptyline OR Paroxetine OR Sertraline OR Tianeptine OR Tianeptine OR Venlafaxine OR "m-chlorophenylpiperazine" OR "m-CPP" OR "1-(3-chlorophenyl)piperazine" OR Atomoxetine OR Strattera OR Dextromethorphan OR Fenfluramine OR Lamotrigine OR Levetiracetam OR Lisdexamfetamine OR Lithium OR MDMA OR "N-Methyl-3,4-methylenedioxyamphetamine" OR Ecstasy OR Methylenedioxymethamphetamine OR Methylphenidate OR Ritalin* OR Oxcarbazepine OR Topiramate OR Valproic Acid OR Divalproex OR Valproate OR Divalproate OR "(+)-5-FPT" OR "PRX-07034" OR Betahistin* OR Bromocriptine OR Buspirone OR Cyproheptadine OR Famotidine OR Levodopa OR "L-Dopa" OR "LP-211" OR "N-(4-cyanophenylmethyl)-4-(2-diphenyl)-1-piperazinehexanamide" OR Sumatriptan OR Volinanserin OR "M100907" OR Clonidine OR Guanfacine OR Dexmedetomidine OR Propranolol OR Acetylcysteine OR "ADX71149" OR "JNJ-40411813" OR "1-butyl-3-chloro-4-(4-phenyl-1-piperidinyl)-(1H)-pyridone" OR Amantadine OR "AZD8529" OR Basimglurant OR "2-chloro-4-(1-(4-fluorophenyl)-2,5-dimethyl-1H-imidazol-4-ylethynyl)pyridine" OR "CDPPB" OR "3-cyano-N-(1,3-diphenyl-1H-pyrazol-5-yl)benzamide" OR "CX516" OR "BDP 12" OR "1-(quinoxalin-6-

ylcarbonyl)piperidine" OR "D-Cycloserine" OR Eglumetad OR Fenobam OR "GRN-529" OR Ketamine OR "LY 341495" OR "LY341495" OR "LY 379268" OR "LY379268" OR "LY 487379" OR "LY487379" OR Mavoglurant OR Memantine OR "MGS0039" OR "MPEP" OR "6-methyl-2-(phenylethynyl)pyridine" OR "MPX-004" OR "MPX-007" OR "MTEP" OR "NCFP" OR Riluzole OR "RO4491533" OR "TASP0433864" OR "A 867744" OR "4-(5-(4-chlorophenyl)-2-methyl-3-propionyl-1H-pyrrol-1-yl)benzenesulfonamide" OR Acamprosate OR "ADX71441" OR Arbaclofen OR "AZD7325" OR Baclofen OR Bumetanide OR "DMXB A" OR "DMXBA" OR "GTS 21" OR "3-(2,4-dimethoxybenzylidene)anabaseine" OR Donepezil OR "EVP-6124" OR "7-chloro-N-quinuclidin-3-yl-benzo(b)thiophene-2-carboxamide" OR Flumazenil OR Galantamin* OR Mecamylamine OR "PNU 120596" OR "PNU120596" OR "1-(5-chloro-2,4-dimethoxyphenyl)-3-(5-methylisoxazol-3-yl)urea" OR Pregnenolone OR Rivastigmine OR "SSR180711" OR Varenicline OR "AF38469" OR "AR-A014418" OR "N-(4-methoxybenzyl)-N'-(5-nitro-1,3-thiazol-2-yl)urea" OR Cannabidiol OR Cannabidivarin OR Celecoxib OR Everolimus OR Fingolimod OR Fluconazole OR Glatiramer OR Hydroxyfasudil OR Lenalidomide OR Minocycline OR Naltrexone OR "NVP-BKM120" OR Buparlisib OR "Oral Human Immunoglobulin" OR Pentoxifylline OR "SB 216763" OR "SB216763" OR Sirolimus OR Rapamycin OR Staurosporine OR Suramin OR Tacrolimus OR "TAK-242" OR "TAK242" OR Temsirolimus OR Tideglusib OR "NP031112" OR Amastatin OR Angiotensin* OR Carnitine OR Levocarnitine OR "CM-AT" OR "Diet Therapy" OR "Diet Therapies" OR "Dietary Supplements" OR "Dietary Supplement" OR Dimethylglycine OR "EPI-743" OR "alpha-Tocotrienol Quinone" OR "Food Supplement" OR "Food Supplements" OR "Gastrin-Releasing Peptide" OR Ghrelin OR "Herbal Supplement" OR "Herbal Supplements" OR Hormone* OR Corticosteroid* OR Corticoid* OR Hydrocortisone OR "IGF-1" OR "Insulin-Like Growth Factor I" OR Lovastatin OR Melanocortin* OR Melatonin OR Metformin OR Mineral* OR "NNZ-2566" OR "NNZ2566" OR "Nutrition Therapy" OR "Nutritional Therapy" OR Oligosaccharide* OR "Omega-3" OR "Omega3" OR "n-3 Fatty" OR "n-3 Polyunsaturated Fatty" OR "n-3 PUFA" OR "n3 PUFA" OR "n 3 Oils" OR "n 3 Oil" OR "n3 Fatty" OR "ORG-2766" OR Oxytocin OR Syntocinon OR Pioglitazone OR Prednisone OR Probiotic* OR Bifidobacter* OR Pyridoxine OR "RG7713" OR "Ro27 3225" OR Secretin OR Sulforaphane OR Sulforafan OR Tetrahydrobiopterin OR Sapropterin OR Thyroxine OR Triiodothyronine OR "T3" OR Trofinetide OR Vasopressin* OR Vitamin* OR "WAY-267464" OR "WAY267464" OR Arachidonic OR Arachidonate OR Ascorbic OR Ascorbate OR Carnosine OR Cyanocobalamin OR Cobalamin* OR Cobamide* OR Hydroxocobalamin OR Docosahexaenoic OR Docosahexaenoate OR Ferrous OR Folic OR Folate OR Ginkgo* OR Gingko* OR Ginko* OR Maidenhair OR Glutathione OR Gluten OR Inositol OR Leucovorin OR Folinic OR Magnesium OR Milk OR Papain OR Pepsin OR Pyridoxal OR Pyridoxamine OR Succimer OR Dimercaptosuccinic Acid OR DMSA OR "Trichuris Suis" OR Ubiquinol).ti,ab.

3. Exp Treatment Effectiveness Evaluation/ OR Clinical Trials/ OR Mental Health Program Evaluation/ OR Placebo/ OR (Random* OR Factorial* OR Crossover* OR Cross Over* OR Placebo* OR ((Singl* OR Doubl* OR Trebl* or Tripl*) adj (Mask* OR Blind*)) OR Assign* OR Allocat* OR Volunteer* OR Groups OR Trial*).ti,ab.
4. 1 AND 2 AND 3

3.2.6 PubMed

("Autistic Disorder"[MH] OR "Autism Spectrum Disorder"[MH] OR "Asperger Syndrome"[MH] OR "Rett Syndrome"[MH] OR "Child Development Disorders, Pervasive"[MH] OR Autis*[TIAB] OR Kanner*[TIAB] OR Asperger*[TIAB] OR "Pervasive Child Development Disorders"[TIAB] OR "Pervasive Developmental Disorder"[TIAB] OR "Pervasive Developmental Disorders"[TIAB] OR "Pervasive Development Disorder"[TIAB] OR "Pervasive Development Disorders"[TIAB] OR "Childhood Disintegrative Disorder"[TIAB] OR Rett*[TIAB]) AND ("Adrenergic alpha-2 Receptor Antagonists"[PA] OR "Antidepressive Agents, Second-Generation"[PA] OR "Anti-Dyskinesia Agents"[PA] OR Antiemetics[PA] OR "Antipsychotic Agents"[PA] OR "Dopamine Agonists"[PA]

OR "Dopamine Antagonists"[PA] OR "Dopamine D2 Receptor Antagonists"[PA] OR "GABA Antagonists"[PA] OR "Serotonin 5-HT2 Receptor Antagonists"[PA] OR "Serotonin Agents"[PA] OR "Serotonin Antagonists"[PA] OR "Serotonin Uptake Inhibitors"[PA] OR "Adrenergic alpha-Antagonists"[PA] OR "Adrenergic Uptake Inhibitors"[PA] OR "Cytochrome P-450 CYP1A2 Inhibitors"[PA] OR "Cytochrome P-450 CYP2C19 Inhibitors"[PA] OR "Cytochrome P-450 CYP2D6 Inhibitors"[PA] OR "Enzyme Inhibitors"[PA] OR "Histamine H1 Antagonists"[PA] OR "Serotonin and Noradrenaline Reuptake Inhibitors"[PA] OR "Anti-Anxiety Agents"[PA] OR "Antidepressive Agents"[PA] OR "Antidepressive Agents, Tricyclic"[PA] OR "Psychotropic Drugs"[PA] OR Anticonvulsants[PA] OR "Antimanic Agents"[PA] OR "Calcium Channel Blockers"[PA] OR "Central Nervous System Stimulants"[PA] OR "Cytochrome P-450 CYP3A Inducers"[PA] OR "Dopamine Uptake Inhibitors"[PA] OR "Excitatory Amino Acid Antagonists"[PA] OR "GABA Agents"[PA] OR Hallucinogens[PA] OR "Neuroprotective Agents"[PA] OR "Nootropic Agents"[PA] OR "Serotonin Receptor Agonists"[PA] OR "Voltage-Gated Sodium Channel Blockers"[PA] OR "Antiparkinson Agents"[PA] OR "Dopamine Agents"[PA] OR "Histamine Agonists"[PA] OR "Histamine H2 Antagonists"[PA] OR "Hormone Antagonists"[PA] OR "Serotonin 5-HT1 Receptor Agonists"[PA] OR "Adrenergic alpha-2 Receptor Agonists"[PA] OR "Adrenergic beta-Antagonists"[PA] OR Sympatholytics[PA] OR "Excitatory Amino Acid Agonists"[PA] OR "Cholinesterase Inhibitors"[PA] OR "GABA Modulators"[PA] OR "GABA-B Receptor Agonists"[PA] OR "Ganglionic Blockers"[PA] OR "Nicotinic Agonists"[PA] OR "Nicotinic Antagonists"[PA] OR Parasympathomimetics[PA] OR "Sodium Potassium Chloride Symporter Inhibitors"[PA] OR "14-alpha Demethylase Inhibitors"[PA] OR "Adjuvants, Immunologic"[PA] OR "Anti-Bacterial Agents"[PA] OR "Antifungal Agents"[PA] OR "Cannabinoid Receptor Agonists"[PA] OR "Cannabinoid Receptor Antagonists"[PA] OR "Cannabinoid Receptor Modulators"[PA] OR "Central Nervous System Agents"[PA] OR "Cyclooxygenase 2 Inhibitors"[PA] OR "Cytochrome P-450 CYP2C9 Inhibitors"[PA] OR "Immunologic Factors"[PA] OR "Immunosuppressive Agents"[PA] OR "Narcotic Antagonists"[PA] OR "Neurotransmitter Agents"[PA] OR "Purinergic Agents"[PA] OR "Anti-Inflammatory Agents"[PA] OR Antioxidants[PA] OR "Central Nervous System Depressants"[PA] OR "Chelating Agents"[PA] OR Hormones[PA] OR Oxytocics[PA] OR "Vitamin B Complex"[PA] OR Vitamins[PA] OR "Pharmaceutical Preparations"[MH:NoExp] OR "Drug Combinations"[MH] OR "Drugs, Chinese Herbal"[MH] OR "Drugs, Essential"[MH] OR "Drugs, Generic"[MH] OR "Drugs, Investigational"[MH] OR "Nonprescription Drugs"[MH] or "Plant Extracts"[MH] OR "Prescription Drugs"[MH] OR Prodrugs[MH] OR "Pharmacologic Actions"[MH] OR "Drug Therapy"[MH] OR "Therapeutic Uses"[MH] OR "Physiological Effects of Drugs"[MH] OR Aripiprazole[MH] OR Clozapine[MH] OR Haloperidol[MH] OR Loxapine[MH] OR "Lurasidone Hydrochloride"[MH] OR "Paliperidone Palmitate"[MH] OR "Quetiapine Fumarate"[MH] OR Risperidone[MH] OR Sulpiride[MH] OR Citalopram[MH] OR Clomipramine[MH] OR Desipramine[MH] OR Fluoxetine[MH] OR Fluvoxamine[MH] OR Imipramine[MH] OR Mianserin[MH] OR Nortriptyline[MH] OR Paroxetine[MH] OR Sertraline[MH] OR "Venlafaxine Hydrochloride"[MH] OR "Atomoxetine Hydrochloride"[MH] OR Dextromethorphan[MH] OR Fenfluramine[MH] OR "Lisdexamfetamine Dimesylate"[MH] OR "Lithium Carbonate"[MH] OR Methylphenidate[MH] OR "N-Methyl-3,4-methylenedioxyamphetamine"[MH] OR "Valproic Acid"[MH] OR Betahistine[MH] OR Bromocriptine[MH] OR Buspirone[MH] OR Cyproheptadine[MH] OR Famotidine[MH] OR Levodopa[MH] OR Sumatriptan[MH] OR Clonidine[MH] OR Guanfacine[MH] OR Dexmedetomidine[MH] OR Propranolol[MH] OR Acetylcysteine[MH] OR Amantadine[MH] OR Ketamine[MH] OR Memantine[MH] OR Riluzole[MH] OR Baclofen[MH] OR Bumetanide[MH] OR Flumazenil[MH] OR Galantamine[MH] OR Mecamylamine[MH] OR Pregnenolone[MH] OR Rivastigmine[MH] OR Varenicline[MH] OR Cannabidiol[MH] OR Celecoxib[MH] OR Everolimus[MH] OR "Fingolimod Hydrochloride"[MH] OR Fluconazole[MH] OR "Glatiramer Acetate"[MH] OR (Immunoglobulins[MH] AND "Administration, Oral"[MH]) OR Minocycline[MH] OR Naltrexone[MH] OR Pentoxifylline[MH] OR Sirolimus[MH] OR Staurosporine[MH] OR Suramin[MH] OR Tacrolimus[MH] OR "Insulin-Like Growth Factor I"[MH] OR"Adrenal Cortex Hormones"[MH] OR "Adrenocorticotropic Hormone"[MH] OR Angiotensins[MH] OR Carnitine[MH]

OR "Diet Therapy"[MH] OR "Dietary Supplements"[MH] OR "Fatty Acids, Omega-3"[MH] OR "Gastrin-Releasing Peptide"[MH] OR Ghrelin[MH] OR Hydrocortisone[MH] OR Lovastatin[MH] OR Melanocortins[MH] OR Melatonin[MH] OR Metformin[MH] OR Minerals[MH] OR "Nutrition Therapy"[MH] OR Oligosaccharides[MH] OR Oxytocin[MH] OR Prednisone[MH] OR Probiotics[MH] OR Pyridoxine[MH] OR Secretin[MH] OR "Thyroid Hormones"[MH] OR Thyroxine[MH] OR Triiodothyronine[MH] OR Vasopressins[MH] OR "Vitamin E"[MH] OR "Arachidonic Acid"[MH] OR "Ascorbic Acid"[MH] OR Carnosine[MH] OR "Docosahexaenoic Acids"[MH] OR "Folic Acid"[MH] OR "Ginkgo biloba"[MH] OR Glutathione[MH] OR Glutens[MH] OR Inositol[MH] OR Leucovorin[MH] OR Magnesium[MH] OR "Magnesium Oxide"[MH] OR Milk[MH] OR Papain[MH] OR Succimer[MH] OR "Vitamin B 12"[MH] OR "Vitamin B 6"[MH] OR "Vitamin D"[MH] OR "Adrenergic alpha-2 Receptor Antagonists"[MH] OR "Antidepressive Agents, Second-Generation"[MH] OR "Anti-Dyskinesia Agents"[MH] OR Antiemetics[MH] OR "Antipsychotic Agents"[MH] OR "Dopamine Agonists"[MH] OR "Dopamine Antagonists"[MH] OR "Dopamine D2 Receptor Antagonists"[MH] OR "GABA Antagonists"[MH] OR "Serotonin 5-HT2 Receptor Antagonists"[MH] OR "Serotonin Agents"[MH] OR "Serotonin Antagonists"[MH] OR "Serotonin Uptake Inhibitors"[MH] OR "Adrenergic alpha-Antagonists"[MH] OR "Adrenergic Uptake Inhibitors"[MH] OR "Cytochrome P-450 CYP1A2 Inhibitors"[MH] OR "Cytochrome P-450 CYP2C19 Inhibitors"[MH] OR "Cytochrome P-450 CYP2D6 Inhibitors"[MH] OR "Enzyme Inhibitors"[MH] OR "Histamine H1 Antagonists"[MH] OR "Serotonin and Noradrenaline Reuptake Inhibitors"[MH] OR "Anti-Anxiety Agents"[MH] OR "Antidepressive Agents"[MH] OR "Antidepressive Agents, Tricyclic"[MH] OR "Psychotropic Drugs"[MH] OR Anticonvulsants[MH] OR "Antimanic Agents"[MH] OR "Calcium Channel Blockers"[MH] OR "Central Nervous System Stimulants"[MH] OR "Cytochrome P-450 CYP3A Inducers"[MH] OR "Dopamine Uptake Inhibitors"[MH] OR "Excitatory Amino Acid Antagonists"[MH] OR "GABA Agents"[MH] OR Hallucinogens[MH] OR "Neuroprotective Agents"[MH] OR "Nootropic Agents"[MH] OR "Serotonin Receptor Agonists"[MH] OR "Voltage-Gated Sodium Channel Blockers"[MH] OR "Antiparkinson Agents"[MH] OR "Dopamine Agents"[MH] OR "Histamine Agonists"[MH] OR "Histamine H2 Antagonists"[MH] OR "Hormone Antagonists"[MH] OR "Serotonin 5-HT1 Receptor Agonists"[MH] OR "Adrenergic alpha-2 Receptor Agonists"[MH] OR "Adrenergic beta-Antagonists"[MH] OR Sympatholytics[MH] OR "Excitatory Amino Acid Agonists"[MH] OR "Cholinesterase Inhibitors"[MH] OR "GABA Modulators"[MH] OR "GABA-B Receptor Agonists"[MH] OR "Ganglionic Blockers"[MH] OR "Nicotinic Agonists"[MH] OR "Nicotinic Antagonists"[MH] OR Parasympathomimetics[MH] OR "Sodium Potassium Chloride Symporter Inhibitors"[MH] OR "14-alpha Demethylase Inhibitors"[MH] OR "Adjuvants, Immunologic"[MH] OR "Anti-Bacterial Agents"[MH] OR "Antifungal Agents"[MH] OR "Cannabinoid Receptor Agonists"[MH] OR "Cannabinoid Receptor Antagonists"[MH] OR "Cannabinoid Receptor Modulators"[MH] OR "Central Nervous System Agents"[MH] OR "Cyclooxygenase 2 Inhibitors"[MH] OR "Cytochrome P-450 CYP2C9 Inhibitors"[MH] OR "Immunologic Factors"[MH] OR "Immunosuppressive Agents"[MH] OR "Narcotic Antagonists"[MH] OR "Neurotransmitter Agents"[MH] OR "Purinergic Agents"[MH] OR "Anti-Inflammatory Agents"[MH] OR Antioxidants[MH] OR "Central Nervous System Depressants"[MH] OR "Chelating Agents"[MH] OR Hormones[MH] OR Oxytocics[MH] OR "Vitamin B Complex"[MH] OR Vitamins[MH] OR "Diet Therapy"[sh] OR Brexpiprazole[NM] OR Olanzapine[NM] OR Sertindole[NM] OR Ziprasidone[NM] OR Agomelatine[NM] OR Milnacipran[NM] OR Mirtazapine[NM] OR Tianeptine[NM] OR Tianeptine[NM] OR "1-(3-chlorophenyl)piperazine"[NM] OR Lamotrigine[NM] OR Levetiracetam[NM] OR Oxcarbazepine[NM] OR Topiramate[NM] OR "N-(4-cyanophenylmethyl)-4-(2-diphenyl)-1-piperazinehexanamide"[NM] OR Volinanserin[NM] OR "1-(quinoxalin-6-ylcarbonyl)piperidine"[NM] OR "1-butyl-3-chloro-4-(4-phenyl-1-piperidinyl)-(1H)-pyridone"[NM] OR "2-((4-tert-butylphenoxy)methyl)-5-methyl-2,3-dihydroimidazo(2,1-b)(1,3)oxazole-6-carboxamide"[NM] OR "2-amino-3-(3,4-dichlorobenzyloxy)-6-fluorobicyclo(3.1.0)hexane-2,6-dicarboxylic acid"[NM] OR "2-chloro-4-(1-(4-fluorophenyl)-2,5-dimethyl-1H-imidazol-4-ylethynyl)pyridine"[NM] OR "3-cyano-N-(1,3-diphenyl-1H-pyrazol-5-yl)benzamide"[NM] OR "4-(3-(2,6-dimethylpyridin-4-yl)phenyl)-7-

methyl-8-trifluoromethyl-1,3-dihydrobenzo(b)(1,4)diazepin-2-one"[NM] OR "6-methyl-2-(phenylethynyl)pyridine"[NM] OR "AZD8529"[NM] OR Eglumetad[NM] OR Fenobam[NM] OR "GRN-529"[NM] OR "LY 341495"[NM] OR "LY 379268"[NM] OR Mavoglurant[NM] OR "MPX-004"[NM] OR "MPX-007"[NM] OR "N-(4-(2-methoxyphenoxy)phenyl)-N-(2,2,2-trifluoroethylsulfonyl)pyrid-3-ylmethylamine"[NM] OR "1-(5-chloro-2,4-dimethoxyphenyl)-3-(5-methylisoxazol-3-yl)urea"[NM] OR "3-(2,4-dimethoxybenzylidene)anabaseine"[NM] OR "4-(5-(4-chlorophenyl)-2-methyl-3-propionyl-1H-pyrrol-1-yl)benzenesulfonamide"[NM] OR "4-amino-8-(2-fluoro-6-methoxy-phenyl)-N-propylcinnoline-3-carboxamide"[NM] OR "7-chloro-N-quinuclidin-3-yl-benzo(b)thiophene-2-carboxamide"[NM] OR Acamprosate[NM] OR "ADX71441"[NM] OR "Arbaclofen Placarbil"[NM] OR Donepezil[NM] OR "SSR180711"[NM] OR "ethyl 6-(N-(2-chloro-4-fluorophenyl)sulfamoyl)cyclohex-1-ene-1-carboxylate"[NM] OR "N-(4-methoxybenzyl)-N'-(5-nitro-1,3-thiazol-2-yl)urea"[NM] OR "AF38469"[NM] OR Cannabidivarin[NM] OR Hydroxyfasudil[NM] OR Lenalidomide[NM] OR "NVP-BKM120"[NM] OR "SB 216763"[NM] OR Temsirolimus[NM] OR "NP 031112"[NM] OR "(6-chloro-1-(2-(dimethylamino)ethyl)indol-3-yl)-spiro(1H-isobenzofuran-3,4'-piperidine)-1'-yl-methanone"[NM] OR "4-(3,5-dihydroxybenzyl)-N-(2-methyl-4-((1-methyl-4,10-dihydropyrazolo(3,4-b)(1,5)benzodiazepin-5(1H)-yl)carbonyl)benzyl)piperazine-1-carboxamide"[NM] OR "alpha-tocotrienol quinone"[NM] OR "butir-His-Phe-Arg-Trp-Sar-NH2"[NM] OR Amastatin[NM] OR Dimethylglycine[NM] OR "NNZ 2566"[NM] OR "ORG 2766"[NM] OR Pioglitazone[NM] OR Sapropterin[NM] OR Sulforafan[NM] OR "Ferrous Sulfate"[NM] OR Ubiquinol[NM] OR Anticonvuls*[TIAB] OR Antiepilep*[TIAB] OR Antipsychotic*[TIAB] OR Psychotropic*[TIAB] OR "Anti-Anxiety"[TIAB] OR Anxiolytic*[TIAB] OR Antidepress*[TIAB] OR "Pharmaco-Therapy"[TIAB] OR "Pharmaco-Therapies"[TIAB] OR Chemotherapy[TIAB] OR Chemotherapies[TIAB] OR Pharmacotherapy[TIAB] OR Pharmacotherapies[TIAB] OR "Pharmacological Interventions"[TIAB] OR "Pharmacological Intervention"[TIAB] OR "Pharmacological Treatment"[TIAB] OR "Pharmacological Treatments"[TIAB] OR "Drug Therapy"[TIAB] OR "Drug Therapies"[TIAB] OR Amisulpride[TIAB] OR Aripiprazol*[TIAB] OR Abilify[TIAB] OR Brexpiprazole[TIAB] OR Clozapine[TIAB] OR Clozaril[TIAB] OR Leponex[TIAB] OR Haloperidol[TIAB] OR Haldol[TIAB] OR Loxapine[TIAB] OR Lurasidone[TIAB] OR Latuda[TIAB] OR Olanzapine[TIAB] OR Zyprexa[TIAB] OR Paliperidone[TIAB] OR Invega[TIAB] OR Quetiapine[TIAB] OR Seroquel[TIAB] OR Risperidone[TIAB] OR Risperdal[TIAB] OR Risperidal[TIAB] OR Sertindole[TIAB] OR Sulpiride[TIAB] OR Dogmatil[TIAB] OR Ziprasidone[TIAB] OR Geodon[TIAB] OR Ziprazidone[TIAB] OR Agomelatine[TIAB] OR Citalopram[TIAB] OR Clomipramine[TIAB] OR Desipramine[TIAB] OR Escitalopram[TIAB] OR Fluoxetine[TIAB] OR Prozac[TIAB] OR Fluvoxamine[TIAB] OR Imipramine[TIAB] OR Mianserin[TIAB] OR Milnacipran[TIAB] OR Mirtazapine[TIAB] OR Nortriptyline[TIAB] OR Paroxetine[TIAB] OR Sertraline[TIAB] OR Tianeptine[TIAB] OR Tianeptine[TIAB] OR Venlafaxine[TIAB] OR "m-chlorophenylpiperazine"[TIAB] OR "m-CPP"[TIAB] OR "1-(3-chlorophenyl)piperazine"[TIAB] OR Atomoxetine[TIAB] OR Strattera[TIAB] OR Dextromethorphan[TIAB] OR Fenfluramine[TIAB] OR Lamotrigine[TIAB] OR Levetiracetam[TIAB] OR Lisdexamfetamine[TIAB] OR Lithium[TIAB] OR MDMA[TIAB] OR "N-Methyl-3,4-methylenedioxyamphetamine"[TIAB] OR Ecstasy[TIAB] OR Methylenedioxymethamphetamine[TIAB] OR Methylphenidate[TIAB] OR Ritalin*[TIAB] OR Oxcarbazepine[TIAB] OR Topiramate[TIAB] OR Valproic Acid[TIAB] OR Divalproex[TIAB] OR Valproate[TIAB] OR Divalproate[TIAB] OR "(+)-5-FPT"[TIAB] OR "PRX-07034"[TIAB] OR Betahistin*[TIAB] OR Bromocriptine[TIAB] OR Buspirone[TIAB] OR Cyproheptadine[TIAB] OR Famotidine[TIAB] OR Levodopa[TIAB] OR "L-Dopa"[TIAB] OR "LP-211"[TIAB] OR "N-(4-cyanophenylmethyl)-4-(2-diphenyl)-1-piperazinehexanamide"[TIAB] OR Sumatriptan[TIAB] OR Volinanserin[TIAB] OR "M100907"[TIAB] OR Clonidine[TIAB] OR Guanfacine[TIAB] OR Dexmedetomidine[TIAB] OR Propranolol[TIAB] OR Acetylcysteine[TIAB] OR "ADX71149"[TIAB] OR "JNJ-40411813"[TIAB] OR "1-butyl-3-chloro-4-(4-phenyl-1-piperidinyl)-(1H)-pyridone"[TIAB] OR Amantadine[TIAB] OR "AZD8529"[TIAB] OR Basimglurant[TIAB] OR "2-chloro-4-(1-(4-fluorophenyl)-2,5-dimethyl-1H-imidazol-4-ylethynyl)pyridine"[TIAB] OR "CDPPB"[TIAB] OR "3-

cyano-N-(1,3-diphenyl-1H-pyrazol-5-yl)benzamide"[TIAB] OR "CX516"[TIAB] OR "BDP 12"[TIAB] OR "1-(quinoxalin-6-ylcarbonyl)piperidine"[TIAB] OR "D-Cycloserine"[TIAB] OR Eglumetad[TIAB] OR Fenobam[TIAB] OR "GRN-529"[TIAB] OR Ketamine[TIAB] OR "LY 341495"[TIAB] OR "LY341495"[TIAB] OR "LY 379268"[TIAB] OR "LY379268"[TIAB] OR "LY 487379"[TIAB] OR "LY487379"[TIAB] OR Mavoglurant[TIAB] OR Memantine[TIAB] OR "MGS0039"[TIAB] OR "MPEP"[TIAB] OR "6-methyl-2-(phenylethynyl)pyridine"[TIAB] OR "MPX-004"[TIAB] OR "MPX-007"[TIAB] OR "MTEP"[TIAB] OR "NCFP"[TIAB] OR Riluzole[TIAB] OR "RO4491533"[TIAB] OR "TASP0433864"[TIAB] OR "A 867744"[TIAB] OR "4-(5-(4-chlorophenyl)-2-methyl-3-propionyl-1H-pyrrol-1-yl)benzenesulfonamide"[TIAB] OR Acamprosate[TIAB] OR "ADX71441"[TIAB] OR Arbaclofen[TIAB] OR "AZD7325"[TIAB] OR Baclofen[TIAB] OR Bumetanide[TIAB] OR "DMXB A"[TIAB] OR "DMXBA"[TIAB] OR "GTS 21"[TIAB] OR "3-(2,4-dimethoxybenzylidene)anabaseine"[TIAB] OR Donepezil[TIAB] OR "EVP-6124"[TIAB] OR "7-chloro-N-quinuclidin-3-yl-benzo(b)thiophene-2-carboxamide"[TIAB] OR Flumazenil[TIAB] OR Galantamin*[TIAB] OR Mecamylamine[TIAB] OR "PNU 120596"[TIAB] OR "PNU120596"[TIAB] OR "1-(5-chloro-2,4-dimethoxyphenyl)-3-(5-methylisoxazol-3-yl)urea"[TIAB] OR Pregnenolone[TIAB] OR Rivastigmine[TIAB] OR "SSR180711"[TIAB] OR Varenicline[TIAB] OR "AF38469"[TIAB] OR "AR-A014418"[TIAB] OR "N-(4-methoxybenzyl)-N'-(5-nitro-1,3-thiazol-2-yl)urea"[TIAB] OR Cannabidiol[TIAB] OR Cannabidivarin[TIAB] OR Celecoxib[TIAB] OR Everolimus[TIAB] OR Fingolimod[TIAB] OR Fluconazole[TIAB] OR Glatiramer[TIAB] OR Hydroxyfasudil[TIAB] OR Lenalidomide[TIAB] OR Minocycline[TIAB] OR Naltrexone[TIAB] OR "NVP-BKM120"[TIAB] OR Buparlisib[TIAB] OR "Oral Human Immunoglobulin"[TIAB] OR Pentoxifylline[TIAB] OR "SB 216763"[TIAB] OR "SB216763"[TIAB] OR Sirolimus[TIAB] OR Rapamycin[TIAB] OR Staurosporine[TIAB] OR Suramin[TIAB] OR Tacrolimus[TIAB] OR "TAK-242"[TIAB] OR "TAK242"[TIAB] OR Temsirolimus[TIAB] OR Tideglusib[TIAB] OR "NP031112"[TIAB] OR Amastatin[TIAB] OR Angiotensin*[TIAB] OR Carnitine[TIAB] OR Levocarnitine[TIAB] OR "CM-AT"[TIAB] OR "Diet Therapy"[TIAB] OR "Diet Therapies"[TIAB] OR "Dietary Supplements"[TIAB] OR "Dietary Supplement"[TIAB] OR Dimethylglycine[TIAB] OR "EPI-743"[TIAB] OR "alpha-Tocotrienol Quinone"[TIAB] OR "Food Supplement"[TIAB] OR "Food Supplements"[TIAB] OR "Gastrin-Releasing Peptide"[TIAB] OR Ghrelin[TIAB] OR "Herbal Supplement"[TIAB] OR "Herbal Supplements"[TIAB] OR Hormone*[TIAB] OR Corticosteroid*[TIAB] OR Corticoid*[TIAB] OR Hydrocortisone[TIAB] OR "IGF-1"[TIAB] OR "Insulin-Like Growth Factor I"[TIAB] OR Lovastatin[TIAB] OR Melanocortin*[TIAB] OR Melatonin[TIAB] OR Metformin[TIAB] OR Mineral*[TIAB] OR "NNZ-2566"[TIAB] OR "NNZ2566"[TIAB] OR "Nutrition Therapy"[TIAB] OR "Nutritional Therapy"[TIAB] OR Oligosaccharide*[TIAB] OR "Omega-3"[TIAB] OR "Omega3"[TIAB] OR "n-3 Fatty"[TIAB] OR "n-3 Polyunsaturated Fatty"[TIAB] OR "n-3 PUFA"[TIAB] OR "n3 PUFA"[TIAB] OR "n 3 Oils"[TIAB] OR "n 3 Oil"[TIAB] OR "n3 Fatty"[TIAB] OR "ORG-2766"[TIAB] OR Oxytocin[TIAB] OR Syntocinon[TIAB] OR Pioglitazone[TIAB] OR Prednisone[TIAB] OR Probiotic*[TIAB] OR Bifidobacter*[TIAB] OR Pyridoxine[TIAB] OR "RG7713"[TIAB] OR "Ro27 3225"[TIAB] OR Secretin[TIAB] OR Sulforaphane[TIAB] OR Sulforafan[TIAB] OR Tetrahydrobiopterin[TIAB] OR Sapropterin[TIAB] OR Thyroxine[TIAB] OR Triiodothyronine[TIAB] OR "T3"[TIAB] OR Trofinetide[TIAB] OR Vasopressin*[TIAB] OR Vitamin*[TIAB] OR "WAY-267464"[TIAB] OR "WAY267464"[TIAB] OR Arachidonic[TIAB] OR Arachidonate[TIAB] OR Ascorbic[TIAB] OR Ascorbate[TIAB] OR Carnosine[TIAB] OR Cyanocobalamin[TIAB] OR Cobalamin*[TIAB] OR Cobamide*[TIAB] OR Hydroxocobalamin[TIAB] OR Docosahexaenoic[TIAB] OR Docosahexaenoate[TIAB] OR Ferrous[TIAB] OR Folic[TIAB] OR Folate[TIAB] OR Ginkgo*[TIAB] OR Gingko*[TIAB] OR Ginko*[TIAB] OR Maidenhair[TIAB] OR Glutathione[TIAB] OR Gluten[TIAB] OR Inositol[TIAB] OR Leucovorin[TIAB] OR Folinic[TIAB] OR Magnesium[TIAB] OR Milk[TIAB] OR Papain[TIAB] OR Pepsin[TIAB] OR Pyridoxal[TIAB] OR Pyridoxamine[TIAB] OR Succimer[TIAB] OR Dimercaptosuccinic Acid[TIAB] OR DMSA[TIAB] OR "Trichuris Suis"[TIAB] OR Ubiquinol[TIAB]) AND ("Randomized Controlled Trial"[PT] OR "Controlled Clinical Trial"[PT]

OR Randomized[TIAB] OR Randomised[TIAB] OR Placebo*[TIAB] OR "Drug Therapy"[SH] OR Randomly[TIAB] OR Trial[TIAB] OR Groups[TIAB]) NOT (Animals[MH] NOT Humans[MH])

3.2.7 World Health Organization International Clinical Trials Registry Platform (WHO ICTRP)

Including:

- Australian New Zealand Clinical Trials Registry, last data file imported on 2 July 2018
- Chinese Clinical Trial Registry, last data file imported on 2 July 2018
- ClinicalTrials.gov, last data file imported on 2 July 2018
- EU Clinical Trials Register (EU-CTR), last data file imported on 25 June 2018
- ISRCTN, last data file imported on 2 July 2018
- The Netherlands National Trial Register, last data file imported on 2 July 2018
- Brazilian Clinical Trials Registry (ReBec), last data file imported on 20 June 2018
- Clinical Trials Registry - India, last data file imported on 18 June 2018
- Clinical Research Information Service - Republic of Korea, last data file imported on 18 June 2018
- Cuban Public Registry of Clinical Trials, last data file imported on 18 June 2018
- German Clinical Trials Register, last data file imported on 18 June 2018
- Iranian Registry of Clinical Trials, last data file imported on 20 June 2018
- Japan Primary Registries Network, last data file imported on 20 June 2018
- Pan African Clinical Trial Registry, last data file imported on 22 May 2018
- Sri Lanka Clinical Trials Registry, last data file imported on 18 June 2018
- Thai Clinical Trials Registry (TCTR), last data file imported on 20 June 2018
- Peruvian Clinical Trials Registry (REPEC), last data file imported on 18 June 2018

<u>Advanced Search</u>

Autism OR Autistic OR Asperger OR Rett OR Pervasive OR Disintegrative OR Hyperammonemia OR Hyperammonaemia <u>in the Condition</u>

<u>Recruitment status is ALL</u>

3.3 References

1. Lefebvre C, Manheimer E, Glanville J. Chapter 6: Searching for studies. In: Higgins JPT, Green S, editors. Cochrane Handbook for Systematic Reviews of Interventions. Version 5.1.0 [updated March 2011] ed.: The Cochrane Collaboration; 2011.
2. Higgins JPT, Lasserson T, Chandler J, Tovey D, Churchill R. Methodological Expectations of Cochrane Intervention Reviews. London: Cochrane; 2016.
3. Moher D, Liberati A, Tetzlaff J, Altman DG. Preferred reporting items for systematic reviews and meta-analyses: the PRISMA statement. PLoS Medicine. 2009;6(7):e1000097. doi: 10.1371/journal.pmed.1000097.
4. McGowan J, Sampson M, Salzwedel DM, Cogo E, Foerster V, Lefebvre C. PRESS peer review of electronic search strategies: 2015 guideline statement. Journal of Clinical Epidemiology. 2016;75:40-6.
5. Tromans S, Adams C. Brief Report: Autism Spectrum Disorder: A Comprehensive Survey of Randomized Controlled Trials. Journal of Autism and Developmental Disorders. 2018. doi: 10.1007/s10803-018-3569-y.
6. Grossetta Nardini HK, Wang L: The Yale MeSH Analyzer [Internet]. http://mesh.med.yale.edu/ (2018). Accessed Date cited: 7th July 2018.
7. Glanville J, Kaunelis D, Mensinkai S, Picheca L: Pruning Emtree: does focusing Embase subject headings impact search strategy precision and sensitivity? [Internet]. https://www.cadth.ca/pruning-emtree-embase (2015). Accessed Date cited: 7th July 2018.
8. Eisinga A: Embase animal filter. https://bit.ly/2IRTda1 (2013). Accessed.
9. Agarwal A, Johnston BC, Vernooij RW, Carrasco-Labra A, Brignardello-Petersen R, Neumann I, et al. Authors seldom report the most patient-important outcomes and absolute effect measures in systematic review abstracts. Journal of Clinical Epidemiology. 2017;81:3-12. doi: 10.1016/j.jclinepi.2016.08.004.
10. Chalmers I, Glasziou P. Avoidable waste in the production and reporting of research evidence. Lancet. 2009;374(9683):86-9. doi: 10.1016/s0140-6736(09)60329-9.
11. Chan AW, Hrobjartsson A, Haahr MT, Gotzsche PC, Altman DG. Empirical evidence for selective reporting of outcomes in randomized trials: comparison of protocols to published articles. JAMA. 2004;291(20):2457-65. doi: 10.1001/jama.291.20.2457.
12. Glasziou P, Altman DG, Bossuyt P, Boutron I, Clarke M, Julious S, et al. Reducing waste from incomplete or unusable reports of biomedical research. Lancet. 2014;383(9913):267-76. doi: 10.1016/s0140-6736(13)62228-x.
13. Mantziari S, Demartines N. Poor outcome reporting in medical research; building practice on spoilt grounds. Annals of Translational Medicine. 2017;5(Suppl 1):S15. doi: 10.21037/atm.2017.03.75.

eAppendix-4 Study selection

4.1 PRISMA flow diagram of study selection 50
4.2 Excluded records 51
4.2.1 Excluded records 51
4.2.2 References of excluded records 51
4.3 Eligible trials, included and ongoing 110
4.3.1 Included records in the systematic review 110
4.3.2 Ongoing trials (as of 06.10.2021) 144
4.4 Contacting corresponding authors for additional data/clarifications 150

4.1 PRISMA flow diagram of study selection

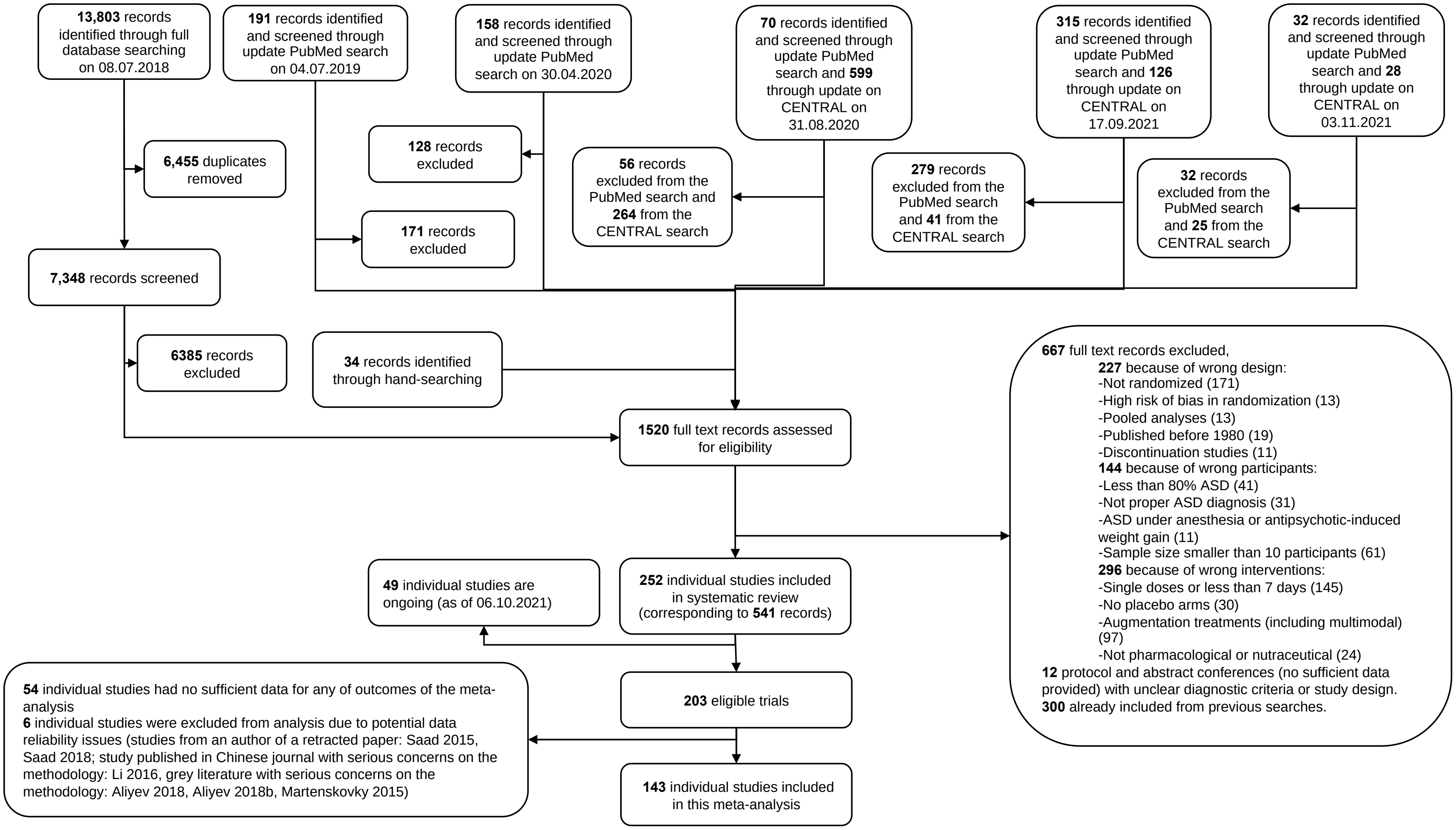

4.2 Excluded records

4.2.1 Excluded records

1) Excluded records (*k= 667*)
   a) Wrong design (k=227):
      i) not randomized (k=171):[1–159, 866-873, 880-882, 950]
      ii) high risk of bias in randomization (sequence generation or allocation concealment, k=13):[160–170, 877-878]
      iii) pooled analyses (k=13):[171–181, 837-838, 951]
      iv) placebo-discontinuation studies (k=11):[182–189, 836, 834, 981]
      v) published before 1980 (k=19):[190–208]
   b) Wrong participants (k=144):
      i) Less than 80% had ASD (k=41):[209–245, 954-957]
      ii) Inappropriate ASD diagnosis (standardized diagnostic criteria or validated diagnostic tools were not used for diagnosis, or not a diagnosis of ASD by inclusion criteria, k=31):[246–263, 854-865, 953]
      iii) ASD under anesthesia or antipsychotic-induced weight gain (combination treatment) (k=11):[264–272, 819, 952]
      iv) Sample size smaller than 10 participants (including withdrawn studies, k=61):[273-274, 276–333, 978]
   c) Wrong interventions (k=296):
      i) Single dose interventions or less than seven days of treatments (k=145):[333–456, 820-833, 963-969]
      ii) Different dose of the same medication or no treatment control groups (k=30):[457–476, 874-876, 958-962, 980, 982]
      iii) Combination and multimodal treatments (k=97):[477–553, 835, 839-853, 974-977]
      iv) Not pharmacological or dietary supplement interventions (k=24):[554-572, 879, 970-973]
2) Protocol and abstract conferences (no sufficient data provided) with unclear diagnostic criteria or study design (*k=12*):[573, 575-577, 579–586]
3) Already included or excluded from the previous searches (*k=300*):[587–818, 883-949, 979]

4.2.2 References of excluded records

1. Acosta MT. Pharmacotherapy in autism: where to start? Drug Discovery Today. 2004;9(11):474.
2. Aman M. The use of methylphenidate in autism. Journal of the American Academy of Child and Adolescent Psychiatry. 1988;27(6):821–822.
3. Anonymous. Single dose secretin 'no more effective than placebo' for autism. Pharmaceutical Journal. 2000;264(7077):8.
4. Anonymous. Children with autism may benefit from risperidone. The Pharmaceutical Journal. 2002;269(7210):184.
5. Anonymous. Autism: a new treatment seems unsuccessful. Child Health Alert. 2004;22:1–2.
6. Anonymous. Drug fails to subdue repetitive behavior in children with autism spectrum disorders. Harv Ment Health Lett. 2009;26(1057-5022 (Linking)):4.
7. Bates G, Willson SW. 'Use of selective serotonin reuptake inhibitors in children with pervasive developmental disorder: risk of treatment emergent mania'. Developmental Medicine & Child Neurology. 2003;45(5):359; author reply 360.

8. Bent S, Bertoglio K, Hendren RL. Regarding omega-3 fatty acids in severe autism. Archives of Medical Research. 2009;40(1):64; author reply 65.
9. Bou Khalil R. Would some cannabinoids ameliorate symptoms of autism? European Child and Adolescent Psychiatry. 2012;21(4):237–238.
10. Brulotte J, Bukutu C FVohra, Sunita, Vohra S. Complementary, holistic, and integrative medicine: fish oils and neurodevelopmental disorders.
11. Buitelaar JK, Willemsen-Swinkels S, Engel, H. Treatment of autism and self-injury with naltrexone. Xth world congress of psychiatry; 1996 aug 23-28; madrid, spain. 1996.
12. Buitelaar JK, Willemsen-Swinkels S, van Engel, H. Naltrexone in children with autism. Journal of the American Academy of Child and Adolescent Psychiatry. 1998;37(8):800–802.
13. Caicedo C, Williams SH. Risperidone improves behavior in children with autism. Journal of family practice. 2002;51(11):915.
14. Campbell M. The effect of neuroleptics on cognition and diagnosis, and their influence on stereotypies. Journal of Mental Deficiency Research. 1987;31:220–225.
15. Campbell M. Fenfluramine treatment of autism. Journal of Child Psychology & Psychiatry & Allied Disciplines. 1988;29(1):1–10.
16. Campbell M. Resolved: Autistic children should have a trial of naltrexone": Affirmative rebuttal. Journal of the American Academy of Child and Adolescent Psychiatry. 1996;35(2):249–250.
17. Campbell M, Harris JC. Resolved: autistic children should have a trial of naltrexone. Journal of the American Academy of Child and Adolescent Psychiatry. 1996;35(2):246-9; discussion 249-51.
18. Campbell M, Palij M. Behavioral and cognitive measures used in psychopharmacological studies of infantile autism. Psychopharmacology Bulletin. 1985;21(4):1047–1053.
19. Connors SL, Crowell DE. Secretin and autism: the role of cysteine. Journal of the American Academy of Child and Adolescent Psychiatry. 1999;38(7):795–796.
20. Corey R. Hopkins. ACS Chemical Neuroscience Molecule Spotlight on STX209 (Arbaclofen). ACS Chem Neurosci. 2011;2(8):381. doi:10.1021/cn200019z.
21. Cysneiros RM, Terra VC, Machado HR, et al. May the best friend be an enemy if not recognized early: possible role of omega-3 against cardiovascular abnormalities due to antipsychotics in the treatment of autism. Arquivos de Neuro-Psiquiatria. 2009;67(3):922–926.
22. Farber JM. Fenfluramine and autism. Developmental Medicine & Child Neurology. 1986;28(6):817–818.
23. Fenfluramine in Autism. New England Journal of Medicine. 1982;307(23):1450–1451. doi:10.1056/NEJM198212023072314.
24. Fox NS, Roman AS. Beta 2 adrenergic agents and autism. American Journal of Obstetrics & Gynecology. 2010;203(4):e15. doi:10.1016/j.ajog.2010.06.064.
25. Ghanizadeh A. Methionine sulfoximine may improve inflammation in autism, a novel hypothesized treatment for autism. Archives of Medical Research. 2010;41(8):651–652.
26. Ghanizadeh A. Ghrelin as a promising therapeutic target for co-occurring autism and epilepsy. Epilepsy & Behavior. 2011;20(2):420–421.
27. Gordon D. Early negative results not the last word on secretin/autism story. Gastroenterology. 2000;118(2):250.
28. Goulden KJ. In children with autism, is intravenous secretin more effective than placebo in improving social skills, communication, behaviour or global functioning? Part B: Clinical commentary. Paediatrics and child health;9(4):246.
29. Heisler MA, Guidry JR, McQueen JM, Heck AM. Comment: Secretin for autism: Unproven treatment or ineffective treatment? [5] (mulitiple letters). Annals of Pharmacotherapy. 2002;36(7):1294–1295.
30. Holl, er E. Translational experimental therapeutics of inflammation and fever in autism spectrum disorder: Hot tubs, locus coeruleus modulation and helminth therapy. Neuropsychopharmacology;2:S92-S93.

31. Jayach, ra S. Is secretin effective in treatment for autism spectrum disorders (ASD)? International Journal of Psychiatry in Medicine. 2005;35(1):99–101.
32. Johnson KP, Malow BA. Assessment and pharmacologic treatment of sleep disturbance in autism.
33. Johnson SM, Holl, er E. Evidence that eicosapentaenoic acid is effective in treating autism. J Clin Psychiatry;64(7):848–849.
34. Jorgensen M, Thomsen PH, Henriksen JH. [Secretin treatment of autism?]. Ugeskrift for Laeger. 2002;164(12):1676.
35. Lensing P, Klingler D, Panksepp J, et al. [Opiate hypothesis of the origin of early childhood autism and sequelae for psychopharmacotherapy]. Zeitschrift fur Kinder- und Jugendpsychiatrie. 1992;20(3):185–196.
36. Leventhal BL, Cook, Edwin H., Jr., Lord C. The irony of autism. Archives of General Psychiatry. 1998;55(7):643–644.
37. Levitas A, Zarcone JR, Hellings JA, Schroeder SR. Reader response to Zarcone et al. (2001), "Effects of risperidone on aberrant behavior in persons with developmental disabilities: I. A double-blind crossover study using multiple measures" (multiple letters). American Journal on Mental Retardation. 1;108(3):212–216.
38. Lightdale JR, Heyman MB. Secretin: cure or snake oil for autism in the new millennium? Journal of Pediatric Gastroenterology & Nutrition. 1999;29(2):114–115.
39. Linday LA. Saccharomyces boulardii: potential adjunctive treatment for children with autism and diarrhea. Journal of child neurology. 2001;16(5):387.
40. Longhurst JG, Potenza MN, McDougle CJ. Autism. New England Journal of Medicine. 1997;337(21):1555–1556.
41. Mehlinger R, Scheftner WA, Poznanski E. Fluoxetine and Autism. Journal of the American Academy of Child and Adolescent Psychiatry. 1990;29(6):985. doi:10.1097/00004583-199011000-00032.
42. Meyer-Lindenberg A. Impact of prosocial neuropeptides on human brain function. Advances in Vasopressin and Oxytocin - From Genes to Behaviour to Disease. 2008:463–470.
43. Munarriz R, Bennett L, Goldstein I. Risperidone in children with autism and serious behavioral problems. New England Journal of Medicine. 2002;347(23):1890-1; author reply 1890-1.
44. Nau JY. The effectiveness of bumetanide in the management of autism. Revue Medicale Suisse. 2017;13(556):722–723.
45. Niederhofer H. Also Topiramate might have some benefit in psychopharmacological treatment of autism.
46. Niederhofer H. Treating autism pharmacologically: also tacrine might improve symptomatology in some cases.
47. Paczynski M. Piracetam: a novel therapy for autism? Journal of Autism & Developmental Disorders. 1997;27(5):628–630.
48. Parsonson BS. Using psychoactive medication to intervene in children's behaviour: an evidence-based practice? J Prim Health Care. 2009;1(1):6–10.
49. Petryk S. In children with autism, is intravenous secretin more effective than placebo in improving social skills, communication, behaviour or global functioning? Part A: Evidence-based answer and summary. Paediatrics and child health;9(4):244–245.
50. Pretest for January 2004. CNS Spectrums. 2003;8(12):962–964. doi:10.1017/S1092852900028741.
51. Rickards EH, Prendergast M. Fluoxetine and serotonin in autism. The American Journal of Psychiatry. 1992;149(6):851.
52. Riml, B. High dose vitamin B6 and magnesium in treating autism: response to study by Findling et al. Journal of Autism & Developmental Disorders. 1998;28(6):581–582.
53. Riml, B. Secretin: real therapeutic potential (response). Journal of Pediatric Gastroenterology & Nutrition. 2000;30(2):113; author reply 113-4.

54. Said SI, Bodanszky M. Secretin treatment for autism. New England Journal of Medicine. 2000;342(16):1217–1218.
55. Stokstad E. Stalled Trial for Autism Highlights Dilemma of Alternative Treatments. Science. 2008;321(5887):326. doi:10.1126/science.321.5887.326.
56. Strayhorn J. More on methylphenidate in autism. Journal of the American Academy of Child and Adolescent Psychiatry. 1989;28(2):299.
57. Theoharides TC, Asadi S. Unwanted interactions among psychotropic drugs and other treatments for autism spectrum disorders. Journal of Clinical Psychopharmacology. 2012;32(4):437–440. doi:10.1097/JCP.0b013e31825e00e4.
58. Volkmar FR. Lessons from secretin. New England Journal of Medicine. 1999;341(24):1842–1844.
59. Wink LK, Erickson CA, Stigler KA, McDougle CJ. Riluzole in autistic disorder. Journal of Child and Adolescent Psychopharmacology. 2011;21(4):375–379. doi:10.1089/cap.2010.0154.
60. Albertini G, Majolini L, Di Gennaro G, Quarato P, Scoppetta C, Onorati P. Oral dyskinesia induced by fluoxetine therapy for infantile autism. Pediatric Neurology. 2004;31(1):76.
61. Alessi NE. Ziprasidone in autism. Journal of the American Academy of Child and Adolescent Psychiatry. 2003;42(6):622–623.
62. Alessi N, Alkhouri I, Fluent T, Quinlan P, Williams K. Haloperidol decanoate in children. Journal of the American Academy of Child and Adolescent Psychiatry. 2001;40(8):865–866.
63. Bernhardt EB, Walsh KH, Posey DJ, McDougle CJ. Memantine for comorbid obsessive-compulsive disorder and Asperger disorder suggests a link in glutamatergic dysregulation. Journal of Clinical Psychopharmacology. 2011;31(5):673–675.
64. Caixeta M, Caixeta L. [Topiramate reduces irritability and self-injuries in autistic children]. Revista Brasileira de Psiquiatria. 2005;27(4):345–346.
65. Craven-Thuss B, Nicolson R. Amoxapine treatment of interfering behaviors in autistic disorder. Journal of the American Academy of Child and Adolescent Psychiatry. 2003;42(5):515–516.
66. Decocq G, K, elaft N, Compagnon M. [Effects of naltrexone on automutilation behavior in autistic psychosis]. Presse Medicale. 1996;25(7):305.
67. Demb HB. Risperidone in young children with pervasive developmental disorders and other developmental disabilities. Journal of Child & Adolescent Psychopharmacology. 1996;6(1):79–80.
68. Doan RJ. Risperidone for insomnia in PDDs. Canadian Journal of Psychiatry - Revue Canadienne de Psychiatrie. 1998;43(10):1050–1051.
69. Duggal HS. Mood stabilizers in Asperger’s syndrome. Australian and New Zealand Journal of Psychiatry. 2001;35(3):390–391.
70. Duggal HS. Ziprasidone for maladaptive behavior and attention-deficit/hyperactivity disorder symptoms in autistic disorder.
71. Erickson CA, Chambers JE. Memantine for disruptive behavior in autistic disorder. J Clin Psychiatry;67(6):1000.
72. Fisman S, Steele M, Pipher B. Risperidone in PDD. Journal of the American Academy of Child and Adolescent Psychiatry. 1998;37(1):15–16.
73. Gudarzi SS, Yasamy M, Akhondzadeh S. Cyproheptadine in treatment of autism. European Psychiatry: the Journal of the Association of European Psychiatrists. 2002;17(4):230–231.
74. Horrigan JP, Barnhill LJ. More on melatonin. Journal of the American Academy of Child and Adolescent Psychiatry;36(8):1014.
75. Kapetanovic S. Oxcarbazepine in youths with autistic disorder and significant disruptive behaviors.
76. Leboyer M, Bouvard MP, Dugas M. Effects on naltrexone on infantile autism. Lancet. 1988;1(8587):715.

77. Magen J. Negative results with clomipramine. Journal of the American Academy of Child and Adolescent Psychiatry. 1993;32(5):1079–1080.
78. Malek-Ahmadi P, Simonds JF. Olanzapine for autistic disorder with hyperactivity. Journal of the American Academy of Child and Adolescent Psychiatry. 1998;37(9):902.
79. McCracken JT, Martin W. Clonidine side effect. Journal of the American Academy of Child and Adolescent Psychiatry. 1997;36(2):160–161.
80. Niederhofer H FDamodharan, Senthil Kumar, Damodharan SK FJoji, Rekha, Joji R FCorfield, Alison, Corfield A. Atomoxetine treating patients with Autistic disorder.
81. Ozbayrak KR. Sertraline in PDD. Journal of the American Academy of Child and Adolescent Psychiatry. 1997;36(1):7–8.
82. Pardini M, Guida S, Gialloreti LE. Aripiprazole treatment for coprophagia in autistic disorder. J Neuropsychiatry Clin Neurosci. 2010;22(4):451-s.e33-451.e33. doi:10.1176/jnp.2010.22.4.451.e33.
83. Posey DI, Litwiller M, Koburn A, McDougle CJ. Paroxetine in autism. Journal of the American Academy of Child and Adolescent Psychiatry. 1999;38(2):111–112.
84. Realmuto GM, August GJ, Garfinkel BD. Clinical effect of buspirone in autistic children. Journal of Clinical Psychopharmacology. 1989;9(2):122–125.
85. Shahani L. Use of lithium for sexual obsessions in Asperger's disorder. J Neuropsychiatry Clin Neurosci. 2012;24(4):E17. doi:10.1176/appi.neuropsych.11090232.
86. Snead RW, Boon F, Presberg J. Paroxetine for self-injurious behavior. Journal of the American Academy of Child and Adolescent Psychiatry. 1994;33(6):909–910.
87. Sporn A, Pinsker H. Use of stimulant medication in treating pervasive developmental disorder. The American Journal of Psychiatry. 1981;138(7):997.
88. Stigler KA, Erickson CA, Mullett JE, Posey DJ, McDougle CJ. Paliperidone for irritability in autistic disorder. Journal of Child & Adolescent Psychopharmacology. 2010;20(1):75–78.
89. Szabo CP, Bracken C. Imipramine and Asperger’s. Journal of the American Academy of Child and Adolescent Psychiatry. 1994;33(3):431–432.
90. Tufan AE, Kutlu H. Adjunctive quetiapine may help depression comorbid with pervasive developmental disorders. Progress in Neuro-Psychopharmacology and Biological Psychiatry. 2009;33(8):1570–1571. doi:10.1016/j.pnpbp.2009.09.009.
91. Todd RD. Fluoxetine in autism. The American Journal of Psychiatry. 1991;148(8):1089.
92. Aman MG, Armstrong SA. Regarding secretin for treating autistic disorder. Journal of Autism & Developmental Disorders. 2000;30(1):71–72.
93. Arnold LE, Aman MG, Li X, et al. Research Units of Pediatric Psychopharmacology (RUPP) autism network randomized clinical trial of parent training and medication: one-year follow-up. Journal of the American Academy of Child and Adolescent Psychiatry. 2012;51(11):1173–1184.
94. August GJ, Raz N, Baird TD. Effects of fenfluramine on behavioral, cognitive, and affective disturbances in autistic children. Journal of Autism & Developmental Disorders. 1985;15(1):97–107.
95. Awad GA. The use of selective serotonin reuptake inhibitors in young children with pervasive developmental disorders: some clinical observations. Canadian Journal of Psychiatry - Revue Canadienne de Psychiatrie. 1996;41(6):361–366. doi:10.1177/070674379604100606.
96. Bent S, Ailarov A, Dang KT, Widjaja F, Lawton BL, Hendren RL. Open-Label Trial of Vitamin D3 Supplementation in Children with Autism Spectrum Disorder. Journal of Alternative & Complementary Medicine. 2017;23(5):394–395.
97. Bent S, Lawton B, Warren T, et al. Identification of urinary metabolites that correlate with clinical improvements in children with autism treated with sulforaphane from broccoli. Molecular Autism. 2018;9:35.
98. Birmaher B, Quintana H, Greenhill LL. Methylphenidate treatment of hyperactive autistic children. Journal of the American Academy of Child and Adolescent Psychiatry. 1988;27(2):248–251.

99. Campbell M, Adams P, Small AM, Tesch LM, Curren EL. Naltrexone in infantile autism. Psychopharmacology Bulletin. 1988;24(1):135–139.
100. Campbell M, Deutsch SI, Perry R, Wolsky BB, Palij M. Short-term efficacy and safety of fenfluramine in hospitalized preschool-age autistic children: an open study. Psychopharmacology Bulletin. 1986;22(1):141–147.
101. Campbell M, Perry R, Polonsky BB, Deutsch SI, Palij M, Lukashok D. An open study of fenfluramine in hospitalized young autistic children. Journal of Autism & Developmental Disorders. 1986;16(4):495–506.
102. ChiCTR1800016113, Children's Hospital of Chongqing Medical, University. Prospective Study of Vitamin A and Vitamin D Treatment in Children with Autism Spectrum Disorders. 2018.
103. ChiCTR1800016473, Xuanwu Hospital, Capital Medical University. The effect of improving gut microbiota for treating children with Autism Spectrum Disorder(ASD). 2018.
104. ChiCTR-CCC-13004498, Bethune First Hospital of Jilin, University. The associate of polymorphisms of vitamin D metabolism-related genes with autism, and the treatment of autism with vitamin D. 2013.
105. ChiCTR-ROC-14005442, Children's Hospital, Chongqing Medical University. The roles of vitamin A and its nuclear receptors in the pathogenesis of autism spectrum disorder. 2014.
106. CTRI/2018/06/014379, All India Institute of, Ayurveda. Effect of Abhaya Ghrita and Panchabhautika Taila Nasya in treating Autism Spectrum Disorders in children. 2018.
107. Desousa A. An Open-label Trial of Risperidone and Fluoxetine in Children with Autistic Disorder. Indian Journal of Psychological Medicine. 2010;32(1):17–21.
108. Deutsch SI, Milstoc M, Platovsky G, Wolsky BB, Perry R, Green WH. Cholinesterase activities in blood in infantile autism. Biological Psychiatry. 1987;22(2):234–236.
109. Erickson CA, Ray B, Maloney B, et al. Impact of acamprosate on plasma amyloid-beta precursor protein in youth: a pilot analysis in fragile X syndrome-associated and idiopathic autism spectrum disorder suggests a pharmacodynamic protein marker. Journal of Psychiatric Research. 2014;59:220–228.
110. Erickson CA, Wink LK, Early MC, et al. Brief report: pilot single-blind placebo lead-in study of acamprosate in youth with autistic disorder. Journal of autism and developmental disorders. 2014;44(4):981–987.
111. EUCTR2012-001616-33-GB, Forest Research Institute I. An Open-Label Study Of The Safety And Tolerability Of Memantine In Pediatric Patients With Autism, Asperger's Disorder, Or Pervasive Developmental Disorder Not Otherwise Specified (PDD-NOS). 2012.
112. Frye RE, Sequeira JM, Quadros EV, James SJ, Rossignol DA. Cerebral folate receptor autoantibodies in autism spectrum disorder. Molecular Psychiatry. 2013;18(3):369–381.
113. Geier DA, Geier MR. A clinical trial of combined anti-androgen and anti-heavy metal therapy in autistic disorders. Neuroendocrinology Letters. 2006;27(6):833–838.
114. Ghaziuddin M, Tsai L, Ghaziuddin N. Fluoxetine in autism with depression. Journal of the American Academy of Child and Adolescent Psychiatry. 1991;30(3):508–509.
115. Gupta S. Treatment of children with autism with intravenous immunoglobulin. Journal of child neurology. 1999;14(3):203–205.
116. Gvozdjakova A, Kucharska J, Ostatnikova D, Babinska K, Nakladal D, Crane FL. Ubiquinol improves symptoms in children with autism. Oxidative medicine & cellular longevity. 2014;2014:798957.
117. IRCT20130504013215N2, Shahid Beheshti University of Medical, Sciences. Probiotics Effect in Reducing Behavioral Symptoms and Severity of Autism. 2018.
118. IRCT2015100424337N1, Vice chancellor for research, Tehran University of Medical Sciences. Cerebrolysin effect On cognitive and verbal aspects of children suffering from autism spectrum disorder. 2015.
119. James SJ, Cutler P, Melnyk S, et al. Metabolic biomarkers of increased oxidative stress and impaired methylation capacity in children with autism. American Journal of Clinical Nutrition. 2004;80(6):1611–1617.

120. Joshi G, Biederman J, Wozniak J, et al. Response to second generation antipsychotics in youth with comorbid bipolar disorder and autism spectrum disorder. CNS Neuroscience & Therapeutics. 2012;18(1):28–33.
121. JPRN-UMIN000006558, Department of Neuropsychiatry, Faculty of Medical Sciences University of Fukui. A research of efficacy and safety of aripiprazole treatment for the behavioral symptoms in subjects with pervasive developmental disordes. 2011.
122. JPRN-UMIN000016770, Environmental M, Council for N. Vitamin D Status in Autism Spectrum Disorder and the Efficacy of Vitamin D Supplementation in Autistic Children. 2015.
123. JPRN-UMIN000021433, Assiut u. Ketogenic diet versus gluten free casein free diet in autistic children: a case-control study. 2016.
124. Julie Hess, Johnny Matson, Daniene Neal, et al. A Comparison of Psychotropic Drug Side Effect Profiles in Adults Diagnosed With Intellectual Disabilities and Autism Spectrum Disorders. Journal of Mental Health Research in Intellectual Disabilities. 2010;3(2):85–96. doi:10.1080/19315861003690588.
125. Kaluzna-Czaplinska J, Jozwik-Pruska J, Chirumbolo S, Bjorklund G. Tryptophan status in autism spectrum disorder and the influence of supplementation on its level. Metabolic Brain Disease. 2017;32(5):1585–1593.
126. Kaluzna-Czaplinska J, Michalska M, Rynkowski J. Vitamin supplementation reduces the level of homocysteine in the urine of autistic children. Nutrition Research. 2011;31(4):318–321.
127. Kaluzna-Czaplinska J, Socha E, Rynkowski J. B vitamin supplementation reduces excretion of urinary dicarboxylic acids in autistic children. Nutrition Research. 2011;31(7):497–502.
128. Legido A, Goldenthal M, Garvin B, et al. Effect of a combination of carnitine, coenzyme q10 and alpha-lipoic acid (mitococktail) on mitochondrial function and neurobehavioral performance in children with autism spectrum disorder. Neurology. Conference: 70th Annual Meeting of the American Academy of Neurology, AAN. 2018;90(15).
129. Martineau J, Barthelemy C, Jouve J, Muh J-P, LeLord G. Monoamines (serotonin and catecholamines) and their derivatives in infantile autism: Age-related changes and drug effects. Developmental Medicine & Child Neurology. 1992;34(7):593–603.
130. Martsenkovsky I. Open-label atomoxetine for attention-deficit/ hyperactivity disorder symptoms associated with high-functioning autism spectrum disorders. European Child and Adolescent Psychiatry;1:S221.
131. Martsenkovsky I, Bikshaieva I, Vashenko O, Martsenkovsky D. Memantine therapy of cognitive, behavioral, and social dysfunction in children witch autism spectrum disorders (ASD). International Journal of Neuropsychopharmacology;1:138.
132. Max Horovitz, Johnny L. Matson, Alyse Barker. The relationship between symptoms of autism spectrum disorders and psychotropic medication use in infants and toddlers. Research in autism spectrum disorders. 2012;6(4):1406–1411. doi:10.1016/j.rasd.2011.05.013.
133. Meguid NA, Hashish AF, Anwar M, Sidhom G. Reduced serum levels of 25-hydroxy and 1,25-dihydroxy vitamin D in Egyptian children with autism. Journal of alternative and complementary medicine (new york, N.Y.). 2010;16(6):641–645. doi:10.1089/acm.2009.0349.
134. Milin R, Simeon JG, Batth S, Thatte S, Dare GJ, Walker S. An open trial of olanzapine in children and adolescents with Asperger Disorder. Journal of Clinical Psychopharmacology. 2006;26(1):90–92.
135. Min Guo, Jiang Zhu, Ting Yang, et al. Vitamin A and vitamin D deficiencies exacerbate symptoms in children with autism spectrum disorders. Nutritional neuroscience. 2018;0(0):1–11. doi:10.1080/1028415X.2017.1423268.
136. Minderaa RB FAnderson, G M, Anderson GM FVolkmar, F R, Volkmar FR FAkkerhuis, G W, Akkerhuis GW FCohen, D J, Cohen DJ. Urinary 5-hydroxyindoleacetic acid and whole blood serotonin and tryptophan in autistic and normal subjects.
137. NCT00325572, Thrasher Research F, Penn State U. Evaluation and Treatment of Copper/Zinc Imbalance in Children With Autism. 2006.

138. NCT00549562, Ortho-McNeil Janssen Scientific Affairs, L. L. C., Indiana University School of, Medicine. Study of Paliperidone ER in Adolescents and Young Adults With Autism. 2007.
139. NCT00619190, Bristol-Myers S, University of North Carolina, Chapel Hill. Study of Aripiprazole to Treat Children and Adolescents With Autism. 2008.
140. NCT01050582, amp, Johnson Pharmaceutical R, Development LLC, Johnson. A Study to Evaluate the Safety and the Effects of Risperidone Compared With Other Atypical Antipsychotic Drugs on the Growth and Sexual Maturation in Children. 2010.
141. NCT01205282, Holl, Bloorview Kids Rehabilitation H, Evdokia A. Dose Finding Study of Pioglitazone in Children With Autism Spectrum Disorders (ASD) (PIO). 2010.
142. NCT01352611, University of M-C. Open Label Treatment of Severe Tactile Defensiveness With Intrathecal Baclofen. 2011.
143. NCT01731119, Foundation of Hope, North Carolina, University of North Carolina, Chapel Hill. Study of Lurasidone in Treating Antipsychotic Naive or Quasi-Naive Children and Adolescents. 2012.
144. NCT01881737, Stanford U. A Study of Pregnenolone in the Treatment of Individuals With Autism. 2011.
145. NCT03432065, Massachusetts General H. A Pilot Study of Buspirone for the Treatment of Anxiety in Youth With Autism Spectrum Disorders. 2018.
146. Perry R, Bangaru BS. Secretin in autism. Journal of Child & Adolescent Psychopharmacology. 1998;8(4):247–248.
147. Perry R, Campbell M, Green WH, et al. Neuroleptic-related dyskinesias in autistic children: a prospective study. Psychopharmacology Bulletin. 1985;21(1):140–143.
148. Plioplys AV. Intravenous immunoglobulin treatment in autism. Journal of Autism & Developmental Disorders. 2000;30(1):73–74.
149. Ritvo ER, Freeman BJ, Yuwiler A, et al. Study of fenfluramine in outpatients with the syndrome of autism. Journal of pediatrics. 1984;105(5):823–828.
150. Simon-Soret C, Borenstein P. [A trial of bromocriptine in the treatment of infantile autism]. Presse Medicale. 1987;16(26):1286.
151. Stubbs EG, Budden SS, Jackson RH, Terdal LG, Ritvo ER. Effects of fenfluramine on eight outpatients with the syndrome of autism. Developmental medicine and child neurology. 1986;28(2):229–235.
152. Tachibana M, Kagitani-Shimono K, Mohri I, et al. Long-term administration of intranasal oxytocin to early adolescents with autistic spectrum disorder. Developmental medicine and child neurology. 2012;54:66.
153. Valdovinos MG, Bailey L, Taylor SL. Examining risperidone use in those diagnosed with autism 1 year after FDA approval. J Clin Psychiatry. 2010;71(5):651–652.
154. Zeiner P, Gjevik E, Weidle B. Response to atomoxetine in boys with high-functioning autism spectrum disorders and attention deficit/hyperactivity disorder. Acta Paediatr. 2011;100(9):1258–1261. doi:10.1111/j.1651-2227.2011.02263.x.
155. Ritvo ER, Freeman BJ, Yuwiler A, et al. Fenfluramine treatment of autism: UCLA collaborative study of 81 patients at nine medical centers. Psychopharmacology Bulletin. 1986;22(1):133–140.
156. Malow BA, Adkins KW, McGrew SG, Surdyka K, Goldman SE, Wofford D. Impact of supplemental melatonin on sleep and behavior in children with autism spectrum disorders. Sleep. 2009:A64.
157. Malow BA, Adkins KW, McGrew SG, Surdyka K, Wofford D. Supplemental melatonin improves sleep in children with autism spectrum disorders. Annals of Neurology. 2009;1:S31.
158. Malow B, Adkins KW, McGrew SG, et al. Melatonin for sleep in children with autism: a controlled trial examining dose, tolerability, and outcomes. Journal of autism and developmental disorders. 2012;42(8):1729-37; author reply 1738.
159. NCT00927030, Eunice Kennedy Shriver National Institute of Child, Health, Human D, V, erbilt U. Melatonin for Sleep in Children With Autism. 2009.

160. Chez MG, Buchanan CP, Aimonovitch MC, et al. Double-blind, placebo-controlled study of L-carnosine supplementation in children with autistic spectrum disorders. Journal of child neurology. 2002;17(11):833–837.
161. Buitelaar JK, Dekker ME, Ree JM, Engel, H. A controlled trial with ORG 2766, an ACTH-(4-9) analog, in 50 relatively able children with autism. European neuropsychopharmacology. 1996;6(1):13–19.
162. Verbaten MN, Kemner C, Buitelaar JK, et al. Effects of ORG-2766 on brain event-related potentials of autistic children. Psychiatry research. 1996;63(1):33–45.
163. Klykylo WM, Feldis D, O'Grady D, Ross DL, Halloran C. Clinical effects of fenfluramine in ten autistic subjects. Journal of Autism & Developmental Disorders. 1985;15(4):417–423.
164. NCT01962870, National Institute of Mental, Health, Stanford U. The Role of Vasopressin in the Social Deficits of Autism. 2013.
165. Parker K, Oztan O, Libove R, et al. Intranasal vasopressin treatment improves social abilities in children with Autism. Neuropsychopharmacology. Conference: 55th annual meeting of the american college of neuropsychopharmacology, ACNP 2016. United states. Conference start: 20161204. Conference end: 20161208. 2016;41:S341.
166. Parker KJ, Oztan O, Libove RA, et al. A randomized placebo-controlled pilot trial shows that intranasal vasopressin improves social deficits in children with autism. Sci Transl Med. 2019;11(491).
167. Luby J, Mrakotsky C, Stalets MM, et al. Risperidone in preschool children with autistic spectrum disorders: an investigation of safety and efficacy. Journal of Child & Adolescent Psychopharmacology. 2006;16(5):575–587.
168. Luby JL, Mrakotsky C, Stalets MM, et al. Risperidone in preschool children with autistic spectrum disorders: An investigation of safety and efficacy. Luby, Joan L [Ed]. 2009.
169. NCT00374764, Washington University School of, Medicine. Comparison of Applied Behavioral Analysis (ABA) Versus ABA and Risperidone. 2006.
170. Chez MG, Buchanan TM, Becker M, Kessler J, Aimonovitch MC, Mrazek SR. Donepezil hydrochloride: A double-blind study in autistic children. Journal of Pediatric Neurology. 2003;1(2):83–88.
171. Aman MG, Kasper W, Manos G, et al. Line-item analysis of the Aberrant Behavior Checklist: results from two studies of aripiprazole in the treatment of irritability associated with autistic disorder. Journal of Child & Adolescent Psychopharmacology. 2010;20(5):415–422.
172. Calarge CA, Ziegler EE, Castillo N, et al. Iron homeostasis during risperidone treatment in children and adolescents. Journal of clinical psychiatry. 2015;76(11):1500–1505.
173. Ernst M, Devi L, Silva RR, et al. Plasma beta-endorphin levels, naltrexone, and haloperidol in autistic children. Psychopharmacology Bulletin. 1993;29(2):221–227.
174. L, sberg W, Loze JY, et al. Safety and tolerability of aripiprazole in the treatment of irritability associated with autistic disorder in pediatric patients: Results from a 52-week open-label study. European Psychiatry. 2011;26.
175. Lewis D, Owen R, Couch DM. Efficacy and safety of aripiprazole for the treatment of irritability associated with autistic disorder in children and adolescents (6-17 years): results from two 8-week, randomized, double-blind, placebo-controlled trials. Neurology. 2009;72(11):A428, Abstract no: S50.005.
176. Locascio JJ, Malone RP, Small AM, et al. Factors related to haloperidol response and dyskinesias in autistic children. Psychopharmacology Bulletin. 1991;27(2):119–126.
177. NCT00211770, Mount Sinai School of, Medicine. Use of Functional Behavioral Assessments to Evaluate Stereotypy and Repetitive Behaviors in a Double-blind, Placebo Controlled Trials of Various Medications Used to Treat Children With Autism. 2005.
178. NCT00399698, Ohio State U. Study to Determine Whether There Are Any Cognitive or Motor Effects From Taking the Medicine Risperidone. 2006.
179. Owada K, Okada T, Munesue T, et al. Quantitative facial expression analysis revealed the efficacy and time course of oxytocin in autism. Brain. 2019.

180. Robb AS, Andersson C, Bellocchio EE, et al. Safety and tolerability of aripiprazole in the treatment of irritability associated with autistic disorder in pediatric subjects (6-17 years Old): results from a pooled analysis of 2 studies. Primary care companion to the journal of clinical psychiatry. 2011;13(1):e1-e9.
181. Varni JW, H, en BL, et al. Effect of Aripiprazole 2 to 15 mg/d on Health-Related Quality of Life in the Treatment of Irritability Associated with Autistic Disorder in Children: a Post Hoc Analysis of Two Controlled Trials. Clinical therapeutics. 2012;34(4):980–992.
182. EUCTR2006-005346-37-NL, Company L, Eli L. A Randomized, Double-Blind, Placebo-Controlled Maintenance of Effect Study of Olanzapine in the Treatment of Disruptive Behavioral Symptoms in Children and Adolescents with Pervasive Developmental Disorders - HGMR. 2006.
183. Dolske MC, Spollen J, McKay S, Lancashire E, Tolbert L. A preliminary trial of ascorbic acid as supplemental therapy for autism. Progress in neuro-psychopharmacology & biological psychiatry. 1993;17(5):765–774.
184. Findling RL, Mankoski R, Timko K, et al. A randomized controlled trial investigating the safety and efficacy of aripiprazole in the long-term maintenance treatment of pediatric patients with irritability associated with autistic disorder. Journal of clinical psychiatry. 2014;75(1):22–30.
185. NCT01227668. Phase IV Long-term Maintenance Study of Aripiprazole in the Treatment of Irritability Associated With Autistic Disorder.
186. NTR294, Adolescent P, Accare, Division University Center for Child. Risperidone in Children and Adolescents with severe disruptive behavior problems. 2005.
187. Tolbert L, Haigler T, Waits MM, Dennis T. Brief report: lack of response in an autistic population to a low dose clinical trial of pyridoxine plus magnesium. Journal of autism and developmental disorders. 1993;23(1):193–199.
188. EUCTR2012-001568-31-GB, Forest Research Institute I. A Double-Blind, Placebo-Controlled, Randomized Withdrawal Study of the Safety and Efficacy of Memantine in Pediatric Patients with Autism, Asperger's Disorder, or Pervasive Developmental Disorder Not Otherwise Specified (PDD-NOS) Previously Treated with Memantine. 2012.
189. Hardan AY, Hendren RL, Aman MG, et al. Efficacy and safety of memantine in children with autism spectrum disorder: Results from three phase 2 multicenter studies. Autism. 2019;23(8):2096–2111.
190. Bonisch E. [Experiences with pyrithioxin in brain-damaged children with autistic syndrome]. Praxis der Kinderpsychologie und Kinderpsychiatrie. 1968;17(8):308–310.
191. Bruce AG. Lucidril for autism. New Zealand Medical Journal. 1971;73(466):173.
192. Buck RP de. Antiautistic effect of flupentixol. Acta Psychiatrica Belgica. 1974;74(5):520–525.
193. Campbell M, Fish B, Korein J, Shapiro T, Collins P, Koh C. Lithium and chlorpromazine: a controlled crossover study of hyperactive severely disturbed young children. Journal of autism and childhood schizophrenia. 1972;2(3):234–263.
194. Campbell M, Small AM, Holl, et al. A controlled crossover study of triiodothyronine in autistic children. Journal of autism and childhood schizophrenia. 1978;8(4):371–381.
195. Collard J. [Sulpiride, an unusual antiautistic and thymanaleptic neuroleptic agent]. Therapeutique. 1970;46(5):503–506.
196. Collard J, Dufrasne M, Fraipont J. The place of sulpiride (Dogmatil) in chemotherapy of autism. Acta Psychiatrica Belgica. 1971;71(1):42–55.
197. Collard J, Fraipont J, Dufrasne M. [Sulpiride, autism and course under MMPI]. Lille Medical. 1972;17: Suppl 1:33-6.
198. Hoshino Y, Yashima Y, Ishige K, Kaneko M, Kumashiro H. Effects of small doses of haloperidol on autistic children. Fukushima Journal of Medical Science. 1979;26(1):43–54.
199. Kehrer HE. [Infantile autism and drug therapy]. Bibliotheca Psychiatrica. 1978(157):91–97.
200. Kurtis LB. Clinical study of the response to nortriptyline on autistic children. International Journal of Neuropsychiatry. 1966;2(4):298–301.

201. Luyckx A. [Clinical experimentation with a long-acting neuroleptic fluspirilene (R 6218)]. Acta Psychiatrica Belgica. 1972;72(6):748–755.
202. Miller B, Wallis H. The mode of action of sulpiride in autistic children. A double blind study. [German] Uber Die Wirkungsweise Von Sulpirid Bei Autistischen Kindern. Eine Doppelblinduntersuchungen. Munchener Medizinische Wochenschrift. 1979;121(19):667–669.
203. Moss N, Boverman H. Megavitamin therapy for autistic children. The American Journal of Psychiatry. 1978;135(11):1425–1426.
204. Rimland B., Callaway E, Dreyfus P. The effect of high doses of vitamin B6 on autistic children: a double-blind crossover study. American journal of psychiatry. 1978;135(4):472–475.
205. Ritvo ER, Yuwiler A, Geller E, et al. Effects of L-dopa in autism. Journal of autism and developmental disorders. 1971;1(2):190–205. doi:10.1007/BF01537957.
206. Simmons JQ, Leiken SJ, Lovaas OI, Schaeffer B, Perloff B. Modification of autistic behavior with LSD-25. The American Journal of Psychiatry. 1966;122(11):1201–1211.
207. Campbell M, Anderson LT, Meier M, et al. A comparison of haloperidol and behavior therapy and their interaction in autistic children. Journal of the American Academy of Child Psychiatry. 1978;17(4):640–655.
208. Campbell M, Anderson LT, Meier M. A comparison of haloperidol, behavior therapy, and their interaction in autistic children [proceedings]. Psychopharmacology Bulletin. 1979;15(2):84–86.
209. Crewther D, Bauer I, Crewther S, Pipingas A. Non-linear visual evoked potentials - A sensitive assay for nutraceutical effects. Clinical EEG and Neuroscience. 2011;42:127–128.
210. Dager SR, Corrigan NM, Richards T, Dunner D, Lyoo IK, Renshaw PF. Imaging brain metabolism: evidence for altered brain bioenergetics in bipolar disorder. Bipolar disorders. 2014;16:25.
211. Feng C, Hackett PD, DeMarco AC, et al. Oxytocin and vasopressin effects on the neural response to social cooperation are modulated by sex in humans. Brain Imaging and Behavior. 2015;9(4):754–764.
212. Goldstein R, Joja O, Psatta DM, Petrescu M, Paraschiv I, Popa M. Vasotocin improves intelligence and attention in mentally retarded children. Physiology & behavior. 1989;46(6):967–970.
213. H, en BL, Sahl R, Hardan AY. Guanfacine in children with autism and/or intellectual disabilities. Journal of developmental and behavioral pediatrics. 2008;29(4):303–308.
214. Mancini J, Dubus JC, Jouve E, et al. Effect of desipramine on patients with breathing disorders in RETT syndrome. Annals of Clinical and Translational Neurology. 2018;5(2):118–127.
215. NCT00205699, National Institute of Mental, Health, Washington University School of, Medicine. Metabolic Effects of Antipsychotics in Children. 2005.
216. NCT02149823, James, J. Peters Veterans Affairs Medical Center, Visn 3 Mental Illness Research, Education, Clinical C, Maria de las Mercedes Perez, Rodriguez. Examining Dose-Related Effects of Oxytocin on Social Cognition Across Populations. 2014.
217. SLCTR/2009/006, Habib J. Effectiveness of Omega-3 and Omega-6 in childhood behaviour disorders. 2009.
218. Xu X, Li J, Chen Z, Kendrick KM, Becker B. Oxytocin reduces top-down control of attention by increasing bottom-up attention allocation to social but not non-social stimuli - A randomized controlled trial. Psychoneuroendocrinology. 2019;108:62–69.
219. Appleton RE, Gringras P. Mends: The use of melatonin in children with neuro-developmental disorders and impaired sleep-A randomised, double-blind, placebocontrolled, parallel trial. Archives of disease in childhood;1:A1.
220. Uillemsen Svinkels, S. Kh N., Bautelaar Ia K, Neikhof GI, Engel, Kh. Failure of naltrexone hydrochloride to reduce self-injurious and autistic behavior in mentally retarded adults: double-blind placebo-controlled studies. Sotsialnaia I klinicheskaia psikhiatriia. 1997:63–74.

221. Willemsen-Swinkels SH, Buitelaar JK, Nijhof GJ, Engl, H. Failure of naltrexone hydrochloride to reduce self-injurious and autistic behavior in mentally retarded adults. Double-blind placebo-controlled studies. Archives of General Psychiatry. 1995;52(9):766–773.
222. Boone KM, Gracious B, Klebanoff MA, et al. Omega-3 and -6 fatty acid supplementation and sensory processing in toddlers with ASD symptomology born preterm: A randomized controlled trial.[Erratum appears in Early Hum Dev. 2018 Mar 2;:; PMID: 29506901]. Early Human Development. 2017;115:64–70.
223. Keim SA, Gracious B, Boone KM, et al. omega-3 and omega-6 Fatty Acid Supplementation May Reduce Autism Symptoms Based on Parent Report in Preterm Toddlers. Journal of Nutrition. 2018;148(2):227–235.
224. Sheppard KW, Boone KM, Gracious B, et al. Effect of Omega-3 and -6 Supplementation on Language in Preterm Toddlers Exhibiting Autism Spectrum Disorder Symptoms. Journal of autism and developmental disorders. 2017;47(11):3358–3369.
225. Wasdell MB, Jan JE, Bomben MM, et al. A randomized, placebo-controlled trial of controlled release melatonin treatment of delayed sleep phase syndrome and impaired sleep maintenance in children with neurodevelopmental disabilities. Journal of pineal research. 2008;44(1):57–64.
226. Simonoff E, Taylor E, Baird G, Bernard S. Commentary: RCT of optimal dose methylphenidate in children and adolescents with severe ADHD and ID-a reply to Arnold (2013). Journal of Child Psychology & Psychiatry & Allied Disciplines. 2013;54(6):703–704.
227. Simonoff E, Taylor E, Baird G, et al. Randomized controlled double-blind trial of optimal dose methylphenidate in children and adolescents with severe attention deficit hyperactivity disorder and intellectual disability. Journal of child psychology and psychiatry, and allied disciplines. 2013;54(5):527–535.
228. Joseph L, Grant P, Swedo S. A placebo-controlled trial of riluzole for treatment of childhood-onset obsessive compulsive disorder. Neuropsychopharmacology. 2011;36:S228-s229.
229. NCT00251303, National Institute of Mental, Health. Riluzole to Treat Child and Adolescent Obsessive-Compulsive Disorder With or Without Autism Spectrum Disorders. 2005.
230. Sandman CA. Opiate Control of Self-Injury in Mental Retardation. 8th european college of neuropsychopharmacology congress. 1995.
231. Sandman CA. Opiate Control of Self-Injury in Mental Retardation CONFERENCE ABSTRACT. 8th european college of neuropsychopharmacology congress. Venice, italy. 30th september - 4th october, 1995. 1995.
232. Sandman CA, Hetrick W, Talyor D, Marion S, Chicz-Demet A. Uncoupling of proopiomelanocortin (POMC) fragments is related to self- injury. Peptides. 2000;21(6):785–791.
233. Tyrer P, Oliver-Africano P, Romeo R, et al. Neuroleptics in the treatment of aggressive challenging behaviour for people with intellectual disabilities: A randomised controlled trial (NACHBID). Health Technology Assessment;13(21):1–54.
234. Overwater IE, Rietman AB, Mous SE, et al. A randomized controlled trial with everolimus for IQ and autism in tuberous sclerosis complex. Neurology. Conference: 65th American Academy of Neurology Annual Meeting. San Diego, CA United States. Conference Publication. 2019.
235. CTRI/2017/01/007738, Newron Pharmaceuticals Sp A. A 6 Month Study to Evaluate the Efficacy, Safety and Tolerability of drug â??Sarizotanâ?? in Patients with Rett Syndrome having difficulty in breathing. This is a Randomized, Double-Blind (the doctor and the patient not knowing the treatment), Placebo (inactive substance) -Controlled study. 2017.
236. Diego-Otero Y de, Calvo-Medina R, Quintero-Navarro C, et al. A combination of ascorbic acid and alpha-tocopherol to test the effectiveness and safety in the fragile X syndrome: study protocol for a phase II, randomized, placebo-controlled trial. Trials;15:345.
237. NCT03569631. A 2-Period Crossover Study of BPN14770 in Adults Males With Fragile X Syndrome. https://ClinicalTrials.gov/show/NCT03569631.

238. O'Leary HM, Kaufmann WE, Barnes KV, et al. Placebo-controlled crossover assessment of mecasermin for the treatment of Rett syndrome. Annals of Clinical and Translational Neurology;5(3):323–332.
239. Percy A, Glaze D, Neul J, et al. Trofinetide, a novel IGF-1 related treatment for neurodevelopmental disorders, demonstrates efficacy for children and adolescents with rett syndrome. Annals of Neurology;82:S342-S343.
240. Percy A, Glaze DG, Neul JL, et al. Trofinetide, a novel IGF-1 related treatment for neurodevelopmental disorders, demonstrates efficacy for children and adolescents with Rett syndrome. Journal of the american academy of child and adolescent psychiatry. Conference: 64th annual meeting american academy of child and adolescent psychiatry, AACAP 2017. United states. 2017;56(10):S168-s169.
241. Sahu JK, Gulati S, Sapra S, et al. Effectiveness and safety of donepezil in boys with fragile x syndrome: a double-blind, randomized, controlled pilot study. Journal of child neurology. 2013;28(5):570–575.
242. Smith-Hicks CL, Gupta S, Ewen JB, et al. Randomized open-label trial of dextromethorphan in Rett syndrome. Neurology. 2017;89(16):1684–1690.
243. Siper P, Tavassoli T, George-Jones J, et al. The sensory domain as a target for treatment in ASD clinical trials: Electrophysiological and behavioral markers of therapeutic change. Biological Psychiatry;83:S370.
244. Al Olaby RR, Hagerman R, Abbeduto L, Tassone F. Identification of molecular biomarkers predictive of response to targeted treatment in fragile X syndrome and autism spectrum disorder. Journal of Intellectual Disability Research;61:828.
245. Greiss Hess L, Fitzpatrick SE, Nguyen DV, et al. A Randomized, Double-Blind, Placebo-Controlled Trial of Low-Dose Sertraline in Young Children With Fragile X Syndrome. Journal of developmental and behavioral pediatrics. 2016;37(8):619–628.
246. Jung SS, Lee YC. A double blind study of dimethylglycine treatment in children with autism. [Korean]. Tzu Chi Medical Journal. 2000;12(2):111–121.
247. Grimaldi R, Gibson GR, Vulevic J, et al. A prebiotic intervention study in children with autism spectrum disorders (ASDs). Microbiome. 2018;6(1):133.
248. NCT02720900, University of R, Clasado L. Prebiotic Intervention for Autism Spectrum Disorders. 2015.
249. Naruse H, Hayashi T, Takesada M, Nakane A, Yamazaki K. Metabolic changes in aromatic amino acids and monoamines in infantile autism and development of new treatment related to the finding. No to hattatsu = brain and development. 1989;21(2):181–189.
250. Naruse H, Takesada M, Nakane Y, et al. Clinical Evaluation of R-Tetrahydrobiopterin (SUN 0588) on Infantile Autism: a Double-Blind Comparative Study Using Placebo as a Control. Rinsho iyaku (journal of clinical therapeutics and medicines). 1990;6(7):1343–1368.
251. A multi centered double blind trial of pimozide (Orap), haloperidol and placebo for abnormal behavior in children using crossover design. Rinsho hyoka /clinical evaluation. 1980;8(3):629–673.
252. Naruse H, Nagahata M, Nakane Y. A multi-center double-blind trial of pimozide (Orap), haloperidol and placebo in children with behavioral disorders, using crossover design. Acta Paedopsychiatrica. 1982;48(4):173–184.
253. Moorthy MP, Srinivasan AV, Bhanu K, Mugundan K, Sivakumar S. L-Carnosine in pediatric cognitive disorders. Neurorehabilitation and Neural Repair;32:372–373.
254. Parracho, H. M. R. T., Gibson GR, Knott F, Bosscher D, Kleerebezem M, McCartney AL. A double-blind, placebo-controlled, crossover-designed probiotic feeding study in children diagnosed with autistic spectrum disorders. International Journal of Probiotics and Prebiotics. 2010;5(2):69–74.
255. Kohler JA, Shortl, G., Rolles CJ. Effect of fenfluramine on autistic symptoms. British medical journal (clinical research ed.). 1987;295(6603):885.

256. Adams JB, Holloway C. Pilot study of a moderate dose multivitamin/mineral supplement for children with autistic spectrum disorder.[Erratum appears in J Altern Complement Med. 2005 Aug;11(4):749]. Journal of Alternative & Complementary Medicine. 2004;10(6):1033–1039.
257. Adams JB, Audhya T, McDonough-Means S, et al. Effect of a vitamin/mineral supplement on children and adults with autism. BMC pediatrics. 2011;11:111.
258. NCT01225198, Autism Research I, Legacy F, Arizona State U. Vitamin/Mineral Supplement for Children and Adults With Autism. 2010.
259. Barthelemy C, Garreau B, Leddet I, et al. Relevance of behavior scales and dosage levels of homovanillic acid in the urine to controlling the effects of a treatment combining vitamin B6 and magnesium administered to children with autistic behavior. [French] Interet des echelles de comportement et des dosages de l'acide homovanilique urinaire pour le controle des effets d'un traitement associant vitamine B6 et magnesium chez des enfants ayant un comportement autistique. Neuropsychiatrie de l'Enfance et de l'Adolescence. 1983;31(5):289–301.
260. Barthelemy C, Garreau B, Leddet I, et al. Value of behavior scales and urinary homovanillic acid determinations in monitoring the combined treatment with vitamin B6 and magnesium of children displaying autistic behavior. Neuropsychiatrie de l'Enfance et de l'Adolescence. 1983;31(5):289–301.
261. Lelord G, Callaway E, Muh JP. Clinical and biological effects of high doses of vitamin B6 and magnesium on autistic children. Acta vitaminologica ET enzymologica. 1982;4(1):27–44.
262. Lelord G, Muh JP, Barthelemy C, Martineau J, Garreau B, Callaway E. Effects of pyridoxine and magnesium on autistic symptoms: Initial observations. Journal of Autism & Developmental Disorders. 1981;11(2):219–230.
263. Garstang J, Wallis M. Randomized controlled trial of melatonin for children with autistic spectrum disorders and sleep problems. Child Care Health Dev. 2006;32(5):585–589.
264. NCT00709202, Stanley Medical Research I, Nathan Kline Institute for Psychiatric, Research. Efficacy and Tolerability Study of Betahistine to Ameliorate Antipsychotic Associated Weight Gain in Adolescents and Young Adults. 2008.
265. Reeves GM, Keeton C, Correll CU, et al. Improving metabolic parameters of antipsychotic child treatment (IMPACT) study: rationale, design, and methods. Child Adolesc Psychiatry Ment Health;7(1):31.
266. Aman MG, Hollway JA, Veenstra-V, et al. Effects of Metformin on Spatial and Verbal Memory in Children with ASD and Overweight Associated with Atypical Antipsychotic Use. Journal of Child and Adolescent Psychopharmacology. 2018;28(4):266–273.
267. Anagnostou E, Aman MG, H, et al. Metformin for Treatment of Overweight Induced by Atypical Antipsychotic Medication in Young People With Autism Spectrum Disorder: a Randomized Clinical Trial. JAMA psychiatry. 2016;73(9):928–937.
268. Anonymous. Erratum: metformin for treatment of overweight induced by atypical antipsychotic medication in young people with autism spectrum disorder: a randomized clinical trial (JAMA Psychiatry (2016) 73: 9 (928-937)). JAMA psychiatry. 2016;73(12):1295.
269. Garfunkel D, Anagnostou EA, Aman MG, et al. Pharmacogenetics of Metformin for Medication-Induced Weight Gain in Autism Spectrum Disorder. Journal of Child & Adolescent Psychopharmacology. 2019;29(6):448–455.
270. NCT01825798, Massachusetts General H, V, et al. Treatment of Overweight Induced by Antipsychotic Medication in Young People With Autism Spectrum Disorders (ASD). 2013.
271. Larr AS, Vakhrusheva J, Marino P, Maayan L. A double-blind, placebo controlled trial of betahistine to ameliorate antipsychotic associated weight gain in adolescents and young adults: Preliminary safety and efficacy data. Schizophrenia Research;1:S355.
272. Li BL, Yuen V-Y, Zhang N, et al. Intranasal dexmedetomidine with and without buccal midazolam for procedural sedation in autistic children: a double-blind randomised controlled trial. The lancet. Conference: chinese academy of medical sciences health summit, CAMS 2017. China. 2017;390:26.

273. NCT03008889, Emory U. A Feasibility Study of N-acetylcysteine for Self-injurious Behavior in Children With Autism Spectrum Disorder. 2016.
274. NCT01395953, Massachusetts General H. Double-blind Trial of Buspirone for the Treatment of Anxiety in Youth With Autism Spectrum Disorders. 2011.
275. [The published manuscript of the trial NCT00965068 was found in the update search in September 2021, which met the eligibility criteria (at least 10 participants were randomized). Therefore, it was removed from the list of excluded studies.]
276. NCT00318162, Jerusalem Institute for Child, Development, Hadassah Medical O. Trial of Low-Dose Naltrexone for Children With Pervasive Developmental Disorder (PDD). 2006.
277. NCT02007447, University of Sao Paulo General, Hospital. Oxytocin in Adolescents With Autism Spectrum Disorders. 2013.
278. Jonas C, Etienne T, Barthelemy C, Jouve J, Mariotte N. [Clinical and biochemical value of Magnesium + vitamin B6 combination in the treatment of residual autism in adults]. Therapie. 1984;39(6):661–669.
279. Stigler K, Wang Y, McDonald B, et al. Effects of aripiprazole on brain circuitry in youth with pervasive developmental disorders. Neuropsychopharmacology;1:S367.
280. ISRCTN72571312, Coventry U. A placebo controlled pilot study to explore the affects of GABA tea on children with autistic spectrum conditions. 2017.
281. Jaselskis CA, Cook EH, Fletcher KE, Leventhal BL. Clonidine treatment of hyperactive and impulsive children with autistic disorder. Journal of Clinical Psychopharmacology. 1992;12(5):322–327.
282. Woodard C, Groden J, Goodwin M, Bodfish J. A placebo double-blind pilot study of dextromethorphan for problematic behaviors in children with autism. Autism. 2007;11(1):29–41.
283. Wirojanan J, Jacquemont S, Diaz R, et al. The efficacy of melatonin for sleep problems in children with autism, fragile X syndrome, or autism and fragile X syndrome.[Erratum appears in J Clin Sleep Med. 2010 Aug 15;6(4):preceding 311]. Journal of Clinical Sleep Medicine. 2009;5(2):145–150.
284. Williams PG, Allard AM, Sears L, Dalrymple N, Bloom AS. Brief report: Case reports on naltexone use in children with autism: Controlled observations regarding benefits and practical issues of medication management. Journal of autism and developmental disorders. 2001;31(1):103–108.
285. Sanchez LE, Adams PB, Uysal S, Hallin A, Campbell M, Small AM. A comparison of live and videotape ratings: clomipramine and haloperidol in autism. Psychopharmacology Bulletin. 1995;31(2):371–378.
286. S, man CA. Beta-endorphin disregulation in autistic and self-injurious behavior: a neurodevelopmental hypothesis. Synapse (new york, N.Y.). 1988;2(3):193–199.
287. Sandman CA. B-endorphin disregulation in autistic and self-injurious behavior: A neurodevelopmental hypothesis. Synapse. 1988;2(3):193–199.
288. Ross DL, Klykylo WM, Hitzemann R. Reduction of elevated CSF beta-endorphin by fenfluramine in infantile autism. Pediatric Neurology. 1987;3(2):83–86.
289. Reiss AL, Egel AL, Feinstein C, Goldsmith B, Borengasser-Caruso MA. Effects of fenfluramine on social behavior in autistic children. Journal of autism and developmental disorders. 1988;18(4):617–625.
290. Pritchard WS, Raz N, August GJ. No effect of chronic fenfluramine on the P300 component of the event-related potential. International journal of neuroscience. 1987;35(1):105–110.
291. Piggott LR, Gdowski CL, Villanueva D, Fischhoff J, Frohman CF. Side effects of fenfluramine in autistic children. Journal of the American Academy of Child Psychiatry. 1986;25(2):287–289.
292. Ney P, Neal T, Manku MS. Double blind cross-over trial with fenfluramine. Canadian Journal of Psychiatry - Revue Canadienne de Psychiatrie. 1988;33(6):574.

293. NCT02812368, Autism Treatment N, Autism Intervention Research Network on Physical, Health, Ohio State U. Clonidine for Sleep Disturbances in Children With Autism Spectrum Disorder. 2016.
294. NCT02552147, Autism S, Yale U. Nicotinic Cholinergic Modulation as a Novel Treatment Strategy for Aggression Associated With Autism. 2015.
295. NCT02414451, University of M-C. Trial of Propranolol in Adults and Adolescents With ASD and Predictors of Response. 2015.
296. NCT02111551, University of Colorado D. Phase I Nicotinic Agonist Treatment Trial for Autism. 2014.
297. NCT02094651, University of L, Boston Children's H. Treatment of Children With Autism Spectrum Disorders and Epileptiform EEG With Divalproex Sodium. 2014.
298. NCT01887132, National Institute of Mental, Health. A Trial of the Drug Donepezil for Sleep Enhancement and Behavioral Change in Children With Autism. 2013.
299. NCT01734941, Hadassah Medical O. TSO in Pediatric Autistic Spectrum Disorders. 2012.
300. NCT01248130, Massachusetts General H. Omega-3 Fatty Acids Monotherapy in Children and Adolescents With Autism Spectrum Disorders. 2010.
301. NCT01078844, Forest L, Johns Hopkins U. Memantine in Adult Autism Spectrum Disorder. 2010.
302. NCT00467753, Dentistry of New J, National Alliance for Research on, Schizophrenia, Depression, University of M. Oxcarbazepine Versus Placebo in Childhood Autism. 2007.
303. NCT02081027, Children's Hospital Medical Center, Cincinnati. Pilot Study of Riluzole for Drug-Refractory Irritability in Autism Spectrum Disorders. 2013.
304. Wink LK, Adams R, Horn PS, et al. A randomized placebo-controlled cross-over pilot study of riluzole for drug-refractory irritability in autism spectrum disorder. Journal of autism and developmental disorders. 2018.
305. Wink LK, Adams R, Horn PS, et al. A Randomized Placebo-Controlled Cross-Over Pilot Study of Riluzole for Drug-Refractory Irritability in Autism Spectrum Disorder. Journal of Autism & Developmental Disorders. 2018;48(9):3051–3060.
306. Linday L, Tsiouris JA, Cohen IL, DeCresce R. Famotidine treatment of young children with autistic spectrum disorders. 152nd annual meeting of the american psychiatric association; 1999 may 15-20; washington, DC. 1999.
307. Linday LA, Tsiouris JA, Cohen IL, Shindledecker R, DeCresce R. Famotidine treatment of children with autistic spectrum disorders: pilot research using single subject research design. Journal of neural transmission (vienna, austria : 1996). 2001;108(5):593–611.
308. Stephenson MB. Famotidine (Pepcid) and Autistic Spectrum Disorders: A Reason for Optimism, or for Heartburn? The Scientific Review of Mental Health Practice: Objective Investigations of Controversial and Unorthodox Claims in Clinical Psychology, Psychiatry, and Social Work. 2002;1(2):184–188.
309. Kamiyama M, Kuriyama S, Watanabe M. A clinical study of pyridoxine treatment for pervasive developmental disorders with hypersensitivity to sound. [Japanese]. No to Hattatsu [Brain & Development]. 2006;38(4):277–282.
310. Kuriyama, Kamiyama, Watanabe, et al. Pyridoxine treatment in a subgroup of children with pervasive developmental disorders. Developmental Medicine & Child Neurology. 2002;44(4):284–286.
311. Lewis AS, van Schalkwyk GI, Lopez MO, Volkmar FR, Picciotto MR, Sukhodolsky DG. An Exploratory Trial of Transdermal Nicotine for Aggression and Irritability in Adults with Autism Spectrum Disorder. Journal of autism and developmental disorders. 2018;48:1–10.
312. Beeghly JH, Kuperman S, Perry PJ, Wright GJ, Tsai LY. Fenfluramine treatment of autism: relationship of treatment response to blood levels of fenfluramine and norfenfluramine. Journal of autism and developmental disorders. 1987;17(4):541–548.
313. Black SL. Naltrexone in infantile autism. Journal of Autism & Developmental Disorders. 1994;24(2):236–239.

314. Leboyer M, Bouvard MP, Lensing P, et al. Opioid excess hypothesis of autism: A double-blind study of naltrexone. Brain Dysfunction. 1990;3(5):285–298.
315. Leboyer M, Bouvard MP, Launay J-M, et al. A double-blind study of naltrexone in infantile autism. Journal of Autism & Developmental Disorders. 1992;22(2):309–319.
316. Leboyer M, Bouvard MP, Launay JM, et al. Brief report: a double-blind study of naltrexone in infantile autism. Journal of autism and developmental disorders. 1992;22(2):309–319.
317. Leboyer M, Bouvard MP, Launay JM, et al. Opiate hypothesis in infantile autism? Therapeutic trials with naltrexone. Encephale. 1993;19(2):95–102.
318. Buchsbaum MS, Holl, er E, et al. Effect of fluoxetine on regional cerebral metabolism in autistic spectrum disorders: a pilot study. The international journal of neuropsychopharmacology. 2001;4(2):119–125.
319. Coggins TE, Morisset C, Krasney L, Frederickson R, Holm VA, Raisys VA. Brief Report: Does fenfluramine treatment enhance the cognitive and communicative functioning of autistic children. Journal of Autism & Developmental Disorders. 1988;18(3):425–434.
320. Dollfus S, Petit M, Menard JF, Lesieur P. Amisulpride versus bromocriptine in infantile autism: a controlled crossover comparative study of two drugs with opposite effects on dopaminergic function. Journal of autism and developmental disorders. 1992;22(1):47–60.
321. Dollfus S, Petit P, Menard JF. Pharmacoclinical study of an agonist and an antagonist of dopamine in early infantile autism. Neuropsychiatrie de l'Enfance et de l'Adolescence. 1992;40(5):300–309.
322. Dollfus S, Petit M, Launay JM, et al. Platelet serotonin in infantile autism. Cross-over effects of a dopamine agonist and an antagonist. Encephale. 1992;18(6):605–610.
323. Dollfus S, Petit M, Garnier JP, et al. Catecholamines in autistic disorder: Effects of amisulpride and bromocriptine in a controlled crossover study. Journal of Child and Adolescent Psychopharmacology. 1993;3(3):145–156.
324. Ho HH, Lockitch G, Eaves L, Jacobson B. Blood serotonin concentrations and fenfluramine therapy in autistic children. Journal of pediatrics. 1986;108(3):465–469.
325. Zingarelli G, Ellman G, Hom A, Wymore M, Heidorn S, Chicz-Demet A. Clinical effects of naltrexone on autistic behavior. American Journal on Mental Retardation. 1992;97(1):57–63.
326. NCT01170325, National Institute of Mental, Health. A Study of Divalproex Sodium in Children With ASD and Epileptiform EEG. 2010.
327. Fankhauser MP, Karumanchi VC, German ML, Yates A, Karumanchi SD. A double-blind, placebo-controlled study of the efficacy of transdermal clonidine in autism. Journal of clinical psychiatry. 1992;53(3):77–82.
328. NCT00936182, Related D, The International Child Development Resource, Center, Thoughtful H, The Center for A. Study of Fluconazole in Children With Autism Spectrum Disorder. 2009.
329. Cohen IL, Campbell M, Posner D. A study of haloperidol in young autistic children: a within-subjects design using objective rating scales. Psychopharmacology Bulletin. 1980;16:63–65.
330. EUCTR2016-000106-11-FR. EVALUATION OF THE EFFICIENCY OF TREATMENT BY BUMETANIDE ON AUTISTIC CHILDREN WITH A KNOWN ETIOLOGY: MULTICENTER AND DOUBLE-BLIND STUDY WITH RANDOMIZED PARALLEL GROUP, AGAINST PLACEBO. https://www.clinicaltrialsregister.eu/ctr-search/trial/2016-000106-11/FR/.
331. NCT02947880, University Hospital L. Evaluation of the Efficiency of Treatment by BUMETANIDE on Autistic Children With a Known Ethiology. 2016.
332. EUCTR2008-003712-36-FR, Assistance Publique, Hopitaux De Paris. Etude de la réponse clinique et neurofonctionnelle à la fluoxétine dans l'autisme infantile - FAIR. 2008.
333. NCT00873834, Assistance Publique - Hôpitaux de, Paris. Fluoxetine Essay in Children With Autism. 2009.
334. NCT00889538, Cumberl, Pharmaceuticals, Norton H, University of L. Study of Glutathione, Vitamin C and Cysteine in Children With Autism and Severe Behavior Problems. 2009.

335. NCT02140112, Coronado Biosciences I. Efficacy and Safety of Trichuris Suis Ova (TSO) as Compared to Placebo in Autism Spectrum Disorder. 2014.
336. NCT00376194, National Institute of Mental, Health. Mercury Chelation to Treat Autism. 2006.
337. Holl, er E, Ferretti CJ, et al. Trichuris suis ova (TSO) as an immuneinflammatory treatment for repetitive behaviors in ASD. Neuropsychopharmacology. 2013;38:S391-s392.
338. Holl, er E, Ferretti CJ, Taylor BP, Noone RH, Racine E. Trichuris Suis Ova (TSO) as an immuneinflammatory treatment for repetitive behaviors in autism spectrum disorders (ASD). European neuropsychopharmacology. 2015;2:S723.
339. Hollander E, Uzunova G, Taylor BP, et al. Randomized crossover feasibility trial of helminthic Trichuris suis ova versus placebo for repetitive behaviors in adult autism spectrum disorder. World J Biol Psychiatry. 2018:1–9.
340. NCT01040221, Simons F, Montefiore Medical C. Trichuris Suis Ova in Autism Spectrum Disorders. 2009.
341. ACTRN12609000784213, University University of New South, Wales. Oxytocin and social interactions in young people with autism spectrum disorders. 2009.
342. Dadds MR, MacDonald E, Cauchi A, Williams K, Levy F, Brennan J. Nasal oxytocin for social deficits in childhood autism: a randomized controlled trial. Journal of autism and developmental disorders. 2014;44(3):521–531.
343. Wray, Wilkins S, O'Connor, et al. Lack of communication and behavioural response of children with autism from single dose of intravenous porcine secretin. Cochrane Developmental, Psychosocial and Learning Problems Group. 2000.
344. Strathearn L, Kim S, Bastian DA, et al. Visual systemizing preference in children with autism: A randomized controlled trial of intranasal oxytocin. Dev Psychopathol;30(2):511–521.
345. Sponheim E, Oftedal G, Helverschou SB. Multiple doses of secretin in the treatment of autism: a controlled study. Acta paediatrica. 2002;91(5):540–545.
346. Sirigu A. How oxytocin affects the human brain and behavior. Hormone Research in Paediatrics;2:7.
347. Saklayen SS. Effects of propranolol on cognition and eye contact in autism spectrum disorder (ASD). Dissertation Abstracts International: Section B: The Sciences and Engineering. 2011;71(11):6601.
348. S, ler AD, Sutton KA, et al. Lack of benefit of a single dose of synthetic human secretin in the treatment of autism and pervasive developmental disorder. New England Journal of Medicine. 1999;341(24):1801–1806.
349. Roseman B, Schneider E, Crimmins D, et al. What to Measure in Autism Drug Trials. Journal of autism and developmental disorders. 2001;31(3):361–362.
350. Roberts W, Weaver L, Brian J, et al. Repeated doses of porcine secretin in the treatment of autism: a randomized, placebo-controlled trial. Pediatrics. 2001;107(5):E71.
351. Rahman A, Freedman R, Holl, er E. Alpha-7 nicotinic acetylcholine receptor positive allosteric modulator galantamine in autism spectrum disorder. Biological psychiatry. Conference: 73rd annual scientific convention and meeting of the society of biological psychiatry, SOBP 2018. United states. 2018;83(9):S369-s370.
352. Quintana DS, Westlye LT, Hope S, et al. Dose-dependent social-cognitive effects of intranasal oxytocin delivered with novel Breath Powered device in adults with autism spectrum disorder: a randomized placebo-controlled double-blind crossover trial. Translational psychiatry. 2017;7(5):e1136.
353. Quintana D, Westlye L, Hope S, et al. Dose-dependent social-cognitive effects of intranasal oxytocin delivered with novel breath powered device in adults with autism spectrum disorder: a randomized placebo-controlled double-blind crossover trial. Biological psychiatry. Conference: 72nd annual scientific convention and meeting of the society of biological psychiatry, SOBP 2017. United states. 2017;81(10):S167.

354. Pelphrey K. Oxytocin engages target neural systems for social motivation and social cognition. Neuropsychopharmacology. 2014;39:S63-s64.
355. Peled L, Wagner S, Perry A, Shamay-Tsoory SG. Get in touch: The role of oxytocin in social touch. Journal of Molecular Neuroscience. 2013;1:S90.
356. Owley T, Steele E, Corsello C, Risi S, McKaig K, Lord C. A double-blind, placebo-controlled trial of secretin for the treatment of autistic disorder. Medscape general medicine. 1999;1(3):e1006.
357. Owley T, McMahon W, Cook EH, et al. Multisite, double-blind, placebo-controlled trial of porcine secretin in autism. Journal of the American Academy of Child and Adolescent Psychiatry. 2001;40(11):1293–1299.
358. Novotny S, Holl, er E, et al. Increased repetitive behaviours and prolactin responsivity to oral m-chlorophenylpiperazine in adults with autism spectrum disorders. The international journal of neuropsychopharmacology. 2004;7(3):249–254.
359. Novotny S, Holl, er E, et al. Increased growth hormone response to sumatriptan challenge in adult autistic disorders. Psychiatry research. 2000;94(2):173–177.
360. Niederhofer H, Staffen W, Mair A. Immunoglobulins as an alternative strategy of psychopharmacological treatment of children with autistic disorder. Neuropsychopharmacology : official publication of the American College of Neuropsychopharmacology. 2003;28(5):1014–1015.
361. NCT03537950, King's College L. Shifting Brain Excitation-Inhibition Balance in Autism Spectrum Disorder. 2018.
362. NCT03183674, University of Sao Paulo General, Hospital. Oxytocin in Spectrum Autism Disorders. 2017.
363. NCT03033784, National Institute of Mental, Health, Emory U. Autism Oxytocin Brain Project. 2017.
364. NCT02874690, Children's Hospital Medical Center, Cincinnati. Stimulant Autism Test. 2016.
365. NCT02493426, Translational Science I, University of Minnesota C. Single Dose Intranasal Oxytocin and Cognitive Effects in Autism. 2015.
366. NCT02302209, University of California, San Francisco. Dyad Oxytocin Study (DOS). 2014.
367. NCT02278328, Simons F, Clinical Research Associates, L. L. C., Children's Hospital of P. MEG Study of STX209. 2014.
368. NCT02090829, Translational Science I, Children's Hospital of P, University of Minnesota C. Intranasal Oxytocin and Learning in Autism. 2014.
369. Kruppa JA, Gossen A, Oberwelland Weiss E, et al. Neural modulation of social reinforcement learning by intranasal oxytocin in male adults with high-functioning autism spectrum disorder: a randomized trial. Neuropsychopharmacology. 2019;44(4):749–756.
370. NCT01712464. Modulation of Reinforcement Learning. https://ClinicalTrials.gov/show/NCT01712464.
371. NCT01417026, Robert S. Intranasal Oxytocin and Learning in Autism. 2011.
372. NCT01183221, Bartz JPD. The Effects of Oxytocin on Complex Social Cognition in Autism Spectrum Disorders. 2010.
373. NCT01093768, National Institute of Mental, Health. Brain Imaging Study of Adults With Autism Spectrum Disorders. 2010.
374. Zamzow RM, Christ SE, Saklayen SS, et al. Effect of propranolol on facial scanning in autism spectrum disorder: a preliminary investigation. Journal of clinical and experimental neuropsychology. 2014;36(4):431–445.
375. Zamzow RM, Ferguson BJ, Ragsdale AS, Lewis ML, Beversdorf DQ. Effects of acute beta-adrenergic antagonism on verbal problem solving in autism spectrum disorder and exploration of treatment response markers. Journal of clinical and experimental neuropsychology. 2017;39(6):596–606.

376. Yamada T, Ohta H, Watanabe H, et al. Intranasal oxytocin restrictively improves emotion recognition for men with autism spectrum disorders. Neuropsychiatrie de l'Enfance et de l'Adolescence. 2012;60(5):S219.
377. NCT00263796, Anagnostou EMD. An fMRI Study of the Effect of Intravenous Oxytocin vs. Placebo on Response Inhibition and Face Processing in Autism. 2005.
378. NCT00065962, Human D, National Institute on D, Other Communication D, Eunice Kennedy Shriver National Institute of Child, Health. Secretin for the Treatment of Autism. 2003.
379. Unis AS, Munson JA, Rogers SJ, et al. A randomized, double-blind, placebo-controlled trial of porcine versus synthetic secretin for reducing symptoms of autism. Journal of the American Academy of Child and Adolescent Psychiatry. 2002;41(11):1315–1321.
380. Naviaux RK, Curtis B, Li K, et al. Low-dose suramin in autism spectrum disorder: a small, phase I/II, randomized clinical trial. Annals of Clinical and Translational Neurology. 2017;4(7):491–505.
381. NCT02508259, University of California, San Diego. University of California, San Diego (UCSD) Suramin Treatment Trial for Autism. 2015.
382. Narayanan A. Pharmacological modulation of functional connectivity in neuropsychological disorders. Dissertation Abstracts International: Section B: The Sciences and Engineering. 2013;74(5).
383. Molloy CA, Manning-Courtney P, Swayne S, et al. Lack of benefit of intravenous synthetic human secretin in the treatment of autism. Journal of autism and developmental disorders. 2002;32(6):545–551.
384. Marchezan J, Becker M, Schwartsmann G, et al. A Placebo-Controlled Crossover Trial of Gastrin-Releasing Peptide in Childhood Autism. Clinical neuropharmacology. 2017;40(3):108–112.
385. Lefevre A, Mottolese R, Redoute J, et al. Oxytocin Fails to Recruit Serotonergic Neurotransmission in the Autistic Brain. Cereb Cortex:1–10.
386. Krusch DA. Effects of repeated secretin administration on a subset of children with pervasive developmental disorder. Dissertation Abstracts International: Section B: The Sciences and Engineering. 2004;65(2).
387. Khan K, Corbett B, Czapansky-Beilman D, et al. The effect of intravenous secretin on gastrointestinal symptoms in autistic children: a double-blind placebo-controlled trial. Pediatric research. 2001;49(4):117a.
388. Kern JK, Miller S, Evans PA, Trivedi MH. Efficacy of porcine secretin in children with autism and pervasive developmental disorder. Journal of autism and developmental disorders. 2002;32(3):153–160.
389. JPRN-UMIN000016389, University of Fukui, Research Center for Child Mental Development Age Division. A research of efficacy and safety of oxytocin administration to detect others altruism in children and adolescents with reactive attachment disorder. 2015.
390. Honomichl RD, Goodlin-Jones BL, Burnham MM, Hansen RL, Anders TF. Secretin and sleep in children with autism. Child psychiatry and human development. 2002;33(2):107–123.
391. Holl, er E, Novotny S, et al. The relationship between repetitive behaviors and growth hormone response to sumatriptan challenge in adult autistic disorder. Neuropsychopharmacology. 2000;22(2):163–167.
392. Holl, er E, Novotny S, et al. Oxytocin infusion reduces repetitive behaviors in adults with autistic and Asperger's disorders. Neuropsychopharmacology. 2003;28(1):193–198.
393. Holl, er E, Bartz J, et al. Oxytocin increases retention of social cognition in autism. Biological Psychiatry. 2007;61(4):498–503.
394. Novotny Sherie L. Decreased repetitive behaviors in response to oxytocin challenge in adult autistic disorders. 155th annual meeting of the american psychiatric association. 2002.
395. Holl, er E. V1A antagonist (RG7713) proof of mechanism study in high functioning autism spectrum disorder: clinical, biomarker and social learning effects. Neuropsychopharmacology. 2014;39:S63.

396. Holl, er E, Valle Rubido M, et al. Affective speech recognition clinical biomarker effects of a novel vasopressin 1a receptor antagonist vs placebo in adult autism. Biological Psychiatry. 2014;75(9):324s-325s.
397. Holl, er E, Valle Rubido M, et al. Clinical and biomarker effects of a novel vasopressin 1a receptor antagonist (RG7713) vs. Placebo in high functioning adult autism. Neuropsychopharmacology. 2014;39:S374-s375.
398. NCT01474278, Hoffmann-La R. A Study of RO5028442 in Adult Male High-Functioning Autistic Patients. 2011.
399. Umbricht D, Valle Rubido M, Shik F, et al. Deficient olfaction is associated with impaired ability to recognize emotions in high functioning autistic subjects and may be improved by a vasopressin 1a receptor antagonist. Biological Psychiatry. 2014;75(9):388s.
400. Umbricht D, Valle Rubido M, Shic F, et al. Olfaction is associated with ability to recognize emotions in high functioning autistic subjects. Neuropsychopharmacology. 2014;39:S584.
401. Valle Rubido M, Holl, er E, et al. A multi-center, observational study to explore the relationship between exploratory biomarkers and functional dimensions in adults with autistic spectrum disorders. European neuropsychopharmacology. Conference: 29th european college of neuropsychopharmacology congress, ECNP 2016. Austria. Conference start: 20160917. Conference end: 20160920. 2016;26:S193.
402. Valle Rubido M, Umbricht D, Shic F, et al. Results from a phase I proof-of-mechanism study with a vasopressin 1A receptor antagonist in autism spectrum disorder. European neuropsychopharmacology. 2015;25:S646-s647.
403. Hall SS, Lightbody AA, McCarthy BE, Parker KJ, Reiss AL. Effects of intranasal oxytocin on social anxiety in males with fragile X syndrome. Psychoneuroendocrinology. 2012;37(4):509–518.
404. Greene RK, Spanos M, Alderman C, et al. The effects of intranasal oxytocin on reward circuitry responses in children with autism spectrum disorder. J Neurodev Disord;10(1):12.
405. Gordon I, Jack A, Pretzsch CM, et al. Intranasal Oxytocin Enhances Connectivity in the Neural Circuitry Supporting Social Motivation and Social Perception in Children with Autism. Scientific reports. 2016;6:35054.
406. Francis SM, Kirkpatrick MG, Wit H, Jacob S. Urinary and plasma oxytocin changes in response to MDMA or intranasal oxytocin administration. Psychoneuroendocrinology. 2016;74:92–100.
407. EUCTR2012-003750-89-DE, Philipps-University M. Empathy, Autism and Oxytocin – an investigation by means of functional magnetic resonance imaging and moleculargenetic analyses. 2012.
408. EUCTR2010-022511-18-DE, Personality P, Albert-Ludwig University Freiburg, Laboratory for Biological. Behavioral effects and neural correlates of oxytocin on social attention [Verhaltenseffekte und neuronales Korrelat von Oxytocin im Kontext sozialer Aufmerksamkeit]. 2010.
409. Kanat M, Heinrichs M, Domes G. Intranasal oxytocin enhances neural correlates of face processing in autism. Journal of Intellectual Disability Research. 2015;59:117.
410. Kanat M, Spenthof I, Riedel A, Elst LT, Heinrichs M, Domes G. Restoring effects of oxytocin on the attentional preference for faces in autism. Translational psychiatry. 2017;7(4):e1097.
411. JPRN-UMIN000005809, Department of Neuropsychiatry, Showa University School of Medicine. A single-blind and crossover study examining the efficacy of intranasal oxytocin administration for social impairments in subjects with pervasive developmental disorders. 2011.
412. Lin IF, Kashino M, Ohta H, et al. The effect of intranasal oxytocin versus placebo treatment on the autonomic responses to human sounds in autism: a single-blind, randomized, placebo-controlled, crossover design study. Mol Autism;5(1):20.

413. Corbett BA, Bales KL, Swain D, et al. Comparing oxytocin and cortisol regulation in a double-blind, placebo-controlled, hydrocortisone challenge pilot study in children with autism and typical development. J Neurodev Disord. 2016;8:32.
414. Daly EM, Deeley Q, Ecker C, et al. Serotonin and the neural processing of facial emotions in adults with autism: An fMRI study using acute tryptophan depletion. Archives of General Psychiatry. 2012;69(10):1003–1013.
415. Daly E, Ecker C, Hallahan B, et al. Response inhibition and serotonin in autism: A functional MRI study using acute tryptophan depletion. Brain. 2014;137(9):2600–2610.
416. ACTRN12609000368235, University University of S. The effect of Oxytocin (OT) on social cognition and behaviour in youth with Autism Spectrum Disorders (ASD). 2009.
417. Guastella AJ, Einfeld SL, Gray KM, et al. Intranasal oxytocin improves emotion recognition for youth with autism spectrum disorders. Biological Psychiatry. 2010;67(7):692–694.
418. Ajram L. Pharmacological modulation of excitatory/ inhibitory balance in autism spectrum disorder. European neuropsychopharmacology. 2015;25:S127.
419. Ajram L, Horder J, Mendez MA, et al. Pharmacological modulation of excitatory/inhibitory balance in autism spectrum disorder. European neuropsychopharmacology. 2015;25:S61.
420. ACTRN12614000747628, University University of S. A Within-Subject Randomized Controlled Trial on the Effects of Phenytoin on Social Cognition and Behaviour in Males aged 16 Years and Older with Autism Spectrum Disorders. 2014.
421. ACTRN12615001059550, University of S. A Within-Subject Single Dose Trial on the Effects of Bremelanotide on Social Cognition and Behaviour. 2015.
422. Althaus M, Groen Y, Wijers AA, Noltes H, Tucha O, Hoekstra PJ. Oxytocin enhances orienting to social information in a selective group of high-functioning male adults with autism spectrum disorder. Neuropsychologia. 2015;79:53–69.
423. Andari E, Duhamel JR, Zalla T, Herbrecht E, Leboyer M, Sirigu A. Promoting social behavior with oxytocin in high-functioning autism spectrum disorders. Proceedings of the national academy of sciences of the united states of america. 2;107(9):4389–4394.
424. Andari E, Schneider F, Vindras P, Mottolese R, Leboyer M, Sirigu A. Oxytocin's fingerprints in social deficits of autism spectrum disorders. [French, English] Le role de l'ocytocine dans l'autisme. Encephale;1:S18.
425. Aoki Y, Watanabe T, Abe O, et al. Oxytocin's neurochemical effects in the medial prefrontal cortex underlie recovery of task-specific brain activity in autism: a randomized controlled trial. Molecular Psychiatry. 2015;20(4):447–453.
426. Aoki Y, Yahata N, Watanabe T, et al. Oxytocin improves behavioural and neural deficits in inferring others' social emotions in autism. Brain. 2014;137:3073–3086.
427. JPRN-UMIN000002241, Department of Neuropsychiatry, Graduate School of Medicine University of Tokyo. Pilot study searching neural correlates of changes in social impairments induced by intranasal oxytocin administration in subjects with autism spectrum disorder. 2009.
428. JPRN-UMIN000004393, Department of Neuropsychiatry, Graduate School of Medicine University of Tokyo. Searching neural correlates of changes in social impairments induced by intranasal oxytocin administration and its association with genotypes related tooxytocin in subjects with autism spectrum disorder. 2010.
429. Watanabe T, Abe O, Kuwabara H, et al. Mitigation of sociocommunicational deficits of autism through oxytocin-induced recovery of medial prefrontal activity: a randomized trial. JAMA psychiatry. 2014;71(2):166–175.
430. Auyeung B, Lombardo MV, Heinrichs M, et al. Oxytocin increases eye contact during a real-time, naturalistic social interaction in males with and without autism. Translational psychiatry. 2015;5:e507.
431. Beversdorf D, Zamzow R, Ferguson B, Martin T, Lewis M, Stichter J. Predictors of response to propranolol for social functioning in autism spectrum disorder. Neurology. Conference: 66th American Academy of Neurology Annual Meeting, AAN. 2014;82(10).

432. Carey T, Ratliff-Schaub K, Funk J, Weinle C, Myers M, Jenks J. Double-blind placebo-controlled trial of secretin: effects on aberrant behavior in children with autism. Journal of autism and developmental disorders. 2002;32(3):161–167.
433. Chantiluke K, Barrett N, Giampietro V, Brammer M, Simmons A, Rubia K. Disorder-dissociated effects of fluoxetine on brain function of working memory in attention deficit hyperactivity disorder and autism spectrum disorder. Psychological medicine. 2015;45(6):1195–1205.
434. Chantiluke K, Barrett N, Giampietro V, et al. Inverse Effect of Fluoxetine on Medial Prefrontal Cortex Activation During Reward Reversal in ADHD and Autism. Cerebral cortex (new york, N.Y. : 1991). 2015;25(7):1757–1770.
435. Chantiluke K, Barrett N, Giampietro V, et al. Inverse fluoxetine effects on inhibitory brain activation in non-comorbid boys with ADHD and with ASD. Psychopharmacology. 2015;232(12):2071–2082.
436. Chez MG, Buchanan CP, Bagan BT, et al. Secretin and autism: a two-part clinical investigation. Journal of autism and developmental disorders. 2000;30(2):87–94.
437. Clark CE. Re: Secretin and autism: a two-part clinical investigation. Journal of autism and developmental disorders. 2001;31(2):248–249.
438. Riml, B. Comments on "Secretin and autism: a two-part clinical investigation" by M.G. Chez et al. Journal of autism and developmental disorders. 2000;30(2):95; discussion 97-8.
439. Coniglio SJ, Lewis JD, Lang C, et al. A randomized, double-blind, placebo-controlled trial of single-dose intravenous secretin as treatment for children with autism. Journal of pediatrics. 2001;138(5):649–655.
440. Coplan J, Souders MC, Mulberg AE, et al. Children with autistic spectrum disorders. II: parents are unable to distinguish secretin from placebo under double-blind conditions. Archives of disease in childhood. 2003;88(8):737–739.
441. Levy SE, Souders MC, Wray J, et al. Children with autistic spectrum disorders. I: comparison of placebo and single dose of human synthetic secretin. Archives of disease in childhood. 2003;88(8):731–736.
442. Corbett B, Khan K, Czapansky-Beilman D, et al. A double-blind, placebo-controlled crossover study investigating the effect of porcine secretin in children with autism. Clinical pediatrics. 2001;40(6):327–331.
443. DRKS00008984, Philipps-Universität Marburg vertreten durch das Koordinierungszentrum für Klinische, Studien. A placebo-controlled, double blind, randomised trial with crossover-design investigating the effect of oxytocin nasal spray on neuronal processes of empathy. 2015.
444. DRKS00010053, Max-Planck-Institut für Kognitions- und, Neurowissenschaften. Pacebo-controlled, double-blind, randomised phase II study with crossover-design investigating the modulatory effects of intranasal Oxytocin on social cognition in patients with Autism-Spectrum-Disorder. 2016.
445. Domes G, Heinrichs M, Kumbier E, Grossmann A, Hauenstein K, Herpertz SC. Effects of intranasal oxytocin on the neural basis of face processing in autism spectrum disorder. Biological Psychiatry. 2013;74(3):164–171.
446. Domes G, Kumbier E, Heinrichs M, Herpertz SC. Oxytocin promotes facial emotion recognition and amygdala reactivity in adults with asperger syndrome. Neuropsychopharmacology. 2014;39(3):698–706.
447. Dunn-Geier J, Ho HH, Auersperg E, et al. Effect of secretin on children with autism: a randomized controlled trial. Developmental medicine and child neurology. 2000;42(12):796–802.
448. Gordon I, Wyk BCV, Lucas MV, et al. The neural attunement effects of oxytocin in children with autism disorders. Biological Psychiatry. 2014;75(9):84s.
449. H, en BL, Hofkosh D. Secretin in Children with Autistic Disorder: A Double-Blind, Placebo-Controlled Trial. Journal of Developmental and Physical Disabilities. 2005;17(2):95–106.

450. Hegarty JP, Zamzow RM, Ferguson BJ, et al. Beta-adrenergic antagonism alters functional connectivity during associative processing in a preliminary study of individuals with and without autism. Autism. 2020;24(3):795–801.
451. Pretzsch CM, Voinescu B, Mendez MA, et al. The effect of cannabidiol (CBD) on low-frequency activity and functional connectivity in the brain of adults with and without autism spectrum disorder (ASD). Journal of psychopharmacology (oxford, england). 2019;33(9):1141–1148.
452. Pretzsch CM, Voinescu B, Lythgoe D, et al. Effects of cannabidivarin (CBDV) on brain excitation and inhibition systems in adults with and without Autism Spectrum Disorder (ASD): a single dose trial during magnetic resonance spectroscopy. Translational psychiatry. 2019;9(1):313.
453. Procyshyn TL, Lombardo MV, Lai M-C, et al. Effects of oxytocin administration on salivary sex hormone levels in autistic and neurotypical women. Molecular Autism. 2020;11(1):20.
454. Roberts TPL, Bloy L, Blaskey L, et al. A MEG Study of Acute Arbaclofen (STX-209) Administration. Front Integr Neurosci. 2019;13:69.
455. Borowiak K, von Kriegstein K. Intranasal oxytocin modulates brain responses to voice-identity recognition in typically developing individuals, but not in ASD. Translational psychiatry. 2020;10(1):221.
456. Wong NML, Findon JL, Wichers RH, et al. Serotonin differentially modulates the temporal dynamics of the limbic response to facial emotions in male adults with and without autism spectrum disorder (ASD): a randomised placebo-controlled single-dose crossover trial. Neuropsychopharmacology : official publication of the American College of Neuropsychopharmacology. 2020.
457. Urbano MR, Okwara L, Manser P, Hartmann K, Deutsch S. A trial of d-cycloserine to treat the social deficit in older adolescents and young adults with autism spectrum disorders. Neuropsychopharmacology;2:S505-S506.
458. Urbano M, Okwara L, Manser P, Hartmann K, Herndon A, Deutsch SI. A trial of D-cycloserine to treat stereotypies in older adolescents and young adults with autism spectrum disorder. Clinical neuropharmacology. 2014;37(3):69–72.
459. Urbano M, Okwara L, Manser P, Hartmann K, Deutsch SI. A trial of d-cycloserine to treat the social deficit in older adolescents and young adults with autism spectrum disorders. Journal of neuropsychiatry and clinical neurosciences. 2015;27(2):133–138.
460. Carmen Galán de Isla, Soledad Sánchez Mateos, Lourdes Franco Hernández, Rafael Bravo Santos, Montserrat Rivero Urgell, Ana Beatriz Rodríguez Moratinos, Carmen Barriga Ibars. Tryptophan-enriched antioxidant cereals improve sleep in children with autistic spectrum and attention deficit hyperactivity disorders. J Cell Neurosci Oxid Stress. 2017;9(1):608–616.
461. Galan C, Sanchez S, Franco L, Bravo R, Rodriguez A, Barriga C. Intake of tryptophanenriched cereals and its influence on the sleep of children with neurological disorders. Acta Physiologica. 2017;698:73.
462. TCTR20180414001, No. Efficacy and adverse drug reaction betweenRisperidone solution local made and original drug in treatment of autism spectrum disorders. 2018.
463. NCT02255565, Pfizer, Seattle Children's H. Dose Response Effects of Quillivant XR in Children With ADHD and Autism: A Pilot Study. 2014.
464. Adams JB, Baral M, Geis E, et al. Safety and efficacy of oral DMSA therapy for children with autism spectrum disorders: part A-medical results. BMC clinical pharmacology. 2009;9:16.
465. Adams JB, Baral M, Geis E, et al. Safety and efficacy of oral DMSA therapy for children with autism spectrum disorders: part B - behavioral results. BMC clinical pharmacology. 2009;9:17.
466. NCT00811083, Southwest College of Naturopathic, Medicine. Dimercaptosuccinic Acid (DMSA) Treatment of Children With Autism and Heavy Metal Toxicity. 2008.
467. NCT02086110, University of California D. Effect of Milk Oligosaccharides and Bifidobacteria on the Intestinal Microflora of Children With Autism. 2014.
468. NCT02383758, Organization for Autism R, Emory U. An Interdisciplinary Approach to the Treatment of Encopresis in Children With Autism Spectrum Disorders. 2015.

469. NCT03408886, Arizona State U. Microbiota Transfer Therapy for Adults With Autism Spectrum Disorder (ASD) Who Have Gastrointestinal Disorders. 2018.
470. Chan AS, Sze SL, Han YMY. An intranasal herbal medicine improves executive functions and activates the underlying neural network in children with autism. Research in autism spectrum disorders. 2014;8(6):681–691.
471. ChiCTR-TRC-12001857, The Chinese University of Hong, Kong. Herbal Nose Drop for Patients with Brain Dysfunction: A Pilot Study. 2012.
472. NCT03115671, Institute of Mental Health, Singapore. Efficacy Study of Vayarin in Children With Autism and Comorbid Attention Deficit Hyperactivity Disorder (ADHD). 2017.
473. NCT02059577, Autism Research I, Arizona State U. Nutritional and Dietary Treatment Study for Children/Adults With Autism. 2014.
474. Goodarzi M, Hemayattalab R. Bone mineral density accrual in students with autism spectrum disorders: effects of calcium intake and physical training. Research in autism spectrum disorders. 2012;6(2):690–695.
475. Johnson CR, H, en BL, Zimmer M, Sacco K. Polyunsaturated fatty acid supplementation in young children with autism. Journal of Developmental and Physical Disabilities. 2010;22(1):1–10.
476. Zhang L, Huang C-C, Dai Y, et al. Symptom improvement in children with autism spectrum disorder following bumetanide administration is associated with decreased GABA/glutamate ratios. Translational psychiatry. 2020;10(1):9.
477. Carminati GG, Gerber F, Darbellay B, et al. Using venlafaxine to treat behavioral disorders in patients with autism spectrum disorder. Progress in neuro-psychopharmacology & biological psychiatry. 2016;65:85–95.
478. Akhondzadeh S, Asadabadi M. Risperidone plus celecoxib in children with autistic disorder: a double-blind, randomized trial. British journal of clinical pharmacology. 2012;73(6):983–984.
479. Asadabadi M, Mohammadi MR, Ghanizadeh A, et al. Celecoxib as adjunctive treatment to risperidone in children with autistic disorder: a randomized, double-blind, placebo-controlled trial. Psychopharmacology. 2013;225(1):51–59.
480. IRCT138711091556N2, Tehran University of Medical, Sciences. Celecoxib and Autism. 2009.
481. IRCT2017041333406N1, Vice Chancellor for Research of Mashhad University of Medical, Sciences. The efficacy of augmentation Donepezil to risperidon in treatment of autism spectrum disorders. 2017.
482. IRCT201702171556N96, Tehran University of Medical, Sciences. Palmitoylethanolamide as adjunctive treatment of Autism: A double blind and placebo controlled trial. 2017.
483. Khalaj M, Saghazadeh A, Shirazi E, et al. Palmitoylethanolamide as adjunctive therapy for autism: Efficacy and safety results from a randomized controlled trial. Journal of Psychiatric Research. 2018;103:104–111.
484. IRCT2017013132326N1, Vice Chancellor for Research of Mashhad University of Medical, Sciences. The efficacy ofaugmentation Flavonoid Quercetin torisperidon in treatment of autism spectrum disorders. 2017.
485. IRCT201701131556N95, Tehran University of Medical, Sciences. Baclofenin the treatment of Autism. 2017.
486. Mahdavinasab S-M, Saghazadeh A, Motamed-Gorji N, et al. Baclofen as an adjuvant therapy for autism: a randomized, double-blind, placebo-controlled trial. European child & adolescent psychiatry. 2019;28(12):1619–1628.
487. IRCT2016022826802N1, Vice Chancellor for Research of Mashhad University of Medical, Sciences. Assessment the efficacy of atomoxetin(stramox) in autism spectrum disorders. 2016.
488. IRCT201602041556N86, Tehran University of Medical, Sciences. Simvastatinin the treatment of Autism. 2016.
489. Moazen-Zadeh E, Shirzad F, Karkhaneh-Yousefi MA, Khezri R, Mohammadi MR, Akhondzadeh S. Simvastatin as an Adjunctive Therapy to Risperidone in Treatment of Autism: a

Randomized, Double-Blind, Placebo-Controlled Clinical Trial. Journal of Child and Adolescent Psychopharmacology. 2018;28(1):82–89.
490. IRCT201405273930N34, Vice chancellor for research, Shiraz University of Medical sciences. Vitamin D for treating autism. 2014.
491. IRCT201402043930N33, Vice chancellor for research, Shiraz University of Medical sciences. short-term co-administration of acid folicfor treating children and adolescents with autism. 2014.
492. IRCT201110281556N29, Tehran University of Medical, Sciences. N-acetyl cysteine in the treatment of autism. 2011.
493. Nikoo M, Radnia H, Farokhnia M, Mohammadi MR, Akhondzadeh S. N-acetylcysteine as an adjunctive therapy to risperidone for treatment of irritability in autism: a randomized, double-blind, placebo-controlled clinical trial of efficacy and safety. Clinical neuropharmacology. 2015;38(1):11–17.
494. IRCT201106101556N25, Tehran University of Medical, Sciences. Amantadine in the treatment of autism. 2011.
495. Mohammadi MR, Yadegari N, Hassanzadeh E, et al. Double-blind, placebo-controlled trial of risperidone plus amantadine in children with autism: a 10-week randomized study. Clinical neuropharmacology. 2013;36(6):179–184.
496. IRCT201108155280N5, Mashhad University of Medical, Scinces. A comparative study on the effectiveness of Risperidone versus Risperidone plus naltrexone in treatment of autistic spectrum disorder in children with 6-12 years old. 2013.
497. IRCT20090117001556N107, Tehran University of Medical, Sciences. Sulforaphane as adjunctive treatment of irritability in children with Autism spectrum disorder. 2018.
498. Momtazmanesh S, Amirimoghaddam-Yazdi Z, Moghaddam HS, Mohammadi MR, Akhondzadeh S. Sulforaphane as an adjunctive treatment for irritability in children with autism spectrum disorder: A randomized, double-blind, placebo-controlled clinical trial. Psychiatry and clinical neurosciences. 2020;74(7):398–405.
499. Hendouei F, Sanjari Moghaddam H, Mohammadi MR, Taslimi N, Rezaei F, Akhondzadeh S. Resveratrol as adjunctive therapy in treatment of irritability in children with autism: A double-blind and placebo-controlled randomized trial. Journal of clinical pharmacy and therapeutics. 2020;45(2):324–334.
500. IRCT20090117001556N104, Tehran University of Medical, Sciences. Resveratrolin treatment of autism. 2017.
501. IRCT20090117001556N102, Tehran University of Medical, Sciences. Prednisolone inautism spectrum disorders. 2017.
502. Malek M, Ashraf-Ganjouei A, Moradi K, Bagheri S, Mohammadi M-R, Akhondzadeh S. Prednisolone as Adjunctive Treatment to Risperidone in Children With Regressive Type of Autism Spectrum Disorder: A Randomized, Placebo-Controlled Trial. Clinical neuropharmacology. 2020;43(2):39–45.
503. IRCT138901141556N9, Tehran University of Medical, Sciences. Tpoiramate in the treatment of autism. 2010.
504. Rezaei V, Mohammadi MR, Ghanizadeh A, et al. Double-blind, placebo-controlled trial of risperidone plus topiramate in children with autistic disorder. Progress in neuro-psychopharmacology & biological psychiatry. 2010;34(7):1269–1272.
505. Hasanzadeh E, Mohammadi MR, Ghanizadeh A, et al. A double-blind placebo controlled trial of Ginkgo biloba added to risperidone in patients with autistic disorders. Child psychiatry and human development. 2012;43(5):674–682.
506. IRCT201012031556N19, Kurdistan University of Medical, Sciences, Tehran University of Medical, Sciences. Ginkgo biloba in the treatment of autistic disorder. 2010.
507. Akhondzadeh S, Erfani S, Mohammadi MR, et al. Cyproheptadine in the treatment of autistic disorder: a double-blind placebo-controlled trial. Journal of clinical pharmacy and therapeutics. 2004;29(2):145–150.

508. Akhondzadeh S, Fallah J, Mohammadi MR, et al. Double-blind placebo-controlled trial of pentoxifylline added to risperidone: effects on aberrant behavior in children with autism. Progress in neuro-psychopharmacology & biological psychiatry. 2010;34(1):32–36.
509. IRCT138711161556N7, Tehran University of Medical, Sciences. Pentoxifylline in the treatment of autism. 2009.
510. Akhondzadeh S, Tajdar H, Mohammadi MR, et al. A double-blind placebo controlled trial of piracetam added to risperidone in patients with autistic disorder. Child psychiatry and human development. 2008;39(3):237–245.
511. Ghaleiha A, Alikhani R, Kazemi MR, et al. Minocycline as Adjunctive Treatment to Risperidone in Children with Autistic Disorder: a Randomized, Double-Blind Placebo-Controlled Trial. Journal of Child and Adolescent Psychopharmacology. 2016;26(9):784–791.
512. IRCT201302201556N50, Tehran University of Medical, Sciences. Minocycline in the treatment of autism. 2013.
513. Ghaleiha A, Asadabadi M, Mohammadi MR, et al. Memantine as adjunctive treatment to risperidone in children with autistic disorder: a randomized, double-blind, placebo-controlled trial. The international journal of neuropsychopharmacology. 2013;16(4):783–789.
514. IRCT1138901151556N10, Tehran University of Medical, Sciences. memantine in the treatment of autism. 2010.
515. Ghaleiha A, Ghyasv, M., et al. Galantamine efficacy and tolerability as an augmentative therapy in autistic children: a randomized, double-blind, placebo-controlled trial. Journal of psychopharmacology (oxford, england). 2014;28(7):677–685.
516. IRCT201204081556N40, Tehran University of Medical, Sciences. Galantamine in the treatment of autism. 2012.
517. Ghaleiha A, Mohammadi E, Mohammadi MR, et al. Riluzole as an adjunctive therapy to risperidone for the treatment of irritability in children with autistic disorder: a double-blind, placebo-controlled, randomized trial. Paediatric drugs. 2013;15(6):505–514.
518. IRCT201107281556N27, Tehran University of Medical, Sciences. Riluzole in the treatment of autism. 2011.
519. Ghaleiha A, Rasa SM, Nikoo M, Farokhnia M, Mohammadi MR, Akhondzadeh S. A pilot double-blind placebo-controlled trial of pioglitazone as adjunctive treatment to risperidone: effects on aberrant behavior in children with autism. Psychiatry research. 2015;229(1):181–187.
520. IRCT201202281556N37, Tehran University of Medical, Sciences. pioglitazone in the treatment of autism. 2012.
521. Ghanizadeh A, Ayoobzadehshirazi A. A randomized double-blind placebo-controlled clinical trial of adjuvant buspirone for irritability in autism. Pediatric Neurology. 2015;52(1):77–81.
522. IRCT201307303930N28, Shiraz University of Medical, sciences. A randomized double blind placebo controlled clinical trial of buspirone for treating autism spectrum disorders. 2014.
523. Ghanizadeh A, Moghimi-Sarani E. A randomized double blind placebo controlled clinical trial of N-Acetylcysteine added to risperidone for treating autistic disorders. BMC Psychiatry. 2013;13:196.
524. IRCT201106103930N6, Vice chancellor for research, Shiraz University of Medical sciences. N-Acetylcysteine augmentation with Rispridone in treatment of Autism in children. 2011.
525. Hajizadeh-Zaker R, Ghajar A, Mesgarpour B, Afarideh M, Mohammadi MR, Akhondzadeh S. L-Carnosine As an Adjunctive Therapy to Risperidone in Children with Autistic Disorder: a Randomized, Double-Blind, Placebo-Controlled Trial. Journal of Child and Adolescent Psychopharmacology. 2018;28(1):74–81.
526. IRCT201512081556N83, Tehran University of Medical, Sciences. L Carnosinein the treatment of Autism. 2015.
527. IRCT201101105280N3, Mashhad University of Medical, Sciences. Cyproheptadin plus Risperidon in treatment of children with Autistic Disorder: a double blind, placebo controlled study. 2011.

528. Behmanesh H, Moghaddam HS, Mohammadi M-R, Akhondzadeh S. Risperidone Combination Therapy With Propentofylline for Treatment of Irritability in Autism Spectrum Disorders: A Randomized, Double-Blind, Placebo-Controlled Clinical Trial. Clinical neuropharmacology. 2019;42(6):189–196.
529. Ayatollahi A, Bagheri S, Ashraf-Ganjouei A, Moradi K, Mohammadi MR, Akhondzadeh S. Does Pregnenolone Adjunct to Risperidone Ameliorate Irritable Behavior in Adolescents With Autism Spectrum Disorder: A Randomized, Double-Blind, Placebo-Controlled Clinical Trial? Clinical neuropharmacology. 2020.
530. Martsenkovsky I, Martsenkovska I. The safety and efficacy of memantine hydrochloride versus placebo for children under 3 years old with autism spectrum disorders. European neuropsychopharmacology. Conference: 29th european college of neuropsychopharmacology congress, ECNP 2016. Austria. Conference start: 20160917. Conference end: 20160920. 2016;26:S729.
531. ACTRN12618001029280, None, University of S. Oxytocin in Preschoolers with Autism receiving Social Learning Therapy. 2018.
532. DRKS00008952, Zentralinstitut für seelische Gesundheit, Klinik für Psychiatrie und Psychotherapie des Kindes-und Jugendalters. Oxytocin-induced enhancement of Social Skills Training in Adolescents with ASD. 2015.
533. EUCTR2010-024202-34-DE, Klinik für Psychiatrie und Psychotherapie des Kindes- und Jugendalters am Zentralinstitut für Seelische, Gesundheit. group-therapy, autism and oxytocin - an investigation with the question "Does oxytocin (OT) enhance therapy effects in autism?“. 2013.
534. Kamp-Becker I, Poustka L, Bachmann C, et al. Study protocol of the ASD-Net, the German research consortium for the study of Autism Spectrum Disorder across the lifespan: from a better etiological understanding, through valid diagnosis, to more effective health care. BMC Psychiatry. 2017;17(1):206.
535. Preckel K, Kanske P, Singer T, Paulus FM, Krach S. Clinical trial of modulatory effects of oxytocin treatment on higher-order social cognition in autism spectrum disorder: a randomized, placebo-controlled, double-blind and crossover trial. BMC Psychiatry;16(1):329.
536. Wang M, Jiang L, Tang X. Levetiracetam is associated with decrease in subclinical epileptiform discharges and improved cognitive functions in pediatric patients with autism spectrum disorder. Neuropsychiatric disease and treatment. 2017;13:2321–2326.
537. Scahill L, McDougle CJ, Aman MG, et al. Effects of risperidone and parent training on adaptive functioning in children with pervasive developmental disorders and serious behavioral problems. Journal of the American Academy of Child and Adolescent Psychiatry. 2012;51(2):136–146.
538. Rezaei M, Moradi A, Tehrani-Doost M, Hassanabadi H, Khosroabadi R. Effects of Combining Medication and Pivotal Response Treatment on Aberrant Behavior in Children with Autism Spectrum Disorder. Children. 2018;5(2):30.
539. NCT03370510, Yale U. Translating Neuroprediction Into Precision Medicine Via Brain Priming. 2017.
540. NCT03242772, Eunice Kennedy Shriver National Institute of Child, Health, Human D, Duke U. Impact of Combined Medication and Behavioral Treatment for ASD & ADHD. 2017.
541. NCT02574741, University of California, Los Angeles. Combination Treatment for Augmenting Language in Children With ASD. 2015.
542. NCT02428205, Autism Science F, University of M-C. Combined Effects of Early Behavioral Intervention and Propranolol on ASD. 2015.
543. NCT02008396. Placebo-controlled, Randomized, Blinded, Dose Finding Phase 2 Pilot Safety Study of MDMA-assisted Therapy for Social Anxiety in Autistic Adults. Http://clinicaltrials.gov/show/nct02008396. 2014.
544. NCT02008396, Los Angeles Biomedical Research, Institute, Multidisciplinary Association for Psychedelic, Studies. MDMA-assisted Therapy for Social Anxiety in Autistic Adults. 2013.

545. NCT01914939, Massachusetts Institute of T, Massachusetts General H. A Randomized, Controlled Trial of Intranasal Oxytocin as an Adjunct to Behavioral Therapy for Autism Spectrum Disorder. 2013.
546. Minshawi NF, Wink LK, Shaffer R, et al. A randomized, placebo-controlled trial of D-cycloserine for the enhancement of social skills training in autism spectrum disorders. Molecular Autism. 2016;7:2.
547. NCT01086475, United States Department of, Defense, Indiana U. D-Cycloserine and Social Skills Training in Autism Spectrum Disorders. 2010.
548. Wink LK, Minshawi NF, Shaffer RC, et al. d-Cycloserine enhances durability of social skills training in autism spectrum disorder. Molecular Autism. 2017;8:2.
549. Du L, Shan L, Wang B, et al. A Pilot Study on the Combination of Applied Behavior Analysis and Bumetanide Treatment for Children with Autism. Journal of Child & Adolescent Psychopharmacology. 2015;25(7):585–588.
550. Zhou Y-y, Huang C-j, Liu J, Luo X-r. Efficacy of medication combined with sensory integration therapy for children with comorbid high-functioning autism spectrum disorder and attention deficit and hyperactivity disorder. [Chinese]. Chinese Journal of Clinical Psychology. 2014;22(6):1137–1140.
551. Karahmadi M, Tarrahi MJ, Vatankhah Ardestani SS, Omranifard V, Farzaneh B. Efficacy of Memantine as Adjunct Therapy for Autism Spectrum Disorder in Children Aged <14 Years. Adv Biomed Res. 2018;7:131.
552. Lee SH, Shin S, Kim T-H, et al. Safety, effectiveness, and economic evaluation of an herbal medicine, Ukgansangajinpibanha granule, in children with autism spectrum disorder: a study protocol for a prospective, multicenter, randomized, double-blinded, placebo-controlled, parallel-group clinical trial. Trials. 2019;20(1):434.
553. L. Fang, XM. Jiang, YK. Huang, YX. Sun, YF. Xia, L. Wang. Efficacy of vitamin D combined with omega-3 fatty acids in treatment of children with autism spectrum disorder. Pharm Care Res. 2018;18(5):347–350.
554. Al-Ayadhi LY, Halepoto DM, Al-Dress AM, Mitwali Y, Zainah R. Behavioral Benefits of Camel Milk in Subjects with Autism Spectrum Disorder. Journal of the college of physicians and surgeons-pakistan : JCPSP. 2015;25(11):819–823.
555. Bashir S, Al-Ayadhi LY. Effect of camel milk on thymus and activation-regulated chemokine in autistic children: double-blind study. Pediatric research. 2014;75(4):559–563.
556. ChiCTR-OON-14005638, The first Hospital of Jilin, University. The therapeutic effect and mechanism of ketogenic diet for children with autism. 2014.
557. IRCT201404212017N20, Tabriz University of Medical, Sciences. The effect of gluten free diet on gastrointestinal and behavioral indices in children with ASD. 2014.
558. Karkelis S, Papadaki-Pap, reou O, Lykogeorgou M, Chrousos G. Fecal calprotectin in autistic children before and after the use of elemental diet. Journal of pediatric gastroenterology and nutrition. 2010;2:E198.
559. McColl E, Adams S, Burton N, et al. Development of double blind gluten & casein free (GFCF) test foods for autism trial. Trials. 2013;14:151dummy.
560. Navarro FA, Pearson D, Lovel, et al. Intestinal permeability and behavior in children with autism spectrum disorder (ASD) on gluten and dairy-containing diet (GD). Journal of pediatric gastroenterology and nutrition;1:E16-E17.
561. Navarro F, Pearson DA, Fatheree N, Mansour R, Hashmi SS, Rhoads JM. Are 'leaky gut' and behavior associated with gluten and dairy containing diet in children with autism spectrum disorders? Nutritional neuroscience. 2015;18(4):177–185.
562. NCT02911194, Research E, a2 Milk Company L, Northumbria U. a2 Milk for Autism and Attention-deficit Hyperactivity Disorder (ADHD). 2016.
563. Papadaki OUR, Lykogeorgou MAR, Pap, reou THA, Lianou LOU, Chrousos GEO. Elemental formula diet in autistic children SAV Karkelis. Paediatrics and child health. 2010;15:42a.

564. Pedroza Garcia KA, Ronquillo D, Palacios Delgado JR, Anaya-Loyola MA, Rosado JL. Consumption of milk products with 100% b-casein a2 improves overall gastrointestinal tolerance but had no effect on behavior of Mexican children with autism spectrum disorder. Annals of nutrition and metabolism. Conference: 21st international congress of nutrition, ICN 2017. Argentina. 2017;71:389.
565. JPRN-UMIN000015708, Shimane University School of, Medicine. Efficacy and Safety of Yokukansan in Autism Spectrum disorder: A Randomized, Multi-center, Double-Blind, Placebo-Controlled Trial. 2014.
566. Chan AS, Sze SL, Han YMY, Cheung MC. A Chan dietary intervention enhances executive functions and anterior cingulate activity in autism spectrum disorders: A randomized controlled trial. Evidence-based Complementary and Alternative Medicine. 2012;2012(262136).
567. CTRI/2015/10/006284, Ayush CCo. Study on purified and standardized UNANI Brahmi preparation to improve brain function in Autism. 2015.
568. CTRI/2016/04/006856, Central Council for Research in, Homoeopathy. Effectiveness of Homoeopathic medicines in Autism. 2016.
569. CTRI/2018/05/014017, Scsvmv U. AYURVEDA FOR AUTISM. 2018.
570. ChiCTR-IIR-16008468, Chinese, P. L. A. General Hospital. Fecal Microbiota Preparation Treatment for Autism Spectrum Disorder: A Prospective, Open-label, Randomized, Controlled Trial. 2016.
571. NCT00065936. Self-Injury: diagnosis and Treatment. Https://clinicaltrials.gov/show/nct00065936. 2003.
572. NCT03426826, Children's Hospital Los A. The Gut-Brain Study. 2018.
573. ISRCTN04516575, University of R. Investigation of WCFS1 on the gut microbiota of autistic spectrum disorder (ASD) children. 2011.
574. [The results of NCT00467818 were posted in the website of the registration. Despite the diagnostic criteria were unclear according to the registrered protocol, the study was included because the research team has experience and diagnostic criteria or evaluation tools were most probably used. The study was removed from the unclear studies and it was added in the list of included studies.]
575. NCT02674984, Texas Higher Education Control, Board, The University of Texas Health Science Center, Houston. Road to Discovery for Combination Probiotic BB-12 With LGG in Treating Autism Spectrum Disorder. 2015.
576. EUCTR2009-012102-39-IT, Azienda Ospedaliera Maggiore Della Carita‘ Di, Novara. CLINICAL STUDIES ON THE EFFECTIVENESS OF THE GLUTEN-FREE DIET AND CASEIN AND THERAPY ANTI-INFLAMMATORY BOWEL CHANGE IN PSYCHIATRIC SYMPTOMS INTESTINAL AND IN PATIENTS WITH CHILDHOOD AUTISMO. 2009.
577. IRCT2012111011421N1, Tehran University of Medical, Sciences. Effect of omega-3 supplementation on Autistic patients. 2013.
578. [The trial IRCT2015122625699N1 was found to meet the eligibility criteria, after the publication was found in the update search in September 2021. Therefore, it was added in the included studies, and was removed from the unclear studies]
579. NCT00054730, Foundation F, Cortex P. Effects of CX516 on Functioning in Fragile X Syndrome and Autism. 2003.
580. NCT00036231, Repligen C. Synthetic Human Secretin in Children With Autism and Gastrointestinal Dysfunction. 2002.
581. NCT00036244. A phase III, randomized, double-blind, placebo-controlled, multiple dose study to assess the efficacy, safety and tolerability of RG1068 (synthetic human secretin) in children with autism. Https://clinicaltrials.gov/ct2/show/nct00036244. 2002.
582. NCT00036244, Repligen C. Synthetic Human Secretin in Children With Autism. 2002.
583. EUCTR2006-006126-25-FR, Michel, Cnrs Van Der Rest. Emotional and Social deficits in Asperger syndrome - Asperger and Oxytocin. 2007.

584. EUCTR2009-009475-35-NL, jeudpsychiatrie, Karakter universitair centrum voor kinder- en. Lack of Empathy as a Symptom in various Psychiatric Disorders - Psychopathology and the Lack of Empathy. 2009.
585. Kanmani VK, Kumar S, Doshi V, Sivalingam, Nambi S. A randomised double blind placebo control study of joint attention, language, social responsiveness, behaviour and epileptic discharge following 8 weeks of levo-carnosine in children with autism spectrum disorder. Indian journal of psychiatry. Conference: 70th annual national conference of indian psychiatric society, ANCIPS 2018. India. 2018;60(5):S87.
586. Stigler KA, Hummer TA, Wang Y, McDonald BC, Saykin AJ. Social impairment is related to frontolimbic structural connectivity and functional activity in autism spectrum disorders. Neuropsychopharmacology;2:S200-S201.
587. Alaerts K, Bernaerts S, Prinsen J, Dillen C, Steyaert J, Wenderoth N. Oxytocin induces long-lasting adaptations within amygdala circuitry in autism: a treatment-mechanism study with randomized placebo-controlled design. Neuropsychopharmacology : official publication of the American College of Neuropsychopharmacology. 2020;45(7):1141–1149.
588. Arnold LE, Luna R.A, Williams K, et al. Probiotics for Gastrointestinal Symptoms and Quality of Life in Autism: a placebo-controlled pilot trial. In-press.
589. Ballester P, Martinez MJ, Inda M-D-M, et al. Evaluation of agomelatine for the treatment of sleep problems in adults with autism spectrum disorder and co-morbid intellectual disability. Journal of psychopharmacology (oxford, england). 2019:269881119864968. doi:10.1177/0269881119864968.
590. Chez M, Kile S, Lepage C, Parise C, Benabides B, Hankins A. A Randomized, Placebo-Controlled, Blinded, Crossover, Pilot Study of the Effects of Dextromethorphan/Quinidine for the Treatment of Neurobehavioral Symptoms in Adults with Autism. Journal of autism and developmental disorders. 2020;50(5):1532–1538.
591. Gabis LV, Ben-Hur R, Shefer S, Jokel A, Shalom DB. Improvement of Language in Children with Autism with Combined Donepezil and Choline Treatment. J. Mol. Neurosci. 2019;69(2):224–234.
592. Herscu P, Handen BL, Arnold LE, et al. The SOFIA Study: Negative Multi-center Study of Low Dose Fluoxetine on Repetitive Behaviors in Children and Adolescents with Autistic Disorder. Journal of autism and developmental disorders. 2020;50(9):3233–3244.
593. Overwater IE, Rietman AB, Mous SE, et al. A randomized controlled trial with everolimus for IQ and autism in tuberous sclerosis complex. Neurology. Conference: 70th Annual Meeting of the American Academy of Neurology, AAN. 2019;93(2):e200-e209.
594. Schroder CM, Malow BA, Maras A, et al. Pediatric Prolonged-Release Melatonin for Sleep in Children with Autism Spectrum Disorder: Impact on Child Behavior and Caregiver's Quality of Life. Journal of autism and developmental disorders. 2019;49(8):3218–3230.
595. Wang Y, Li N, Yang JJ, et al. Probiotics and fructo-oligosaccharide intervention modulate the microbiota-gut brain axis to improve autism spectrum reducing also the hyper-serotonergic state and the dopamine metabolism disorder. Pharmacol Res. 2020;157:104784.
596. Yamasue H, Okada T, Munesue T, et al. Effect of intranasal oxytocin on the core social symptoms of autism spectrum disorder: a randomized clinical trial. Molecular Psychiatry. 2020;25(8):1849–1858.
597. ACTRN12613000334707 (2013): A randomized controlled trial of fish-oil supplementation for children with autism spectrum disorder. Available online at https://www.cochranelibrary.com/central/doi/10.1002/central/CN-01859443/full.
598. ACTRN12618001029280 (2018): Oxytocin in Preschoolers with Autism receiving Social Learning Therapy. Available online at https://www.cochranelibrary.com/central/doi/10.1002/central/CN-01909769/full.
599. Al Olaby, R. R.; Hagerman, R.; Abbeduto, L.; Tassone, F. (2017): Identification of molecular biomarkers predictive of response to targeted treatment in fragile X syndrome and

autism spectrum disorder 61 (9), 828-. Available online at https://www.cochranelibrary.com/central/doi/10.1002/central/CN-01612144/full.
600. Alaerts, K.; Bernaerts, S.; Prinsen, J.; Dillen, C.; Steyaert, J.; Wenderoth, N. (2020): Oxytocin induces long-lasting adaptations within amygdala circuitry in autism: a treatment-mechanism study with randomized placebo-controlled design. In Neuropsychopharmacology. Available online at https://www.cochranelibrary.com/central/doi/10.1002/central/CN-02096262/full.
601. AlOlaby, R. R.; Jiraanont, P.; Durbin-Johnson, B.; Jasoliya, M.; Tang, H-T; Hagerman, R.; Tassone, F. (2020): Molecular Biomarkers Predictive of Sertraline Treatment Response in Young Children With Autism Spectrum Disorder 11. Available online at https://www.cochranelibrary.com/central/doi/10.1002/central/CN-02120407/full.
602. Aman, M. G.; Hollway, J. A.; Veenstra-VanderWeele, J.; Handen, B. L.; Sanders, K. B.; Chan, J. et al. (2018a): Effects of Metformin on Spatial and Verbal Memory in Children with ASD and Overweight Associated with Atypical Antipsychotic Use 28 (4), pp. 266–273. Available online at https://www.cochranelibrary.com/central/doi/10.1002/central/CN-01944358/full.
603. Aman, M. G.; Hollway, J. A.; Veenstra-VanderWeele, J.; Handen, B. L.; Sanders, K. B.; Chan, J. et al. (2018b): Effects of Metformin on Spatial and Verbal Memory in Children with ASD and Overweight Associated with Atypical Antipsychotic Use 28 (4), pp. 266–273. Available online at https://www.cochranelibrary.com/central/doi/10.1002/central/CN-01605463/full.
604. Andari, E.; Duhamel, J-R; Zalla, T.; Herbrecht, E.; Leboyer, M.; Sirigu, A. (2010): Promoting social behavior with oxytocin in high-functioning autism spectrum disorders. In Proceedings of the national academy of sciences of the united states of america 107 (9), pp. 4389–4394. Available online at https://www.cochranelibrary.com/central/doi/10.1002/central/CN-01769394/full.
605. Anonymous (2002): Children with autism may benefit from risperidone. In Pharmaceutical Journal 269 (7210), 184-. Available online at https://www.cochranelibrary.com/central/doi/10.1002/central/CN-01764947/full.
606. Anonymous (2009): Citalopram ineffective for reducing repetitive behavior in autism spectrum disorders 101 (9), 976-. Available online at https://www.cochranelibrary.com/central/doi/10.1002/central/CN-01756156/full.
607. Ayatollahi, A.; Bagheri, S.; Ashraf-Ganjouei, A.; Moradi, K.; Mohammadi, M-R; Akhondzadeh, S. (2020a): Does Pregnenolone Adjunct to Risperidone Ameliorate Irritable Behavior in Adolescents With Autism Spectrum Disorder: a Randomized, Double-Blind, Placebo-Controlled Clinical Trial? In Clinical neuropharmacology. Available online at https://www.cochranelibrary.com/central/doi/10.1002/central/CN-02159272/full.
608. Ayatollahi, A.; Bagheri, S.; Ashraf-Ganjouei, A.; Moradi, K.; Mohammadi, M-R; Akhondzadeh, S. (2020b): Does Pregnenolone Adjunct to Risperidone Ameliorate Irritable Behavior in Adolescents With Autism Spectrum Disorder: a Randomized, Double-Blind, Placebo-Controlled Clinical Trial? 43 (5), pp. 139–145. Available online at https://www.cochranelibrary.com/central/doi/10.1002/central/CN-02177104/full.
609. Ballester, P.; Martínez, M. J.; Inda, M. D.; Javaloyes, A.; Richdale, A. L.; Muriel, J. et al. (2019): Evaluation of agomelatine for the treatment of sleep problems in adults with autism spectrum disorder and co-morbid intellectual disability 33 (11), pp. 1395–1406. Available online at https://www.cochranelibrary.com/central/doi/10.1002/central/CN-01989874/full.
610. Behmanesh, H.; Moghaddam, H. S.; Mohammadi; Akhondzadeh, S. (2019): Risperidone Combination Therapy With Propentofylline for Treatment of Irritability in Autism Spectrum Disorders: a Randomized, Double-Blind, Placebo-Controlled Clinical Trial. In Clin Neuropharmacol 42 (6), pp. 189–196. Available online at https://www.cochranelibrary.com/central/doi/10.1002/central/CN-02005533/full.
611. Bernaerts, S.; Boets, B.; Bosmans, G.; Steyaert, J.; Alaerts, K. (2020): Behavioral effects of multiple-dose oxytocin treatment in autism: a randomized, placebo-controlled trial with long-

term follow-up 11 (1). Available online at https://www.cochranelibrary.com/central/doi/10.1002/central/CN-02075159/full.
612. Bolognani, F.; Del Valle Rubido, M.; Squassante, L.; Wandel, C.; Derks, M.; Murtagh, L. et al. (2019): A phase 2 clinical trial of a vasopressin V1a receptor antagonist shows improved adaptive behaviors in men with autism spectrum disorder 11 (491). Available online at https://www.cochranelibrary.com/central/doi/10.1002/central/CN-01963182/full.
613. Borowiak, K.; Kriegstein, K. von (2020): Intranasal oxytocin modulates brain responses to voice-identity recognition in typically developing individuals, but not in ASD 10 (1). Available online at https://www.cochranelibrary.com/central/doi/10.1002/central/CN-02139252/full.
614. Brito, A. R.; Vairo, GDPT; Dias, APBH; Olej, B.; Nascimento, O. J.M.; Vasconcelos, M. M. (2020): Effect of prednisolone on language function in children with autistic spectrum disorder: a randomized clinical trial. Available online at https://www.cochranelibrary.com/central/doi/10.1002/central/CN-02120696/full.
615. Chez, M.; Kile, S. (2017): A Randomized, placebo-controlled, blinded, crossover, single-center study of the effects of nuedexta in the treatment of neurobehavioral symptoms of adults with Autism spectrum disorder 88 (16). Available online at https://www.cochranelibrary.com/central/doi/10.1002/central/CN-01766847/full.
616. Chez, M.; Kile, S.; Lepage, C.; Parise, C.; Benabides, B.; Hankins, A. (2018): A Randomized, Placebo-Controlled, Blinded, Crossover, Pilot Study of the Effects of Dextromethorphan/Quinidine for the Treatment of Neurobehavioral Symptoms in Adults with Autism. Available online at https://www.cochranelibrary.com/central/doi/10.1002/central/CN-01630105/full.
617. Chez, M.; Kile, S.; Lepage, C.; Parise, C.; Benabides, B.; Hankins, A. (2020): A Randomized, Placebo-Controlled, Blinded, Crossover, Pilot Study of the Effects of Dextromethorphan/Quinidine for the Treatment of Neurobehavioral Symptoms in Adults with Autism 50 (5), pp. 1532–1538. Available online at https://www.cochranelibrary.com/central/doi/10.1002/central/CN-02180353/full.
618. Chez, M. G.; Buchanan, T. M.; Becker, M.; Kessler, J.; Aimonovitch, M. C.; Mrazek, SR (2003): Donepezil hydrochloride: a double-blind study in autistic children 1 (2), pp. 83–88. Available online at https://www.cochranelibrary.com/central/doi/10.1002/central/CN-02106644/full.
619. ChiCTR-IPR-16009627 (2016): A randomized double-blind placebo-controlled trial of the efficiency and mechanism of bumetanide on children with Autism Spectrum Disorder. Available online at https://www.cochranelibrary.com/central/doi/10.1002/central/CN-01812693/full.
620. Dean, O. M.; Gray, K.; Dodd, S.; Villagonzalo, K.; Brown, E.; Tonge, B. et al. (2019): Does N-acetylcysteine improve behaviour in children with autism?: a mixed-methods analysis of the effects of N-acetylcysteine 44 (4), pp. 474–480. Available online at https://www.cochranelibrary.com/central/doi/10.1002/central/CN-02126536/full.
621. DeVane, C. L.; Charles, J. M.; Abramson, R. K.; Williams, J. E.; La Carpenter; Raven, S. et al. (2019): Pharmacotherapy of Autism Spectrum Disorder: results from the Randomized BAART Clinical Trial 39 (6), pp. 626–635. Available online at https://www.cochranelibrary.com/central/doi/10.1002/central/CN-01956993/full.
622. DRKS00008952 (2015): Oxytocin-induced enhancement of Social Skills Training in Adolescents with ASD. Available online at https://www.cochranelibrary.com/central/doi/10.1002/central/CN-01872977/full.
623. DRKS00008984 (2015): A placebo-controlled, double blind, randomised trial with crossover-design investigating the effect of oxytocin nasal spray on neuronal processes of empathy. Available online at https://www.cochranelibrary.com/central/doi/10.1002/central/CN-01873116/full.
624. DRKS00010053 (2016): Pacebo-controlled, double-blind, randomised phase II study with crossover-design investigating the modulatory effects of intranasal Oxytocin on social cognition in

patients with Autism-Spectrum-Disorder. Available online at https://www.cochranelibrary.com/central/doi/10.1002/central/CN-01852938/full.
625. Effects of oxytocin administration on salivary sex hormone levels in autistic and neurotypical women (2020) 11 (1). Available online at https://www.cochranelibrary.com/central/doi/10.1002/central/CN-02147527/full.
626. EUCTR2006-006126-25-FR (2007): Emotional and Social deficits in Asperger syndrome - Asperger and Oxytocin. Available online at https://www.cochranelibrary.com/central/doi/10.1002/central/CN-01822562/full.
627. EUCTR2007-006444-21-ES (2010): EFFECT OF 8-WEEK FATTY ACIDS OMEGA-3 TREATMENT ON OXIDATIVE METABOLISM IN PATIENTS WITH AUTISM SPECTRUM DISORDER: a RANDOMISED DOUBLE-BLIND CROSSOVER PLACEBO-CONTROLLED TRIAL. - Omega-3 tr. Available online at https://www.cochranelibrary.com/central/doi/10.1002/central/CN-01871639/full.
628. EUCTR2008-003712-36-FR (2008): Etude de la réponse clinique et neurofonctionnelle à la fluoxétine dans l'autisme infantile - FAIR. Available online at https://www.cochranelibrary.com/central/doi/10.1002/central/CN-01811517/full.
629. EUCTR2009-010393-38-FR (2009): ETUDE DE L'EFFICACITE D'UN TRAITEMENT PAR BUMETANIDE DANS UNE POPULATION D'ENFANTS AUTISTES. Available online at https://www.cochranelibrary.com/central/doi/10.1002/central/CN-01863271/full.
630. EUCTR2010-018740-13-NL (2010): Short- and long-term effects of oxytocin on empathy and social behaviour in autistic and antisocial male adults. - Oxytocin effects in autistic and antisocial male adults. Available online at https://www.cochranelibrary.com/central/doi/10.1002/central/CN-01821731/full.
631. EUCTR2010-022511-18-DE (2010): Behavioral effects and neural correlates of oxytocin on social attention. Available online at https://www.cochranelibrary.com/central/doi/10.1002/central/CN-01804454/full.
632. EUCTR2010-024202-34-DE (2013): group-therapy, autism and oxytocin - an investigation with the question "Does oxytocin (OT) enhance therapy effects in autism?“. Available online at https://www.cochranelibrary.com/central/doi/10.1002/central/CN-01841327/full.
633. EUCTR2011-003313-42-ES (2011): Agomelatine efficacy of the drug to improve sleep problems in autistic people. Available online at https://www.cochranelibrary.com/central/doi/10.1002/central/CN-01873829/full.
634. EUCTR2012-003750-89-DE (2012): Empathy, Autism and Oxytocin – an investigation by means of functional magnetic resonance imaging and moleculargenetic analyses. Available online at https://www.cochranelibrary.com/central/doi/10.1002/central/CN-01883651/full.
635. EUCTR2013-001230-17-FR (2015): La mélatonine restaure-t-elle l'architecture du sommeil chez les enfants avec autisme ? Etude de phase II. Available online at https://www.cochranelibrary.com/central/doi/10.1002/central/CN-01811030/full.
636. EUCTR2013-003259-39-ES (2014): Study in children and adolescents with autism. Available online at https://www.cochranelibrary.com/central/doi/10.1002/central/CN-01833800/full.
637. EUCTR2014-000586-45-BE (2014): The use of Oxytocin for Autism Spectrum Disorders: investigating the effect on behavior and at the level of the brain. Available online at https://www.cochranelibrary.com/central/doi/10.1002/central/CN-01877991/full.
638. EUCTR2014-001560-35-NL (2016): Bumetanide for Autism Treatment Study. Available online at https://www.cochranelibrary.com/central/doi/10.1002/central/CN-01862639/full.
639. EUCTR2014-003080-38-DE (2014): Glutamatergic medication in the treatment of Obsessive Compulsive Disorder (OCD) and Autism Spectrum Disorder (ASD). Available online at https://www.cochranelibrary.com/central/doi/10.1002/central/CN-01821676/full.
640. EUCTR2015-000955-25-FR (2015): Evaluation of the efficiency of B9 vitamin on the reduction of autistic spectrum symptoms: a pilot study "EFFET. Available online at https://www.cochranelibrary.com/central/doi/10.1002/central/CN-01886957/full.

641. EUCTR2015-001320-31-Outside-EU/EEA (2015): A Study to Evaluate the Efficacy and Safety of Risperidone (R064766) in Children and Adolescents with Irritability Associated with Autistic Disorder. Available online at https://www.cochranelibrary.com/central/doi/10.1002/central/CN-01870569/full.
642. EUCTR2016-000106-11-FR (2016): EVALUATION OF THE EFFICIENCY OF TREATMENT BY BUMETANIDE ON AUTISTIC CHILDREN WITH A KNOWN ETIOLOGY: MULTICENTER AND DOUBLE-BLIND STUDY WITH RANDOMIZED PARALLEL GROUP, AGAINST PLACEBO. Available online at https://www.cochranelibrary.com/central/doi/10.1002/central/CN-01890427/full.
643. Frye, R. E.; Slattery, J.; Delhey, L.; Furgerson, B.; Strickland, T.; Tippett, M. et al. (2018a): Folinic acid improves verbal communication in children with autism and language impairment: a randomized double-blind placebo-controlled trial 23 (2), pp. 247–256. Available online at https://www.cochranelibrary.com/central/doi/10.1002/central/CN-01704696/full.
644. Frye, R. E.; Slattery, J.; Delhey, L.; Furgerson, B.; Strickland, T.; Tippett, M. et al. (2018b): Folinic acid improves verbal communication in children with autism and language impairment: a randomized double-blind placebo-controlled trial 23 (2), pp. 247–256. Available online at https://www.cochranelibrary.com/central/doi/10.1002/central/CN-01461483/full.
645. Gabis, L. V.; Ben-Hur, R.; Shefer, S.; Jokel, A.; Shalom, D. B. (2019): Improvement of Language in Children with Autism with Combined Donepezil and Choline Treatment 69 (2), pp. 224–234. Available online at https://www.cochranelibrary.com/central/doi/10.1002/central/CN-01959307/full.
646. Ghaleiha, A.; Ghyasvand, M.; Mohammadi; Farokhnia, M.; Yadegari, N.; Tabrizi, M. et al. (2014): Galantamine efficacy and tolerability as an augmentative therapy in autistic children: a randomized, double-blind, placebo-controlled trial 28 (7), pp. 677–685. Available online at https://www.cochranelibrary.com/central/doi/10.1002/central/CN-01705551/full.
647. Ghodsi, R.; Kheirouri, S.; Nosrati, R. (2019a): Carnosine supplementation does not affect serum concentrations of advanced glycation and precursors of lipoxidation end products in autism: a randomized controlled clinical trial 56 (1), pp. 148–154. Available online at https://www.cochranelibrary.com/central/doi/10.1002/central/CN-02004956/full.
648. Ghodsi, R.; Kheirouri, S.; Nosrati, R. (2019b): Carnosine supplementation does not affect serum concentrations of advanced glycation and precursors of lipoxidation end products in autism: a randomized controlled clinical trial 56 (1), pp. 148–154. Available online at https://www.cochranelibrary.com/central/doi/10.1002/central/CN-01718671/full.
649. Grimaldi, R.; Gibson, G. R.; Vulevic, J.; Giallourou, N.; Castro-Mejia, J. L.; Hansen, L. H. et al. (2018a): A prebiotic intervention study in children with autism spectrum disorders (ASDs) 6 (1). Available online at https://www.cochranelibrary.com/central/doi/10.1002/central/CN-01629377/full.
650. Grimaldi, R.; Gibson, G. R.; Vulevic, J.; Giallourou, N.; Castro-Mejía, J. L.; Hansen, L. H. et al. (2018b): A prebiotic intervention study in children with autism spectrum disorders (ASDs) 6 (1), p. 133. Available online at https://www.cochranelibrary.com/central/doi/10.1002/central/CN-01703292/full.
651. Hajizadeh-Zaker, R.; Ghajar, A.; Mesgarpour, B.; Afarideh, M.; Mohammadi; Akhondzadeh, S. (2018): l-Carnosine As an Adjunctive Therapy to Risperidone in Children with Autistic Disorder: a Randomized, Double-Blind, Placebo-Controlled Trial 28 (1), pp. 74–81. Available online at https://www.cochranelibrary.com/central/doi/10.1002/central/CN-01461516/full.
652. Hegarty, J. P.; Zamzow, R.; Ferguson, B. J.; Christ, S.; Porges, E.; Johnson, J. D.; Beversdorf, D. Q. (2019): Beta-adrenergic antagonism alters functional connectivity during associative processing in a preliminary study of individuals with and without autism. In Autism. Available online at https://www.cochranelibrary.com/central/doi/10.1002/central/CN-02081462/full.
653. Hendouei, F.; Sanjari Moghaddam, H.; Mohammadi; Taslimi, N.; Rezaei, F.; Akhondzadeh, S. (2019): Resveratrol as adjunctive therapy in treatment of irritability in children with autism: a

double-blind and placebo-controlled randomized trial. Available online at https://www.cochranelibrary.com/central/doi/10.1002/central/CN-02006823/full.
654. Herscu, P.; Handen, B. L.; Le Arnold; Snape, M. F.; Bregman, J. D.; Ginsberg, L. et al. (2020): The SOFIA Study: negative Multi-center Study of Low Dose Fluoxetine on Repetitive Behaviors in Children and Adolescents with Autistic Disorder 50 (9), pp. 3233–3244. Available online at https://www.cochranelibrary.com/central/doi/10.1002/central/CN-01964349/full.
655. Hollander, E.; Uzunova, G.; Taylor, B. P.; Noone, R.; Racine, E.; Doernberg, E. et al. (2018): Randomized crossover feasibility trial of helminthic Trichuris suis ova versus placebo for repetitive behaviors in adult autism spectrum disorder. Available online at https://www.cochranelibrary.com/central/doi/10.1002/central/CN-01747754/full.
656. Hollway, J. A.; Mendoza-Burcham, M.; Andridge, R.; Aman, M. G.; Handen, B.; Le Arnold et al. (2018a): Atomoxetine, Parent Training, and Their Effects on Sleep in Youth with Autism Spectrum Disorder and Attention-Deficit/Hyperactivity Disorder 28 (2), pp. 130–135. Available online at https://www.cochranelibrary.com/central/doi/10.1002/central/CN-01464404/full.
657. Hollway, J. A.; Mendoza-Burcham, M.; Andridge, R.; Aman, M. G.; Handen, B.; Le Arnold et al. (2018b): Atomoxetine, Parent Training, and Their Effects on Sleep in Youth with Autism Spectrum Disorder and Attention-Deficit/Hyperactivity Disorder 28 (2), pp. 130–135. Available online at https://www.cochranelibrary.com/central/doi/10.1002/central/CN-01914038/full.
658. Huffman, G. B. (1997): Fluvoxamine for the treatment of autistic disorders in adults 55 (4), pp. 1375–1376. Available online at https://www.cochranelibrary.com/central/doi/10.1002/central/CN-01759247/full.
659. Ichikawa, H.; Mikami, K.; Okada, T.; Yamashita, Y.; Ishizaki, Y.; Tomoda, A. et al. (2017): Aripiprazole in the Treatment of Irritability in Children and Adolescents with Autism Spectrum Disorder in Japan: a Randomized, Double-blind, Placebo-controlled Study 48 (5), pp. 796–806. Available online at https://www.cochranelibrary.com/central/doi/10.1002/central/CN-01616150/full.
660. IRCT1138901151556N10 (2010): memantine in the treatment of autism. Available online at https://www.cochranelibrary.com/central/doi/10.1002/central/CN-01821582/full.
661. IRCT138711091556N2 (2009): Celecoxib and Autism. Available online at https://www.cochranelibrary.com/central/doi/10.1002/central/CN-01882804/full.
662. IRCT138711161556N7 (2009): Pentoxifylline in the treatment of autism. Available online at https://www.cochranelibrary.com/central/doi/10.1002/central/CN-01835462/full.
663. IRCT138901141556N9 (2010): Tpoiramate in the treatment of autism. Available online at https://www.cochranelibrary.com/central/doi/10.1002/central/CN-01845314/full.
664. IRCT20090117001556N102 (2017): Prednisolone in autism spectrum disorders. Available online at https://www.cochranelibrary.com/central/doi/10.1002/central/CN-01896190/full.
665. IRCT20090117001556N104 (2017): Resveratrol in treatment of autism. Available online at https://www.cochranelibrary.com/central/doi/10.1002/central/CN-01896192/full.
666. IRCT20090117001556N107 (2018): Sulforaphane as adjunctive treatment of irritability in children with Autism spectrum disorder. Available online at https://www.cochranelibrary.com/central/doi/10.1002/central/CN-01896193/full.
667. IRCT201012031556N19 (2010): Ginkgo biloba in the treatment of autistic disorder. Available online at https://www.cochranelibrary.com/central/doi/10.1002/central/CN-01830986/full.
668. IRCT201101105280N3 (2011): Cyproheptadin plus Risperidon in treatment of children with Autistic Disorder: a double blind, placebo controlled study. Available online at https://www.cochranelibrary.com/central/doi/10.1002/central/CN-01814563/full.
669. IRCT201106101556N25 (2011): Amantadine in the treatment of autism. Available online at https://www.cochranelibrary.com/central/doi/10.1002/central/CN-01865016/full.
670. IRCT201106103930N6 (2011): N-Acetylcysteine augmentation with Rispridone in treatment of Autism in children. Available online at https://www.cochranelibrary.com/central/doi/10.1002/central/CN-01833520/full.

671. IRCT201107281556N27 (2011): Riluzole in the treatment of autism. Available online at https://www.cochranelibrary.com/central/doi/10.1002/central/CN-01876275/full.
672. IRCT201108155280N5 (2013): A comparative study on the effectiveness of Risperidone versus Risperidone plus naltrexone in treatment of autistic spectrum disorder in children with 6-12 years old. Available online at https://www.cochranelibrary.com/central/doi/10.1002/central/CN-01835241/full.
673. IRCT201110233930N15 (2011): Aripiprazole versus risperidone for treatment of autism. Available online at https://www.cochranelibrary.com/central/doi/10.1002/central/CN-01818163/full.
674. IRCT201110281556N29 (2011): N-acetyl cysteine in the treatment of autism. Available online at https://www.cochranelibrary.com/central/doi/10.1002/central/CN-01868068/full.
675. IRCT201202281556N37 (2012): pioglitazone in the treatment of autism. Available online at https://www.cochranelibrary.com/central/doi/10.1002/central/CN-01842471/full.
676. IRCT201204037202N5 (2012): Comparing efficacy and side effects of Memantine and Risperidone in treating autistic patients. Available online at https://www.cochranelibrary.com/central/doi/10.1002/central/CN-01805001/full.
677. IRCT201204081556N40 (2012): Galantamine in the treatment of autism. Available online at https://www.cochranelibrary.com/central/doi/10.1002/central/CN-01820250/full.
678. IRCT2012111011421N1 (2013): Effect of omega-3 supplementation on Autistic patients. Available online at https://www.cochranelibrary.com/central/doi/10.1002/central/CN-01798545/full.
679. IRCT201302201556N50 (2013): Minocycline in the treatment of autism. Available online at https://www.cochranelibrary.com/central/doi/10.1002/central/CN-01805755/full.
680. IRCT20131013014994N5 (2018): effect of vitamin D on Autism Spectrum Disorders. Available online at https://www.cochranelibrary.com/central/doi/10.1002/central/CN-01896491/full.
681. IRCT201402043930N33 (2014): short-term co-administration of acid folicfor treating children and adolescents with autism. Available online at https://www.cochranelibrary.com/central/doi/10.1002/central/CN-01821113/full.
682. IRCT201405273930N34 (2014): Vitamin D for treating autism. Available online at https://www.cochranelibrary.com/central/doi/10.1002/central/CN-01838225/full.
683. IRCT20150519022323N2 (2017): The effect of Perceptual motor Exercises along with music and Vitamin D3 Supplementation in children with autism spectrum disorder. Available online at https://www.cochranelibrary.com/central/doi/10.1002/central/CN-01900252/full.
684. IRCT201512081556N83 (2015): L Carnosine in the treatment of Autism. Available online at https://www.cochranelibrary.com/central/doi/10.1002/central/CN-01859089/full.
685. IRCT2015122625699N1 (2016): The effect of Omega-3 on Autism. Available online at https://www.cochranelibrary.com/central/doi/10.1002/central/CN-01838052/full.
686. IRCT201602041556N86 (2016): Simvastatin in the treatment of Autism. Available online at https://www.cochranelibrary.com/central/doi/10.1002/central/CN-01872142/full.
687. IRCT2016022826802N1 (2016): Assessment the efficacy of atomoxetin(stramox) in autism spectrum disorders. Available online at https://www.cochranelibrary.com/central/doi/10.1002/central/CN-01798828/full.
688. IRCT2016061711689N4 (2016): Effects Of Carnosine On Autism. Available online at https://www.cochranelibrary.com/central/doi/10.1002/central/CN-01818759/full.
689. IRCT2016061711689N5 (2016): Effects of carnosine supplementation on autism disorder. Available online at https://www.cochranelibrary.com/central/doi/10.1002/central/CN-01818758/full.
690. IRCT201701131556N95 (2017): Baclofen in the treatment of Autism. Available online at https://www.cochranelibrary.com/central/doi/10.1002/central/CN-01815138/full.

691. IRCT2017013132326N1 (2017): The efficacy of augmentation Flavonoid Quercetin to risperidon in treatment of autism spectrum disorders. Available online at https://www.cochranelibrary.com/central/doi/10.1002/central/CN-01852934/full.
692. IRCT201702171556N96 (2017): Palmitoylethanolamide as adjunctive treatment of Autism: a double blind and placebo controlled trial. Available online at https://www.cochranelibrary.com/central/doi/10.1002/central/CN-01822992/full.
693. IRCT2017041333406N1 (2017): The efficacy of augmentation Donepezil to risperidon in treatment of autism spectrum disorders. Available online at https://www.cochranelibrary.com/central/doi/10.1002/central/CN-01890013/full.
694. ISRCTN04516575 (2011): Investigation of WCFS1 on the gut microbiota of autistic spectrum disorder (ASD) children. Available online at https://www.cochranelibrary.com/central/doi/10.1002/central/CN-01809536/full.
695. ISRCTN54273114 (2010): A Clinical Trial of Levocarnitine to Treat Autism Spectrum Disorders. Available online at https://www.cochranelibrary.com/central/doi/10.1002/central/CN-01873054/full.
696. ISRCTN72571312 (2017): A placebo controlled pilot study to explore the affects of GABA tea on children with autistic spectrum conditions. Available online at https://www.cochranelibrary.com/central/doi/10.1002/central/CN-01893328/full.
697. ISRCTN77884120 (2008): Melatonin treatment for sleep problems in children with autism: a randomised controlled crossover trial. Available online at https://www.cochranelibrary.com/central/doi/10.1002/central/CN-01874369/full.
698. Javadfar, Z.; Abdollahzad, H.; Moludi, J.; Rezaeian, S.; Amirian, H.; Foroughi, A. A. et al. (2020): Effects of vitamin D supplementation on core symptoms, serum serotonin, and interleukin-6 in children with autism spectrum disorders: a randomized clinical trial 79. Available online at https://www.cochranelibrary.com/central/doi/10.1002/central/CN-02175422/full.
699. Jorgensen, M.; Thomsen, P. H.; Henriksen, J. H. (2002): Secretin treatment of autism? 164 (12), 1676-. Available online at https://www.cochranelibrary.com/central/doi/10.1002/central/CN-01712998/full.
700. JPRN-JapicCTI-121862 (2012): A short treatment study of aripiprazole in pediatric patients with Autistic Disorder. Available online at https://www.cochranelibrary.com/central/doi/10.1002/central/CN-01859499/full.
701. JPRN-JMA-IIA00041 (2010): Therapeutic Effect of Dietary Omega-6 and Omega-3 Fatty Acids in Improving Social Impairment in Youth With Autism Spectrum Disorders: a Double-Blind, Randomized, Placebo-Controlled Trial. Available online at https://www.cochranelibrary.com/central/doi/10.1002/central/CN-01806446/full.
702. JPRN-UMIN000002241 (2009): Pilot study searching neural correlates of changes in social impairments induced by intranasal oxytocin administration in subjects with autism spectrum disorder. Available online at https://www.cochranelibrary.com/central/doi/10.1002/central/CN-01810009/full.
703. JPRN-UMIN000004393 (2010): Searching neural correlates of changes in social impairments induced by intranasal oxytocin administration and its association with genotypes related to oxytocin in subjects with autism spectrum disorder. Available online at https://www.cochranelibrary.com/central/doi/10.1002/central/CN-01799807/full.
704. JPRN-UMIN000005211 (2011): A research of therapy evaluation to prosocial behavior after intranasal oxytocin administration. Available online at https://www.cochranelibrary.com/central/doi/10.1002/central/CN-01844520/full.
705. JPRN-UMIN000005809 (2011): A single-blind and crossover study examining the efficacy of intranasal oxytocin administration for social impairments in subjects with pervasive developmental disorders. Available online at https://www.cochranelibrary.com/central/doi/10.1002/central/CN-01840341/full.
706. JPRN-UMIN000007122 (2012): A randomized, double-blind and cross-over trial to examine effects of continuous administration of intranasal oxytocin on social dysfunction in

subjects with autism spectrum disorders. Available online at https://www.cochranelibrary.com/central/doi/10.1002/central/CN-01831328/full.
707. JPRN-UMIN000007250 (2012): A randomized, double-blind, placebo-controlled, cross-over trial of oxytocin in patients with autism spectrum disorder. Available online at https://www.cochranelibrary.com/central/doi/10.1002/central/CN-01831601/full.
708. JPRN-UMIN000009075 (2012): Effects of long-term administration of intranasal oxytocin on autism spectrum disorders. Available online at https://www.cochranelibrary.com/central/doi/10.1002/central/CN-01818604/full.
709. JPRN-UMIN000015264 (2014): A multicenter, parallel group, placebo-controlled, double blind, confirmatory trial of intranasal oxytocin in participants with autism spectrum disorders. Available online at https://www.cochranelibrary.com/central/doi/10.1002/central/CN-01866050/full.
710. JPRN-UMIN000016389 (2015): A research of efficacy and safety of oxytocin administration to detect others altruism in children and adolescents with reactive attachment disorder. Available online at https://www.cochranelibrary.com/central/doi/10.1002/central/CN-01869954/full.
711. JPRN-UMIN000017876 (2015): Effects of long-term administration of intranasal oxytocin in children with autism spectrum disorder. Available online at https://www.cochranelibrary.com/central/doi/10.1002/central/CN-01877181/full.
712. Kanmani, V. K.; Kumar, S.; Doshi, V.; Sivalingam; Nambi, S. (2018): A randomised double blind placebo control study of joint attention, language, social responsiveness, behaviour and epileptic discharge following 8 weeks of levo-carnosine in children with autism spectrum disorder 60 (5), S87-. Available online at https://www.cochranelibrary.com/central/doi/10.1002/central/CN-01468463/full.
713. Khalaj, M.; Saghazadeh, A.; Shirazi, E.; Shalbafan; Alavi, K.; Shooshtari, M. H. et al. (2018a): Palmitoylethanolamide as adjunctive therapy for autism: efficacy and safety results from a randomized controlled trial 103, pp. 104–111. Available online at https://www.cochranelibrary.com/central/doi/10.1002/central/CN-01982422/full.
714. Khalaj, M.; Saghazadeh, A.; Shirazi, E.; Shalbafan, M-R; Alavi, K.; Shooshtari, M. H. et al. (2018b): Palmitoylethanolamide as adjunctive therapy for autism: efficacy and safety results from a randomized controlled trial 103, pp. 104–111. Available online at https://www.cochranelibrary.com/central/doi/10.1002/central/CN-01611928/full.
715. Kong, X-J; Liu, J.; Li, J.; Kwong, K.; Koh, M.; Sukijthamapan, P. et al. (2020): Probiotics and oxytocin nasal spray as neuro-social-behavioral interventions for patients with autism spectrum disorders: a pilot randomized controlled trial protocol 6 (1). Available online at https://www.cochranelibrary.com/central/doi/10.1002/central/CN-02099060/full.
716. Kruppa, J. A.; Gossen, A.; Oberwelland Weiss, E.; Kohls, G.; Grossheinrich, N.; Cholemkery, H. et al. (2018): Neural modulation of social reinforcement learning by intranasal oxytocin in male adults with high-functioning autism spectrum disorder: a randomized trial. Available online at https://www.cochranelibrary.com/central/doi/10.1002/central/CN-01666372/full.
717. Kruppa, J. A.; Gossen, A.; Oberwelland Weiß, E.; Kohls, G.; Großheinrich, N.; Cholemkery, H. et al. (2019): Neural modulation of social reinforcement learning by intranasal oxytocin in male adults with high-functioning autism spectrum disorder: a randomized trial 44 (4), pp. 749–756. Available online at https://www.cochranelibrary.com/central/doi/10.1002/central/CN-02004880/full.
718. Kuriyama, S.; Kamiyama, M.; Watanabe, M.; Tamahashi, S.; Muraguchi, I.; Watanabe, T. et al. (2002): Pyridoxine treatment in a subgroup of children with pervasive developmental disorders 44 (4), pp. 284–286. Available online at https://www.cochranelibrary.com/central/doi/10.1002/central/CN-01742773/full.
719. La Potter; Da Scholze; Biag, H. M.B.; Schneider, A.; Chen, Y.; Nguyen, D. V. et al. (2019): A Randomized Controlled Trial of Sertraline in Young Children With Autism Spectrum Disorder 10.

Available online at https://www.cochranelibrary.com/central/doi/10.1002/central/CN-02049208/full.
720. Le Arnold; Farmer, C.; Kraemer, H. C.; Davies, M.; Witwer, A.; Chuang, S. et al. (2010): Moderators, mediators, and other predictors of risperidone response in children with autistic disorder and irritability 20 (2), pp. 83–93. Available online at https://www.cochranelibrary.com/central/doi/10.1002/central/CN-01779269/full.
721. Le Arnold; Luna, R. A.; Williams, K.; Chan, J.; Parker, R. A.; Wu, Q. et al. (2019): Probiotics for Gastrointestinal Symptoms and Quality of Life in Autism: a Placebo-Controlled Pilot Trial 29 (9), pp. 659–669. Available online at https://www.cochranelibrary.com/central/doi/10.1002/central/CN-01988408/full.
722. Le Arnold; Ober, N.; Aman, M. G.; Handen, B.; Smith, T.; Pan, X. et al. (2018): A 1.5-Year Follow-Up of Parent Training and Atomoxetine for Attention-Deficit/Hyperactivity Disorder Symptoms and Noncompliant/Disruptive Behavior in Autism 28 (5), pp. 322–330. Available online at https://www.cochranelibrary.com/central/doi/10.1002/central/CN-01611566/full.
723. Lecavalier, L.; Pan, X.; Smith, T.; Handen, B. L.; Le Arnold; Silverman, L. et al. (2018): Parent Stress in a Randomized Clinical Trial of Atomoxetine and Parent Training for Children with Autism Spectrum Disorder 48 (4), pp. 980–987. Available online at https://www.cochranelibrary.com/central/doi/10.1002/central/CN-01914087/full.
724. Lefevre, A.; Mottolese, R.; Redoute, J.; Costes, N.; Le Bars, D.; Geoffray, M-M et al. (2018): Oxytocin fails to recruit serotonergic neurotransmission in the autistic brain 28 (12), pp. 4169–4178. Available online at https://www.cochranelibrary.com/central/doi/10.1002/central/CN-01732747/full.
725. Lewis, A. S.; van Schalkwyk, G. I.; Lopez, M. O.; Volkmar, F. R.; Picciotto; Sukhodolsky, D. G. (2018): An Exploratory Trial of Transdermal Nicotine for Aggression and Irritability in Adults with Autism Spectrum Disorder 48 (8), pp. 2748–2757. Available online at https://www.cochranelibrary.com/central/doi/10.1002/central/CN-01952124/full.
726. Liu, Y. W.; Liong, M. T.; Chung, Y. E.; Huang, H. Y.; Peng, W. S.; Cheng, Y. F. et al. (2019): Effects of Lactobacillus plantarum PS128 on Children with Autism Spectrum Disorder in Taiwan: a Randomized, Double-Blind, Placebo-Controlled Trial 11 (4). Available online at https://www.cochranelibrary.com/central/doi/10.1002/central/CN-01940122/full.
727. Mahdavinasab, S. M.; Saghazadeh, A.; Motamed-Gorji, N.; Vaseghi, S.; Mohammadi; Alichani, R.; Akhondzadeh, S. (2019): Baclofen as an adjuvant therapy for autism: a randomized, double-blind, placebo-controlled trial 28 (12), pp. 1619–1628. Available online at https://www.cochranelibrary.com/central/doi/10.1002/central/CN-01940282/full.
728. Malek, M.; Ashraf-Ganjouei, A.; Moradi, K.; Bagheri, S.; Mohammadi, M-R; Akhondzadeh, S. (2020): Prednisolone as Adjunctive Treatment to Risperidone in Children With Regressive Type of Autism Spectrum Disorder: a Randomized, Placebo-Controlled Trial 43 (2), pp. 39–45. Available online at https://www.cochranelibrary.com/central/doi/10.1002/central/CN-02099690/full.
729. Malow, Beth A.; Findling, Robert L.; Schroder, Carmen M.; Maras, Athanasios; Breddy, John; Nir, Tali et al. (2020): Sleep, Growth, and Puberty After Two Years of Prolonged-Release Melatonin in Children With Autism Spectrum Disorder. In Journal of the American Academy of Child and Adolescent Psychiatry. Available online at https://www.cochranelibrary.com/central/doi/10.1002/central/CN-02129270/full.
730. Marcus, R. N.; Owen, R.; Manos, G.; Mankoski, R.; Kamen, L.; McQuade, R. D. et al. (2011): Aripiprazole in the treatment of irritability in pediatric patients (Aged 6-17 Years) with autistic disorder: results from a 52-week, open-label study 21 (3), pp. 229–236. Available online at https://www.cochranelibrary.com/central/doi/10.1002/central/CN-01776074/full.
731. Mazahery, H.; Conlon, C. A.; Beck, K. L.; Mugridge, O.; Kruger, M. C.; Stonehouse, W. et al. (2018): A randomised controlled trial of vitamin D and omega-3 long chain polyunsaturated fatty acids in the treatment of irritability and hyperactivity among children with autism spectrum disorder. Available online at https://www.cochranelibrary.com/central/doi/10.1002/central/CN-01667971/full.

732. Mazahery, H.; Conlon, C. A.; Beck, K. L.; Mugridge, O.; Kruger, M. C.; Stonehouse, W. et al. (2019a): A randomised controlled trial of vitamin D and omega-3 long chain polyunsaturated fatty acids in the treatment of irritability and hyperactivity among children with autism spectrum disorder 187, pp. 9–16. Available online at https://www.cochranelibrary.com/central/doi/10.1002/central/CN-01943800/full.
733. Mazahery, H.; Conlon, C. A.; Beck, K. L.; Mugridge, O.; Kruger, M. C.; Stonehouse, W. et al. (2019b): A Randomised-Controlled Trial of Vitamin D and Omega-3 Long Chain Polyunsaturated Fatty Acids in the Treatment of Core Symptoms of Autism Spectrum Disorder in Children. Available online at https://www.cochranelibrary.com/central/doi/10.1002/central/CN-01793905/full.
734. Mazahery, H.; Conlon, C. A.; Beck, K. L.; Mugridge, O.; Kruger, M. C.; Stonehouse, W. et al. (2019c): A Randomised-Controlled Trial of Vitamin D and Omega-3 Long Chain Polyunsaturated Fatty Acids in the Treatment of Core Symptoms of Autism Spectrum Disorder in Children 49 (5), pp. 1778–1794. Available online at https://www.cochranelibrary.com/central/doi/10.1002/central/CN-01943861/full.
735. Mazahery, H.; Conlon, C. A.; Beck, K. L.; Mugridge, O.; Kruger, M. C.; Stonehouse, W. et al. (2020): Inflammation (IL-1β) Modifies the Effect of Vitamin D and Omega-3 Long Chain Polyunsaturated Fatty Acids on Core Symptoms of Autism Spectrum Disorder-An Exploratory Pilot Study‡ 12 (3). Available online at https://www.cochranelibrary.com/central/doi/10.1002/central/CN-02099822/full.
736. Mehrazad-Saber, Z.; Kheirouri, S.; Noorazar, S. G. (2018): Effects of l-Carnosine Supplementation on Sleep Disorders and Disease Severity in Autistic Children: a Randomized, Controlled Clinical Trial 123 (1), pp. 72–77. Available online at https://www.cochranelibrary.com/central/doi/10.1002/central/CN-01643031/full.
737. Michael, G. Aman; Jill, A. Hollway; Christopher, J. McDougle; Lawrence, Scahill; Elaine, Tierney; James, T. McCracken et al. (2008): Cognitive Effects of Risperidone in Children with Autism and Irritable Behavior 18 (3), pp. 227–236. Available online at https://www.cochranelibrary.com/central/doi/10.1002/central/CN-02117883/full.
738. Moazen-Zadeh, E.; Shirzad, F.; Karkhaneh-Yousefi, M. A.; Khezri, R.; Mohammadi; Akhondzadeh, S. (2018a): Simvastatin as an Adjunctive Therapy to Risperidone in Treatment of Autism: a Randomized, Double-Blind, Placebo-Controlled Clinical Trial 28 (1), pp. 82–89. Available online at https://www.cochranelibrary.com/central/doi/10.1002/central/CN-01914161/full.
739. Moazen-Zadeh, E.; Shirzad, F.; Karkhaneh-Yousefi, M-A; Khezri, R.; Mohammadi, M-R; Akhondzadeh, S. (2018b): Simvastatin as an Adjunctive Therapy to Risperidone in Treatment of Autism: a Randomized, Double-Blind, Placebo-Controlled Clinical Trial 28 (1), pp. 82–89. Available online at https://www.cochranelibrary.com/central/doi/10.1002/central/CN-01461514/full.
740. Momtazmanesh, S.; Amirimoghaddam-Yazdi, Z.; Moghaddam, H. S.; Mohammadi; Akhondzadeh, S. (2020): Sulforaphane as an adjunctive treatment for irritability in children with autism spectrum disorder: a randomized, double-blind, placebo-controlled clinical trial 74 (7), pp. 398–405. Available online at https://www.cochranelibrary.com/central/doi/10.1002/central/CN-02132115/full.
741. Moorthy, M. P.; Srinivasan, A. V.; Bhanu, K.; Mugundan, K.; Sivakumar, S. (2018): L-Carnosine in pediatric cognitive disorders (4), pp. 372–373. Available online at https://www.cochranelibrary.com/central/doi/10.1002/central/CN-01616735/full.
742. Moradi, H.; Sohrabi, M.; Taheri, H.; Khodashenas, E.; Movahedi, A. (2018a): Comparison of the effects of perceptual-motor exercises, vitamin D supplementation and the combination of these interventions on decreasing stereotypical behavior in children with autism disorder. Available online at https://www.cochranelibrary.com/central/doi/10.1002/central/CN-01727093/full.

743. Moradi, H.; Sohrabi, M.; Taheri, H.; Khodashenas, E.; Movahedi, A. (2018b): The effects of different combinations of perceptual-motor exercises, music, and vitamin D supplementation on the nerve growth factor in children with high-functioning autism 31, pp. 139–145. Available online at https://www.cochranelibrary.com/central/doi/10.1002/central/CN-02198454/full.
744. NCT00005014 (2000): Treatment of Autism in Children and Adolescents. Available online at https://www.cochranelibrary.com/central/doi/10.1002/central/CN-02024836/full.
745. NCT00086645 (2004): Citalopram for Children With Autism and Repetitive Behavior (STAART Study 1). Available online at https://www.cochranelibrary.com/central/doi/10.1002/central/CN-02035262/full.
746. NCT00332241 (2006): Study of Aripiprazole in the Treatment of Children and Adolescents With Autistic Disorder (AD). Available online at https://www.cochranelibrary.com/central/doi/10.1002/central/CN-02023518/full.
747. NCT00337571 (2006): Study of Aripiprazole in the Treatment of Children and Adolescents With Autistic Disorder (AD). Available online at https://www.cochranelibrary.com/central/doi/10.1002/central/CN-02023519/full.
748. NCT00380692 (2006): Atomoxetine Versus Placebo for Symptoms of Attention-Deficit/Hyperactivity Disorder (ADHD) in Children and Adolescents With Autism Spectrum Disorder. Available online at https://www.cochranelibrary.com/central/doi/10.1002/central/CN-02016359/full.
749. NCT00453180 (2007): A Study of Oral N-Acetylcysteine in Children With Autism Spectrum Disorders. Available online at https://www.cochranelibrary.com/central/doi/10.1002/central/CN-02036459/full.
750. NCT00576732 (2007): A Study of the Effectiveness and Safety of Two Doses of Risperidone in the Treatment of Children and Adolescents With Autistic Disorder. Available online at https://www.cochranelibrary.com/central/doi/10.1002/central/CN-02024908/full.
751. NCT00627705 (2008): A Study of N-Acetyl Cysteine in Children With Autism. Available online at https://www.cochranelibrary.com/central/doi/10.1002/central/CN-02035806/full.
752. NCT00786799 (2008): Omega-3 Fatty Acids for Autism Treatment. Available online at https://www.cochranelibrary.com/central/doi/10.1002/central/CN-02020259/full.
753. NCT00844753 (2008): Atomoxetine, Placebo and Parent Management Training in Autism. Available online at https://www.cochranelibrary.com/central/doi/10.1002/central/CN-02030524/full.
754. NCT00850070 (2009): Sapropterin as a Treatment for Autistic Disorder. Available online at https://www.cochranelibrary.com/central/doi/10.1002/central/CN-02040270/full.
755. NCT00873509 (2009): Buspirone in the Treatment of 2-6 Year Old Children With Autistic Disorder. Available online at https://www.cochranelibrary.com/central/doi/10.1002/central/CN-02032490/full.
756. NCT01039792 (2009): Trial of Methyl B12 on Behavioral and Metabolic Measures in Children With Autism. Available online at https://www.cochranelibrary.com/central/doi/10.1002/central/CN-02034295/full.
757. NCT01078714 (2010): Efficiency of Bumetanide in Autistic Children. Available online at https://www.cochranelibrary.com/central/doi/10.1002/central/CN-02018877/full.
758. NCT01225198 (2010): Vitamin/Mineral Supplement for Children and Adults With Autism. Available online at https://www.cochranelibrary.com/central/doi/10.1002/central/CN-02016929/full.
759. NCT01333072 (2011): Biomarkers in Autism of Aripiprazole and Risperidone Treatment (BAART). Available online at https://www.cochranelibrary.com/central/doi/10.1002/central/CN-02042123/full.
760. NCT01602016 (2012): A Folinic Acid Intervention for Autism Spectrum Disorders. Available online at https://www.cochranelibrary.com/central/doi/10.1002/central/CN-02037837/full.

761. NCT01694667 (2012): Omega-3 Fatty Acids for Hyperactivity Treatment in Autism Spectrum Disorder. Available online at https://www.cochranelibrary.com/central/doi/10.1002/central/CN-02039540/full.
762. NCT01962870 (2013): The Role of Vasopressin in the Social Deficits of Autism. Available online at https://www.cochranelibrary.com/central/doi/10.1002/central/CN-02045000/full.
763. NCT02081027 (2013): Pilot Study of Riluzole for Drug-Refractory Irritability in Autism Spectrum Disorders. Available online at https://www.cochranelibrary.com/central/doi/10.1002/central/CN-02035904/full.
764. NCT02508922 (2015): Trial of Vitamin D3 Supplementation in Paediatric Autism. Available online at https://www.cochranelibrary.com/central/doi/10.1002/central/CN-02033528/full.
765. NCT02552147 (2015): Nicotinic Cholinergic Modulation as a Novel Treatment Strategy for Aggression Associated With Autism. Available online at https://www.cochranelibrary.com/central/doi/10.1002/central/CN-02042829/full.
766. NCT02561481 (2015): Sulforaphane Treatment of Children With Autism Spectrum Disorder (ASD). Available online at https://www.cochranelibrary.com/central/doi/10.1002/central/CN-02046692/full.
767. NCT02708901 (2016): Gut to Brain Interaction in Autism. Role of Probiotics on Clinical, Biochemical and Neurophysiological Parameters. Available online at https://www.cochranelibrary.com/central/doi/10.1002/central/CN-02043821/full.
768. NCT02720900 (2016): Prebiotic Intervention for Autism Spectrum Disorders. Available online at https://www.cochranelibrary.com/central/doi/10.1002/central/CN-02039522/full.
769. NCT03434366 (2018): Intranasal Ketamine With Dexmedetomidine for the Treatment of Children With Autism Spectrum Disorder. Available online at https://www.cochranelibrary.com/central/doi/10.1002/central/CN-01593409/full.
770. NCT03466671 (2018): A Trial of TTA-121 on Autism Spectrum Disorder. Available online at https://www.cochranelibrary.com/central/doi/10.1002/central/CN-01589711/full.
771. NCT03487770 (2018): Aripiprazole Oral Solution in the Treatment of Children and Adolescents With Autistic Disorder. Available online at https://www.cochranelibrary.com/central/doi/10.1002/central/CN-01567792/full.
772. NCT03514784 (2018): Combination Probiotic: BB-12 With LGG (Different Doses) in Treating Children With Autism Spectrum Disorder. Available online at https://www.cochranelibrary.com/central/doi/10.1002/central/CN-01574101/full.
773. NCT03537950 (2018): Shifting Brain Excitation-Inhibition Balance in Autism Spectrum Disorder. Available online at https://www.cochranelibrary.com/central/doi/10.1002/central/CN-01659868/full.
774. NCT03550209 (2018): Fatty Acid Supplementation in Children With ASD. Available online at https://www.cochranelibrary.com/central/doi/10.1002/central/CN-01660160/full.
775. NCT03553875 (2018): Memantine for the Treatment of Social Deficits in Youth With Disorders of Impaired Social Interactions. Available online at https://www.cochranelibrary.com/central/doi/10.1002/central/CN-01609193/full.
776. Niederhofer, H. (2004): Venlafaxine has modest effects in autistic children 1 (1), pp. 87–90. Available online at https://www.cochranelibrary.com/central/doi/10.1002/central/CN-01764682/full.
777. NTR294 (2005): Risperidone in Children and Adolescents with severe disruptive behavior problems. Available online at https://www.cochranelibrary.com/central/doi/10.1002/central/CN-01826301/full.
778. NTR6325 (2017): Bumetanide for the Autism Spectrum Clinical Effectiveness Trial. Available online at https://www.cochranelibrary.com/central/doi/10.1002/central/CN-01885359/full.
779. Overwater, I. E.; Rietman, AB; Mous, S. E.; Bindels-de Heus, K.; Rizopoulos, D.; Hoopen, L. W. ten et al. (2019): A randomized controlled trial with everolimus for IQ and autism in tuberous

sclerosis complex 93 (2), e200-e209. Available online at https://www.cochranelibrary.com/central/doi/10.1002/central/CN-01960058/full.
780. Owada, K.; Okada, T.; Munesue, T.; Kuroda, M.; Fujioka, T.; Uno, Y. et al. (2019a): Quantitative facial expression analysis revealed the efficacy and time course of oxytocin in autism. In Brain. Available online at https://www.cochranelibrary.com/central/doi/10.1002/central/CN-02010761/full.
781. Owada, K.; Okada, T.; Munesue, T.; Kuroda, M.; Fujioka, T.; Uno, Y. et al. (2019b): Quantitative facial expression analysis revealed the efficacy and time course of oxytocin in autism 142 (7), pp. 2127–2136. Available online at https://www.cochranelibrary.com/central/doi/10.1002/central/CN-01986455/full.
782. Parellada, M.; Llorente, C.; Calvo, R.; Gutierrez, S.; Lázaro, L.; Graell, M. et al. (2017): Randomized trial of omega-3 for autism spectrum disorders: effect on cell membrane composition and behavior 27 (12), pp. 1319–1330. Available online at https://www.cochranelibrary.com/central/doi/10.1002/central/CN-01615399/full.
783. Parker, K. J.; Oztan, O.; Libove, R. A.; Mohsin, N.; Karhson, D. S.; Sumiyoshi, R. D. et al. (2019): A randomized placebo-controlled pilot trial shows that intranasal vasopressin improves social deficits in children with autism 11 (491). Available online at https://www.cochranelibrary.com/central/doi/10.1002/central/CN-01963183/full.
784. Parracho, HMRT; Gibson, G. R.; Knott, F.; Bosscher, D.; Kleerebezem, M.; McCartney, A. L. (2010): A double-blind, placebo-controlled, crossover-designed probiotic feeding study in children diagnosed with autistic spectrum disorders 5 (2), pp. 69–74. Available online at https://www.cochranelibrary.com/central/doi/10.1002/central/CN-01756686/full.
785. Pearson, D.; Santos, C. W.; Aman, M.; Arnold, L. E.; Lane, D. M.; Loveland KA et al. (2020): Effects of Extended-Release Methylphenidate Treatment on Cognitive Task Performance in Children with Autism Spectrum Disorder and Attention-Deficit/Hyperactivity Disorder. In J Child Adolesch Psychopharmacol. Available online at https://www.cochranelibrary.com/central/doi/10.1002/central/CN-02182538/full.
786. Pearson DA; Santos CW; Aman MG; Arnold LE; Lane DM; Loveland KA et al. (2020): Effects of Extended-Release Methylphenidate Treatment on Cognitive Task Performance in Children with Autism Spectrum Disorder and Attention-Deficit/Hyperactivity Disorder. In Journal of Child & Adolescent Psychopharmacology. Available online at https://pubmed.ncbi.nlm.nih.gov/32644833/.
787. Petryk, S. (2004): In children with autism, is intravenous secretin more effective than placebo in improving social skills, communication, behaviour or global functioning? Part A: evidence-based answer and summary 9 (4), pp. 244–245. Available online at https://www.cochranelibrary.com/central/doi/10.1002/central/CN-01770710/full.
788. Politte, L. C.; Scahill, L.; Figueroa, J.; McCracken, J. T.; King, B.; McDougle, C. J. (2018a): A randomized, placebo-controlled trial of extended-release guanfacine in children with autism spectrum disorder and ADHD symptoms: an analysis of secondary outcome measures, pp. 1–7. Available online at https://www.cochranelibrary.com/central/doi/10.1002/central/CN-01464247/full.
789. Politte, L. C.; Scahill, L.; Figueroa, J.; McCracken, J. T.; King, B.; McDougle, C. J. (2018b): A randomized, placebo-controlled trial of extended-release guanfacine in children with autism spectrum disorder and ADHD symptoms: an analysis of secondary outcome measures 43 (8), pp. 1772–1778. Available online at https://www.cochranelibrary.com/central/doi/10.1002/central/CN-01703813/full.
790. Pretzsch, C. M.; Freyberg, J.; Voinescu, B.; Lythgoe, D.; Horder, J.; Mendez, M. A. et al. (2019a): Effects of cannabidiol on brain excitation and inhibition systems; a randomised placebo-controlled single dose trial during magnetic resonance spectroscopy in adults with and without autism spectrum disorder. Available online at https://www.cochranelibrary.com/central/doi/10.1002/central/CN-01793875/full.

791. Pretzsch, C. M.; Freyberg, J.; Voinescu, B.; Lythgoe, D.; Horder, J.; Mendez, M. A. et al. (2019b): Effects of cannabidiol on brain excitation and inhibition systems; a randomised placebo-controlled single dose trial during magnetic resonance spectroscopy in adults with and without autism spectrum disorder 44 (8), pp. 1398–1405. Available online at https://www.cochranelibrary.com/central/doi/10.1002/central/CN-02090329/full.
792. Pretzsch, C. M.; Voinescu, B.; Lythgoe, D.; Horder, J.; Mendez, M. A.; Wichers, R. et al. (2019c): Effects of cannabidivarin (CBDV) on brain excitation and inhibition systems in adults with and without Autism Spectrum Disorder (ASD): a single dose trial during magnetic resonance spectroscopy 9 (1), p. 313. Available online at https://www.cochranelibrary.com/central/doi/10.1002/central/CN-02008387/full.
793. Pretzsch, C. M.; Voinescu, B.; Mendez, M. A.; Wichers, R.; Ajram, L.; Ivin, G. et al. (2019d): The effect of cannabidiol (CBD) on low-frequency activity and functional connectivity in the brain of adults with and without autism spectrum disorder (ASD) 33 (9), pp. 1141–1148. Available online at https://www.cochranelibrary.com/central/doi/10.1002/central/CN-01993910/full.
794. Quintana, D. S.; Westlye, L. T.; Hope, S.; Naerland, T.; Smerud, K. T.; Mahmoud, R. A. et al. (2017): Dose-dependent social-cognitive effects of intranasal oxytocin delivered with novel breath powered device in adults with autism spectrum disorder: a randomized placebo-controlled double-blind crossover trial 29, pp. 18–19. Available online at https://www.cochranelibrary.com/central/doi/10.1002/central/CN-01934176/full.
795. Rahman, A.; Freedman, R.; Hollander, E. (2018): Alpha-7 nicotinic acetylcholine receptor positive allosteric modulator galantamine in autism spectrum disorder 83 (9), S369-S370. Available online at https://www.cochranelibrary.com/central/doi/10.1002/central/CN-01608272/full.
796. Reddihough, D.; Marraffa, C.; Mouti, A.; O'Sullivan, M.; Lee, K. J.; Orsini, F. et al. (2019a): A randomised placebo-controlled trial to determine if fluoxetine is effective for improving autistic behaviours 55, pp. 8–9. Available online at https://www.cochranelibrary.com/central/doi/10.1002/central/CN-01956407/full.
797. Reddihough, D. S.; Marraffa, C.; Mouti, A.; O'Sullivan, M.; Lee, K. J.; Orsini, F. et al. (2019b): Effect of Fluoxetine on Obsessive-Compulsive Behaviors in Children and Adolescents With Autism Spectrum Disorders: a Randomized Clinical Trial 322 (16), pp. 1561–1569. Available online at https://www.cochranelibrary.com/central/doi/10.1002/central/CN-01999818/full.
798. Renard, E.; Leheup, B.; Gueant-Rodriguez, R-M; Oussalah, A.; Quadros, E. V.; Gueant, J-L (2020): Folinic acid improves the score of Autism in the EFFET placebo-controlled randomized trial 173, pp. 57–61. Available online at https://www.cochranelibrary.com/central/doi/10.1002/central/CN-02122828/full.
799. am Reynolds; Connolly, H. V.; Katz, T.; Goldman, S. E.; Weiss, S. K.; Halbower, A. C. et al. (2020): Randomized, Placebo-Controlled Trial of Ferrous Sulfate to Treat Insomnia in Children With Autism Spectrum Disorders. Available online at https://www.cochranelibrary.com/central/doi/10.1002/central/CN-02073713/full.
800. Roberts, T. P.L.; Bloy, L.; Blaskey, L.; Kuschner, E.; Gaetz, L.; Anwar, A. et al. (2019): A MEG Study of Acute Arbaclofen (STX-209) Administration 13. Available online at https://www.cochranelibrary.com/central/doi/10.1002/central/CN-02075743/full.
801. Saad, K.; Abdel-Rahman, A.; Elserogy, Y.; Al-Atram, A.; El-Houfey, A.; Othman, H. et al. (2019a): Retraction: randomized controlled trial of vitamin D supplementation in children with autism spectrum disorder 60 (6), 711-. Available online at https://www.cochranelibrary.com/central/doi/10.1002/central/CN-02003424/full.
802. Saad, K.; Abdel-Rahman, A. A.; Elserogy, Y. M.; Al-Atram, A. A.; El-Houfey, A. A.; Othman, H. A. et al. (2018): Randomized controlled trial of vitamin D supplementation in children with autism spectrum disorder 59 (1), pp. 20–29. Available online at https://www.cochranelibrary.com/central/doi/10.1002/central/CN-01622375/full.
803. Saad, K.; Abdel-Rahman, A.; Elserogy, Y.; Al-Atram, A.; El-Houfey, A.; Othman, H. et al. (2019b): Retraction: randomized controlled trial of vitamin D supplementation in children with

autism spectrum disorder…Saad k, Rahman A, Elserogy Y, et al. Randomized controlled trial of vitamin D supplementation in children with autism spectrum disorder, J CHILD PSYCHOL PSYCHIATRY, Jan 2018; 59 (1): 20-29. (10p) 60 (6), p. 711. Available online at https://www.cochranelibrary.com/central/doi/10.1002/central/CN-02116723/full.
804. Saad, Khaled (2018): Response to letters: randomized controlled trial of vitamin D supplementation in children with autism spectrum disorder - correction and additional information 59 (1), e3-e5. Available online at https://www.cochranelibrary.com/central/doi/10.1002/central/CN-01925876/full.
805. Santocchi, E.; Guiducci, L.; Prosperi, M.; Calderoni, S.; Gaggini, M.; Apicella, F. et al. (2020): Effects of Probiotic Supplementation on Gastrointestinal, Sensory and Core Symptoms in Autism Spectrum Disorders: a Randomized Controlled Trial 11. Available online at https://www.cochranelibrary.com/central/doi/10.1002/central/CN-02192717/full.
806. SLCTR/2009/006 (2009): Effectiveness of Omega-3 and Omega-6 in childhood behaviour disorders. Available online at https://www.cochranelibrary.com/central/doi/10.1002/central/CN-01860608/full.
807. Spanos M; Chandrasekhar T; Kim SJ; Hamer RM; King BH; McDougle CJ et al. (2020a): Rationale, design, and methods of the autism centers of excellence (ACE) network study of oxytocin in autism to improve reciprocal social behaviors (SOARS-B). In Contemporary clinical trials, 106103-. Available online at https://www.cochranelibrary.com/central/doi/10.1002/central/CN-02183313/full.
808. Spanos M; Chandrasekhar T; Kim SJ; Hamer RM; King BH; McDougle CJ et al. (2020b): Rationale, design, and methods of the Autism Centers of Excellence (ACE) network Study of Oxytocin in Autism to improve Reciprocal Social Behaviors (SOARS-B). In Contemporary clinical trials 98. Available online at https://www.cochranelibrary.com/central/doi/10.1002/central/CN-02207105/full.
809. Sprengers, J. J.; van Andel, D. M.; Zuithoff, N. P.; Keijzer-Veen, M. G.; Schulp, A. J.; Scheepers, F. E. et al. (2020): Bumetanide for Core Symptoms of Autism Spectrum Disorder (BAMBI): a Single Center, Double-Blinded, Participant-Randomized, Placebo-Controlled, Phase Two, Superiority Trial. Available online at https://www.cochranelibrary.com/central/doi/10.1002/central/CN-02160281/full.
810. Stivaros, S.; Garg, S.; Tziraki, M.; Cai, Y.; Thomas, O.; Mellor, J. et al. (2018): Randomised controlled trial of simvastatin treatment for autism in young children with neurofibromatosis type 1 (SANTA) 9, p. 12. Available online at https://www.cochranelibrary.com/central/doi/10.1002/central/CN-01656011/full.
811. Strathearn, L.; Kim, S.; Da Bastian; Jung, J.; Iyengar, U.; Martinez, S. et al. (2018): Visual systemizing preference in children with autism: a randomized controlled trial of intranasal oxytocin 30 (2), pp. 511–521. Available online at https://www.cochranelibrary.com/central/doi/10.1002/central/CN-01649967/full.
812. TCTR20180414001 (2018): Efficacy and adverse drug reaction between Risperidone solution local made and original drug in treatment of autism spectrum disorders. Available online at https://www.cochranelibrary.com/central/doi/10.1002/central/CN-01899324/full.
813. Wang, Ying; Li, Ning; Yang, Jun-Jie; Zhao, Dong-Mei; Chen, Bin; Zhang, Guo-Qing et al. (2020): Probiotics and fructo-oligosaccharide intervention modulate the microbiota-gut brain axis to improve autism spectrum reducing also the hyper-serotonergic state and the dopamine metabolism disorder. In Pharmacological research 157. Available online at https://www.cochranelibrary.com/central/doi/10.1002/central/CN-02164416/full.
814. Wink, L. K.; Adams, R.; Horn, P. S.; Tessier, C. R.; Bantel, A. P.; Hong, M. et al. (2018): A Randomized Placebo-Controlled Cross-Over Pilot Study of Riluzole for Drug-Refractory Irritability in Autism Spectrum Disorder 48 (9), pp. 3051–3060. Available online at https://www.cochranelibrary.com/central/doi/10.1002/central/CN-01629225/full.
815. Wink, L. K.; Reisinger, D. L.; Horn, P.; Shaffer, R. C.; O'Brien, K.; Schmitt, L. et al. (2020): Brief Report: intranasal Ketamine in Adolescents and Young Adults with Autism Spectrum

Disorder—Initial Results of a Randomized, Controlled, Crossover, Pilot Study. Available online at https://www.cochranelibrary.com/central/doi/10.1002/central/CN-02144333/full.
816. Wong, N. M.L.; Findon, J. L.; Wichers, R. H.; Giampietro, V.; Stoencheva, V.; Murphy, C. M. et al. (2020): Serotonin differentially modulates the temporal dynamics of the limbic response to facial emotions in male adults with and without autism spectrum disorder (ASD): a randomised placebo-controlled single-dose crossover trial. Available online at https://www.cochranelibrary.com/central/doi/10.1002/central/CN-02123596/full.
817. Yamasue, H.; Okada, T.; Munesue, T.; Kuroda, M.; Fujioka, T.; Uno, Y. et al. (2018): Effect of intranasal oxytocin on the core social symptoms of autism spectrum disorder: a randomized clinical trial, pp. 1–10. Available online at https://www.cochranelibrary.com/central/doi/10.1002/central/CN-01619072/full.
818. Zimmerman, A.; Diggins, E.; Connors, S.; Singh, K. (2018): Sulforaphane treatment of children with autism spectrum disorder (ASD) - A progress report 90 (15). Available online at https://www.cochranelibrary.com/central/doi/10.1002/central/CN-01608794/full.
819. ACTRN12616001078448 (2016): A trial to compare the quality of induction using two different premedication combinations in children with Autism Spectrum Disorder (ASD) undergoing surgery. Available online at https://www.cochranelibrary.com/central/doi/10.1002/central/CN-01821604/full.
820. EUCTR2018-000769-35-BE (2018): The use of Oxytocin for Autism Spectrum Disorders: investigating the effect on behavior and at the level of the brain. Available online at https://www.cochranelibrary.com/central/doi/10.1002/central/CN-01906150/full.
821. NCT03538431 (2018): Improving Driving in Young People With Autism Spectrum Disorders. Available online at https://www.cochranelibrary.com/central/doi/10.1002/central/CN-01659883/full.
822. NCT03594552 (2018): Modulation of the Brain Excitatory/Inhibitory (E/I) Balance in Autism Spectrum Disorder (ASD). Available online at https://www.cochranelibrary.com/central/doi/10.1002/central/CN-01661305/full.
823. NCT03640156 (2018): Modulating Socially Adaptive Mirror System Functioning in Autism by Oxytocin. Available online at https://www.cochranelibrary.com/central/doi/10.1002/central/CN-01662450/full.
824. NCT03678129 (2018): GABA Pathways in Autism Spectrum Disorder (ASD). Available online at https://www.cochranelibrary.com/central/doi/10.1002/central/CN-01663437/full.
825. NCT03826940 (2019): From Molecules to Cognition: inhibitory Mechanisms in ASD and NF1. Available online at https://www.cochranelibrary.com/central/doi/10.1002/central/CN-01919013/full.
826. NCT04053036 (2019): Effects of Drugs on Responses to Brain and Emotional Processes. Available online at https://www.cochranelibrary.com/central/doi/10.1002/central/CN-01954403/full.
827. NCT04145076 (2019): Brain Response to Serotonergic Medications in ASD. Available online at https://www.cochranelibrary.com/central/doi/10.1002/central/CN-02001515/full.
828. NCT04270708 (2020): Intranasal Dexmedetomidine vs Oral Triclofos Sodium for EEG in Children With Autism. Available online at https://www.cochranelibrary.com/central/doi/10.1002/central/CN-02088187/full.
829. NCT04278898 (2020): The Neurobiology of Restricted and Repetitive Behaviors in Children With Autism Using N-acetylcysteine. Available online at https://www.cochranelibrary.com/central/doi/10.1002/central/CN-02088373/full.
830. P.206 Excitatory-inhibitory neurochemical response to GABA-B receptor challenge is different in adults with and without an autism spectrum condition (2019). In European neuropsychopharmacology 29, S159-. Available online at https://www.cochranelibrary.com/central/doi/10.1002/central/CN-02136237/full.
831. RBR-8cq282; Rudimar dos Santos Riesgo - Porto Alegre, R. S. Brazil; Gilberto Schwartsmann - Porto Alegre, R. S. Brazil; de Pós-Graduação em Saúde da Criança e

Adolescente - Universidade Federal do Rio Gr, Programa; e do Sul - Porto Alegre, R. S. Brazil (2015): Use of Gastrin-Releasing Peptide in Children with Autism Diagnosis. Available online at https://www.cochranelibrary.com/central/doi/10.1002/central/CN-01834525/full.
832. Sutoko, S.; Monden, Y.; Tokuda, T.; Ikeda, T.; Nagashima, M.; Kiguchi, M. et al. (2019): Distinct methylphenidate-evoked response measured using functional near-infrared spectroscopy during go/no-go task as a supporting differential diagnostic tool between attention-deficit/hyperactivity disorder and autism spectrum disorder comorbid children. In Front Hum Neurosci 13. Available online at https://www.cochranelibrary.com/central/doi/10.1002/central/CN-01978203/full.
833. Wichers, R. H.; Findon, J. L.; Jelsma, A.; Giampietro, V.; Stoencheva, V.; Robertson, D. M. et al. (2019): Modulation of brain activation during executive functioning in autism with citalopram 9 (1), p. 286. Available online at https://www.cochranelibrary.com/central/doi/10.1002/central/CN-02009436/full
834. NCT03887754 (2019): Therapeutic Issues for Autism. Available online at https://www.cochranelibrary.com/central/doi/10.1002/central/CN-01931052/full.

835. NCT03963479 (2019): Vitamin B6 and Magnesium- A Clinical Trial on ASD Patients. Available online at https://www.cochranelibrary.com/central/doi/10.1002/central/CN-01933082/full.

836. ISRCTN17120714 (2005): Risperidone in children and adolescents with severe disruptive behaviour problems. Available online at https://www.cochranelibrary.com/central/doi/10.1002/central/CN-01816266/full.
837. Crutel, V.; Lambert, E.; Penelaud, P-F; Albarran Severo, C.; Fuentes, J.; Rosier, A. et al. (2020): Bumetanide Oral Liquid Formulation for the Treatment of Children and Adolescents with Autism Spectrum Disorder: design of Two Phase III Studies (SIGN Trials). Available online at https://www.cochranelibrary.com/central/doi/10.1002/central/CN-02204753/full.

838. Zhu, Y.; Gleissl, T.; Sanders, K.; Squassante, L.; Murtagh, L.; Ahlers, S. et al. (2020): 6.9 CLUSTERS OF MENTAL HEALTH COMORBIDITIES AND CONCOMITANT MEDICATIONS OF PARTICIPANTS IN THREE RANDOMIZED CONTROLLED TRIALS (VANILLA, V1ADUCT, AV1ATION) WITH AUTISM SPECTRUM DISORDER 59 (10), S162-. Available online at https://www.cochranelibrary.com/central/doi/10.1002/central/CN-02192228/full.
839. Batebi, N.; Moghaddam, H. S.; Hasanzadeh, A.; Fakour, Y.; Mohammadi; Akhondzadeh, S. (2020): Folinic Acid as Adjunctive Therapy in Treatment of Inappropriate Speech in Children with Autism: a Double-Blind and Placebo-Controlled Randomized Trial. In Child psychiatry and human development. Available online at https://www.cochranelibrary.com/central/doi/10.1002/central/CN-02191703/full.
840. Eslamzadeh, M.; Hebrani, P.; Behdani, F.; Moghadam, M. D.; Panaghi, L.; Mirzadeh, M.; Arabgol, F. (2018): Assessment the efficacy of atomoxetine in autism spectrum disorders: a randomized, double-blind, placebo-controlled trial 12 (2). Available online at https://www.cochranelibrary.com/central/doi/10.1002/central/CN-01617384/full.
841. IRCT20090117001556N112 (2018): Efficacy of pregnenolon in treatment of irritability inteeanagers with autism. Available online at https://www.cochranelibrary.com/central/doi/10.1002/central/CN-01905374/full.
842. IRCT20090117001556N113 (2018): Effect of propentofylline in children with autism. Available online at https://www.cochranelibrary.com/central/doi/10.1002/central/CN-01947549/full.
843. IRCT20090117001556N114 (2018): Folinic acid in the treatment of autism spectrum disorder. Available online at https://www.cochranelibrary.com/central/doi/10.1002/central/CN-01947548/full.
844. IRCT20090117001556N124 (2020): Cilostazol in the treatment of Autism. Available online at https://www.cochranelibrary.com/central/doi/10.1002/central/CN-02169868/full

845. IRCT20190129042536N1 (2019): Effect of L-carnitine in autism. Available online at https://www.cochranelibrary.com/central/doi/10.1002/central/CN-01947460/full.
846. IRCT20190714044199N1 (2019): Efficacy of N-Acetyl cysteine in patients with autism spectrum disorder. Available online at https://www.cochranelibrary.com/central/doi/10.1002/central/CN-02069606/full.
847. IRCT20190915044774N1 (2020): The effect of Curcumin on autism spectrum disorder. Available online at https://www.cochranelibrary.com/central/doi/10.1002/central/CN-02170944/full.
848. IRCT20200317046801N2 (2020): The effect of ondansetron on autism. Available online at https://www.cochranelibrary.com/central/doi/10.1002/central/CN-02171580/full.
849. IRCT20200712048084N1 (2020): Effect of Vitamin C in treatment of Autism Spectrum Disorders. Available online at https://www.cochranelibrary.com/central/doi/10.1002/central/CN-02187976/full.
850. Ann Abraham, D.; Narasimhan, U.; Christy, S.; Muhasaparur Ganesan, R. (2020): Effect of l-Carnosine as adjunctive therapy in the management of children with autism spectrum disorder: a randomized controlled study. In Amino acids. Available online at https://www.cochranelibrary.com/central/doi/10.1002/central/CN-02204805/full.
851. CTRI/2019/07/020102 (2019): Effect of supplementations in children with Autism Spectrum Disorders. Available online at https://www.cochranelibrary.com/central/doi/10.1002/central/CN-02065919/full.
852. CTRI/2019/09/021079 (2019): Effect of vitamin D in autism spectrum disorder: a clinical trial. Available online at https://www.cochranelibrary.com/central/doi/10.1002/central/CN-02066398/full.
853. Fang, L.; Jiang, X.; Huang, Y.; Sun, Y.; Xie, Y.; Wang, L. (2018): Efficacy of Vitamin D combined with ω-3 fatty acid in treatment of children with autism spectrum disorder 18 (5), 347-350 and 363. Available online at https://www.cochranelibrary.com/central/doi/10.1002/central/CN-01788897/full.
854. Bartz, J. A.; Nitschke, J. P.; Krol, S. A.; Tellier, P. P. (2019): Oxytocin Selectively Improves Empathic Accuracy: a Replication in Men and Novel Insights in Women. In Biological psychiatry. Cognitive neuroscience and neuroimaging 4 (12), pp. 1042–1048. Available online at https://www.cochranelibrary.com/central/doi/10.1002/central/CN-01936831/full.
855. EUCTR2010-019519-39-NL (2011): Efficacy of RAD001/everolimus in Autism and NeuroPsychological deficits in children with tuberous sclerosis complex (RAPIT-trial). Available online at https://www.cochranelibrary.com/central/doi/10.1002/central/CN-01806504/full.
856. EUCTR2011-006302-28-IT (2012): Dopamine modulation of oxytocin prosocial effects. Available online at https://www.cochranelibrary.com/central/doi/10.1002/central/CN-01851676/full.
857. Hojgaard, DRMA; Skarphedinsson, G.; Ivarsson, T.; Weidle, B.; Nissen, J.; Hybel, K. A. et al. (2020): 24.4 THE NORDIC LONG-TERM OCD TREATMENT STUDY: SPECIFIC POPULATIONS AND OUTCOME FROM CBT. In Journal of the American Academy of Child and Adolescent Psychiatry 59 (10), S303-. Available online at https://www.cochranelibrary.com/central/doi/10.1002/central/CN-02192269/full.
858. IRCT201107071483N2 (2011): Methylphenidate effect on fine motor skills of children with attention deficit/hyperactivity disorder. Available online at https://www.cochranelibrary.com/central/doi/10.1002/central/CN-01797967/full.
859. IRCT201203167462N5 (2012): Evaluating effects of Cyproheptadine and Folic acid in children with ADHD receiving Ritalin. Available online at https://www.cochranelibrary.com/central/doi/10.1002/central/CN-01893547/full.
860. IRCT2013012712302N1 (2013): Effectiveness of Medication and Combined Medication and Parent Management Training on children with Attention Deficit/Hyperactivity Disorder.

Available online at https://www.cochranelibrary.com/central/doi/10.1002/central/CN-01851266/full.
861. JPRN-UMIN000025922 (2017): Safety and pharmacokinetics of single and repeated dose of intra-nasal TTA-121 in healthy volunteers (Phase 1 trial). Available online at https://www.cochranelibrary.com/central/doi/10.1002/central/CN-01824747/full.
862. Keim, S. A.; Gracious, B.; Boone, K. M.; Klebanoff, M. A.; Rogers, L. K.; Rausch, J. et al. (2018): ω-3 and ω-6 Fatty Acid Supplementation May Reduce Autism Symptoms Based on Parent Report in Preterm Toddlers 148 (2), pp. 227–235. Available online at https://www.cochranelibrary.com/central/doi/10.1002/central/CN-01617572/full.
863. Mizuguchi, M.; Ikeda, H.; Kagitani-Shimono, K.; Yoshinaga, H.; Suzuki, Y.; Aoki, M. et al. (2019): Everolimus for epilepsy and autism spectrum disorder in tuberous sclerosis complex: eXIST-3 substudy in Japan 41 (1), pp. 1–10. Available online at https://www.cochranelibrary.com/central/doi/10.1002/central/CN-01913389/full.
864. NL8930 (2020): First-in-Human study of RGH-338. Available online at https://www.cochranelibrary.com/central/doi/10.1002/central/CN-02189070/full.
865. Siper, P.; Tavassoli, T.; George-Jones, J.; Lurie, S.; Rowe, M.; Weissman, J. et al. (2018): The sensory domain as a target for treatment in ASD clinical trials: electrophysiological and behavioral markers of therapeutic change. In Biological Psychiatry (9), S370. Available online at https://www.cochranelibrary.com/central/doi/10.1002/central/CN-01919994/full.
866. ChiCTR-ONRC-11001542 (2011): Efficacy of folic acid treatment on methylation capacity and oxidatie stress condition in children with autism. Available online at https://www.cochranelibrary.com/central/doi/10.1002/central/CN-01803867/full.
867. ChiCTR-ONRC-12002686 (2012): Rudimental research into effects of combination therapy of rehabilitation training and oral bumetanide on children with autism. Available online at https://www.cochranelibrary.com/central/doi/10.1002/central/CN-01877951/full.
868. JPRN-UMIN000003812 (2010): Evaluation of efficacy of intranasal oxytocin spray on children with autistic disorder. Available online at https://www.cochranelibrary.com/central/doi/10.1002/central/CN-01804032/full.
869. JPRN-UMIN000024270 (2016): study of everolimus for autistic spectrum disorder in tuberous sclerosis. Available online at https://www.cochranelibrary.com/central/doi/10.1002/central/CN-01828904/full.
870. JPRN-UMIN000028350 (2017): Tipepidine in children with Autism Spectrum Disorder : a 4-week, open-label, preliminary study. Available online at https://www.cochranelibrary.com/central/doi/10.1002/central/CN-01890134/full.
871. NCT01617460 (2012): A Long-term, Extended Treatment Study of Aripiprazole in Pediatric Patients With Autistic Disorder. Available online at https://www.cochranelibrary.com/central/doi/10.1002/central/CN-02036574/full.
872. Findling, R. L. (2019): 48.4 VASOPRESSIN RECEPTOR ANTAGONISM AS A POTENTIAL TREATMENT OPTION FOR THE CORE SYMPTOMS OF ASD: RESULTS OF BALOVAPTAN FROM EARLY DEVELOPMENT. In Journal of the American Academy of Child and Adolescent Psychiatry 58 (10), S373-. Available online at https://www.cochranelibrary.com/central/doi/10.1002/central/CN-01996620/full.
873. Le Arnold (2018): Placebo-Controlled Pilot Data for Three Complementary/Alternative Treatments in Autism 57 (10), S117-. Available online at https://www.cochranelibrary.com/central/doi/10.1002/central/CN-01654973/full.
874. IRCT20191113045429N1 (2020): Virtual Reality and Autism. Available online at https://www.cochranelibrary.com/central/doi/10.1002/central/CN-02171074/full.
875. JPRN-UMIN000041613 (2020): Hyperbaric oxygen therapy versus Risperidone in autistic children: a comparative study. Available online at https://www.cochranelibrary.com/central/doi/10.1002/central/CN-02188818/full.

876. Sanctuary, M. R.; Kain, J. N.; Chen, S. Y.; Kalanetra, K.; Lemay, D. G.; Rose, D. R. et al. (2019): Pilot study of probiotic/colostrum supplementation on gut function in children with autism and gastrointestinal symptoms 14. Available online at https://www.cochranelibrary.com/central/doi/10.1002/central/CN-01706698/full.
877. 36.2 ADVANCING AUTISM SPECTRUM DISORDER DETECTION AND TREATMENT: a TRANSLATIONAL APPROACH (2020) 59 (10), S321-S322. Available online at https://www.cochranelibrary.com/central/doi/10.1002/central/CN-02207714/full.
878. Mousavinejad, E.; Ghaffari, M. A.; Riahi, F.; Hajmohammadi, M.; Tiznobeyk, Z.; Mousavinejad, M. (2018): Coenzyme Q10 supplementation reduces oxidative stress and decreases antioxidant enzyme activity in children with autism spectrum disorders 265, pp. 62–69. Available online at https://www.cochranelibrary.com/central/doi/10.1002/central/CN-01608134/full.
879. Nogay, N. H.; Walton, J.; Roberts, K. M.; Nahikian-Nelms, M.; an Witwer (2020): The Effect of the Low FODMAP Diet on Gastrointestinal Symptoms, Behavioral Problems and Nutrient Intake in Children with Autism Spectrum Disorder: a Randomized Controlled Pilot Trial. Available online at https://www.cochranelibrary.com/central/doi/10.1002/central/CN-02194284/full.
880. Levetiracetam does not improve behavioral disturbances associated with autism (2007). In Brown university child & adolescent psychopharmacology update, 9 (1), pp. 1–3. Available online at https://www.cochranelibrary.com/central/doi/10.1002/central/CN-02105869/full.
881. Omega-3 fatty acids examined in autism, associated symptoms (2007). In Brown university child & adolescent psychopharmacology update 9 (6), pp. 5–6. Available online at https://www.cochranelibrary.com/central/doi/10.1002/central/CN-02105901/full.
882. Study of risperidone with autistic children finds no detrimental cognitive effects (2008). In Brown university child & adolescent psychopharmacology update 10 (9), pp. 3–4. Available online at https://www.cochranelibrary.com/central/doi/10.1002/central/CN-02105671/full.
883. 36.2 ADVANCING AUTISM SPECTRUM DISORDER DETECTION AND TREATMENT: a TRANSLATIONAL APPROACH (2020) 59 (10), S321-S322. Available online at https://www.cochranelibrary.com/central/doi/10.1002/central/CN-02207714/full.
884. Alaerts, K.; Steyaert, J.; Vanaudenaerde, B.; Wenderoth, N.; Bernaerts, S. (2020): Changes in endogenous oxytocin levels after intranasal oxytocin treatment in adult men with autism: an exploratory study with long-term follow-up. Available online at https://www.cochranelibrary.com/central/doi/10.1002/central/CN-02214466/full.
885. Ann Abraham, D.; Narasimhan, U.; Christy, S.; Muhasaparur Ganesan, R. (2020): Effect of L-Carnosine as adjunctive therapy in the management of children with autism spectrum disorder: a randomized controlled study 52 (11), pp. 1521–1528. Available online at https://www.cochranelibrary.com/central/doi/10.1002/central/CN-02204805/full.
886. Ann Abraham D; Narasimhan U; Christy S; Muhasaparur Ganesan R (2020): Effect of L-Carnosine as adjunctive therapy in the management of children with autism spectrum disorder: a randomized controlled study. In *Amino acids* 52 (11), pp. 1521–1528. Available online at https://pubmed.ncbi.nlm.nih.gov/33170378/.
887. Ayatollahi, A.; Bagheri, S.; Ashraf-Ganjouei, A.; Moradi, K.; Mohammadi; Akhondzadeh, S. (2020a): Does Pregnenolone Adjunct to Risperidone Ameliorate Irritable Behavior in Adolescents With Autism Spectrum Disorder: a Randomized, Double-Blind, Placebo-Controlled Clinical Trial? 43 (5), pp. 139–145. Available online at https://www.cochranelibrary.com/central/doi/10.1002/central/CN-02177104/full.
888. Ayatollahi, A.; Bagheri, S.; Ashraf-Ganjouei, A.; Moradi, K.; Mohammadi, M-R; Akhondzadeh, S. (2020b): Does Pregnenolone Adjunct to Risperidone Ameliorate Irritable Behavior in Adolescents With Autism Spectrum Disorder: a Randomized, Double-Blind,

Placebo-Controlled Clinical Trial? Available online at https://www.cochranelibrary.com/central/doi/10.1002/central/CN-02159272/full.
889. Ayuse, T.; Ozaki-Honda, Y.; Kurata, S.; Mishima, G.; Kiriishi, K.; Magata, N. et al. (2020): Study on the preventive effect of ramelteon on the onset of sleep disorder after general anesthesia in patients with autism spectrum disorder: a study protocol 99 (43), e22826-. Available online at https://www.cochranelibrary.com/central/doi/10.1002/central/CN-02202627/full.
890. Batebi, N.; Moghaddam, H. S.; Hasanzadeh, A.; Fakour, Y.; Mohammadi; Akhondzadeh, S. (2020): Folinic Acid as Adjunctive Therapy in Treatment of Inappropriate Speech in Children with Autism: a Double-Blind and Placebo-Controlled Randomized Trial. Available online at https://www.cochranelibrary.com/central/doi/10.1002/central/CN-02191703/full.
891. Batebi N; Moghaddam HS; Hasanzadeh A; Fakour Y; Mohammadi MR; Akhondzadeh S (2021): Folinic Acid as Adjunctive Therapy in Treatment of Inappropriate Speech in Children with Autism: A Double-Blind and Placebo-Controlled Randomized Trial. In *Child psychiatry and human development* 52 (5), pp. 928–938. Available online at https://pubmed.ncbi.nlm.nih.gov/33029705/.
892. Benner, S.; Aoki, Y.; Watanabe, T.; Endo, N.; Abe, O.; Kuroda, M. et al. (2021): Neurochemical evidence for differential effects of acute and repeated oxytocin administration 26 (2), pp. 710–720. Available online at https://www.cochranelibrary.com/central/doi/10.1002/central/CN-02277619/full.
893. Bernaerts, S.; Boets, B.; Steyaert, J.; Wenderoth, N.; Alaerts, K. (2020): Oxytocin treatment attenuates amygdala activity in autism: a treatment-mechanism study with long-term follow-up 10 (1), p. 383. Available online at https://www.cochranelibrary.com/central/doi/10.1002/central/CN-02200814/full.
894. Bernaerts S; Boets B; Steyaert J; Wenderoth N; Alaerts K (2020): Oxytocin treatment attenuates amygdala activity in autism: a treatment-mechanism study with long-term follow-up. In *Translational psychiatry* 10 (1), p. 383. Available online at https://pubmed.ncbi.nlm.nih.gov/33159033/.
895. Borowiak, K.; Kriegstein, K. von (2020): Intranasal oxytocin modulates brain responses to voice-identity recognition in typically developing individuals, but not in ASD 10 (1), p. 221. Available online at https://www.cochranelibrary.com/central/doi/10.1002/central/CN-02139252/full.
896. Brito, A. R.; Vairo, G. P.T.; Dias, APBH; Olej, B.; Nascimento, O. J.M.; Vasconcelos, M. M. (2021): Effect of prednisolone on language function in children with autistic spectrum disorder: a randomized clinical trial 97 (1), pp. 22–29. Available online at https://www.cochranelibrary.com/central/doi/10.1002/central/CN-02233239/full.
897. Brito AR; Vairo GPT; Dias APBH; Olej B; Nascimento OJM; Vasconcelos MM (2021): Effect of prednisolone on language function in children with autistic spectrum disorder: a randomized clinical trial. In *J Pediatr (Rio J)* 97 (1), pp. 22–29. Available online at https://pubmed.ncbi.nlm.nih.gov/32330433/.
898. Cannabinoid treatment for autism: a proof-of-concept randomized trial (2021) 12 (1). Available online at https://www.cochranelibrary.com/central/doi/10.1002/central/CN-02271090/full.
899. Castellanos, F. X. (2019): A placebo-controlled double-blind trial of cannabinoids in children and adolescents with autism spectrum disorder. In *Neuropsychopharmacology* 44, pp. 61–62. Available online at https://www.cochranelibrary.com/central/doi/10.1002/central/CN-02147378/full.
900. Chez, M.; Kile, S.; Lepage, C.; Parise, C.; Benabides, B.; Hankins, A. (2020): A Randomized, Placebo-Controlled, Blinded, Crossover, Pilot Study of the Effects of Dextromethorphan/Quinidine for the Treatment of Neurobehavioral Symptoms in Adults with Autism 50 (5), pp. 1532–1538. Available online at https://www.cochranelibrary.com/central/doi/10.1002/central/CN-02180353/full.

901. Crutel, V.; Lambert, E.; Penelaud, P. F.; Albarrán Severo, C.; Fuentes, J.; Rosier, A. et al. (2021): Bumetanide Oral Liquid Formulation for the Treatment of Children and Adolescents with Autism Spectrum Disorder: design of Two Phase III Studies (SIGN Trials) 51 (8), pp. 2959–2972. Available online at https://www.cochranelibrary.com/central/doi/10.1002/central/CN-02204753/full.
902. Crutel V; Lambert E; Penelaud PF; Albarrán Severo C; Fuentes J; Rosier A et al. (2021): Bumetanide Oral Liquid Formulation for the Treatment of Children and Adolescents with Autism Spectrum Disorder: Design of Two Phase III Studies (SIGN Trials). In *Journal of autism and developmental disorders* 51 (8), pp. 2959–2972. Available online at https://pubmed.ncbi.nlm.nih.gov/33151500/.
903. Effects of Extended-Release Methylphenidate Treatment on Cognitive Task Performance in Children with Autism Spectrum Disorder and Attention-Deficit/Hyperactivity Disorder (2020). Available online at https://www.cochranelibrary.com/central/doi/10.1002/central/CN-02182538/full.
904. Hayashi, M.; Mishima, K.; Fukumizu, M.; Takahashi, H.; Ishikawa, Y.; Hamada, I. et al. (2021): Melatonin Treatment and Adequate Sleep Hygiene Interventions in Children with Autism Spectrum Disorder: a Randomized Controlled Trial. Available online at https://www.cochranelibrary.com/central/doi/10.1002/central/CN-02294484/full.
905. Herscu P; Handen BL; Arnold LE; Snape MF; Bregman JD; Ginsberg L et al. (2020): The SOFIA Study: Negative Multi-center Study of Low Dose Fluoxetine on Repetitive Behaviors in Children and Adolescents with Autistic Disorder. In *Journal of autism and developmental disorders* 50 (9), pp. 3233–3244. Available online at https://pubmed.ncbi.nlm.nih.gov/31267292/.
906. Hollander, E.; Jacob, S.; Jou, R. J.; McNamara, N.; Sikich, L.; Tobe, R. et al. (2020a): A PHASE 2 RANDOMIZED CONTROLLED TRIAL OF BALOVAPTAN IN PEDIATRIC PARTICIPANTS WITH AUTISM SPECTRUM DISORDER 59 (10), S262-S263. Available online at https://www.cochranelibrary.com/central/doi/10.1002/central/CN-02192238/full.
907. Hollander, E.; Uzunova, G.; Taylor, B. P.; Noone, R.; Racine, E.; Doernberg, E. et al. (2020b): Randomized crossover feasibility trial of helminthic Trichuris suis ova versus placebo for repetitive behaviors in adult autism spectrum disorder 21 (4), pp. 291–299. Available online at https://www.cochranelibrary.com/central/doi/10.1002/central/CN-02248993/full.
908. IRCT20090117001556N124 (2020): Cilostazol in the treatment of Autism. Available online at https://www.cochranelibrary.com/central/doi/10.1002/central/CN-02169868/full.
909. IRCT20190915044774N1 (2020): The effect of Curcumin on autism spectrum disorder. Available online at https://www.cochranelibrary.com/central/doi/10.1002/central/CN-02170944/full.
910. IRCT20191113045429N1 (2020): Virtual Reality and Autism. Available online at https://www.cochranelibrary.com/central/doi/10.1002/central/CN-02171074/full.
911. IRCT20200317046801N2 (2020): The effect of ondansetron on autism. Available online at https://www.cochranelibrary.com/central/doi/10.1002/central/CN-02171580/full.
912. IRCT20200712048084N1 (2020): Effect of Vitamin C in treatment of Autism Spectrum Disorders. Available online at https://www.cochranelibrary.com/central/doi/10.1002/central/CN-02187976/full.
913. Jacob, S.; Veenstra-VanderWeele, J.; Murphy, D.; McCracken, J. T.; Smith, J.; Sanders, K. et al. (2020): 6.14 PHASE 3 RANDOMIZED CONTROLLED TRIAL OF BALOVAPTAN IN ADULTS WITH AUTISM SPECTRUM DISORDER 59 (10), S163-S164. Available online at https://www.cochranelibrary.com/central/doi/10.1002/central/CN-02192230/full.
914. Javadfar, Z.; Abdollahzad, H.; Moludi, J.; Rezaeian, S.; Amirian, H.; Foroughi, A. A. et al. (2020): Effects of vitamin D supplementation on core symptoms, serum serotonin, and interleukin-6 in children with autism spectrum disorders: a randomized clinical trial 79, p. 110986. Available online at https://www.cochranelibrary.com/central/doi/10.1002/central/CN-02175422/full.

915. Javadfar Z; Abdollahzad H; Moludi J; Rezaeian S; Amirian H; Foroughi AA et al. (2020): Effects of vitamin D supplementation on core symptoms, serum serotonin, and interleukin-6 in children with autism spectrum disorders: A randomized clinical trial. In *Nutrition (Burbank, Los Angeles County, Calif.)* 79, p. 110986. Available online at https://pubmed.ncbi.nlm.nih.gov/32966919/.
916. JPRN-JMA-IIA00438 (2020): The efficacy and safety of pyridoxamine in patients with autism spectrum disorder; Exploratory physician-led Phase 2 trial. Available online at https://www.cochranelibrary.com/central/doi/10.1002/central/CN-02172398/full.
917. JPRN-jRCT2021200001 (2020): The efficacy and safety of pyridoxamine in patients with autism spectrum disorder; Exploratory physician-led Phase 2 trial. Available online at https://www.cochranelibrary.com/central/doi/10.1002/central/CN-02172414/full.
918. JPRN-UMIN000041613 (2020): Hyperbaric oxygen therapy versus Risperidone in autistic children: a comparative study. Available online at https://www.cochranelibrary.com/central/doi/10.1002/central/CN-02188818/full.
919. Kato, Y.; Kuwabara, H.; Okada, T.; Munesue, T.; Benner, S.; Kuroda, M. et al. (2021): Oxytocin-induced increase in N,N-dimethylglycine and time course of changes in oxytocin efficacy for autism social core symptoms 12 (1). Available online at https://www.cochranelibrary.com/central/doi/10.1002/central/CN-02265057/full.
920. Kong, X. J.; Liu, J.; Liu, K.; Koh, M.; Sherman, H.; Liu, S. et al. (2021): Probiotic and Oxytocin Combination Therapy in Patients with Autism Spectrum Disorder: a Randomized, Double-Blinded, Placebo-Controlled Pilot Trial 13 (5). Available online at https://www.cochranelibrary.com/central/doi/10.1002/central/CN-02273749/full.
921. Moerkerke, M.; Daniels, N.; van der Donck, S.; Steyaert, J.; Alaerts, K.; Boets, B. (2021): P.318 Neurobiological marker and intervention for socio-communicative impairments in autism spectrum disorders 44, S51-S52. Available online at https://www.cochranelibrary.com/central/doi/10.1002/central/CN-02266664/full.
922. Moradi, H.; Sohrabi, M.; Taheri, H.; Khodashenas, E.; Movahedi, A. (2018): The effects of different combinations of perceptual-motor exercises, music, and vitamin D supplementation on the nerve growth factor in children with high-functioning autism 31, pp. 139–145. Available online at https://www.cochranelibrary.com/central/doi/10.1002/central/CN-02198454/full.
923. NCT04509401 (2020): Effects of High Dose of Vitamin B6 With Magnesium in Children With Autism Spectrum Disorder. Available online at https://www.cochranelibrary.com/central/doi/10.1002/central/CN-02146043/full.
924. NCT04517799 (2020): Trial of Cannabidiol to Treat Severe Behavior Problems in Children With Autism. Available online at https://www.cochranelibrary.com/central/doi/10.1002/central/CN-02162910/full.
925. NCT04520685 (2020): CAnnabidiol Study in Children With Autism Spectrum DisordEr. Available online at https://www.cochranelibrary.com/central/doi/10.1002/central/CN-02162969/full.
926. NCT04623398 (2020): Effect of Lithium in Patients With Autism Spectrum Disorder and Phelan-McDermid Syndrome (SHANK3 Haploinsufficiency). Available online at https://www.cochranelibrary.com/central/doi/10.1002/central/CN-02197416/full.
927. NL8930 (2020): First-in-Human study of RGH-338. Available online at https://www.cochranelibrary.com/central/doi/10.1002/central/CN-02189070/full.
928. Nogay, N. H.; Walton, J.; Roberts, K. M.; Nahikian-Nelms, M.; an Witwer (2021): The Effect of the Low FODMAP Diet on Gastrointestinal Symptoms, Behavioral Problems and Nutrient Intake in Children with Autism Spectrum Disorder: a Randomized Controlled Pilot Trial 51 (8), pp. 2800–2811. Available online at https://www.cochranelibrary.com/central/doi/10.1002/central/CN-02194284/full.
929. Nogay NH; Walton J; Roberts KM; Nahikian-Nelms M; Witwer AN (2021): The Effect of the Low FODMAP Diet on Gastrointestinal Symptoms, Behavioral Problems and Nutrient Intake in Children with Autism Spectrum Disorder: A Randomized Controlled Pilot Trial. In *Journal of*

autism and developmental disorders 51 (8), pp. 2800–2811. Available online at https://pubmed.ncbi.nlm.nih.gov/33057858/.
930. Pearson DA; Santos CW; Aman MG; Arnold LE; Lane DM; Loveland KA et al. (2020): Effects of Extended-Release Methylphenidate Treatment on Cognitive Task Performance in Children with Autism Spectrum Disorder and Attention-Deficit/Hyperactivity Disorder. In *Journal of Child & Adolescent Psychopharmacology* 30 (7), pp. 414–426. Available online at https://pubmed.ncbi.nlm.nih.gov/32644833/.
931. Posey, D. J.; McDougle, C. J.; Aman, M. G.; Arnold, L. E.; Scahill, L.; McCracken, J. T. (2004): A randomized, double-blind, placebo-controlled, crossover trial of methylphenidate in children with hyperactivity associated with pervasive developmental disorders 29, S142-3. Available online at https://www.cochranelibrary.com/central/doi/10.1002/central/CN-02279342/full.
932. Procyshyn, T. L.; Lombardo, M. V.; Lai, M. C.; Auyeung, B.; Crockford, S. K.; Deakin, J. et al. (2020): Effects of oxytocin administration on salivary sex hormone levels in autistic and neurotypical women 11 (1), p. 20. Available online at https://www.cochranelibrary.com/central/doi/10.1002/central/CN-02147527/full.
933. Rationale, design, and methods of the autism centers of excellence (ACE) network study of oxytocin in autism to improve reciprocal social behaviors (SOARS-B) (2020), 106103-. Available online at https://www.cochranelibrary.com/central/doi/10.1002/central/CN-02183313/full.
934. Rationale, design, and methods of the Autism Centers of Excellence (ACE) network Study of Oxytocin in Autism to improve Reciprocal Social Behaviors (SOARS-B) (2020) 98. Available online at https://www.cochranelibrary.com/central/doi/10.1002/central/CN-02207105/full.
935. Santocchi, E.; Guiducci, L.; Prosperi, M.; Calderoni, S.; Gaggini, M.; Apicella, F. et al. (2020): Effects of Probiotic Supplementation on Gastrointestinal, Sensory and Core Symptoms in Autism Spectrum Disorders: a Randomized Controlled Trial 11. Available online at https://www.cochranelibrary.com/central/doi/10.1002/central/CN-02192717/full.
936. Santocchi E; Guiducci L; Prosperi M; Calderoni S; Gaggini M; Apicella F et al. (2020): Effects of Probiotic Supplementation on Gastrointestinal, Sensory and Core Symptoms in Autism Spectrum Disorders: A Randomized Controlled Trial. In *Frontiers in psychiatry* 11, p. 550593. Available online at https://pubmed.ncbi.nlm.nih.gov/33101079/.
937. Sharifzadeh, N.; Ghasemi, A.; Tavakol Afshari, J.; Moharari, F.; Soltanifar, A.; Talaei, A. et al. (2020): Intrathecal autologous bone marrow stem cell therapy in children with autism: a randomized controlled trial. Available online at https://www.cochranelibrary.com/central/doi/10.1002/central/CN-02213646/full.
938. Spanos M; Chandrasekhar T; Kim SJ; Hamer RM; King BH; McDougle CJ et al. (2020): Rationale, design, and methods of the Autism Centers of Excellence (ACE) network Study of Oxytocin in Autism to improve Reciprocal Social Behaviors (SOARS-B). In *Contemporary clinical trials* 98, p. 106103. Available online at https://pubmed.ncbi.nlm.nih.gov/32777383/.
939. Sprengers, J. J.; van Andel, D. M.; Zuithoff, N. P.A.; Keijzer-Veen, M. G.; Schulp, A. J.A.; Scheepers, F. E. et al. (2021): Bumetanide for Core Symptoms of Autism Spectrum Disorder (BAMBI): a Single Center, Double-Blinded, Participant-Randomized, Placebo-Controlled, Phase-2 Superiority Trial 60 (7), pp. 865–876. Available online at https://www.cochranelibrary.com/central/doi/10.1002/central/CN-02160281/full.
940. Sprengers JJ; van Andel DM; Zuithoff NPA; Keijzer-Veen MG; Schulp AJA; Scheepers FE et al. (2021): Bumetanide for Core Symptoms of Autism Spectrum Disorder (BAMBI): A Single Center, Double-Blinded, Participant-Randomized, Placebo-Controlled, Phase-2 Superiority Trial. In *Journal of the American Academy of Child and Adolescent Psychiatry* 60 (7), pp. 865–876. Available online at https://pubmed.ncbi.nlm.nih.gov/32730977/.
941. TCTR20200522003 (2020): Folate receptor alpha autoantibody in children with autism spectrum disorder: establishment of in-house ELISA and efficacy of folinic acid â?? a

randomized controlled trial. Available online at https://www.cochranelibrary.com/central/doi/10.1002/central/CN-02189820/full.
942. Wang, Y.; Li, N.; Yang, J. J.; Zhao, D. M.; Chen, B.; Zhang, G. Q. et al. (2020): Probiotics and fructo-oligosaccharide intervention modulate the microbiota-gut brain axis to improve autism spectrum reducing also the hyper-serotonergic state and the dopamine metabolism disorder 157, p. 104784. Available online at https://www.cochranelibrary.com/central/doi/10.1002/central/CN-02164416/full.
943. Wichers, R. H.; Findon, J. L.; Jelsma, A.; Giampietro, V.; Stoencheva, V.; Robertson, D. M. et al. (2021): Modulation of atypical brain activation during executive functioning in autism: a pharmacological MRI study of tianeptine 12 (1). Available online at https://www.cochranelibrary.com/central/doi/10.1002/central/CN-02264963/full.
944. Wink, L. K.; Reisinger, D. L.; Horn, P.; Shaffer, R. C.; O'Brien, K.; Schmitt, L. et al. (2021): Brief Report: intranasal Ketamine in Adolescents and Young Adults with Autism Spectrum Disorder-Initial Results of a Randomized, Controlled, Crossover, Pilot Study 51 (4), pp. 1392–1399. Available online at https://www.cochranelibrary.com/central/doi/10.1002/central/CN-02144333/full.
945. Wink LK; Reisinger DL; Horn P; Shaffer RC; O'Brien K; Schmitt L et al. (2021): Brief Report: Intranasal Ketamine in Adolescents and Young Adults with Autism Spectrum Disorder-Initial Results of a Randomized, Controlled, Crossover, Pilot Study. In *Journal of autism and developmental disorders* 51 (4), pp. 1392–1399. Available online at https://pubmed.ncbi.nlm.nih.gov/32642957/.
946. Wong NML; Findon JL; Wichers RH; Giampietro V; Stoencheva V; Murphy CM et al. (2020): Serotonin differentially modulates the temporal dynamics of the limbic response to facial emotions in male adults with and without autism spectrum disorder (ASD): a randomised placebo-controlled single-dose crossover trial. In *Neuropsychopharmacology : official publication of the American College of Neuropsychopharmacology* 45 (13), pp. 2248–2256. Available online at https://pubmed.ncbi.nlm.nih.gov/32388538/.
947. Yamasue, H.; Okada, T.; Munesue, T.; Kuroda, M.; Fujioka, T.; Uno, Y. et al. (2020): Effect of intranasal oxytocin on the core social symptoms of autism spectrum disorder: a randomized clinical trial 25 (8), pp. 1849–1858. Available online at https://www.cochranelibrary.com/central/doi/10.1002/central/CN-02252987/full.
948. Zhu, Y.; Gleissl, T.; Sanders, K.; Squassante, L.; Murtagh, L.; Ahlers, S. et al. (2020): 6.9 CLUSTERS OF MENTAL HEALTH COMORBIDITIES AND CONCOMITANT MEDICATIONS OF PARTICIPANTS IN THREE RANDOMIZED CONTROLLED TRIALS (VANILLA, V1ADUCT, AV1ATION) WITH AUTISM SPECTRUM DISORDER 59 (10), S162-. Available online at https://www.cochranelibrary.com/central/doi/10.1002/central/CN-02192228/full.
949. Zimmerman, A. W.; Singh, K.; Connors, S. L.; Liu, H.; Panjwani, A. A.; Lee, L-C et al. (2021): Randomized controlled trial of sulforaphane and metabolite discovery in children with Autism Spectrum Disorder 12 (1). Available online at https://www.cochranelibrary.com/central/doi/10.1002/central/CN-02283363/full.

950. Ben-Ari Y; Lemonnier E (2021): Using bumetanide to treat autism appears promising but further clinical trials are needed to confirm this approach. In *Acta paediatrica (Oslo, Norway : 1992)* 110 (5), pp. 1395–1397. Available online at https://pubmed.ncbi.nlm.nih.gov/33484191/

951. Jacob, S.; Anagnostou, E.; Hollander, E.; Jou, R.; McNamara, N.; Sikich, L. et al. (2020): Key insights gained from the balovaptan clinical development program 45, 329-. Available online at https://www.cochranelibrary.com/central/doi/10.1002/central/CN-02243595/full.

952. Ayuse T; Ozaki-Honda Y; Kurata S; Mishima G; Kiriishi K; Magata N et al. (2020): Study on the preventive effect of ramelteon on the onset of sleep disorder after general anesthesia in patients with autism spectrum disorder: A study protocol. In *Medicine* 99 (43), e22826. Available online at https://pubmed.ncbi.nlm.nih.gov/33120808/

953. Fastman, J.; Foss-Feig, J.; Frank, Y.; Halpern, D.; Harony-Nicolas, H.; Layton, C. et al. (2021): A randomized controlled trial of intranasal oxytocin in Phelan-McDermid syndrome. In *Molecular Autism* 12 (1), p. 62. DOI: 10.1186/s13229-021-00459-1.
954. Boone KM; Parrott A; Rausch J; Yeates KO; Klebanoff MA; Norris Turner A; Keim SA (2020): Fatty Acid Supplementation and Socioemotional Outcomes: Secondary Analysis of a Randomized Trial. In *Pediatrics* 146 (4). Available online at https://pubmed.ncbi.nlm.nih.gov/32887793/.
955. Daniels, N.; Soriano, JR; Prinsen, J.; Alaerts, K. (2021): P.310 Effects of intranasal administration of the neuromodulator oxytocin on autonomic cardiac function 44, S45-S46. Available online at https://www.cochranelibrary.com/central/doi/10.1002/central/CN-02266667/full.
956. EUCTR2016-002875-81-NL (2016): Bumetanide treatment for autism in clinical practice trial. Available online at https://www.cochranelibrary.com/central/doi/10.1002/central/CN-02237900/full.
957. Jiang X; Ma X; Geng Y; Zhao Z; Zhou F; Zhao W et al. (2021): Intrinsic, dynamic and effective connectivity among large-scale brain networks modulated by oxytocin. In *NeuroImage* 227, p. 117668. Available online at https://pubmed.ncbi.nlm.nih.gov/33359350/
958. ACTRN12620001197921 (2020): Combined gut-brain therapy for children with autism. Available online at https://www.cochranelibrary.com/central/doi/10.1002/central/CN-02239523/full.
959. IRCT20180503039517N5 (2020): Effects of vitamin D and/or aquatic exercise on IL-1ß and IL-1RA serum levels and behavior of children with autism spectrum disorder. Available online at https://www.cochranelibrary.com/central/doi/10.1002/central/CN-02187437/full.
960. Lai X; Zhang Q; Zhu J; Yang T; Guo M; Li Q et al. (2021): A weekly vitamin A supplementary program alleviates social impairment in Chinese children with autism spectrum disorders and vitamin A deficiency. In *European journal of clinical nutrition* 75 (7), pp. 1118–1125. Available online at https://pubmed.ncbi.nlm.nih.gov/33328600/.
961. NCT04639141 (2020): Combined Gut-brain Therapy for Children With Autism. Available online at https://www.cochranelibrary.com/central/doi/10.1002/central/CN-02205811/full.
962. Soltani Kouhbanani S; Khosrorad R; Zarenezhad S; Arabi SM (2021): Comparing the Effect of Risperidone, Virtual Reality and Risperidone on Social Skills, and Behavioral Problems in Children with Autism: A Follow-up Randomized Clinical Trial. In *Archives of Iranian medicine* 24 (7), pp. 534–541. Available online at https://pubmed.ncbi.nlm.nih.gov/34488318/.
963. Andari, E.; Cotton, A.; Cubells, J.; Rilling, J.; Young, L. (2020): Oxytocin dose-dependent effects on acc and amygdala activity during a socially dynamic game in autism spectrum disorders 45, 291-. Available online at https://www.cochranelibrary.com/central/doi/10.1002/central/CN-02242713/full.
964. Andari, E.; Cotton, A.; Cubells, J.; Rilling, J.; Young, L. (2021): Oxytocin Dose-Dependent Effects on Brain Function During a Socially Dynamic Game in Autism Spectrum Disorders 89 (9), S114-. Available online at https://www.cochranelibrary.com/central/doi/10.1002/central/CN-02267840/full.
965. EUCTR2014-005452-26-NO (2014): Effect of intranasal oxytocin on sosial cogitive tasks in autism spectrum disorder. Available online at https://www.cochranelibrary.com/central/doi/10.1002/central/CN-02186655/full.
966. Mayer AV; Wermter AK; Stroth S; Alter P; Haberhausen M; Stehr T et al. (2021): Randomized clinical trial shows no substantial modulation of empathy-related neural activation by intranasal oxytocin in autism. In *Scientific reports* 11 (1), p. 15056. Available online at https://pubmed.ncbi.nlm.nih.gov/34301983/.

967. Pretzsch CM; Floris DL; Voinescu B; Elsahib M; Mendez MA; Wichers R et al. (2021): Modulation of striatal functional connectivity differences in adults with and without autism spectrum disorder in a single-dose randomized trial of cannabidivarin. In *Molecular Autism* 12 (1), p. 49. Available online at https://pubmed.ncbi.nlm.nih.gov/34210360/.
968. Wichers RH; Findon JL; Jelsma A; Giampietro V; Stoencheva V; Robertson DM et al. (2021): Modulation of atypical brain activation during executive functioning in autism: a pharmacological MRI study of tianeptine. In *Molecular Autism* 12 (1), p. 14. Available online at https://pubmed.ncbi.nlm.nih.gov/33608048/.
969. Xin F; Zhou F; Zhou X; Ma X; Geng Y; Zhao W et al. (2021): Oxytocin Modulates the Intrinsic Dynamics Between Attention-Related Large-Scale Networks. In *Cerebral cortex (new york, N.Y. : 1991)* 31 (3), pp. 1848–1860. Available online at https://pubmed.ncbi.nlm.nih.gov/30535355/.
970. CTRI/2020/09/028149 (2020): A clinical trial to Evaluate Efficacy and Safety of Traditional Indian Medicine (TIM) of Siddha formulations in Children with Autism. Available online at https://www.cochranelibrary.com/central/doi/10.1002/central/CN-02186157/full.
971. IRCT20200115046137N2 (2021): Gluten-free diet in Autistic children. Available online at https://www.cochranelibrary.com/central/doi/10.1002/central/CN-02280465/full.
972. Nguyen LT; Nguyen PH; Hoang DM (2021): A phase II randomized clinical trial of the safety and efficacy of intravenous umbilical cord blood infusion for treatment of children with autism spectrum disorder. In *The Journal of pediatrics* 230, pp. 271–272. Available online at https://pubmed.ncbi.nlm.nih.gov/33271192/.
973. Sharifzadeh N; Ghasemi A; Tavakol Afshari J; Moharari F; Soltanifar A; Talaei A et al. (2021): Intrathecal autologous bone marrow stem cell therapy in children with autism: A randomized controlled trial. In *Asia-Pacific psychiatry : official journal of the Pacific Rim College of Psychiatrists* 13 (2), e12445. Available online at https://pubmed.ncbi.nlm.nih.gov/33150703/.
974. IRCT20090117001556N136 (2021): L-Carnitine for the treatment of autism. Available online at https://www.cochranelibrary.com/central/doi/10.1002/central/CN-02280097/full.
975. IRCT20190917044793N1 (2020): Transcranial electrical stimulation and pharmacological interventions in Autism Spectrum Disorder. Available online at https://www.cochranelibrary.com/central/doi/10.1002/central/CN-02187616/full.
976. Khan, Farhana; Rahman, Md Sayedur; Akhter, Shaheen; Momen, Abdul Basit Ibne; Raihan, Sheikh Golam (2021): Vitamin B6 and Magnesium on Neurobehavioral Status of Autism Spectrum Disorder: A Randomized, Double-Blind, Placebo Controlled Study. In *Bangla J Med* 32 (1), pp. 12–18. DOI: 10.3329/bjm.v32i1.51089.
977. Raghavan, Kadalraja; Dedeepiya, Vidyasagar Devaprasad; Ikewaki, Nobunao; Sonoda, Tohru; Iwasaki, Masaru; Preethy, Senthilkumar; Abraham, Samuel J. K. (2021): Improvement of behavioural pattern and alpha-synuclein levels in autism spectrum disorder after consumption of a beta-glucan food supplement in a randomized, parallel-group pilot clinical study
978. NCT04233502 (2020): Efficacy and Safety of Slenyto for Insomnia in Children With ASD. Available online at https://www.cochranelibrary.com/central/doi/10.1002/central/CN-02054185/full.
979. Malow BA; Findling RL; Schroder CM; Maras A; Breddy J; Nir T et al. (2021): Sleep, Growth, and Puberty After 2 Years of Prolonged-Release Melatonin in Children With Autism Spectrum Disorder. In *Journal of the American Academy of Child and Adolescent Psychiatry* 60 (2), 252-261.e3. Available online at https://pubmed.ncbi.nlm.nih.gov/31982581/
980. NCT02757066, Nobelpharma. Verification of the Efficacy of NPC-15 for Sleep Disorders of Children With Autism Spectrum Disorders. 2016. (the published manuscript is not a placebo-controlled trial)

981. Mohamed, A., M Ahmad, H., A Abdelrahman, A., F Ali, U., & A Khaled, K. (In Press). Therapeutic Impacts of Hyperbaric Oxygen Therapy and Risperidone on Children With Autism: A Clinical Trial. Basic and Clinical Neuroscience. Just Accepted publication Jul. 3, 2021. Doi: http://dx.doi.org/10.32598/bcn.2021.3122.1
982. NCT05013164. Role of Folinic Acid in Improving the Adaptive Skills and Language Impairment in Children With Autism Spectrum Disorder.

4.3 Eligible trials, included and ongoing

4.3.1 Included records in the systematic review

From the 203 eligible trials, 143 were included in the quantitative analysis, i.e. data for at least an outcome (underlined). Six studies were not included in the quantitative in spite of availability of data (*italics*), becauase of data reliability issues and retraction of the paper (k=2), or important concerns on methodology and reported data (k=4). Fifty-four studies did not provide appropriate data, due to crossover design, insufficient reporting, use of not appropriate scales for our review, assessment of outcomes not in the interest of our review.

1. Akkok 1995[3]
    - Akkok F, Gokler B, Oktem F, Reid LD, Sucuoglu B. Behavioral and biochemical papameters of naltrexone in the treatment of autism. [Turkish] Otizm'de naltrekson sagaltiminin davranissal ve biyokimyasal boyutlari. Turk Psikiyatri Dergisi. 1995;6(4):251–262.
2. *Aliyev_2018*[4] (excluded from the quantitative analysis due to serious concerns on the methodology reported in predatory journals)
    - Aliyev NA. A Double-Blind Placebo-Controlled Trial of Acediprol (Valproate Sodium) For Global Severity in Child Autism Spectrum Disorders. OJNBD. 2018;2(1). doi:10.32474/OJNBD.2018.02.000127.
3. *Aliyev_2018b*[5] (excluded from the quantitative analysis due to serious concerns on the methodology reported in predatory journals)
    - N. Aliyev, Z. Aliyev. A Double-Blind Placebo-Controlled Trial of levetiracetam for Global Severity in Child Autism Spectrum Disorders. ijirms. 2018;3(10). doi:10.23958/ijirms/vol03-i10/455.
4. Aman 2017[6–9]
    - Aman MG, Findling RL, Hardan AY, et al. Safety and Efficacy of Memantine in Children with Autism: randomized, Placebo-Controlled Study and Open-Label Extension. Journal of Child and Adolescent Psychopharmacology. 2017;27(5):403–412.
    - EUCTR2012-001630-33-GB, Forest Research Institute I. An Open-Label Extension Study of the Safety and Tolerability of Memantine in Pediatric Patients with Autism, Asperger's Disorder or Pervasive Developmental Disorder Not Otherwise Specified (PDD-NOS). 2012.
    - Katz E, Lateiner J, Spera A, Palmer R, Graham S. Memantine for the treatment of autism spectrum disorder: overview of the phase II clinical development program. Neurology. 2014;82(10).
    - NCT00872898, Merz Pharmaceuticals Gmb H, Forest L. Study of Pharmacokinetics, Safety, Efficacy, and Tolerability of Memantine in Children With Autism. 2009.
5. Amminger 2008[10, 11]
    - Gilbert DL. Regarding "Omega-3 Fatty Acids Supplementation in Children with Autism: A Double-Blind Randomized, Placebo-Controlled Pilot Study. Biological Psychiatry. 15;63(2):e13.
    - Amminger GP, Berger GE, Schäfer MR, Klier C, Friedrich MH, Feucht M. Omega-3 fatty acids supplementation in children with autism: a double-blind randomized, placebo-controlled pilot study. Biological Psychiatry. 2007;61(4):551–553.
6. Anagnostou 2012[12–14]

- Anagnostou E, Soorya L, Chaplin W, et al. Intranasal oxytocin versus placebo in the treatment of adults with autism spectrum disorders: a randomized controlled trial. Mol Autism. 2012;3(1):16.
- NCT00490802, Icahn School of Medicine at Mount, Sinai, Evdokia A. Intranasal Oxytocin in the Treatment of Autism. 2007.
- NCT01337687, National Alliance for Research on, Schizophrenia, Depression, Montefiore Medical C. Intranasal Oxytocin for the Treatment of Autism Spectrum Disorders. 2010.

7. Anderson 1984 (Campbell 1982) [15–17]
   - Anderson LT, Campbell M, Grega DM. Haloperidol in the treatment of infantile autism: effects on learning and behavioral symptoms. American journal of psychiatry. 1984;141(10):1195–1202.
   - Campbell M, Anderson LT, Small AM, Perry R, Green WH, Caplan R. The effects of haloperidol on learning and behavior in autistic children. Journal of autism and developmental disorders. 1982;12(2):167–175.
   - Ornitz EM. Should autistic children be treated with haloperidol? The American Journal of Psychiatry. 1985;142(7):883–884.
8. Anderson 1989[18]
   - Anderson LT, Campbell M, Adams P, Small AM, Perry R, Shell J. The effects of haloperidol on discrimination learning and behavioral symptoms in autistic children. Journal of autism and developmental disorders. 1989;19(2):227–239.
9. Arnold 2006[19]
   - Arnold LE, Aman MG, Cook AM, et al. Atomoxetine for hyperactivity in autism spectrum disorders: placebo-controlled crossover pilot trial. Journal of the American Academy of Child and Adolescent Psychiatry. 2006;45(10):1196–1205.
10. Arnold 2012[20, 21]
    - Arnold LE, Aman MG, Hollway J, et al. Placebo-controlled pilot trial of mecamylamine for treatment of autism spectrum disorders. Journal of Child & Adolescent Psychopharmacology. 2012;22(3):198–205.
    - NCT00773812, Autism S, Ohio State U. Placebo-Controlled Pilot Trial of Mecamylamine for Treatment of Autism Spectrum Disorders. 2008.
11. Arnold_2019[22, 23]
    - Arnold LE, Luna R.A, Williams K, et al. Probiotics for Gastrointestinal Symptoms and Quality of Life in Autism: a placebo-controlled pilot trial. In-press.
    - NCT02903030, Autism Treatment N, Autism S, Ohio State U. Probiotics for Quality of Life in Autism Spectrum Disorders. 2016.
12. August 1987[24]
    - August GJ, Raz N, Baird TD. Fenfluramine response in high and low functioning autistic children. Journal of the American Academy of Child and Adolescent Psychiatry. 1987;26(3):342–346.
13. Ballester 2015[25–28]
    - Ballester P, Martinez MJ, Javaloyes A, Hern, ez L, Peiro AM. Agomelatine effectiveness in sleep disturbances in autism spectrum disorder. Clinical therapeutics. 2015;37(8):e132-e133.
    - Ballester P, Martinez MJ, Inda M-D-M, et al. Evaluation of agomelatine for the treatment of sleep problems in adults with autism spectrum disorder and co-morbid intellectual disability. Journal of psychopharmacology (oxford, england). 2019:269881119864968. doi:10.1177/0269881119864968.

- EUCTR-2011-003313-42. Efficacy of agomelatine on sleep disturbance in Autism Spectrum Disorder (ASD). EU clinical trials register [www.clinicaltrialsregister.eu]. 2011.
- EUCTR2011-003313-42-ES, Hospital General Universitario de, Alicante. Agomelatine efficacy of the drug to improve sleep problems in autistic people. 2011.

14. Barthelemy 1989[29]
    - Barthelemy C, Bruneau N, Jouve J, Martineau J, Muh JP, Lelord G. Urinary dopamine metabolites as indicators of the responsiveness to fenfluramine treatment in children with autistic behavior. Journal of autism and developmental disorders. 1989;19(2):241–254.
15. Belsito 2001[30, 31]
    - Belsito KM, Kirk KS, L, a RJ, Law PA, Zimmerman AW. Lamotrigine therapy for childhood autism: a randomised, double-blind, placebo-controlled trial. Neurology. 1998;50(4):A85.
    - Belsito KM, Law PA, Kirk KS, L, a RJ, Zimmerman AW. Lamotrigine therapy for autistic disorder: a randomized, double-blind, placebo-controlled trial. Journal of autism and developmental disorders. 2001;31(2):175–181.
16. Bent 2011[32, 33]
    - Bent S, Bertoglio K, Ashwood P, Bostrom A, Hendren RL. A pilot randomized controlled trial of omega-3 fatty acids for autism spectrum disorder. Journal of autism and developmental disorders. 2011;41(5):545–554.
    - NCT00786799, Autism S, University of California, San Francisco. Omega-3 Fatty Acids for Autism Treatment. 2008.
17. Bent 2014[34, 35]
    - Bent S, Hendren RL, Z, et al. Internet-based, randomized, controlled trial of omega-3 fatty acids for hyperactivity in autism. Journal of the American Academy of Child and Adolescent Psychiatry. 2014;53(6):658–666.
    - NCT01694667, University of California, San Francisco, Hugo W. Moser Research Institute at Kennedy Krieger, Inc. Omega-3 Fatty Acids for Hyperactivity Treatment in Autism Spectrum Disorder. 2012.
18. Bernaets 2020[36–40, 4001]
    - Alaerts K, Bernaerts S, Prinsen J, Dillen C, Steyaert J, Wenderoth N. Oxytocin induces long-lasting adaptations within amygdala circuitry in autism: a treatment-mechanism study with randomized placebo-controlled design. Neuropsychopharmacology : official publication of the American College of Neuropsychopharmacology. 2020.
    - Bernaerts S, Boets B, Bosmans G, Steyaert J, Alaerts K. Behavioral effects of multiple-dose oxytocin treatment in autism: a randomized, placebo-controlled trial with long-term follow-up. Molecular Autism. 2020;11:6. doi:10.1186/s13229-020-0313-1.
    - Bernaerts S, Dillen C, Steyaert J, Alaerts K. The effects of four weeks of intranasal oxytocin on social responsiveness and repetitive and restricted behaviors in autism spectrum disorders: a randomized controlled trial. Biological psychiatry. Conference: 72nd annual scientific convention and meeting of the society of biological psychiatry, SOBP 2017. United states. 2017;81(10):S349-s350.
    - EUCTR2014-000586-45-BE, Leuven KU. The use of Oxytocin for Autism Spectrum Disorders: Investigating the effect on behavior and at the level of the brain. 2014.
    - NCT02940574. Neural and Behavioral Effects of Oxytocin in Autism Spectrum Disorders. https://ClinicalTrials.gov/show/NCT02940574. 2016.

    - Alaerts, K.; Bernaerts, S. (2019): P.509 Continual oxytocin treatment induces long-lasting adaptations within amygdala circuitry in autism: a randomized placebo-controlled trial. In European neuropsychopharmacology 29, S358-S359. Available online at https://www.cochranelibrary.com/central/doi/10.1002/central/CN-02049376/full.

- Alaerts, K.; Bernaerts, S.; Vanaudenaerde, B.; Daniels, N.; Wenderoth, N. (2019): Amygdala-Hippocampal Connectivity Is Associated With Endogenous Levels of Oxytocin and Can Be Altered by Exogenously Administered Oxytocin in Adults With Autism. In Biol Psychiatry Cogn Neurosci Neuroimaging 4 (7), pp. 655–663. Available online at https://www.cochranelibrary.com/central/doi/10.1002/central/CN-01962587/full.
- Alaerts K; Steyaert J; Vanaudenaerde B; Wenderoth N; Bernaerts S (2021): Changes in endogenous oxytocin levels after intranasal oxytocin treatment in adult men with autism: An exploratory study with long-term follow-up. In *European neuropsychopharmacology : the journal of the European College of Neuropsychopharmacology* 43, pp. 147–152. Available online at https://pubmed.ncbi.nlm.nih.gov/33309460/
- Bernaerts, S.; Boets, B.; Steyaert, J.; Wenderoth, N.; Alaerts, K. (2020): Oxytocin treatment attenuates amygdala activity in autism: a treatment-mechanism study with long-term follow-up 10 (1). Available online at https://www.cochranelibrary.com/central/doi/10.1002/central/CN-02200814/full.

19. Bertoglio 2010[41, 42]
    - Bertoglio K, Jill James S, Deprey L, Brule N, Hendren RL. Pilot study of the effect of methyl B12 treatment on behavioral and biomarker measures in children with autism. Journal of alternative and complementary medicine (new york, N.Y.). 2010;16(5):555–560.
    - NCT00273650, University of California D. Efficacy Study of Subcutaneous Methyl-B12 in Children With Autism. 2006.
20. Bolman 1999[43]
    - Bolman WM, Richmond JA. A double-blind, placebo-controlled, crossover pilot trial of low dose dimethylglycine in patients with autistic disorder. Journal of autism and developmental disorders. 1999;29(3):191–194.
21. Bolognani_2019[44, 45]
    - Bolognani F, Del Valle Rubido M, Squassante L, et al. A phase 2 clinical trial of a vasopressin V1a receptor antagonist shows improved adaptive behaviors in men with autism spectrum disorder. Sci Transl Med. 2019;11(491).
    - NCT01793441, Hoffmann-La R. A Study of RG7314 to Investigate Efficacy and Safety in Individuals With Autism Spectrum Disorders (ASD). 2013.
22. Bouvard 1995[46]
    - Bouvard MP, Leboyer M, Launay JM, et al. Low-dose naltrexone effects on plasma chemistries and clinical symptoms in autism: a double-blind, placebo-controlled study. Psychiatry research. 1995;58(3):191–201.
23. Buitelaar 1990[47–49]
    - Buitelaar JK, Engel, H., Ree JM, Wied D. Behavioral effects of Org 2766, a synthetic analog of the adrenocorticotrophic hormone (4-9), in 14 outpatient autistic children. Journal of autism and developmental disorders. 1990;20(4):467–478.
    - Buitelaar JK, Engel, H., et al. The use of adrenocorticotrophic hormone (4-9) analog ORG 2766 in autistic children: effects on the organization of behavior. Biological Psychiatry. 1992;31(11):1119–1129.
    - Buitelaar JK, van Engel, H., et al. Deficits in social behavior in autism and their modification by a synthetic adrenocorticotrophic hormone (4-9) analog. Experientia. 1992;48(4):391–394.
24. Buitelaar 1992[50]
    - Buitelaar JK, Engel, H., et al. The adrenocorticotrophic hormone (4-9) analog ORG 2766 benefits autistic children: report on a second controlled clinical trial. Journal of the American Academy of Child and Adolescent Psychiatry. 1992;31(6):1149–1156.
25. Campbell 1987[51, 52]

- Campbell M, Adams P, Small AM, et al. Efficacy and safety of fenfluramine in autistic children. J. AM. ACAD. Child adolesc. PSYCHIATRY. 1988;27(4):434–439.
- Campbell M, Small AM, Palij M, et al. The efficacy and safety of fenfluramine in autistic children: preliminary analysis of a double-blind study. Psychopharmacology Bulletin. 1987;23(1):123–127.

26. Campbell 1990[53–56]
    - Campbell M, Anderson LT, Small AM, Locascio JJ, Lynch NS, Choroco MC. Naltexone in autistic children: a double-blind and placebo-controlled study. Psychopharmacology Bulletin. 1990;26(1):130–135.
    - Campbell M, Anderson LT, Small AM, Locascio JJ, Lynch NS, Choroco MC. Naltrexone in autistic children: a double-blind and placebo-controlled study. Psychopharmacology Bulletin. 1990;26(1):130–135.
    - Campbell M, Anderson LT, Small AM, Adams P, Gonzalez NM, Ernst M. Naltrexone in autistic children: behavioral symptoms and attentional learning. Journal of the American Academy of Child and Adolescent Psychiatry. 1993;32(6):1283–1291.
    - Gonzalez NM, Campbell M, Small AM, et al. Naltrexone plasma levels, clinical response and effect on weight in autistic children. Psychopharmacology Bulletin. 1994;30(2):203–208.
27. Chez 2017[57–59]
    - Chez M, Kile S. A Randomized, placebo-controlled, blinded, crossover, single-center study of the effects of nuedexta in the treatment of neurobehavioral symptoms of adults with Autism spectrum disorder. Neurology. Conference: 70th Annual Meeting of the American Academy of Neurology, AAN. 2017;88(16).
    - Chez M, Kile S, Lepage C, Parise C, Benabides B, Hankins A. A Randomized, Placebo-Controlled, Blinded, Crossover, Pilot Study of the Effects of Dextromethorphan/Quinidine for the Treatment of Neurobehavioral Symptoms in Adults with Autism. Journal of Autism & Developmental Disorders. 2018.
    - NCT01630811, Sutter H. Nuedexta for the Treatment of Adults With Autism. 2012.
28. Chugani 2016[60, 61]
    - Chugani DC, Chugani HT, Wiznitzer M, et al. Efficacy of Low-Dose Buspirone for Restricted and Repetitive Behavior in Young Children with Autism Spectrum Disorder: a Randomized Trial. Journal of pediatrics. 2016;170:45-53.e1-4.
    - NCT00873509, National Institute of Neurological, Disorders, Stroke, Chugani DC. Buspirone in the Treatment of 2-6 Year Old Children With Autistic Disorder. 2009.
29. Cohen 1980[62]
    - Cohen IL, Campbell M, Posner D. Behavioral effects of haloperidol in young autistic children. An objective analysis using a within-subjects reversal design. J am acad child psychiatr. 1980;19(4):665–677.
30. Cortesi 2012[63]
    - Cortesi F, Giannotti F, Sebastiani T, Panunzi S, Valente D. Controlled-release melatonin, singly and combined with cognitive behavioural therapy, for persistent insomnia in children with autism spectrum disorders: a randomized placebo-controlled trial. Journal of sleep research. 2012;21(6):700–709.
31. Danfors 2005[64]
    - Danfors T, Knorring AL, Hartvig P, et al. Tetrahydrobiopterin in the treatment of children with autistic disorder: a double-blind placebo-controlled crossover study. Journal of Clinical Psychopharmacology. 2005;25(5):485–489.
32. Dean 2017[65–67]

- ACTRN12610000635066, University University of M. Efficacy Of N-Acetyl Cysteine In Autism: A Double-Blind, Placebo-Controlled Randomised Trial. 2010.
- Dean OM, Gray KM, Villagonzalo KA, et al. A randomised, double blind, placebo-controlled trial of a fixed dose of N-acetyl cysteine in children with autistic disorder. Australian and New Zealand Journal of Psychiatry. 2017;51(3):241–249.
- Dean OM, Gray K, Dodd S, et al. Does n-acetylcysteine improve behaviour in children with autism?: A mixed-methods analysis of the effects of n-acetylcysteine. Journal of Intellectual and Developmental Disability. 2018.

33. DeVane 2019[68, 69]
    - DeVane CL, Charles JM, Abramson RK, et al. Pharmacotherapy of Autism Spectrum Disorder: Results from the Randomized BAART Clinical Trial. Pharmacotherapy. 2019;39(6):626–635.
    - NCT01333072, Medical University of South, Carolina. Biomarkers in Autism of Aripiprazole and Risperidone Treatment (BAART). 2010.
34. Duker 1991[70]
    - Duker PC, Welles K, Seys D, Rensen H. Brief report: effects of fenfluramine on communicative, stereotypic, and inappropriate behaviors of autistic-type mentally handicapped individuals. Journal of autism and developmental disorders. 1991;21(3):355–363.
35. Ekman 1989[71]
    - Ekman G, Mir, a-Linné F, Gillberg C, Garle M, Wetterberg L. Fenfluramine treatment of twenty children with autism. Journal of autism and developmental disorders. 1989;19(4):511–532.
36. EUCTR2010-018740-13-NL[72] (unclear status, probably completed)
    - EUCTR2010-018740-13-NL, University Medical Center G. Short- and long-term effects of oxytocin on empathy and social behaviour in autistic and antisocial male adults. - Oxytocin effects in autistic and antisocial male adults. 2010.
37. EUCTR2014-001560-35-NL(Sprengers 2020) [73–75, 751, 752]
    - EUCTR2014-001560-35-NL, Brain Center Rudolf Magnus, University Medical Center Utrecht Department of Psychiatry Utrecht the Netherl, S. Bumetanide for Autism Treatment Study. 2016.
    - NTR6325, University Medical Center U. Bumetanide for the Autism Spectrum Clinical Effectiveness Trial. 2017.
    - Sprengers, J.; Andel van, D.; Oranje, B.; Linkenkaer-Hansen, K. K.; Hilgo, B. (2019a): P.4.11 Bumetanide in Autism Medication and Biomarker (BAMBI) study: medication response profiles 29, S708-S709. Available online at https://www.cochranelibrary.com/central/doi/10.1002/central/CN-01788411/full.
    - Sprengers, J.; Oranje, B.; van Marije Andel, D.; Juarez-Martinez, E.; Simpraga, S.; Linkenkaer-Hansen, K.; Bruining, H. (2019b): Behavioural and Neurophysiological Outcomes of the Bumetanide in Autism Medication and Biomarker (BAMBI) Trial 85 (10), S91-S92. Available online at https://www.cochranelibrary.com/central/doi/10.1002/central/CN-01998986/full.
    - van Marije Andel, D.; Sprengers, J.; Juarez-Martinez, E.; Simpraga, S.; Oranje, B.; Linkenkaer-Hansen, K. et al. (2019): Cognitive Outcomes of the Bumetanide in Autism Medication and Biomarker (BAMBI) Trial 85 (10), S92-. Available online at https://www.cochranelibrary.com/central/doi/10.1002/central/CN-01997821/full.

- Sprengers JJ, van Andel DM, Zuithoff NP, et al. Bumetanide for Core Symptoms of Autism Spectrum Disorder (BAMBI): A Single Center, Double-Blinded, Participant-Randomized, Placebo-Controlled, Phase Two, Superiority Trial. Journal of the American Academy of Child and Adolescent Psychiatry. 2020. doi:10.1016/j.jaac.2020.07.888.
- Juarez-Martinez EL; Sprengers JJ; Cristian G; Oranje B; van Andel DM; Avramiea AE et al. (2021): Prediction of behavioral improvement through resting-state EEG and clinical severity in a randomized controlled trial testing bumetanide in autism spectrum disorder. In *Biological psychiatry. Cognitive neuroscience and neuroimaging*. Available online at https://pubmed.ncbi.nlm.nih.gov/34506972/
- Sprengers, J.; van Andel, D.; Zuithoff, N.; Keijzer-Veen, M.; Annelien, S.; Scheepers, F. et al. (2020): P.130 Bumetanide versus placebo for core symptoms of autism spectrum disorder at 91 days (BAMBI): a single-centre, double-blinded, patient-randomized, placebo-controlled, phase-2-trial 40, S80-S81. Available online at https://www.cochranelibrary.com/central/doi/10.1002/central/CN-02212093/ful

38. Fahmy 2013[78, 79]
    - Fahmy SF, El-Hamamsy M, Zaki O, Badary OA. Effect of l-carnitine on behavioral disorder in autistic children. Value in health. 2013;16(3):A15.
    - Fahmy SF, El-Hamamsy MH, Zaki OK, Badary OA. L-Carnitine supplementation improves the behavioral symptoms in autistic children. Research in autism spectrum disorders. 2013;7(1):159–166.
39. Findling 1997[80]
    - Findling RL, Maxwell K, Scotese-Wojtila L, Huang J, Yamashita T, Wiznitzer M. High-dose pyridoxine and magnesium administration in children with autistic disorder: an absence of salutary effects in a double-blind, placebo-controlled study. Journal of autism and developmental disorders. 1997;27(4):467–478.
40. Frye 2018[81, 82]
    - Frye RE, Slattery J, Delhey L, et al. Folinic acid improves verbal communication in children with autism and language impairment: a randomized double-blind placebo-controlled trial. Molecular Psychiatry. 2018;23(2):247–256.
    - NCT01602016, Arkansas Children's Hospital Research, Institute, University of A. A Folinic Acid Intervention for Autism Spectrum Disorders. 2012.
41. Gabis 2019[83, 84]
    - Gabis LV, Ben-Hur R, Shefer S, Jokel A, Shalom DB. Improvement of Language in Children with Autism with Combined Donepezil and Choline Treatment. J Mol Neurosci. 2019.
    - NCT01098383, The Israeli Society of Clinical, Pediatrics, Sheba Medical C. Treatment With Acetyl-Choline Esterase Inhibitors in Children With Autism Spectrum Disorders. 2010.
42. Geier 2011[85, 86]
    - Geier DA, Kern JK, Davis G, et al. A prospective double-blind, randomized clinical trial of levocarnitine to treat autism spectrum disorders. Medical science monitor. 2011;17(6):Pi15-23.
    - ISRCTN54273114, Autism Research I. A Clinical Trial of Levocarnitine to Treat Autism Spectrum Disorders. 2010.
43. Ghanizadeh 2014[87, 88]
    - Ghanizadeh A, Sahraeizadeh A, Berk M. A head-to-head comparison of aripiprazole and risperidone for safety and treating autistic disorders, a randomized double blind clinical trial. Child psychiatry and human development. 2014;45(2):185–192.

- IRCT201110233930N15, Vice chancellor for research, Shiraz University of Medical sciences. Aripiprazole versus risperidone for treatment of autism. 2011.

44. Ghodsi 2018[89]
    - Ghodsi R, Kheirouri S, Nosrati R. Carnosine supplementation does not affect serum concentrations of advanced glycation and precursors of lipoxidation end products in autism: a randomized controlled clinical trial. Ann Clin Biochem. 2019;56(1):148–154.
45. Ghuman 2009[90]
    - Ghuman JK, Aman MG, Lecavalier L, et al. Randomized, placebo-controlled, crossover study of methylphenidate for attention-deficit/hyperactivity disorder symptoms in preschoolers with developmental disorders. Journal of Child & Adolescent Psychopharmacology. 2009;19(4):329–339
46. Gordon 1993[91, 92]
    - Gordon CT, Rapoport JL, Hamburger SD, State RC, Mannheim GB. Differential response of seven subjects with autistic disorder to clomipramine and desipramine. American journal of psychiatry. 1992;149(3):363–366.
    - Gordon CT, State RC, Nelson JE, Hamburger SD, Rapoport JL. A double-blind comparison of clomipramine, desipramine, and placebo in the treatment of autistic disorder. Archives of General Psychiatry. 1993;50(6):441–447.
47. Gringras 2017[93–99]
    - Findling RL, Gringras P, Nir T, Zisapel N. Short- and long-term prolonged release melatonin treatment for sleep disorders in children with autism spectrum disorders - Results of a phase III randomized clinical trial. Journal of the american academy of child and adolescent psychiatry. Conference: 64th annual meeting american academy of child and adolescent psychiatry, AACAP 2017. United states. 2017;56(10):S167.
    - Gringras P, Findling RL, Nir T, Zisapel N. Short and long term prolonged release melatonin treatment for sleep disorders in children with autism spectrum disorders: results of a phase III randomized clinical trial. Sleep medicine. Conference: 14th world sleep congress. Czech republic. 2017;40:e119.
    - Gringras P, Findling R, Nir T, Zisapel N. Short and long term prolonged release melatonin treatment for sleep disorders in children with autism spectrum disorders: results of a phase iii randomized clinical trial. Developmental medicine and child neurology. Conference: 44th annual conference of the british paediatric neurology association, BPNA 2018. United kingdom. 2017;59:27.
    - Gringras P, Nir T, Breddy J, Frydman-Marom A, Findling RL. Efficacy and Safety of Pediatric Prolonged-Release Melatonin for Insomnia in Children With Autism Spectrum Disorder. Journal of the American Academy of Child and Adolescent Psychiatry. 2017;56(11):948-957.e4.

    - EUCTR2006-004025-28-GB (2007): MENDS: the use of MElatonin in children with Neuro-developmental Disorders and impaired Sleep; a randomised, double-blind, placebo-controlled, parallel study - MENDS. Available online at https://www.cochranelibrary.com/central/doi/10.1002/central/CN-01833993/full.
    - Maras, A.; Schroder, C. M.; Malow, B. A.; Findling, R. L.; Breddy, J.; Nir, T. et al. (2018): Long-Term Efficacy and Safety of Pediatric Prolonged-Release Melatonin for Insomnia in Children with Autism Spectrum Disorder 28 (10), pp. 699–710. Available online at https://www.cochranelibrary.com/central/doi/10.1002/central/CN-02124428/full.
    - Schroder, C. M.; Malow, B. A.; Maras, A.; Melmed, R. D.; Findling, R. L.; Breddy, J. et al. (2019): Pediatric Prolonged-Release Melatonin for Sleep in Children with Autism Spectrum Disorder: impact on Child Behavior and Caregiver's Quality of Life 49 (8), pp. 3218–3230.

Available online at https://www.cochranelibrary.com/central/doi/10.1002/central/CN-01941953/full.
- Malow BA, Findling RL, Schroder CM, et al. Sleep, Growth, and Puberty After Two Years of Prolonged-Release Melatonin in Children With Autism Spectrum Disorder. Journal of the American Academy of Child and Adolescent Psychiatry. 2020.
- NCT01906866. Efficacy and Safety of Circadin® in the Treatment of Sleep Disturbances in Children With Neurodevelopment Disabilities. https://ClinicalTrials.gov/show/NCT01906866.
- Schroder CM, Malow BA, Maras A, et al. Pediatric Prolonged-Release Melatonin for Sleep in Children with Autism Spectrum Disorder: Impact on Child Behavior and Caregiver's Quality of Life. Journal of Autism & Developmental Disorders. 2019.

48. Guastella 2015[100–102]
    - ACTRN12609000513213, University University of S. A course of oxytocin nasal spray (OT) to treat social problems in youth with autism spectrum disorders. 2009.
    - Guastella AJ. A randomized controlled trial of oxytocin nasal spray to treat youth diagnosed with autism spectrum disorders. Biological Psychiatry. 2012;71(8):234s.
    - Guastella AJ, Gray KM, Rinehart NJ, et al. The effects of a course of intranasal oxytocin on social behaviors in youth diagnosed with autism spectrum disorders: a randomized controlled trial. Journal of child psychology and psychiatry, and allied disciplines. 2015;56(4):444–452.
49. Hage 2016 (EUCTR2004-003080-38-DE)[103, 104]
    - EUCTR2014-003080-38-DE, Behaviour, Radboud University Nijmegen Medical Centre, Donders Institute for Brain Cognition. Glutamatergic medication in the treatment of Obsessive Compulsive Disorder (OCD) and Autism Spectrum Disorder (ASD). 2014.
    - Häge A, Banaschewski T, Buitelaar JK, et al. Glutamatergic medication in the treatment of obsessive compulsive disorder (OCD) and autism spectrum disorder (ASD) - study protocol for a randomised controlled trial. Trials. 2016;17(1):141.
50. Handen 2000[105]
    - Handen BL, Johnson CR, Lubetsky M. Efficacy of methylphenidate among children with autism and symptoms of attention-deficit hyperactivity disorder. Journal of autism and developmental disorders. 2000;30(3):245–255.
51. Handen 2009[106, 107]
    - Handen BL, Melmed RD, Hansen RL, et al. A double-blind, placebo-controlled trial of oral human immunoglobulin for gastrointestinal dysfunction in children with autistic disorder. Journal of autism and developmental disorders. 2009;39(5):796–805.
    - NCT00110708, PediaMed P. Safety and Efficacy Study in the Treatment of Intestinal Problems Associated With Autism. 2005.
52. Handen 2012[108–111]
    - Handen, Benjamin L., Department of Psychiatry, Merck Program, Western Psychiatric Institute and Clinic, 3811 O'Hara St., Pittsburgh, PA, US, 15213, Johnson CR, McAuliffe-Bellin S, Hardan A. Safety and efficacy of Donepezil in children and adolescents with autism: Behavioral measures. Merrick, Joav [Ed]. 2012.
    - Handen, Benjamin L., Department of Psychiatry, Merck Program, Western Psychiatric Institute and Clinic, 3811 O'Hara St., Pittsburgh, PA, US, 15213, Johnson CR, McAuliffe-Bellin S, Hardan A. Safety and efficacy of donepezil in children and adolescents with autism: Behavioral measures. Zachor, Ditza A [Ed]. 2013.

- Handen BL, Johnson CR, McAuliffe-Bellin S, Murray PJ, Hardan AY. Safety and efficacy of donepezil in children and adolescents with autism: neuropsychological measures. Journal of Child & Adolescent Psychopharmacology. 2011;21(1):43–50.
- NCT00047697, National Institute of Mental, Health, University of P. Donepezil HCl & Cognitive Deficits in Autism. 2002.

53. Handen 2015[112–118]
    - Handen BL, Aman MG, Arnold LE, et al. Atomoxetine, Parent Training, and Their Combination in Children With Autism Spectrum Disorder and Attention-Deficit/Hyperactivity Disorder. Journal of the American Academy of Child and Adolescent Psychiatry. 2015;54(11):905–915.
    - Arnold LE, Ober N, Aman MG, et al. A 1.5-Year Follow-Up of Parent Training and Atomoxetine for Attention-Deficit/Hyperactivity Disorder Symptoms and Noncompliant/Disruptive Behavior in Autism. Journal of Child and Adolescent Psychopharmacology;28(5):322–330.
    - Hollway JA, Aman MG, Mendoza-Burcham MI, et al. Caregiver Satisfaction with a Multisite Trial of Atomoxetine and Parent Training for Attention-Deficit/Hyperactivity Disorder and Behavioral Noncompliance in Children with Autism Spectrum Disorder. Journal of Child and Adolescent Psychopharmacology. 2016;26(9):807–814.
    - Hollway JA, Mendoza-Burcham M, Andridge R, et al. Atomoxetine, Parent Training, and Their Effects on Sleep in Youth with Autism Spectrum Disorder and Attention-Deficit/Hyperactivity Disorder. Journal of Child and Adolescent Psychopharmacology. 2018;28(2):130–135.
    - Lecavalier L, Pan X, Smith T, et al. Parent Stress in a Randomized Clinical Trial of Atomoxetine and Parent Training for Children with Autism Spectrum Disorder. Journal of autism and developmental disorders;48(4):980–987.
    - NCT00844753, University of P, Ohio State U, University of R. Atomoxetine, Placebo and Parent Management Training in Autism. 2008.
    - Tumuluru RV, Corbett-Dick P, Aman MG, et al. Adverse Events of Atomoxetine in a Double-Blind Placebo-Controlled Study in Children with Autism. Journal of Child and Adolescent Psychopharmacology. 2017;27(8):708–714.
54. Hardan 2012[119, 120]
    - Hardan AY, Fung LK, Libove RA, et al. A randomized controlled pilot trial of oral N-acetylcysteine in children with autism. Biological Psychiatry. 2012;71(11):956–961.
    - NCT00627705, Stanford U. A Study of N-Acetyl Cysteine in Children With Autism. 2008.
55. Harfterkamp 2013[121–127]
    - Harfterkamp M, Buitelaar JK, Minderaa RB, Loo-Neus G, Gaag RJ, Hoekstra PJ. Long-term treatment with atomoxetine for attention-deficit/hyperactivity disorder symptoms in children and adolescents with autism spectrum disorder: an open-label extension study. Journal of Child & Adolescent Psychopharmacology. 2013;23(3):194–199.
    - Harfterkamp M, Buitelaar JK, Minderaa RB, Loo-Neus G, Gaag RJ, Hoekstra PJ. Atomoxetine in autism spectrum disorder: no effects on social functioning; some beneficial effects on stereotyped behaviors, inappropriate speech, and fear of change. Journal of Child & Adolescent Psychopharmacology. 2014;24(9):481–485.
    - Harfterkamp M, Loo-Neus G, Minderaa RB, et al. A randomized double-blind study of atomoxetine versus placebo for attention-deficit/hyperactivity disorder symptoms in children with autism spectrum disorder. Journal of the American Academy of Child and Adolescent Psychiatry. 2012;51(7):733–741.

- Harfterkamp M, van der Meer J. A Randomized double-blind study of atomoxetine vs. placebo followed by an open label extension period of treatment with atomoxetine for ADHD symptoms in children with ASD. European Child and Adolescent Psychiatry;1:S216-S217.
- Harfterkamp M, van der Meer D, van der Loo-Neus G, Buitelaar JK, Minderaa RB, Hoekstra PJ. No evidence for predictors of response to atomoxetine treatment of attention-deficit/hyperactivity disorder symptoms in children and adolescents with autism spectrum disorder. Journal of Child and Adolescent Psychopharmacology. 2015;25(4):372–375.
- Meer JM, Harfterkamp M, Loo-Neus G, et al. A randomized, double-blind comparison of atomoxetine and placebo on response inhibition and interference control in children and adolescents with autism spectrum disorder and comorbid attention-deficit/hyperactivity disorder symptoms. Journal of Clinical Psychopharmacology. 2013;33(6):824–827.
- NCT00380692, Company, Eli L. Atomoxetine Versus Placebo for Symptoms of Attention-Deficit/Hyperactivity Disorder (ADHD) in Children and Adolescents With Autism Spectrum Disorder. 2006.

56. Hellings 2005[128, 129]
    - NCT00065884, Human D, National Institute of Mental, Health, Eunice Kennedy Shriver National Institute of Child, Health. Valproate Response in Aggressive Autistic Adolescents. 2003.
    - Hellings JA, Weckbaugh M, Nickel EJ, et al. A double-blind, placebo-controlled study of valproate for aggression in youth with pervasive developmental disorders. Journal of Child & Adolescent Psychopharmacology. 2005;15(4):682–692.
57. Hellings 2006[130–132]
    - Hellings JA, Cardona AM, Schroeder SR. Long-term safety and adverse events of risperidone in children, adolescents, and adults with pervasive developmental disorders. Journal of Mental Health Research in Intellectual Disabilities. 2010;3(3):132–144.
    - Hellings JA, Zarcone JR, Cr, all K, Wallace D, Schroeder SR. Weight gain in a controlled study of risperidone in children, adolescents and adults with mental retardation and autism. Journal of Child & Adolescent Psychopharmacology. 2001;11(3):229–238.
    - Hellings JA, Zarcone JR, Reese RM, et al. A crossover study of risperidone in children, adolescents and adults with mental retardation. Journal of autism and developmental disorders. 2006;36(3):401–411.
58. Hendren 2016[133–135]
    - Hendren RL, James SJ, Widjaja F, Lawton B, Rosenblatt A, Bent S. Randomized, Placebo-Controlled Trial of Methyl B12 for Children with Autism. Journal of Child and Adolescent Psychopharmacology. 2016;26(9):774–783.
    - NCT01039792, University of California D, Arkansas Children's Hospital Research, Institute, University of California, San Francisco. Trial of Methyl B12 on Behavioral and Metabolic Measures in Children With Autism. 2009.
    - Widjaja F, James SJ, Hendren RL. Double-blind placebo controlled trial of methyl B12 on behavioral and metabolic measures in children with autism. Neuropsychiatrie de l'Enfance et de l'Adolescence. 2012;60(5):S221
59. Herscu 2019[136, 137]
    - Herscu P, Handen BL, Arnold LE, et al. The SOFIA Study: Negative Multi-center Study of Low Dose Fluoxetine on Repetitive Behaviors in Children and Adolescents with Autistic Disorder. Journal of Autism & Developmental Disorders. 2019.
    - NCT00515320, Autism S, Neuropharm. Study of Fluoxetine in Autism. 2007.
60. Hollander 2006[138, 139]

- Anagnostou E, Esposito K, Soorya L, et al. Divalproex versus placebo for the prevention of irritability associated with fluoxetine treatment in autism spectrum disorder [11]. Journal of Clinical Psychopharmacology;26(4):444–446.
- Hollander E, Soorya L, Wasserman S, Esposito K, Chaplin W, Anagnostou E. Divalproex sodium vs. placebo in the treatment of repetitive behaviours in autism spectrum disorder. The international journal of neuropsychopharmacology. 2006;9(2):209–213.

61. Hollander 2005[140–142]
    - Hollander E, Phillips A, Chaplin W, et al. A placebo controlled crossover trial of liquid fluoxetine on repetitive behaviors in childhood and adolescent autism. Neuropsychopharmacology. 2005;30(3):582–589.
    - Hollander E, Swanson E, Anagnostou E, Phillips A, Chaplin W, Wasserman S. Liquid fluoxetine versus placebo for repetitive behaviors in childhood autism. Cummings, Jeffrey L [Ed]. 2006.
    - NCT00004486, Mount Sinai School of, Medicine. Randomized Study of Fluoxetine in Children and Adolescents With Autism. 1999.
62. Hollander 2006b[143]
    - Hollander E, Wasserman S, Swanson EN, et al. A double-blind placebo-controlled pilot study of olanzapine in childhood/adolescent pervasive developmental disorder. Journal of Child & Adolescent Psychopharmacology. 2006;16(5):541–548.
63. Hollander 2010[144, 145]
    - Hollander E, Chaplin W, Soorya L, et al. Divalproex sodium vs placebo for the treatment of irritability in children and adolescents with autism spectrum disorders. Neuropsychopharmacology. 2010;35(4):990–998.
    - NCT00211757, National Institute of Neurological, Disorders, Stroke, Montefiore Medical C. Divalproex Sodium vs. Placebo in Childhood/Adolescent Autism. 2005.
64. Hollander 2012[146–149]
    - Hollander E. A double-blind placebo-controlled trial of fluoxetine for repetitive behaviors and global severity in adult autism spectrum disorders (American Journal of Psychiatry (2012) 169 (292-299)). American journal of psychiatry. 2012;169(5):540.
    - Hollander E, Soorya L, Chaplin W, et al. A double-blind placebo-controlled trial of fluoxetine for repetitive behaviors and global severity in adult autism spectrum disorders. American journal of psychiatry. 2012;169(3):292–299.
    - Hollander E, Soorya L, Chaplin W, et al. A double-blind placebo-controlled trial of fluoxetine for repetitive behaviors and global severity in adult spectrum disorders" Correction. The American Journal of Psychiatry. 2012;169(5):540.
    - NCT00027404, Mount Sinai School of, Medicine. Study of Fluoxetine in Adults With Autistic Disorder. 2001.
65. Ichikawa 2017[150–153]
    - Ichikawa H, Mikami K, Okada T, et al. Aripiprazole in the Treatment of Irritability in Children and Adolescents with Autism Spectrum Disorder in Japan: A Randomized, Double-blind, Placebo-controlled Study. Child psychiatry and human development. 2017;48(5):796–806.
    - JPRN-JapicCTI-121862, Otsuka Pharmaceutical Co L. A short treatment study of aripiprazole in pediatric patients with Autistic Disorder. 2012.
    - JPRN-JapicCTI-121863, Otsuka Pharmaceutical Co L. A Long-term, Extended Treatment Study of Aripiprazole in Pediatric Patients With Autistic Disorder. 2012.
    - NCT01617447, Otsuka Pharmaceutical Co L. A Short Treatment Study of Aripiprazole in Pediatric Patients With Autistic Disorder. 2012.

- EUCTR2016-005111-40-Outside-EU/EEA (2017): Study of Aripiprazole in the Treatment of Serious Behavioral Problems in Children and Adolescents With Autistic Disorder (AD). Available online at https://www.cochranelibrary.com/central/doi/10.1002/central/CN-01805218/full.

66. ISRCTN20233876[155]
    - ISRCTN20233876, Indywidualna Specjalistyczna Praktyka Lekarska w Miejscu, Wezwania. Benefits of polyunsaturated fatty acid (PUFA) supplementation in therapy of children and teenagers with Asperger?s Syndrome. 2012.
67. Kent 2013[156–159]
    - Kent JM, Hough D, Singh J, Karcher K, P, ina G. An open-label extension study of the safety and efficacy of risperidone in children and adolescents with autistic disorder. Journal of Child & Adolescent Psychopharmacology. 2013;23(10):676–686.
    - Kent JM, Kushner S, Ning X, et al. Risperidone dosing in children and adolescents with autistic disorder: a double-blind, placebo-controlled study. Journal of autism and developmental disorders. 2013;43(8):1773–1783.
    - EUCTR2015-001220-31-Outside-EU/EEA, amp, Johnson Pharmaceutical R, Development LLC, Johnson. A Study of the Effectiveness and Safety of Two Doses of Risperidone in the Treatment of Children and Adolescents With Autistic Disorder. 2015.
    - NCT00576732, amp, Johnson Pharmaceutical R, Development LLC, Johnson. A Study of the Effectiveness and Safety of Two Doses of Risperidone in the Treatment of Children and Adolescents With Autistic Disorder. 2007.
68. Kerley 2017[160, 161]
    - Kerley CP, Power C, Gallagher L, Coghlan D. Lack of effect of Vitamin D 3 supplementation in autism: a 20-week, placebo-controlled RCT. Archives of disease in childhood. 2017;102(11):1030–1036.
    - NCT02508922, The National Children's Hospital, Tallaght, University of Dublin, Trinity College. Trial of Vitamin D3 Supplementation in Paediatric Autism. 2015.
    - Kerley, C. P.; Elnazir, B.; Greally, P.; Coghlan, D. (2018): Blunted serum 25(OH)D response to vitamin D3 supplementation in children with autism. Available online at https://www.cochranelibrary.com/central/doi/10.1002/central/CN-01652724/full.
69. Kern 2001[162]
    - Kern JK, Miller VS, Cauller PL, Kendall PR, Mehta PJ, Dodd M. Effectiveness of N,N-dimethylglycine in autism and pervasive developmental disorder. Journal of child neurology. 2001;16(3):169–173.
70. King 2001[163]
    - King BH, Wright DM, H, et al. Double-blind, placebo-controlled study of amantadine hydrochloride in the treatment of children with autistic disorder. Journal of the American Academy of Child and Adolescent Psychiatry. 2001;40(6):658–665.
71. King 2009[164–170]
    - King BH, Dukes K, Donnelly CL, et al. Baseline factors predicting placebo response to treatment in children and adolescents with autism spectrum disorders: a multisite randomized clinical trial. JAMA pediatrics. 2013;167(11):1045–1052.
    - King BH, Holl, er E, et al. Lack of efficacy of citalopram in children with autism spectrum disorders and high levels of repetitive behavior: citalopram ineffective in children with autism. Archives of General Psychiatry. 2009;66(6):583–590.
    - King BH, Holl, er E, et al. Factors influencing placebo response in the STAART citalopram trial. Annals of Neurology. 2010;14:S118.

- Scahill L, McCracken JT, Bearss K, et al. Design and subject characteristics in the federally-funded citalopram trial in children with pervasive developmental disorders. Journal of autism and developmental disorders. 2012;42(3):432–440.
- Volkmar FR. Citalopram treatment in children with autism spectrum disorders and high levels of repetitive behavior. Archives of General Psychiatry. 2009;66(6):581–582.
- NCT00086645, National Institute of Mental, Health, Boston U. Citalopram for Children With Autism and Repetitive Behavior (STAART Study 1). 2004.
- Anonymous. Citalopram ineffective for reducing repetitive behavior in autism spectrum disorders. Journal of the National Medical Association;101(9):976.

72. Klaiman 2013[171, 172]
    - Klaiman C, Huffman L, Masaki L, Elliott GR. Tetrahydrobiopterin as a treatment for autism spectrum disorders: a double-blind, placebo-controlled trial. Journal of Child & Adolescent Psychopharmacology. 2013;23(5):320–328.
    - NCT00850070, BioMarin P, The Children's Health C. Sapropterin as a Treatment for Autistic Disorder. 2009.
73. Kolmen 1997[173–175]
    - Feldman HM, Kolmen BK, Gonzaga AM. Naltrexone and communication skills in young children with autism. Journal of the American Academy of Child and Adolescent Psychiatry. 1999;38(5):587–593.
    - Kolmen BK, Feldman HM, H, en BL, Janosky JE. Naltrexone in young autistic children: a double-blind, placebo-controlled crossover study. Journal of the American Academy of Child and Adolescent Psychiatry. 1995;34(2):223–231.
    - Kolmen BK, Feldman HM, H, en BL, Janosky JE. Naltrexone in young autistic children: replication study and learning measures. Journal of the American Academy of Child and Adolescent Psychiatry. 1997;36(11):1570–1578.
74. Kosaka 2016[176, 177]
    - Kosaka H, Okamoto Y, Munesue T, et al. Oxytocin efficacy is modulated by dosage and oxytocin receptor genotype in young adults with high-functioning autism: a 24-week randomized clinical trial. Translational psychiatry. 2016;6(8):e872.
    - JPRN-UMIN000005211, Department of Neuropsychiatry, Faculty of Medical Sciences University of Fukui. A research of therapy evaluation to prosocial behavior after intranasal oxytocin administration. 2011.
75. Lamberti 2016[178]
    - Lamberti M, Siracusano R, Italiano D, et al. Head-to-Head Comparison of Aripiprazole and Risperidone in the Treatment of ADHD Symptoms in Children with Autistic Spectrum Disorder and ADHD: a Pilot, Open-Label, Randomized Controlled Study. Paediatric drugs. 2016;18(4):319–329.
76. Lemonnier 2012[179–183]
    - Hadjikhani N, Åsberg Johnels J, Lassalle A, et al. Bumetanide for autism: more eye contact, less amygdala activation. Scientific reports. 2018;8(1):3602. doi:10.1038/s41598-018-21958-x.
    - Hadjikhani N, Zurcher NR, Rogier O, et al. Improving emotional face perception in autism with diuretic bumetanide: a proof-of-concept behavioral and functional brain imaging pilot study. Autism. 2015;19(2):149–157. doi:10.1177/1362361313514141.
    - Lemonnier E, Degrez C, Phelep M, et al. A randomised controlled trial of bumetanide in the treatment of autism in children. Translational psychiatry. 2012;2:e202.

- EUCTR2009-010393-38-FR, BREST, C. H. U. de. ETUDE DE L'EFFICACITE D'UN TRAITEMENT PAR BUMETANIDE DANS UNE POPULATION D'ENFANTS AUTISTES. 2009.
- NCT01078714, University Hospital B. Efficiency of Bumetanide in Autistic Children. 2010.

77. Lemonnier 2017[184, 185]
    - Lemonnier E, Villeneuve N, Sonie S, et al. Effects of bumetanide on neurobehavioral function in children and adolescents with autism spectrum disorders.[Erratum appears in Transl Psychiatry. 2017 May 9;7(5):e1124; PMID: 28485727]. Transl Psychiatry Psychiatry. 2017;7(3):e1056.
    - EUCTR2013-003259-39-ES, Neurochlore. Study in children and adolescents with autism. 2014.
    - Falissard, B.; Severo, C. A.; Lambert, E.; Crutel, V.; Kyaga, S.; Serret, S. et al. (2019): P.809 Correlation between childhood autism rating scale 2 and clinical global impression improvement. In European neuropsychopharmacology 29, S538-S539. Available online at https://www.cochranelibrary.com/central/doi/10.1002/central/CN-02051752/full.
78. Leventhal 1993[186]
    - Leventhal BL, Cook EH, Morford M, Ravitz AJ, Heller W, Freedman DX. Clinical and neurochemical effects of fenfluramine in children with autism. Journal of neuropsychiatry and clinical neurosciences. 1993;5(3):307–315.
79. Levine 1997[187]
    - Levine J, Aviram A, Holan A, Ring A, Barak Y, Belmaker RH. Inositol treatment of autism. Journal of neural transmission. 1997;104(2):307–310.
80. *Li 2016*[188] (the study was not included in the quantitative analysis, due to serious concerns on the methodology, e.g. it was published in a Chinese journal, the male to female ratio was close to one, and effect sizes of the comparison between paliperidone and aripiprazole were suspiciously large; no response from the author)
    - Li YC, Ma J, Xu HM, Yang GY, Zhang JC. Efficacy and safety of paliperidone and aripiprazole in the treatment of autism. [Chinese]. Chinese Journal of New Drugs. 30;25(16):1893–1897.
81. Liu 2019[189, 190]
    - ACTRN12616001002471, Yu-Yu W, Yen-Wenn L. Lactobacillus plantarum PS128 on behavior activity of children with autism. 2016.
    - Liu Y-W, Liong MT, Chung Y-CE, et al. Effects of Lactobacillus plantarum PS128 on Children with Autism Spectrum Disorder in Taiwan: A Randomized, Double-Blind, Placebo-Controlled Trial. Nutrients. 2019;11(4). doi:10.3390/nu11040820.
82. Loebel 2016[191, 192]
    - Loebel A, Brams M, Goldman RS, et al. Lurasidone for the Treatment of Irritability Associated with Autistic Disorder. Journal of autism and developmental disorders. 2016;46(4):1153–1163.
    - NCT01911442, Sunovion. Lurasidone Pediatric Autism Study. 2013.
83. Malone 2001[193]
    - Malone RP, Cater J, Sheikh RM, Choudhury MS, Delaney MA. Olanzapine versus haloperidol in children with autistic disorder: an open pilot study. Journal of the American Academy of Child and Adolescent Psychiatry. 2001;40(8):887–894.
84. Malone 2010[194, 195]
    - Malone RP, West SH, Ghaffari M, et al. Metabolic effects of olanzapine in children with autistic disorder. Journal of Child and Adolescent Psychopharmacology;20:531–532.

- Ghaffari M, West SH, Malone RP, et al. The effects of olanzapine on QTc in children with autistic disorder. Journal of Child and Adolescent Psychopharmacology;20:532.
- NCT00183404, National Institute of Mental, Health, Drexel U. Long-Term Olanzapine Treatment in Children With Autism. 2005.

85. Mankad 2015[196, 197]
    - Mankad D, Dupuis A, Smile S, et al. A randomized, placebo controlled trial of omega-3 fatty acids in the treatment of young children with autism. Mol Autism. 2015;6:18.
    - NCT01248728, Holl, Bloorview Kids Rehabilitation H, The Hospital for Sick, Children, Evdokia A. Omega-3 Fatty Acids For Treatment Of Young Children With Autism (OMG). 2010.
86. Marcus 2009[198–203]
    - Benton TD. Aripiprazole to treat irritability associated with autism: a placebo-controlled, fixed-dose trial. Current psychiatry reports. 2011;13(2):77–79.
    - Mankoski R, Stockton G, Manos G, et al. Aripiprazole treatment of irritability associated with autistic disorder and the relationship between prior antipsychotic exposure, adverse events, and weight change. Journal of Child & Adolescent Psychopharmacology. 2013;23(8):572–576.
    - Marcus RN, Owen R, Kamen L, et al. A placebo-controlled, fixed-dose study of aripiprazole in children and adolescents with irritability associated with autistic disorder. Journal of the American Academy of Child and Adolescent Psychiatry. 2009;48(11):1110–1119.
    - Marcus RN, Owen R, Manos G, Mankoski R, Kamen L, McQuade RD. Aripiprazole in the treatment of irritability in pediatric patients (Aged 6-17 Years) with autistic disorder: results from a 52-week, open-label study. Journal of Child & Adolescent Psychopharmacology. 2011;21(3):229–236.
    - Marcus RN, Owen R, Manos G, et al. Safety and tolerability of aripiprazole for irritability in pediatric patients with autistic disorder: a 52-week, open-label, multicenter study. Journal of clinical psychiatry. 2011;72(9):1270–1276.
    - NCT00337571, amp, Commercialization I, Otsuka America P, Otsuka Pharmaceutical D. Study of Aripiprazole in the Treatment of Children and Adolescents With Autistic Disorder (AD). 2006.
87. Martineuau 1985[204–206]
    - Barthelemy C, Garreau B, Leddet I, et al. Biological and clinical effects of oral magnesium and associated magnesium-vitamin B6 administration on certain disorders observed in infantile autism. [French] Effets Cliniques Et Biologiques De L’administration Orale Du Magnesium Seul Ou Du Magnesium Associe a La Vitamine B6 Sur Certains Troubles Observes Dans L’autisme Infantile. Therapie. 1980;35(5):627–632.
    - Martineau J, Barthelemy C, Garreau B, Lelord G. Vitamin B6, magnesium, and combined B6-Mg: therapeutic effects in childhood autism. Biological Psychiatry. 1985;20(5):467–478.
    - Martineau J, Barthelemy C, Roux S, Garreau B, Lelord G. Electrophysiological effects of fenfluramine or combined vitamin B6 and magnesium on children with autistic behaviour. Developmental Medicine & Child Neurology. 1989;31(6):721–727
88. Martsenkovska 2015[207, 208] (the reported data could not be used in the meta-analysis, only GAF scores for risperidone arm were reported; no response from the author)
    - Martsenkovska I. Risperidone and atomoxetine in the treatment of severe and challenging behaviours in children with pervasive developmental disorders. European neuropsychopharmacology. 2015;25:S649.

- Martsenkovsky I, Martsenkovska I, Martsenkovskyi D. Risperidon and atomoxetine in the treatment of several and challending behaviors in children with PDD. European Psychiatry. 2015;30:195.

89. *Martsenkovsky 2014*[209] (excluded from the quantitative analysis due to reporting of trials resuls in a conference abstract and there are serious concerns on the methodology, e.g. standard deviations are smaller than expected and seems not to be standard errors; no response from study author)
    - Martsenkovsky I. Divalproex sodium and risperidone in the treatment of cognitive, behavioral and social dysfunction in preschool children with PDD and ADHD. European neuropsychopharmacology;2:S721-S722.
90. Mazahery 2016[210–214]
    - ACTRN12615000144516, Waitemata District Health B, Massey U. Omega-3, vitamin D and Autism in children. 2015.
    - Mazahery H, Conlon C, Beck KL, et al. Vitamin D and omega-3 fatty acid supplements in children with autism spectrum disorder: a study protocol for a factorial randomised, double-blind, placebo-controlled trial. Trials. 2016;17(1):295.
    - Mazahery H, Conlon CA, Beck KL, et al. A randomised controlled trial of vitamin D and omega-3 long chain polyunsaturated fatty acids in the treatment of irritability and hyperactivity among children with autism spectrum disorder. J Steroid Biochem Mol Biol. 2019;187:9–16.
    - Mazahery H, Conlon CA, Beck KL, et al. A Randomised-Controlled Trial of Vitamin D and Omega-3 Long Chain Polyunsaturated Fatty Acids in the Treatment of Core Symptoms of Autism Spectrum Disorder in Children. Journal of Autism & Developmental Disorders. 2019;49(5):1778–1794.
    - Mazahery H, Conlon CA, Beck KL, et al. Inflammation (IL-1β) Modifies the Effect of Vitamin D and Omega-3 Long Chain Polyunsaturated Fatty Acids on Core Symptoms of Autism Spectrum Disorder-An Exploratory Pilot Study‡. Nutrients. 2020;12(3).
91. McDougle 1996[215–217]
    - Huffman GB. Fluvoxamine for the treatment of autistic disorders in adults. American Family Physician. 1997;55(4):1375–1376.
    - McDougle CJ, Naylor ST, Cohen DJ, Volkmar FR, Heninger GR, Price LH. A double-blind, placebo-controlled study of fluvoxamine in adults with autistic disorder. Archives of General Psychiatry. 1996;53(11):1001–1008.
    - Vegso SJ. A double-blind placebo-controlled study of fluvoxamine in treating the symptoms of autism. 1995.
92. McDougle 1998[218]
    - McDougle CJ, Holmes JP, Carlson DC, Pelton GH, Cohen DJ, Price LH. A double-blind, placebo-controlled study of risperidone in adults with autistic disorder and other pervasive developmental disorders. Archives of General Psychiatry. 1998;55(7):633–641.
93. McDougle 2000[219] (this unpublished study was reported in the cited review)
    - McDougle C, Le Kresch, Posey DJ. Repetitive Thoughts and Behavior in Pervasive Developmental Disorders: Treatment with Serotonin Reuptake Inhibitors. J Autism Dev Disord. 2000;30(5):427–435.
94. Mehrazad-Saber 2018[220–222]
    - IRCT2016061711689N4, Technology Of Tabriz University Of Medical Science, Nutritional Science Resea, Deputy of r. Effects Of Carnosine On Autism. 2016.
    - IRCT2016061711689N5, technology of Tabriz university of medical, science, Deputy of r. Effects of carnosine supplementation on autism disorder. 2016.

- Mehrazad-Saber Z, Kheirouri S, Noorazar SG. Effects of l-Carnosine Supplementation on Sleep Disorders and Disease Severity in Autistic Children: A Randomized, Controlled Clinical Trial. Basic Clin Pharmacol Toxicol. 2018;123(1):72–77.

95. Miral 2008[223]
    - Miral S, Gencer O, Inal-Emiroglu FN, Baykara B, Baykara A, Dirik E. Risperidone versus haloperidol in children and adolescents with AD : a randomized, controlled, double-blind trial. European child & adolescent psychiatry. 2008;17(1):1–8.
96. Moradi 2018[224–226]
    - IRCT20150519022323N2, Ferdowsi University Of Mashhad, Mashhad. The effect of Perceptual motor Exercises along with music and Vitamin D3 Supplementation in children with autism spectrum disorder. 2017.
    - Moradi H, Sohrabi M, Taheri H, Khodashenas E, Movahedi A. The effects of different combinations of perceptual-motor exercises, music, and vitamin D supplementation on the nerve growth factor in children with high-functioning autism. Complementary Therapies in Clinical Practice. 2018;31:139–145.
    - Moradi H, Sohrabi M, Taheri H, Khodashenas E, Movahedi A. Comparison of the effects of perceptual-motor exercises, vitamin D supplementation and the combination of these interventions on decreasing stereotypical behavior in children with autism disorder. International Journal of Developmental Disabilities. 2020;66(2):122–132. doi:10.1080/20473869.2018.1502068.
97. Munasinghe 2010[227]
    - Munasinghe SA, Oliff C, Finn J, Wray JA. Digestive enzyme supplementation for autism spectrum disorders: a double-blind randomized controlled trial. Journal of autism and developmental disorders. 2010;40(9):1131–1138.
98. Munesue 2016[228–230]
    - Higashida H, Munesue T, Kosaka H, Yamasue H, Yokoyama S, Kikuchi M. Social Interaction Improved by Oxytocin in the Subclass of Autism with Comorbid Intellectual Disabilities. Diseases. 2019;7(1).
    - JPRN-UMIN000007250, Research Center for Child Mental Development, Kanazawa University. A randomized, double-blind, placebo-controlled, cross-over trial of oxytocin in patients with autism spectrum disorder. 2012.
    - Munesue T, Nakamura H, Kikuchi M, et al. Oxytocin for male subjects with autism spectrum disorder and comorbid intellectual disabilities: a randomized pilot study. Frontiers in psychiatry. 2016;7.
99. Nagaraj 2006[231]
    - Nagaraj R, Singhi P, Malhi P. Risperidone in children with autism: randomized, placebo-controlled, double-blind study. Journal of child neurology. 2006;21(6):450–455.
100. NCT00057408[232]
    - NCT00057408, National Institute of Mental, Health, Development, F. D. A. Office of Orphan Products. A Controlled Study of Olanzapine in Children With Autism. 2003.
101. NCT00166621[233]
    - NCT00166621, Chugani DC. Early Pharmacotherapy Aimed at Neuroplasticity in Autism: Safety and Efficacy. 2005.
102. NCT00183339[234]
    - NCT00183339, National Institute of Mental, Health, University of North Carolina, Chapel Hill. Early Intervention With Fluoxetine in Autism. 2005
103. NCT00198107[236]

- NCT00198107, National Institute of Mental, Health, Indiana U. Evaluating the Effectiveness of Aripiprazole and D-Cycloserine to Treat Symptoms Associated With Autism. 2005.

104. NCT00198120[237]
    - NCT00198120, National Institute of Mental, Health, National Alliance for Research on, Schizophrenia, Depression, Indiana University School of, Medicine, Indiana U. Safety and Effectiveness of D-Cycloserine in Children With Autism. 2005.
105. NCT00252603[238]
    - NCT00252603, Dentistry of New J, National Alliance for Autism, Research, University of M. Galantamine Versus Placebo in Childhood Autism. 2005.
106. NCT00468130[239]
    - NCT00468130, Dentistry of New J, University of M. Efficacy of Aripiprazole Versus Placebo in the Reduction of Aggressive and Aberrant Behavior in Autistic Children. 2007.
107. NCT00498173[240]
    - NCT00498173, National Institute of Mental, Health, Massachusetts General H. Effectiveness of Atomoxetine in Treating ADHD Symptoms in Children and Adolescents With Autism. 2007.
108. NCT00572741[241]
    - NCT00572741, Arkansas Children's Hospital Research, Institute. Treating Oxidative Stress and the Metabolic Pathology of Autism. 2007.
109. NCT00609531[242]
    - NCT00609531, National Institute of Mental, Health, University of North Carolina, Chapel Hill. Functional MRI Evaluation of the Effect of Citalopram in Autism Spectrum Disorders. 2008.
110. NCT00655174[243]
    - NCT00655174, The Hospital for Sick, Children. Fluvoxamine and Sertraline in Childhood Autism - Does SSRI Therapy Improve Behaviour and/or Mood? 2008.
111. NCT00672360[244]
    - NCT00672360, Baylor College of M. Folate Rechallenge. 2008.
112. NCT00870727[245]
    - NCT00870727, National Institute of Mental, Health, Bristol-Myers S, Indiana U. Study of Aripiprazole in the Treatment of Pervasive Developmental Disorders. 2009.
113. NCT00881452[246, 247]
    - M. F. Heil, D. A. Pearson, R. Hendren, S. R. Raines and J. Fallon, ed. Pancreatic Replacement Therapy with CM-at Is Associated with Reduction in Maladaptive Behaviors in Preschoolers with Autism.; 2019.
    - NCT00881452, Curemark. A Trial of CM-AT in Children With Autism. 2009.
114. NCT01171937[248]
    - NCT01171937, Eunice Kennedy Shriver National Institute of Child, Health, Human D, University of California, Los Angeles. Risperidone Treatment In Children With Autism Spectrum Disorder And High Levels Of Repetitive Behavior. 2010.
115. NCT01230359[249]
    - NCT01230359, Qatar U, Heidelberg U, Hamad Medical C. Early Nutritional Intervention in Patients With Autism Spectrum Disorders. 2010.
116. NCT01260961[250]
    - NCT01260961, Rutgers, The State University of New Jersey. Developing Treatment, Treatment Validation and Treatment Scope in the Setting of an Autism Clinical Trial. 2010.
117. NCT01302964[251]

- NCT01302964, Autism S, Massachusetts General H. Mirtazapine Treatment of Anxiety in Children and Adolescents With Pervasive Developmental Disorders. 2010.

118. Castejon 2021 (NCT01366859)[252]
    - NCT01366859, Immunotec I, Nova Southeastern U. Nutritional Intervention in Children With Autism Using Whey Protein (Immunocal): Impact on Core Areas of Behavior. 2011
    - Castejon, A. M., J. A. Spaw, I. Rozenfeld, N. Sheinberg, S. Kabot, A. Shaw, P. Hardigan, R. Faillace and E. E. Packer (2021). "Improving Antioxidant Capacity in Children With Autism: A Randomized, Double-Blind Controlled Study With Cysteine-Rich Whey Protein." Frontiers in Psychiatry **12**(1548).
119. NCT01372449[253, 2531]
    - NCT01372449, Icahn School of Medicine at Mount, Sinai, Rush University Medical C, Nationwide Children's H, Evdokia A. A Multi-site Double-blind Placebo-controlled Trial of Memantine Versus Placebo in Children With Autism (MEM). 2011.
    - Soorya, Latha Valluripalli; Fogg, Louis; Ocampo, Edith; Printen, Madison; Youngkin, Sarah; Halpern, Danielle et al. (2021): Neurocognitive Outcomes from Memantine: A Pilot, Double-Blind, Placebo-Controlled Trial in Children with Autism Spectrum Disorder. In *Journal of Child and Adolescent Psychopharmacology* 31 (7), pp. 475–484. DOI: 10.1089/cap.2021.0010
120. NCT01661855[254]
    - NCT01661855, Holl, Bloorview Kids Rehabilitation H, et al. A Pilot Study of Riluzole Versus Placebo in the Treatment of Children and Adolescents With ASD. 2012.
121. NCT01745497[255, 256]
    - NCT01745497, Autism Treatment N, Massachusetts General H, et al. Iron Treatment of Sleep Disorders in Children With Autism Spectrum Disorder. 2012.
    - Reynolds AM, Connolly HV, Katz T, et al. Randomized, Placebo-Controlled Trial of Ferrous Sulfate to Treat Insomnia in Children With Autism Spectrum Disorders. Pediatric Neurology. 2020;104:30–39. doi:10.1016/j.pediatrneurol.2019.07.015.
122. NCT01788072[257, 258]
    - NCT01788072, Holl, Bloorview Kids Rehabilitation H, McMaster U, St. Michael's Hospital T, Evdokia A. INtranasal OXyTocin for the Treatment of Autism Spectrum Disorders. 2013.
    - E. Anagnostou, J. A. Brian, J. Goldberg, S. Prat, L. Capano, A. Iaboni, A. Solish, D. Frisch, R. Hastie Adams, L. Genore, I. Patriciu, F. Tran and M. Woodbury-Smith, ed. A Phase 2 Randomized, Placebo-Controlled Trial of Intranasal Oxytocin in Adults with Autism Spectrum Disorder; INSAR 2019
123. NCT01908205[259, 260]
    - NCT01908205, United States Department of, Defense, Evdokia A. Intranasal Oxytocin for the Treatment of Children and Adolescents With Autism Spectrum Disorders (ASD). 2013.
    - E. Anagnostou, ed. Randomized Controlled Trial of Intranasal Oxytocin in Autism Spectrum Disorder; INSAR, 2020.
124. NCT01944046[261–264, 2614]
    - L. Sikich , A. Kolevzon , J. Veenstra-Vander Weele , C. McDougle and B. King, ed. Challenges in Evaluating Improvements in Soars-B: Study of Oxytocin in ASD for Enhancing Reciprocal Social Behaviors.
    - NCT01944046, Eunice Kennedy Shriver National Institute of Child, Health, Human D, Linmarie S. Study of Oxytocin in Autism to Improve Reciprocal Social Behaviors. 2013.
    - Sikich L, ed. A Large, Heterogenous OubleBlinded Trial of Oxytocin to Enhance Social Behaviours in ASD; INSAR, 2020.

- Spanos M, Chandrasekhar T, Kim SJ, et al. Rationale, design, and methods of the autism centers of excellence (ACE) network study of oxytocin in autism to improve reciprocal social behaviors (SOARS-B). Contemporary clinical trials. 2020:106103.
- Sikich, L., A. Kolevzon, B. H. King, C. J. McDougle, K. B. Sanders, S.-J. Kim, M. Spanos, T. Chandrasekhar, M. D. P. Trelles, C. M. Rockhill, M. L. Palumbo, A. Witters Cundiff, A. Montgomery, P. Siper, M. Minjarez, L. A. Nowinski, S. Marler, L. C. Shuffrey, C. Alderman, J. Weissman, B. Zappone, J. E. Mullett, H. Crosson, N. Hong, S. K. Siecinski, S. N. Giamberardino, S. Luo, L. She, M. Bhapkar, R. Dean, A. Scheer, J. L. Johnson, S. G. Gregory and J. Veenstra-VanderWeele (2021). "Intranasal Oxytocin in Children and Adolescents with Autism Spectrum Disorder." New England Journal of Medicine 385(16): 1462-1473.

125. NCT01966679[265]
    - NCT01966679, University of California, Los Angeles. Targeting GABA-A for the Treatment of Social Disability in Young Adults With Autism Spectrum Disorders: A Phase II Proof of Mechanism Trial. 2013.
126. NCT01972074[267]
    - NCT01972074, McLean H, Massachusetts General H. Behavioral and Neural Response to Memantine in Adolescents With Autism Spectrum Disorder. 2013.
127. NCT02222285[268]
    - NCT02222285, Enzymotec. An Exploratory, Double-Blind, Placebo-Controlled Study of the Medical Food Vayarin in Children With Autism Spectrum Disorder (ASD). 2014.
128. NCT02385799[269–271]
    - Alolaby RR, Jiraanont P, Durbin-Johnson B, et al. Molecular Biomarkers Predictive of Sertraline Treatment Response in Young Children With Autism Spectrum Disorder. Front Genet. 2020;11:308.
    - NCT02385799, Health R, Services A, R, i J. Hagerman, M. D. A Trial of Sertraline in Young Children With Autism Spectrum Disorder. 2015.
    - Potter LA, Scholze DA, Biag HMB, et al. A Randomized Controlled Trial of Sertraline in Young Children With Autism Spectrum Disorder. Frontiers in psychiatry. 2019;10:810. doi:10.3389/fpsyt.2019.00810.
129. NCT02410902[272]
    - NCT02410902, Curemark. A Trial of CM-AT in Children With Autism With All Levels of FCT (The Blum Study). 2015.
130. NCT02550912[274]
    - NCT02550912, Ain Shams U. A Study Evaluating the Effect of Vitamin D on Clinical Outcome in Autistic Children. 2015.
131. NCT02586935[275–277]
    - E. Anagnostou , R. Nicolson and T. Bennett, ed. A RCT of Tideglusib Vs Placebo: Data from the Pond Network; 2018.
    - E. Anagnostou , J. P. Horrigan and A. Yaroshinsky, ed. The Utility of Omnibus Statistical Approaches in Go/No-Go Decision Making in a Phase 2 Study in Adolescents with Autism Spectrum Disorder: A Case Example; 2019.
    - NCT02586935, Holl, Bloorview Kids Rehabilitation H, et al. Tideglusib vs. Placebo in the Treatment of Adolescents With Autism Spectrum Disorders. 2015.
132. NCT02871349[282, 283]
    - Beversdorf D, Ferguson B, Hunter S, et al., eds. Preliminary Report on Results from an Open- Label Extension Trial of the Effects of Propranolol on Core Symptoms and Anxiety in Autism Spectrum Disorder; 2020.

- NCT02871349, University of M-C. Trial of Propranolol in Children and Youth With Autism Spectrum Disorder and Predictors of Response. 2016.

133. NCT02879110[284, 2841]
- NCT02879110, Davis family f, University of C, University of Illinois at, Chicago, Central South U. A 12-weeks Study to Evaluate Sulforaphane in Treatment of Autism Spectrum Disorder. 2016.
- Smith, R.; Ou, J.; Jin, H.; Wu, R.; Fahey, J.; Arriaza, J. et al. (2020): Sulforaphane as a treatment for autism: a randomized double-blind study 45, pp. 79–80. Available online at https://www.cochranelibrary.com/central/doi/10.1002/central/CN-02244678/full

134. NCT02901431[285, 2851, 2852] (terminated, data are available in the website of the registry)
- NCT02901431, Hoffmann-La R. A Study to Investigate the Efficacy and Safety of RO5285119 in Participants With Autism Spectrum Disorder (ASD). 2016.
- Hollander, E.; Jacob, S.; Jou, R. J.; McNamara, N.; Sikich, L.; Tobe, R. et al. (2020): A PHASE 2 RANDOMIZED CONTROLLED TRIAL OF BALOVAPTAN IN PEDIATRIC PARTICIPANTS WITH AUTISM SPECTRUM DISORDER. In Journal of the American Academy of Child and Adolescent Psychiatry 59 (10), S262-S263. Available online at https://www.cochranelibrary.com/central/doi/10.1002/central/CN-02192238/full.
- Stark, F. S.; Chavanne, C.; Lennon-Chrimes, S.; Diack, C.; Derks, M.; Smith, J. (2020): P.133 Paediatric dosing of balovaptan for the treatment of the core symptoms of autism spectrum disorder: data from a Phase 2 study (aV1ation; NCT02901431) 40, S82-S83. Available online at https://www.cochranelibrary.com/central/doi/10.1002/central/CN-02212087/full

135. NCT02909959[286]
- NCT02909959, North Carolina T, Clinical Sciences I, University of North Carolina, Chapel Hill. Sulforaphane for the Treatment of Young Men With Autism Spectrum Disorder. 2016.

136. NCT02947048[287, 288, 2881, 2882]
- J. Rothman , P. Halas , E. J. Bartky , T. Fischer and J. T. Megerian 1, ed. An Open Label and Double Blind Randomized Placebo Controlled Pilot Study of L1-79 [D,L Alpha-Methyl-Para-Tyrosine (DL-AMPT)] for the Treatment of the Core Symptoms of Autism Spectrum Disorder (ASD) in Adolescent and Young Adult Males; 2019.
- NCT02947048, Yamo Pharmaceuticals, L. L. C., Halas FP. Safety of L1-79 in Autism. 2016.
- Fitzgerald, Michael (Ed.) (2021): Autism Spectrum Disorder - Profile, Heterogeneity, Neurobiology and Intervention: IntechOpen.
- Rothman, John (2021): L1-79 and the Role of Catecholamines in Autism. In Michael Fitzgerald (Ed.): Autism Spectrum Disorder - Profile, Heterogeneity, Neurobiology and Intervention: IntechOpen

137. NCT02956226[289, 290, 2901, 2902]
- Aran, M. Harel , L. Polyansky , A. Schnapp , N. Barnoy , N. Wattad , D. Shmueli , Y. Pollak and H. Cassuto, ed. A Placebo-Controlled Trial of Cannabinoids in Children with ASD; 2019.
- NCT02956226, Shaare Zedek Medical C. Cannabinoids for Behavioral Problems in Children With ASD. 2016.
- Aran A; Harel M; Cassuto H; Polyansky L; Schnapp A; Wattad N et al. (2021): Cannabinoid treatment for autism: a proof-of-concept randomized trial. In *Molecular Autism* 12 (1), p. 6. Available online at https://pubmed.ncbi.nlm.nih.gov/33536055/
- Castellanos, F. X. (2019): A placebo-controlled double-blind trial of cannabinoids in children and adolescents with autism spectrum disorder. In Neuropsychopharmacology 44,

pp. 61–62. Available online at https://www.cochranelibrary.com/central/doi/10.1002/central/CN-02147378/full.

138. NCT03156153[291, 292, 2921]
   - NCT03156153, Shanghai Jiao Tong University School of, Medicine, Xinhua Hospital, Shanghai Jiao Tong University School of Medicine. A Study of Bumetanide for the Treatment of Autism Spectrum Disorders. 2017.
   - ChiCTR-IPR-16009627, Xinhua Hospital Affiliated to Shanghai Jiaotong University School of, Medicine. A randomized double-blind placebo-controlled trial of the efficiency and mechanism of bumetanide on children with Autism Spectrum Disorder. 2016.
   - Dai, Yuan; Zhang, Lingli; Yu, Juehua; Zhou, Xin; He, Hua; Ji, Yiting et al. (2021): Improved symptoms following bumetanide treatment in children aged 3−6 years with autism spectrum disorder: a randomized, double-blind, placebo-controlled trial. In *Science Bulletin* 66 (15), pp. 1591–1598. DOI: 10.1016/j.scib.2021.01.008

139. NCT03337035[295, 296, 2961]
   - Kong X-J, Liu J, Li J, et al. Probiotics and oxytocin nasal spray as neuro-social-behavioral interventions for patients with autism spectrum disorders: a pilot randomized controlled trial protocol. Pilot Feasibility Stud. 2020;6:20.
   - Kong XJ; Liu J; Liu K; Koh M; Sherman H; Liu S et al. (2021): Probiotic and Oxytocin Combination Therapy in Patients with Autism Spectrum Disorder: A Randomized, Double-Blinded, Placebo-Controlled Pilot Trial. In *Nutrients* 13 (5). Available online at https://pubmed.ncbi.nlm.nih.gov/34062986/
   - NCT03337035, Massachusetts General H. Probiotics and Oxytocin Nasal Spray on Social Behaviors of Autism Spectrum Disorder (ASD) Children. 2017.

140. NCT03504917[300, 3001] (terminated, results are posted in the registration website)
   - NCT03504917, Hoffmann-La R. A Study of Balovaptan in Adults With Autism Spectrum Disorder With a 2-Year Open-Label Extension. 2018.
   - Jacob, S.; Veenstra-VanderWeele, J.; Murphy, D.; McCracken, J. T.; Smith, J.; Sanders, K. et al. (2020): 6.14 PHASE 3 RANDOMIZED CONTROLLED TRIAL OF BALOVAPTAN IN ADULTS WITH AUTISM SPECTRUM DISORDER. In Journal of the American Academy of Child and Adolescent Psychiatry 59 (10), S163-S164. Available online at https://www.cochranelibrary.com/central/doi/10.1002/central/CN-02192230/full.

141. NCT03550209[301]
   - NCT03550209, National Center for C, Integrative H, Sarah K. Fatty Acid Supplementation in Children With ASD. 2018.

142. Niederhofer 2002[303, 304]
   - Niederhofer H, Staffen W, Mair A. Galantamine may be effective in treating autistic disorder [5]. British Medical Journal. 14;325(7377):1422.
   - Niederhofer H, Staffen W, Mair A. Galantamine may be effective in treating autistic disorder. BMJ (clinical research ed.). 2002;325(7377):1422

143. Niederhofer 2002b[305, 306]
   - Niederhofer H, Staffen W, Mair A. Lofexidine in hyperactive and impulsive children with autistic disorder. Journal of the American Academy of Child and Adolescent Psychiatry. 2002;41(12):1396–1397.
   - Niederhofer H, Staffen W, Mair A. Lofexidine in hyperactive impulsive children with autistic disorder. Journal of the American Academy of Child and Adolescent Psychiatry. 2002;41(12):1396–1397.

144. Niederhofer 2003[307]

- Niederhofer H, Staffen W, Mair A. Tianeptine: a novel strategy of psychopharmacological treatment of children with autistic disorder. Human Psychopharmacology. 2003;18(5):389–393.

145. Niederhofer 2004[308]
   - Niederhofer H. Venlafaxine has modest effects in autistic children. Therapy. 2004;1(1):87–90.
146. Nikvarz 2017[309, 310]
   - IRCT201204037202N5, Tehran University of Medical, Sciences. Comparing efficacy and side effects of Memantine and Risperidone in treating autistic patients. 2012.
   - Nikvarz N, Alaghb, -Rad J, Tehrani-Doost M, Alimadadi A, Ghaeli P. Comparing efficacy and side effects of memantine vs. risperidone in the treatment of autistic disorder. Pharmacopsychiatry. 2017;50(1):19–25.
147. Noone 2014[311–313]
   - NCT01337700, Forest L, Montefiore Medical C. Milnacipran in Autism and the Functional Locus Coeruleus and Noradrenergic Model of Autism. 2010.
   - Noone R, Ferretti C, Taylor B, Racine E, Holl, er E. Milnacipran vs. Placebo in adult autism spectrum disorder: impact on hyperactivity/ impulsivity domain. Neuropsychopharmacology. 2014;39:S363-s364.
   - Noone RH, Ferretti CJ, Taylor BP, et al. Modulation of the locus coeruleus-noradrenergic system with milnacipran vs placebo in autism spectrum disorder. Biological Psychiatry. 2014;75(9):324s.
148. Owen 2008[314–316]
   - Lewis DW, Couch DM, Marcus RN, Manos G, Mankoski R, Carson WH. Efficacy and safety of flexibly-dosed aripiprazole for the treatment of irritability associated with autistic disorder in children and adolescents (6?17 years). Annals of Neurology. 2009;66:S110-111, Abstract no: 43.
   - NCT00332241, amp, Commercialization I, Otsuka America P, Otsuka Pharmaceutical D. Study of Aripiprazole in the Treatment of Children and Adolescents With Autistic Disorder (AD). 2006.
   - Owen R, Sikich L, Marcus RN, et al. Aripiprazole in the treatment of irritability in children and adolescents with autistic disorder. Pediatrics. 2009;124(6):1533–1540.
149. Parellada 2017[317–320]
   - EUCTR2007-006444-21-ES, Fundación para la Investigación Biomédica Hospital Gregorio, Marañón. EFFECT OF 8-WEEK FATTY ACIDS OMEGA-3 TREATMENT ON OXIDATIVE METABOLISM IN PATIENTS WITH AUTISM SPECTRUM DISORDER: A RANDOMISED DOUBLE-BLIND CROSSOVER PLACEBO-CONTROLLED TRIAL. - Omega-3 tr. 2010.
   - Moreno C, Calvo-Escalona R, Gutierrez S, et al. Effect of omega-3 polyunsaturated fatty acids on oxidative stress in children and adolescents with autism spectrum disorders. European neuropsychopharmacology. 2014;24:S725.
   - Parellada M, Llorente C, Calvo R, et al. Double-blind crossed-over randomized controlled-trial with omega-3 fatty acids for autism spectrum disorders. European neuropsychopharmacology. 2015;25:S138.
   - Parellada M, Llorente C, Calvo R, et al. Randomized trial of omega-3 for autism spectrum disorders: Effect on cell membrane composition and behavior. European neuropsychopharmacology. 2017;27(12):1319–1330.
150. Parker 2017[321, 322]

- NCT01624194, Stanford U. Intranasal Oxytocin Treatment for Social Deficits in Children With Autism. 2012.
- Parker KJ, Oztan O, Libove RA, et al. Intranasal oxytocin treatment for social deficits and biomarkers of response in children with autism. Proceedings of the national academy of sciences of the united states of america. 2017;114(30):8119–8124.

151. Pearson 2013[323–325]
    - NCT00178503, National Institute of Mental, Health, The University of Texas Health Science Center, Houston. Methylphenidate for Attention Deficit Hyperactivity Disorder and Autism in Children. 2005.
    - Pearson DA, Santos CW, Aman MG, et al. Effects of Extended-Release Methylphenidate Treatment on Cognitive Task Performance in Children with Autism Spectrum Disorder and Attention-Deficit/Hyperactivity Disorder. Journal of Child & Adolescent Psychopharmacology. 2020.
    - Pearson DA, Santos CW, Aman MG, et al. Effects of extended release methylphenidate treatment on ratings of attention-deficit/hyperactivity disorder (ADHD) and associated behavior in children with autism spectrum disorders and ADHD symptoms. Journal of Child & Adolescent Psychopharmacology. 2013;23(5):337–351.
152. Pusponegoro 2015[326]
    - Pusponegoro HD, Ismael S, Firmansyah A, Sastroasmoro S, V, enplas Y. Gluten and casein supplementation does not increase symptoms in children with autism spectrum disorder. Acta paediatrica. 2015;104(11):e500-5.
153. Quintana 1995[327, 328]
    - Quintana H, Birmaher B, Stedge D, et al. Use of methylphenidate in the treatment of children with autistic disorder. Journal of autism and developmental disorders. 1995;25(3):283–294.
    - Quintana H, Birmaher B, Stedge D, Lennon S, al. e. Use of methylphenidate in the treatment of children with autistic disorder. Annual Progress in Child Psychiatry & Child Development. 1996:295–307.
154. Ratliff 2005[329]
    - Ratliff-Schaub K, Carey T, Reeves GD, Rogers MA. Randomized controlled trial of transdermal secretin on behavior of children with autism. Autism. 2005;9(3):256–265.
155. Realmuto 1986[330]
    - Realmuto GM, Jensen J, Klykylo W, et al. Untoward effects of fenfluramine in autistic children. Journal of Clinical Psychopharmacology. 1986;6(6):350–355.
156. Reddihough 2019[331–334]
    - ACTRN12608000173392, None, Victorian Medical Insurance A. Fluoxetine for the treatment of repetitive behaviours in children and adolescents with autism: A randomised double-blind placebo-controlled trial. 2008.
    - Mouti A, Reddihough D, Marraffa C, et al. Fluoxetine for Autistic Behaviors (FAB trial): study protocol for a randomized controlled trial in children and adolescents with autism. Trials. 2014;15:230.
    - Reddihough D, Marraffa C, Mouti A, et al. A randomised placebo-controlled trial to determine if fluoxetine is effective for improving autistic behaviours. preprint-Lancet. 2019.
    - Reddihough DS, Marraffa C, Mouti A, et al. Effect of Fluoxetine on Obsessive-Compulsive Behaviors in Children and Adolescents With Autism Spectrum Disorders: A Randomized Clinical Trial. JAMA. 2019;322(16):1561–1569.
157. Remington 2001[335–338]

- King R, Fay G, Wheildon H. Re: Clomipramine vs. haloperidol in the treatment of autistic disorder: A double-blind, placebo, crossover study. Journal of Clinical Psychopharmacology. 2002;22(5):525–526.
- Remington G, Sloman L, Konstantareas M, Parker K, Gow R. Clomipramine versus haloperidol in the treatment of autistic disorder: a double-blind, placebo-controlled, crossover study. Journal of Clinical Psychopharmacology. 2001;21(4):440–444.
- Sloman L, Konstantareas M, Remington G. Re: Clomipramine vs. haloperidol in the treatment of autistic disorder: A double-blind, placebo, crossover study." Reply to Dr. King and associates. Journal of Clinical Psychopharmacology. 2002;22(5):526.
- Sloman L, Remington G, Konstantareas M, Parker K. Haloperidol versus clomipramine in autistic disorder. 151st annual meeting of the american psychiatric association; 1998 may 30 - jun 4; toronto. 1998(17).

158. EUCTR2015-000955-25-FR (NCT02551380; Renard 2020) [339–341]
    - EUCTR2015-000955-25-FR, Centre Hospitalier Régional Universitaire de, Nancy. Evaluation of the efficiency of B9 vitaminon the reduction of autistic spectrum symptoms:a pilot study "EFFET. 2015.
    - NCT02551380. Folinic Acid in Children With Autism Spectrum Disorders. https://ClinicalTrials.gov/show/NCT02551380. 2015.
    - Renard E, Leheup B, Guéant-Rodriguez R-M, Oussalah A, Quadros EV, Guéant J-L. Folinic acid improves the score of Autism in the EFFET placebo-controlled randomized trial. Biochimie. 2020. doi:10.1016/j.biochi.2020.04.019.
159. RISAUT-JPN (NCT01624675) [342, 343]
    - EUCTR2015-001320-31-Outside-EU/EEA, Janssen Pharmaceutical KK. A Study to Evaluate the Efficacy and Safety of Risperidone (R064766) in Children and Adolescents with Irritability Associated with Autistic Disorder. 2015.
    - NCT01624675, Janssen Pharmaceutical KK. A Study to Evaluate the Efficacy and Safety of Risperidone (R064766) in Children and Adolescents With Irritability Associated With Autistic Disorder. 2012.
160. RUPP 2002[344–363]
    - Adetunji B FMathews, Maju, Mathews M FOsinowo, Thomas, Osinowo T FWilliams, Adedapo, Williams A. Risperidone for the core symptom domains of autism.
    - Aman MG, Arnold LE, McDougle CJ, et al. Acute and long-term safety and tolerability of risperidone in children with autism. Journal of Child & Adolescent Psychopharmacology. 2005;15(6):869–884.
    - Aman MG, Hollway JA, McDougle CJ, et al. Cognitive effects of risperidone in children with autism and irritable behavior. Journal of Child & Adolescent Psychopharmacology. 2008;18(3):227–236.
    - Aman M, Rettiganti M, Nagaraja HN, et al. Tolerability, Safety, and Benefits of Risperidone in Children and Adolescents with Autism: 21-Month Follow-up After 8-Week Placebo-Controlled Trial. Journal of Child & Adolescent Psychopharmacology. 2015;25(6):482–493.
    - Anderson GM, Scahill L, McCracken JT, et al. Effects of short- and long-term risperidone treatment on prolactin levels in children with autism. Biological Psychiatry. 2007;61(4):545–550.
    - Arnold LE, Farmer C, Kraemer HC, et al. Moderators, mediators, and other predictors of risperidone response in children with autistic disorder and irritability. Journal of Child and Adolescent Psychopharmacology. 1;20(2):83–93.

- Arnold LE, Vitiello B, McDougle C, et al. Parent-defined target symptoms respond to risperidone in RUPP autism study: customer approach to clinical trials. Journal of the American Academy of Child and Adolescent Psychiatry. 2003;42(12):1443–1450.
- Carroll D, Hallett V, McDougle CJ, et al. Examination of aggression and self-injury in children with autism spectrum disorders and serious behavioral problems. Child and Adolescent Psychiatric Clinics of North America. 2014;23(1):57–72.
- Levine SZ, Kodesh A, Goldberg Y, et al. Initial severity and efficacy of risperidone in autism: results from the RUPP trial. European Psychiatry. 2016;32:16–20.
- Lindsay RL, Eugene Arnold L, Aman MG, et al. Dietary status and impact of risperidone on nutritional balance in children with autism: a pilot study. Journal of Intellectual and Developmental Disability. 2006;31(4):204–209.
- McDougle CJ, Scahill L, Aman MG, et al. Risperidone for the core symptom domains of autism: results from the study by the autism network of the research units on pediatric psychopharmacology. American journal of psychiatry. 2005;162(6):1142–1148.
- NCT00005014, National Institute of Mental, Health. Treatment of Autism in Children and Adolescents. 2000.
- Nurmi EL, Spilman SL, Whelan F, et al. Moderation of antipsychotic-induced weight gain by energy balance gene variants in the RUPP autism network risperidone studies. Translational psychiatry;3:e274.
- Research Units on Pediatric Psychopharmacology Autism Network. Risperidone in children with autism and serious behavioral problems. New England Journal of Medicine. 2002;347(5):314–321.
- Risperidone treatment of autistic disorder: longer-term benefits and blinded discontinuation after 6 months. American journal of psychiatry. 2005;162(7):1361–1369.
- Scahill L, Hallett V, Aman MG, et al. Brief Report: social disability in autism spectrum disorder: results from Research Units on Pediatric Psychopharmacology (RUPP) Autism Network trials. Journal of autism and developmental disorders. 2013;43(3):739–746.
- Scahill L, McCracken J, McDougle CJ, et al. Methodological issues in designing a multisite trial of risperidone in children and adolescents with autism. Journal of Child & Adolescent Psychopharmacology. 2001;11(4):377–388.
- Vitiello B, Aman MG, Scahill L, et al. Research knowledge among parents of children participating in a randomized clinical trial. Journal of the American Academy of Child and Adolescent Psychiatry;44(2):145–149.
- Vitiello B, Davies M, Arnold LE, et al. Assessment of the integrity of study blindness in a pediatric clinical trial of risperidone. Journal of Clinical Psychopharmacology. 2005;25(6):565–569.
- Vo LC, Snyder C, McCracken C, et al. No Apparent Cardiac Conduction Effects of Acute Treatment with Risperidone in Children with Autism Spectrum Disorder. Journal of Child and Adolescent Psychopharmacology. 2016;26(10):900–908.

161. RUPP 2005[364–370]
   - Jahromi LB, Kasari CL, McCracken JT, et al. Positive effects of methylphenidate on social communication and self-regulation in children with pervasive developmental disorders and hyperactivity. Journal of autism and developmental disorders. 2009;39(3):395–404.
   - McCracken JT, Badashova KK, Posey DJ, et al. Positive effects of methylphenidate on hyperactivity are moderated by monoaminergic gene variants in children with autism spectrum disorders. Pharmacogenomics journal. 2014;14(3):295–302.
   - NCT00025779, National Institute of Mental, Health. Methylphenidate in Children and Adolescents With Pervasive Developmental Disorders. 2001.

- Posey DJ, Aman MG, McCracken JT, et al. Positive effects of methylphenidate on inattention and hyperactivity in pervasive developmental disorders: an analysis of secondary measures. Biological Psychiatry. 2007;61(4):538–544.
- Research Units on Pediatric Psychopharmacology (RUPP) Autism Network. Randomized, controlled, crossover trial of methylphenidate in pervasive developmental disorders with hyperactivity. Archives of General Psychiatry. 2005;62(11):1266–1274.
- Scahill L, Aman MG, McDougle CJ, et al. A prospective open trial of guanfacine in children with pervasive developmental disorders. Journal of Child and Adolescent Psychopharmacology;16(5):589–598.
- Scahill L, Bearss K, Sarhangian R, et al. Using a Patient-Centered Outcome Measure to Test Methylphenidate Versus Placebo in Children with Autism Spectrum Disorder. Journal of Child and Adolescent Psychopharmacology. 2017;27(2):125–131.

162. *Saad 2015*[371] (excluded from the quantitative analysis due to concerns on data reliability; another trial of the same corresponding authors was retracted-Saad 2018)
   - Saad K, Eltayeb AA, Mohamad IL, et al. A Randomized, Placebo-controlled Trial of Digestive Enzymes in Children with Autism Spectrum Disorders. Clinical Psychopharmacology and Neuroscience. 2015;13(2):188–193.

163. *Saad 2018*[372–374] (excluded from the quantitative analysis due to concerns on data reliability; the trials was retracted for this reason)
   - Saad K. Response to letters: Randomized controlled trial of vitamin d supplementation in children with autism spectrum disorder - correction and additional information. Journal of Child Psychology and Psychiatry. 2018;59(1):e3-e5.
   - Saad K, Abdel-Rahman AA, Elserogy YM, et al. Randomized controlled trial of vitamin d supplementation in children with autism spectrum disorder. Journal of Child Psychology and Psychiatry. 2018;59(1):20–29.
   - JPRN-UMIN000020281 (2015): Randomized-Controlled Trial of Vitamin D Supplementation in Children with Autism Spectrum Disorder. Available online at https://www.cochranelibrary.com/central/doi/10.1002/central/CN-01837785/full.
   - Sonuga-Barke, E. (2018): Letter to the author from Editor-in-Chief seeking clarifications…Saad, K., Abhttp: //bkstrm2.epnet.com: 8080/ai/images/next.gifdel-Rahman, A.A., Elserogy, Y.M., Al-Atram, A.A., El-Houfey, A.A., Othman, H.A.K., … & Abdel-Salam, A.M. (2016). Randomized controlled trial of vitamin D supplementation in children with autism spectrum disor- der. Journal of Child Psychology and Psychiatry, 59, 20 – 29 59 (1), e2. Available online at https://www.cochranelibrary.com/central/doi/10.1002/central/CN-02113530/full.
   - Saad K, Abdel-Rahman A, Elserogy Y, et al. Retraction: Randomized controlled trial of vitamin D supplementation in children with autism spectrum disorder. Journal of Child Psychology and Psychiatry. 2019;60(6):711.

164. Santocchi 2016[375–377]
   - NCT02708901, Ministry of Health I, Istituto di Fisiologia Clinica, C. N. R., Maris IFS. Gut to Brain Interaction in Autism. Role of Probiotics on Clinical, Biochemical and Neurophysiological Parameters. 2016.
   - Santocchi E, Guiducci L, Prosperi M, et al., eds. Eect of Probiotic Supplementation on Behavioral and Gastrointestinal Symptoms in Autism Spectrum Disorders: A Randomized Double Blind, Placebo-Controlled Trial; 2019.
   - Santocchi E, Guiducci L, Fulceri F, et al. Gut to brain interaction in Autism Spectrum Disorders: a randomized controlled trial on the role of probiotics on clinical, biochemical and neurophysiological parameters. BMC Psychiatry. 2016;16:183.

- Santocchi, E., et al. "Effects of Probiotic Supplementation on Gastrointestinal, Sensory and Core Symptoms in Autism Spectrum Disorders: A Randomized Controlled Trial. Front Psychiatry. 2020; 11: 550593.

165. Scahill 2015[378–380]
    - NCT01238575, Emory U, Massachusetts General H, et al. Guanfacine for the Treatment of Hyperactivity in Pervasive Developmental Disorder. 2010.
    - Politte LC, Scahill L, Figueroa J, McCracken JT, King B, McDougle CJ. A randomized, placebo-controlled trial of extended-release guanfacine in children with autism spectrum disorder and ADHD symptoms: an analysis of secondary outcome measures. Neuropsychopharmacology;43(8):1772–1778.
    - Scahill L, McCracken JT, King BH, et al. Extended-Release Guanfacine for Hyperactivity in Children With Autism Spectrum Disorder. American journal of psychiatry. 2015;172(12):1197–1206.
166. Scifo 1991[381–383]
    - Marchetti B, Scifo R, Batticane N, Scapagnini U. Immunological significance of opioid peptide dysfunction in infantile autism. Brain Dysfunction. 1990;3(5):346–354.
    - Scifo R, Batticane N, Quattropani MC, Spoto G, Marchetti B. A double-blind trial with naltrexone in autism. Brain Dysfunction. 1991;4(6):301–307.
    - Scifo R, Cioni M, Nicolosi A, et al. Opioid-immune interactions in autism: behavioural and immunological assessment during a double-blind treatment with naltrexone. Annali dell'istituto superiore di sanita. 1996;32(3):351–359.
167. Shea 2004[384–388]
    - Kastner TA. Use of Risperidone in Developmentally Disabled Children. Pediatrics. 2005;115(5):1447. doi:10.1542/peds.2005-0156.
    - Light M, Dunbar F, Shea SE. Efficacy and safety of risperidone in the treatment of children with autistic and other pervasive developmental disorders (PDD): a randomized, double-blind, placebo controlled trial (P2.104). European neuropsychopharmacology. 2004;14:S278.
    - NCT00261508, Janssen-Ortho Inc C. A Study of the Effectiveness and Safety of Risperidone Versus Placebo in the Treatment of Children With Autistic Disorder and Other Pervasive Developmental Disorders (PDD). 2005.
    - Pandina GJ, Bossie CA, Youssef E, Zhu Y, Dunbar F. Risperidone improves behavioral symptoms in children with autism in a randomized, double-blind, placebo-controlled trial. Journal of autism and developmental disorders. 2007;37(2):367–373.
    - Shea S, Turgay A, Carroll A, et al. Risperidone in the treatment of disruptive behavioral symptoms in children with autistic and other pervasive developmental disorders. Pediatrics. 2004;114(5):e634-41.
168. Sherman 1989[389]
    - Sherman J, Factor DC, Swinson R, Darjes RW. The effects of fenfluramine (hydrochloride) on the behaviors of fifteen autistic children. Journal of autism and developmental disorders. 1989;19(4):533–543.
169. Sikich 2013 (NCT01308749) [390, 391]
    - NCT01308749, Autism S, University of North Carolina, Chapel Hill. A Study of Oxytocin in Children and Adolescents With Autistic Disorder. 2011.
    - Sikich L, Alderman C, Hazzard L, Bethea TC, Gregory S, Johnson J. Pilot study of sustained oxytocin treatment in children and adolescents with autistic disorder. Biological Psychiatry. 2013;73(9):145s.
170. Singh 2014[392, 393]

- NCT01474993, Johns Hopkins U, Andrew Z. Sulforaphane-rich Broccoli Sprout Extract for Autism. 2011.
- Singh, K.; Diggins, E.; Connors, S.; Zimmerman, A. (2018): Sulforaphane treatment of children with autism spectrum disorder (ASD)-A progress report 84, S309-S310. Available online at https://www.cochranelibrary.com/central/doi/10.1002/central/CN-01654140/full.
- Singh K, Connors SL, Macklin EA, et al. Sulforaphane treatment of autism spectrum disorder (ASD). Proceedings of the national academy of sciences of the united states of america. 2014;111(43):15550–15555.

171. NCT01993251 (SOMELIA 2015) [394, 395]
    - EUCTR2013-001230-17-FR, Hospices Civils de L. La mélatonine restaure-t-elle l'architecture du sommeil chez les enfants avec autisme ? Etude de phase II. 2015.
    - NCT01993251. Does Melatonin Restore Sleep Architecture in Autistic Children. https://ClinicalTrials.gov/show/NCT01993251. 2013
172. Stern 1990[396, 397]
    - Oades RD, Stern LM, Walker MK, Clark CR, Kapoor V. Event-related potentials and monoamines in autistic children on a clinical trial of fenfluramine. International journal of psychophysiology. 1990;8(3):197–212.
    - Stern LM, Walker MK, Sawyer MG, Oades RD, Badcock NR, Spence JG. A controlled crossover trial of fenfluramine in autism. Journal of child psychology and psychiatry, and allied disciplines. 1990;31(4):569–585.
173. Stivaros 2018[398]
    - Stivaros S, Garg S, Tziraki M, et al. Randomised controlled trial of simvastatin treatment for autism in young children with neurofibromatosis type 1 (SANTA). Molecular Autism. 22;9(190).
    - EUCTR2012-005742-38-GB (2013): Early phase triple blind placebo controlled RCT of simvastatin treatment for autism in young children with Neurofibromatosis Type 1. Available online at https://www.cochranelibrary.com/central/doi/10.1002/central/CN-01872255/full.
174. Sugie 2005[399–402]
    - Fukuda T, Sugie H, Ito M, Sugie Y. [Clinical evaluation of treatment with fluvoxamine, a selective serotonin reuptake inhibitor in children with autistic disorder]. No to Hattatsu [Brain & Development]. 2001;33(4):314–318.
    - Fukuda T, Sugie H, Ito M, Sugie Y. Clinical evaluation of treatment with fluvoxamine, a selective serotonin reuptake inhibitor, in children with autistic disorder. [Japanese]. No to Hattatsu [Brain & Development]. 2001;33(4):314–318.
    - Sugie Y, Sugie H, Fukuda T, Ito M, Ohzeki T. Serotonin 2A receptor gene polymorphism and clinical efficacy of fluvoxamine in children with autistic disorder. No to hattatsu = brain and development. 2003;35(1):23–28.
    - Sugie Y, Sugie H, Fukuda T, et al. Clinical efficacy of fluvoxamine and functional polymorphism in a serotonin transporter gene on childhood autism. Journal of autism and developmental disorders. 2005;35(3):377–385.
175. Sugiyama 1998[403, 404]
    - Sugiyama N, Sugie H, Igarashi Y, Ito M, Fukuda T. Low dose levodopa therapy of autistic disorder: Evaluation of clinical effectiveness. [Japanese]. No to Hattatsu [Brain & Development]. 1998;30(1):51–55.
    - Sugiyama N, Sugie H, Igarashi Y, Ito M, Fukuda T. Low-dose levodopa therapy of autistic disorder: evaluation of clinical effectiveness. No to hattatsu = brain and development. 1998;30(1):51–55.

176. Tordjman 2013[405–407]
- EUCTR2008-001689-97-FR, Rennes, C. H. U. de. Etude de la relation dose-effet de la mélatonine dans l'autisme infantile. - MELADOSE. 2009.
- NCT01780883, Centre Hospitalier Guillaume Régnier, Rennes, Rennes University H. Melatonin Dose-effect Relation in Childhood Autism. 2013.
- Tordjman S, Kermarrec S, Cohen D, et al. MELADOSE: Study of the dose-response relationship for melatonin in childhood autism. [French] Meladose: Etude de la relation dose-effet de la melatonine dans l'autisme infantile. Neuropsychiatrie de l'Enfance et de l'Adolescence. 2013;61(7):415–416.

177. UMIN000009075[409] (terminated with target at 40 participants; last modified on 11.10.2014)
- JPRN-UMIN000009075, Osaka University H. Effects of long-term administration of intranasal oxytocin on autism spectrum disorders. 2012.

178. Vasconcelos 2014[411–413]
- Brito AR, Vairo, Giselle de Paula Teixeira, Dias, Ana Paula Botelho Henriques, Olej B, Nascimento OJM, Vasconcelos MM. Effect of prednisolone on language function in children with autistic spectrum disorder: a randomized clinical trial. J Pediatr (Rio J). 2020.
- RBR-7nq8m7, Brazil H. Steroid Therapy for Autistic Children. 2011.
- Vasconcelos MM, Brito AR, Vairo GT, et al. c(corticosteroids for autism - A scientific trial) study. Annals of Neurology. 2014;76:S189-s190.

179. Veenstra-Vanderweele 2017[414–417]
- Kaufmann WE, Walton-Bowen KL, Kuriyama N, et al. Randomized, controlled, phase2 trial of STX209 (arbaclofen) for social function in autism spectrum disorder. Annals of Neurology. 2013;74:S129-s130.
- NCT01288716, Seaside Therapeutics I. Study of Arbaclofen for the Treatment of Social Withdrawal in Subjects With Autism Spectrum Disorders. 2011.
- NCT01706523, Seaside Therapeutics I. Open Label Extension Study of STX209 (Arbaclofen) in Autism Spectrum Disorders. 2012.
- Veenstra-V, erweele J, Cook EH, et al. Arbaclofen in Children and Adolescents with Autism Spectrum Disorder: a Randomized, Controlled, Phase 2 Trial. Neuropsychopharmacology. 2017;42(7):1390–1398.

180. Voigt 2014[418, 419]
- NCT00577447, Dsm Nutritional Products I, Mayo C. Docosahexaenoic Acid in the Treatment of Autism. 2007.
- Voigt RG, Mellon MW, Katusic SK, et al. Dietary docosahexaenoic acid supplementation in children with autism. Journal of pediatric gastroenterology and nutrition. 2014;58(6):715–722.

181. Wang 2020[420, 421]
- ChiCTR2000028985. Clinical study for intervention effect of the probiotics in autistic children. 2020.
- Wang Y, Li N, Yang J-J, et al. Probiotics and fructo-oligosaccharide intervention modulate the microbiota-gut brain axis to improve autism spectrum reducing also the hyper-serotonergic state and the dopamine metabolism disorder. Pharmacol Res. 2020:104784. doi:10.1016/j.phrs.2020.104784.

182. Wasserman 2006[422]
- Wasserman S, Iyengar R, Chaplin WF, et al. Levetiracetam versus placebo in childhood and adolescent autism: a double-blind placebo-controlled study. International Clinical Psychopharmacology. 2006;21(6):363–367.

183. Watanabe 2015[423–426]

- Benner S, Aoki Y, Watanabe T, et al. Neurochemical evidence for differential effects of acute and repeated oxytocin administration. Mol Psychiatry. 2018.
- JPRN-UMIN000007122, Showa University School of, Medicine, Department of Neuropsychiatry, Graduate School of Medicine University of Tokyo. A randomized, double-blind and cross-over trial to examine effects of continuous administration of intranasal oxytocin on social dysfunction in subjects with autism spectrum disorders. 2012.
- Owada K, Watanabe T, Kuroda M, et al. Development of quantitative facial expressions as a surrogate marker for autism spectrum disorder and oxytocin's effect on it. Biological Psychiatry;1:124S-125S.
- Watanabe T, Kuroda M, Kuwabara H, et al. Clinical and neural effects of six-week administration of oxytocin on core symptoms of autism. Brain. 2015;138:3400–3412.

184. Willemsen-Swinkels 1996[427, 428]
- Willemsen-Swinkels SH, Buitelaar JK, Weijnen FG, Engel, H. Placebo-controlled acute dosage naltrexone study in young autistic children. Psychiatry research. 1995;58(3):203–215.
- Willemsen-Swinkels SH, Buitelaar JK, Engel, H. The effects of chronic naltrexone treatment in young autistic children: a double-blind placebo-controlled crossover study. Biological Psychiatry. 1996;39(12):1023–1031.

185. Wink 2016[429, 430]
- NCT00453180, National Alliance for Autism, Research, Indiana University School of, Medicine. A Study of Oral N-Acetylcysteine in Children With Autism Spectrum Disorders. 2007.
- Wink LK, Adams R, Wang Z, et al. A randomized placebo-controlled pilot study of N-acetylcysteine in youth with autism spectrum disorder. Molecular Autism. 2016;7:26.

186. Wink 2020 [431, 432]
- NCT02611921, Roivant Sciences I, Cures Within R, Children's Hospital Medical Center, Cincinnati. Study of Intranasal Ketamine for Social Impairment in Autism Spectrum Disorder. 2015.
- Wink, L. K. (2019): Results of a Double-Blind, Placebo-Controlled Crossover Study of Intranasal Ketamine in Adolescents and Young Adults With Autism Spectrum Disorder 58 (10), S298-. Available online at https://www.cochranelibrary.com/central/doi/10.1002/central/CN-01999528/full.
- Wink, L. K.; Horn, P.; Reisinger, D.; Shaffer, R.; O'Brien, K.; Pedapati, E. V. et al. (2019): 5.14 RESULTS OF A DOUBLE-BLIND, PLACEBO-CONTROLLED CROSSOVER STUDY OF INTRANASAL KETAMINE IN ADOLESCENTS AND YOUNG ADULTS WITH ASD 58 (10), S249-. Available online at https://www.cochranelibrary.com/central/doi/10.1002/central/CN-01999527/full.
- Wink LK, Reisinger DL, Horn P, et al. Brief Report: Intranasal Ketamine in Adolescents and Young Adults with Autism Spectrum Disorder-Initial Results of a Randomized, Controlled, Crossover, Pilot Study. Journal of autism and developmental disorders. 2020.

187. Wright 2011[433, 434]
- ISRCTN77884120, York Primary Care T, North Y. Melatonin treatment for sleep problems in children with autism: a randomised controlled crossover trial. 2008.
- Wright B, Sims D, Smart S, et al. Melatonin versus placebo in children with autism spectrum conditions and severe sleep problems not amenable to behaviour management strategies: a randomised controlled crossover trial. Journal of autism and developmental disorders. 2011;41(2):175–184.

188. Yamasue 2018[435, 436, 4361]

- JPRN-UMIN000015264, Kanazawa University Hospital, Nagoya University Hospital University of Fukui Hospital, Department of Neuropsychiatry, The University of Tokyo Hospital. A multicenter, parallel group, placebo-controlled, double blind, confirmatory trial of intranasal oxytocin in participants with autism spectrum disorders. 2014.
- Kato Y; Kuwabara H; Okada T; Munesue T; Benner S; Kuroda M et al. (2021): Oxytocin-induced increase in N,N-dimethylglycine and time course of changes in oxytocin efficacy for autism social core symptoms. In *Molecular Autism* 12 (1), p. 15. Available online at https://pubmed.ncbi.nlm.nih.gov/33622389/
- Yamasue H, Okada T, Munesue T, et al. Effect of intranasal oxytocin on the core social symptoms of autism spectrum disorder: a randomized clinical trial. Molecular Psychiatry. 2018;29:1–10.

189. Yarbrough 1987[439]
- Yarbrough E, Santat U, Perel I, Webster C, Lombardi R. Effects of fenfluramine on autistic individuals residing in a state developmental center. Journal of autism and developmental disorders. 1987;17(3):303–314.

190. Yatawara 2017[440, 441]
- ACTRN12611000061932, University University of S. Effects of Oxytocin on Social Behavior and Repetitive Behavior in Children with Autism. 2011.
- Yatawara CJ, Einfeld SL, Hickie IB, Davenport TA, Guastella AJ. The effect of oxytocin nasal spray on social interaction deficits observed in young children with autism: a randomized clinical crossover trial. Molecular Psychiatry. 2016;21(9):1225–1231.

191. Yui 2013[442–447]
- JPRN-JMA-IIA00041, Kunio Y. Therapeutic Effect of Dietary Omega-6 and Omega-3 Fatty Acids in Improving Social Impairment in Youth With Autism Spectrum Disorders: A Double-Blind, Randomized, Placebo-Controlled Trial. 2010.
- NCT01154894, Ashiya U. Dietary Fatty Acids Improve Social Impairment in Autism Spectrum Disorders. 2010.
- Yui K, Koshiba M, Nakamura S, Onishi M. [Therapeutic effects of larger doses of arachidonic acid added to DHA on social impairment and its relation to alterations of polyunsaturated fatty acids in individuals with autism spectrum disorders]. Nihon Shinkei Seishin Yakurigaku Zasshi. 2011;31(3):117–124.
- Yui K, Koshiba M, Nakamura S, Kobayashi Y. Effects of large doses of arachidonic acid added to docosahexaenoic acid on social impairment in individuals with autism spectrum disorders: a double-blind, placebo-controlled, randomized trial. Journal of Clinical Psychopharmacology. 2012;32(2):200–206.
- Yui K, Koshiba K, Nakamura S. Effects of adding large doses of arachidonic acid to docosahexaenoic acid on social impairment in individuals with autism spectrum disorders. Current psychopharmacology. 2013;2(1):84–90.
- Yui K, Murphy D, Hamakawa H. [Effects of arachidonic acids on social behavior in patients with autism spectrum disorders]. Seishin Shinkeigaku Zasshi - Psychiatria et Neurologia Japonica. 2009;111(11):1387–1396.

192. Zimmerman 2021 (NCT02561481) [448, 449, 4491, 4492]

- Zimmerman A, Diggins E, Connors S, Singh K. Sulforaphane treatment of children with autism spectrum disorder (ASD) - A progress report. Neurology. Conference: 70th annual meeting of the american academy of neurology, AAN 2018. United states. 2018;90(15).

- NCT02561481, Congressionally Directed Medical Research, Programs, Johns Hopkins U, University of Massachusetts W. Sulforaphane Treatment of Children With Autism Spectrum Disorder (ASD). 2015
- Correction to: randomized controlled trial of sulforaphane and metabolite discovery in children with Autism Spectrum Disorder (Molecular Autism, (2021), 12, 1, (38), 10.1186/s13229-021-00447-5) (2021) 12 (1). Available online at https://www.cochranelibrary.com/central/doi/10.1002/central/CN-02299375/full.
- Zimmerman AW; Singh K; Connors SL; Liu H; Panjwani AA; Lee LC et al. (2021): Randomized controlled trial of sulforaphane and metabolite discovery in children with Autism Spectrum Disorder. In *Molecular Autism* 12 (1), p. 38. Available online at https://pubmed.ncbi.nlm.nih.gov/34034808/

193. IRCT20131013014994N5[154]
    - IRCT20131013014994N5, Kermanshah University of Medical, Sciences. effect of vitamin D on Autism Spectrum Disorders. 2018.
    - Javadfar Z, Abdollahzad H, Moludi J, Rezaeian S, Amirian H, Akbar Foroughi A, Mostafa Nachvak S, Goharmehr N, and Mostafai R. Effects of vitamin D supplementation on core symptoms, serum serotonin and interleukin 6 in children with autism spectrum disorders: A Randomized Clinical Trial. Nutrition. 2020: 110986.
194. NCT00965068[1231, 1232]
    - NCT00965068, Human D, Eunice Kennedy Shriver National Institute of Child, Health. Cholesterol in ASD: Characterization and Treatment. 2009.
    - Ging-Jehli, Nadja Rita; Manda, Deepa; Hollway, Jill; Hurt, Elizabeth; Moone, Stacey; Arnold, L. Eugene (2021): A Placebo-Controlled Pilot Exploration of Cholesterol Supplementation for Autistic Symptoms in Children with Low Cholesterol. In *J Dev Phys Disabil* 33 (5), pp. 819–837. DOI: 10.1007/s10882-020-09776-4.
195. NCT01813318[452] (completed, last updated 03.02.2021)
    - NCT01813318, Autism S, Children's Hospital Medical Center, Cincinnati. Study of Acamprosate in Autism. 2013.
196. NCT03487770[299] (completed; last update posted on 03.01.2021)
    - NCT03487770, Otsuka Beijing Research I. Aripiprazole Oral Solution in the Treatment of Children and Adolescents With Autistic Disorder. 2018.
197. NCT03369431[297] (completed; last update posted on 29.04.2021)
    - NCT03369431, University College L. Efficacy of Vivomixx on Behaviour and Gut Function in Autism Spectrum Disorder. 2017.
198. NCT02487082[273] (terminated at 12 participants; last update posted on 28.08.2021)
    - NCT02487082, Korea Institute of S, Technology, Stony Brook U. Pilot Study of Sleep Therapy and Biomarkers in Children With Autism Spectrum Disorders. 2015.
199. NCT03715166[76, 7671, 7672] (terminated due to negative interim results based on 211 participants; last update posted on 09.07.2021 in the website of the sponsor https://servier.com/en/communique/servier-and-neurochlore-announce-the-main-results-of-the-two-phase-3-clinical-studies-assessing-bumetanide-in-the-treatment-of-autism-spectrum-disorders-in-children-and-adolescents/#_ftnref1)
    - EUCTR2017-004419-38-NL, Institut de Recherches Internationales, Servier. Efficacy and safety of bumetanide oral liquid formulation in children and adolescents aged from 7 to less than 18 years old with Autism Spectrum Disorder. 2018.
    - EUCTR2017-004419-38-DE (2018): Efficacy and safety of bumetanide oral liquid formulation in children and adolescents aged from 7 to less than 18 years old with Autism

Spectrum Disorder. Available online at https://www.cochranelibrary.com/central/doi/10.1002/central/CN-01904740/full.
    - NCT03715166 (2018): Efficacy and Safety of Bumetanide Oral Liquid Formulation in Children and Adolescents Aged From 7 to Less Than 18 Years Old With Autism Spectrum Disorder. Available online at https://www.cochranelibrary.com/central/doi/10.1002/central/CN-01648185/full.

200. NCT03715153[7771, 7772, 7773] (terminated due to negative interim results based on 211 participants; last update posted on 09.07.2021 in the website of the sponsor https://servier.com/en/communique/servier-and-neurochlore-announce-the-main-results-of-the-two-phase-3-clinical-studies-assessing-bumetanide-in-the-treatment-of-autism-spectrum-disorders-in-children-and-adolescents/#_ftnref1)
    - EUCTR2017-004420-30-NL, Institut de Recherches Internationales, Servier. Efficacy and safety of bumetanide oral liquid formulation in children aged from 2 to less than 7 years old with Autism Spectrum Disorder. 2018.
    - EUCTR2017-004420-30-PT (2018): Efficacy and safety of bumetanide oral liquid formulation in children aged from 2 to less than 7 years old with Autism Spectrum Disorder. Available online at https://www.cochranelibrary.com/central/doi/10.1002/central/CN-01909773/full.
    - NCT03715153 (2018): Efficacy and Safety of Bumetanide Oral Liquid Formulation in Children Aged From 2 to Less Than 7 Years Old With Autism Spectrum Disorder. Available online at https://www.cochranelibrary.com/central/doi/10.1002/central/CN-01648229/full.

201. Doaei 2021[1241, 1242]
    - IRCT2015122625699N1, Food Technology Research I, National N. The effect of Omega-3 on Autism. 2016.
    - Doaei S; Bourbour F; Teymoori Z; Jafari F; Kalantari N; Abbas Torki S et al. (2021): The effect of omega-3 fatty acids supplementation on social and behavioral disorders of children with autism: a randomized clinical trial. In *Pediatric endocrinology, diabetes, and metabolism* 27 (1), pp. 12–18. Available online at https://pubmed.ncbi.nlm.nih.gov/33599431/

202. Hayashi 2021[1]
    - Hayashi M; Mishima K; Fukumizu M; Takahashi H; Ishikawa Y; Hamada I et al. (2021): Melatonin Treatment and Adequate Sleep Hygiene Interventions in Children with Autism Spectrum Disorder: A Randomized Controlled Trial. In *Journal of autism and developmental disorders*. Available online at https://pubmed.ncbi.nlm.nih.gov/34181143/.

203. NCT00467818[1]
    - NCT00467818 Dentistry of New J, National Center for C, Integrative H, University of M. Omega 3 Fatty Acids in the Treatment of Children With Autism Spectrum Disorders. 2007.

4.3.2 Ongoing trials (as of 06.10.2021)

1. NCT03279471[450] (recruiting; last update posted 13.01.2021)
    - NCT03279471, University of California D. Specifying and Treating Anxiety in Autism Research. 2017.
2. NCT03514784[451] (recruiting; last update posted 17.11.2020)
    - NCT03514784, The University of Texas Health Science Center, Houston. Combination Probiotic: BB-12 With LGG (Different Doses) in Treating Children With Autism Spectrum Disorder. 2018.

3. NCT02839915[281] (recruiting; last update posted on 24.08.2021)
   - NCT02839915, Phoenix Children's H, Harvard U, Emory U. Folinic Acid and Language Impairment in Autism Spectrum Disorder. 2016.
4. Yamasue 2018b[437, 438] (active; last updated posted on 20.12.2019)
   - JPRN-UMIN000031412, Hamamatsu University School of Medicine, Department of Psychiatry. An early phase II trial for efficacy and safety of TTA-121 on autism spectrum disorder. 2018.
   - NCT03466671, Japan Agency for Medical, Research, Development, Hamamatsu U. A Trial of TTA-121 on Autism Spectrum Disorder. 2018

5. NCT03553875[302] (recruiting; last update posted on 09.07.2021)
   - NCT03553875, Massachusetts General H. Memantine for the Treatment of Social Deficits in Youth With Disorders of Impaired Social Interactions. 2018.

6. UMIN000002650[408] (pre-initiation status; last modified on 24.10.2013)
   - JPRN-UMIN000002650, Department of Pediatrics Tohoku University School of Medicine National Institute of Mental Health: National Center of, Neurology, Psychiatry Yasuhara Children C, Department of Disaster Public, Health. Effects of vitamin B6 in children with autism: a randomized controlled trial. 2009.

7. UMIN000017876[410] (enrolling by invitation; last modified on 10.11.2015)
   - JPRN-UMIN000017876, United Graduate School of Child Development, Osaka University. Effects of long-term administration of intranasal oxytocin in children with autism spectrum disorder. 2015.

8. NCT03434366[298] (recruiting; last update posted on 26.03.2020)
   - NCT03434366, Children's Medical C, Guangzhou W. Intranasal Ketamine With Dexmedetomidine for the Treatment of Children With Autism Spectrum Disorder. 2018.

9. NCT03204786[294] (recruiting; last update posted on 05.10.2021)
   - NCT03204786, Eunice Kennedy Shriver National Institute of Child, Health, Human D, Stanford U. Intranasal Vasopressin Treatment in Children With Autism. 2017.

10. NCT03202303[293] (recruiting; last update posted on 05.08.2021)
    - NCT03202303, United States Department of, Defense, Eric H, er. Cannabidivarin (CBDV) vs. Placebo in Children With Autism Spectrum Disorder (ASD). 2017.

11. NCT02677051[279] (recruiting; last update posted on 14.07.2021)
    - NCT02677051, Rowan U, Rutgers, The State University of New Jersey. Sulforaphane in a New Jersey (NJ) Population of Individuals With Autism. 2016.

12. NCT02627508[278] (recruiting; last update posted on 03.05.2021)
    - NCT02627508, Simons F, Stanford U. Pilot Trial of Pregnenolone in Autism. 2015.

13. NCT01970345[266] (recruiting; last update posted on 15.07.2021)
    - NCT01970345, Autism Science F, Icahn School of Medicine at Mount, Sinai. A Pilot Treatment Study of Insulin-Like Growth Factor-1 (IGF-1) in Autism Spectrum Disorder. 2013.
14. ACTRN12613000334707[1] (ongoing; last updated on 26.03.2013)
    - ACTRN12613000334707, Telethon Institute for Child Health Research, University of Western Australia, Hospital Princess Margaret H. A randomized controlled trial of fish-oil supplementation for children with autism spectrum disorder. 2013.

15. ACTRN12617000441314[2] (ongoing; last updated on 22.02.2019)
    - ACTRN12617000441314, University of Western A, University of S. A Course of Oxytocin to Improve Social Communication in Young Children with Autism. 2017.
16. ACTRN1219000615189 (ongoing; last update posted on 17.02.2021)
    - ACTRN12619000615189 (2019): Prebiotic supplement use, the gut microbiome and behaviour change in children with autism spectrum disorder (ASD). Available online at https://www.cochranelibrary.com/central/doi/10.1002/central/CN-01974264/full.
17. UMIN000035172 (ongoing; last update posted on 10.06.2021)
    - JPRN-JMA-IIA00438 (2020): The efficacy and safety of pyridoxamine in patients with autism spectrum disorder; Exploratory physician-led Phase 2 trial. Available online at https://www.cochranelibrary.com/central/doi/10.1002/central/CN-02172398/full.
    - JPRN-jRCT2021200001 (2020): The efficacy and safety of pyridoxamine in patients with autism spectrum disorder; Exploratory physician-led Phase 2 trial. Available online at https://www.cochranelibrary.com/central/doi/10.1002/central/CN-02172414/full.
    - JPRN-UMIN000035172 (2018): The efficacy and safety of pyridoxamine in patients with autism spectrum disorder; Exploratory physician-led Phase II trial. Available online at https://www.cochranelibrary.com/central/doi/10.1002/central/CN-01946502/full.
18. UMIN000035175 (ongoing; last update posted on 17.12.2018)
    - JPRN-jRCTs031180411 (2019): Rare sugar in the patient with autism. Available online at https://www.cochranelibrary.com/central/doi/10.1002/central/CN-01971045/full.
    - JPRN-UMIN000035175 (2018): Safety and efficacy of rare sugar in children with autism spectrum disorder. Available online at https://www.cochranelibrary.com/central/doi/10.1002/central/CN-01946503/full.
19. JPRCTS051190017 (ongoing; last update posted on 17.05.2021)
    - JPRN-jRCTs051190017 (2019): Double blinded randomized placebo controlled trial to examine if intake of 5-aminolevulinic acid supplement can improve clinical symptoms of individuals with Autism Spectrum Disorder. Available online at https://www.cochranelibrary.com/central/doi/10.1002/central/CN-01972240/full.
20. UMIN000033113 (ongoing; last update posted on 01.08.2019)
    - JPRN-UMIN000033113 (2018): RCT of Autism spectrum disorder and probiotics. Available online at https://www.cochranelibrary.com/central/doi/10.1002/central/CN-01910855/full.
21. NCT03682978 (ongoing; last update posted on 28.08.2021)
    - NCT03682978 (2018): Arbaclofen in Children and Adolescents With ASD. Available online at https://www.cochranelibrary.com/central/doi/10.1002/central/CN-01663564/full.
    - Parellada M; San José Cáceres A; Palmer M; Delorme R; Jones EJH; Parr JR et al. (2021): A Phase II Randomized, Double-Blind, Placebo-Controlled Study of the Efficacy, Safety, and Tolerability of Arbaclofen Administered for the Treatment of Social Function in Children and Adolescents With Autism Spectrum Disorders: Study Protocol for AIMS-2-TRIALS-CT1. In *Frontiers in psychiatry* 12, p. 701729. Available online at https://pubmed.ncbi.nlm.nih.gov/34504446/
22. NCT03757585 (ongoing; last update posted on 24.02.2021)
    - NCT03757585 (2018): Management of Emotional Dysregulation in Youth With Non-verbal Learning Disability (NVLD) and/or Autism Spectrum Disorders (ASD) Using Telepsychiatry of Complementary and Alternative Treatments. Available online at https://www.cochranelibrary.com/central/doi/10.1002/central/CN-01918501/full.
23. NCT03887676 (ongoing; last update posted on 13.08.2021)

- NCT03887676 (2019): Arbaclofen vs. Placebo in the Treatment of Children and Adolescents With ASD (ARBA). Available online at https://www.cochranelibrary.com/central/doi/10.1002/central/CN-01919372/full.

24. NCT04031755 (ongoing; last update posted on 10.03.2021)
    - NCT04060017 (2019): Early Treatment of Language Impairment in Young Children With Autism Spectrum Disorder With Leucovorin Calcium. Available online at https://www.cochranelibrary.com/central/doi/10.1002/central/CN-01983530/full.
25. NCT04174365 (ongoing; last update posted on 28.05.202)

NCT04174365 (2019): Brexpiprazole in Treatment of Children and Adolescents With Irritability Associated With Autism Spectrum Disorder. Available online at https://www.cochranelibrary.com/central/doi/10.1002/central/CN-02004225/full

26. NCT04060030 (ongoing; last update posted on 10.03.2021)
    - NCT04060030 (2019): Treatment of Social and Language Deficits With Leucovorin for Young Children With Autism. Available online at https://www.cochranelibrary.com/central/doi/10.1002/central/CN-01983531/full.
27. NCT04293783 (ongoing; last update posted on 03.03.2020)
    - NCT04293783 (2020): Randomized Double-blind Clinical Trial With L.Reuteri Supplementation in Children With Autism Spectrum Disorder. Available online at https://www.cochranelibrary.com/central/doi/10.1002/central/CN-02088723/full.
28. NCT04312152 (ongoing; last update posted on 18.03.2020)
    - NCT04312152 (2020): Q10 Ubiquinol in Autism Spectrum Disorder and in Phelan-McDermid Syndrome. Available online at https://www.cochranelibrary.com/central/doi/10.1002/central/CN-02089167/full.
29. NCT04312932 (ongoing; last update posted on 09.09.2021)
    - NCT04312932 (2020): Fatty Acid Supplementation in Children With ASD (Study 2). Available online at https://www.cochranelibrary.com/central/doi/10.1002/central/CN-02089174/full.
30. NCT04517799 (ongoing; last update posted on 05.09.2021)
    - NCT04517799 (2020): Trial of Cannabidiol to Treat Severe Behavior Problems in Children With Autism. Available online at https://www.cochranelibrary.com/central/doi/10.1002/central/CN-02162910/full.
31. NCT04520685 (ongoing; last update posted on 23.07.2021)
    - NCT04520685 (2020): CAnnabidiol Study in Children With Autism Spectrum DisordEr. Available online at https://www.cochranelibrary.com/central/doi/10.1002/central/CN-02162969/full.
32. NCT04623398 (ongoing; last update posted on 16.09.2021)
    - NCT04623398 (2020): Effect of Lithium in Patients With Autism Spectrum Disorder and Phelan-McDermid Syndrome (SHANK3 Haploinsufficiency). Available online at https://www.cochranelibrary.com/central/doi/10.1002/central/CN-02197416/full.
33. TCTR20200522003 (ongoing; last update posted on)
    - TCTR20200522003 (2020): Folate receptor alpha autoantibody in children with autism spectrum disorder: establishment of in-house ELISA and efficacy of folinic acid â?? a randomized controlled trial. Available online at https://www.cochranelibrary.com/central/doi/10.1002/central/CN-02189820/full.
34. ACTRN12621000801819 (ongoing; version 2 of the protocol posted on 24.06.2021)
    - ACTRN12621000801819 (2021): SerTRaline for AnxieTy in adults with a diagnosis of Autism (STRATA) A randomised controlled trial. Available online at https://www.cochranelibrary.com/central/doi/10.1002/central/CN-02280170/full.

- ISRCTN15984604 (2021): Sertraline for anxiety in adults with a diagnosis of autism. Available online at https://www.cochranelibrary.com/central/doi/10.1002/central/CN-02238056/full.

35. ACTRN12621000029897 (ongoing; last update posted on 12.04.2021)
    - ACTRN12621000029897 (2021): Effect of Probiotic supplementation on gut bacteria in children with autism - a pilot randomized controlled trial. Available online at https://www.cochranelibrary.com/central/doi/10.1002/central/CN-02241654/full.
36. ChiCTR2000037941 (last update posted on 30.03.2021)
    - ChiCTR2000037941 (2020): The role of probiotics in children with autism spectrum disorders: a randomized controlled trial. Available online at https://www.cochranelibrary.com/central/doi/10.1002/central/CN-02241091/full.
37. ChiCTR2000040336 (last update posted on 16.02.2021)
    - ChiCTR2000040336 (2020): Lithium treatment for children with autism. Available online at https://www.cochranelibrary.com/central/doi/10.1002/central/CN-02239734/full.
38. ChiCTR2100042512 (ongoing; last update posted on 03.05.2021)
    - ChiCTR2100042512 (2021): A randomized, double-blind, controlled study of nutrition intervention in children with autism with bifidobacterium lactate M8+ low carbon balanced diet. Available online at https://www.cochranelibrary.com/central/doi/10.1002/central/CN-02280595/full.
39. CTRI/2020/12/030101 (ongoing; last update posted on 28.12.2020)
    - CTRI/2020/12/030101 (2020): Studying vitamin D status and the efficacy of vitamin D supplementation in autistic children in Bangladesh. Available online at https://www.cochranelibrary.com/central/doi/10.1002/central/CN-02238940/full.
40. JPRN-UMIN000034939 (ongoing; last update posted on 31.03.2021)
    - JPRN-UMIN000034939 (2021): Double blinded randomized placebo controlled trial to examine if intake of 5-aminolevulinic acid supplement can improve clinical symptoms of individuals with Autism Spectrum Disorder. Available online at https://www.cochranelibrary.com/central/doi/10.1002/central/CN-02255864/full.
41. NCT04745026 (ongoing; last update posted on 11.06.2021)
    - NCT04745026 (2021): Trial to Investigate the Safety and Efficacy of Cannabidiol Oral Solution (GWP42003-P; CBD-OS) in Children and Adolescents With Autism Spectrum Disorder. Available online at https://www.cochranelibrary.com/central/doi/10.1002/central/CN-02218356/full.
42. NCT04766177 (ongoing; last update posted on 23.02.2021)
    - NCT04766177 (2021): Role of Bumetanide in Treatment of Autism. Available online at https://www.cochranelibrary.com/central/doi/10.1002/central/CN-02236565/full.
43. NCT04878198 (ongoing; last update posted on 11.05.2021)
    - NCT04878198 (2021): Treatment of Sleep Disturbance in Children With ASD. Available online at https://www.cochranelibrary.com/central/doi/10.1002/central/CN-02270250/full.
44. NCT04903353 (ongoing; last update posted on 21.09.2021)
    - NCT04903353 (2021): Pragmatic Trial Comparing Weight Gain in Children With Autism Taking Risperidone Versus Aripiprazole. Available online at https://www.cochranelibrary.com/central/doi/10.1002/central/CN-02289599/full.
45. NCT04939974 (ongoing; last update posted on 25.06.2021)
    - NCT04939974 (2021): Probiotic in Autism. Available online at https://www.cochranelibrary.com/central/doi/10.1002/central/CN-02279189/full.
46. NCT04942522 (ongoing; last update posted on 28.06.2021)
    - NCT04942522 (2021): Application of Probiotic PS128 in Children With ASD. Available online at https://www.cochranelibrary.com/central/doi/10.1002/central/CN-02279247/full.
47. RBR-5wr2cqq (ongoing; last update posted on 17.12.2020)

- RBR-5wr2cqq (2021): Evaluation of the efficacy and safety of medicinal cannabis in children with autism. Available online at https://www.cochranelibrary.com/central/doi/10.1002/central/CN-02241594/full

48. CTRI/2021/07/034901

- CTRI/2021/07/34901. Evaluation of the efficacy of oral Folinic acid supplementation in children with Autism Spectrum disorders: a randomized double blind, placebo controlled trial - FIAT

49. NCT05015439

- NCT05015439. Cannabidiol (CBD) in Adults With ASD.

4.4 Contacting corresponding authors for additional data/clarifications

Study authors of eligible and unclear studies were contacted in our previous analysis of placebo-controlled studies (Siafis et al 2018, *Molecular Autism*). In this analysis, we contacted study authors of 18 head-to-head not placebo-controlled as well as newly identified studies that we had no contacted before, from which we acquired additional data and clarifications for two (except for studies identified during the update searches of August 2020 and September 2021).

	**Study name**	**In the review**	**Contacted**	**Replied**	**Provided additional data and clarifications**	**Comment (up to 16.09.2020)**
1	DeVane_2019	Included in the analysis	Yes	Yes	No	
2	EUCTR2014-001560-35	Included in the analysis	Yes	Yes	No	Most of the data were available in the manuscript
3	Ghanizadeh_2014	Included in the analysis	Yes	Yes	No	No access to the data
4	IRCT2012111011421N1	Unpublished protocol	Yes	No	No	
5	ISRCTN04516575	Unpublished protocol	No	No	No	No working email was found
6	Lamberti_2016	Included in the analysis	Yes	Yes	No	
7	Li_2016	Excluded from the analysis due to unclear methdoology	Yes	No	No	
8	Miral_2008	Included in the analysis	Yes	No	No	
9	NCT00467818	Unpublished protocol	No	No	No	No working email was found
10	NCT02385799	Including in the analysis	Yes	Yes	Yes	
11	NCT02551380	Including in the analysis	Yes	No	No	
12	NCT02674984	Unclear trial for inclusion and ongoing	Yes	No	No	Recruiting
13	NCT02909959	Included in the analysis	Yes	Yes	Yes	
14	NCT03550209	Unpublished protocol	Yes	Yes	No	Data were not shared before publication of the trial
15	Nikvharz_2017	Included in the analysis	Yes	No	No	
16	Reynolds_2019	Included in the analysis	Yes	Yes	No	
17	Vasconcelos_2014	Included in the analysis	Yes	Yes	No	Further analysis of the data is planned
18	Wang_2020	Included in the analysis	Yes	No	No	

eAppendix-5 Study characteristics

5.1.1 Summary of characteristics of the sample 152
5.1.2 Characteristics of included studies for quantitative analysis 155
5.2 Risk of bias 167
5.2.1 Risk of bias of included studies 167
5.2.2 Risk of bias summary of included studies 173
5.2.2.1 Children/adolescents 173
5.2.2.2 Adults/mixed populations 174
5.3. Eligible scales 175
5.3.1 General strategy 175
5.3.2 Table of eligible scales 175
5.4 References 178

5.1 Tables of study characteristics

5.1.1 Summary of characteristics of the sample

k=143 studies, n=8554 participants			
**Age group**		**Children/adolescents (k=125, n=7450)**	**Adults or mixed populations (k=18, n=1104)**
**Study design**	Publication year	2015 [2006-2019], min/max=1984-2021	2016 [2012-2019], min/max=1996-2021
	Design	Parallel (k=97), crossover (k=28) *<br>Double blind (k=120), single-blind (k=2), open (k=3)<br>Placebo-controlled (k=119), no placebo control (k=6)	Parallel(k=13), crossover (k=5) *<br>Double-blind (k=18)<br>Placebo-controlled (k=18)
	Sites	Single-site (k=71)<br>Number of sites in multi-center studies: 5 [3-16], min/max 2-50, n.i. or unclear (k=13)<br>Only academic sites (k=87), for the rest of the studies the percentage of academic sites ranged between 0%-75% with median 47% [5%-61%], n.i. or unclear (k=24)	Single-site (k=14)<br>Number of sites in multi-center studies: 15 [4-32], min/max:2-51<br>Only academic sites (k=15), for the rest of the studies the percentage of academic sites ranged between 0%-67% with median 62% [31%-64%]
	Duration of treatment	12 weeks [8-13], min/max 1-52 weeks<br>Shorter-term studies (1 to 3 weeks, k=7), medium-term studies (4 to 13 weeks, k=89), longer-term studies (>13 weeks, k=29)	12 weeks [6-12], min/max 2-24 weeks<br>Shorter-term studies (1 to 3 weeks, k=1), medium-term studies (4 to 13 weeks, k=15), longer-term studies (>13 weeks, k=2)
	Number of arms and medications	Two arms (k=110), three arms (k=7), four arms (k=9),<br>All of the studies investigated two medications (including placebo or different doses of the same drug) apart from one study that investigated four medications (placebo, omega-3, vitamin-D, vitamin-D + omega-3 in Mazahery 2019).	Two arms (k=15), three arms (k=2), four arms (k=1),<br>All of the studies investigated two medications (including placebo or different doses of the same drug) apart from one study that investigated three medications (clomipramine, haloperidol, placebo in Remington 2001)
	Sample size	42 [24-80], min/max 10-308	34 [20-47], min/max 10-322
	Sponsorship	Academic sponsorship (k=76), financial interest due to industry sponsorship (k=37) or patent application (k=3), n.i. or unclear sponsorship (k=9)	Academic sponsorship (k=9), financial interest due to industry sponsorship (k=6) or patent application (k=3)
**Intervention**	Experimental intervention	<u>Pharmacological (k=84):</u><br>amantadine (k=1), arbaclofen (k=1), aripiprazole (k=8), atomoxetine (k=4, while two arms with atomoxetine + parental training and parental training were not analyzed), balovaptan (k=1), bumetanide (k=4), buspirone (k=1), cannabinoids (k=1), citalopram (k=2), donepezil (k=2, including one study with donepezil + choline), fenfluramine (k=4), fluoxetine (k=4), fluvoxamine (k=1), guanfacine (k=1), haloperidol (k=4), IGOH (k=1), ketamine (k=1), L1-79 (k=1), lamotrigine (k=1), levetiracetam (k=1), lurasidone (k=1), mecamylamine (k=1), melatonin (k=4, while two arms with melatonin + CBT and CBT were not investigated), memantine (k=4), methylphenidate (k=3), mirtazapine (k=1), naltrexone (k=5), olanzapine (k=2), ORG-2766 (k=2), oxytocin	<u>Pharmacological (k=16)</u><br>balovaptan (k=2), citalopram (k=1), clomipramine (k=1), dextromethorphan/quinidine (k=1), haloperidol (k=1), ketamine (k=1), milnacipran (k=1), oxytocin (k=6), risperidone (k=1)<br><br><u>Dietary supplements (k=2)</u><br>sulforaphane (k=2)

		(k=6), prednisolone (k=1), riluzole (k=1), risperidone (k=10), sertraline (k=1), simvastatin (k=1), tianeptine (k=1), tideglusib (k=1), valproate/divalproex (k=3)<br>Dietary supplements (k=41):<br>carnitine/carnosine (k=4), cholesterol (k=1), digestive enzymes (k=1), dimethylglycine (k=1), ferrous (k=1), folinic acid (k=2), gluten-casein supplement (k=1), inositol (k=1), n-acetylcysteine (k=3), pre/probiotics (k=5), omega-3 (k=11, while an arm with vitamin-D + omega-3 was not analyzed), pyridoxine+Mg (k=1), saproptertin (k=2), sulforaphane (k=1), vitamin-B12 (k=2), vitamin-D (k=4, while three arms with vitamin-D + omega-3, perceptual exercise + vitamin D and perceptual exercise were not analyzed), whey-protein (k=1)	
	Dose administration	Flexible schedule (k=52), fixed (k=70), n.i. (k=4)	Flexible schedule (k=8), fixed (k=10), n.i. (k=4)
	Route of administration	Oral (k=117), intranasal (k=6), subcutaneous (k=2)	Oral (k=11), intranasal (k=7)
**Participants**	Diagnosis	Standardized diagnostic criteria (k=118):<br>DSM-III (k=15), DSM-IV (k=66), DSM-5 (k=29), ICD-10 (k=4), DSM version n.i. (k=2), DSM was assumed (k=2)<br>*Studies could have used more than one diagnostic criteria*<br>Studies that used only diagnostic tools (k=7):<br>ADI-R and/or ADOS (k=3), CARS (k=3), SCQ + clinical diagnosis (k=1, validation of the method was reported**)	Standardized diagnostic criteria (k=18):<br>DSM-III (k=1), DSM-IV (k=13), DSM-5 (k=4)
	Age	8.23 years [6.26-9.51], min/max of mean age 3.62-14.64 years, n.i. (k=5)	24.55 years [21.92-27.91], min/max of mean age 16.33-34.31 years, n.i. (k=1)
	Sex	Percentage of female participants was 16.37% [11.95-20.42%], min/max 0-50%, n.i. (k=7)	Percentage of female participants was 10% [0-20.25%], min/max 0-30%, n.i. (k=1)
	Intellectual disability	Percentage of participants with intellectual impairment was 56% [31.74-83.77%], min/max 0-100%, n.i. (k=87).<br>The mean IQ was 71.6 [62.44-84.31], min/max 40-102.5, n.i. (k= 86). Different scales and versions were used within and between studies.	Percentage of participants with intellectual impairment was 0% [0-8.11%], min/max 0-100%, n.i. (k=5).<br>The mean IQ was 102.14 [79.9-105.55], min/max 30.98-108, n.i. (k=5). Different scales and versions were used within and between studies.
	Associated symptoms	Associated symptoms as inclusion criteria (k=45): Irritability (k=15), ADHD symptoms (k=10), irritability and hyperactivity (k=1), anxiety (k=1), disruptive behaviors (k=1), difficulty in motor skills (k=1), gastrointestinal symptoms (k=1), gastrointestinal symptoms and anxiety (k=1), language impairment (k=2), lower scores in executive functions (k=1), lower levels of vitamin-D (k=2), lower levels of cholesterol (k=1), lower levels of tetrahydrobiopterin in CSF (k=1), maladaptive behaviors and high urine levels of I-FABP (k=1), sleep disorders (k=5), sleep disorders and low levels of ferritin (k=1). Not indicated or unclear (k=2)<br>Genetic syndrome as inclusion criteria (k=1, neurofibromatosis type 1)	Associated symptoms as inclusion criteria (k=2): Irritability or labile emotional state (k=1), self-injurious behavior (k=1)

	Baseline severity	Baseline CGI-Severity 4.87 [4.52-5.13], min/max 3.74-5.8, n.i. (k=82) Baseline ABC-Irritability 18.23 [13.99-23.24], min/max 11.01-29.91, n.i, (k=86) Minimum threshold of ASD core symptoms for inclusion (k=9): SRS (k=2), ABC-L/SW (k=2), CARS (k=1), CPRS items (k=1), C-YBOCS, C-YBOCS-PDD (k=3)***	Baseline CGI-Severity 4.4 [4.2-4.5], min/max 4.1-5.9, n.i. (k=9) Baseline ABC-Irritability 11.41 [8.73-14.04], min/max 8.07-14.56, n.i, (k=14) Minimum threshold of ASD core symptoms for inclusion (k=5): SRS (k=2), ABC-L/SW (k=1), CYBOCS or YBOCS (k=1), YBOCS, RLRS or SIBQ (k=1)***
**Scales**	Social-communication difficulties	69 studies: ABC-L/SW or ABC-SW (k=38), ADOS-SI (k=5), ATEC-S (k=3), BASC-S (k=1), CBCL-Social (k=1), CCC-2-SIDC (k=2), GARS—SI (k=2), SRS-SC (k=4), VABS-S (k=13)	8 studies: ABC-L/SW (k=4), ADOS-SI (k=2), VABS-S (k=2)
	Repetitive behaviors and restricted interests	60 studies: ABC-S (k=30), ADOS-RRBI (k=4), CYBOCS-PDD or similar versions (k=14), GARS-S (k=3), PDDBI-RRBI (k=1), RBS (k=5), SRS-RRBI (k=3)	14 studies: ABC-S (k=5), ADOS-RRBI (k=2), YBOCS or modifications (k=6), RBS (k=1)
	Overall core symptoms	55 studies: ADOS (k=7), CARS (k=12), CPRS-AF (k=2), CSBQ (k=1), GARS (k=2), PDDBI (k=2), RLRS (k=4), SRS (k=25)	9 studies: ADOS (k=1), CARS (k=2), RLRS (k=1), SRS (k=5)
	Irritability	59 studies: ABC-I (k=50), CBCL-Aggression (k=3), CPRS-Anger or Conduct problems (k=2), DBC-Disruptive (k=2), HSQ (k=1), SDQ-CP (k=1)	7 studies: ABC-I (k=5), POMS-Anger (k=1), SIB-Q (k=1)
	ADHD symptoms	50 studies: ABC-H (k=38), ADHD-RS (k=5), CBCL-ADHD (k=1), CPRS-H (k=3), DBC-ADHD (k=1), SDQ-H (k=1), SNAP-IV-ADHD (k=1)	7 studies: ABC-H (k=7)
	Anxiety or depressive symptoms	18 studies: BASC-I (k=2), CASI (k=2), CBCL-I (k=3), DBC-Anxiety (k=2), NCBRF-insecure/anxious (k=1), PARS (k=2), PAS-R (k=1), PRAS-ASD (k=1), SCAS (k=2), SDQ-Emotional (k=1), VAS-Anxiety (k=1)	7 studies: HAM-A (k=1), POMS-Depression (k=1), STAI-State (k=4), VAS-Anxiety (k=1)
	Caregiver stress	14 studies: APSI (k=1), CGSQ (k=2), CSQ (k=3), NOSI (k=1), PedsQL-Family Impact (k=1), PSI (k=5), WHO-5 (k=1)	2 studies: PedsQL-Family Impact (k=2)
	Global functioning	6 studies: CGAS or similar versions (k=6)	3 studies: GAF (k=3)
	Quality of life	5 studies: PedsQL or similar versions (k=5)	5 studies: PedsQL (k=2), WHO-QOL (k=3)

The median of values are presented (of summary data), [] = interquartile ranges, min/max =minimum and maximum of the study-level average values are presented, k= number of studies, n.i. = not indicated or unclear, IGOH= oral human immunoglobulin, CBT=Cognitive-behavioral therapy, PT=Parent Training

*only crossover studies with available data before the crossover

**Lee H, Marvin AR, Watson T, et al. Accuracy of phenotyping of autistic children based on Internet implemented parent report. Am J Med Genet B Neuropsychiatr Genet. 2010;153B(6):1119-1126.

***When scales used only for the confirmation of a diagnosis (such as SRS T-score of 60, CARS score of 30) were not considered as a minimum threshold of symptoms.

5.1.2 Characteristics of included studies for quantitative analysis

ID	Study name	Intervention	n	Duration (weeks)	Blinding (0: open, 1: single-blind, 2: double-blind)	Diagnostic criteria or tools	Age (years)	Participant subgroup defined per inclusion criteria (associated condition, intellectual function, genetic syndrome)
1	Akkok_1995	placebo	9	2	1	DSM-III-R	6.3	
		naltrexone	11				6.3	
2	Aman_2017	placebo	61	12	2	DSM-IV-TR	8.9	participants with irritability were excluded
		memantine	60				9	
3	Amminger_2007	placebo	6	6	2	DSM-IV	12.1	Irritability and no other psychotropic medication
		omega-3	7				10.5	
4	Anagnostou_2012	placebo	9	6	2	DSM-IV-TR	32.9	high functioning
		oxytocin	10				33.8	
5	Anderson_1989	placebo	30	4	2	DSM-III	4.5	hypoactive or low energic participants were excluded
		haloperidol	15				4.5	
6	Arnold_2006	placebo	7	6	2	DSM-IV	9.3	ADHD symptoms
		atomoxetine	9				9.3	
7	Arnold_2012	placebo	8	14	2	DSM-IV	8.4	
		mecamylamine	12				6.8	
8	Arnold_2019	placebo	4	8	2	DSM-5	8.8	gastrointestinal symptoms and anxiety
		probiotics	6				8.8	
9	Barthelemy_1989	placebo	6	4	2	DSM-III	6.3	intellectual disability and no other psychotropic medication
		fenfluramine	7				6.3	
10	Belsito_2001	placebo	16	12	2	(ADI-R, ADOS, CARS)	6.1	
		lamotrigine	19				5.6	
11	Bent_2011	placebo	13	12	2	DSM-IV-TR	5.8	
		omega-3	14				5.8	
12	Bent_2014	placebo	28	6	2	(SCQ and a previous	7.1	ADHD symptoms
		omega-3	29				7.3	

						clinical diagnosis)		
13	Bernaerts_2020	placebo	18	4	2	DSM-IV-TR	24	high functioning
		oxytocin	22				25	
14	Bertoglio_2010	placebo	17	6	2	DSM-IV-TR	(3-8)	
		vitamin-B12	13				(3-8)	
15	Bolognani_2019	placebo	75	12	2	DSM-5, ICD-10	24.7	high functioning
		balovaptan 1.5 mg/d	32				28.2	
		balovaptan 4 mg/d	77				24.5	
		balovaptan 10 mg/d	39				23.9	
16	Bouvard_1995	placebo	5	4	2	DSM-III-R, ICD-10	9.5	
		naltrexone	5				9.5	
17	Buietlaar_1990	placebo	7	4	2	DSM-III-R	8.8	
		ORG-2766	7				8.7	
18	Buitelaar_1992	placebo	11	8	2	DSM-III-R	10.3	
		ORG-2766	10				10.3	
19	Campbell_1982	placebo	20	4	2	DSM-III	4.6	hypoactive or low energic participants were excluded
		haloperidol	20				4.6	
20	Campbell_1987	placebo	14	8	2	DSM-III	4.6	
		fenfluramine	14				4.6	
21	Campbell_1993	placebo	18	3	2	DSM-III-R	4.9	
		naltrexone	23				4.9	
22	Chez_2017	placebo	7	8	2	DSM-IV-TR	21.9	Irritability or emotional lability
		dextromethorphan + quinidine	7				21.9	
23	Chugani_2016	placebo	57	24	2	DSM-IV-TR	3.6	
		buspirone 2.5mg/day	54				3.5	
		buspirone 5 mg/day	55				3.8	
24	Cortesi_2012	placebo	40	12	2	DSM-IV-TR	6.3	sleep disorders
		CBT	40				7.1	
		melatonin	40				6.8	

		melatonin+CBT	40				6.4	
25	Danfors_2005	placebo	6	26	2	DSM-IV	4.9	low tetrahydrobiopterin levels in CSF
		sapropterin	6				5.7	
26	Dean_2017	placebo	51	24	2	DSM-IV-TR	6.2	
		n-acetylcysteine	51				6.5	
27	DeVane_2019	aripiprazole	31	10	2	DSM-IV	8.5	irritability
		risperidone	30				8.3	
28	Ekman_1989	placebo	10	16	2	DSM-III, DSM-III-R	6.2	
		fenfluramine	10				6.2	
29	EUCTR2014-001560-35	placebo	45	13	2	DSM-IV-TR, DSM-5	10.2	
		bumetanide	47				10.5	
30	Fahmy_2013	placebo	14	26	2	(CARS)	5.7	
		carnosine	16				5.8	
31	Findling_1997	placebo	5	4	2	DSM-III-R	6.3	
		pyridoxine+Mg	5				6.3	
32	Frye_2018	placebo	25	12	2	DSM (version not specified)	7.2	language impairment and participants with irritability were excluded
		folinic acid	23				7.6	
33	Gabis_2019	placebo	31	12	2	DSM-IV-TR, DSM-5	9.5	
		donepezil+choline	29				9.5	
34	Geier_2011	placebo	11	13	2	(CARS)	6.7	
		carnosine	19				6.3	
35	Ghanizadeh_2014	aripiprazole	29	8	2	DSM-IV-TR	9.6	
		risperidone	30				9.5	
36	Ghodsi_2018	placebo	22	8	2	DSM-5	8.4	
		carnosine	22				8.9	
37	Ghuman_2009	placebo	6	2	2	DSM-IV-TR	4.8	ADHD symptoms
		methylphenidate	6				4.8	
38	Gringras_2017	placebo	65	13	2	DSM-IV-TR, DSM-5, ICD-10	8.4	sleep disorders
		melatonin	60				9	
39	Guastella_2015	placebo	24	8	2	DSM-IV-TR	14	

		oxytocin	26				13.8	
40	Handen_2000	placebo	4	1	2	(CARS)	7.4	ADHD symptoms
		methylphenidate 0.3mg/kg/d	9				7.4	
41	Handen_2009	placebo	31	12	2	DSM-IV-TR	6.2	gastrointestinal symptoms
		IGOH 140mg/day	32				7.4	
		IGOH 420mg/day	31				8	
		IGOH 840mg/day	31				7.6	
42	Handen_2012	placebo	16	10	2	DSM-IV-TR	11.7	high functioning and lower scores in executive function system
		donepezil	18				11.5	
43	Handen_2015	placebo	32	10	2	DSM-IV-TR	8.2	ADHD symptoms
		atomoextine+PT	32				8	
		atomoxetine	32				8.6	
		PT	32				7.7	
44	Hardan_2012	placebo	18	12	2	DSM-IV-TR	7.2	irritability
		n-acetylcysteine	15				7	
45	Harfterkamp_2013	placebo	49	8	2	DSM-IV-TR	10	ADHD symptoms
		atomoxetine	48				9.9	
46	Hellings_2005	placebo	14	8	2	DSM-IV	12.1	Irritability
		valproate	16				10.3	
47	Hendren_2016	placebo	29	8	2	DSM-IV	4.8	
		vitamin-B12	28				5.6	
48	Herscu_2019	placebo	80	14	2	DSM-IV-TR	8.9	participants with irritability were excluded
		fluoxetine	78				9.1	
49	Hollander_2005	placebo	20	8	2	DSM-IV-TR	7.3	
		fluoxetine	19				9.1	
50	Hollander_2006	placebo	4	8	2	DSM-IV	9.5	
		divalproex	9				9.5	
51	Hollander_2006b	placebo	5	8	2	DSM-IV	8.9	
		olanzapine	6				9.2	

52	Hollander_2010	placebo	11	12	2	DSM-IV-TR	9	Irritability
		divalproex	16				9.7	
53	Hollander_2012	placebo	15	12	2	DSM-IV	38	
		fluoxetine	22				31.8	
54	Ichikawa_2017	placebo	45	8	2	DSM-IV-TR	9.9	Irritability
		aripiprazole	47				10.3	
55	Kent_2013	placebo	35	6	2	DSM-IV-TR	9	Irritability
		risperidone 0.125mg/day	30				10	
		risperidone 1.25mg/day	31				9	
56	Kerley_2017	placebo	20	20	2	DSM (version not specified)	6.9	
		vitamin-D	22				7.9	
57	Kern_2001	placebo	19	4	2	DSM-IV	(3-11)	
		dimethylglycine	18				(3-11)	
58	King_2001	placebo	20	4	2	DSM-IV, ICD-10	7	irritability and ADHD symptoms
		amantadine	19				7	
59	King_2009	placebo	76	12	2	DSM-IV-TR	9.6	participants with irritability were excluded
		citalopram	73				9.1	
60	Klaiman_2013	placebo	23	16	2	DSM-IV-TR	5	participants with irritability were excluded
		sapropterin	23				5	
61	Kosaka_2016	placebo	20	12	2	DSM-IV-TR	24.9	high functioning
		oxytocin 16IU/day	20				23.3	
		oxytocin 32IU/day	20				24.8	
62	Lamberti_2016	aripiprazole	22	24	0	DSM-5	8.4	ADHD symptoms
		risperidone	22				7.9	
63	Lemonnier_2012	placebo	30	13	2	ICD-10	7.1	
		bumetanide	30				6.9	
64	Lemonnier_2017	placebo	23	13	2	ICD-10	8.9	
		bumetanide 1mg/day	20				7.8	
		bumetanide 2mg/day	23				7.9	
		bumetanide 4mg/day	22				8.4	

65	Leventhal_1993	placebo	8	16	2	DSM-III	7.6	intellectual disability
		fenfluramine	7				7.6	
66	Levine_1997	placebo	5	4	2	DSM-III-R	6	intellectual disability
		inositol	5				5.2	
67	Liu_2019	placebo	41	4	2	DSM-5	9.9	
		probiotic	39				10.1	
68	Loebel_2016	placebo	50	6	2	DSM-IV-TR	11	Irritability
		lurasidone 20mg/day	49				10.5	
		lurasidone 60mg/day	51				10.5	
69	Malone_2001	haloperidol	6	6	0	DSM-IV	7.3	Irritability
		olanzapine	6				8.5	
70	Mankad_2015	placebo	19	24	2	DSM-IV-TR	3.5	
		omega-3	19				3.9	
71	Marcus_2009	placebo	52	8	2	DSM-IV-TR	10.2	Irritability
		aripiprazole 5mg/day	53				9	
		aripiprazole 10mg/day	59				10	
		aripiprazole 15mg/day	54				9.5	
72	Mazahery_2019	placebo	29	52	2	DSM-5	5.5	low vitamin-D in serum
		omega-3	29				5	
		vitamin-D	31				5.2	
		vitamin-D+omega-3	28				5.2	
73	McDougle_1996	placebo	15	12	2	DSM-III-R	30.1	
		fluvoxamine	15				30.1	
74	McDougle_1998	placebo	16	12	2	DSM-IV	29.7	YBOCS, RLRS or SIBQ for inclusion (not considered for indirectness)
		risperidone	15				26	
75	McDougle_2000	placebo	16	12	2	(diagnosis not specified; most probably with DSM-IV)	9.5	
		fluvoxamine	18				9.5	
76	Mehrazad_2018	placebo	25	8	2	DSM-V	8.3	sleep disorders
		carnosine	25				8.6	

77	Miral_2008	haloperidol	15	10	2	DSM-IV	10.9	
		risperidone	15				10	
78	Moradi_2018	placebo	25	13	2	DSM-5	7.2	high functioning and low levels of vitamin-D in serum
		perceptual exercise	25				7.6	
		vitamin-D	25				8	
		vitamin-D + perceptual exercise	25				7.6	
79	Munasinghe_2010	placebo	22	13	2	DSM-IV-TR	5.8	
		digestive enzymes	21				5.7	
80	Munesue_2016	placebo	14	8	2	DSM-IV-TR	22.4	intellectual disability
		oxytocin	15				22.6	
81	Nagaraj_2006	placebo	21	24	2	DSM-IV	5.2	
		risperidone	19				4.8	
82	NCT00183339	placebo	10	52	2	DSM-IV-TR	3.7	
		fluoxetine	8				3.5	
83	NCT00198107	placebo	41	8	2	DSM-IV	9.4	Irritability
		aripiprazole	40				9	
84	NCT00498173	placebo	31	8	2	DSM-IV	8.4	ADHD symptoms and exclusion of extreme aggression or self-injury
		atomoxetine	29				9.3	
85	NCT00609531	placebo	6	12	2	DSM-IV-TR	(10-55)	high functionin
		citalopram	6				(10-55)	
86	NCT00870727	placebo	16	8	2	DSM-IV-TR	9.5	Irritability
		aripiprazole	17				9.8	
87	NCT01302964	placebo	10	10	2	DSM-IV-TR	11.3	anxiety
		mirtazapine	20				10.9	
88	NCT01308749	placebo	13	8	2	DSM-IV	10	
		oxytocin	12				10.6	
89	NCT01624675	placebo	18	8	2	DSM-IV-TR	7	irritability
		risperidone	21				8	
90	NCT01661855	placebo	29	12	2	DSM-IV	11.5	

		riluzole	29				11.6	
91	NCT01908205	placebo	30	12	2	DSM-IV	12.4	high functioning
		oxytocin	30				12.5	
92	NCT01972074	placebo	21	12	2	DSM-5	13.3	high functioning
		memantine	22				13.2	
93	NCT02385799	placebo	26	26	2	DSM-5	3.7	
		sertraline	32				4.3	
94	NCT02551380	placebo	10	12	1	(ADOS)	6.4	language impairment and participants with irritability were excluded
		folinic acid	9				6.4	
95	NCT02586935	placebo	40	12	2	DSM-5	(12-17)	
		tideglusib	40				(12-17)	
96	NCT02909959	placebo	24	12	2	DSM-5	17.7	
		sulforaphane	24				16.8	
97	NCT02947048	placebo	10	4	2	DSM-5	(13-21)	
		L1-79 300mg/d	10				(13-21)	
		L1-79 600mg/day	10				(13-21)	
98	NCT02956226	placebo	50	12	2	DSM-5	11.8	disruptive behaviors
		cannabinoids pure mix	50				11.8	
		cannabinoids whole plant	50				11.8	
99	Niederhofer_2003	placebo	6	6	2	ICD-10	7.3	No tolerance or response to previous psychopharmacological treatments
		tianeptine	6				7.3	
100	Nikvarz_2017	memantine	18	8	0	DSM-IV-TR	6.8	
		risperidone	16				6.6	
101	Noone_2014	placebo	5	12	2	DSM-IV-TR	25.2	high functioning
		milnacipran	5				25	
102	Owen_2009	placebo	51	8	2	DSM-IV-TR	8.8	irritability
		aripiprazole	47				9.7	
103	Parellada_2017	placebo	37	8	2	DSM-IV-TR	10	
		omega-3	40				9.4	
104	Parker_2017	placebo	18	4	2		8.1	

		oxytocin	17			DSM-IV-TR, DSM-5	9.3	
105	Pearson_2013	placebo	6	1	2	DSM-IV-TR	8.8	ADHD symptoms
		methylphenidate low dose	6				8.8	
		methylphenidate medium dose	6				8.8	
		methylphenidate high dose	6				8.8	
106	Pusponegoro_2015	placebo	36	1	2	DSM-IV-TR	5.1	maladaptive behaviors and high levels of I-FABP in urine
		gluten-casein	38				5.4	
107	Reddihough_2019	placebo	71	16	2	DSM-IV-TR, DSM-5	11.2	
		fluoxetine	75				11.3	
108	Remington_2001	placebo	10	6	2	DSM-IV	18.5	
		clomipramine	13				16.8	
		haloperidol	13				14.2	
109	Reynold_2019	placebo	11	12	2	DSM-IV-TR	5.7	sleep disorders and low level of ferritin
		ferrous	9				6	
110	RUPP_2002	placebo	52	8	2	DSM-IV	9.1	irritability
		risperidone	49				8.6	
111	Scahill_2015	placebo	32	8	2	DSM-IV	8.5	ADHD symptoms
		guanfacine	30				8.5	
112	Scifo_1991	placebo	6	15	2	DSM-III-R	11.2	intellectual disability
		naltrexone	6				11.2	
113	Shea_2004	placebo	39	8	2	DSM-IV	7.3	
		risperidone	41				7.6	
114	Singh_2014	placebo	15	18	2	DSM-IV-TR	16.6	
		sulforaphane	29				17.9	
115	Stivaros_2018	placebo	16	12	2	(ADI-R, ADOS, SRS)	8.3	genetic syndrome (neurofibromatosis type I)
		simvastatin	14				7.9	
116	Vasconcelos_2014	placebo	20	24	2	DSM-IV-TR	4.6	
		prednisolone	20				4.8	
117	VeenstraVanderWeele_2017	placebo	74	12	2	DSM-IV-TR	11.7	

		arbaclofen	76				11.4	
118	Voigt_2014	placebo	24	26	2	DSM-IV	6.5	
		omega-3	24				5.8	
119	Wang_2020	placebo	10	15	2	CCMD-3, DSM-5	4.3	participants with hyperactivity were excluded
		probiotics	16				4.3	
120	Wasserman_2006	placebo	10	10	2	DSM-IV	9.8	
		levetiracetam	10				7.6	
121	Watanabe_2015	placebo	10	6	2	DSM-IV-TR	29.3	high functioning
		oxytocin	10				35.1	
122	Willemsen_1996	placebo	12	4	2	DSM-III-R	5.5	
		naltrexone	11				5.5	
123	Wink_2016	placebo	15	12	2	DSM-IV	8.2	
		n-acetylcysteine	16				7.6	
124	Wink_2020	placebo	10	2	2	DSM-5	19.5	
		ketamine	11				19.5	
125	Wright_2011	placebo	11	13	2	ICD-10	9	sleep disorders
		melatonin	9				9	
126	Yamasue_2018	placebo	53	6	2	DSM-IV-TR	26.3	high functioning
		oxytocin	53				27.6	
127	Yatawara_2016	placebo	22	5	2	DSM-IV-TR	6.7	
		oxytocin	17				5.7	
128	Yui_2013	placebo	6	16	2	DSM-IV	15.5	high functioning
		omega-3	7				13.9	
129	IRCT20131013014994N5	placebo	26	15	2	DSM-5	8.95	
		Vitamin-D	26				8.88	
130	Santocchi_2019	placebo	43	26	2	DSM-5	4.13	
		probiotics	42				4.16	
131	NCT01372449	placebo	11	24	2	DSM-IV-TR, DSM-5	9.64	difficulty in motor skills and participants with irritability were excluded
		memantine	12				9.25	
132	NCT01944046	placebo	144	24	2	DSM-5	10.4	

		oxytocin	146				10.4	
133	NCT02901431	placebo	81	24	2	DSM-5, ICD-10	12.5	high functioning
		balovaptan 4mg/d (unclear information for this arm, not included in the analysis)	n.i.				n.i.	
		balovaptan 10mg/d	86				12.53	
134	NCT03156153	placebo	60	13	2	DSM-5	4.22	
		bumetanide	60				4.03	
135	NCT03337035	placebo	18	16	2	DSM-IV-TR, DSM-5	9.85	
		probiotics	17				10.7	
136	NCT03504917	placebo	158	24	2	DSM-5	27.6	high functioning
		balovaptan	163				27.9	
137	NCT00965068	placebo	7	12	2	DSM-IV	6.3	low levels of cholesterol
		cholesterol	8				6.9	
138	NCT03550209	placebo	35	13	2	DSM-5	(2-6)	
		omega-3 25mg/d	12					
		omega-3 50mg/d	12					
		omega-3 75mg/d	13					
139	Doaei_2021	placebo	26	8	2	DSM-IV-TR	8.2	
		omega-3	28				8.1	
140	Hayashi_2021	placebo	66	2	2	DSM-5	10.8	sleep difficulties
		melatonin 1mg/d	65				10.8	
		melatonin 4mg/d	65				12	
141	Zimmerman_2021	placebo	29	15	2	DSM-5	7	
		sulforaphane	28				7.4	
142	NCT00467818	placebo	8	12	2	(diagnosis not specified; most probably with DSM-IV)	10.6	
		omega-3	9				11.7	
143	Castejon_2021	whey protein	22	1	2	DSM-IV, DSM-5	3.9	
		placebo	24				3.9	

Arms in *italics* were excluded from the analysis irrespective of data availability: parental treatment, atomoxetine + parental treatment, CBT, melatonin + CBT, vitamin-D + omega-3, perceptual exercise, perceptual exercise + vitamin-D

5.2 Risk of bias

5.2.1 Risk of bias of included studies

	Study name	Random sequence generation	Allocation concealment	Blinding of participants and personnel	Blinding of outcome assessors	Incomplete outcome data	Selective reporting	Other biases	Overall
children/adolescents	Akkok_1995	unclear	unclear	high	unclear	low	low	unclear	moderate
	Aman_2017	low	low	unclear	unclear	low	low	unclear	moderate
	Amminger_2007	unclear	unclear	low	low	high	low	high	high
	Anderson_1989	unclear	unclear	low	low	unclear	unclear	unclear	moderate
	Arnold_2006	unclear	unclear	low	low	low	high	unclear	moderate
	Arnold_2012	low	low	unclear	unclear	low	low	high	moderate
	Arnold_2019	low	low	low	low	high	low	low	moderate
	Barthelemy_1989	unclear	unclear	unclear	unclear	low	high	unclear	moderate
	Belsito_2001	low	low	low	low	high	high	unclear	high
	Bent_2011	low	low	low	low	low	low	low	low
	Bent_2014	low	low	low	low	low	low	unclear	low
	Bertoglio_2010	low	low	unclear	unclear	low	high	unclear	moderate
	Bouvard_1995	unclear	unclear	unclear	unclear	low	high	unclear	moderate
	Buietlaar_1990	unclear	unclear	low	low	low	high	low	moderate
	Buitelaar_1992	unclear	unclear	low	low	high	high	unclear	high
	Campbell_1982	unclear	unclear	low	low	unclear	high	unclear	moderate
	Campbell_1987	unclear	unclear	low	low	unclear	high	high	high
	Campbell_1993	unclear	unclear	unclear	unclear	unclear	high	unclear	moderate
	Castejon_2021	low	low	unclear	unclear	high	high	high	high
	Chugani_2016	low	unclear	unclear	unclear	low	low	high	moderate
	Cortesi_2012	low	low	low	low	high	high	unclear	high
	Danfors_2005	unclear	low	low	low	low	high	high	high
	Dean_2017	low	low	low	low	low	low	low	low
	DeVane_2019	unclear	unclear	low	low	high	high	low	high

	Doaei_2021	low	low	unclear	unclear	low	low	low	low
	Ekman_1989	unclear	unclear	unclear	unclear	high	unclear	unclear	moderate
	EUCTR2014-001560-35	low	low	low	low	high	low	low	moderate
	Fahmy_2013	low	unclear	low	low	unclear	low	high	moderate
	Findling_1997	unclear	unclear	low	low	low	high	unclear	moderate
	Frye_2018	low	low	low	low	low	low	high	moderate
	Gabis_2019	unclear	low	low	low	high	high	unclear	high
	Geier_2011	low	unclear	low	low	high	low	low	moderate
	Ghanizadeh_2014	unclear	unclear	high	unclear	low	low	low	moderate
	Ghodsi_2018	low	unclear	unclear	unclear	high	high	unclear	high
	Ghuman_2009	unclear	unclear	low	low	low	high	unclear	moderate
	Gringras_2017	low	low	low	low	low	high	low	moderate
	Guastella_2015	low	low	low	low	low	low	low	low
	Handen_2000	unclear	unclear	unclear	unclear	low	high	unclear	moderate
	Handen_2009	low	unclear	low	low	low	high	unclear	moderate
	Handen_2012	unclear	low	unclear	unclear	low	low	low	moderate
	Handen_2015	low	low	low	low	low	low	low	low
	Hardan_2012	low	low	low	low	low	low	low	low
	Harfterkamp_2013	low	low	low	low	low	low	high	moderate
	Hayashi_2021	low	low	low	low	low	low	unclear	low
	Hellings_2005	unclear	low	unclear	unclear	unclear	high	low	moderate
	Hendren_2016	low	low	unclear	unclear	low	low	low	low
	Herscu_2019	low	low	low	low	low	low	low	low
	Hollander_2005	unclear	unclear	unclear	unclear	unclear	unclear	low	moderate
	Hollander_2006	unclear	unclear	low	low	low	unclear	low	moderate
	Hollander_2006b	unclear	unclear	low	low	unclear	high	unclear	moderate
	Hollander_2010	unclear	unclear	low	low	low	high	unclear	moderate
	Ichikawa_2017	unclear	low	unclear	unclear	low	low	low	moderate

	IRCT20131013014994N5	low	low	low	low	high	low	low	moderate
	Kent_2013	low	unclear	low	low	low	high	low	moderate
	Kerley_2017	unclear	low	low	low	high	low	low	moderate
	Kern_2001	unclear	low	low	low	high	high	high	high
	King_2001	unclear	unclear	low	low	low	high	low	moderate
	King_2009	low	unclear	low	low	low	low	low	low
	Klaiman_2013	low	low	low	low	low	low	high	moderate
	Lamberti_2016	low	unclear	high	high	unclear	high	low	high
	Lemonnier_2012	low	low	low	low	high	low	low	moderate
	Lemonnier_2017	low	low	low	low	low	low	low	low
	Leventhal_1993	unclear	unclear	unclear	unclear	low	high	unclear	moderate
	Levine_1997	low	low	low	low	low	low	low	low
	Liu_2019	low	low	low	low	high	low	low	moderate
	Loebel_2016	low	unclear	low	low	low	low	low	low
	Malone_2001	low	unclear	high	high	low	low	low	high
	Mankad_2015	low	low	low	low	low	low	low	low
	Marcus_2009	low	low	unclear	unclear	low	low	low	low
	Mazahery_2019	low	low	low	low	high	low	high	high
	McDougle_2000	unclear	unclear	unclear	unclear	unclear	unclear	unclear	moderate
	Mehrazad_2018	low	unclear	low	low	high	low	low	moderate
	Miral_2008	unclear	unclear	unclear	unclear	high	high	low	high
	Moradi_2018	unclear	unclear	unclear	unclear	unclear	high	unclear	moderate
	Munasinghe_2010	low	low	low	low	high	high	unclear	high
	Nagaraj_2006	low	low	low	low	low	low	low	low
	NCT00183339	unclear	unclear	unclear	unclear	unclear	high	unclear	moderate
	NCT00198107	unclear	unclear	unclear	unclear	low	low	unclear	moderate
	NCT00467818	unclear	unclear	unclear	unclear	unclear	unclear	unclear	moderate
	NCT00498173	unclear	unclear	low	low	unclear	low	unclear	moderate
	NCT00870727	unclear	unclear	low	low	low	low	unclear	moderate

	NCT00965068	unclear	unclear	unclear	unclear	unclear	low	high	moderate
	NCT01302964	unclear	unclear	low	low	low	high	unclear	moderate
	NCT01308749	unclear	unclear	unclear	unclear	low	low	unclear	moderate
	NCT01372449	low	low	low	low	low	high	low	moderate
	NCT01624675	unclear	low	low	low	low	high	low	moderate
	NCT01661855	unclear	unclear	unclear	unclear	unclear	unclear	unclear	moderate
	NCT01908205	unclear	unclear	unclear	unclear	unclear	unclear	unclear	moderate
	NCT01944046	low	low	low	low	low	low	low	low
	NCT01972074	low	low	low	low	low	unclear	unclear	low
	NCT02385799	low	low	low	low	low	low	low	low
	NCT02551380	unclear	unclear	high	unclear	low	high	low	high
	NCT02586935	unclear	unclear	unclear	unclear	unclear	unclear	unclear	moderate
	NCT02901431	low	low	low	low	high	unclear	high	high
	NCT02947048	unclear	unclear	unclear	unclear	unclear	unclear	unclear	moderate
	NCT02956226	low	low	low	low	high	high	low	high
	NCT03156153	low	low	low	low	low	low	low	low
	NCT03337035	low	low	low	low	unclear	low	low	low
	NCT03550209	low	low	low	low	unclear	unclear	unclear	moderate
	Niederhofer_2003	unclear	unclear	low	low	unclear	high	unclear	moderate
	Nikvarz_2017	unclear	unclear	high	high	high	low	low	high
	Owen_2009	low	low	unclear	unclear	low	low	low	low
	Parellada_2017	low	low	low	low	high	low	low	moderate
	Parker_2017	low	low	low	low	high	low	low	moderate
	Pearson_2013	unclear	unclear	unclear	unclear	low	high	unclear	moderate
	Pusponegoro_2015	unclear	low	low	low	high	low	low	moderate
	Reddihough_2019	low	low	low	low	high	low	high	high
	Reynold_2019	low	low	low	low	low	high	low	moderate
	RUPP_2002	low	low	low	low	low	low	low	low
	Santocchi_2019	low	low	low	low	high	low	low	moderate

	Scahill_2015	low	low	low	low	low	low	low	low
	Scifo_1991	low	low	low	low	unclear	unclear	unclear	moderate
	Shea_2004	low	low	unclear	unclear	low	low	low	low
	Stivaros_2018	low	low	unclear	unclear	low	low	low	low
	Vasconcelos_2014	low	low	low	low	high	high	unclear	high
	VeenstraVander Weele_2017	low	low	low	low	low	low	low	low
	Voigt_2014	low	low	low	low	high	high	unclear	high
	Wang_2020	low	unclear	unclear	unclear	high	low	low	moderate
	Wasserman_2006	unclear	unclear	low	low	unclear	high	unclear	moderate
	Willemsen_1996	unclear	unclear	unclear	unclear	low	high	unclear	moderate
	Wink_2016	low	low	low	low	high	low	low	moderate
	Wright_2011	unclear	low	low	low	high	high	unclear	high
	Yatawara_2016	low	low	low	low	high	low	low	moderate
	Yui_2013	unclear	low	low	low	unclear	low	low	low
	Zimmerman_2021	low	low	low	low	high	high	low	high
**adults/mixed populations**	Anagnostou_2012	low	low	unclear	unclear	low	low	low	low
	Bernaerts_2020	low	low	low	low	low	low	low	low
	Bolognani_2019	low	low	low	low	low	low	low	low
	Chez_2017	unclear	low	unclear	unclear	low	high	low	moderate
	Hollander_2012	unclear	unclear	unclear	low	low	unclear	low	moderate
	Kosaka_2016	low	low	unclear	unclear	low	low	low	low
	McDougle_1996	unclear	unclear	low	low	low	high	low	moderate
	McDougle_1998	low	unclear	low	low	low	low	low	low
	Munesue_2016	low	low	low	low	low	low	low	low
	NCT00609531	unclear	low	unclear	unclear	unclear	unclear	unclear	moderate
	NCT02909959	low	low	low	low	low	low	unclear	low
	NCT03504917	low	low	low	low	low	unclear	high	moderate
	Noone_2014	unclear	unclear	unclear	unclear	low	unclear	high	moderate

	Remington_2001	unclear	unclear	low	low	unclear	high	unclear	moderate
	Singh_2014	low	low	low	low	low	low	low	low
	Watanabe_2015	low	low	low	low	high	low	low	moderate
	Wink_2020	unclear	low	low	low	high	high	unclear	high
	Yamasue_2018	low	low	low	low	low	low	low	low

5.2.2 Risk of bias summary of included studies

5.2.2.1 Children/adolescents

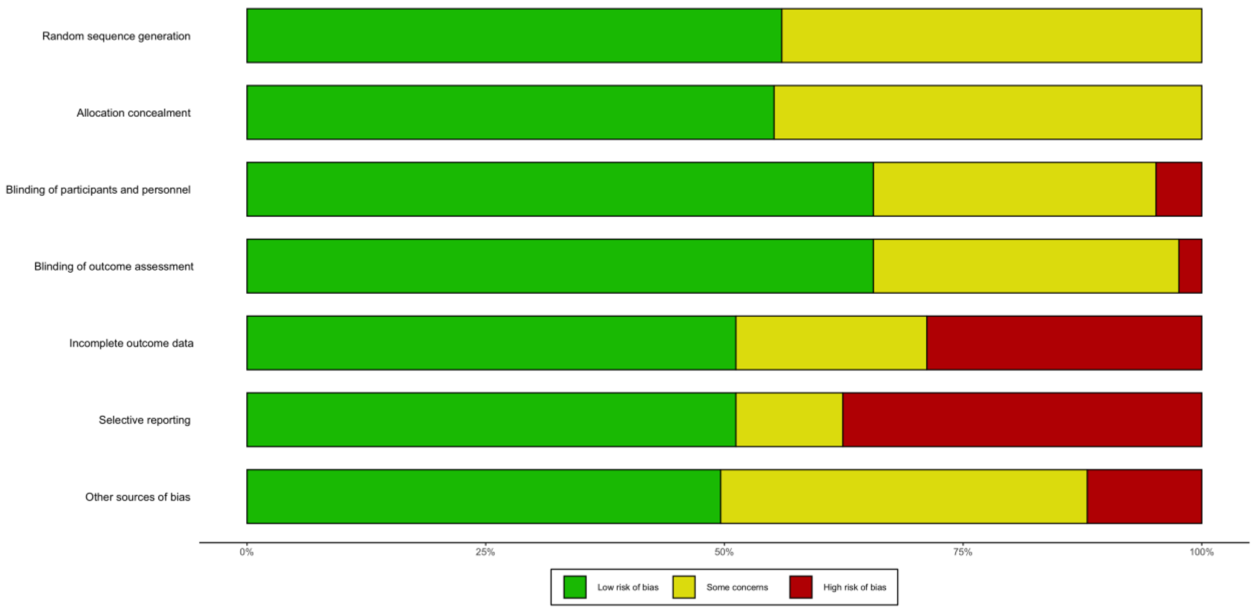

The table below presents the number and percentage of studiesin children/adolescnets (from 125 studies including in the meta-analysis of at least an outcome) with low/unclear/high risk of bias per domain.

**Domain**	**Low risk**	**Unclear risk**	**High risk**
Random sequence generation	70 (56%)	55 (44%)	0 (0%)
Allocation concealment	69 (55.2%)	56 (44.8%)	0 (0%)
Blinding of participants and personnel	82 (65.6%)	37 (29.6%)	6 (4.8%)
Blinding of outcome assessment	82 (65.6%)	40 (32%)	3 (2.4%)
Incomplete outcome data	64 (51.2%)	25 (20%)	36 (28.8%)
Selective reporting	64 (51.2%)	14 (11.2%)	47 (37.6%)
Other bias	62 (49.6%)	48 (38.4%)	15 (12%)
	**Low risk**	**Moderate risk**	**High risk**
Overall	29 (23.2%)	71 (56.8%)	25 (20%)

5.2.2.2 Adults/mixed populations

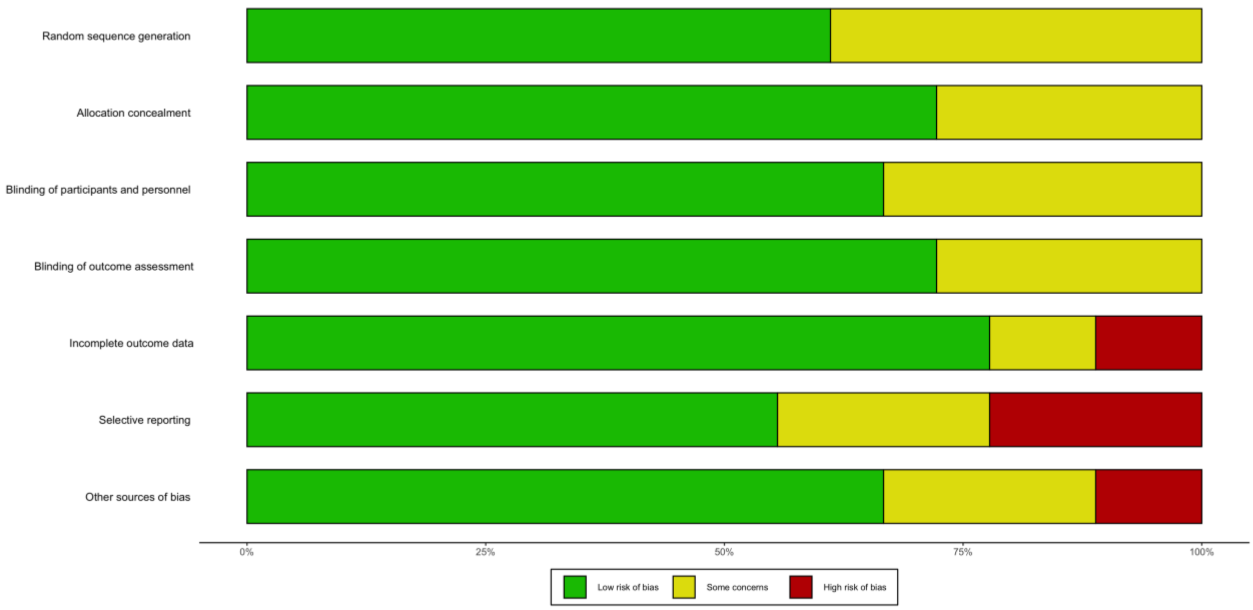

The table below presents the number and percentage of studies in adults or mixed populations (from 18 studies including in the meta-analysis of at least an outcome) with low/unclear/high risk of bias per domain.

Domain	Low risk	Unclear risk	High risk
Random sequence generation	11 (61.1%)	7 (38.9%)	0 (0%)
Allocation concealment	13 (72.2%)	5 (27.8%)	0 (0%)
Blinding of participants and personnel	12 (66.7%)	6 (33.3%)	0 (0%)
Blinding of outcome assessment	13 (72.2%)	5 (27.8%)	0 (0%)
Incomplete outcome data	14 (77.8%)	2 (11.1%)	2 (11.1%)
Selective reporting	10 (55.6%)	4 (22.2%)	4 (22.2%)
Other bias	12 (66.7%)	4 (22.2%)	2 (11.1%)
	**Low risk**	**Moderate risk**	**High risk**
Overall	9 (50%)	8 (44.4%)	1 (5.6%)

5.3. Eligible scales

5.3.1 General strategy

Data from clinicians' (observations or semi-structured interviews), caregivers' and teachers' rating scales were extracted separately for the primary outcomes and we preferred clinician's scales for the secondary outcomes. Self-reporting scales were also used when scores of other raters were not available. Usually one scale per type of informant was reported, but

- Regarding caregivers' and teachers' ratings, we preferred the frequently used scores of ABC-Lethargy/Social Withdrawal [1], ABC-Stereotypic behavior [2], SRS total score [3], ABC-Irritability [4], ABC-Hyperactivity [4], CASI-Anxiety [5]. SRS subscales were also eligible when data from other scales were not available [6, 7].
- Regarding clinicians' ratings, we preferred the frequently used Vineland-Socialization domain (semi-structured interview) [1], CYBOCS-PDD (or C-YBOCS-Compulsion subscale) [2], CARS [3], CPRS [4] and ADHD-RS (clinician-scored version) [8] as well as PARS [5].
- Self-reported scales (e.g. versions of SRS and RBS) were only rarely used in clinical trials, and they were used when scores of other raters were not available.
- No priority was given for any of the scales measuring quality of life, parental stress and global functioning, since up to one scale for each of these outcomes was usually used in the trials.

A note about SRS: The five original subscales of SRS were based on expert consensus rather appropriate factorial analyses. However, a more recent confirmatory factor analysis suggested that a two-factor structure consisting of social-communication difficulties and repetitive behaviors/restricted interests (RRBI) could be acceptable [9]. Therefore, we decided that SRS subscales would be eligible when data from other scales were not available for social-communication deficits or RRBI. We followed the two-factor structure whenever possible, and when the five original SRS subscales were reported, we used as a) measure of RRBI the subscale 'Autistic Mannerism' and as b) measure of social-communication difficulties, we calculated a total score (or average for T-standardized scores) using the four subscales Social Awareness, Social cognition, Social Communication, Social Motivation [7]. To calculate the standard deviation of a total or average score, we assumed a correlation of 0.5 between the subscales [10, 11].

5.3.2 Table of eligible scales

**Social-communication difficulties**	**Repetitive behaviors and restricted interests**	**Overall core symptoms**
Preferred:<br>• ABC-Lethargy/Social Withdrawal<br>• ADOS-Social<br>• AIM-Social reciprocity/ AIM-Peer interaction<br>• ASQ-Social<br>• ATEC-Sociability<br>• BASC-Social skills/BASC-Withdrawal<br>• BOSCC-Social communication<br>• CBCL-Social Problems<br>• GARS-Social	• ABC-Stereotypic behavior<br>• ADOS-Repetitive behaviors<br>• AIM-Repetitive behaviors<br>• ASQ-Stereotyped behavior<br>• BOSCC-Repetitive behaviors<br>• CYBOCS-PDD<br>• CYBOCS/YBOCS-Compulsion subscale (total score also eligible)	• ADOS-CSS (total score also eligible, if the calibrated severity score not available)<br>• BOSCC total score<br>• AIM-Frequency/AIM-Impact<br>• ASQ total score<br>• AUBC total score<br>• BSE-Autism factor<br>• CARS total score<br>• CBCL-PDD scale<br>• CPRS-Autism factor<br>• CBSQ<br>• GARS total score

• PDD-BI-Social approach behaviors/PDD-BI-Social Pragmatic Problems<br>• VABS-Socialization<br>Also eligible, when the former not available:<br>• ADOS-Communication<br>• AIM-Communication<br>• ASQ-Communication<br>• BASC-Functional communication<br>• GARS communication<br>• PDD-BI-Receptive/expressive social communication abilities<br>• VABS-Communication<br>• CCC-2 Social interaction deviance index<br>• SRS-Social communication composite score	• GARS-Stereotyped behavior<br>• PDD-BI-Sensory perceptual approach behaviors/PDDBI-Stereotyped restricted behavior<br>• RBQ<br>• RBS-R total score<br>• SRS-Autistic Mannerisms (when another scale was not available)	• PDD-BI-Autism composite score/ PDD-BI-Approach/Withdrawal problems<br>• RF total score<br>• SRS total score (standardized scores preferred to raw)
**Irritability**	**ADHD symptoms**	**Internalizing symptoms (anxiety and depressive symptoms)**
• ABC-Irritability<br>• CBCL-Aggression<br>• CPRS-Anger/uncooperativeness<br>• CPRS-Conduct Problems factor<br>• DBC-Disruptive/Antisocial subscale<br>• HSQ-ASD<br>• POMS-Anger<br>• SDQ-Conduct Problems subscale<br>• SIB-Q	• ABC-Hyperactivity<br>• ADHD-Rating Scale<br>• CBCL-ADHD<br>• CPRS-Hyperactivity factor<br>• DBC-ADHD<br>• SDQ-H subscale<br>• SNAP-IV-ADHD	• BASC-Internalizing<br>• CASI<br>• CBCL-Internalizing<br>• DBC-Anxiety subscale<br>• NCBRF-Insecure/anxious<br>• PARS<br>• PAS-R<br>• POMS-Depression<br>• PRAS-ASD<br>• SCAS<br>• SDQ-Emotional<br>• STAI-State anxiety<br>• VAS-Anxiety
**Quality of life**	**Global functioning**	**Parental stress**
• PedsQL-Generic Core<br>• WHO-QoL	• CGAS<br>• CGAS-DD<br>• GAF	• APSI<br>• CGSQ<br>• CSQ<br>• NOSI<br>• PedsQL-Family Impact<br>• PSI<br>• WHO-5 for well-being of caregivers

Abbreviations:
ABC, Aberrant Behavior Checklist; ADOS, Autism Diagnostic Observation Scale; AIM, Autism Impact Measure; APSI: Autism Parenting Stress Index; ASQ, Autism Symptoms Questionnaires;

ATEC, Autism Treatment Evaluation Checklist; AUBC, Krug's Autism Behavior Checklist; BASC, Behavior Assessment System for Children; BSE, Behavior Summarized Evaluation; BOSCC, Brief Observation of Social Communication Change; CARS, Childhood Autism Rating Scale; CASI, Childhood Anxiety Senstvity Index; CBCL, Child Behavior Checklist; CBSQ, Children's Social Behavior Questionnaire; CCC-2, Children Communication Checklist; CGAS(-DD), Children Global Assessment Scale (-Developmental Disorders); CGSQ, Caregiver Strain Questionnaire; CPRS, Children's Psychiatric Rating Scale; (C)YBOCS-PDD, (Children) Yale Obsessive Compulsive Scale-Pervasive Developmental Disorders; CSQ, Client Satisfaction Questionnaire; DBC, Developmental Behavior Checklist; GAF, Global Assessment of Functioning; GARS, Gilliam Autism Rating Scale; HSQ-ASD: Home Situation Questionnaire-Autism Spectrum Disorder; NCBR, Nisonger Child Behavior Rating Form; NOSI, Nijmeegse Ouderlijke Stress Index; PAS-R, Preschool Anxiety Scale-Revised; PARS, Pediatric Anxiety Rating Scale; PedsQL, Pediatric Quality of Life Invenotry; PDD-BI, Pervasive Developmental Disorders Behavioral Inventory; POMS, Profile of Mood States; PRAS-ASD, Parent-Rated Anxiety Scale for ASD; RBQ, Repetitive Behaviour Questionnaire; PSI, Parental Stress Index; RBS-R, Repetitive Behavior Scale - Revised; RF, Ritvo-Freeman Real Life Rating Scale; SCAS, Spence Children's Anxiety Scale; SDQ, Strengths and Difficulties Questionnaire; SIB-Q, Self-Injurious Questionnaire; SRS, Social Responsiveness Scale; STAI, State-Trait Anxiety Inventory; VABS, Vineland Adaptive Behavior Scale; VAS, Visual Analogue Scale, WHO-QoL, World Health Organization-Quality of Life; WHO-5, World Healt Organization-Five Well-Being Index

5.4 References

1. Anagnostou E, Jones N, Huerta M, Halladay AK, Wang P, Scahill L, et al. Measuring social communication behaviors as a treatment endpoint in individuals with autism spectrum disorder. Autism. 2014;19(5):622-36. doi: 10.1177/1362361314542955.
2. Scahill L, Aman MG, Lecavalier L, Halladay AK, Bishop SL, Bodfish JW, et al. Measuring repetitive behaviors as a treatment endpoint in youth with autism spectrum disorder. Autism. 2013;19(1):38-52. doi: 10.1177/1362361313510069.
3. European Medicines Agency. Guideline on the clinical development of medicinal products for the treatment of Autism Spectrum Disorder (ASD). In: (CHMP) CfMPfHU, editor.2017.
4. Aman MG, Novotny S, Samango-Sprouse C, Lecavalier L, Leonard E, Gadow KD, et al. Outcome measures for clinical drug trials in autism. CNS Spectr. 2004;9(1):36-47.
5. Lecavalier L, Wood JJ, Halladay AK, Jones NE, Aman MG, Cook EH, et al. Measuring anxiety as a treatment endpoint in youth with autism spectrum disorder. Journal of autism and developmental disorders. 2014;44(5):1128-43. doi: 10.1007/s10803-013-1974-9.
6. Constantino JN, Gruber CP. Social Responsiveness Scale: SRS-2 Software Kit. Western Psychological Services; 2012.
7. Cheon KA, Park JI, Koh YJ, Song J, Hong HJ, Kim YK, et al. The social responsiveness scale in relation to DSM IV and DSM5 ASD in Korean children. Autism Res. 2016;9(9):970-80. doi: 10.1002/aur.1671.
8. Cortese S, Adamo N, Del Giovane C, Mohr-Jensen C, Hayes AJ, Carucci S, et al. Comparative efficacy and tolerability of medications for attention-deficit hyperactivity disorder in children, adolescents, and adults: a systematic review and network meta-analysis. The Lancet Psychiatry. 2018;5(9):727-38. doi: 10.1016/S2215-0366(18)30269-4.
9. Frazier TW, Ratliff KR, Gruber C, Zhang Y, Law PA, Constantino JN. Confirmatory factor analytic structure and measurement invariance of quantitative autistic traits measured by the Social Responsiveness Scale-2. Autism. 2013;18(1):31-44. doi: 10.1177/1362361313500382.
10. Borenstein M, Hedges LV, Higgins JPT, Rothstein HR. Introduction to meta-analysis. John Wiley & Sons; 2011.
11. Balk EM, Earley A, Patel K, Trikalinos TA, Dahabreh IJ. Empirical assessment of within-arm correlation imputation in trials of continuous outcomes. 2012.

eAppendix-6 Results

6.1 Assessment of transitivity and baseline imbalance 182
6.1.1 Assessment of transitivity assumption 182
6.1.1.1 Age (years) 183
6.1.1.2 Trial duration (weeks) 184
6.1.1.3 Baseline ABC-Irritability 185
6.1.1.4 Baseline CGI-Severity 186
6.1.1.5 Type of rater 187
Rater of scales measuring social-communication difficulties 187
Rater of scales measuring repetitive behaviors and restricted interests 188
Rater of scales measuring overall core symptoms 189
6.1.1.6 Presence of associated symptoms as inclusion criteria 190
6.1.2 Assessment of baseline imbalance 191
6.1.2.1 Social-communication difficulties 192
6.1.2.2 Repetitive behaviors and restricted interests 193
6.1.2.3 Scales measuring overall core symptoms 194
6.1.2.4 Irritability 195
6.1.2.5 ADHD symptoms 196
6.1.2.6 Anxiety and depressive symptoms 197
6.1.2.7 Quality of life 198
6.1.2.8 Global functioning 199
6.1.2.9 Parental stress 200
6.2 Network plots 201
6.3 Forest plots of placebo-controlled comparisons for secondary outcomes 202
6.4 League tables 203
6.5. Assessment of heterogeneity and incoherence 204
6.5.1 Table of heterogeneity and incoherence tests 204
6.5.1.1 Children/adolescents 204
6.5.1.2 Adult and mixed populations 205
6.5.2 Forest plots of SIDE 207
6.5.2.1 Social-communication difficulties 207
6.5.2.2 Repetitive behaviors and restricted interests 208
6.5.2.3 Scales measuring overall core symptoms 209
6.5.2.4 Irritability 210
6.5.2.5 ADHD symptoms 211

6.5.2.6 Anxiety and depressive symptoms 211
6.5.2.7 Response to treatment......212
6.5.2.8 Global functioning 213
6.5.2.9 Quality of life......213
6.5.2.10 Parental stress 213
6.5.2.11 Discontinuation due to any reason 213
6.5.2.12 Discontinuation due to adverse event 214
6.5.2.13 Any adverse event 214
6.5.2.14 Weight gain 215
6.5.2.15 Sedation 216
6.5.2.16 Extrapyramidal symptoms......216
6.5.3. References......216
6.6. Sensitivity analysis 217
Social-communication difficulties......217
Children/adolescents......217
Adults......221
Repetitive behaviors 222
Children/adolescents......222
Adults......226
Overall core symptoms 227
Children/adolescents......227
Adults......233
Dichotomous outcomes 233
6.7. Effect sizes of individual studies 234
6.8. Assessment of publication bias and small-study effects 235
6.8.1 Social-communication difficulties 235
6.8.2 Repetitive behaviors and restricted interests 236
Funnel plots for children/adolescents: 236
Funnel plots for adults or mixed populations: 236
6.8.3 Scales measuring overall core symptoms......237
Funnel plots for children/adolescents: 237
6.8.4 Available data across studies and comparisons for the primary outcomes 238
6.8.4.1 Summary table of data per outcome and comparison 238
6.9. Grading the confidence in evidence with CINeMA 252
6.9.1 General 252

6.9.1.1 Domains of CINeMA 252
i. Within-study bias..252
ii. Reporting bias..252
iii. Indirectness..252
iv. Imprecision..252
v. Heterogeneity..253
vi. Incoherence..253
6.9.1.2 Summarizing judgments across the domains..254
6.9.2 CINeMA for each primary outcome..254
6.9.2.1 Social-communication difficulties..255
6.9.2.1.1 Children/adolescents..255
a. Network plot..255
b. Confidence in evidence..255
6.9.2.1.2 Adults or mixed..257
a. Network plot..257
b. Confidence in evidence..257
6.9.2.2 Repetitive behaviors and restricted interests..257
6.9.2.2.1 Children/adolescents..258
a. Network plot..258
b. Confidence in evidence..258
6.9.2.2.2 Adults or mixed..259
a. Network plot..259
b. Confidence in evidence..260
6.9.2.3 Overall core symptoms..260
6.9.2.3.1 Children/adolescents..260
a. Network plot..260
b. Confidence in evidence..260
6.9.2.3.2 Adults or mixed..262
a. Network plot..262
b. Confidence in evidence..262
6.9.3 References..262

6.1 Assessment of transitivity and baseline imbalance

6.1.1 Assessment of transitivity assumption

We assessed transitivity assumption by examining the distribution of potential effect-modifiers (age, trial duration, baseline ABC-irritability, baseline CGI-Severity, type of rater for the primary outcomes and the presence of associated symptoms as inclusion criteria) across treatments, since most of the comparisons were placebo-controlled. Summary statistics (as median, interquartile ranges IQR) are also presented.

6.1.1.1 Age (years)

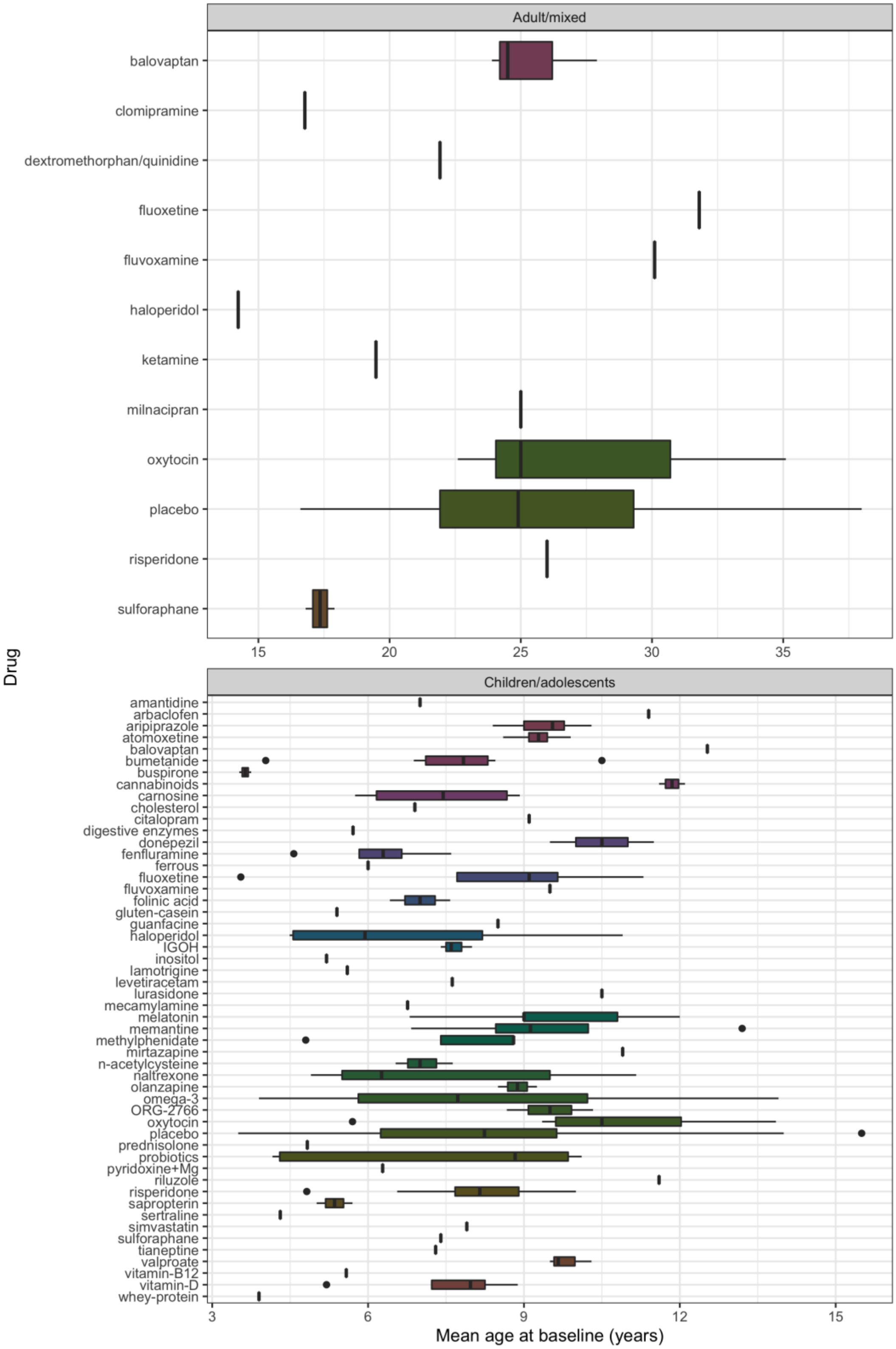

The median [IQR] of mean age was 8.23 [6.26, 9.5] in children/adolescents studies (missing in 5 arms) and 24.6 [21.92, 27.91] in adult/mixed studies (missing in 1 arms).

6.1.1.2 Trial duration (weeks)

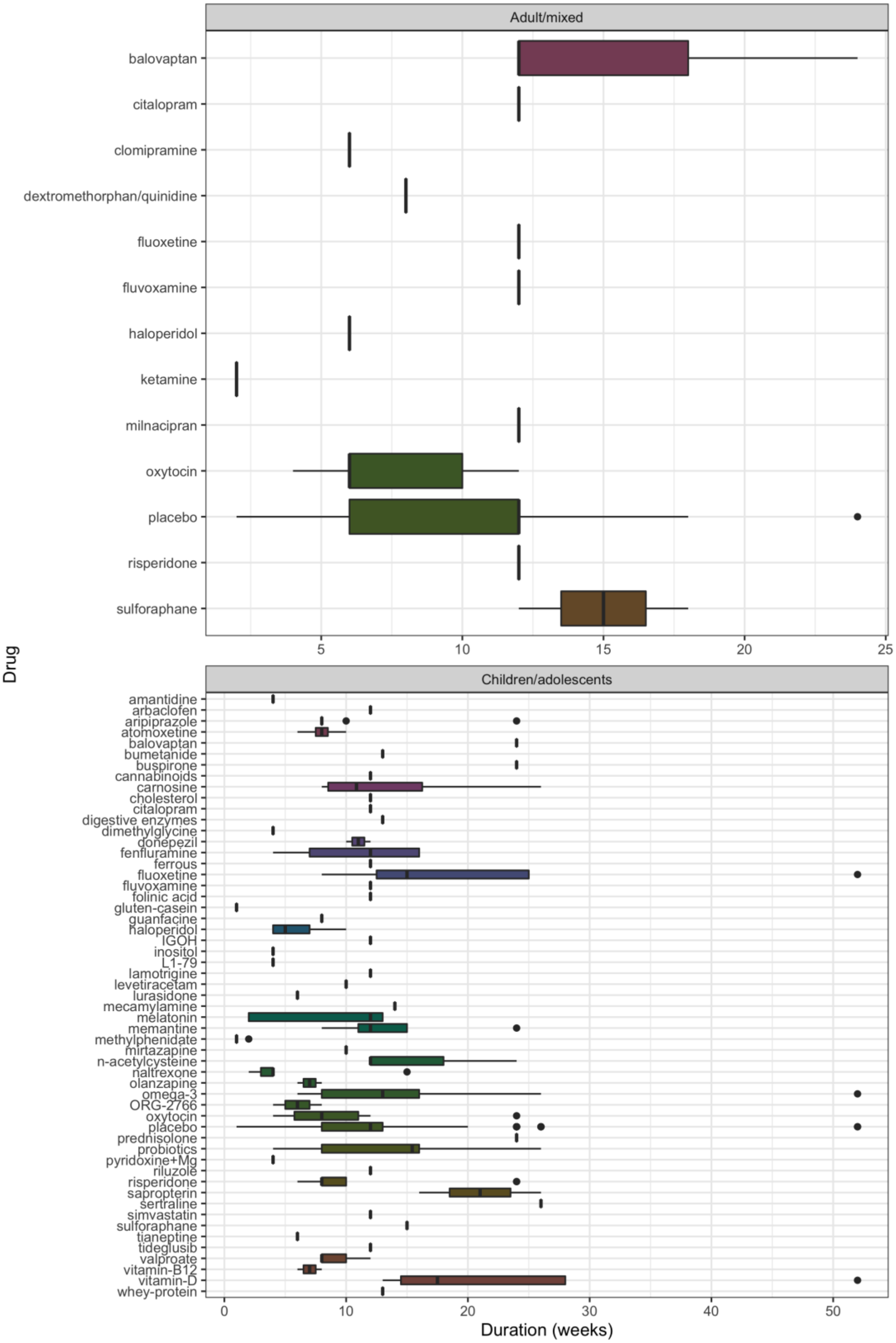

The median [IQR] of duration was 12 [8, 13] weeks in children/adolescents studies and 12 [6, 12] weeks in adult/mixed studies.

6.1.1.3 Baseline ABC-Irritability

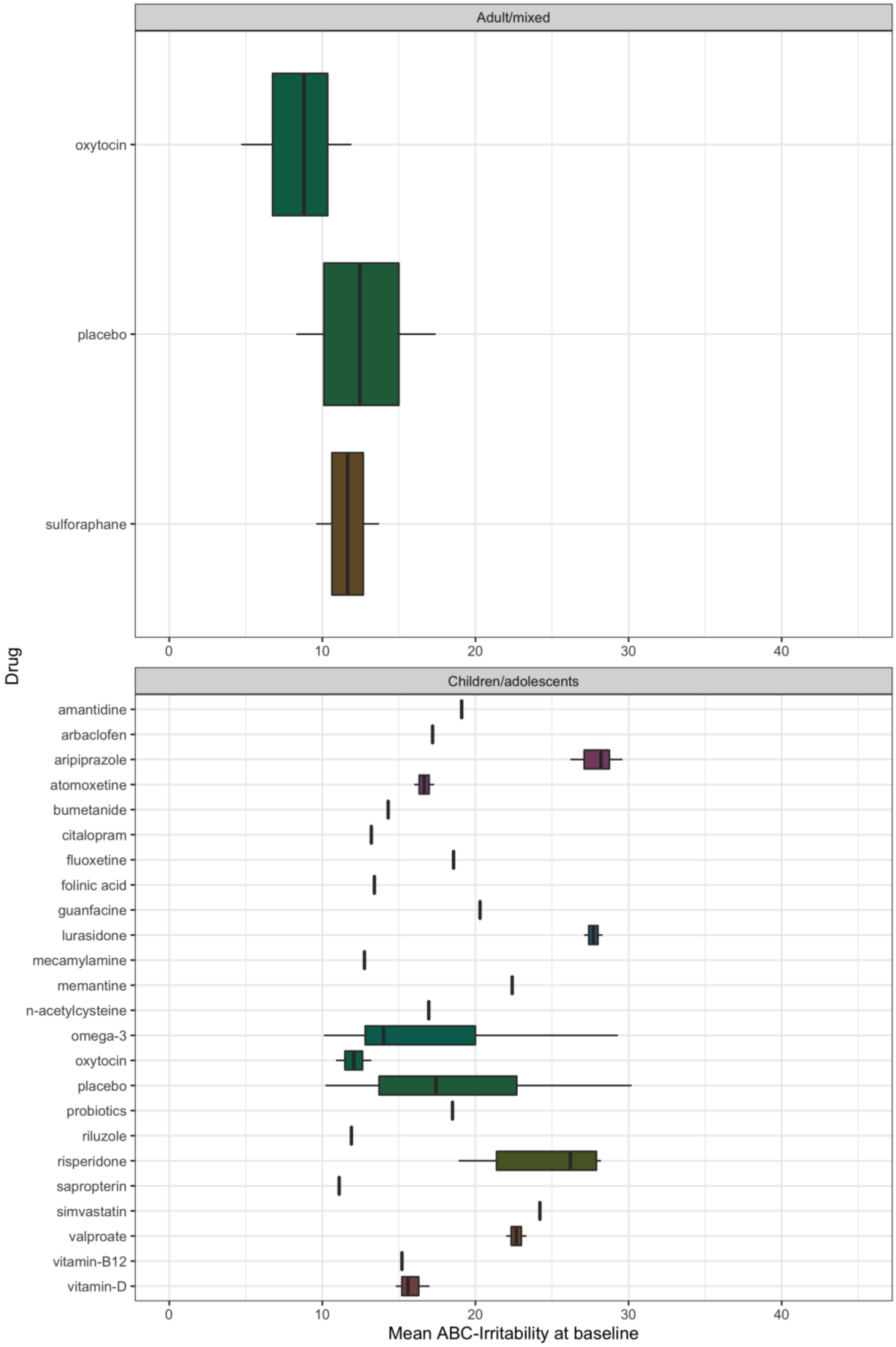

The median [IQR] of baseline ABC-Irritability was 18.23 [13.99, 23.24] in children/adolescents studies (missing in 86 arms) and 11.41 [8.73, 14.04] in adult/mixed studies (missing in 14 arms).

6.1.1.4 Baseline CGI-Severity

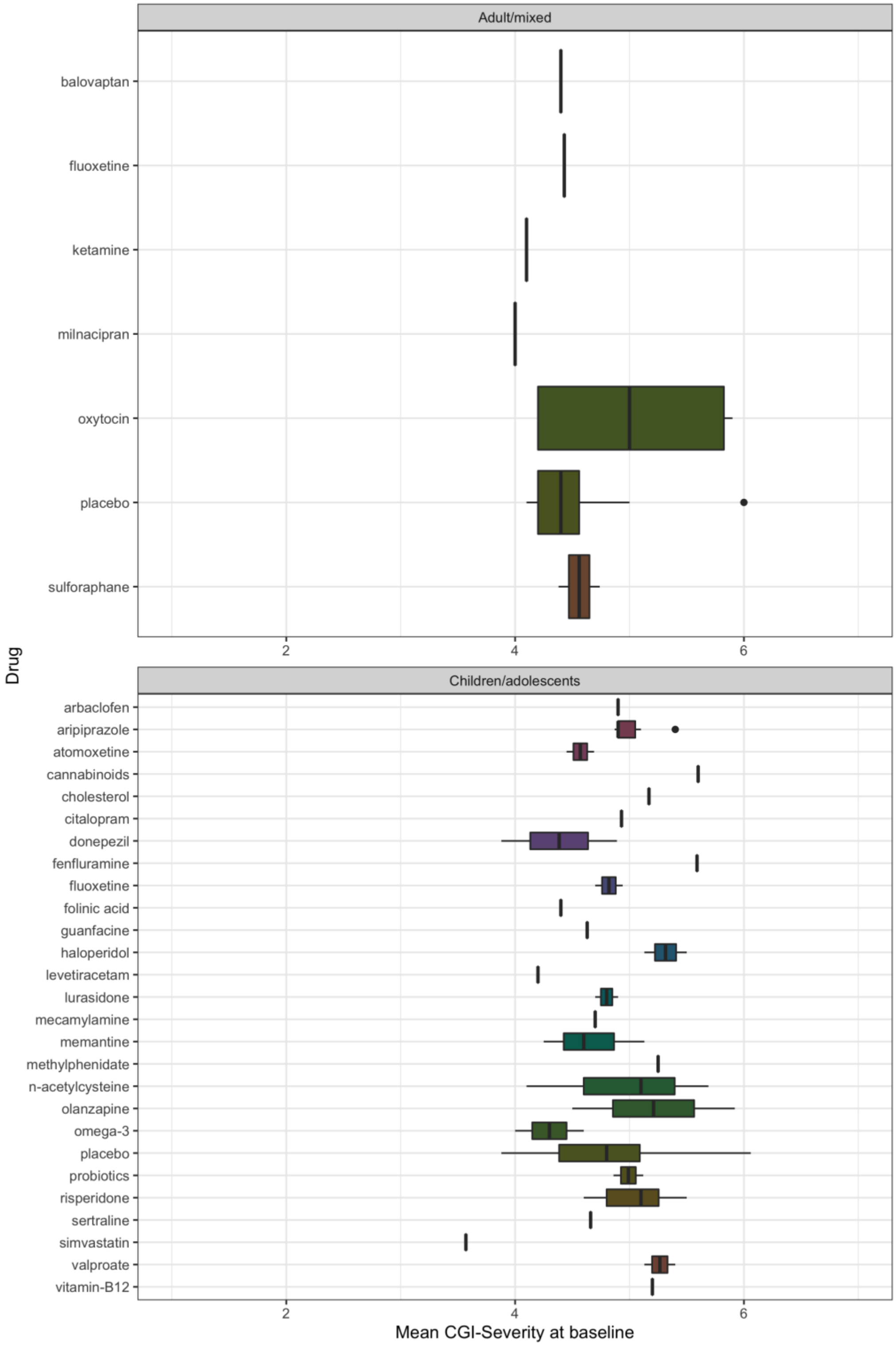

The median [IQR] of baseline CGI-S was 4.87 [4.52, 5.13] in children/adolescents studies (missing in 82 arms) and 4.4 [4.2, 4.5] in adult/mixed studies (missing in 9 arms).

6.1.1.5 Type of rater

Rater of scales measuring social-communication difficulties

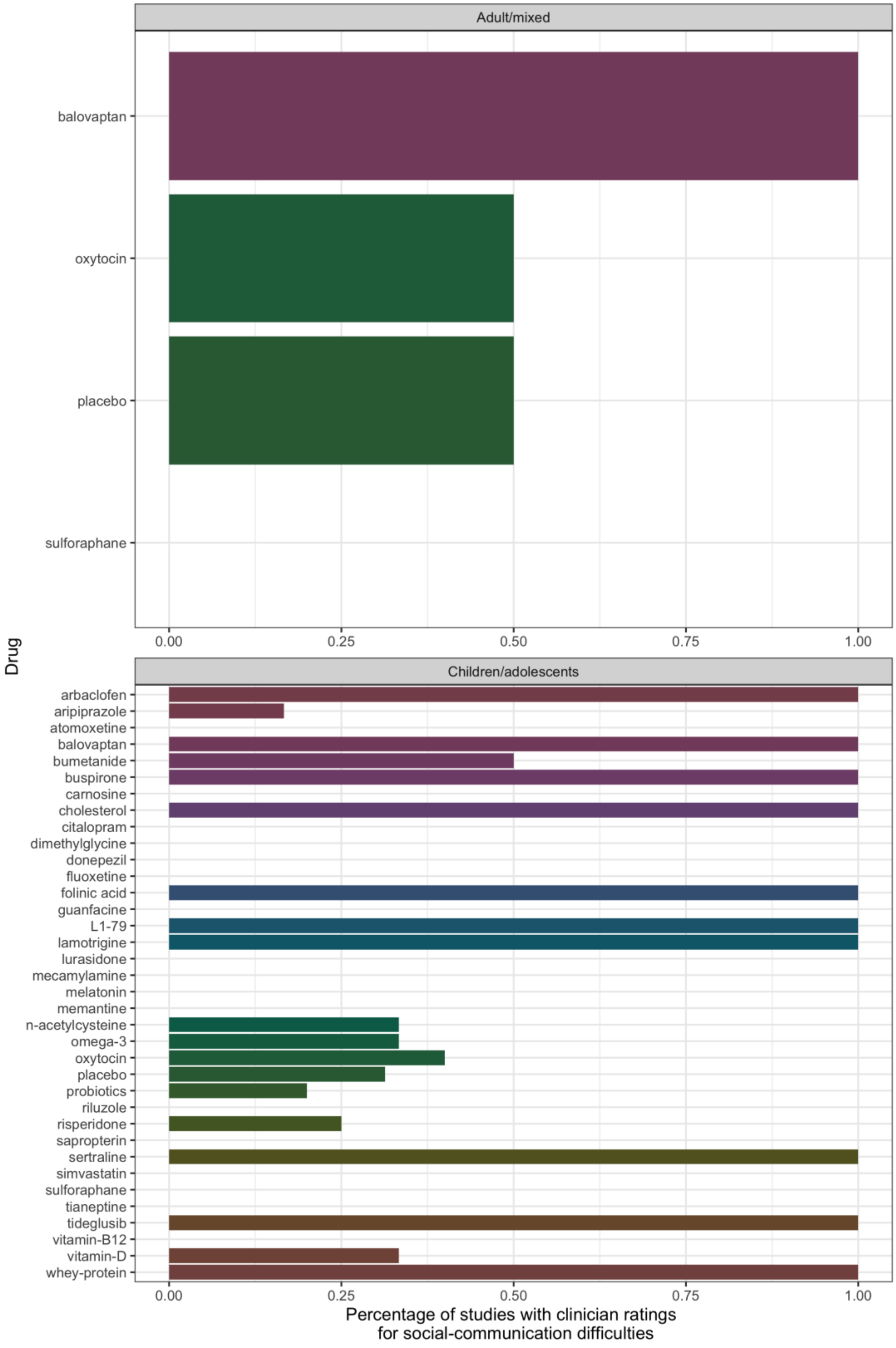

Rater of scales measuring repetitive behaviors and restricted interests

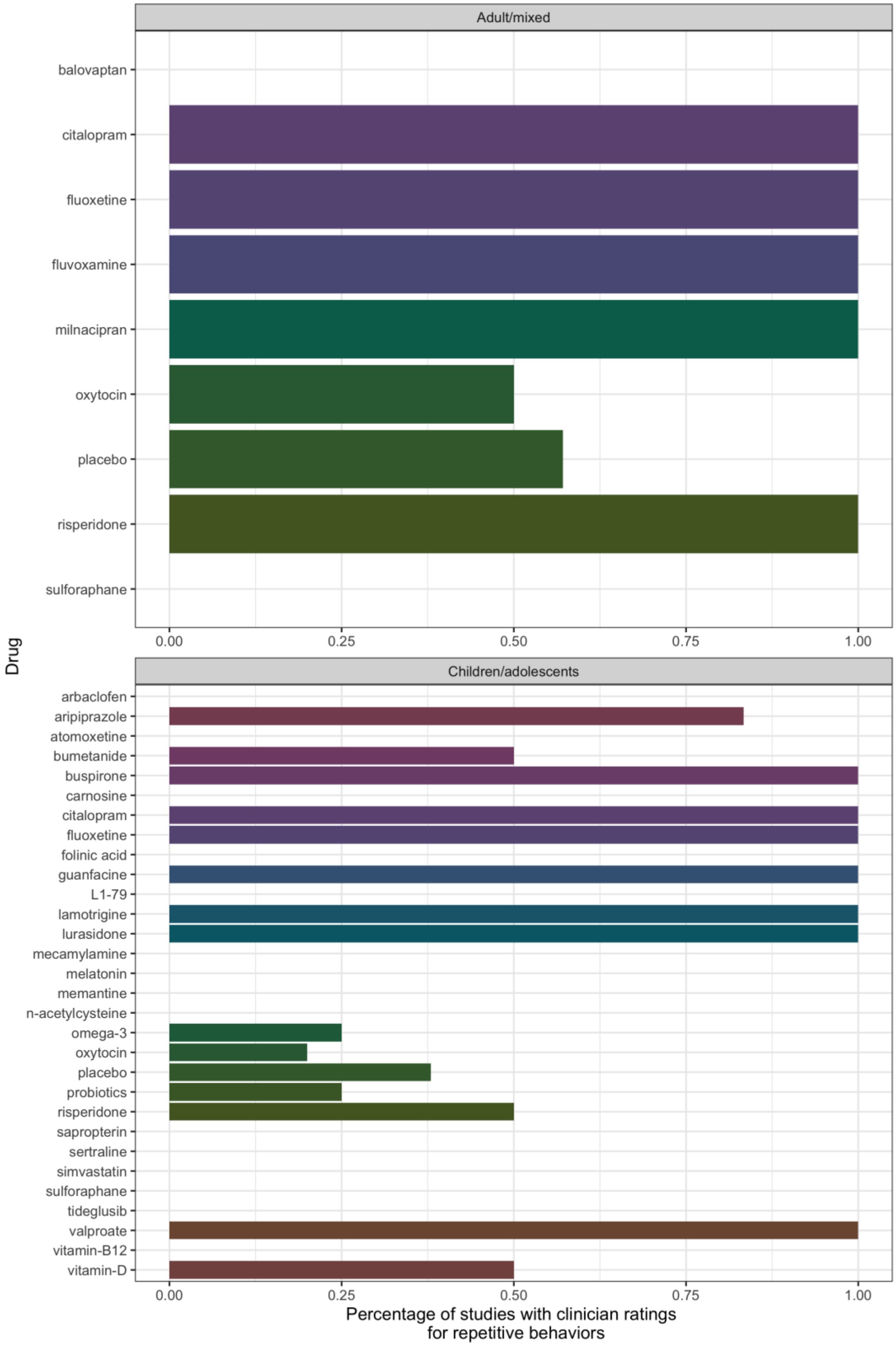

Rater of scales measuring overall core symptoms

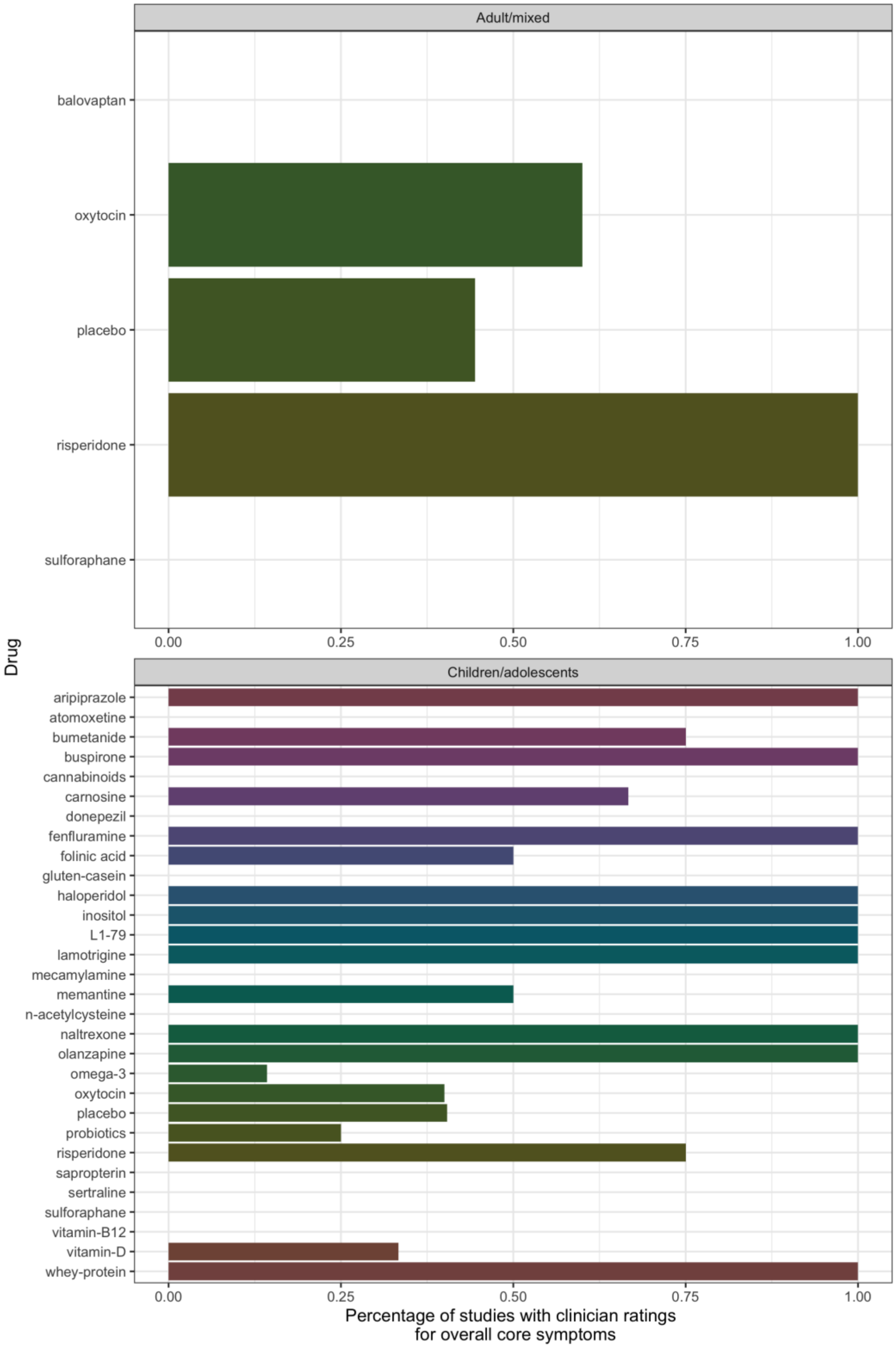

6.1.1.6 Presence of associated symptoms as inclusion criteria

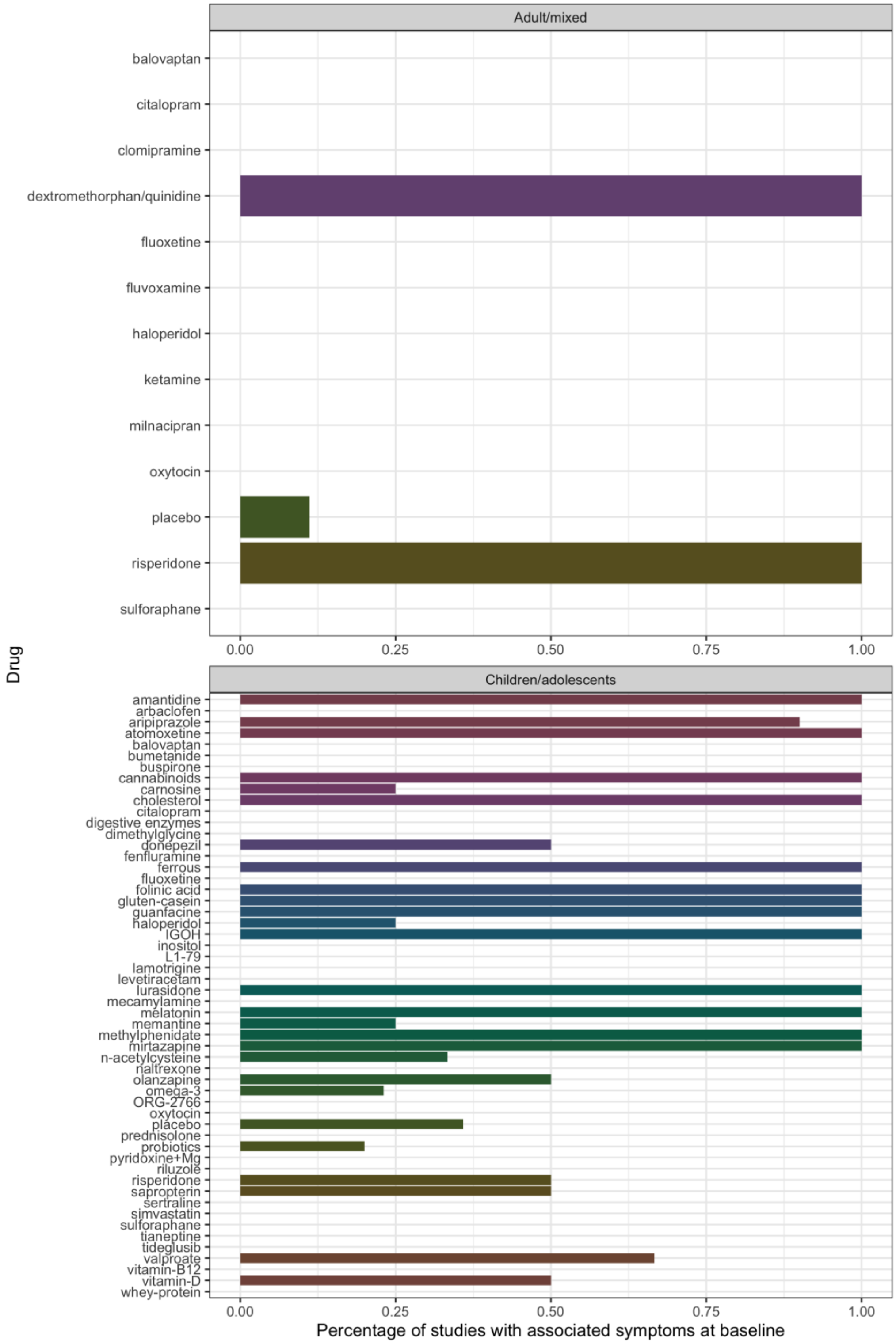

Some drugs were evaluated almost only in participants with associated conditions, 15 in children/adolescents-amantadine, atomoxetine, cannabinoids, cholesterol, ferrous sulfate, folinic acid gluten-casein, guanfacine, IGOH, lurasidone, melatonin, methylphenidate, mirtazapine and aripiprazole (in nine out of 10 studies)-and 2 in adults/mixed-dextromethorphan/quinidine and risperidone.

6.1.2 Assessment of baseline imbalance

We found that baseline score imbalance could have inflated effect sizes, when endpoint scores were used in the analysis. For example, Klaiman 2013 found no difference between saproptertin and placebo in the ABC-L/SW in their primary analysis using a mixed-effects regression models [1]. Baseline and endpoint scores of ABC-L/SW were reported, and there was important baseline imbalance (mean baseline score of saproptertin: 9.5 vs placebo: 16.2). When endpoint scores were used in the meta-analysis, sapropterin was found superior to placebo in improving ABC-L/SW with an SMD of 1.34 (95% CI 0.7; 1.99). In contrast when we estimated change scores (from endpoint and baseline means and standard deviations using a pre-post correlation of 0.5), the results were not significant SMD of 0.21 (95% CI -0.37; 0.79), similar to the primary analysis of the trial.

We further investigated this issue by plotting baseline and endpoint mean scores (when both were reported but not change scores) with their 95% confidence intervals (calculated as mean±1.96*(SD/sqrt(N)).

Baseline imbalance can be detected when the slope between interventions is not zero at baseline (slope of red dashed line) and it could have affected effect sizes when the slope at endpoint (slope of blue dashed line) is the same to baseline, but there is no difference in change scores between interventions.

Therefore, we estimated change scores when endpoint and baseline scores were reported.

1. Klaiman C, Huffman L, Masaki L, Elliott GR. Tetrahydrobiopterin as a treatment for autism spectrum disorders: a double-blind, placebo-controlled trial. J Child Adolesc Psychopharmacol. 2013;23(5):320-8. doi: 10.1089/cap.2012.0127.

6.1.2.1 Social-communication difficulties

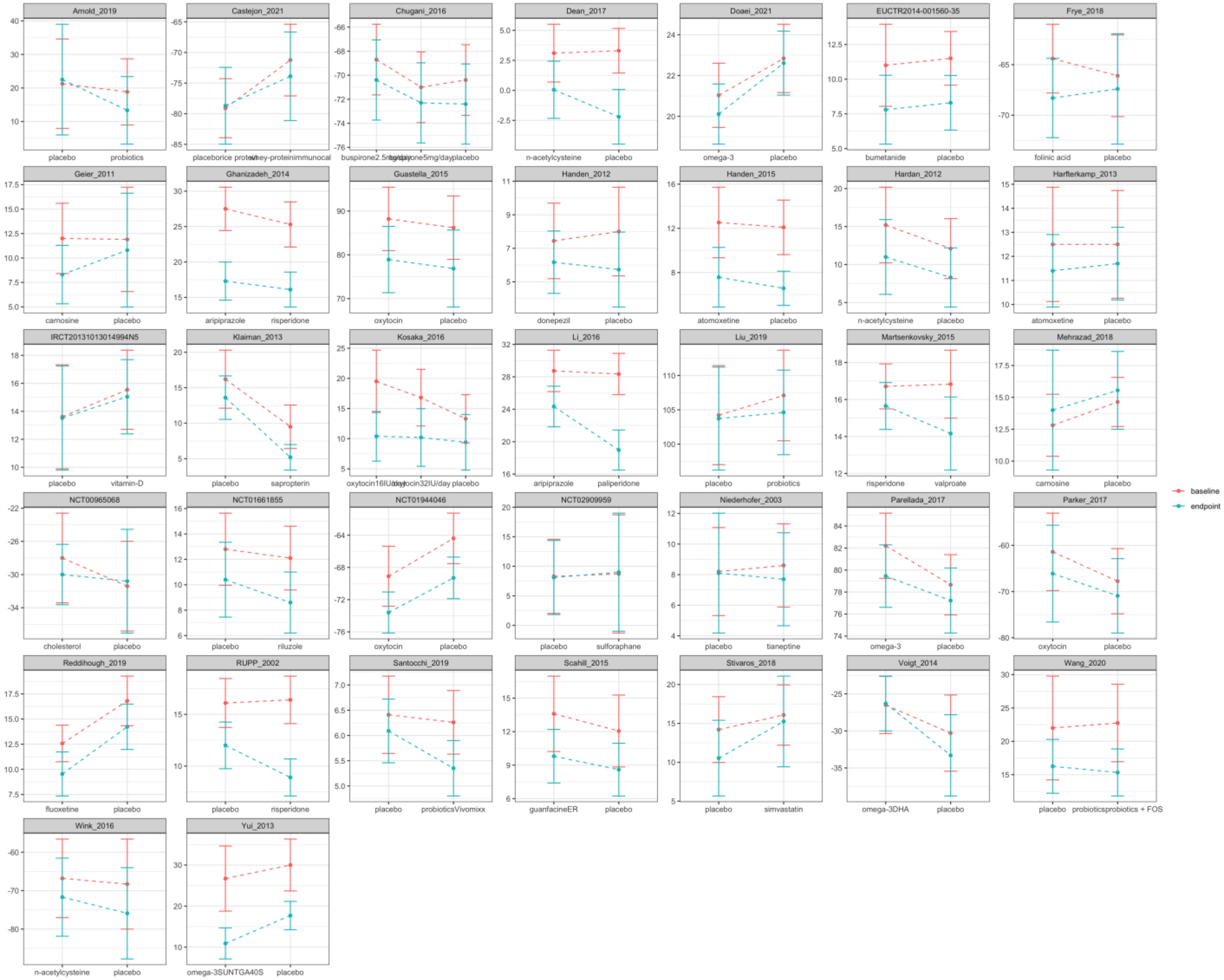

6.1.2.2 Repetitive behaviors and restricted interests

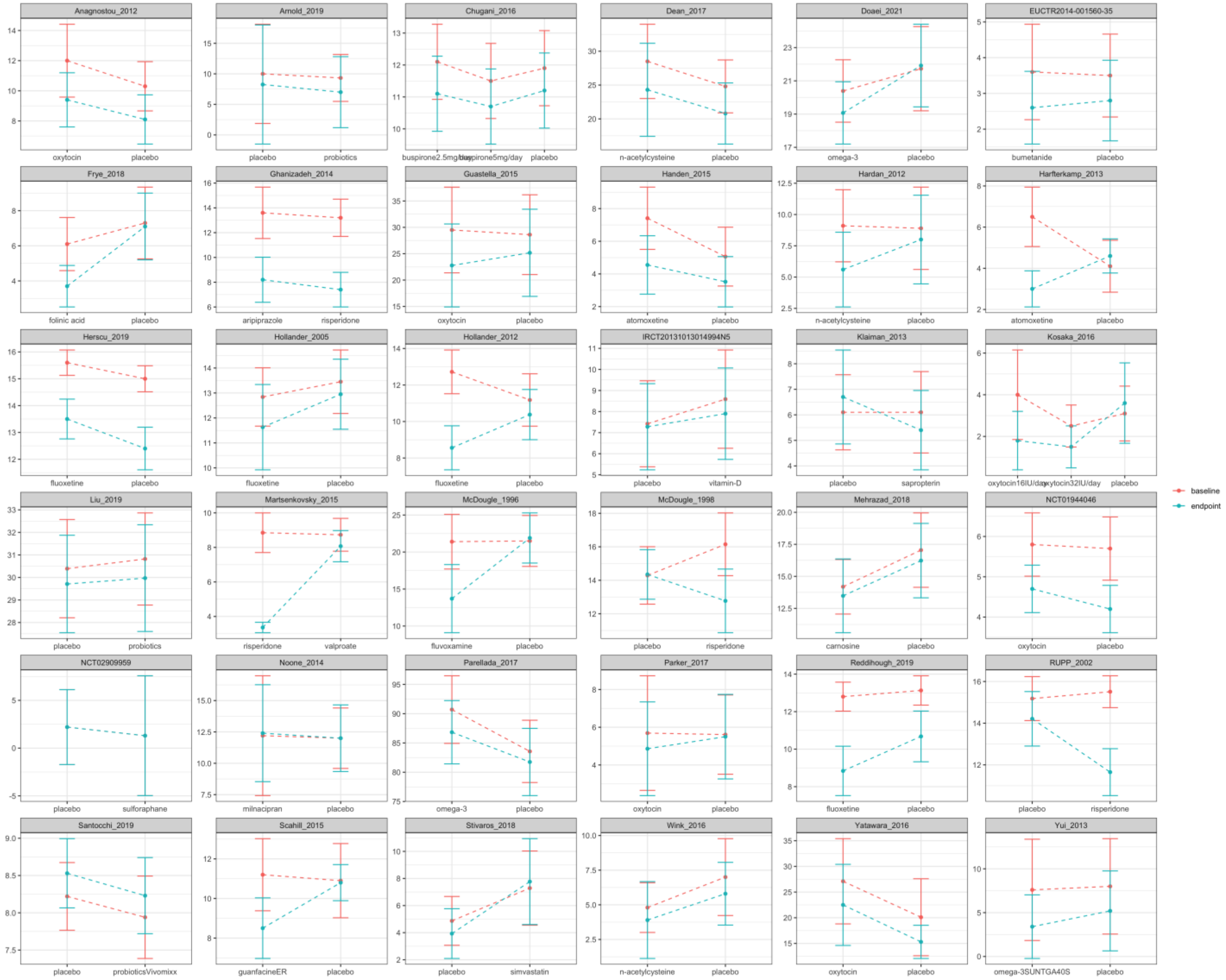

6.1.2.3 Scales measuring overall core symptoms

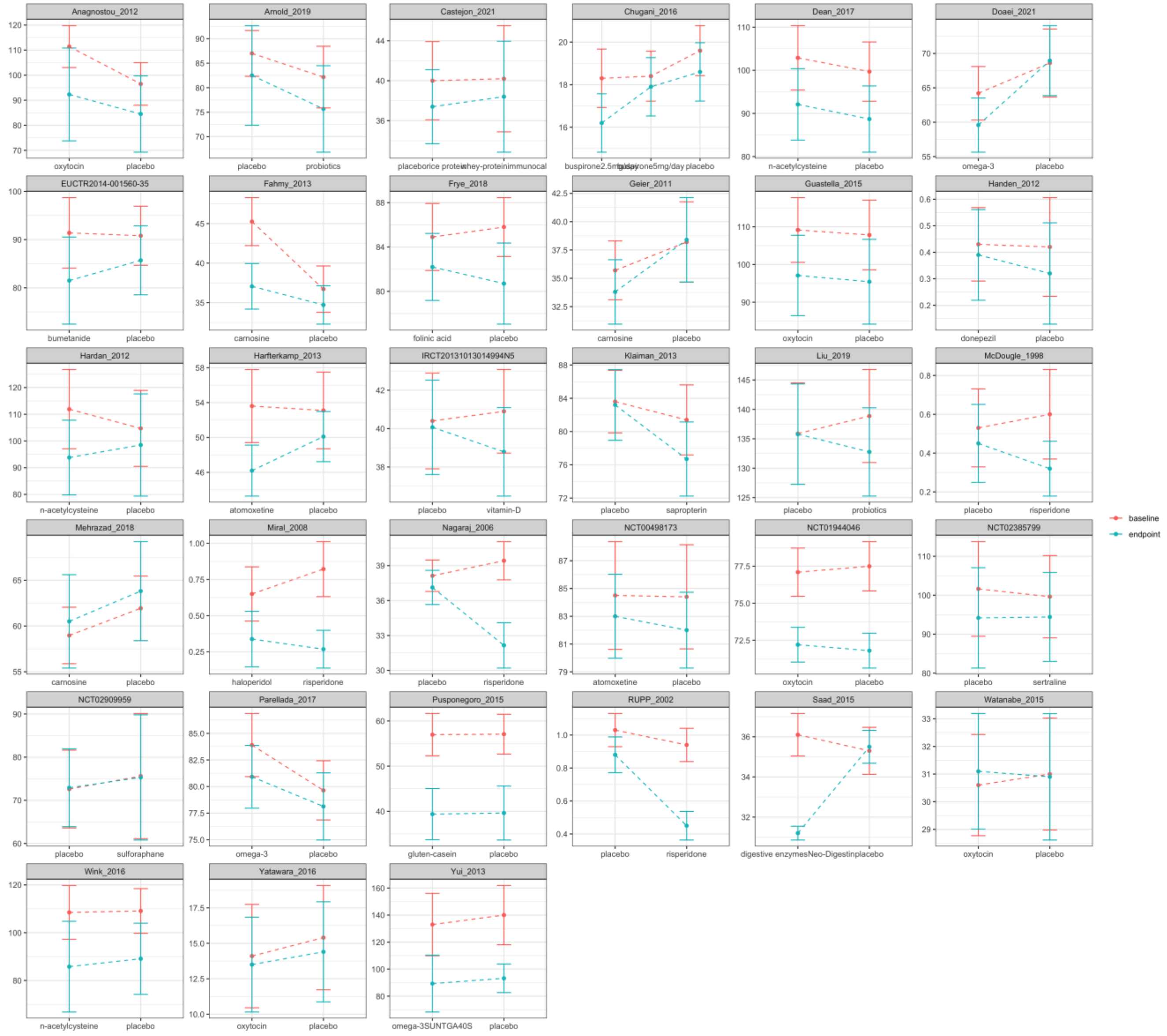

6.1.2.4 Irritability

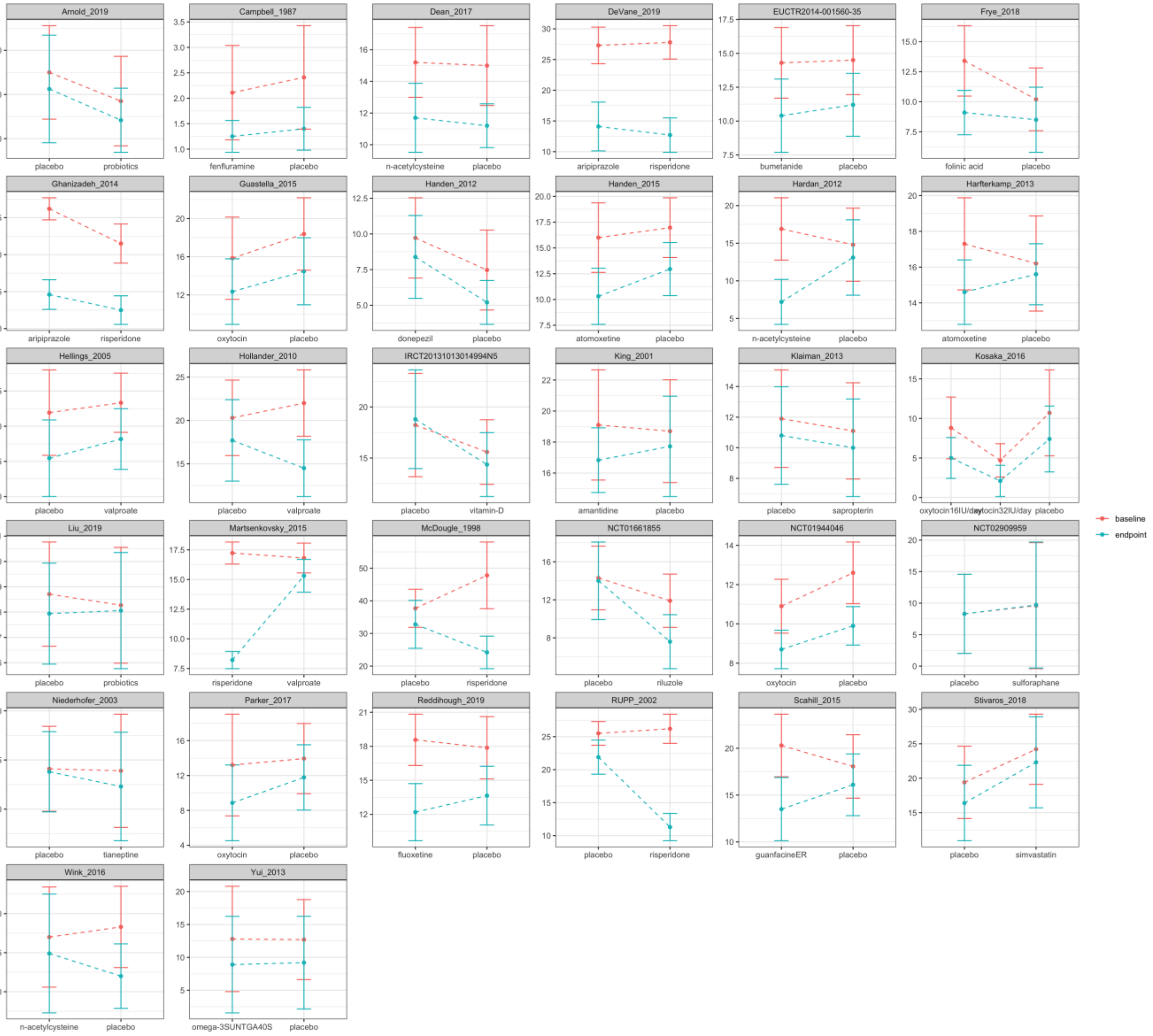

6.1.2.5 ADHD symptoms

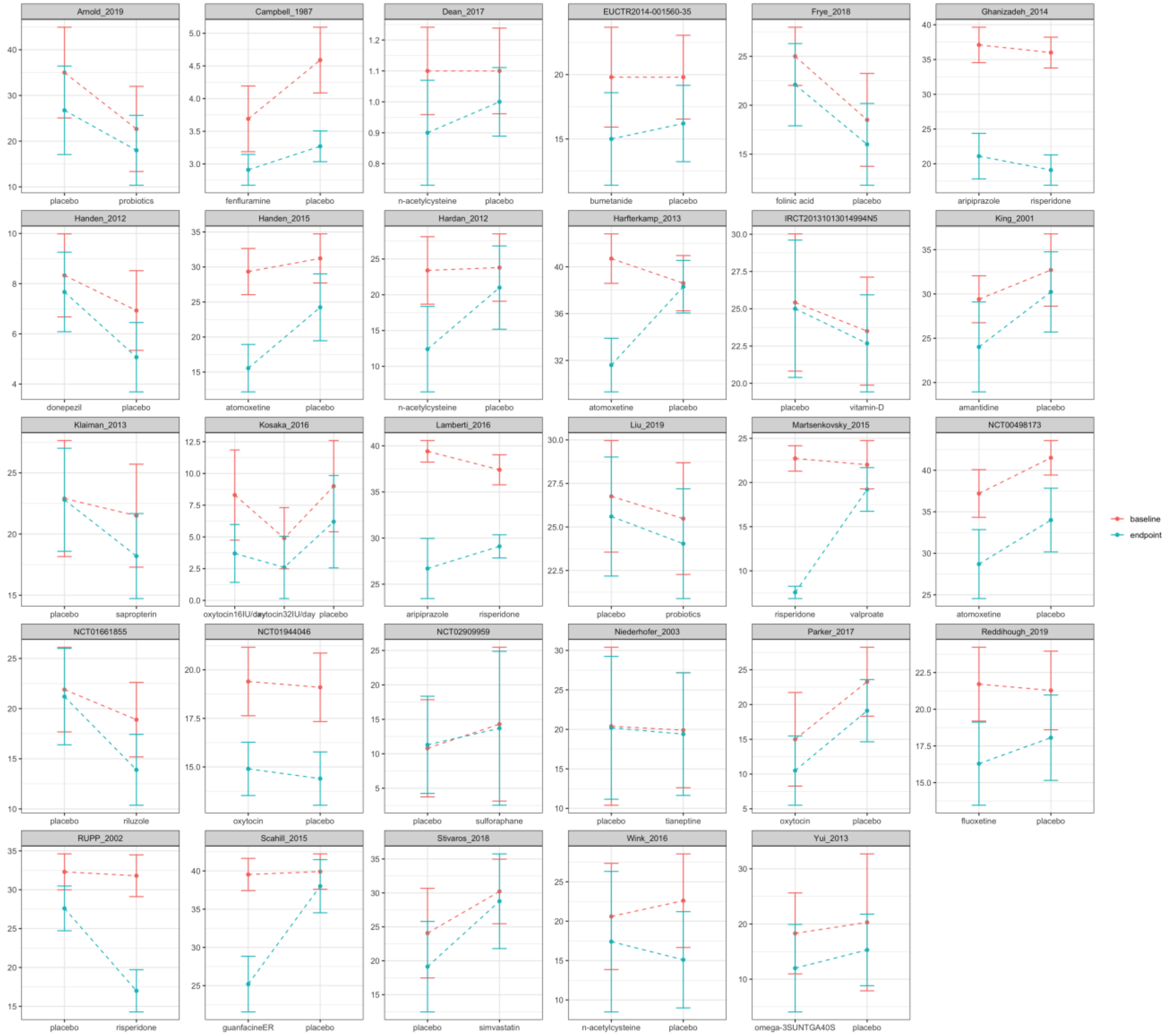

6.1.2.6 Anxiety and depressive symptoms

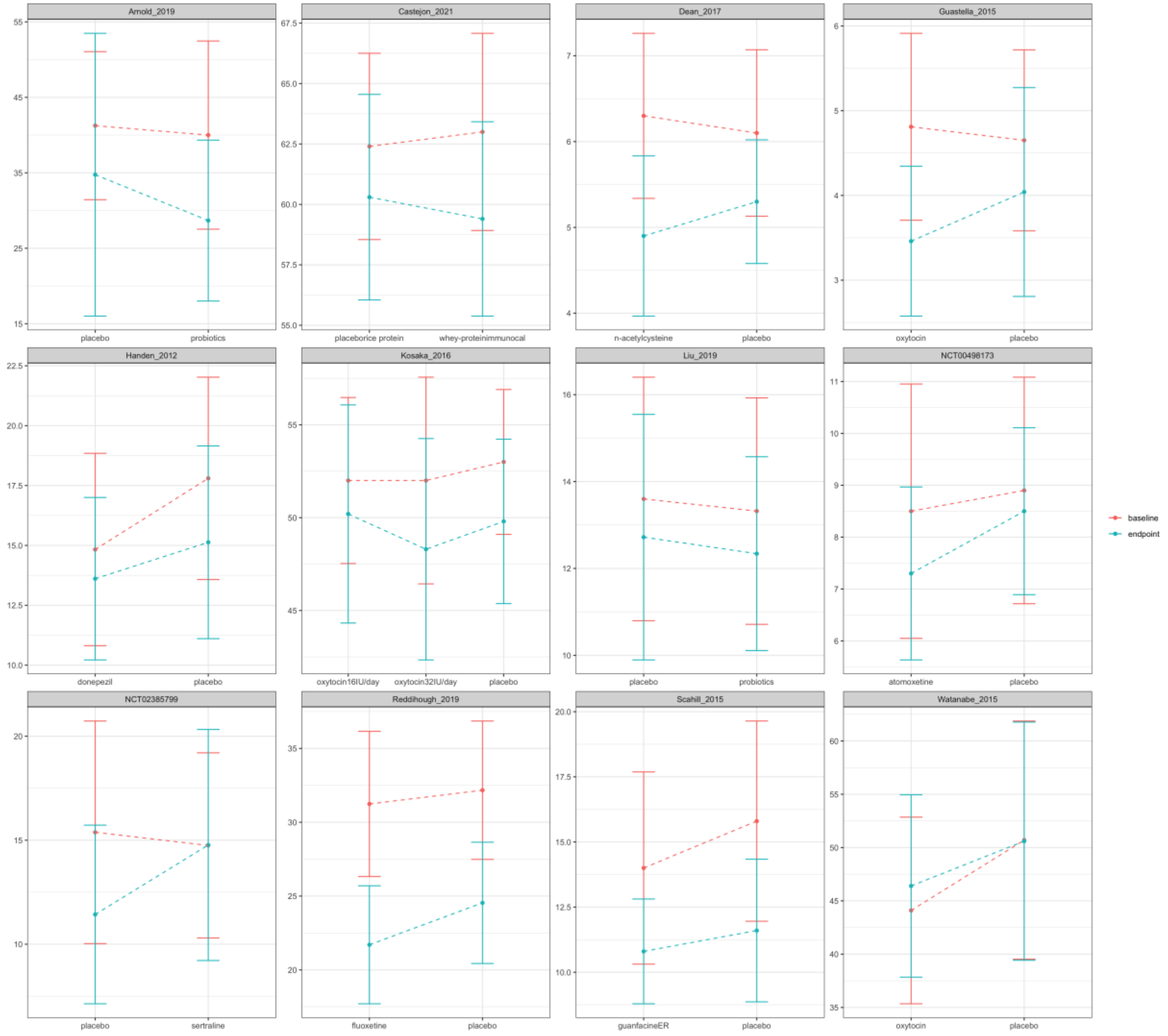

6.1.2.7 Quality of life

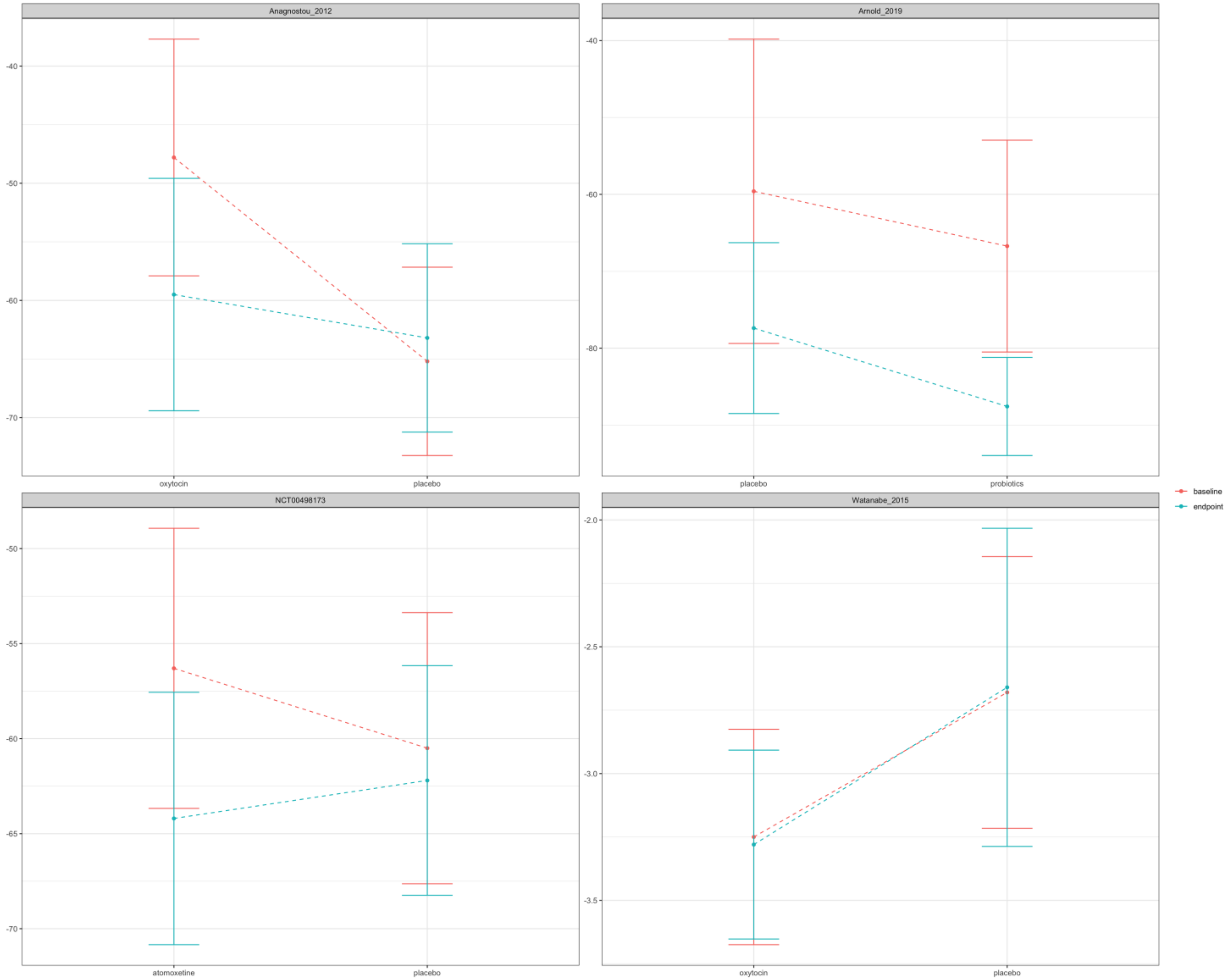

6.1.2.8 Global functioning

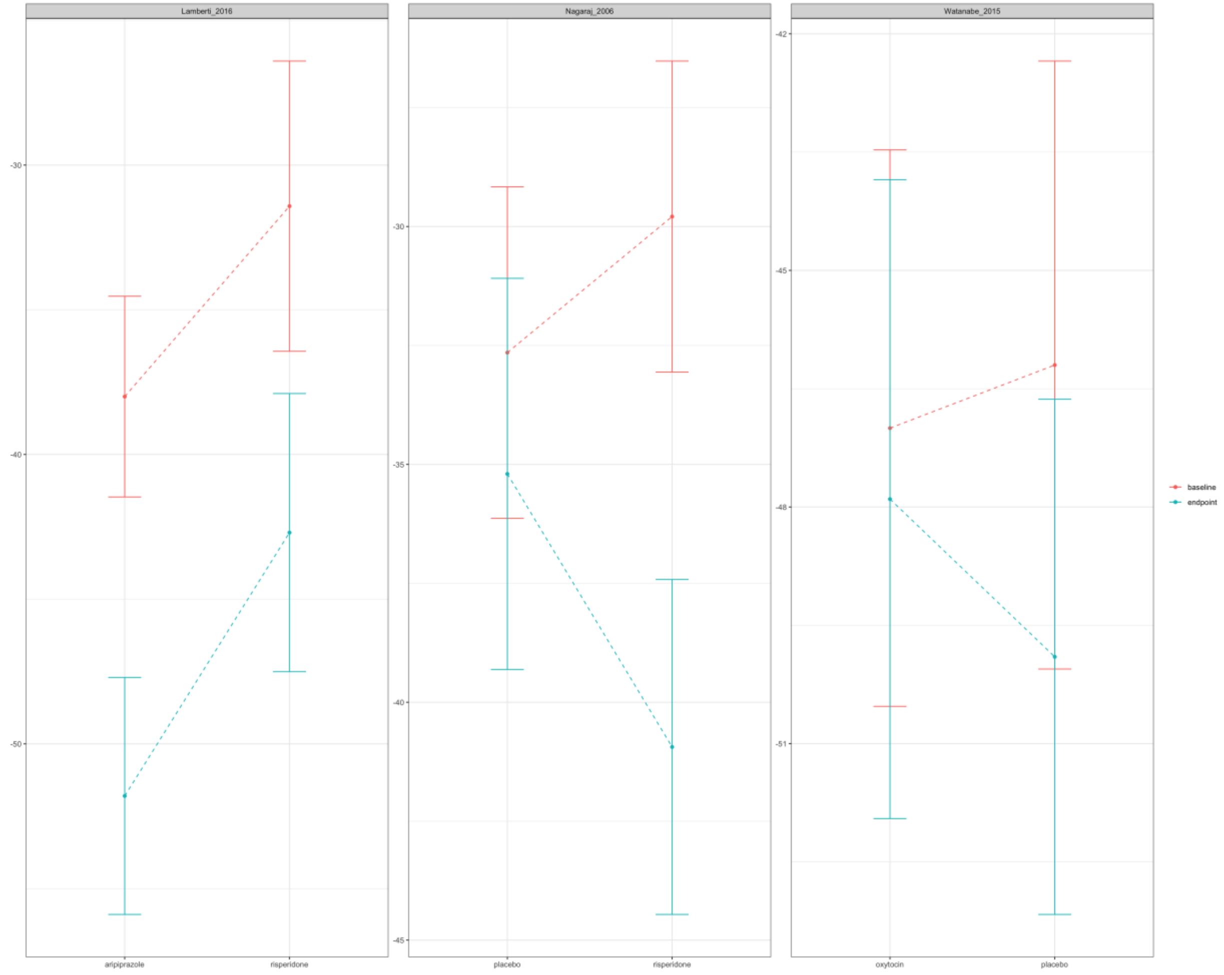

6.1.2.9 Parental stress

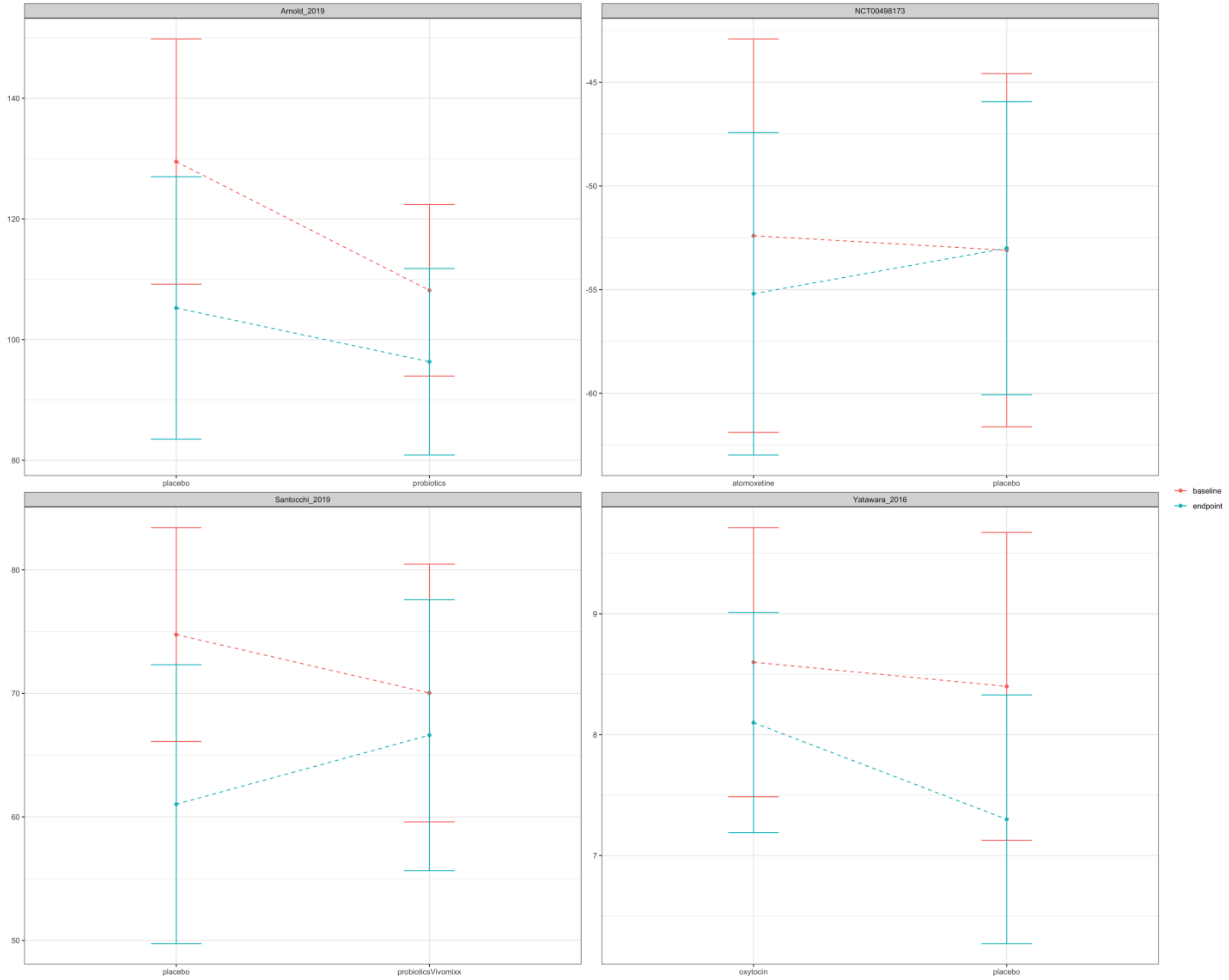

6.2 Network plots

Network plots for all outcomes are presented in Figure-S1. They were mainly star-shaped with placebo as the main node, and a few head-to-head comparisons between other interventions.

6.3 Forest plots of placebo-controlled comparisons for secondary outcomes

Effect sizes of the comparisons between interventions and placebo for the secondary outcomes are presented in Figure-S2.

Interventions are sorted by their P-scores from the best to worst (when a network meta-analysis was conducted) or by their effect sizes from best to worst (when a pairwise meta-analysis was conducted).

6.4 League tables

League tables for each outcome can be found in Table-S1, when a network meta-analysis was conducted. In the lower left corner, effect-sizes based on indirect or mixed evidence are presented. In the upper right corner, effect-sizes based on direct evidence are presented.

Head-to-head comparisons of direct comparisons can also be found in pairwise meta-analysis in Figure-S3.

6.5. Assessment of heterogeneity and incoherence

6.5.1 Table of heterogeneity and incoherence tests

We assessed heterogeneity by comparing the τ^2 of the networks with the empirical distribution of τ^2 of SMDs and ORs. Specifically, the empirical distribution of τ^2 of SMDs for the comparison of pharmacological agents versus placebo on mental health outcomes (such as our outcomes of interest) has a median of 0.049 IQR [0.01, 0.242]. The log-normal empirical distribution of τ^2 of ORs for the comparison of pharmacological agents versus placebo on subjective outcomes has a mean of -2.13 and SD of 1.58 [1], which could correspond to a median of 0.12 IQR [0.04, 0.35]. We considered low heterogeneity when $\tau^2<Q_1$, moderate when $Q_1<\tau^2<Q_2$ and high when $\tau^2>Q_2$. Incoherence was evaluated with a local approach (SIDE approach) and a global approach (design-by-treatment test). We considered the presence of potential evidence of incoherence when the design-by-treatment test was significant (alpha at 0.1) or when there were more than 10% of incoherent loops (alpha at 0.1), since up to 10% of the loops in a network meta-analysis have been found to be incoherent [2].

6.5.1.1 Children/adolescents

**Outcomes (effect size)**	**Heterogeneity in network meta-analysis**		**Incoherence in network meta-analysis**		
	**Between study variance (τ^2) and I^2**	**Heterogeneity assessment**	**Percenta0ge of loops showing inconsistency using SIDDE**	**Design-by-treatment test Q (df), p-value**	**Incoherence assessment**
Social-communication difficulties (SMD)	τ^2=0, I^2=0%	Low	0% out of 8 loops	Q=4.747 (df=4), p-value=0.314	No evidence for incoherence
Repetitive behaviors and restricted interests (SMD)	τ^2=0.017, I^2=20%	Low to moderate	0% out of 6 loops	Q=1.329 (df=3), p-value=0.722	No evidence for incoherence
Scales measuring overall core symptoms (SMD)*	τ^2=0.0382, I^2=30%	Moderate	0% out of 6 loops	Q=2.52 (df=3), p-value=0.472	No evidence for incoherence
Irritability (SMD)	*τ^2=0.0394, I^2=33.7%*	*Moderate*	*0% out of 6 loops*	*Q=10.673 (df=3), p-value=0.014*	*There was evidence of incoherence, and pairwise meta-analysis was conducted.*
ADHD symptoms (SMD)	τ^2=0.032, I^2=30.4%	Moderate	0% out of 6 loops	Q=5.124 (df=3), p-value=0.163	No evidence for incoherence
Anxiety and depressive symptoms (SMD)	τ^2=0.041, I^2=25.4%	Moderate to high	No loops	Cannot be evaluated	Cannot be evaluated
Response to treatment (OR)	*τ^2=0.011, I^2=2.6%*	*Low*	*50% out of 10 loops*	*Q=8.732 (df=4), p-value=0.068*	*There was evidence of incoherence, and pairwise meta-analysis was conducted.*
Global functioning (SMD)	τ^2=0.0169, I^2=15.2%	Low to moderate	0% out of 3 loops	Q=2.357 (df=1), p-value=0.125	No evidence for incoherence
Quality of life (SMD)	τ^2= not applicable,	Not applicable	No loops	Cannot be evaluated	Cannot be evaluated

	I^2= not applicable				
Parental stress (SMD)	τ^2=0.0, I^2=0%	Low	No loops	Cannot be evaluated	Cannot be evaluated
Discontinuation due to any reason (OR)	τ^2=0.0061, I^2=1.6%	Low	12.5% out of 8 loops	Q=4.571 (df=4), p-value=0.334	One closed loop was incoherent (omega-3, vitamin-D, placebo). Network meta-analysis was conducted, since up to 10% of the loops are expected to be incoherent.
Discontinuation due to adverse event (OR)	τ^2=0, I^2=0%	Low	0% out of 3 loops	Q=0.232 (df=1), p-value=0.63	No evidence for incoherence
Any adverse event (OR)	τ^2=0., I^2=0%	Low	0% out of 3 loops	Q=0.11 (df=1), p-value=0.74	No evidence for incoherence
Weight gain (OR)	*τ^2=0, I^2=0%*	*Low*	*50% out of 6 loops*	*Q=6.896 (df=2), p-value=0.032*	*There was evidence of incoherence, and pairwise meta-analysis was conducted.*
Sedation (OR)	*τ^2=0.168, I^2=19.3%*	*High*	*75% out of 8 loops*	*Q=7.761 (df=3), p-value=0.051*	*There was evidence of incoherence, and pairwise meta-analysis was conducted.*
Extrapyramidal symptoms (OR)	Not applicable	Not applicable	Cannot be evaluated	Cannot be evaluated	Cannot be evaluated

Italics when network meta-analysis was not conducted. *For overall core symptoms, a small study (Nikvarz et al. 2017) that compared risperidone with memantine (no difference was found SMD=0.0 [-0.71, 0.72]), was excluded from the primary analysis of this outcome, since introduced incoherence and heterogeneity (τ^2=0.054, 25% out of 8 loops were incoherence, and the design-by-treatment test was marginal Q=7.151, df=4, p-value=0.128). The results did not materially change after the inclusion of this study (Figure-S4)

6.5.1.2 Adult and mixed populations

**Outcomes**	**Heterogeneity in network meta-analysis**		**Incoherence in network meta-analysis**		
	**Between study variance (τ^2)**	**Heterogeneity assessment**	**Percentage of loops showing inconsistency using SIDDE**	**Design-by-treatment test Q (df,p)**	**Incoherence assessment**
Social-communication difficulties (SMD)	τ^2=0.096, I^2=63 %	High	No loops	Cannot be evaluated	Cannot be evaluated
Repetitive behaviors and restricted interests (SMD)	τ^2=0, I^2=0%	Low	No loops	Cannot be evaluated	Cannot be evaluated
Scales measuring overall core symptoms (SMD)	τ^2=0, I^2=0%	Low	No loops	Cannot be evaluated	Cannot be evaluated
Irritability (SMD)	τ^2=0.0281, I^2=21.6%	Moderate	No loops	Cannot be evaluated	Cannot be evaluated
ADHD symptoms (SMD)	τ^2=0, I^2=0%	Low	No loops	Cannot be evaluated	Cannot be evaluated
Anxiety and depressive symptoms (SMD)	τ^2=0, I^2=0%	Low	No loops	Cannot be evaluated	Cannot be evaluated
Response to treatment (OR)	τ^2=0.257, I^2=42.1%	High	No loops	Cannot be evaluated	Cannot be evaluated

Global functioning (SMD)	τ^2=0, I^2=0%	Low	No loops	Cannot be evaluated	Cannot be evaluated
Quality of life (SMD)	τ^2=0, I^2=0%	Low	No loops	Cannot be evaluated	Cannot be evaluated
Parental stress (SMD)	*Not applicable*	*Not applicable*	*No loops*	*Cannot be evaluated*	*Cannot be evaluated*
Discontinuation due to any reason (OR)	τ^2=0, I^2=0%	Low	No loops	Cannot be evaluated	Cannot be evaluated
Discontinuation due to adverse event (OR)	τ^2=0, I^2=0%	Low	No loops	Cannot be evaluated	Cannot be evaluated
Any adverse event (OR)	τ^2=0.049, I^2=20.8%	Low to moderate	No loops	Cannot be evaluated	Cannot be evaluated
Weight gain (OR)	τ^2=0, I^2=0%	Low	No loops	Cannot be evaluated	Cannot be evaluated
Sedation (OR)	τ^2=0, I^2=0%	Low	No loops	Cannot be evaluated	Cannot be evaluated
Extrapyramidal symptoms (OR)	Not applicable	Not applicable	Cannot be evaluated	Cannot be evaluated	Cannot be evaluated

6.5.2 Forest plots of SIDE

Local incoherence using SIDE could not be tested in studies with no closed loops, i.e., quality of life, caregiver stress, anxiety or depressive symptoms in children/adolescents and all networks in adults.

6.5.2.1 Social-communication difficulties

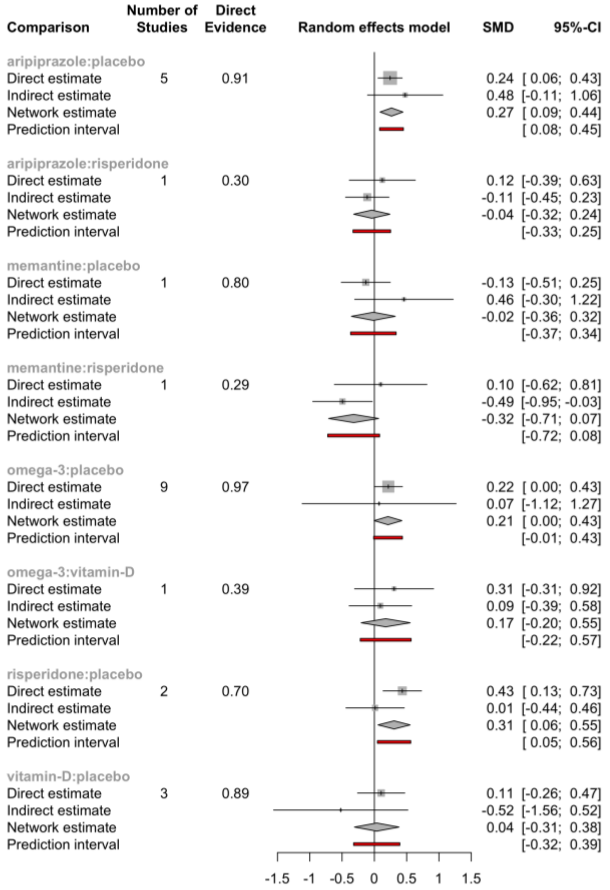

0% of the loops were incoherent.

6.5.2.2 Repetitive behaviors and restricted interests

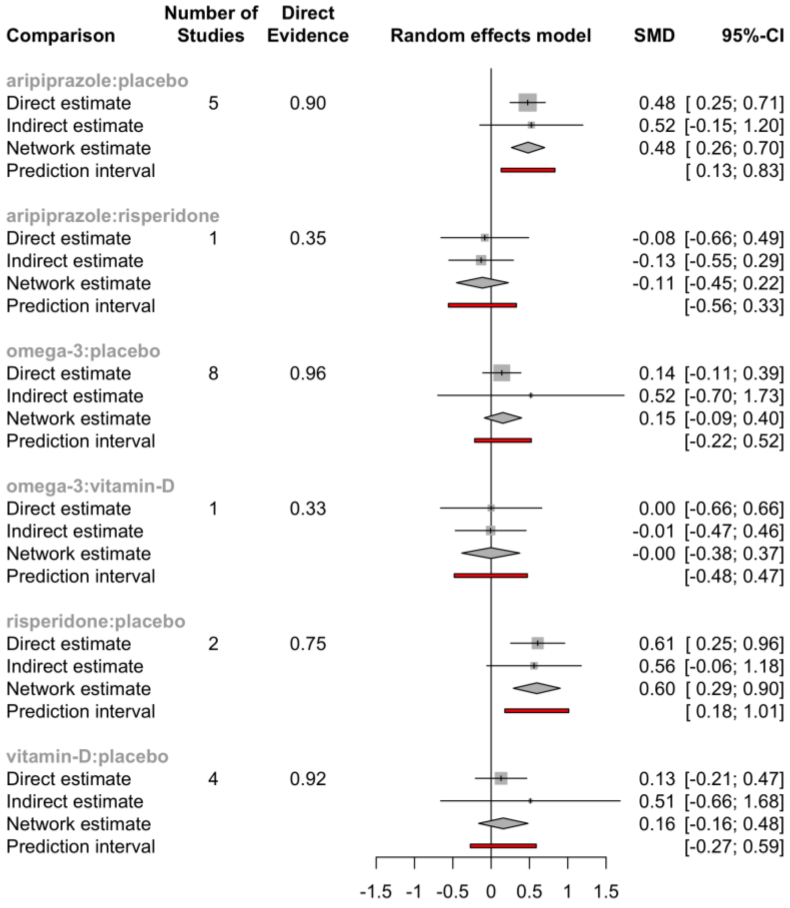

0% of the loops were incoherent.

6.5.2.3 Scales measuring overall core symptoms

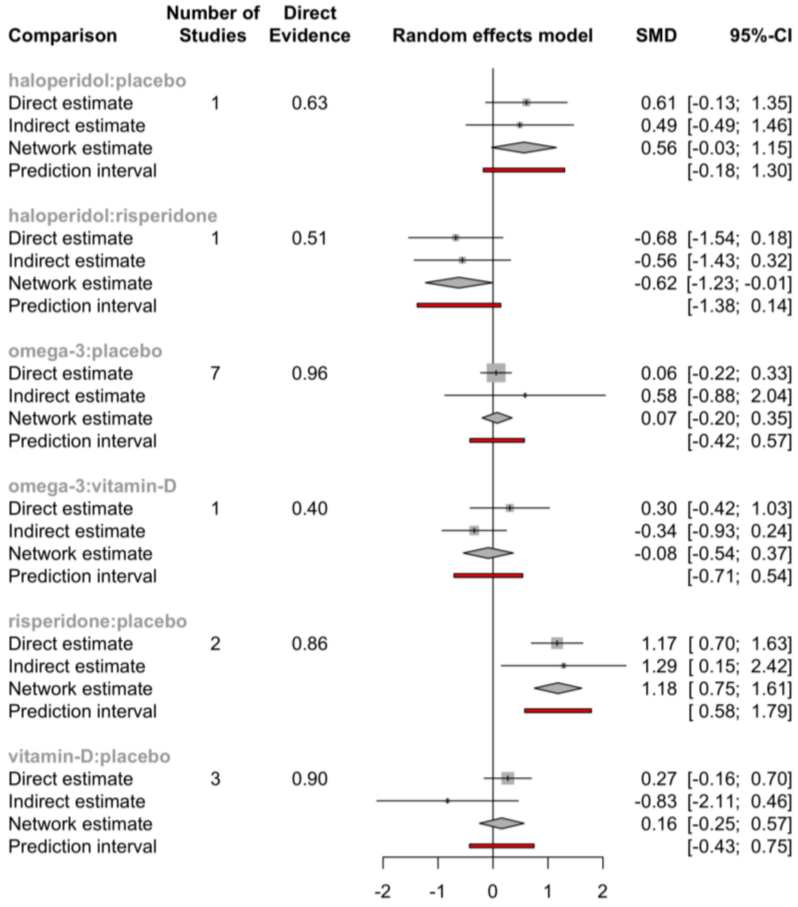

0% of the loops were incoherent. One study was excluded from the primary analysis of this outcome (Nikvarz et al. 2017), that compared risperidone with memantine. This comparison introduced incoherence for the loops between metmantine, risperidone and placebo.

6.5.2.4 Irritability

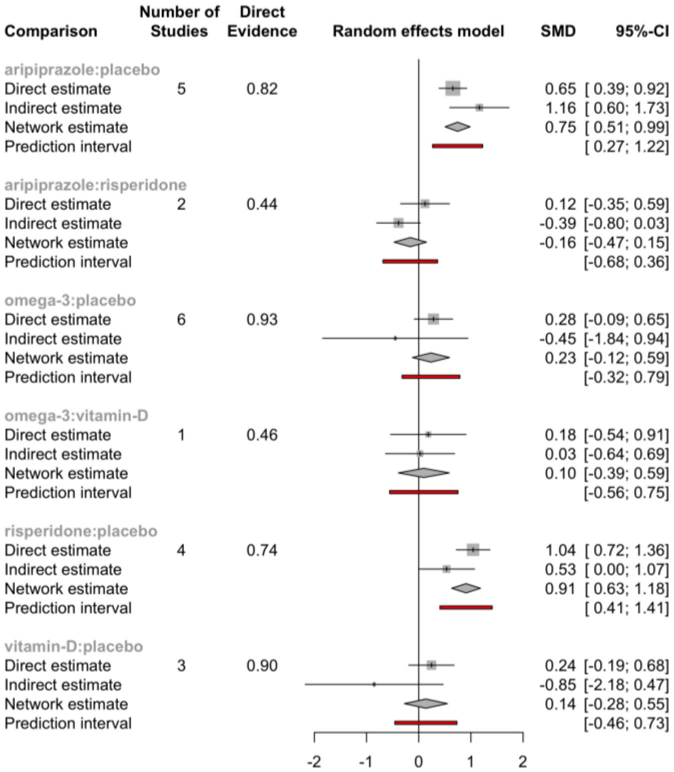

0% of the loops were incoherence

6.5.2.5 ADHD symptoms

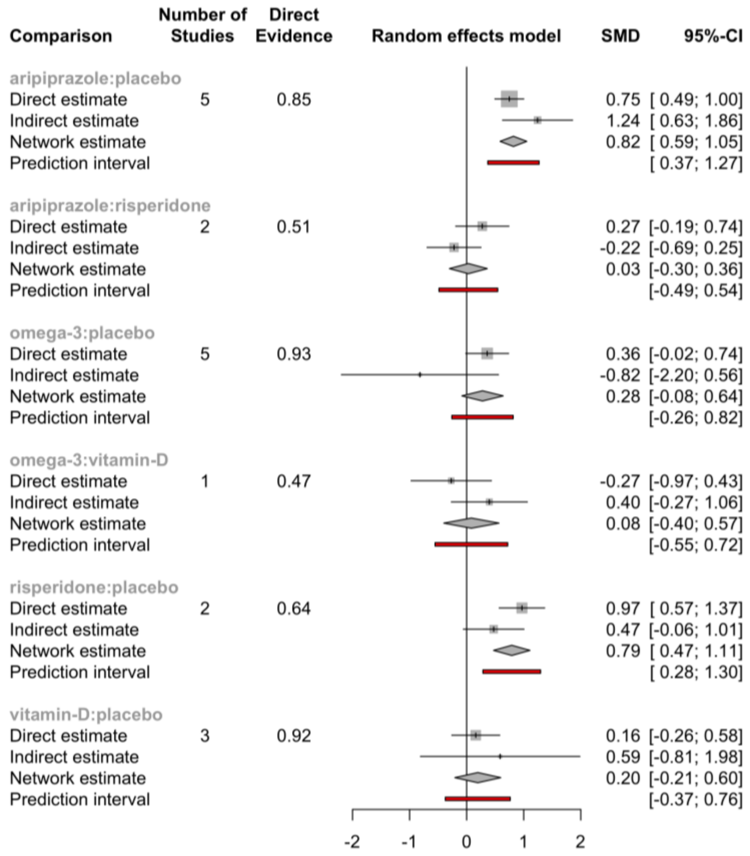

0% of the loops were incoherent.

6.5.2.6 Anxiety and depressive symptoms

No closed loop in children/adolescents and adults/mixed popoulations.

6.5.2.7 Response to treatment

Comparison	Number of Studies	Direct Evidence	OR	95%-CI
**aripiprazole:placebo**				
Direct estimate	5	0.81	3.88	[2.51; 6.00]
Indirect estimate			11.62	[4.77; 28.32]
Network estimate			4.79	[3.24; 7.09]
Prediction interval				[3.03; 7.60]
**aripiprazole:risperidone**				
Direct estimate	3	0.53	1.15	[0.58; 2.26]
Indirect estimate			0.38	[0.19; 0.79]
Network estimate			0.69	[0.42; 1.13]
Prediction interval				[0.39; 1.20]
**haloperidol:olanzapine**				
Direct estimate	1	0.58	0.20	[0.01; 2.93]
Indirect estimate			0.97	[0.04; 23.04]
Network estimate			0.39	[0.05; 3.00]
Prediction interval				[0.05; 3.30]
**haloperidol:risperidone**				
Direct estimate	1	0.86	0.55	[0.12; 2.58]
Indirect estimate			0.11	[0.00; 5.31]
Network estimate			0.44	[0.10; 1.85]
Prediction interval				[0.10; 1.99]
**memantine:placebo**				
Direct estimate	1	0.54	2.94	[0.73; 11.89]
Indirect estimate			2.51	[0.56; 11.26]
Network estimate			2.73	[0.98; 7.60]
Prediction interval				[0.92; 8.09]
**memantine:risperidone**				
Direct estimate	1	0.52	0.36	[0.09; 1.51]
Indirect estimate			0.43	[0.10; 1.87]
Network estimate			0.39	[0.14; 1.10]
Prediction interval				[0.13; 1.17]
**olanzapine:placebo**				
Direct estimate	1	0.57	4.00	[0.26; 60.80]
Indirect estimate			19.35	[0.84; 446.90]
Network estimate			7.87	[1.01; 61.49]
Prediction interval				[0.92; 67.57]
**omega-3:vitamin-D**				
Direct estimate	1	0.96	1.37	[0.35; 5.43]
Indirect estimate			0.00	[0.00; 3.06]
Network estimate			1.07	[0.28; 4.12]
Prediction interval				[0.26; 4.43]
**risperidone:placebo**				
Direct estimate	5	0.59	10.78	[5.84; 19.88]
Indirect estimate			3.70	[1.78; 7.71]
Network estimate			6.95	[4.34; 11.13]
Prediction interval				[4.07; 11.86]
**vitamin-D:placebo**				
Direct estimate	1	0.26	9.65	[0.49; 189.08]
Indirect estimate			0.50	[0.08; 2.92]
Network estimate			1.08	[0.24; 4.96]
Prediction interval				[0.22; 5.35]

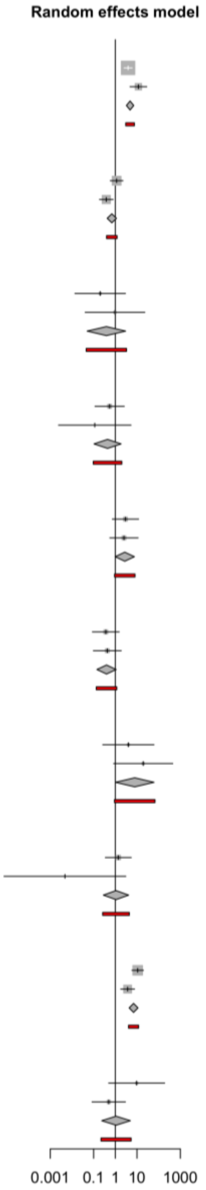

50% of the loops were incoherent, i.e., the comparisons of aripiprazole/placebo, aripiprazole/risperidone, omega-3/vitamin-D, risperidone/placebo, vitamin-D/placebo. Therfore, pairwise meta-analysis was conducted.

6.5.2.8 Global functioning

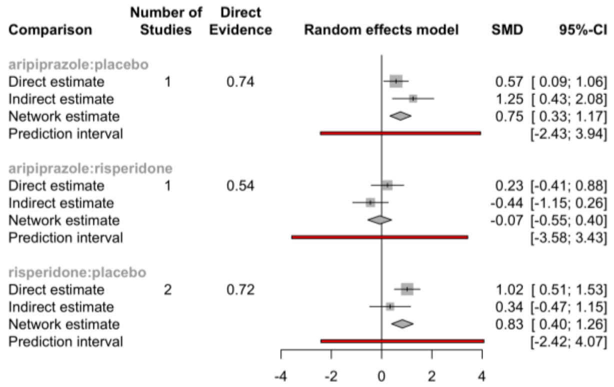

0% of the loops were incoherent.

6.5.2.9 Quality of life
No closed loop in children/adolescents and adults/mixed popoulations.

6.5.2.10 Parental stress
No closed loop in children/adolescents and adults/mixed popoulations.

6.5.2.11 Discontinuation due to any reason

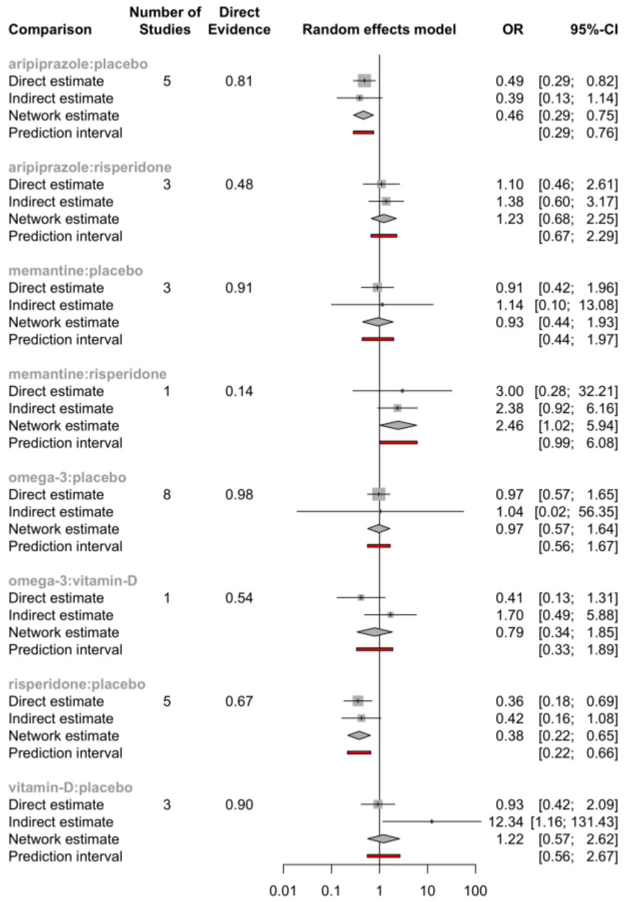

12.5% of the loops were incoherent, i.e., the comparison of vitamin-D and placebo.

6.5.2.12 Discontinuation due to adverse event

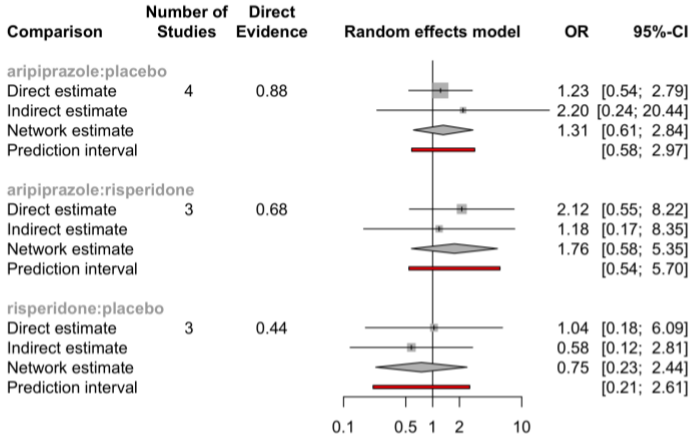

0% of the loops were incoherent.

6.5.2.13 Any adverse event

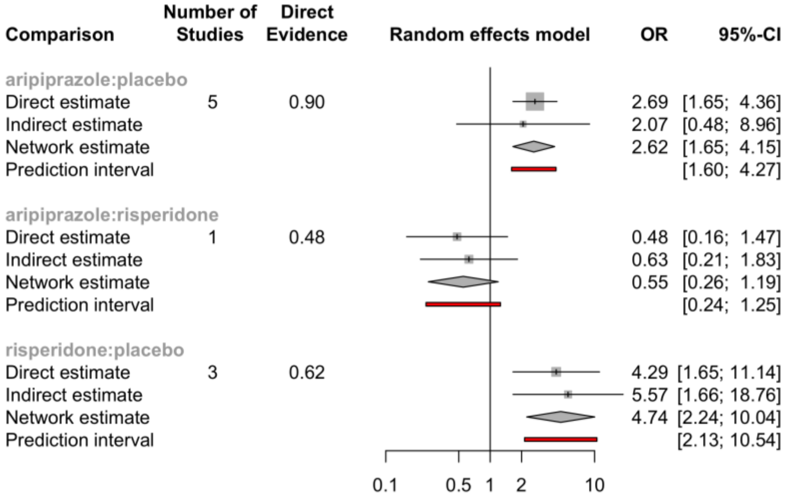

0% of the loops were incoherent.

6.5.2.14 Weight gain

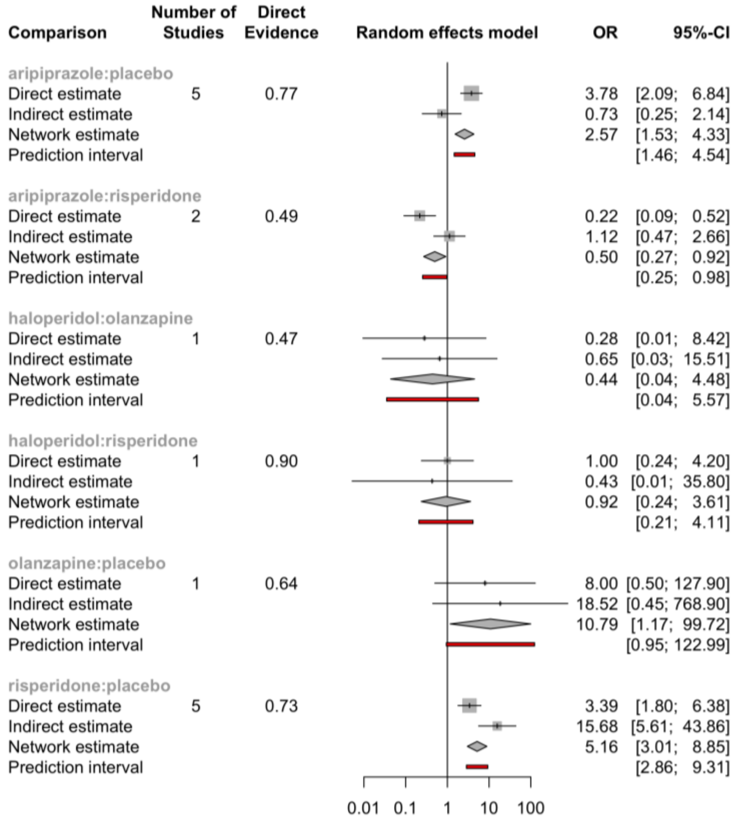

50% of the loops were incoherent, i.e., risperidone/placebo, aripiprazole/placebo, and aripiprazole/risperidone. Therefore, pairwise meta-analysis was conducted.

6.5.2.15 Sedation

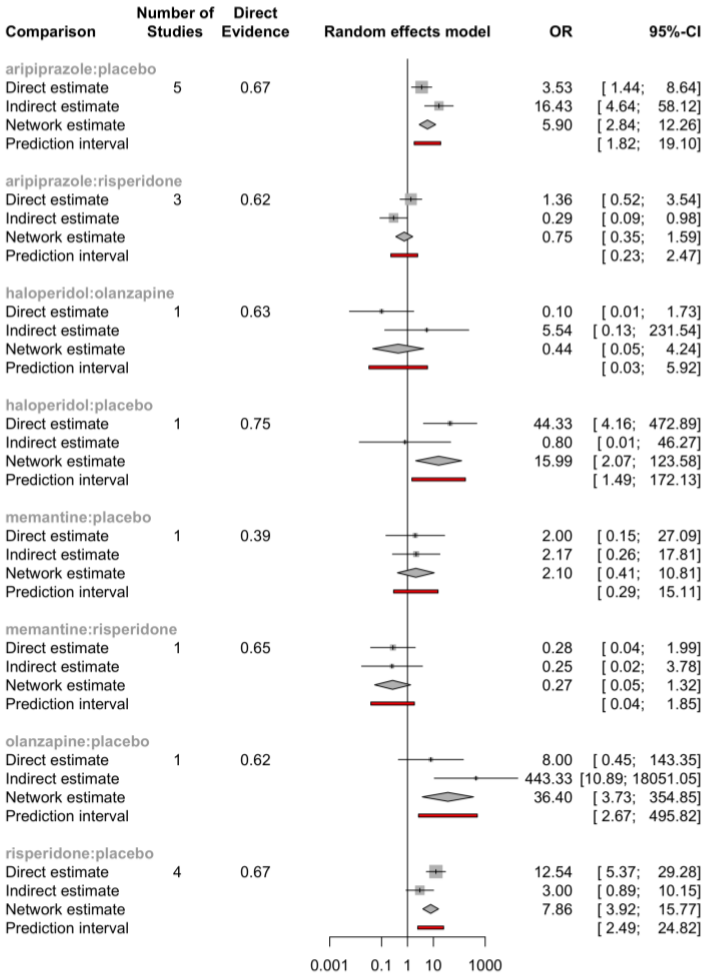

75% of the loops were incoherent, i.e., aripiprazole/placebo, aripiprazole/risperidone, haloperidol/olanzapine, haloperidol/placebo, olanzapine/placebo and risperidone/placebo. Therefore, pairwise meta-analysis was conducted.

6.5.2.16 Extrapyramidal symptoms

No closed loop in children/adolescents and adults/mixed popoulations.

6.5.3. References

1. Turner RM, Davey J, Clarke MJ, Thompson SG, Higgins JP. Predicting the extent of heterogeneity in meta-analysis, using empirical data from the Cochrane Database of Systematic Reviews. International journal of epidemiology. 2012;41(3):818-27. doi: 10.1093/ije/dys041.
2. Veroniki AA, Vasiliadis HS, Higgins JP, Salanti G. Evaluation of inconsistency in networks of interventions. Int J Epidemiol. 2013;42(1):332-45. doi: 10.1093/ije/dys222.

6.6. Sensitivity analysis

Sensitivity analyses for the primary outcomes are presented in Figure-S4. Sensitivity analyses have often smaller statistical power, since fewer studies are usually analyzed. Therefore, we considered the number of studies in the sensitivity analysis, and evaluated changes in the magnitude of the mean effect-sizes, the overlapping of the confidence intervals and whether the sensitivity analysis changed the interpretation of the findings. The results were in general robust across several sensitivity analyses. Some notable differences ($|SMD_{sensitivity} - SMD_{primary}| \geq 0.1$) are discussed below.

Social-communication difficulties

Children/adolescents

	**Sensitivity analysis**				**Primary analysis**			**Evaluation**	
**Drug**	**analysis**	**k_{sens}**	**n_{sens}**	**SMD_{sens}**	**$k_{primary}$**	**$n_{primary}$**	**$SMD_{primary}$**	**$SMD_{sensitivity}$- $SMD_{primary}$**	**Comment**
memantine	04. nonimputed SDs	1	15	0.46	2	69	-0.02	0.47	The results of the sensitivity analysis were based on indirect evidence from a single and small study (Nikvarz et al 2017). Confidence intervals overlapped. Interpretation of the findings of the primary analysis did not change.
memantine	12. ABC-L/SW	1	15	0.46	2	69	-0.02	0.47	The results of the sensitivity analysis were based on indirect evidence from a single and small study (Nikvarz et al 2017). Confidence intervals overlapped. Interpretation of the findings of the primary analysis did not change.
carnosine	10. ITT	1	15	0.35	2	36	0.09	0.26	The results of the sensitivity analysis were based on a single study. Confidence intervals overlapped. Interpretation of the findings of the primary analysis did not change.
carnosine	16. no associated symptoms	1	15	0.35	2	36	0.09	0.26	The results of the sensitivity analysis were based on a single study. Confidence intervals overlapped. Interpretation of the findings of the primary analysis did not change.
carnosine	17. more developed countries	1	15	0.35	2	36	0.09	0.26	The results of the sensitivity analysis were based on a single study. Confidence intervals overlapped. Interpretation of the

									findings of the primary analysis did not change.
omega-3	12. ABC-L/SW	6	79	0.45	10	171	0.21	0.24	SMD was larger when ABC-L/SW was used (0.45 vs. 0.24). Confidence intervals partially overlapped. The results were based on six out of ten studies (46% of the participants). The interpretation of the finding of the primary analysis did not change, i.e., that omega-3 could potentially improve social-communication difficulties with small effect-sizes.
aripiprazole	16. no associated symptoms	1	29	0.41	6	341	0.27	0.14	The results of the sensitivity analysis were based on a single study. Confidence intervals overlapped. Interpretation of the findings of the primary analysis did not change.
cholesterol	06. r=0.75	1	8	0.46	1	8	0.33	0.13	The precision of results is higher when a larger pre-post correlation is assumed (e.g., r=0.75). In that case, SMDs are more precise and larger. However, it is unlikely that scales measuring behavioral symptoms have such large correlations. The interpretation of the findings of the primary analysis did not change.
risperidone	17. more developed countries	2	88	0.43	4	133	0.31	0.13	The results were based on two out of the four studies in the primary analysis. Confidence intervals overlapped. The interpretation of the findings of the primary analysis did not change.
risperidone	02. pairwise	2	88	0.43	4	133	0.31	0.13	The results were based on two out of the four studies (placebo-controlled, direct evidence). Confidence intervals overlapped. The interpretation of the findings of the primary analysis did not change.
bumetanide	17. more developed countries	3	115	0.26	4	174	0.14	0.12	Confidence intervals overlapped, and the interpretation of the findings of the primary analysis did not change.
risperidone	18. low ROB (sequence generation and allocation concealment)	2	88	0.43	4	133	0.31	0.13	Confidence intervals overlapped. The interpretation of the findings of the primary analysis did not change.
n-acetylcysteine	12. ABC-L/SW	2	27	-0.10	3	75	-0.21	0.11	The results were based on two out of the four available studies, and confidence

									intervals overlapped. The interpretation of the primary analysis did not change.
whey-protein	06. r=0.75	1	19	0.32	1	19	0.21	0.10	The precision of results is higher when a larger pre-post correlation is assumed (e.g., r=0.75). In that case, SMDs are more precise and larger. However, it is unlikely that scales measuring behavioral symptoms have such large correlations. The interpretation of the findings of the primary analysis did not change.
folinic acid	02. pairwise	2	32	0.54	2	32	0.44	0.10	There was heterogeneity in the results of the pairwise meta-analysis. Confidence intervals overlapped. Interpretation of the findings of the primary analysis did not change.
probiotics	11. clinician ratings	1	31	0.31	5	92	0.21	0.10	The results of the sensitivity analysis were based on a single study. Confidence intervals overlapped. Interpretation of the findings of the primary analysis did not change.
aripiprazole	18. low RoB (sequence generation and allocation concealment)	2	210	0.16	6	341	0.27	-0.11	The results of the senstivitiy analysis were based on two out of the six studies. Confidence intervals overlapped. Interpretation of the findings of the primary analysis did not change.
memantine	18. low RoB (sequence generation and allocation concealment)	1	54	-0.13	2	69	-0.02	-0.12	The results of the sensitivity analysis were based on a single study. Confidence intervals overlapped. Interpretation of the findings of the primary analysis did not change.
memantine	02. pairwise	1	54	-0.13	2	69	-0.02	-0.12	The results of the sensitivity analysis were based on a single study. Confidence intervals overlapped. Interpretation of the findings of the primary analysis did not change.
memantine	17. more developed countries	1	54	-0.13	2	69	-0.02	-0.12	The results of the sensitivity analysis were based on a single study. Confidence intervals overlapped. Interpretation of the findings of the primary analysis did not change.
memantine	10. ITT	1	54	-0.13	2	69	-0.02	-0.12	The results of the sensitivity analysis were based on a single study. Confidence intervals overlapped. Interpretation of the

									findings of the primary analysis did not change.
memantine	07. low/moderate RoB	1	54	-0.13	2	69	-0.02	-0.12	The results of the sensitivity analysis were based on a single study. Confidence intervals overlapped. Interpretation of the findings of the primary analysis did not change.
memantine	09. blinded trials	1	54	-0.13	2	69	-0.02	-0.12	The results of the sensitivity analysis were based on a single study. Confidence intervals overlapped. Interpretation of the findings of the primary analysis did not change.
carnosine	13. DSM/ICD	1	21	-0.03	2	36	0.09	-0.12	The results of the sensitivity analysis were based on a single study. Confidence intervals overlapped. Interpretation of the findings of the primary analysis did not change.
carnosine	13. DSM/ICD	1	21	-0.03	2	36	0.09	-0.12	The results of the sensitivity analysis were based on a single study. Confidence intervals overlapped. Interpretation of the findings of the primary analysis did not change.
bumetanide	12. ABC-L/SW	1	38	0.00	4	174	0.14	-0.14	The results of the sensitivity analysis are based on a single study. Confidence intervals overlapped. Interpretation of the findings of the primary analysis did not change.
simvastatin	06. r=0.75	1	12	-0.45	1	12	-0.30	-0.15	The precision of results is higher when a larger pre-post correlation is assumed (e.g., r=0.75). In that case, SMDs are more precise and larger. However, it is unlikely that scales measuring behavioral symptoms have such large correlations. Confidence intervals overlapped. The interpretation of the findings of the primary analysis did not change.
omega-3	16. no associated symptoms	6	112	0.05	10	171	0.21	-0.16	SMD was smaller when studies focusing on associated symptoms were excluded (0.05 vs 0.21). Confidence intervals partially overlapped. The results were based on 6 out of 10 studies (65% of the participants).
omega-3	11. clinician ratings	3	53	0.03	10	171	0.21	-0.18	SMD was smaller when clinician ratings were used (0.03 vs 0.21). Confidence

									intervals overlapped. The results were based on 3 out of 10 studies (31% of the participants). It should be noted that SMD was larger when ABC-L/SW (a common caregiver-rating) was used (e.g., see sensitivity analysis above).
folinic acid	18. low RoB (sequence generation and allocation concealment)	1	23	0.23	2	32	0.44	-0.21	The results of the sensitivity analysis were based on a single study. Confidence intervals overlapped. Interpretation of the findings of the primary analysis did not change.
folinic acid	13. DSM/ICD	1	23	0.23	2	32	0.44	-0.21	The results of the sensitivity analysis were based on a single study. Confidence intervals overlapped. Interpretation of the findings of the primary analysis did not change.
folinic acid	13. DSM/ICD	1	23	0.23	2	32	0.44	-0.21	The results of the sensitivity analysis were based on a single study. Confidence intervals overlapped. Interpretation of the findings of the primary analysis did not change.
folinic acid	07. low/moderate RoB	1	23	0.23	2	32	0.44	-0.21	The results of the sensitivity analysis were based on a single study. Confidence intervals overlapped. Interpretation of the findings of the primary analysis did not change.
folinic acid	12. ABC-L/SW	1	23	0.20	2	32	0.44	-0.24	The results of the sensitivity analysis were based on a single study. Confidence intervals overlapped. Interpretation of the findings of the primary analysis did not change.
arbaclofen	12. ABC-L/SW	1	76	-0.11	1	76	0.16	-0.26	SMD was smaller when ABC-L/SW was used (-0.11 vs 0.16). Confidence intervals partially overlapped. They both did not suggest differences with placebo. Therefore, the interpretation of the results of the primary analysis did not change.

Adults

	Sensitivity analysis	Primary analysis	Evaluation

Drug	analysis	k_{sens}	n_{sens}	SMD_{sens}	$k_{primary}$	$n_{primary}$	$SMD_{primary}$	SMD_{sens}-$SMD_{primary}$	Comment
balovaptan	12. ABC-L/SW	1	111	0.31	2	222	0.06	0.25	The results were based on one of the two studies. Confidence intervals overlapped. Interpretation of the findings of the primary analysis did not change.
oxytocin	10. ITT	3	106	-0.11	4	115	0.01	-0.12	Confidence intervals overlapped. Interpretation of the findings of the primary analysis did not change.
oxytocin	12. ABC-L/SW	2	55	-0.18	4	115	0.01	-0.18	Confidence intervals overlapped. Interpretation of the findings of the primary analysis did not change.

Repetitive behaviors

Children/adolescents

	Sensitivity analysis				Primary analysis			Evaluation	
**Drug**	**analysis**	k_{sens}	n_{sens}	SMD_{sens}	$k_{primary}$	$n_{primary}$	$SMD_{primary}$	SMD_{sens}-$SMD_{primary}$	**Comment**
lamotrigine	12. ABC-S	1	14	0.63	1	11	-0.04	0.66	SMD was larger when ABC-S was used, yet still not formally statistically significant at two-sided alpha 0.05. Confidence intervals partially overlapped. The results were based on a single study with unclear reporting of ABC-S (Belsito 2001). Mean scores of ABC-S were extracted from Fig.2. of the manuscript and standard deviations were estimated using the reported p=0.10. Mean scores in Fig.2 ranged from about 65 to about 85, yet the maximum score of ABC-S should be 21 (Aman et al 1985). Nevertheless, the interpretation of the findings of the primary analysis did not change.
vitamin-D	11. clinician ratings	2	47	0.46	5	84	0.16	0.30	SMD seemed to be larger when clinician ratings were used (0.46 vs. 0.16), yet confidence intervals overlapped. The results were based

									on two studies from Iran out of five (56% of the participants) that used clinician-ratings of ABC-S and GARS-2-S. Therefore, the interpretation of the results of the primary analysis did not change.
omega-3	11. clinician ratings	2	35	0.37	9	158	0.15	0.22	The results were based on two out of nine studies and confidence intervals overlapped. The interpretation of the findings of the primary analysis did not change.
folinic acid	06. r=0.75	1	23	0.69	1	23	0.50	0.19	The precision of results is higher when a larger pre-post correlation is assumed (e.g., r=0.75). In that case, SMDs are more precise and larger. However, it is unlikely that scales measuring behavioral symptoms have such large correlations. Confidence intervals overlapped. The interpretation of the findings of the primary analysis did not change.
guanfacine	06. r=0.75	1	30	0.72	1	30	0.55	0.18	The precision of results is higher when a larger pre-post correlation is assumed (e.g., r=0.75). In that case, SMDs are more precise and larger. However, it is unlikely that scales measuring behavioral symptoms have such large correlations. Confidence intervals overlapped. The interpretation of the findings of the primary analysis did not change.
sapropterin	06. r=0.75	1	23	0.46	1	23	0.32	0.14	The precision of results is higher when a larger pre-post correlation is assumed (e.g., r=0.75). In that case, SMDs are more precise and larger. However, it is unlikely that scales measuring behavioral symptoms have such large correlations. Confidence intervals overlapped. The interpretation of the findings of the primary analysis did not change.
atomoxetine	18. low RoB (sequence generation and	2	80	0.64	3	107	0.49	0.15	Confidence intervals overlapped. The interpretation of the findings of the primary analysis did not change.

	allocation concealment)								
memantine	06. r=0.75	1	15	0.61	1	15	0.47	0.14	The precision of results is higher when a larger pre-post correlation is assumed (e.g., r=0.75). In that case, SMDs are more precise and larger. However, it is unlikely that scales measuring behavioral symptoms have such large correlations. Confidence intervals overlapped. The interpretation of the findings of the primary analysis did not change.
risperidone	06. r=0.75	4	133	0.74	4	133	0.60	0.14	The precision of results is higher when a larger pre-post correlation is assumed (e.g., r=0.75). In that case, SMDs are more precise and larger. However, it is unlikely that scales measuring behavioral symptoms have such large correlations. Confidence intervals overlapped. The interpretation of the findings of the primary analysis did not change.
atomoxetine	06. r=0.75	3	107	0.61	3	107	0.49	0.11	The precision of results is higher when a larger pre-post correlation is assumed (e.g., r=0.75). In that case, SMDs are more precise and larger. However, it is unlikely that scales measuring behavioral symptoms have such large correlations. Confidence intervals overlapped. The interpretation of the findings of the primary analysis did not change.
n-acetylcysteine	12. ABC-S	2	27	0.18	3	75	0.08	0.10	Confidence intervals overlapped. The interpretation of the findings of the primary analysis did not change.
atomoxetine	05. r=0.25	3	107	0.40	3	107	0.49	-0.10	Confidence intervals overlapped. The interpretation of the findings of the primary analysis did not change.
folinic acid	05. r=0.25	1	23	0.40	1	23	0.50	-0.10	Confidence intervals overlapped. The interpretation of the findings of the primary analysis did not change.
citalopram	12. ABC-S	1	73	-0.08	1	73	0.03	-0.11	Confidence intervals overlapped. The interpretation of the findings of the primary analysis did not change.

risperidone	16. no associated symptoms	3	84	0.48	4	133	0.60	-0.11	Confidence intervals overlapped. The interpretation of the findings of the primary analysis did not change.
memantine	16. no associated symptoms	1	15	0.36	1	15	0.47	-0.11	Confidence intervals overlapped. The results were based on indirect evidence for both the primary and sensitivity analysis based on a single and small study (Nikvarz et al 2017). The interpretation of the findings of the primary analysis did not change.
vitamin-D	18. low RoB (sequence generation and allocation concealment)	3	41	0.04	5	84	0.16	-0.12	Confidence intervals overlapped. The interpretation of the findings of the primary analysis did not change.
guanfacine	05. r=0.25	1	30	0.42	1	30	0.55	-0.13	Confidence intervals overlapped. The interpretation of the findings of the primary analysis did not change.
simvastatin	06. r=0.75	1	13	-0.44	1	13	-0.30	-0.14	The precision of results is higher when a larger pre-post correlation is assumed (e.g., r=0.75). In that case, SMDs are more precise and larger. However, it is unlikely that scales measuring behavioral symptoms have such large correlations. Confidence intervals overlapped. The interpretation of the findings of the primary analysis did not change.
fluoxetine	07. low/moderate RoB	2	95	-0.07	3	170	0.09	-0.15	The results were based on two out of the three studies (56% of the participants). Confidence intervals overlapped. The interpretation of the findings of the primary analysis did not change.
probiotics	12. ABC-S	2	18	-0.22	4	85	-0.03	-0.19	Confidence intervals overlapped. The interpretation of the findings of the primary analysis did not change.
vitamin-D	12. ABC-S	4	59	-0.04	5	84	0.16	-0.20	Confidence intervals generally overlapped. The interpretation of the findings of the primary analysis did not change.
guanfacine	12. ABC-S	1	30	0.29	1	30	0.55	-0.25	SMD was smaller when ABC-S (a common caregiver-rating) was used (0.29 vs. 0.55). Confidence intervals

									partially overlapped. The interpretation of the findings of the primary analysis did not change, i.e., that guanfacine could potentially improve repetitive behaviors.<br>It should be noted that CYBOCS-PDD (a common clinician-rating) was used in the primary analysis.
fluoxetine	12. ABC-S	1	75	-0.18	3	170	0.09	-0.26	The results were based on one out of the three studies (44% of the participants). Confidence intervals generally overlapped. The interpretation of the findings of the primary analysis did not change.
vitamin-D	16. no associated symptoms	2	40	-0.11	5	84	0.16	-0.26	The results were based on two out of the five studies (about 50% of the participants). Confidence intervals generally overlapped. The interpretation of the findings of the primary analysis did not change.
bumetanide	12. ABC-S	1	38	0.08	4	175	0.35	-0.27	The results were based on one out of the four studies (21% of the participants). Confidence intervals generally overlapped. The interpretation of the findings of the primary analysis did not change.
vitamin-D	17. more developed countries	3	37	-0.16	5	84	0.16	-0.32	Confidence intervals generally overlapped. The interpretation of the findings of the primary analysis did not change.

Adults

	Sensitivity analysis				Primary analysis			Evaluation	
**Drug**	**analysis**	**k_{sens}**	**n_{sens}**	**SMD_{sens}**	**$k_{primary}$**	**$n_{primary}$**	**$SMD_{primary}$**	**SMD_{sens}-$SMD_{primary}$**	**Comment**
fluoxetine	06. r=0.75	1	21	1.70	1	21	1.20	0.49	The precision of results is higher when a larger pre-post correlation is assumed (e.g., r=0.75). In that case, SMDs are more precise and larger. However, it is unlikely that scales measuring behavioral symptoms have such large correlations. The interpretation of the

									findings of the primary analysis did not change.
fluvoxamine	06. r=0.75	1	15	1.51	1	15	1.04	0.47	The precision of results is higher when a larger pre-post correlation is assumed (e.g., r=0.75). In that case, SMDs are more precise and larger. However, it is unlikely that scales measuring behavioral symptoms have such large correlations. The interpretation of the findings of the primary analysis did not change.
risperidone	06. r=0.75	1	14	1.35	1	14	0.97	0.38	The precision of results is higher when a larger pre-post correlation is assumed (e.g., r=0.75). In that case, SMDs are more precise and larger. However, it is unlikely that scales measuring behavioral symptoms have such large correlations. The interpretation of the findings of the primary analysis did not change.
fluvoxamine	05. r=0.25	1	15	0.88	1	15	1.04	-0.16	Confidence intervals overlapped. The interpretation of the findings of the primary analysis did not change.
risperidone	05. r=0.25	1	14	0.78	1	14	0.97	-0.19	Confidence intervals overlapped. The interpretation of the findings of the primary analysis did not change.
fluoxetine	05. r=0.25	1	21	0.98	1	21	1.20	-0.22	Confidence intervals overlapped. The interpretation of the findings of the primary analysis did not change.

Overall core symptoms

Children/adolescents

	**Sensitivity analysis**				**Primary analysis**			**Evaluation**	
**Drug**	**analysis**	k_{sens}	n_{sens}	SMD_{sens}	$k_{primary}$	$n_{primary}$	$SMD_{primary}$	$SMD_{sens}-SMD_{primary}$	**Comment**

folinic acid	11. clinician ratings	1	9	1.11	2	32	0.12	0.99	There was heterogeneity in the primary analysis, which included two small studies on folinic acid. The results of this sensitivity analysis were based on a single and small study that demonstrated a large SMD (NCT02551380). Confidence intervals partially overlapped. The results are inconclusive.
risperidone	06. r=0.75	3	81	1.65	3	81	1.18	0.47	The precision of results is higher when a larger pre-post correlation is assumed (e.g., r=0.75). In that case, SMDs are more precise and larger. However, it is unlikely that scales measuring behavioral symptoms have such large correlations. The interpretation of the findings of the primary analysis did not change.
risperidone	15. no associated symptoms	2	32	1.54	3	81	1.18	0.36	Confidence intervals overlapped. The interpretation of the results of the primary analysis did not change.
risperidone	11. clinician ratings	2	32	1.54	3	81	1.18	0.36	Confidence intervals overlapped. The interpretation of the results of the primary analysis did not change.
omega-3	11. clinician ratings	1	28	0.41	8	151	0.07	0.34	The results are based on one study (19% of the participants). Confidence intervals were generally overlapped. The interpretation of the primary analysis did not change.
memantine	17. including Nikvarz 2017	2	69	0.33	1	54	0.01	0.33	In this sensitivity analysis, a study that introduced incoherence (Nikvarz et al 2017) was included. In the primary analysis, this study was excluded. It compared risperidone and memantine, and found no difference between the two drugs. Confidence intervals partially overlapped. The interpretation of the results of the primary analysis did not change.

carnosine	11. clinician ratings	2	32	0.71	3	53	0.42	0.29	The results were based on two small studies, one of these was from Egypt and demonstrated a large effect-size (SMD=1.06). Confidence intervals partially overlapped. Therefore, the interpretation of the results of the primary analysis did not change.
carnosine	15. no associated symptoms	2	32	0.71	3	53	0.42	0.29	The results were based on the previous analysis. The interpretation of the results of the primary analysis did not change.
probiotics	11. clinician ratings	1	31	0.44	4	85	0.20	0.24	The results were based on one out four studies (36% of the participants). Confidence intervals generally overlapped. The interpretation of the results of the primary analysis did not change.
folinic acid	02. pairwise	32	2	0.35	2	32	0.12	0.23	There was heterogeneity in the primary analysis, which is reflected by the less precise yet a bit larger SMD in the pairwise meta-analysis. The interpretation of the results of the primary analysis did not change.
sapropterin	06. r=0.75	1	23	0.60	1	23	0.41	0.18	The precision of results is higher when a larger pre-post correlation is assumed (e.g., r=0.75). In that case, SMDs are more precise and larger. However, it is unlikely that scales measuring behavioral symptoms have such large correlations. The interpretation of the findings of the primary analysis did not change.
atomoxetine	18. low RoB (sequence generation and allocation concealment)	1	48	0.33	2	73	0.15	0.17	Confidence intervals overlapped. Interpretation of the primary analysis dit not change.
carnosine	06. r=0.75	3	53	0.58	3	53	0.42	0.16	The precision of results is higher when a larger pre-post correlation is assumed (e.g., r=0.75). In that case, SMDs are more precise and larger. However, it is unlikely that scales measuring behavioral symptoms have such large

									correlations. The interpretation of the findings of the primary analysis did not change.
vitamin-D	11. clinician ratings	1	22	0.31	4	59	0.16	0.15	The results were based on one out four studies (37% of the participants). Confidence intervals overlapped. The interpretation of the findings of the primary analysis did not change.
haloperidol	15. no associated symptoms	2	30	0.71	3	36	0.56	0.15	Confidence intervals overlapped. The interpretation of the findings of the primary analysis did not change.
olanzapine	11. clinician ratings	1	6	1.34	1	6	1.20	0.15	Confidence intervals overlapped. The interpretation of the findings of the primary analysis did not change.
haloperidol	11. clinician ratings	3	36	0.71	3	36	0.56	0.15	Confidence intervals overlapped. The interpretation of the findings of the primary analysis did not change.
bumetanide	11. clinician ratings	3	151	0.76	4	189	0.61	0.15	Confidence intervals overlapped. The interpretation of the findings of the primary analysis did not change.
omega-3	12. DSM/ICD	7	122	0.19	8	151	0.07	0.12	The results were based on seven out of eight studies (81% of the participants). One study that did not use standardized diagnostic criteria to include participants was excluded (Bent et al 2014). SMD was small but a bit larger than the primary analysis (0.19 vs. 0.07). Confidence intervals generally overlapped. The interpretation of the findings of the primary analysis did not change.
bumetanide	10. ITT	2	124	0.73	4	189	0.61	0.12	Confidence intervals overlapped. The interpretation of the findings of the primary analysis did not change.
vitamin-D	15. no associated symptoms	2	40	0.27	4	59	0.16	0.11	Confidence intervals overlapped. The interpretation of the findings

									of the primary analysis did not change.
vitamin-D	07. low/moderate RoB	2	40	0.27	4	59	0.16	0.11	Confidence intervals overlapped. The interpretation of the findings of the primary analysis did not change.
vitamin-D	02. pairwise	59	3	0.27	4	59	0.16	0.11	Confidence intervals overlapped. The interpretation of the findings of the primary analysis did not change.
omega-3	07. low/moderate RoB	6	128	-0.02	8	151	0.07	-0.10	Confidence intervals overlapped. The interpretation of the findings of the primary analysis did not change.
vitamin-D	16. more developed countries	3	37	0.06	4	59	0.16	-0.10	Confidence intervals overlapped. The interpretation of the findings of the primary analysis did not change.
bumetanide	16. more developed countries	3	130	0.50	4	189	0.61	-0.11	Confidence intervals overlapped. The interpretation of the findings of the primary analysis did not change.
risperidone	17. including Nikvarz 2017	4	96	1.00	3	81	1.18	-0.18	Confidence intervals overlapped. The interpretation of the results of the primary analysis did not change.
risperidone	05. r=0.25	3	81	0.98	3	81	1.18	-0.20	Confidence intervals overlapped. The interpretation of the results of the primary analysis did not change.
risperidone	16. more developed countries	1	49	0.93	3	81	1.18	-0.25	Confidence intervals overlapped. The interpretation of the results of the primary analysis did not change.
risperidone	04. nonimputed SDs	1	49	0.93	3	81	1.18	-0.25	Confidence intervals overlapped. The interpretation of the results of the primary analysis did not change.
carnosine	12. DSM/ICD	1	21	0.03	3	53	0.42	-0.39	The results were based on one out of the three studies (40% of the participants. Two studies were excluded that did not use standardized diagnostic criteria (Geier 2011 and Fahmy et al.

									2013). The latter was a small study from Egypt that demonstrated a large effect-size (SMD=1.06). In this sensitivity analysis, the SMD was smaller (0.03 vs. 0.42) and confidence intervals partially overlapped. Therefore, the results of the primary analysis are inconclusive.
folinic acid	07. low/moderate RoB	1	23	-0.30	2	32	0.12	-0.42	There was heterogeneity in the primary analysis that was based on two small studies. In this analysis, a study that demonstrated large effect-size (SMD=1.11) and had high risk-of-bias was excluded (NCT02551380). Confidence intervals partially overlapped. The results of the primary analysis were inconclusive.
folinic acid	12. DSM/ICD	1	23	-0.30	2	32	0.12	-0.42	There was heterogeneity in the primary analysis that was based on two small studies. In this analysis, a study that demonstrated large effect-size (SMD=1.11) and did not use standardized diagnostic criteria was excluded (NCT02551380). Confidence intervals partially overlapped. The results of the primary analysis were inconclusive.
folinic acid	18. low RoB (sequence generation and allocation concealment)	1	23	-0.30	2	32	0.12	-0.42	There was heterogeneity in the primary analysis that was based on two small studies. In this analysis, a study that demonstrated large effect-size (SMD=1.11) and did not use standardized diagnostic criteria was excluded (NCT02551380). Confidence intervals partially overlapped. The results of the primary analysis were inconclusive.

Adults

	Sensitivity analysis				Primary analysis			Evaluation	
**Drug**	**analysis**	**k_{sens}**	**n_{sens}**	**SMD_{sens}**	**$k_{primary}$**	**$n_{primary}$**	**$SMD_{primary}$**	**SMD_{sens}-$SMD_{primary}$**	**Comment**
risperidone	06. r=0.75	1	14	0.66	1	14	0.49	0.18	The precision of results is higher when a larger pre-post correlation is assumed (e.g., r=0.75). In that case, SMDs are more precise and larger. However, it is unlikely that scales measuring behavioral symptoms have such large correlations. The interpretation of the findings of the primary analysis did not change.
risperidone	05. r=0.25	1	14	0.39	1	14	0.49	-0.10	Confidence intervals overlapped. The interpretation of the findings of the primary analysis did not change.

Dichotomous outcomes

In addition, relative risks were used for dichotomous in a sensitivity analysis, which is also presented in Figure-S4. The results did not materially change.

6.7. Effect sizes of individual studies

Effect sizes of individual studies and pairwise meta-analysis are presented in Figure-S3.
SMDs>0 are in favor of the first intervention, meaning more symptom reduction or more improvement in quality of life or global functioning. ORs>1 are in favor of the first intervention in case of response to treatment (higher OR means more patients with a response) or in favor of the second intervention in case of dropouts or adverse events (higher OR means fewer dropouts or patients with adverse events).

6.8. Assessment of publication bias and small-study effects

When there were more than 10 studies available, we investigated small-study effects with 1) comparison-adjusted funnel plots for the primary network meta-analysis assuming the direction of bias to the more recent interventions of the comparison (i.e. in our study: i) vitamin-D to omega-3, ii) memantine to aripiprazole to olanzapine to risperidone to clomipramine to haloperidol to placebo, iii) any pharmacological or dietary supplement to placebo); 2) contour-enhanced funnel plots for the placebo-controlled comparisons. Linear regression test for funnel plot asymmetry accompanied the visual inspection of the funnel plots.

6.8.1 Social-communication difficulties

	Comparison-adjusted funnel plot (t, df, p-value)	Placebo-controlled funnel plot (t, df, p-value)
Children/adolescents	t=1.79, df=69, p-value=0.078	t=1.778, df=66, p-value=0.080
Adults or mixed	Less than 10 studies were available for this outcome.	Less than 10 studies were available for this outcome.

Funnel plots for children/adolescents:

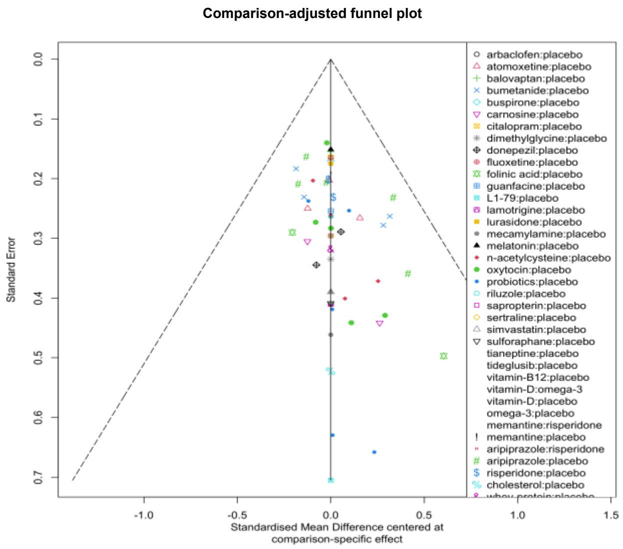

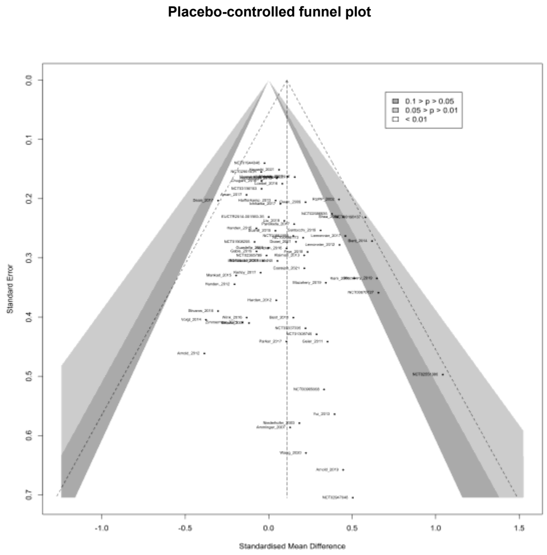

There was asymmetry in the comparison-adjusted and contour-enhanced funnel plot according to visual inspection and statistical tests (p-value=0.078<0.1 and p-value=0.08<0.1, respectively). Asymmetry was mainly due to smaller studies with larger effect sizes comparing aripiprazole, carnosine, folinic acid, n-acetylcysteine omega-3 and probiotics vs. placebo. Therefore, there may be a potential publication bias in studies favoring the experimental intervention in comparison to placebo, which might be more prominent in the above dietary-supplements.
There were less than 10 studies for adults or mixed populations.

6.8.2 Repetitive behaviors and restricted interests

	Comparison-adjusted funnel plot (t, df, p-value)	Placebo-controlled funnel plot (t, df, p-value)
Children/adolescents	t=0.784, df=60, p-value=0.436	t=1.4435, df=57, p-value=0.154
Adults or mixed	t=-0.837, df=12, p-value=0.419	t=0.929, df=12, p-value=0.371

Funnel plots for children/adolescents:

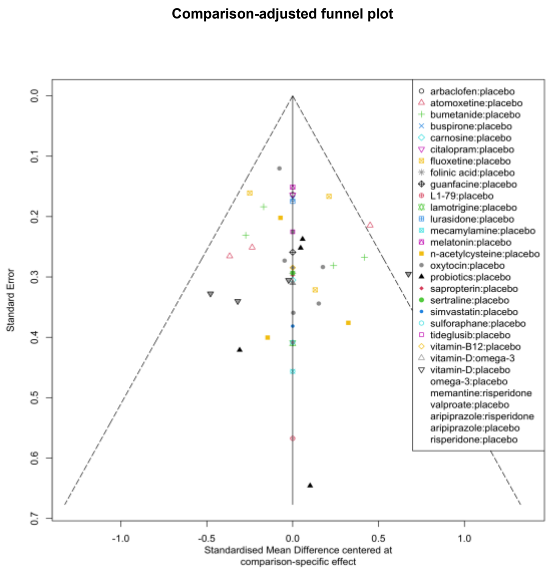

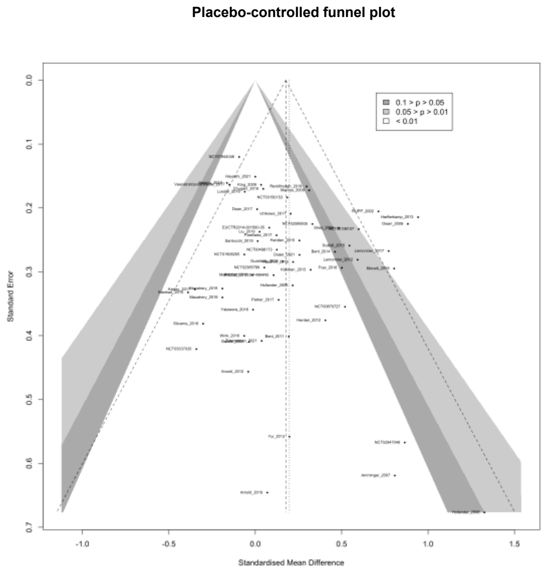

No clear asymmetry was observed in the comparison-adjusted funnel plot, yet asymmetry in the contour-enhanced funnel plot can be observed by visual inspection. This was driven mainly due to three small studies with larger effect sizes (NCT02847048: L1-79 vs. placebo, Amminger 2007: omega-3 vs. placebo and Hollander 2006: valproate vs. placebo). However, statistical tests were not formally significant (at alpha=0.1).

Funnel plots for adults or mixed populations:

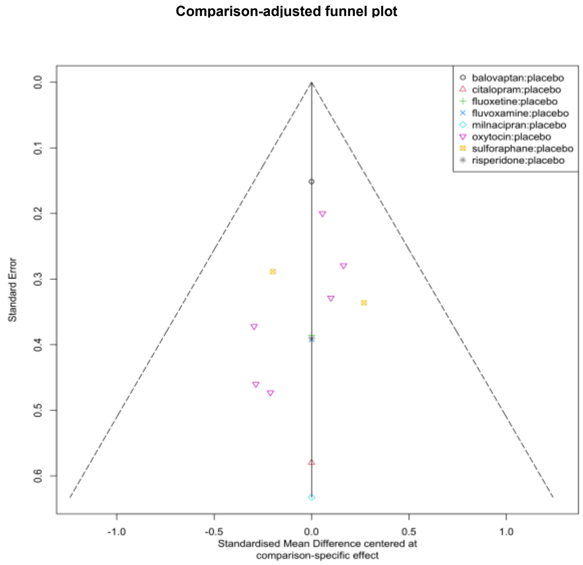

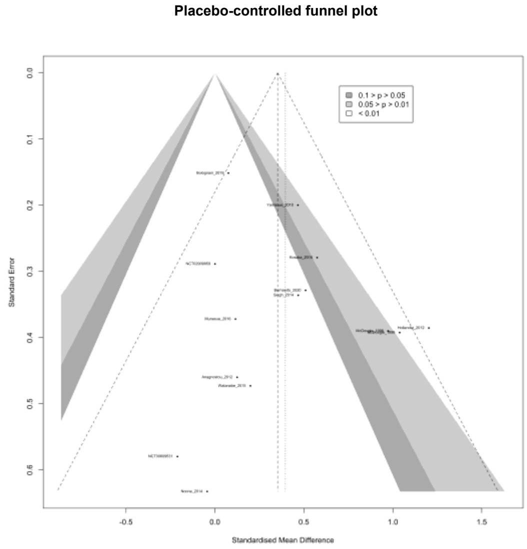

Asymmetry can be visually observed in both comparison-adjusted and contour-enhanced funnel plot, yet statistical tests were not formally significant (at alpha=0.1). The asymmetry was mainly driven by smaller studies that demonstrated smaller effect sizes for the comparison between oxytocin, citalopram and milnacipran vs. placebo.

6.8.3 Scales measuring overall core symptoms

	Comparison-adjusted funnel plot (t, df, p-value)	Placebo-controlled funnel plot (t, df, p-value)
Children/adolescents	t=1.304, df=54, p=0.1977	t=0.277, df=51, p=0.7829
Adults or mixed	Less than 10 studies were available.	Less than 10 studies were available.

Funnel plots for children/adolescents:

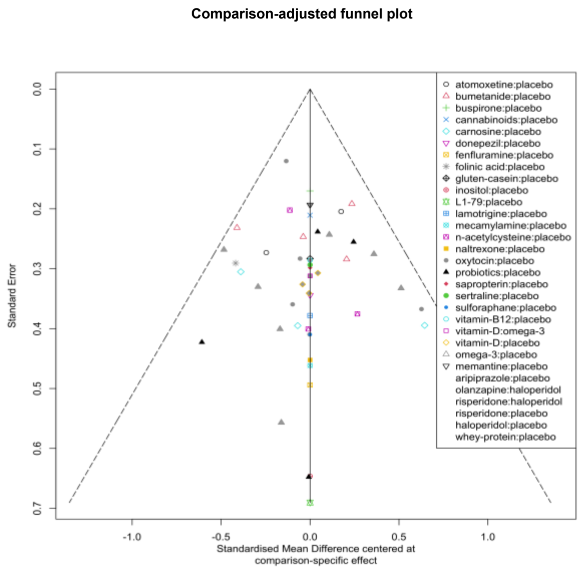

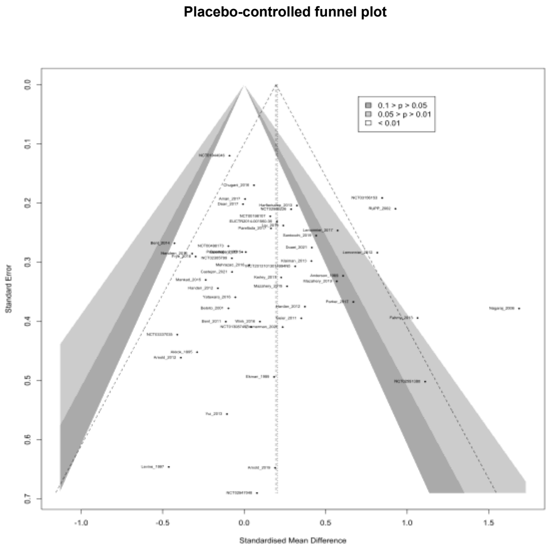

No clear asymmetry was observed in contour-enhanced funnel plot, and statistical tests was formally not significant (p>0.1)

There were less than 10 studies for adults or mixed populations.

6.8.4 Available data across studies and comparisons for the primary outcomes

6.8.4.1 Summary table of data per outcome and comparison

Reporting bias per comparison was also investigated by examining the proportion of studies with available data from the total published and unpublished studies. We expected that almost each trial reported usable data on core symptoms, therefore potential reporting bias was suspected when data were available in less than 75% of the total number of completed studies for a comparison (e.g. less than three out four studies). Neverthelss, we took into consideration the sample size of studies with not available data and the potential impact of their results on the meta-analytic estimates, i.e. for the comparison of balovaptan vs. placebo in adults and oxytocin vs. placebo in children/adolescents.

	comparison	n/n	study name	sample size	SMD 95% CI (>0 favors the first intervention of the comparison) SCI	RRBI	Overall core symptoms	% of studies with data for SCI	RRBI	Overall core symptoms
adults/mixed	agomelatine vs. placebo		Ballester_2015	23	n.i.	n.i.	n.i.	0%	0%	0%
	AZD7325 vs. placebo		NCT01966679	40	unpub.	unpub.	unpub.	0%	0%	0%
	balovaptan vs. placebo	1	Bolognani_2019	223	0.2246 [-0.0729; 0.5221]	0.0754 [-0.2213; 0.3721]	0.0111 [-0.2855; 0.3077]	100%	50% (41% of the participants, but the inclusion of the data of the missing trial may not alter the meta-analytic estimates, since the trial was reported as negative)	50% (41% of the participant, but the inclusion of the data of the missing trial may not alter the meta-analytic estimates, since the trial was reported as negative)
		2	NCT03504917	322	-0.1033 [-0.3745; 0.1678]	n.i.	n.i.			

citalopram vs. placebo		NCT00609531	12	n.i.	-0.2113 [-1.3476; 0.9250]	n.i.	0%	100%	0%
clomipramine vs. desipramine		Gordon_1993	30	crossover	crossover	crossover	0%	0%	0%
clomipramine vs. haloperidol		Remington_2001	37	crossover	crossover	crossover	0%	0%	0%
clomipramine vs. placebo	1	Gordon_1993	30	crossover	crossover	crossover	0%	0%	0%
	2	Remington_2001	37	crossover	crossover	crossover			
desipramine vs. placebo		Gordon_1993	30	crossover	crossover	crossover	0%	0%	0%
dextromethorphan/quinidine vs. placebo		Chez_2017	15	crossover	crossover	crossover	0%	0%	0%
fluoxetine vs. placebo		Hollander_2012	37	n.i.	1.2024 [0.4469; 1.9579]	n.i.	0%	100%	0%
fluvoxamine vs. placebo		McDougle_1996	30	n.i.	1.0382 [0.2691; 1.8074]	n.i.	0%	100%	0%
haloperidol vs. placebo		Remington_2001	37	crossover	crossover	crossover	0%	0%	0%
ketamine vs. placebo		Wink_2020	21	crossover	crossover	crossover	0%	0%	0%
milnacipran vs. placebo		Noone_2014	10	n.i.	-0.0441 [-1.2840; 1.1957]	n.i.	0%	100%	0%
oxytocin vs. placebo	1	Anagnostou_2012	19	n.i.	0.1242 [-0.7774; 1.0259]	0.2896 [-0.6169; 1.1960]	50%	75%	63%
	2	Bernaerts_2020	40	n.i.	0.5093 [-0.1347; 1.1534]	-0.0584 [-0.7529; 0.6361]			

		3	EUCTR2010-018740-NL	78 (antisocial or ASD)	unpub.	unpub.	unpub.			
		4	Kosaka_2016	60	0.3726 [-0.1685; 0.9138]	0.5742 [0.0270; 1.1214]	n.i.			
		5	Munesue_2016	29	-0.8267 [-1.5902; -0.0633]	0.1150 [-0.6140; 0.8440]	-0.0459 [-0.7743; 0.6826]			
		6	NCT01788072	70	unpub.	unpub.	unpub.			
		7	Watanabe_2015	20	0.7312 [-0.2314; 1.6939]	0.1991 [-0.7277; 1.1260]	-0.1801 [-1.1064; 0.7462]			
		8	Yamasue_2018	106	-0.1134 [-0.4999; 0.2732]	0.4660 [0.0743; 0.8577]	0.0243 [-0.3620; 0.4106]			
	propanolol vs. placebo		NCT02871349	69	unpub.	unpub.	unpub.	0%	0%	0%
	risperidone vs. placebo	1	Hellings_2006	40	crossover	crossover	crossover	0%	50%	50%
		2	McDougle_1998	31	n.i.	0.9735 [0.2091; 1.7379]	0.4886 [-0.2409; 1.2180]			
	sulforaphane vs. placebo	1	NCT02909959	48	-0.1033 [-0.3745; 0.1678]	0.0000 [-0.5658; 0.5658]	0.0196 [-0.5463; 0.5854]	100%	100%	100%
		2	Singh_2014	44	0.6194 [-0.0459; 1.2846]	0.4678 [-0.1909; 1.1264]	0.8555 [0.2046; 1.5064]			
[illegible]	acamprosate vs. placebo	1	NCT01813318	36	unpub.	unpub.	unpub.	0%	0%	0%

amantidine vs. placebo		King_2001	39	n.i.	n.i.	n.i.	0%	0%	0%
arbaclofen vs. placebo		VeenstraVanderWeele_2017	150	0.1559 [-0.1647; 0.4765]	-0.15 [-0.47; 0.15]	n.i.	100%	100%	0%
aripiprazole vs. risperidone	1	DeVane_2019	61	n.i.	n.i.	n.i.	33%	33%	0%
	2	Ghanizadeh_2014	59	0.1231 [-0.3879; 0.6340]	-0.0830 [-0.5936; 0.4276]	n.i.			
	3	Lamberti_2016	44	n.i.	n.i.	n.i.			
aripiprazole vs. placebo	1	Ichikawa_2017	92	0.0696 [-0.3393; 0.4786]	0.2047 [-0.2052; 0.6146]	n.i.	71%	71%	14%
	2	Marcus_2009	218	0.1131 [-0.2062; 0.4323]	0.3114 [-0.0266; 0.6494]	n.i.			
	3	NCT00198107	81	0.5789 [0.1253; 1.0326]	0.5985 [0.1413; 1.0556]	0.1600 [-0.2763; 0.5963]			
	4	NCT00468130	15	unpub.	unpub.	unpub.			
	5	NCT00870727	33	0.6568 [-0.0464; 1.3601]	0.5193 [-0.1763; 1.2150]	n.i.			
	6	NCT03487770	111	unpub.	unpub.	unpub.			
	7	Owen_2009	98	0.2206 [-0.1831; 0.6242]	0.8831 [0.4419; 1.3243]E	n.i.			
atomoxetine vs. placebo	1	Arnold_2006	16	crossover	crossover	crossover	60%	60%	40%
	2	Handen_2015	128	-0.0737 [-0.5638; 0.4165]	0.2552 [-0.2369; 0.7474]	n.i.			
	3	Harfterkamp_2013	97	0.0413 [-0.3568; 0.4393]	0.9425 [0.5220; 1.3629]	0.3237 [-0.0771; 0.7244]			
	4	Martsenkovska 2015	80	n.i.	n.i.	n.i.			
	5	NCT00498173	60	0.2062 [-0.3153; 0.7276]	0.1246 [-0.3958; 0.6451]	-0.0972 [-0.6324; 0.4380]			

atomoxetine vs. risperidone	1	Martsenkovska 2015	80	n.i.	n.i.	n.i.	0%	0%	0%
balovaptan vs. placebo	1	NCT02901431	308	-0.0451 [-0.3486; 0.2584]	n.i.	n.i.	100%	0%	0%
bumetanide vs. placebo	1	EUCTR2014-001560-35	92	0.0000 [-0.4527; 0.4527]	0.0807 [-0.3722; 0.5336]	0.2009 [-0.2529; 0.6548]	67%	67%	67%
	2	Lemonnier_2012	60	0.4240 [-0.1210; 0.9690]	0.5920 [0.0409; 1.1431]	0.8171 [0.2602; 1.3740]			
	3	Lemonnier_2017	88	0.4591 [-0.0565; 0.9746]	0.7720 [0.2475; 1.2964]	0.5733 [0.0899; 1.0566]			
	4	NCT03156153	120	-0.0428 [-0.4022; 0.3166]	0.1849 [-0.1753; 0.5450]	0.8468 [0.4711; 1.2224]			
	5	NCT03715153	211	unpub.	unpub.	unpub.			
	6	NCT03715166	211	unpub.	unpub.	unpub.			
buspirone vs. placebo	1	Chugani_2016	166	-0.0433 [-0.3768; 0.2901]	0.0461 [-0.2874; 0.3795]	0.0600 [-0.2735; 0.3935]	50%	50%	50%
	2	NCT00166621	20	unpub.	unpub.	unpub.			
cannabinoids vs. placebo		NCT02956226	150	n.i.	n.i.	0.2882 [-0.1246; 0.7009]	0%	0%	100%
carnosine vs. placebo	1	Fahmy_2013	30	n.i.	n.i.	1.0644 [0.2911; 1.8377]	50%	25%	75%
	2	Geier_2011	30	0.3523 [-0.5131; 1.2176]	n.i.	0.3505 [-0.4238; 1.1248]			
	3	Ghodsi_2018	44	n.i.	n.i.	n.i.			
	4	Mehrazad_2018	50	-0.0340 [-0.6320; 0.5640]	-0.0165 [-0.6145; 0.5814]	0.0306 [-0.5674; 0.6285]			
cholesterol vs. placebo	1	NCT00965068	15	0.3312 [-0.6925; 1.3549]	n.i.	n.i.	100%	0%	0%

citalopram vs. placebo		King_2009	149	0.0516 [-0.2696; 0.3729]	0.0334 [-0.2878; 0.3547]	n.i.	100%	100%	0%
D-cycloserine vs. placebo		NCT00198120	80	unpub.	unpub.	unpub.	0%	0%	0%
digestive enzymes vs. placebo	1	Munasinghe_2010	43	crossove r	crossove r	crossover	0%	0%	0%
	2	NCT00881452	182	unpub.	unpub.	unpub.			
	3	NCT02410902	335	unpub.	unpub.	unpub.			
dimethylglycine vs. placebo	1	Bolman_1999	10	crossove r	crossove r	crossover	50%	0%	0%
	2	Kern_2001	37	0.5125 [-0.1439; 1.1689]	n.i.	n.i.			
valproate vs. placebo	1	Hollander_2006	13	n.i.	1.3261 [-0.0005; 2.6528]	n.i.	0%	33%	0%
	2	Hollander_2010	27	n.i.	n.i.	n.i.			
	3	Hellings_2005	30	n.i.	n.i.	n.i.			
donepezil vs. placebo	1	Handen_2012	34	-0.2048 [-0.8802; 0.4706]	n.i.	-0.1618 [-0.8365; 0.5129]	100%	0%	50%
	2	Gabis_2019	60	-0.0731 [-0.6396; 0.4934]	n.i.	n.i.			
fenfluramine vs. placebo	1	August_1987	10	crossove r	crossove r	crossover	0%	0%	11%
	2	Barthelemy_1989	13	crossove r	crossove r	crossover			
	3	Campbell_1987	28	n.i.	n.i.	n.i.			
	4	Ekman_1989	20	crossove r	crossove r	0.1835 [-0.7849; 1.1520]			
	5	Leventhal_1993	15	crossove r	crossove r	crossover			
	6	Realmuto_1986	14	crossove r	crossove r	crossover			
	7	Sherman_1989	15	crossove r	crossove r	crossover			
	8	Stern_1990	20	crossove r	crossove r	crossover			

	9	Duker_1991	11	crossover	crossover	crossover			
ferrous vs. placebo		Reynold_2019	20	n.i.	n.i.	n.i.	0%	0%	0%
fluoxetine vs. placebo	1	Herscu_2019	158	n.i.	-0.1646 [-0.4800; 0.1509]	n.i.	25%	75%	0%
	2	Hollander_2005	44	n.i.	0.2165 [-0.4134; 0.8465]	n.i.			
	3	NCT00183339	18	n.i.	n.i.	n.i.			
	4	Reddihough_2019	146	0.0478 [-0.2768; 0.3724]	0.2969 [-0.0294; 0.6233]	n.i.			
fluvoxamine vs. placebo	1	McDougle_2000	34	unpub.	unpub.	unpub.	0%	0%	0%
	2	NCT00655174	108	unpub.	unpub.	unpub.			
	3	Sugie_2005	18	crossover	crossover	crossover			
fluvoxamine vs. setraline	1	NCT00655174	108	unpub.	unpub.	unpub.	0%	0%	0%
folinic acid vs. placebo	1	Frye_2018	48	0.2328 [-0.3356; 0.8011]	0.5007 [-0.0752; 1.0765]	-0.2984 [-0.8681; 0.2713]	66%	33%	66%
	2	NCT00672360	13	unpub.	unpub.	unpub.			
	3	NCT02551380	19	1.0426 [0.0681; 2.0171]	n.i.	1.1106 [0.1266; 2.0946]			
galantamine vs. placebo	1	NCT00252603	20	unpub.	unpub.	unpub.	0%	0%	0%
	2	Niederhofer_2002a	20	crossover	crossover	crossover			
gluten-casein vs. placebo		Pusponegoro_2015	74	n.i.	n.i.	0.0094 [-0.5454; 0.5642]	0%	0%	100%
guanfacine vs. placebo		Scahill_2015	62	0.0401 [-0.4580; 0.5383]	0.5450 [0.0371; 1.0529]	n.i.	100%	100%	0%

haloperidol vs. olanzapine		Malone_2001	12	n.i.	n.i.	-0.6316 [-1.8045; 0.5412]	0%	0%	100%
haloperidol vs. risperidone		Miral_2008	30	n.i.	n.i.	-0.6779 [-1.4449; 0.0891]	0%	0%	100%
haloperidol vs. placebo	1	Anderson_1989	45	crossover	crossover	0.6072 [-0.0264; 1.2408]	0%	0%	33%
	2	Campbell_1982	41	crossover	crossover	crossover			
	3	Cohen_1980	10	crossover	crossover	crossover			
IGOH vs. placebo		Handen_2009	125	n.i.	n.i.	n.i.	0%	0%	0%
inositol vs. placebo		Levine_1997	10	n.i.	n.i.	-0.4632 [-1.7299; 0.8035]	0%	0%	100%
L1-79 vs. placebo		NCT02947048	39	0.5036 [-0.8783; 1.8855]	0.8651 [-0.2470; 1.9772]	0.0783 [-1.2749; 1.4315]	100%	100%	100%
lamotrigine vs. placebo		Belsito_2001	37	-0.1181 [-0.9219; 0.6857]	-0.0364 [-0.8394; 0.7666]	-0.0968 [-0.8381; 0.6445]	100%	100%	100%
levetiracetam vs. placebo		Wasserman_2006	20	n.i.	n.i.	n.i.	0%	0%	0%
levodopa vs. placebo		Sugiyama_1998	20	crossover	crossover	crossover	0%	0%	0%
lofexidine vs. placebo		Niederhofer_2002b	12	crossover	crossover	crossover	0%	0%	0%
lurasidone vs. placebo		Loebel_2016	150	0.0812 [-0.2613; 0.4237]	-0.0609 [-0.4033; 0.2815]	n.i.	100%	100%	0%
mecamylamine vs. placebo		Arnold_2012	20	-0.3854 [-1.2899; 0.5191]	-0.0411 [-0.9358; 0.8536]	-0.3889 [-1.2935; 0.5158]	100%	100%	100%
melatonin vs. placebo	1	Cortesi_2012	160	n.i.	n.i.	n.i.	17%	17%	0%
	2	Gringras_2017	125	n.i.	n.i.	n.i.			

	3	Hayashi_2021	196	0.0619 [-0.2344; 0.3582]	0.0000 [-0.2962; 0.2962]	n.i.			
	4	NCT01993251	26	unpub.	unpub.	unpub.			
	5	Tordjman_2013	32	unpub.	unpub.	unpub.			
	6	Wright_2011	20	crossover	crossover	crossover			
melatonin+donepezil vs. placebo		NCT02487082	12	unpub.	unpub.	unpub.	0%	0%	0%
memantine vs. risperidone		Nikvarz_2017	34	0.09 [-0.62; 0.81]	-0.11 [-0.83; 0.61]	0.00 [-0.71; 0.72]	100%	100%	100%
memantine vs. placebo	1	Aman_2017	121	-0.1329 [-0.5123; 0.2465]	n.i.	0.0053 [-0.3736; 0.3843]	25%	0%	25%
	2	Hage_2016	50	unpub.	unpub.	unpub.			
	3	NCT01372449	23	n.i.	n.i.	n.i.			
	4	NCT01972074	43	n.i.	n.i.	n.i.			
methylphenidate vs. placebo	1	Ghuman_2009	12	crossover	crossover	crossover	0%	0%	0%
	2	Handen_2000	13	crossover	crossover	crossover			
	3	Pearson_2013	24	crossover	crossover	crossover			
	4	Quintana_1995	10	crossover	crossover	crossover			
	5	RUPP_2005	72	crossover	crossover	crossover			
mirtazapine vs. placebo		NCT01302964	30	n.i.	n.i.	n.i.	0%	0%	0%
multivitamin vs. placebo		NCT00572741	39	unpub.	unpub.	unpub.	0%	0%	0%
n-acetylcysteine vs. placebo	1	Dean_2017	102	-0.3035 [-0.7019; 0.0949]	0.0104 [-0.3856; 0.4065]	-0.0073 [-0.4034; 0.3888]	100%	100%	100%
	2	Hardan_2012	33	0.0452 [-0.6833; 0.7736]	0.4055 [-0.3314; 1.1425]	0.3724 [-0.3632; 1.1080]			

	3	Wink_2016	31	-0.1320 [-0.9176; 0.6536]	-0.0645 [-0.8494; 0.7204]	0.0963 [-0.6889; 0.8814]			
naltrexone vs. placebo	1	Akkok_1995	20	n.i.	n.i.	-0.2890 [-1.1756; 0.5976]	0%	0%	17%
	2	Bouvard_1995	10	crossover	crossover	crossover			
	3	Campbell_1993	45	n.i.	n.i.	n.i.			
	4	Kolmen_1997	11	crossover	crossover	crossover			
	5	Scifo_1991	12	crossover	crossover	crossover			
	6	Willemsen_1996	23	crossover	crossover	crossover			
olanzapine vs. placebo	1	Hollander_2006b	11	n.i.	n.i.	n.i.	0%	0%	0%
	2	Malone_2010	32	unpub.	unpub.	unpub.			
	3	NCT00057408	78	unpub.	unpub.	unpub.			
omega-3 vs vitamin-D		Mazahery_2019	117	0.3053 [-0.3062; 0.9168]	0.0000 [-0.6076; 0.6076]	0.3046 [-0.3068; 0.9161]	100%	100%	100%
omega-3 vs. placebo	1	Amminger_2007	13	0.1281 [-1.0212; 1.2775]	0.8074 [-0.4060; 2.0209]	n.i.	60%	57%	50%
	2	Bent_2011	27	0.1474 [-0.6385; 0.9332]	0.1938 [-0.5930; 0.9806]	-0.1130 [-0.8984; 0.6723]			
	3	Bent_2014	57	0.6187 [0.0863; 1.1512]	0.4612 [-0.0654; 0.9879]	-0.4284 [-0.9540; 0.0973]			
	4	Doaei_2021	54	0.1630 [-0.3718; 0.6977]	0.2559 [-0.2803; 0.7920]	0.4150 [-0.1250; 0.9549]			
	5	ISRCTN2023387	50	unpub.	unpub.	unpub.			
	6	Mankad_2015	38	-0.1954 [-0.8418; 0.4510]	-0.3896 [-1.0411; 0.2618]	-0.2366 [-0.8838; 0.4106]			
	7	Mazahery_2019	117	0.6478 [-0.0081; 1.3036]	-0.1908 [-0.8304; 0.4488]	0.5678 [-0.0840; 1.2195]			

	8	NCT00467818	17	n.i.	n.i.	n.i.			
	9	NCT01260961	132	unpub.	unpub.	unpub.			
	10	NCT02222285	56	unpub.	unpub.	unpub.			
	11	NCT03550209	72	n.i.	n.i.	n.i.			
	12	Parellada_2017	77	0.1524 [-0.3239; 0.6287]	0.1229 [-0.3532; 0.5989]	0.1642 [-0.3122; 0.6406]			
	13	Voigt_2014	48	-0.3763 [-1.1691; 0.4165]	n.i.	n.i.			
	14	Yui_2013	13	0.3942 [-0.7112; 1.4997]	0.1972 [-0.8970; 1.2914]	-0.1057 [-1.1973; 0.9858]			
ORG-2766 vs. placebo	1	Buietlaar_1990	14	crossover	crossover	crossover	0%	0%	0%
	2	Buitelaar_1992	20	crossover	crossover	crossover			
oxytocin vs. placebo	1	Guastella_2015	50	-0.0030 [-0.5578; 0.5518]	0.1591 [-0.3967; 0.7148]	-0.0126 [-0.5674; 0.5422]	71% (85.3% of the participants are included in the analysis, based also on a large well-powered clinical trial)	71% (88% of the participants are included in the analysis, based also on a large well-powered clinical trial)	71% (81% of the participants are included in the analysis, based also on a large well-powered clinical trial)
	2	NCT01308749	25	0.2866 [-0.5544; 1.1275]	n.i.	0.0414 [-0.7616; 0.8445]			
	3	NCT01908205	60	-0.0848 [-0.6199; 0.4504]	-0.0638 [-0.5989; 0.4712]	n.i.			
	4	NCT01944046	290	-0.0249 [-0.2994; 0.2495]	-0.0940 [-0.3296; 0.1417]	-0.0895 [-0.3251; 0.1462]			
	5	Parker_2017	35	0.1058 [-0.7592; 0.9708]	0.1350 [-0.5393; 0.8093]	0.6701 [-0.0500; 1.3902]			
	6	UMIN000009075	40	unpub.	unpub.	unpub.			

	7	Yatawara_2016	39	n.i.	-0.0133 [-0.7177; 0.6911]	-0.0545 [-0.7591; 0.6501]			
prednisolone vs. placebo		Vasconcelos_2014	40	n.i.	n.i.	n.i.	0%	0%	0%
probiotics vs. placebo	1	Arnold_2019	13	0.4459 [-0.8440; 1.7357]	0.0691 [-1.1967; 1.3348]	0.1896 [-1.0800; 1.4593]	83% (77% of the participants)	67%	67%
	2	Liu_2019	80	0.0932 [-0.3723; 0.5587]	0.0252 [-0.4401; 0.4904]	0.2399 [-0.2271; 0.7070]			
	3	NCT03337035	35	0.2215 [-0.5361; 0.9791]	-0.3493 [-1.1109; 0.4123]	-0.4134 [-1.1776; 0.3509]			
	4	NCT03369431	69	unpub.	unpub.	unpub.			
	5	Santocchi_2019	85	0.3106 [-0.1865; 0.8076]	0.0139 [-0.4800; 0.5078]	0.4418 [-0.0585; 0.9422]			
	6	Wang_2020	26	0.2228 [-1.0112; 1.4568]	n.i.	n.i.			
riluzole vs. placebo		NCT01661855	58	0.1471 [-0.3684; 0.6625]	n.i.	n.i.	100%	0%	0%
risperidone vs. placebo	1	Kent_2013	96	n.i.	n.i.	n.i.	29%	29%	29%
	2	Martsenkovska 2015	80	n.i.	n.i.	n.i.			
	3	Nagaraj_2006	40	n.i.	n.i.	1.6871 [0.9453; 2.4288]			
	4	NCT01171937	41	unpub.	unpub.	unpub.			
	5	NCT01624675	39	n.i.	n.i.	n.i.			
	6	RUPP_2002	101	0.4208 [0.0261; 0.8155]	0.7128 [0.3099; 1.1157]	0.9286 [0.5171; 1.3401]			
	7	Shea_2004	80	0.4477 [-0.0049; 0.9003]	0.4823 [0.0288; 0.9358]	n.i.			

sapropterin vs. placebo	1	Danfors_2005	12	crossover	crossover	crossover	50%	50%	50%
	2	Klaiman_2013	61	0.2123 [-0.3674; 0.7921]	0.3202 [-0.2618; 0.9022]	0.4127 [-0.1720; 0.9973]			
secretin vs. placebo		Ratliff_2005	15	crossover	crossover	crossover	0%	0%	0%
sertraline vs. placebo		NCT00655174	108	unpub.	unpub.	unpub.	50%	50%	50%
		NCT02385799	58	-0.0131 [-0.5933; 0.5670]	0.0523 [-0.5228; 0.6275]	-0.0758 [-0.6510; 0.4995]			
simvastatin vs. placebo		Stivaros_2018	30	-0.3039 [-1.0680; 0.4603]	-0.3022 [-1.0498; 0.4454]	n.i.	100%	100%	0%
sulforaphane vs. placebo	1	NCT02879110	110	unpub.	unpub.	unpub.	50%	50%	50%
	2	Zimmerman_2021	57	-0.1588 [-0.9605; 0.6428]	0.0369 [-0.7634; 0.8371]	0.2359 [-0.5675; 1.0394]			
tianeptine vs. placebo		Niederhofer_2003	13	0.1834 [-0.9517; 1.3186]	n.i.	n.i.	100%	0%	0%
tideglusib vs. placebo		NCT02586935	83	0.3786 [-0.0638; 0.8209]	0.3305 [-0.1109; 0.7719]	n.i.	100%	100%	0%
venlafaxine vs. placebo		Niederhofer_2004	14	crossover	crossover	crossover	0%	0%	0%
vitamin-B12 vs. placebo	1	Bertoglio_2010	30	crossover	crossover	crossover	50%	50%	50%
	2	Hendren_2016	57	0.1072 [-0.4494; 0.6638]	0.2184 [-0.3396; 0.7763]	-0.3196 [-0.8795; 0.2404]			
vitamin-B6 vs. placebo	1	Martineuaeu_1985	60	crossover	crossover	crossover	0%	0%	0%
	2	NCT01230359	40	unpub.	unpub.	unpub.			

	3	Findling_1997	12	crossover	crossover	crossover			
vitamin-D vs. placebo	1	IRCT20131013014994N5	52	0.0524 [-0.5457; 0.6505]	0.1058 [-0.4926; 0.7042]	0.27 [−0.1; 0.64]	60%	80%	60%
	2	Kerley_2017	42	-0.0484 [-0.6852; 0.5885]	-0.3491 [-0.9912; 0.2931]	0.2271 [-0.4120; 0.8661]			
	3	Mazahery_2019	117	0.3421 [-0.3284; 1.0125]	-0.1903 [-0.8570; 0.4764]	0.2629 [-0.4053; 0.9312]			
	4	Moradi_2018	100	n.i.	0.8038 [0.2257; 1.3820]	n.i.			
	5	NCT02550912	42	unpub.	unpub.	unpub.			
whey-protein vs. placebo		Castejon_2021	81	0.2132 [-0.4093; 0.8357]	n.i.	-0.0744 [-0.6952; 0.5464]	100%	0%	100%

6.9. Grading the confidence in evidence with CINeMA

6.9.1 General

We evaluated confidence in the evidence of network meta-analytic estimates of placebo-controlled comparisons using Confidence In Network Meta-Analysis (CINeMA) framework [1] and the online tool (https://cinema.ispm.unibe.ch/)

6.9.1.1 Domains of CINeMA

i. Within-study bias

Within-study bias was assessed using the Cochrane's Risk-of-bias tool [2] and an overall judgment of risk of bias was assigned to each study, i.e., 'low, 'moderate' and 'high' risk [3] (see eAppendix-5.2). The contribution of each study to each effect size was estimated in CINeMA and a contribution matrix was constructed. Within-study bias for each comparison was classified into 'no concerns', 'some concerns' and 'major concerns' based on the average overall risk of bias according to the contribution matrix.

ii. Reporting bias

Our search was comprehensive and funnel plot analysis suggested small-study effects for social-communication dififculties in children/adolescnets, yet funnel plots for other outcomes were inconclusive (see eAppendix-6.8.1, 6.8.2, 6.8.3). In addition, there was no single comparison with at least 10 studies in order to perform a funnel plot analysis for a specific comparison. Therefore, we further assessed reporting bias for a comparison by examining the percentage of studies with sufficient data. A comparison was judged with 'suspected' reporting bias when less than 75% of available studies report sufficient data for this outcome, otherwise reporting bias was 'unsuspected". Neverthelss, we also took into consideration the sample size of missing studies and the potential impact of their results (in case they were impartially reported) on the meta-analytic estimates, i.e., for the comparison of balovaptan vs. placebo in adults and oxytocin vs. placebo in children/adolescents. See eAppendix-6.8 for the decision on reporting bias for each comparison.

iii. Indirectness

We assigned 'moderate' levels of indirectness to studies focused on a specific subgroup of patients by utilizing inclusion/exclusion criteria for an associated condition (e.g. irritability, ADHD symptoms, sleep disorders, intolerance or non-response to previous psychopharmacological treatments), a genetic syndrome (e.g. NF1), intellectual disability or high-functioning participants (see study characteristics in eAppendix-5.1.2). The rest of the studies were rated with 'low' levels of indirectness. There was no apparent reason to of indirectness due to interventions or outcomes. A comparison was judged with 'no concerns' or 'some concerns' due to indirectness based on the average indirectness of the contributions of each study to the comparison.

iv. Imprecision

Since the majority of the interventions were examined in one or two trials (usually with a small sample size), the available evidence was often based on a small number of participants. We set the optimal information size (OIS) at 200 participants, since an assumed trial with equal randomization would have been powered (80%) to detect a small to medium effect size of SMD=0.4 (see figure below) with an alpha of 0.05. Nevertheless, larger sample sizes are required to detect smaller effect sizes, i.e. 350 participants are required for SMD=0.3 and 786 for SMD=0.2, while smaller sample sizes are required for larger effect sizes, i.e., 90 participants treated with an intervention for SMD=0.6 and 130 for SMD=0.5. Given that small-to-medium effect sizes might be expected for the core symptoms of ASD, we set the threshold of sufficient evidence at 200 participants.

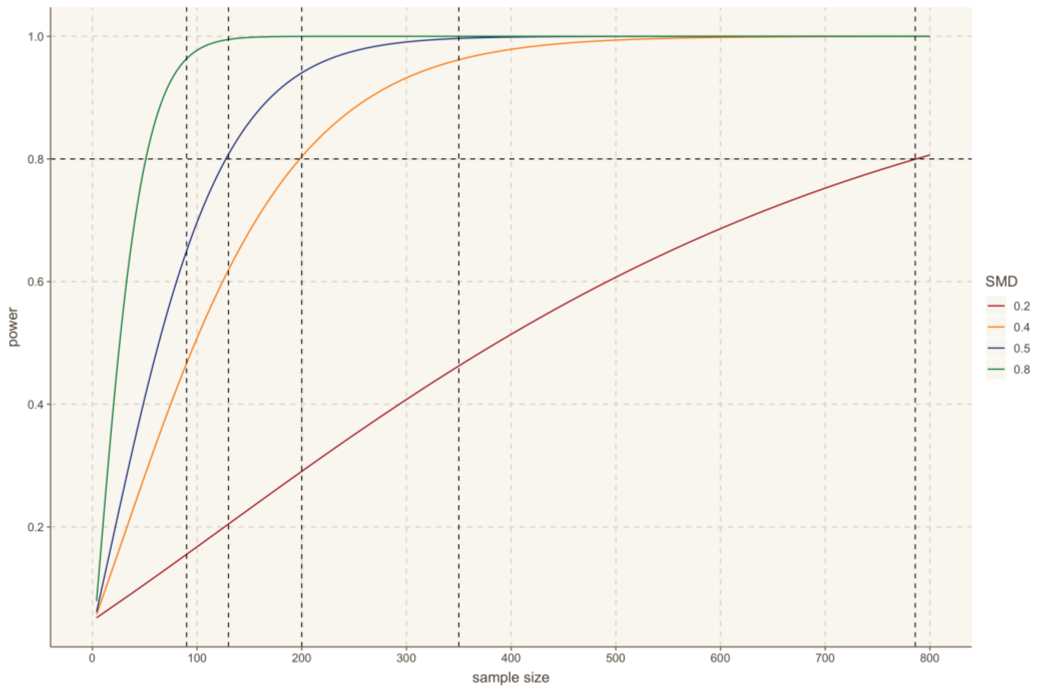

SMD: standardized mean difference, power: statistical power to detect an effect size at alpha 0.05

We set as clinically meaningful threshold of SMD the range between -0.21 and 0.21 (area of equivalence), since effect sizes within this range are often considered trivial. Imprecision was evaluated based on the relation between the sample size (number of participants received the medication in the network meta-analysis and number of participants received placebo in the studies of the direct comparison) and the OIS [4], and between the 95% confidence intervals of the effect size and the clinically meaningful threshold according to the rules described in CINeMA documentation [1].

Imprecision of a comparison could be classified as ‘no concerns’, ‘some concerns’ and ‘major concerns’: 1) “no concerns” when the OIS was met, and the 95%CI were within the area of equivalence or did not cross the effect line, 2) “some concerns” when the OIS was not met and/or the 95%CI crossed the null effect line but did not cross beyond the area of equivalence, 3) “major concerns” when the 95%CI crossed beyond the area of equivalence to both sides around the null effect line (irrespective if the OIS was met or not).

v. Heterogeneity

Heterogeneity was evaluated based on the relation of both the 95% confidence intervals and the 95% prediction intervals with the clinically meaningful threshold defined above in the domain of imprecision. The rules described in CINeMA documentation were used [1]. Since our sample was mainly consisted of a few small studies per comparison, we further assessed heterogeneity by examining the τ^2 of the pairwise comparisons, when there were more than two studies for the comparison. Similar to the assessment of heterogeneity in eAppendix-6.7, we compared τ^2 with its empirical distribution, and the magnitude of heterogeneity was classified as low, moderate or high levels. In comparisons with at least two studies, when the assessment according to prediction intervals and τ^2 did not agree, we used the assessment based on τ^2. Accordingly, comparisons could be classified according to heterogeneity evaluation as ‘no concerns’, ‘some concerns’, ‘major concerns.

vi. Incoherence

Incoherence was evaluated using a design-by-treatment test (for comparisons with only direct or indirect evidence) and the SIDE approach (when both direct and indirect evidence was available) according to CINeMA documentation [1]. Comparisons could be classified according to

incoherence evaluation as ‘no concerns’, ‘some concerns’, ‘major concerns’. When there was only indirect evidence, incoherence was classified as ‘some concerns’. In case there were no closed loops and incoherence could not be evaluated, we rated incoherence as “no concerns” in contrast to CINeMA documentation, since we evaluated evidence on placebo-controlled comparisons, for which there was direct evidence.

6.9.1.2 Summarizing judgments across the domains

Judgments for each domain were summarized in an overall judgment on the confidence in the NMA estimate. The confidence was classified as ‘very low’, ‘low’, ‘moderate’ and ‘high’, and starting from ‘high’, it could be downgraded by one level for ‘some concerns’ (or ‘suspected bias’) and two levels for ‘major concerns’ in a domain. We jointly examined the domains imprecision and heterogeneity, as well as incoherence, since they are interconnected [5]. Therefore, the confidence could have been downgraded by up to 1) two levels for within-study bias, 2) one level for reporting bias, 3) one level for indirectness, 4) two levels for the common domain of imprecision-heterogeneity/incoherence. The latter was rated as below:

Judgment of the joint domain of heterogeneity, imprecision and incoherence	Number of subdomains with ‘major concerns’	Number of subdomains with ‘some concerns’	Number of subdomains with ‘no concerns’
‘major concerns’	2	1	0
‘major concerns’	2	0	1
‘major concerns’	1	1	1
‘major concerns’	1	2	0
‘some concerns’	1	0	2
‘some concerns’	0	3	0
‘some concerns’	0	2	1
‘some concerns’	0	1	2
‘no concerns’	0	0	3

6.9.2 CINeMA for each primary outcome

In the network plots below, the color of the edge represents the average overall risk of bias and the color of the node the distribution of indirectness. Low risk of bias or low levels of indirectness is represented with green color, unclear risk of bias or moderate levels of indirectness with yellow and high risk of bias with red.

6.9.2.1 Social-communication difficulties

6.9.2.1.1 Children/adolescents

a. Network plot

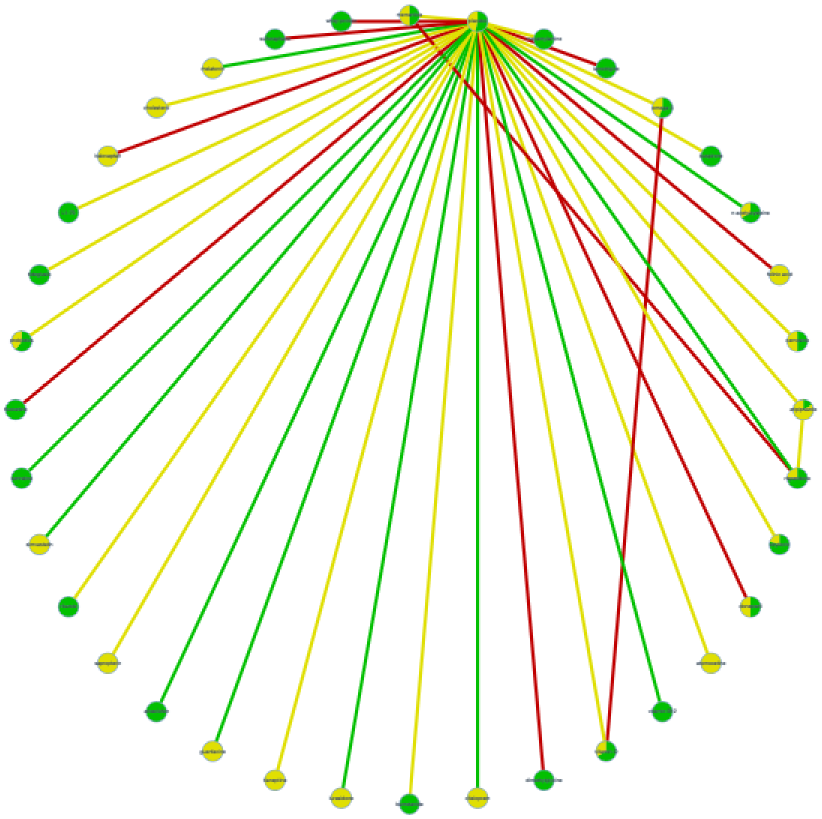

b. Confidence in evidence

Drug	Within-study bias	Reporting bias	Indirectness	Imprecision	Heterogeneity	Incoherence	Confidence
arbaclofen	No concerns	Undetected	No concerns	Some concerns	No concerns	No concerns	Moderate
aripiprazole	No concerns	Suspected	Some concerns	No concerns	No concerns	No concerns	Low
atomoxetine	Some concerns	Suspected	Some concerns	Major concerns	No concerns	No concerns	Very Low
balovaptan	Major concerns	Undetected	Some concerns	Major concerns	No concerns	No concerns	Very Low
bumetanide	No concerns	Suspected	No concerns	Some concerns	Some concerns	No concerns	Low
buspirone	Some concerns	Suspected	No concerns	Major concerns	No concerns	No concerns	Very Low
carnosine	Some concerns	Suspected	Some concerns	Major concerns	No concerns	No concerns	Very Low
cholesterol	Some concerns	Undetected	Some concerns	Major concerns	No concerns	No concerns	Very Low
citalopram	No concerns	Undetected	Some concerns	Major concerns	No concerns	No concerns	Low
dimethylglycine	Major concerns	Suspected	No concerns	Some concerns	No concerns	No concerns	Very Low
donepezil	Major concerns	Undetected	No concerns	Major concerns	No concerns	No concerns	Very Low

fluoxetine	Major concerns	Suspected	No concerns	Major concerns	No concerns	No concerns	Very Low
folinic acid	Some concerns	Suspected	Some concerns	Some concerns	Major concerns	No concerns	Very Low
guanfacine	No concerns	Undetected	Some concerns	Major concerns	No concerns	No concerns	Low
L1-79	Some concerns	Undetected	No concerns	Major concerns	No concerns	No concerns	Low
lamotrigine	Major concerns	Undetected	No concerns	Major concerns	No concerns	No concerns	Very Low
lurasidone	No concerns	Undetected	Some concerns	Major concerns	No concerns	No concerns	Low
mecamylamine	Some concerns	Undetected	No concerns	Major concerns	No concerns	No concerns	Low
melatonin	No concerns	Suspected	Some concerns	Major concerns	No concerns	No concerns	Very Low
memantine	Some concerns	Suspected	Some concerns	Major concerns	No concerns	No concerns	Very Low
n-acetylcysteine	No concerns	Undetected	No concerns	Some concerns	No concerns	No concerns	Moderate
omega-3	Some concerns	Suspected	No concerns	Some concerns	No concerns	No concerns	Very Low
oxytocin	No concerns	Undetected	No concerns	Some concerns	No concerns	No concerns	Moderate
probiotics	Some concerns	Undetected	No concerns	Some concerns	No concerns	No concerns	Low
riluzole	Some concerns	Undetected	No concerns	Major concerns	No concerns	No concerns	Low
risperidone	No concerns	Suspected	Some concerns	No concerns	No concerns	No concerns	Low
sapropterin	Some concerns	Suspected	Some concerns	Major concerns	No concerns	No concerns	Very Low
sertraline	No concerns	Suspected	No concerns	Major concerns	No concerns	No concerns	Low
simvastatin	No concerns	Undetected	Some concerns	Major concerns	No concerns	No concerns	Low
sulforaphane	Major concerns	Suspected	No concerns	Major concerns	No concerns	No concerns	Very Low
tianeptine	Some concerns	Undetected	Some concerns	Major concerns	No concerns	No concerns	Very Low
tideglusib	Some concerns	Undetected	No concerns	Some concerns	No concerns	No concerns	Low
vitamin-B12	No concerns	Suspected	No concerns	Major concerns	No concerns	No concerns	Low
vitamin-D	Some concerns	Suspected	No concerns	Major concerns	No concerns	No concerns	Very Low
whey-protein	Major concerns	Undetected	No concerns	Major concerns	No concerns	No concerns	Very Low

6.9.2.1.2 Adults or mixed

a. Network plot

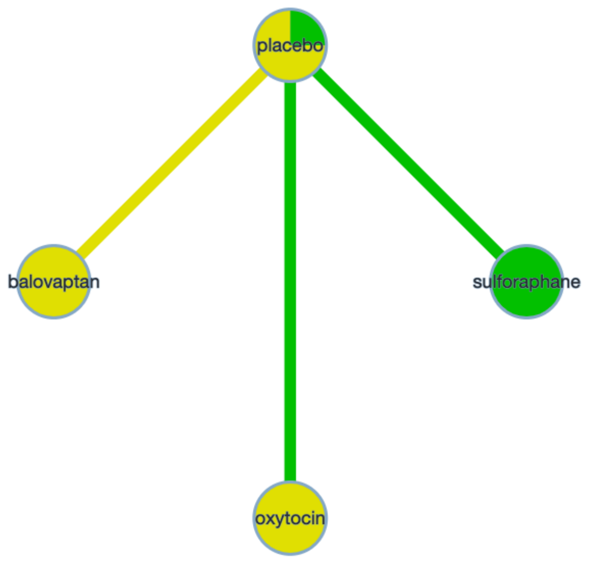

b. Confidence in evidence

Drug	Within-study bias	Reporting bias	Indirectness	Imprecision	Heterogeneity	Incoherence	Confidence
balovaptan	Some concerns	Undetected	Some concerns	Major concerns	Some concerns	No concerns	Very Low
oxytocin	No concerns	Suspected	Some concerns	Major concerns	Major concerns	No concerns	Very Low
sulforaphane	No concerns	Undetected	No concerns	Major concerns	Major concerns	No concerns	Low

6.9.2.2 Repetitive behaviors and restricted interests

6.9.2.2.1 Children/adolescents

a. Network plot

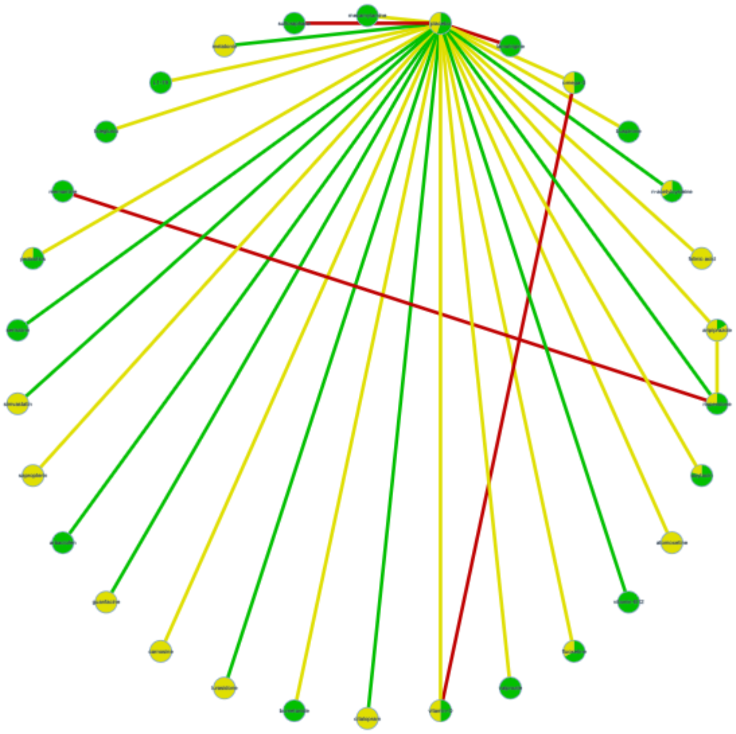

b. Confidence in evidence

Drug	Within-study bias	Reporting bias	Indirectness	Imprecision	Heterogeneity	Incoherence	Confidence
arbaclofen	No concerns	Undetected	No concerns	Major concerns	No concerns	No concerns	Moderate
aripiprazole	Some concerns	Suspected	Some concerns	No concerns	Some concerns	No concerns	Very Low
atomoxetine	Some concerns	Suspected	Some concerns	No concerns	Major concerns	No concerns	Very Low
bumetanide	No concerns	Suspected	No concerns	No concerns	Some concerns	No concerns	Low
buspirone	Some concerns	Suspected	No concerns	Major concerns	No concerns	No concerns	Very Low
carnosine	Some concerns	Suspected	Some concerns	Major concerns	No concerns	No concerns	Very Low
citalopram	No concerns	Undetected	Some concerns	Major concerns	No concerns	No concerns	Low
fluoxetine	Some concerns	Undetected	No concerns	Some concerns	Some concerns	No concerns	Low
folinic acid	Some concerns	Suspected	Some concerns	Some concerns	No concerns	No concerns	Very Low
guanfacine	No concerns	Undetected	Some concerns	Some concerns	No concerns	No concerns	Low
L1-79	Some concerns	Undetected	No concerns	Major concerns	No concerns	No concerns	Low
lamotrigine	Major concerns	Undetected	No concerns	Major concerns	No concerns	No concerns	Very Low
lurasidone	No concerns	Undetected	Some concerns	Major concerns	No concerns	No concerns	Low

mecamylamine	Some concerns	Undetected	No concerns	Major concerns	No concerns	No concerns	Low
melatonin	No concerns	Suspected	Some concerns	Major concerns	No concerns	No concerns	Very Low
memantine	Some concerns	Suspected	No concerns	Major concerns	No concerns	No concerns	Very Low
n-acetylcysteine	No concerns	Undetected	No concerns	Major concerns	No concerns	No concerns	Moderate
omega-3	Some concerns	Suspected	No concerns	Some concerns	No concerns	No concerns	Very Low
oxytocin	No concerns	Undetected	No concerns	Major concerns	No concerns	No concerns	Moderate
probiotics	Some concerns	Suspected	No concerns	Major concerns	No concerns	No concerns	Very Low
risperidone	No concerns	Suspected	Some concerns	No concerns	No concerns	No concerns	Low
sapropterin	Some concerns	Suspected	Some concerns	Major concerns	No concerns	No concerns	Very Low
sertraline	No concerns	Suspected	No concerns	Major concerns	No concerns	No concerns	Low
simvastatin	No concerns	Undetected	Some concerns	Major concerns	No concerns	No concerns	Low
sulforaphane	Major concerns	Suspected	No concerns	Major concerns	No concerns	No concerns	Very Low
tideglusib	Some concerns	Undetected	No concerns	Some concerns	Some concerns	No concerns	Low
valproate	Some concerns	Suspected	No concerns	Some concerns	No concerns	No concerns	Very Low
vitamin-B12	No concerns	Suspected	No concerns	Major concerns	No concerns	No concerns	Low
vitamin-D	Some concerns	Undetected	No concerns	Some concerns	Major concerns	No concerns	Very Low

6.9.2.2.2 Adults or mixed

a. Network plot

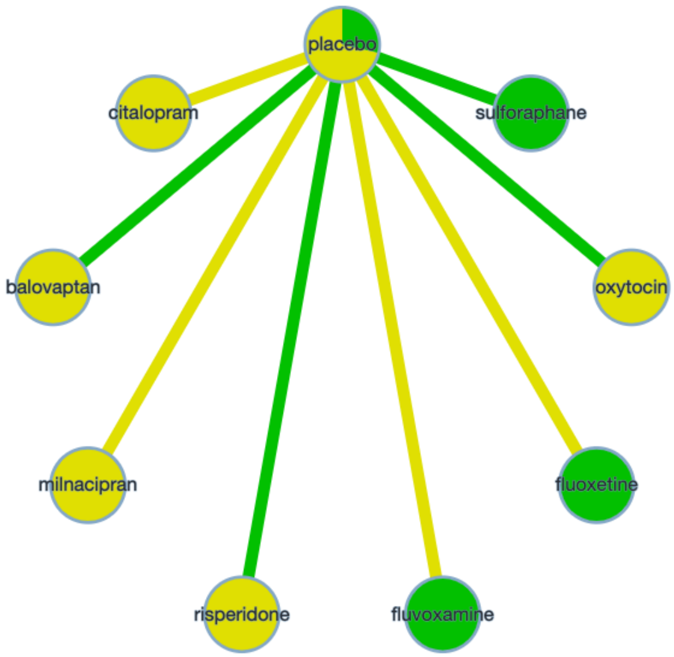

b. Confidence in evidence

Drug	Within-study bias	Reporting bias	Indirectness	Imprecision	Heterogeneity	Incoherence	Confidence
balovaptan	No concerns	Undetected	Some concerns	Major concerns	No concerns	No concerns	Low
citalopram	Some concerns	Undetected	Some concerns	Major concerns	No concerns	No concerns	Very Low
fluoxetine	Some concerns	Undetected	No concerns	Some concerns	No concerns	No concerns	Low
fluvoxamine	Some concerns	Undetected	No concerns	Some concerns	No concerns	No concerns	Low
milnacipran	Some concerns	Undetected	Some concerns	Major concerns	No concerns	No concerns	Very Low
oxytocin	No concerns	Undetected	Some concerns	No concerns	No concerns	No concerns	Moderate
risperidone	No concerns	Suspected	Some concerns	Some concerns	Some concerns	No concerns	Very Low
sulforaphane	No concerns	Undetected	No concerns	Major concerns	Some concerns	No concerns	Low

6.9.2.3 Overall core symptoms

6.9.2.3.1 Children/adolescents

a. Network plot

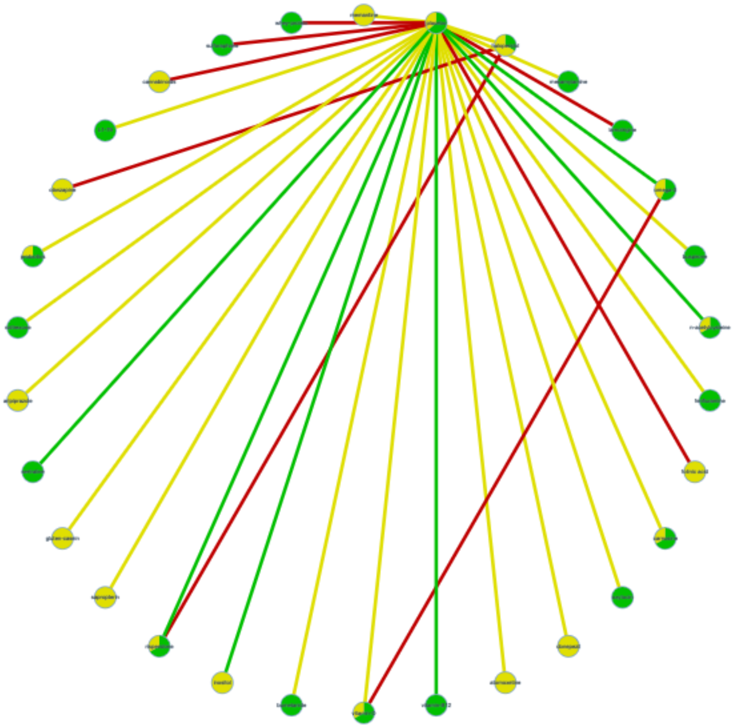

b. Confidence in evidence

Drug	Within-Study bias	Reporting bias	Indirectness	Imprecision	Heterogeneity	Incoherence	Confidence
L1-79	Some concerns	Undetected	No concerns	Major concerns	No concerns	No concerns	Low
aripiprazole	Some concerns	Suspected	Some concerns	Major concerns	No concerns	No concerns	Very Low

atomoxetine	Some concerns	Suspected	Some concerns	Major concerns	Some concerns	No concerns	Very Low
bumetanide	No concerns	Suspected	No concerns	No concerns	Some concerns	No concerns	Low
buspirone	Some concerns	Suspected	No concerns	Major concerns	No concerns	No concerns	Very Low
cannabinoids	Major concerns	Undetected	Some concerns	Major concerns	No concerns	No concerns	Very Low
carnosine	Some concerns	Undetected	No concerns	Some concerns	Major concerns	No concerns	Very Low
donepezil	Some concerns	Suspected	Some concerns	Major concerns	No concerns	No concerns	Very Low
fenfluramine	Some concerns	Suspected	No concerns	Major concerns	No concerns	No concerns	Very Low
folinic acid	Some concerns	Suspected	Some concerns	Major concerns	Major concerns	No concerns	Very Low
gluten-casein	Some concerns	Undetected	Some concerns	Major concerns	No concerns	No concerns	Very Low
haloperidol	Some concerns	Suspected	Some concerns	Some concerns	No concerns	No concerns	Very Low
inositol	No concerns	Undetected	Some concerns	Major concerns	No concerns	No concerns	Low
lamotrigine	Major concerns	Undetected	No concerns	Major concerns	No concerns	No concerns	Very Low
mecamylamine	Some concerns	Undetected	No concerns	Major concerns	No concerns	No concerns	Low
memantine	Some concerns	Suspected	Some concerns	Major concerns	No concerns	No concerns	Very Low
n-acetylcysteine	No concerns	Undetected	No concerns	Major concerns	No concerns	No concerns	Moderate
naltrexone	Some concerns	Suspected	No concerns	Major concerns	No concerns	No concerns	Very Low
omega-3	Some concerns	Suspected	No concerns	Some concerns	Some concerns	No concerns	Very Low
oxytocin	No concerns	Undetected	No concerns	Major concerns	No concerns	No concerns	Moderate
probiotics	Some concerns	Suspected	No concerns	Some concerns	No concerns	No concerns	Very Low
risperidone	No concerns	Suspected	Some concerns	Some concerns	Major concerns	No concerns	Very Low
sapropterin	Some concerns	Suspected	Some concerns	Major concerns	No concerns	No concerns	Very Low
sertraline	No concerns	Suspected	No concerns	Major concerns	No concerns	No concerns	Low
sulforaphane	Major concerns	Suspected	No concerns	Major concerns	No concerns	No concerns	Very Low
vitamin-B12	No concerns	Suspected	No concerns	Major concerns	No concerns	No concerns	Low
vitamin-D	Some concerns	Suspected	No concerns	Major concerns	No concerns	No concerns	Very Low
whey-protein	Major concerns	Undetected	No concerns	Major concerns	No concerns	No concerns	Very Low
olanzapine	Some concerns	Suspected	Some concerns	Some concerns	Some concerns	Some concerns	Very Low

6.9.2.3.2 Adults or mixed

a. Network plot

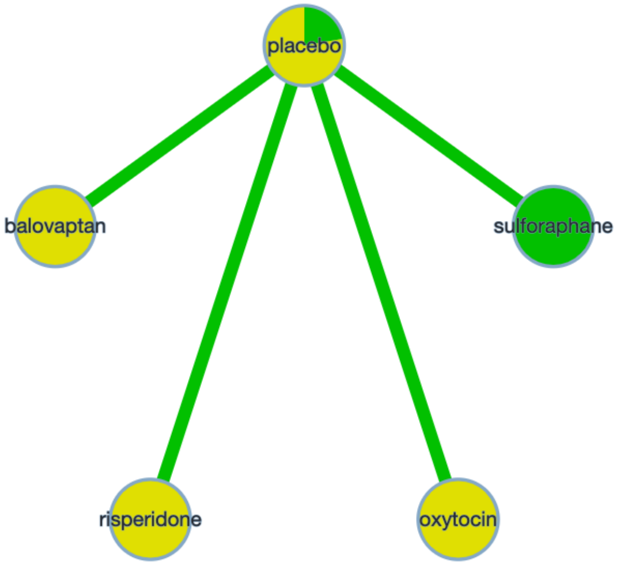

b. Confidence in evidence

Drug	Within-study bias	Reporting bias	Indirectness	Imprecision	Heterogeneity	Incoherence	Confidence
balovaptan	No concerns	Undetected	Some concerns	Major concerns	No concerns	No concerns	Low
oxytocin	No concerns	Suspected	Some concerns	Major concerns	No concerns	No concerns	Very Low
risperidone	No concerns	Suspected	Some concerns	Major concerns	No concerns	No concerns	Very Low
sulforaphane	No concerns	Undetected	No concerns	Some concerns	Major concerns	No concerns	Low

6.9.3 References

1. Nikolakopoulou A, Higgins JPT, Papakonstantinou T, Chaimani A, Del Giovane C, Egger M, et al. CINeMA: An approach for assessing confidence in the results of a network meta-analysis. PLOS Medicine. 2020;17(4):e1003082. doi: 10.1371/journal.pmed.1003082.
2. Higgins JPT, Altman DG, Gøtzsche PC, Jüni P, Moher D, Oxman AD, et al. The Cochrane Collaboration's tool for assessing risk of bias in randomised trials. Bmj. 2011;343:d5928.
3. Furukawa TA, Salanti G, Atkinson LZ, Leucht S, Ruhe HG, Turner EH, et al. Comparative efficacy and acceptability of first-generation and second-generation antidepressants in the acute treatment of major depression: protocol for a network meta-analysis. BMJ Open. 2016;6(7):e010919. doi: 10.1136/bmjopen-2015-010919.
4. Guyatt G, Oxman AD, Akl EA, Kunz R, Vist G, Brozek J, et al. GRADE guidelines: 1. Introduction—GRADE evidence profiles and summary of findings tables. J Clin Epidemiol. 2011;64(4):383-94.
5. Veroniki AA, Vasiliadis HS, Higgins JP, Salanti G. Evaluation of inconsistency in networks of interventions. Int J Epidemiol. 2013;42(1):332-45. doi: 10.1093/ije/dys222.

1. Nikolakopoulou A, Higgins JPT, Papakonstantinou T, Chaimani A, Del Giovane C, Egger M, et al. CINeMA: An approach for assessing confidence in the results of a network meta-analysis. PLOS Medicine. 2020;17(4):e1003082. doi: 10.1371/journal.pmed.1003082.
2. Higgins JPT, Altman DG, Gøtzsche PC, Jüni P, Moher D, Oxman AD, et al. The Cochrane Collaboration's tool for assessing risk of bias in randomised trials. Bmj. 2011;343:d5928.
3. Furukawa TA, Salanti G, Atkinson LZ, Leucht S, Ruhe HG, Turner EH, et al. Comparative efficacy and acceptability of first-generation and second-generation antidepressants in the acute treatment of major depression: protocol for a network meta-analysis. BMJ Open. 2016;6(7):e010919. doi: 10.1136/bmjopen-2015-010919.
4. Guyatt G, Oxman AD, Akl EA, Kunz R, Vist G, Brozek J, et al. GRADE guidelines: 1. Introduction—GRADE evidence profiles and summary of findings tables. J Clin Epidemiol. 2011;64(4):383-94.
5. Veroniki AA, Vasiliadis HS, Higgins JP, Salanti G. Evaluation of inconsistency in networks of interventions. Int J Epidemiol. 2013;42(1):332-45. doi: 10.1093/ije/dys222.

1. Nikolakopoulou A, Higgins JPT, Papakonstantinou T, Chaimani A, Del Giovane C, Egger M, et al. CINeMA: An approach for assessing confidence in the results of a network meta-analysis. PLOS Medicine. 2020;17(4):e1003082. doi: 10.1371/journal.pmed.1003082.
2. Higgins JPT, Altman DG, Gøtzsche PC, Jüni P, Moher D, Oxman AD, et al. The Cochrane Collaboration's tool for assessing risk of bias in randomised trials. Bmj. 2011;343:d5928.
3. Furukawa TA, Salanti G, Atkinson LZ, Leucht S, Ruhe HG, Turner EH, et al. Comparative efficacy and acceptability of first-generation and second-generation antidepressants in the acute treatment of major depression: protocol for a network meta-analysis. BMJ Open. 2016;6(7):e010919. doi: 10.1136/bmjopen-2015-010919.
4. Guyatt G, Oxman AD, Akl EA, Kunz R, Vist G, Brozek J, et al. GRADE guidelines: 1. Introduction—GRADE evidence profiles and summary of findings tables. J Clin Epidemiol. 2011;64(4):383-94.
5. Veroniki AA, Vasiliadis HS, Higgins JP, Salanti G. Evaluation of inconsistency in networks of interventions. Int J Epidemiol. 2013;42(1):332-45. doi: 10.1093/ije/dys222.

1. Nikolakopoulou A, Higgins JPT, Papakonstantinou T, Chaimani A, Del Giovane C, Egger M, et al. CINeMA: An approach for assessing confidence in the results of a network meta-analysis. PLOS Medicine. 2020;17(4):e1003082. doi: 10.1371/journal.pmed.1003082.
2. Higgins JPT, Altman DG, Gøtzsche PC, Jüni P, Moher D, Oxman AD, et al. The Cochrane Collaboration's tool for assessing risk of bias in randomised trials. Bmj. 2011;343:d5928.
3. Furukawa TA, Salanti G, Atkinson LZ, Leucht S, Ruhe HG, Turner EH, et al. Comparative efficacy and acceptability of first-generation and second-generation antidepressants in the acute treatment of major depression: protocol for a network meta-analysis. BMJ Open. 2016;6(7):e010919. doi: 10.1136/bmjopen-2015-010919.
4. Guyatt G, Oxman AD, Akl EA, Kunz R, Vist G, Brozek J, et al. GRADE guidelines: 1. Introduction—GRADE evidence profiles and summary of findings tables. J Clin Epidemiol. 2011;64(4):383-94.
5. Veroniki AA, Vasiliadis HS, Higgins JP, Salanti G. Evaluation of inconsistency in networks of interventions. Int J Epidemiol. 2013;42(1):332-45. doi: 10.1093/ije/dys222.

1. Nikolakopoulou A, Higgins JPT, Papakonstantinou T, Chaimani A, Del Giovane C, Egger M, et al. CINeMA: An approach for assessing confidence in the results of a network meta-analysis. PLOS Medicine. 2020;17(4):e1003082. doi: 10.1371/journal.pmed.1003082.
2. Higgins JPT, Altman DG, Gøtzsche PC, Jüni P, Moher D, Oxman AD, et al. The Cochrane Collaboration's tool for assessing risk of bias in randomised trials. Bmj. 2011;343:d5928.
3. Furukawa TA, Salanti G, Atkinson LZ, Leucht S, Ruhe HG, Turner EH, et al. Comparative efficacy and acceptability of first-generation and second-generation antidepressants in the acute

treatment of major depression: protocol for a network meta-analysis. BMJ Open. 2016;6(7):e010919. doi: 10.1136/bmjopen-2015-010919.
4. Guyatt G, Oxman AD, Akl EA, Kunz R, Vist G, Brozek J, et al. GRADE guidelines: 1. Introduction—GRADE evidence profiles and summary of findings tables. J Clin Epidemiol. 2011;64(4):383-94.
5. Veroniki AA, Vasiliadis HS, Higgins JP, Salanti G. Evaluation of inconsistency in networks of interventions. Int J Epidemiol. 2013;42(1):332-45. doi: 10.1093/ije/dys222.

1. Nikolakopoulou A, Higgins JPT, Papakonstantinou T, Chaimani A, Del Giovane C, Egger M, et al. CINeMA: An approach for assessing confidence in the results of a network meta-analysis. PLOS Medicine. 2020;17(4):e1003082. doi: 10.1371/journal.pmed.1003082.
2. Higgins JPT, Altman DG, Gøtzsche PC, Jüni P, Moher D, Oxman AD, et al. The Cochrane Collaboration's tool for assessing risk of bias in randomised trials. Bmj. 2011;343:d5928.
3. Furukawa TA, Salanti G, Atkinson LZ, Leucht S, Ruhe HG, Turner EH, et al. Comparative efficacy and acceptability of first-generation and second-generation antidepressants in the acute treatment of major depression: protocol for a network meta-analysis. BMJ Open. 2016;6(7):e010919. doi: 10.1136/bmjopen-2015-010919.
4. Guyatt G, Oxman AD, Akl EA, Kunz R, Vist G, Brozek J, et al. GRADE guidelines: 1. Introduction—GRADE evidence profiles and summary of findings tables. J Clin Epidemiol. 2011;64(4):383-94.
5. Veroniki AA, Vasiliadis HS, Higgins JP, Salanti G. Evaluation of inconsistency in networks of interventions. Int J Epidemiol. 2013;42(1):332-45. doi: 10.1093/ije/dys222.

1. Nikolakopoulou A, Higgins JPT, Papakonstantinou T, Chaimani A, Del Giovane C, Egger M, et al. CINeMA: An approach for assessing confidence in the results of a network meta-analysis. PLOS Medicine. 2020;17(4):e1003082. doi: 10.1371/journal.pmed.1003082.
2. Higgins JPT, Altman DG, Gøtzsche PC, Jüni P, Moher D, Oxman AD, et al. The Cochrane Collaboration's tool for assessing risk of bias in randomised trials. Bmj. 2011;343:d5928.
3. Furukawa TA, Salanti G, Atkinson LZ, Leucht S, Ruhe HG, Turner EH, et al. Comparative efficacy and acceptability of first-generation and second-generation antidepressants in the acute treatment of major depression: protocol for a network meta-analysis. BMJ Open. 2016;6(7):e010919. doi: 10.1136/bmjopen-2015-010919.
4. Guyatt G, Oxman AD, Akl EA, Kunz R, Vist G, Brozek J, et al. GRADE guidelines: 1. Introduction—GRADE evidence profiles and summary of findings tables. J Clin Epidemiol. 2011;64(4):383-94.
5. Veroniki AA, Vasiliadis HS, Higgins JP, Salanti G. Evaluation of inconsistency in networks of interventions. Int J Epidemiol. 2013;42(1):332-45. doi: 10.1093/ije/dys222.

1. Nikolakopoulou A, Higgins JPT, Papakonstantinou T, Chaimani A, Del Giovane C, Egger M, et al. CINeMA: An approach for assessing confidence in the results of a network meta-analysis. PLOS Medicine. 2020;17(4):e1003082. doi: 10.1371/journal.pmed.1003082.
2. Papakonstantinou T, Nikolakopoulou A, Higgins JPT, Egger M, Salanti G. CINeMA: Software for semiautomated assessment of the confidence in the results of network meta-analysis. Campbell Systematic Reviews. 2020;16(1):e1080. doi: 10.1002/cl2.1080.
3. Guyatt G, Oxman AD, Akl EA, Kunz R, Vist G, Brozek J, et al. GRADE guidelines: 1. Introduction—GRADE evidence profiles and summary of findings tables. J Clin Epidemiol. 2011;64(4):383-94.
4. Gradepro GDT. GRADEpro guideline development tool [software]. McMaster University. 2015;435.
5. Higgins JPT, Altman DG, Gøtzsche PC, Jüni P, Moher D, Oxman AD, et al. The Cochrane Collaboration's tool for assessing risk of bias in randomised trials. Bmj. 2011;343:d5928.
6. Furukawa TA, Salanti G, Atkinson LZ, Leucht S, Ruhe HG, Turner EH, et al. Comparative efficacy and acceptability of first-generation and second-generation antidepressants in the acute

treatment of major depression: protocol for a network meta-analysis. BMJ Open. 2016;6(7):e010919. doi: 10.1136/bmjopen-2015-010919.
7. Veroniki AA, Vasiliadis HS, Higgins JP, Salanti G. Evaluation of inconsistency in networks of interventions. Int J Epidemiol. 2013;42(1):332-45. doi: 10.1093/ije/dys222.
