# Supplementary material for: Pharmacological and dietary-supplement treatments for autism spectrum disorder: a systematic review and network meta-analysis
Source: Mol Autism. 2022 Mar 4;13:10. doi: 10.1186/s13229-022-00488-4 (PMC8896153; doi:10.1186/s13229-022-00488-4)
Supplement: Supplementary file 2 — Additional file 2. Fig. S1. Network plots. [file 13229_2022_488_MOESM2_ESM.pdf]

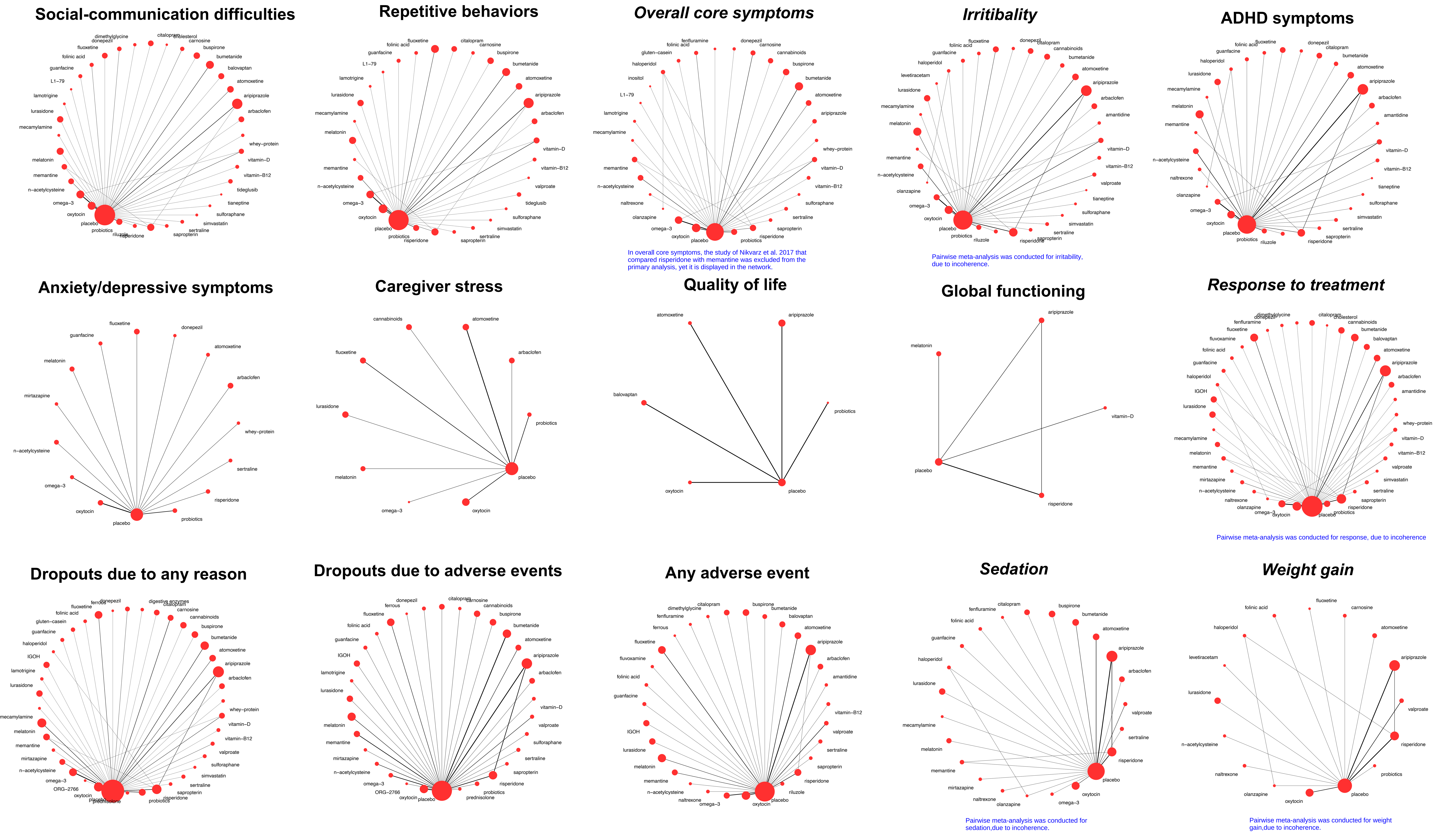

Social-communication difficulties
dimethylglycine
citalopram
cholesterol
donepezil
carnosine
fluoxetine
buspirone
folinic acid
bumetanide
guanfacine
balovaptan
L1−79
atomoxetine
lamotrigine
aripiprazole
lurasidone
arbaclofen
mecamylamine
whey−protein
melatonin
vitamin−D
memantine
vitamin−B12
n−acetylcysteine
tideglusib
omega−3
tianeptine
oxytocin
sulforaphane
placebo
simvastatin
probiotics
sertraline
riluzole
risperidone
sapropterin
Repetitive behaviors
fluoxetine
citalopram
folinic acid
carnosine
guanfacine
buspirone
L1−79
bumetanide
lamotrigine
atomoxetine
lurasidone
aripiprazole
mecamylamine
arbaclofen
melatonin
vitamin−D
memantine
vitamin−B12
n−acetylcysteine
valproate
omega−3
tideglusib
oxytocin
sulforaphane
placebo
simvastatin
probiotics
sertraline
risperidone
sapropterin
Overall core symptoms
fenfluramine
donepezil
folinic acid
carnosine
gluten−casein
cannabinoids
haloperidol
buspirone
inositol
bumetanide
L1−79
atomoxetine
lamotrigine
aripiprazole
mecamylamine
whey−protein
memantine
vitamin−D
n−acetylcysteine
vitamin−B12
naltrexone
sulforaphane
olanzapine
sertraline
omega−3
sapropterin
oxytocin
placebo
probiotics
risperidone
In overall core symptoms, the study of Nikvarz et al. 2017 that compared risperidone with memantine was excluded from the primary analysis, yet it is displayed in the network.
Irritability
fluoxetine
donepezil
folinic acid
citalopram
guanfacine
cannabinoids
haloperidol
bumetanide
levetiracetam
atomoxetine
lurasidone
aripiprazole
mecamylamine
arbaclofen
melatonin
amantidine
memantine
vitamin−D
n−acetylcysteine
vitamin−B12
olanzapine
valproate
omega−3
tianeptine
oxytocin
sulforaphane
placebo
simvastatin
probiotics
sertraline
riluzole
risperidone
sapropterin
Pairwise meta-analysis was conducted for irritability, due to incoherence.
ADHD symptoms
folinic acid
fluoxetine
donepezil
guanfacine
citalopram
haloperidol
bumetanide
lurasidone
atomoxetine
mecamylamine
aripiprazole
melatonin
arbaclofen
memantine
amantidine
n−acetylcysteine
vitamin−D
naltrexone
vitamin−B12
olanzapine
tianeptine
omega−3
sulforaphane
oxytocin
simvastatin
placebo
sertraline
probiotics
riluzole
risperidone
sapropterin
Anxiety/depressive symptoms
fluoxetine
donepezil
guanfacine
atomoxetine
melatonin
arbaclofen
mirtazapine
whey−protein
n−acetylcysteine
sertraline
omega−3
risperidone
oxytocin
probiotics
placebo
Caregiver stress
cannabinoids
atomoxetine
fluoxetine
arbaclofen
lurasidone
probiotics
melatonin
placebo
omega−3
oxytocin
Quality of life
atomoxetine
aripiprazole
balovaptan
probiotics
oxytocin
placebo
Global functioning
aripiprazole
melatonin
vitamin−D
placebo
risperidone
Response to treatment
dimethylglycine
citalopram
donepezil
cholesterol
fenfluramine
cannabinoids
fluoxetine
bumetanide
fluvoxamine
balovaptan
folinic acid
atomoxetine
guanfacine
aripiprazole
haloperidol
arbaclofen
IGOH
amantidine
lurasidone
whey−protein
mecamylamine
vitamin−D
melatonin
vitamin−B12
memantine
valproate
mirtazapine
simvastatin
n−acetylcysteine
sertraline
naltrexone
sapropterin
olanzapine
risperidone
omega−3
probiotics
oxytocin
placebo
Pairwise meta-analysis was conducted for response, due to incoherence
Dropouts due to any reason
ferrous
donepezil
digestive enzymes
fluoxetine
citalopram
folinic acid
carnosine
gluten−casein
cannabinoids
guanfacine
buspirone
haloperidol
bumetanide
IGOH
atomoxetine
lamotrigine
aripiprazole
arbaclofen
lurasidone
whey−protein
mecamylamine
vitamin−D
melatonin
vitamin−B12
memantine
valproate
mirtazapine
sulforaphane
n−acetylcysteine
simvastatin
omega−3
sertraline
ORG−2766
sapropterin
oxytocin
risperidone
placebo
prednisolone
probiotics
Dropouts due to adverse events
donepezil
citalopram
ferrous
carnosine
cannabinoids
fluoxetine
buspirone
folinic acid
bumetanide
atomoxetine
guanfacine
aripiprazole
IGOH
arbaclofen
lamotrigine
lurasidone
vitamin−D
melatonin
valproate
memantine
sulforaphane
mirtazapine
sertraline
n−acetylcysteine
sapropterin
omega−3
risperidone
ORG−2766
probiotics
oxytocin
placebo
prednisolone
Any adverse event
citalopram
buspirone
dimethylglycine
bumetanide
fenfluramine
balovaptan
ferrous
atomoxetine
aripiprazole
fluoxetine
arbaclofen
fluvoxamine
amantidine
folinic acid
guanfacine
vitamin−B12
IGOH
valproate
lurasidone
sertraline
melatonin
sapropterin
memantine
risperidone
n−acetylcysteine
riluzole
naltrexone
omega−3
oxytocin
placebo
Sedation
citalopram
fenfluramine
buspirone
bumetanide
folinic acid
atomoxetine
guanfacine
aripiprazole
haloperidol
arbaclofen
lurasidone
valproate
mecamylamine
sertraline
melatonin
risperidone
memantine
placebo
mirtazapine
oxytocin
naltrexone
olanzapine
omega−3
Pairwise meta-analysis was conducted for sedation,due to incoherence.
Weight gain
fluoxetine
folinic acid
carnosine
haloperidol
atomoxetine
levetiracetam
aripiprazole
lurasidone
valproate
n−acetylcysteine
risperidone
naltrexone
probiotics
olanzapine
oxytocin
placebo
Pairwise meta-analysis was conducted for weight gain,due to incoherence.

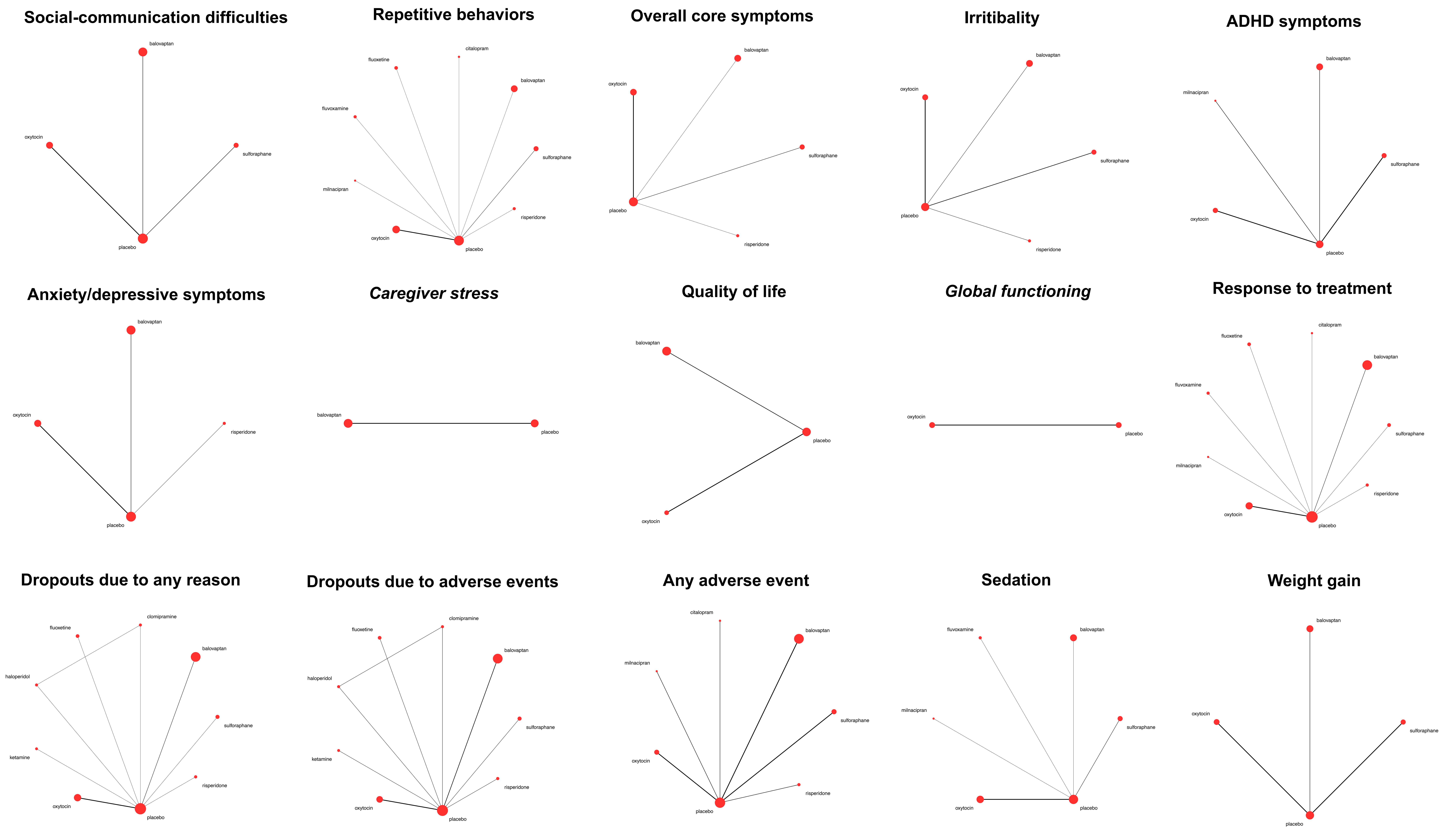
Social-communication difficulties
balovaptan
oxytocin
sulforaphane
placebo
Repetitive behaviors
citalopram
fluoxetine
balovaptan
fluvoxamine
sulforaphane
milnacipran
risperidone
oxytocin
placebo
Overall core symptoms
balovaptan
oxytocin
sulforaphane
placebo
risperidone
Irritibality
balovaptan
oxytocin
sulforaphane
placebo
risperidone
ADHD symptoms
balovaptan
milnacipran
sulforaphane
oxytocin
placebo
Anxiety/depressive symptoms
balovaptan
oxytocin
risperidone
placebo
Caregiver stress
balovaptan
placebo
Quality of life
balovaptan
placebo
oxytocin
Global functioning
oxytocin
placebo
Response to treatment
citalopram
fluoxetine
balovaptan
fluvoxamine
sulforaphane
milnacipran
risperidone
oxytocin
placebo
Dropouts due to any reason
clomipramine
fluoxetine
balovaptan
haloperidol
sulforaphane
ketamine
risperidone
oxytocin
placebo
Dropouts due to adverse events
clomipramine
fluoxetine
balovaptan
haloperidol
sulforaphane
ketamine
risperidone
oxytocin
placebo
Any adverse event
citalopram
balovaptan
milnacipran
sulforaphane
oxytocin
risperidone
placebo
Sedation
fluvoxamine
balovaptan
milnacipran
sulforaphane
oxytocin
placebo
Weight gain
balovaptan
oxytocin
sulforaphane
placebo
