# Supplementary material for: Pharmacological and dietary-supplement treatments for autism spectrum disorder: a systematic review and network meta-analysis
Source: Mol Autism. 2022 Mar 4;13:10. doi: 10.1186/s13229-022-00488-4 (PMC8896153; doi:10.1186/s13229-022-00488-4)
Supplement: Supplementary file 3 — Additional file 3. Fig. S2. Forest plots for secondary outcomes. [file 13229_2022_488_MOESM3_ESM.pdf]

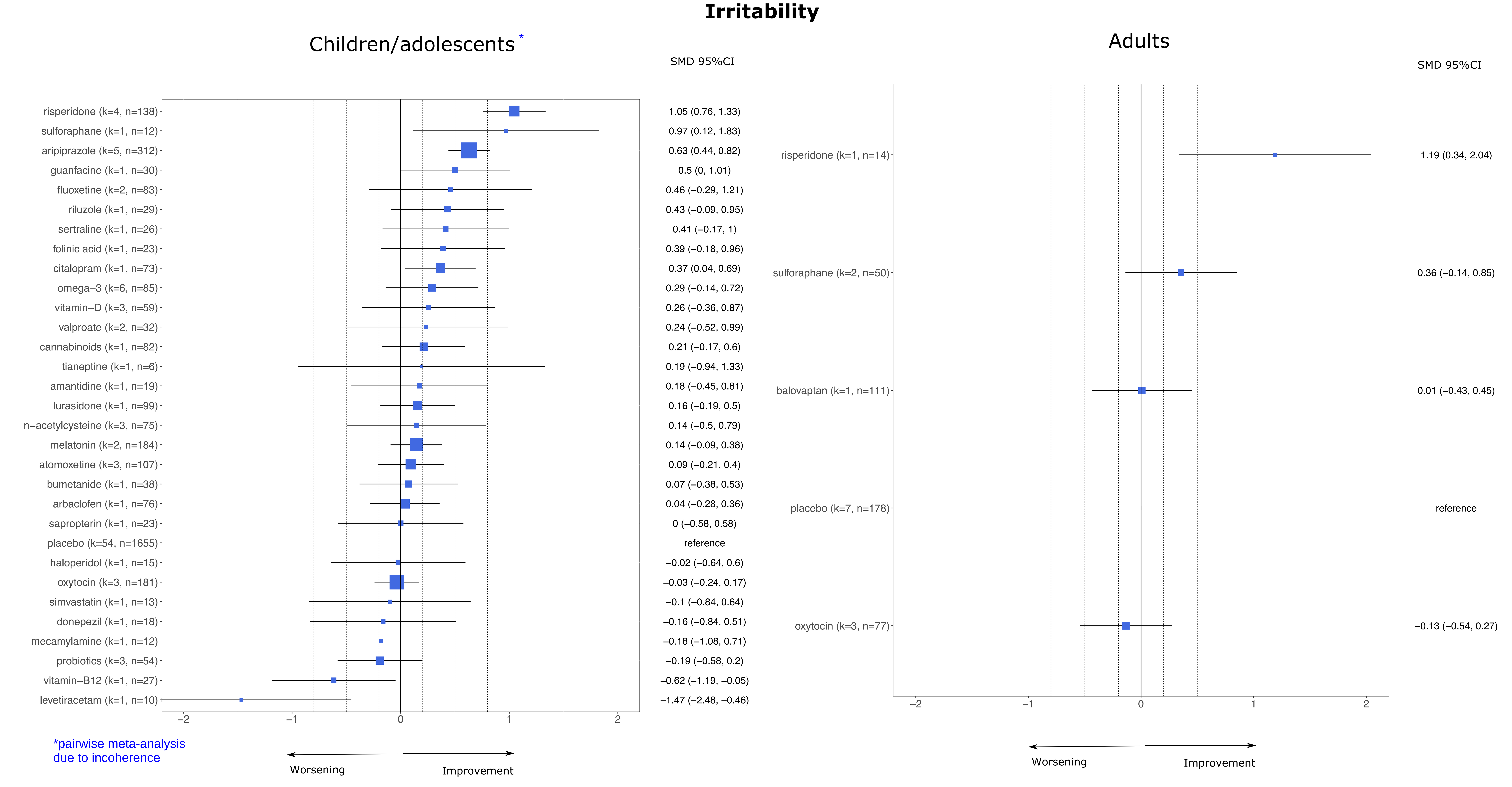

Irritability
Children/adolescents *
SMD 95%CI
risperidone (k=4, n=138) 1.05 (0.76, 1.33)
sulforaphane (k=1, n=12) 0.97 (0.12, 1.83)
aripiprazole (k=5, n=312) 0.63 (0.44, 0.82)
guanfacine (k=1, n=30) 0.5 (0, 1.01)
fluoxetine (k=2, n=83) 0.46 (−0.29, 1.21)
riluzole (k=1, n=29) 0.43 (−0.09, 0.95)
sertraline (k=1, n=26) 0.41 (−0.17, 1)
folinic acid (k=1, n=23) 0.39 (−0.18, 0.96)
citalopram (k=1, n=73) 0.37 (0.04, 0.69)
omega−3 (k=6, n=85) 0.29 (−0.14, 0.72)
vitamin−D (k=3, n=59) 0.26 (−0.36, 0.87)
valproate (k=2, n=32) 0.24 (−0.52, 0.99)
cannabinoids (k=1, n=82) 0.21 (−0.17, 0.6)
tianeptine (k=1, n=6) 0.19 (−0.94, 1.33)
amantidine (k=1, n=19) 0.18 (−0.45, 0.81)
lurasidone (k=1, n=99) 0.16 (−0.19, 0.5)
n−acetylcysteine (k=3, n=75) 0.14 (−0.5, 0.79)
melatonin (k=2, n=184) 0.14 (−0.09, 0.38)
atomoxetine (k=3, n=107) 0.09 (−0.21, 0.4)
bumetanide (k=1, n=38) 0.07 (−0.38, 0.53)
arbaclofen (k=1, n=76) 0.04 (−0.28, 0.36)
sapropterin (k=1, n=23) 0 (−0.58, 0.58)
placebo (k=54, n=1655) reference
haloperidol (k=1, n=15) −0.02 (−0.64, 0.6)
oxytocin (k=3, n=181) −0.03 (−0.24, 0.17)
simvastatin (k=1, n=13) −0.1 (−0.84, 0.64)
donepezil (k=1, n=18) −0.16 (−0.84, 0.51)
mecamylamine (k=1, n=12) −0.18 (−1.08, 0.71)
probiotics (k=3, n=54) −0.19 (−0.58, 0.2)
vitamin−B12 (k=1, n=27) −0.62 (−1.19, −0.05)
levetiracetam (k=1, n=10) −1.47 (−2.48, −0.46)
−2 −1 0 1 2
Worsening
Improvement
*pairwise meta-analysis
due to incoherence
Adults
SMD 95%CI
risperidone (k=1, n=14) 1.19 (0.34, 2.04)
sulforaphane (k=2, n=50) 0.36 (−0.14, 0.85)
balovaptan (k=1, n=111) 0.01 (−0.43, 0.45)
placebo (k=7, n=178) reference
oxytocin (k=3, n=77) −0.13 (−0.54, 0.27)
−2 −1 0 1 2
Worsening
Improvement

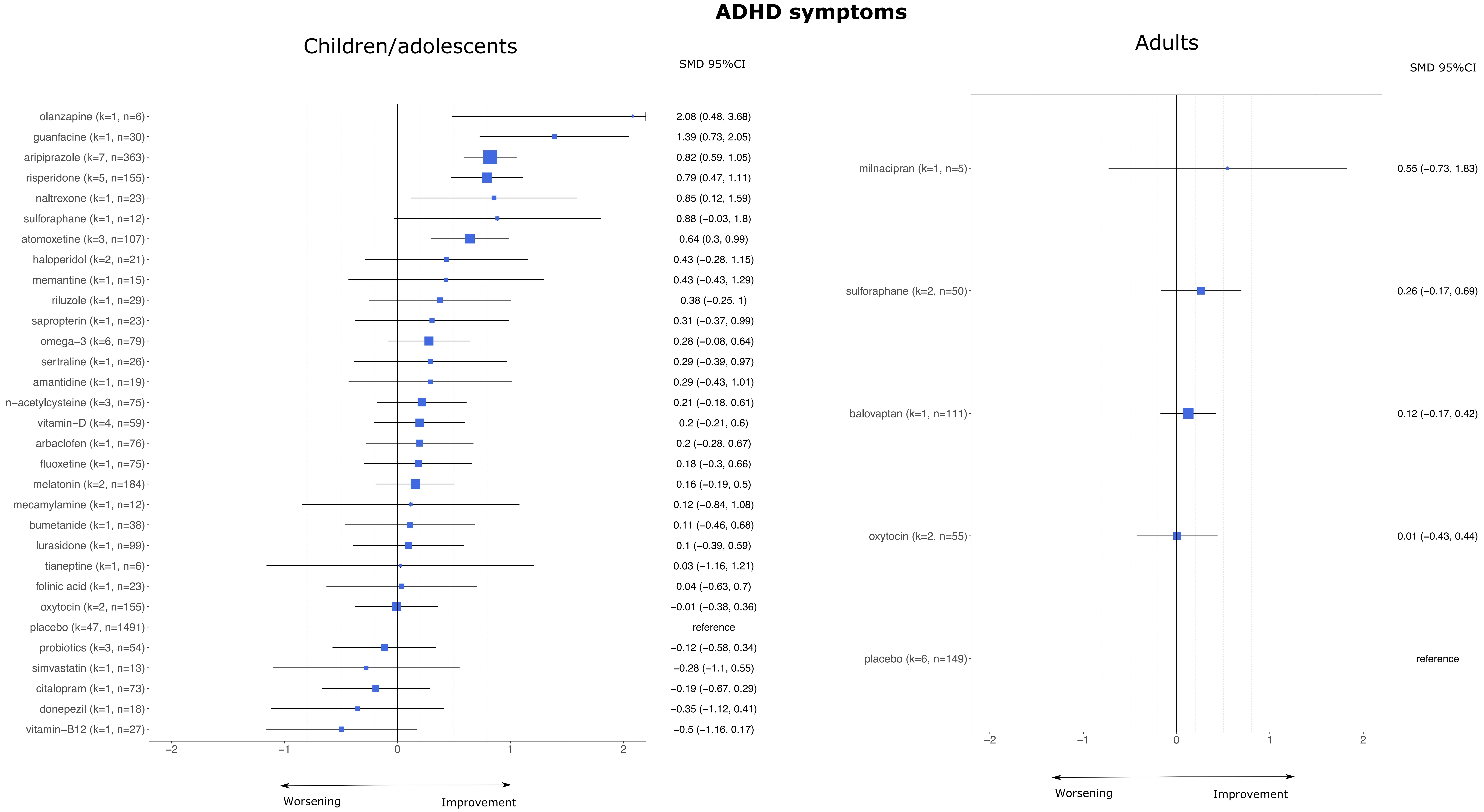
ADHD symptoms
Children/adolescents
SMD 95%CI
olanzapine (k=1, n=6)
2.08 (0.48, 3.68)
guanfacine (k=1, n=30)
1.39 (0.73, 2.05)
aripiprazole (k=7, n=363)
0.82 (0.59, 1.05)
risperidone (k=5, n=155)
0.79 (0.47, 1.11)
naltrexone (k=1, n=23)
0.85 (0.12, 1.59)
sulforaphane (k=1, n=12)
0.88 (−0.03, 1.8)
atomoxetine (k=3, n=107)
0.64 (0.3, 0.99)
haloperidol (k=2, n=21)
0.43 (−0.28, 1.15)
memantine (k=1, n=15)
0.43 (−0.43, 1.29)
riluzole (k=1, n=29)
0.38 (−0.25, 1)
sapropterin (k=1, n=23)
0.31 (−0.37, 0.99)
omega−3 (k=6, n=79)
0.28 (−0.08, 0.64)
sertraline (k=1, n=26)
0.29 (−0.39, 0.97)
amantidine (k=1, n=19)
0.29 (−0.43, 1.01)
n−acetylcysteine (k=3, n=75)
0.21 (−0.18, 0.61)
vitamin−D (k=4, n=59)
0.2 (−0.21, 0.6)
arbaclofen (k=1, n=76)
0.2 (−0.28, 0.67)
fluoxetine (k=1, n=75)
0.18 (−0.3, 0.66)
melatonin (k=2, n=184)
0.16 (−0.19, 0.5)
mecamylamine (k=1, n=12)
0.12 (−0.84, 1.08)
bumetanide (k=1, n=38)
0.11 (−0.46, 0.68)
lurasidone (k=1, n=99)
0.1 (−0.39, 0.59)
tianeptine (k=1, n=6)
0.03 (−1.16, 1.21)
folinic acid (k=1, n=23)
0.04 (−0.63, 0.7)
oxytocin (k=2, n=155)
−0.01 (−0.38, 0.36)
placebo (k=47, n=1491)
reference
probiotics (k=3, n=54)
−0.12 (−0.58, 0.34)
simvastatin (k=1, n=13)
−0.28 (−1.1, 0.55)
citalopram (k=1, n=73)
−0.19 (−0.67, 0.29)
donepezil (k=1, n=18)
−0.35 (−1.12, 0.41)
vitamin−B12 (k=1, n=27)
−0.5 (−1.16, 0.17)
−2
−1
0
1
2
Worsening
Improvement
Adults
SMD 95%CI
milnacipran (k=1, n=5)
0.55 (−0.73, 1.83)
sulforaphane (k=2, n=50)
0.26 (−0.17, 0.69)
balovaptan (k=1, n=111)
0.12 (−0.17, 0.42)
oxytocin (k=2, n=55)
0.01 (−0.43, 0.44)
placebo (k=6, n=149)
reference
−2
−1
0
1
2
Worsening
Improvement

Anxiety or depressive symptoms

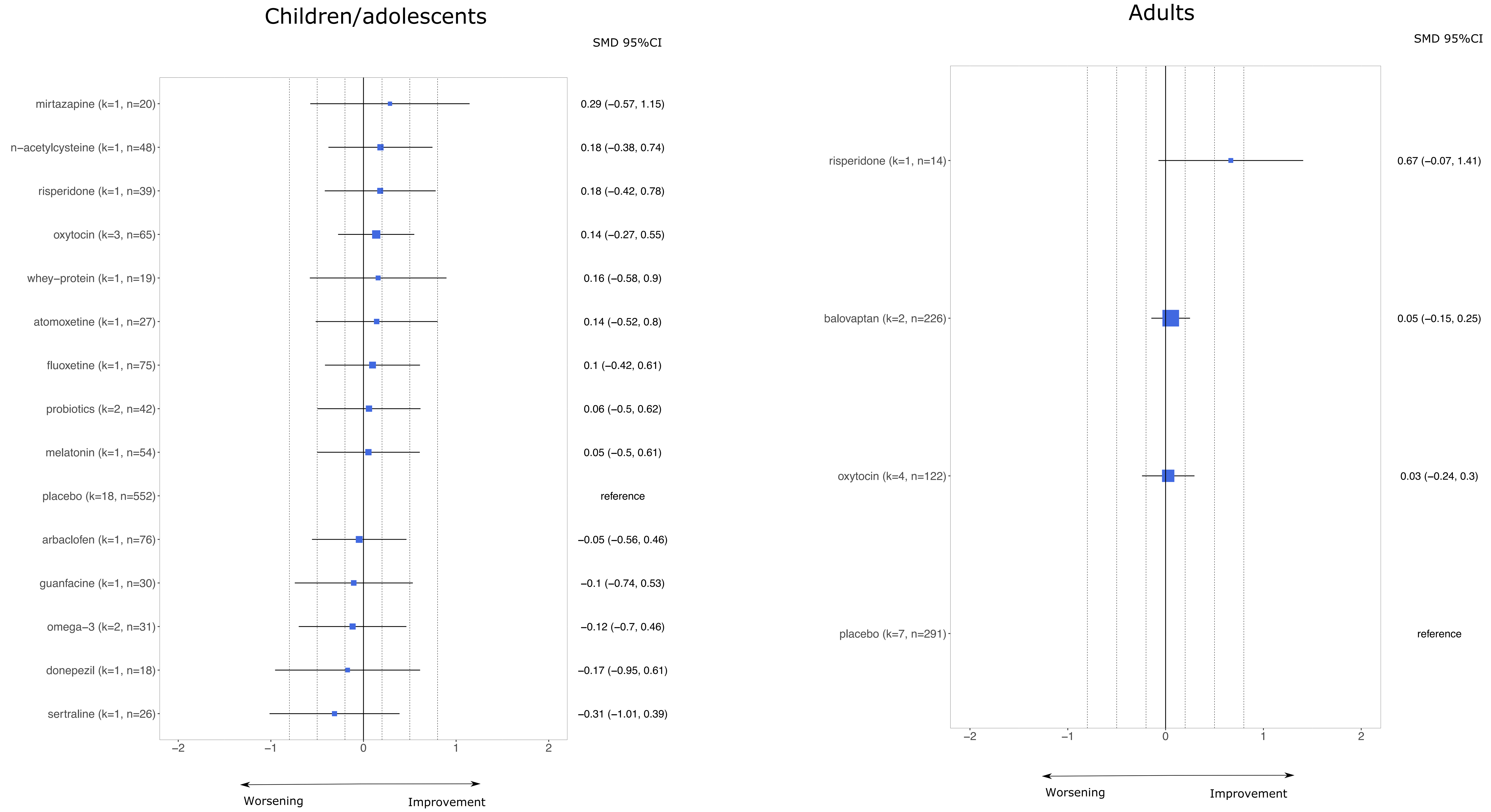

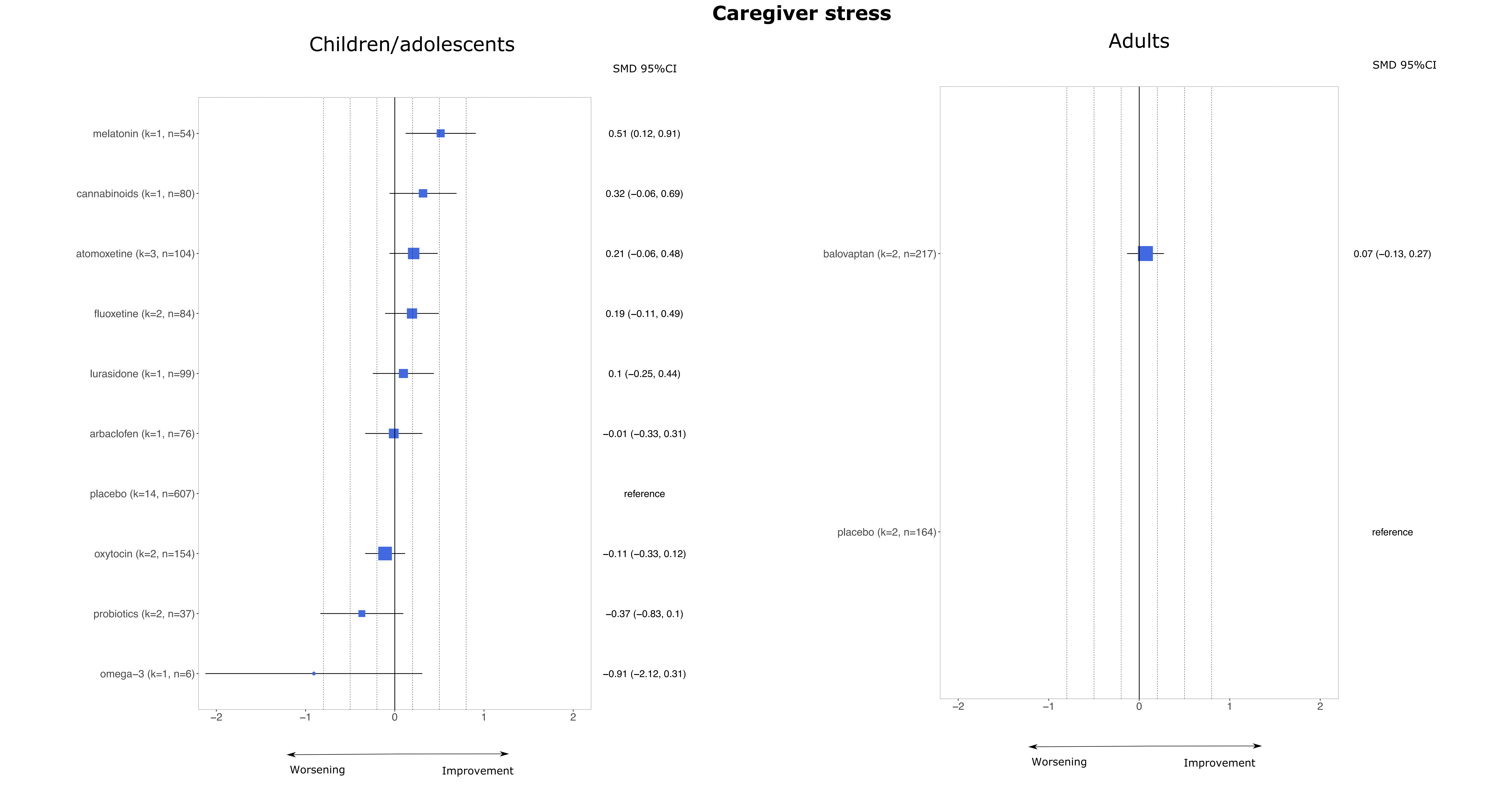

Caregiver stress
Children/adolescents
SMD 95%CI
melatonin (k=1, n=54)
0.51 (0.12, 0.91)
cannabinoids (k=1, n=80)
0.32 (−0.06, 0.69)
atomoxetine (k=3, n=104)
0.21 (−0.06, 0.48)
fluoxetine (k=2, n=84)
0.19 (−0.11, 0.49)
lurasidone (k=1, n=99)
0.1 (−0.25, 0.44)
arbaclofen (k=1, n=76)
−0.01 (−0.33, 0.31)
placebo (k=14, n=607)
reference
oxytocin (k=2, n=154)
−0.11 (−0.33, 0.12)
probiotics (k=2, n=37)
−0.37 (−0.83, 0.1)
omega−3 (k=1, n=6)
−0.91 (−2.12, 0.31)
−2
−1
0
1
2
Worsening
Improvement
Adults
SMD 95%CI
balovaptan (k=2, n=217)
0.07 (−0.13, 0.27)
placebo (k=2, n=164)
reference
−2
−1
0
1
2
Worsening
Improvement

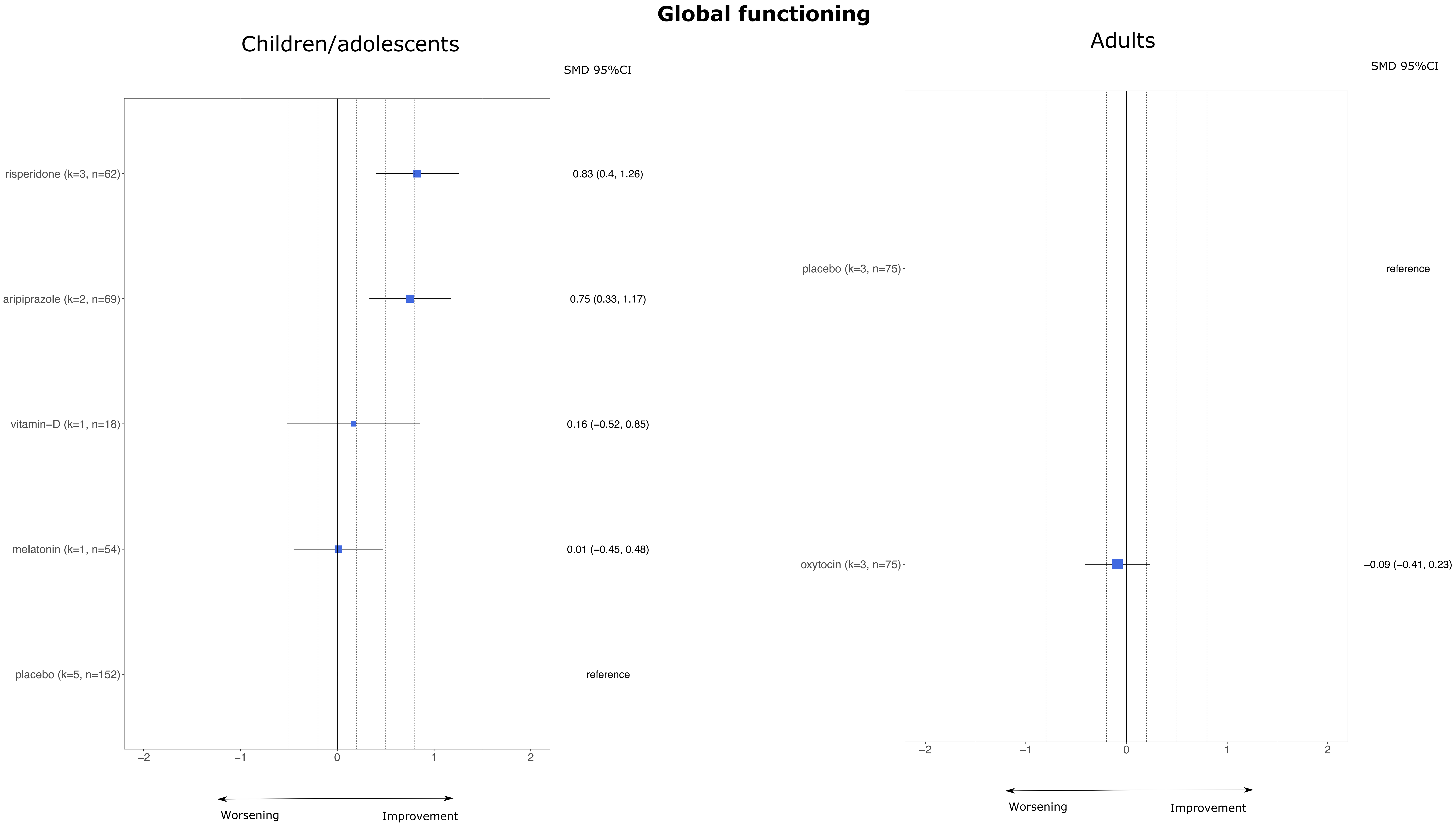
Global functioning
Children/adolescents
SMD 95%CI
risperidone (k=3, n=62)
0.83 (0.4, 1.26)
aripiprazole (k=2, n=69)
0.75 (0.33, 1.17)
vitamin−D (k=1, n=18)
0.16 (−0.52, 0.85)
melatonin (k=1, n=54)
0.01 (−0.45, 0.48)
placebo (k=5, n=152)
reference
−2
−1
0
1
2
Worsening
Improvement
Adults
SMD 95%CI
placebo (k=3, n=75)
reference
oxytocin (k=3, n=75)
−0.09 (−0.41, 0.23)
−2
−1
0
1
2
Worsening
Improvement

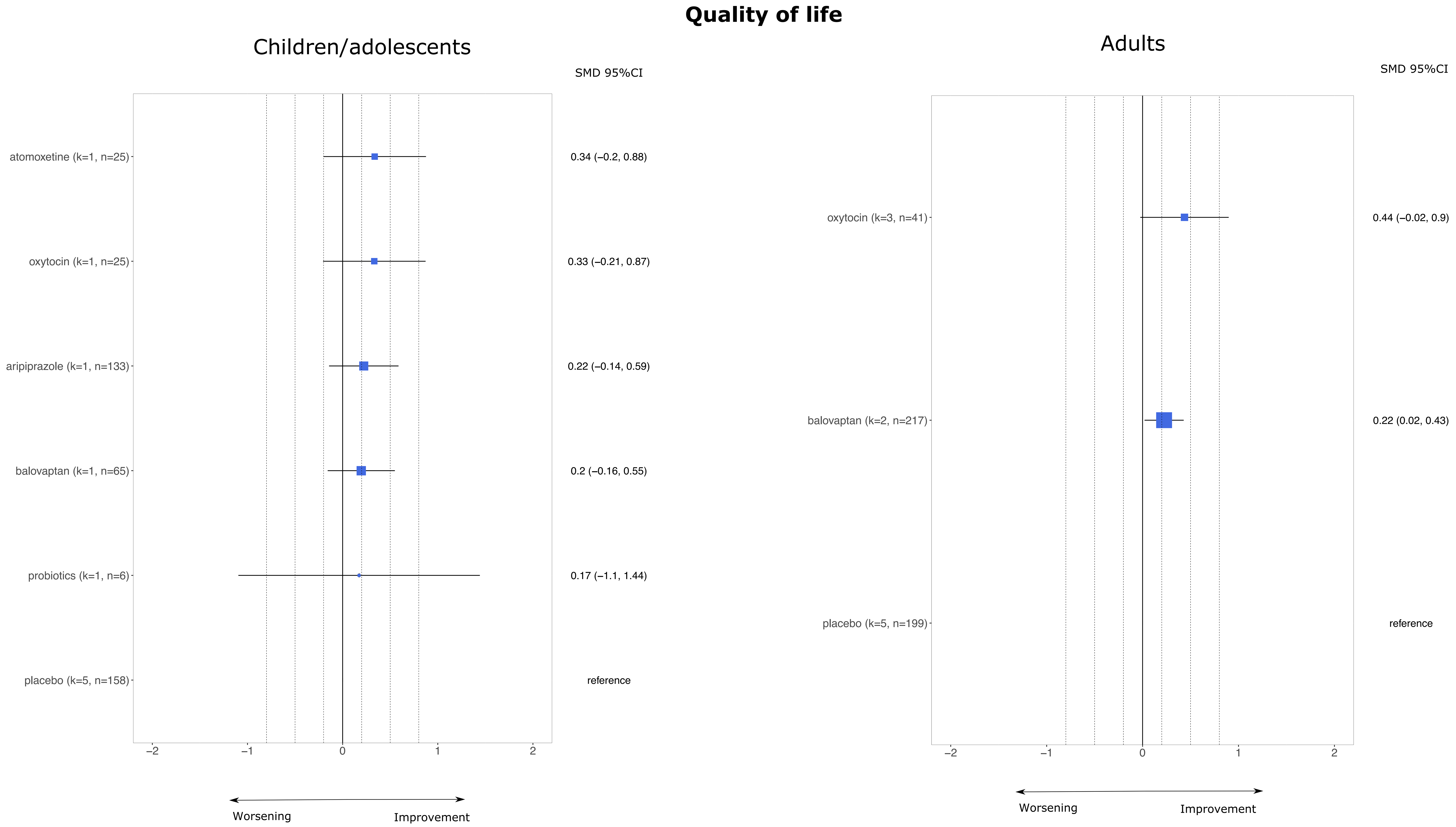
Quality of life
Children/adolescents
SMD 95%CI
atomoxetine (k=1, n=25)
0.34 (−0.2, 0.88)
oxytocin (k=1, n=25)
0.33 (−0.21, 0.87)
aripiprazole (k=1, n=133)
0.22 (−0.14, 0.59)
balovaptan (k=1, n=65)
0.2 (−0.16, 0.55)
probiotics (k=1, n=6)
0.17 (−1.1, 1.44)
placebo (k=5, n=158)
reference
−2
−1
0
1
2
Worsening
Improvement
Adults
SMD 95%CI
oxytocin (k=3, n=41)
0.44 (−0.02, 0.9)
balovaptan (k=2, n=217)
0.22 (0.02, 0.43)
placebo (k=5, n=199)
reference
−2
−1
0
1
2
Worsening
Improvement

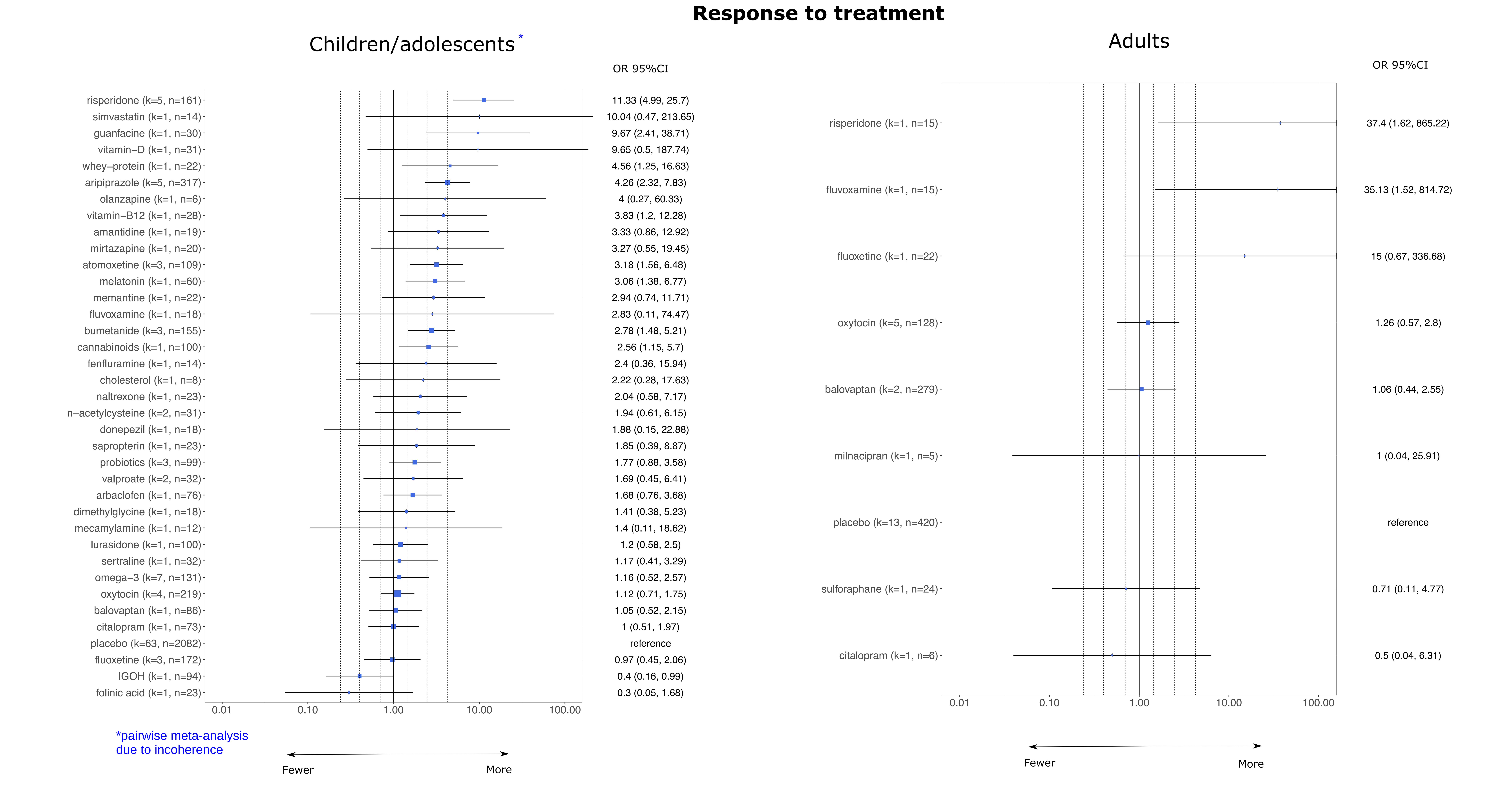

Response to treatment
Children/adolescents *
OR 95%CI
risperidone (k=5, n=161) 11.33 (4.99, 25.7)
simvastatin (k=1, n=14) 10.04 (0.47, 213.65)
guanfacine (k=1, n=30) 9.67 (2.41, 38.71)
vitamin−D (k=1, n=31) 9.65 (0.5, 187.74)
whey−protein (k=1, n=22) 4.56 (1.25, 16.63)
aripiprazole (k=5, n=317) 4.26 (2.32, 7.83)
olanzapine (k=1, n=6) 4 (0.27, 60.33)
vitamin−B12 (k=1, n=28) 3.83 (1.2, 12.28)
amantidine (k=1, n=19) 3.33 (0.86, 12.92)
mirtazapine (k=1, n=20) 3.27 (0.55, 19.45)
atomoxetine (k=3, n=109) 3.18 (1.56, 6.48)
melatonin (k=1, n=60) 3.06 (1.38, 6.77)
memantine (k=1, n=22) 2.94 (0.74, 11.71)
fluvoxamine (k=1, n=18) 2.83 (0.11, 74.47)
bumetanide (k=3, n=155) 2.78 (1.48, 5.21)
cannabinoids (k=1, n=100) 2.56 (1.15, 5.7)
fenfluramine (k=1, n=14) 2.4 (0.36, 15.94)
cholesterol (k=1, n=8) 2.22 (0.28, 17.63)
naltrexone (k=1, n=23) 2.04 (0.58, 7.17)
n−acetylcysteine (k=2, n=31) 1.94 (0.61, 6.15)
donepezil (k=1, n=18) 1.88 (0.15, 22.88)
sapropterin (k=1, n=23) 1.85 (0.39, 8.87)
probiotics (k=3, n=99) 1.77 (0.88, 3.58)
valproate (k=2, n=32) 1.69 (0.45, 6.41)
arbaclofen (k=1, n=76) 1.68 (0.76, 3.68)
dimethylglycine (k=1, n=18) 1.41 (0.38, 5.23)
mecamylamine (k=1, n=12) 1.4 (0.11, 18.62)
lurasidone (k=1, n=100) 1.2 (0.58, 2.5)
sertraline (k=1, n=32) 1.17 (0.41, 3.29)
omega−3 (k=7, n=131) 1.16 (0.52, 2.57)
oxytocin (k=4, n=219) 1.12 (0.71, 1.75)
balovaptan (k=1, n=86) 1.05 (0.52, 2.15)
citalopram (k=1, n=73) 1 (0.51, 1.97)
placebo (k=63, n=2082) reference
fluoxetine (k=3, n=172) 0.97 (0.45, 2.06)
IGOH (k=1, n=94) 0.4 (0.16, 0.99)
folinic acid (k=1, n=23) 0.3 (0.05, 1.68)
0.01
0.10
1.00
10.00
100.00
*pairwise meta-analysis
due to incoherence
Fewer
More
Adults
OR 95%CI
risperidone (k=1, n=15) 37.4 (1.62, 865.22)
fluvoxamine (k=1, n=15) 35.13 (1.52, 814.72)
fluoxetine (k=1, n=22) 15 (0.67, 336.68)
oxytocin (k=5, n=128) 1.26 (0.57, 2.8)
balovaptan (k=2, n=279) 1.06 (0.44, 2.55)
milnacipran (k=1, n=5) 1 (0.04, 25.91)
placebo (k=13, n=420) reference
sulforaphane (k=1, n=24) 0.71 (0.11, 4.77)
citalopram (k=1, n=6) 0.5 (0.04, 6.31)
0.01
0.10
1.00
10.00
100.00
Fewer
More

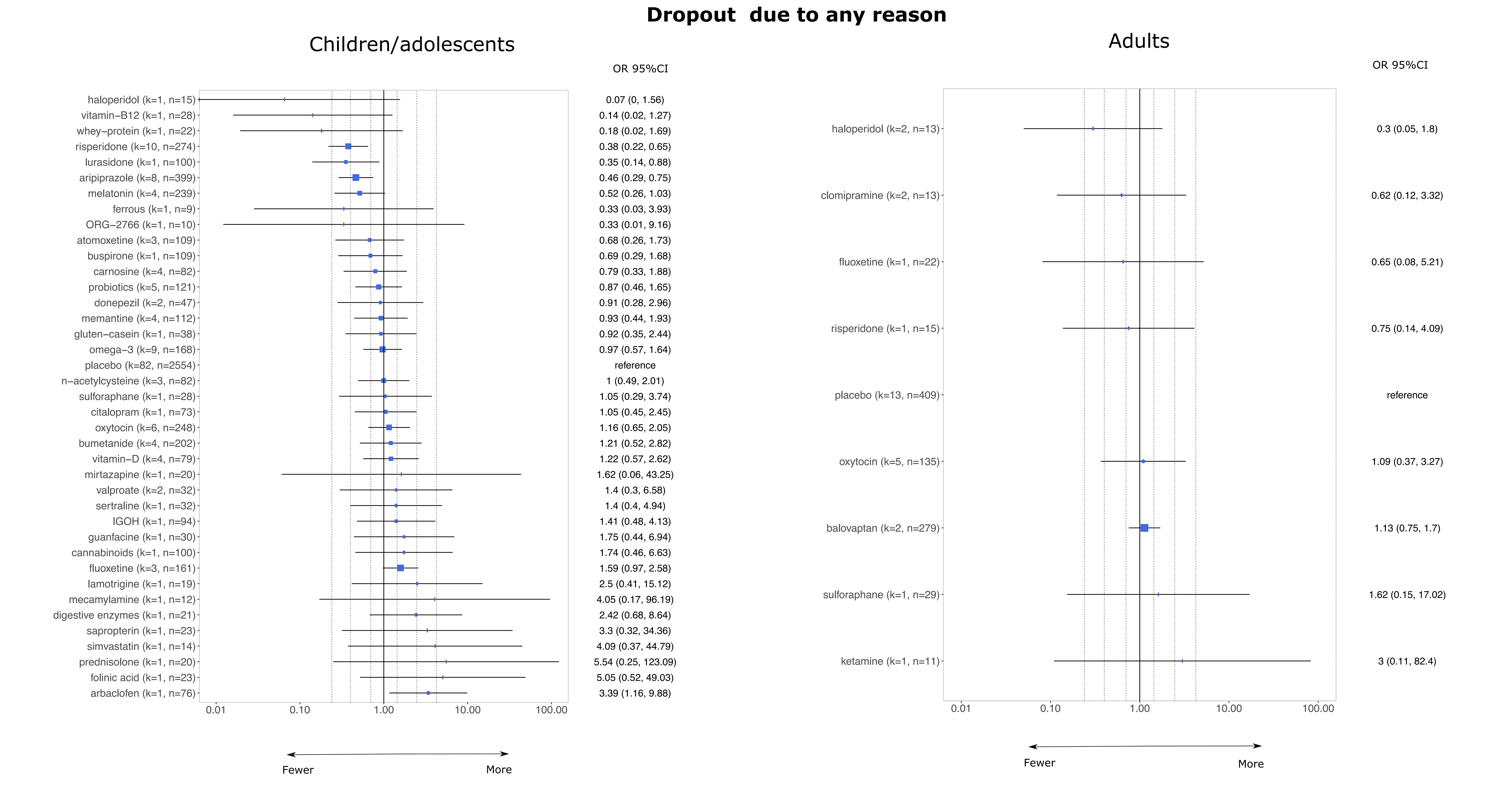
Dropout due to any reason
Children/adolescents
OR 95%CI
haloperidol (k=1, n=15) 0.07 (0, 1.56)
vitamin−B12 (k=1, n=28) 0.14 (0.02, 1.27)
whey−protein (k=1, n=22) 0.18 (0.02, 1.69)
risperidone (k=10, n=274) 0.38 (0.22, 0.65)
lurasidone (k=1, n=100) 0.35 (0.14, 0.88)
aripiprazole (k=8, n=399) 0.46 (0.29, 0.75)
melatonin (k=4, n=239) 0.52 (0.26, 1.03)
ferrous (k=1, n=9) 0.33 (0.03, 3.93)
ORG−2766 (k=1, n=10) 0.33 (0.01, 9.16)
atomoxetine (k=3, n=109) 0.68 (0.26, 1.73)
buspirone (k=1, n=109) 0.69 (0.29, 1.68)
carnosine (k=4, n=82) 0.79 (0.33, 1.88)
probiotics (k=5, n=121) 0.87 (0.46, 1.65)
donepezil (k=2, n=47) 0.91 (0.28, 2.96)
memantine (k=4, n=112) 0.93 (0.44, 1.93)
gluten−casein (k=1, n=38) 0.92 (0.35, 2.44)
omega−3 (k=9, n=168) 0.97 (0.57, 1.64)
placebo (k=82, n=2554) reference
n−acetylcysteine (k=3, n=82) 1 (0.49, 2.01)
sulforaphane (k=1, n=28) 1.05 (0.29, 3.74)
citalopram (k=1, n=73) 1.05 (0.45, 2.45)
oxytocin (k=6, n=248) 1.16 (0.65, 2.05)
bumetanide (k=4, n=202) 1.21 (0.52, 2.82)
vitamin−D (k=4, n=79) 1.22 (0.57, 2.62)
mirtazapine (k=1, n=20) 1.62 (0.06, 43.25)
valproate (k=2, n=32) 1.4 (0.3, 6.58)
sertraline (k=1, n=32) 1.4 (0.4, 4.94)
IGOH (k=1, n=94) 1.41 (0.48, 4.13)
guanfacine (k=1, n=30) 1.75 (0.44, 6.94)
cannabinoids (k=1, n=100) 1.74 (0.46, 6.63)
fluoxetine (k=3, n=161) 1.59 (0.97, 2.58)
lamotrigine (k=1, n=19) 2.5 (0.41, 15.12)
mecamylamine (k=1, n=12) 4.05 (0.17, 96.19)
digestive enzymes (k=1, n=21) 2.42 (0.68, 8.64)
sapropterin (k=1, n=23) 3.3 (0.32, 34.36)
simvastatin (k=1, n=14) 4.09 (0.37, 44.79)
prednisolone (k=1, n=20) 5.54 (0.25, 123.09)
folinic acid (k=1, n=23) 5.05 (0.52, 49.03)
arbaclofen (k=1, n=76) 3.39 (1.16, 9.88)
0.01
0.10
1.00
10.00
100.00
Fewer
More
Adults
OR 95%CI
haloperidol (k=2, n=13) 0.3 (0.05, 1.8)
clomipramine (k=2, n=13) 0.62 (0.12, 3.32)
fluoxetine (k=1, n=22) 0.65 (0.08, 5.21)
risperidone (k=1, n=15) 0.75 (0.14, 4.09)
placebo (k=13, n=409) reference
oxytocin (k=5, n=135) 1.09 (0.37, 3.27)
balovaptan (k=2, n=279) 1.13 (0.75, 1.7)
sulforaphane (k=1, n=29) 1.62 (0.15, 17.02)
ketamine (k=1, n=11) 3 (0.11, 82.4)
0.01
0.10
1.00
10.00
100.00
Fewer
More

Dropout due to adverse event

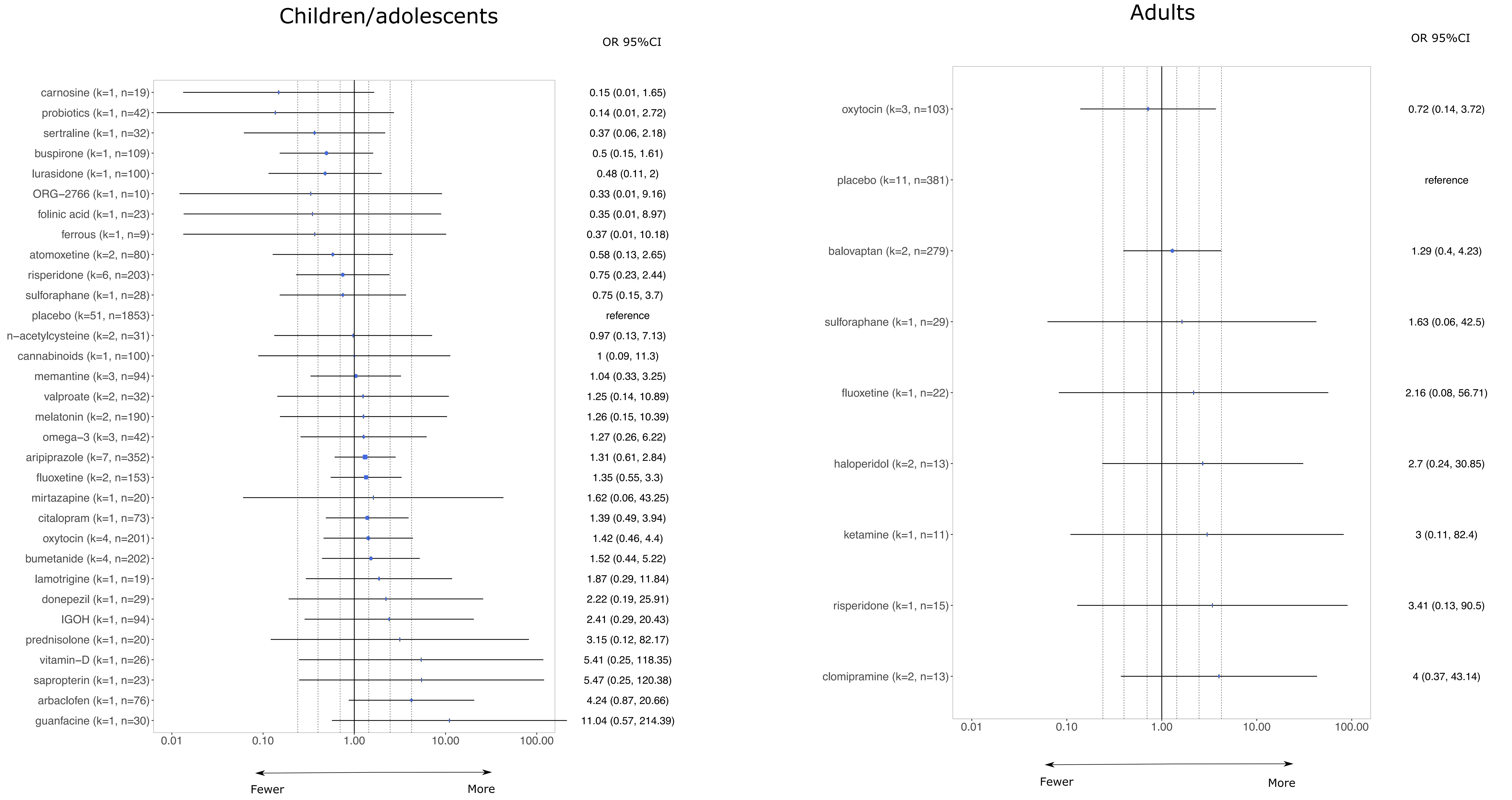

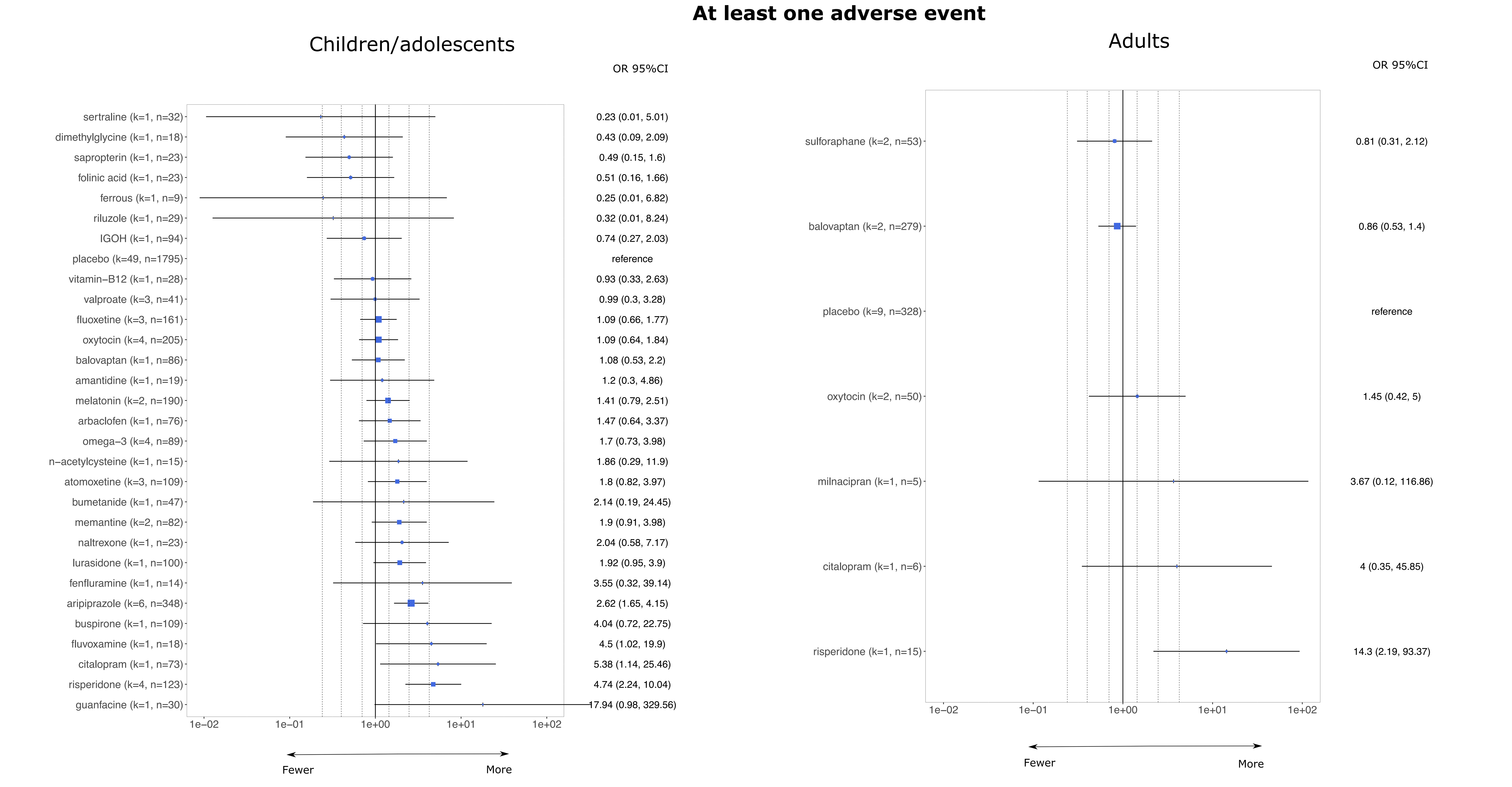
At least one adverse event
Children/adolescents
OR 95%CI
sertraline (k=1, n=32) 0.23 (0.01, 5.01)
dimethylglycine (k=1, n=18) 0.43 (0.09, 2.09)
sapropterin (k=1, n=23) 0.49 (0.15, 1.6)
folinic acid (k=1, n=23) 0.51 (0.16, 1.66)
ferrous (k=1, n=9) 0.25 (0.01, 6.82)
riluzole (k=1, n=29) 0.32 (0.01, 8.24)
IGOH (k=1, n=94) 0.74 (0.27, 2.03)
placebo (k=49, n=1795) reference
vitamin−B12 (k=1, n=28) 0.93 (0.33, 2.63)
valproate (k=3, n=41) 0.99 (0.3, 3.28)
fluoxetine (k=3, n=161) 1.09 (0.66, 1.77)
oxytocin (k=4, n=205) 1.09 (0.64, 1.84)
balovaptan (k=1, n=86) 1.08 (0.53, 2.2)
amantidine (k=1, n=19) 1.2 (0.3, 4.86)
melatonin (k=2, n=190) 1.41 (0.79, 2.51)
arbaclofen (k=1, n=76) 1.47 (0.64, 3.37)
omega−3 (k=4, n=89) 1.7 (0.73, 3.98)
n−acetylcysteine (k=1, n=15) 1.86 (0.29, 11.9)
atomoxetine (k=3, n=109) 1.8 (0.82, 3.97)
bumetanide (k=1, n=47) 2.14 (0.19, 24.45)
memantine (k=2, n=82) 1.9 (0.91, 3.98)
naltrexone (k=1, n=23) 2.04 (0.58, 7.17)
lurasidone (k=1, n=100) 1.92 (0.95, 3.9)
fenfluramine (k=1, n=14) 3.55 (0.32, 39.14)
aripiprazole (k=6, n=348) 2.62 (1.65, 4.15)
buspirone (k=1, n=109) 4.04 (0.72, 22.75)
fluvoxamine (k=1, n=18) 4.5 (1.02, 19.9)
citalopram (k=1, n=73) 5.38 (1.14, 25.46)
risperidone (k=4, n=123) 4.74 (2.24, 10.04)
guanfacine (k=1, n=30) 17.94 (0.98, 329.56)
1e−02
1e−01
1e+00
1e+01
1e+02
Fewer
More
Adults
OR 95%CI
sulforaphane (k=2, n=53) 0.81 (0.31, 2.12)
balovaptan (k=2, n=279) 0.86 (0.53, 1.4)
placebo (k=9, n=328) reference
oxytocin (k=2, n=50) 1.45 (0.42, 5)
milnacipran (k=1, n=5) 3.67 (0.12, 116.86)
citalopram (k=1, n=6) 4 (0.35, 45.85)
risperidone (k=1, n=15) 14.3 (2.19, 93.37)
1e−02
1e−01
1e+00
1e+01
1e+02
Fewer
More

Sedation

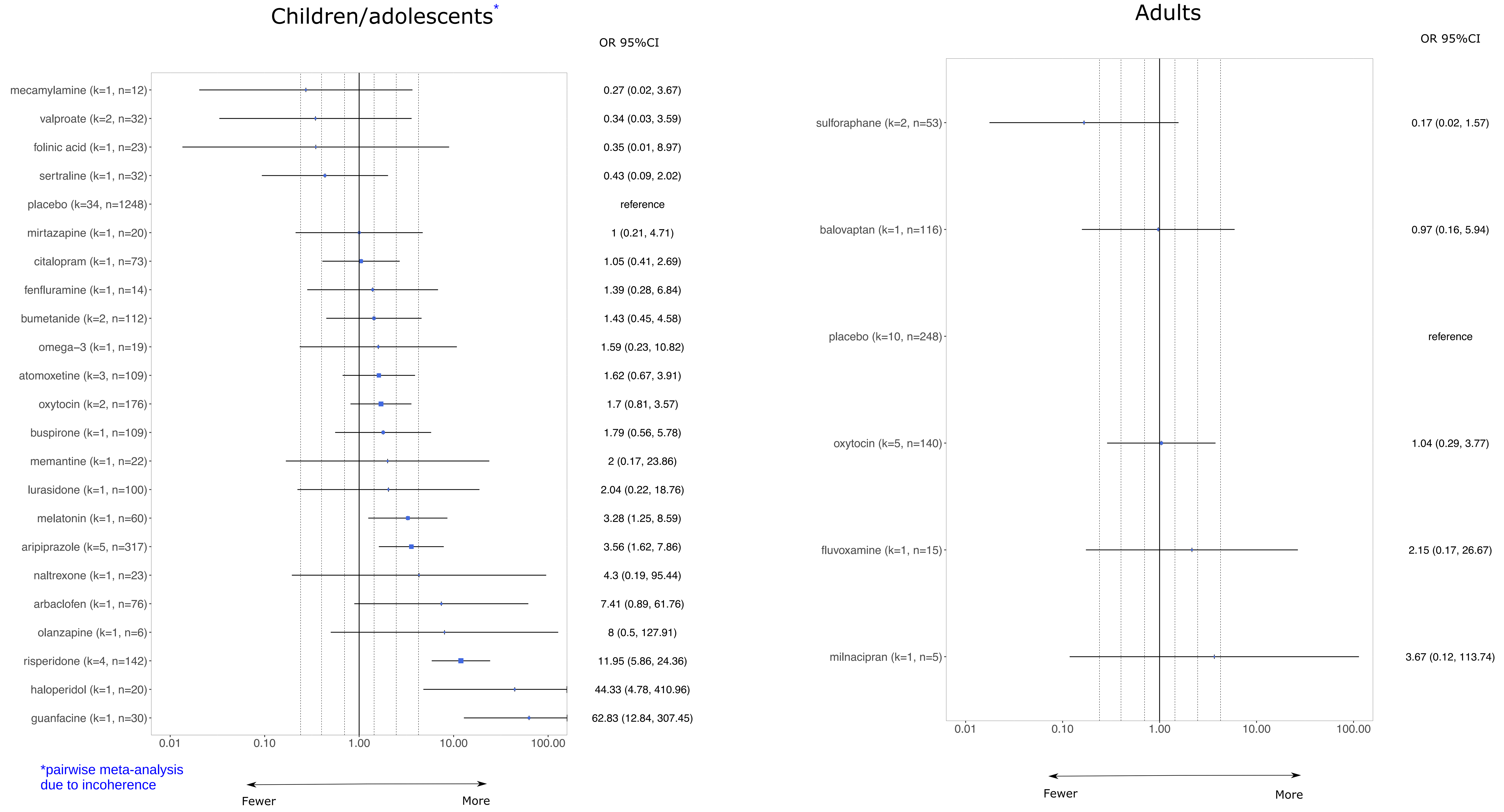
Children/adolescents*
OR 95%CI
mecamylamine (k=1, n=12)
valproate (k=2, n=32)
folinic acid (k=1, n=23)
sertraline (k=1, n=32)
placebo (k=34, n=1248)
mirtazapine (k=1, n=20)
citalopram (k=1, n=73)
fenfluramine (k=1, n=14)
bumetanide (k=2, n=112)
omega−3 (k=1, n=19)
atomoxetine (k=3, n=109)
oxytocin (k=2, n=176)
buspirone (k=1, n=109)
memantine (k=1, n=22)
lurasidone (k=1, n=100)
melatonin (k=1, n=60)
aripiprazole (k=5, n=317)
naltrexone (k=1, n=23)
arbaclofen (k=1, n=76)
olanzapine (k=1, n=6)
risperidone (k=4, n=142)
haloperidol (k=1, n=20)
guanfacine (k=1, n=30)
0.27 (0.02, 3.67)
0.34 (0.03, 3.59)
0.35 (0.01, 8.97)
0.43 (0.09, 2.02)
reference
1 (0.21, 4.71)
1.05 (0.41, 2.69)
1.39 (0.28, 6.84)
1.43 (0.45, 4.58)
1.59 (0.23, 10.82)
1.62 (0.67, 3.91)
1.7 (0.81, 3.57)
1.79 (0.56, 5.78)
2 (0.17, 23.86)
2.04 (0.22, 18.76)
3.28 (1.25, 8.59)
3.56 (1.62, 7.86)
4.3 (0.19, 95.44)
7.41 (0.89, 61.76)
8 (0.5, 127.91)
11.95 (5.86, 24.36)
44.33 (4.78, 410.96)
62.83 (12.84, 307.45)
0.01
0.10
1.00
10.00
100.00
*pairwise meta-analysis
due to incoherence
Fewer
More
Adults
OR 95%CI
sulforaphane (k=2, n=53)
balovaptan (k=1, n=116)
placebo (k=10, n=248)
oxytocin (k=5, n=140)
fluvoxamine (k=1, n=15)
milnacipran (k=1, n=5)
0.17 (0.02, 1.57)
0.97 (0.16, 5.94)
reference
1.04 (0.29, 3.77)
2.15 (0.17, 26.67)
3.67 (0.12, 113.74)
0.01
0.10
1.00
10.00
100.00
Fewer
More

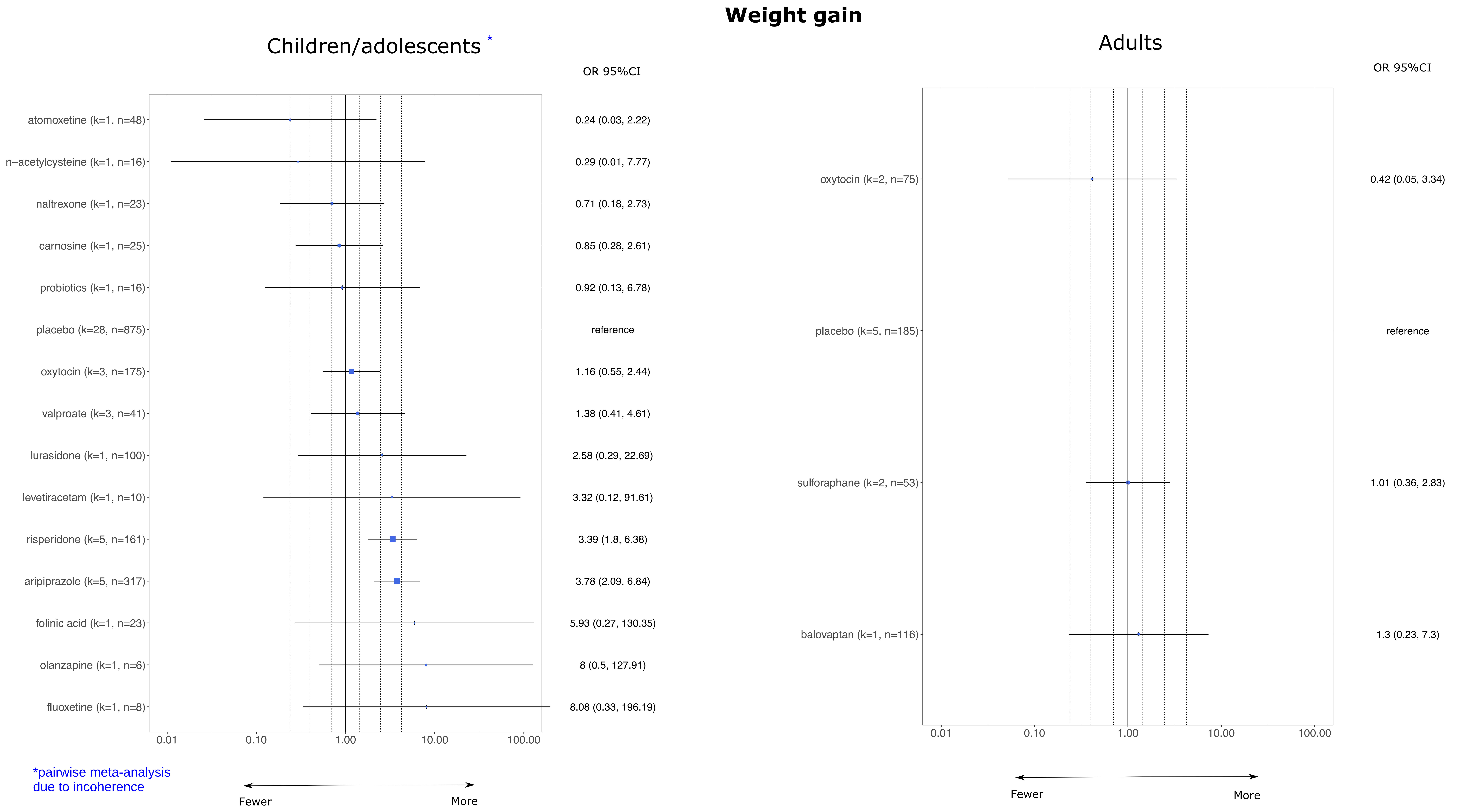
Weight gain
Children/adolescents *
OR 95%CI
atomoxetine (k=1, n=48)
0.24 (0.03, 2.22)
n−acetylcysteine (k=1, n=16)
0.29 (0.01, 7.77)
naltrexone (k=1, n=23)
0.71 (0.18, 2.73)
carnosine (k=1, n=25)
0.85 (0.28, 2.61)
probiotics (k=1, n=16)
0.92 (0.13, 6.78)
placebo (k=28, n=875)
reference
oxytocin (k=3, n=175)
1.16 (0.55, 2.44)
valproate (k=3, n=41)
1.38 (0.41, 4.61)
lurasidone (k=1, n=100)
2.58 (0.29, 22.69)
levetiracetam (k=1, n=10)
3.32 (0.12, 91.61)
risperidone (k=5, n=161)
3.39 (1.8, 6.38)
aripiprazole (k=5, n=317)
3.78 (2.09, 6.84)
folinic acid (k=1, n=23)
5.93 (0.27, 130.35)
olanzapine (k=1, n=6)
8 (0.5, 127.91)
fluoxetine (k=1, n=8)
8.08 (0.33, 196.19)
0.01
0.10
1.00
10.00
100.00
*pairwise meta-analysis
due to incoherence
Fewer
More
Adults
OR 95%CI
oxytocin (k=2, n=75)
0.42 (0.05, 3.34)
placebo (k=5, n=185)
reference
sulforaphane (k=2, n=53)
1.01 (0.36, 2.83)
balovaptan (k=1, n=116)
1.3 (0.23, 7.3)
0.01
0.10
1.00
10.00
100.00
Fewer
More

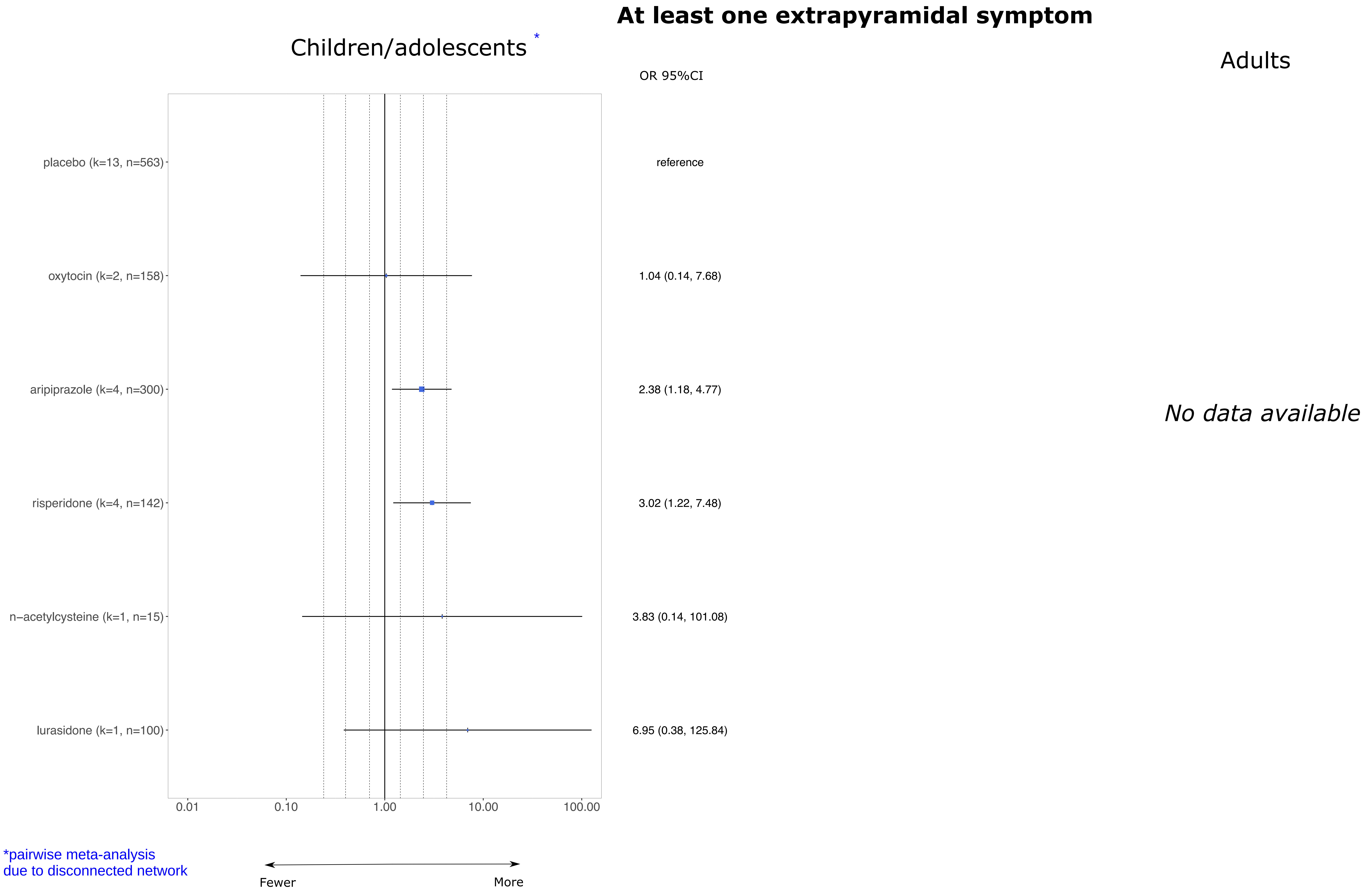
At least one extrapyramidal symptom
Children/adolescents *
Adults
OR 95%CI
placebo (k=13, n=563)
reference
oxytocin (k=2, n=158)
1.04 (0.14, 7.68)
aripiprazole (k=4, n=300)
2.38 (1.18, 4.77)
risperidone (k=4, n=142)
3.02 (1.22, 7.48)
n−acetylcysteine (k=1, n=15)
3.83 (0.14, 101.08)
lurasidone (k=1, n=100)
6.95 (0.38, 125.84)
0.01
0.10
1.00
10.00
100.00
Fewer
More
No data available
*pairwise meta-analysis
due to disconnected network
