# Supplementary figures and images for: Pharmacological and dietary-supplement treatments for autism spectrum disorder: a systematic review and network meta-analysis
Source: Mol Autism. 2022 Mar 4;13:10. doi: 10.1186/s13229-022-00488-4 (PMC8896153; doi:10.1186/s13229-022-00488-4)

## Social-communication difficulties in adults

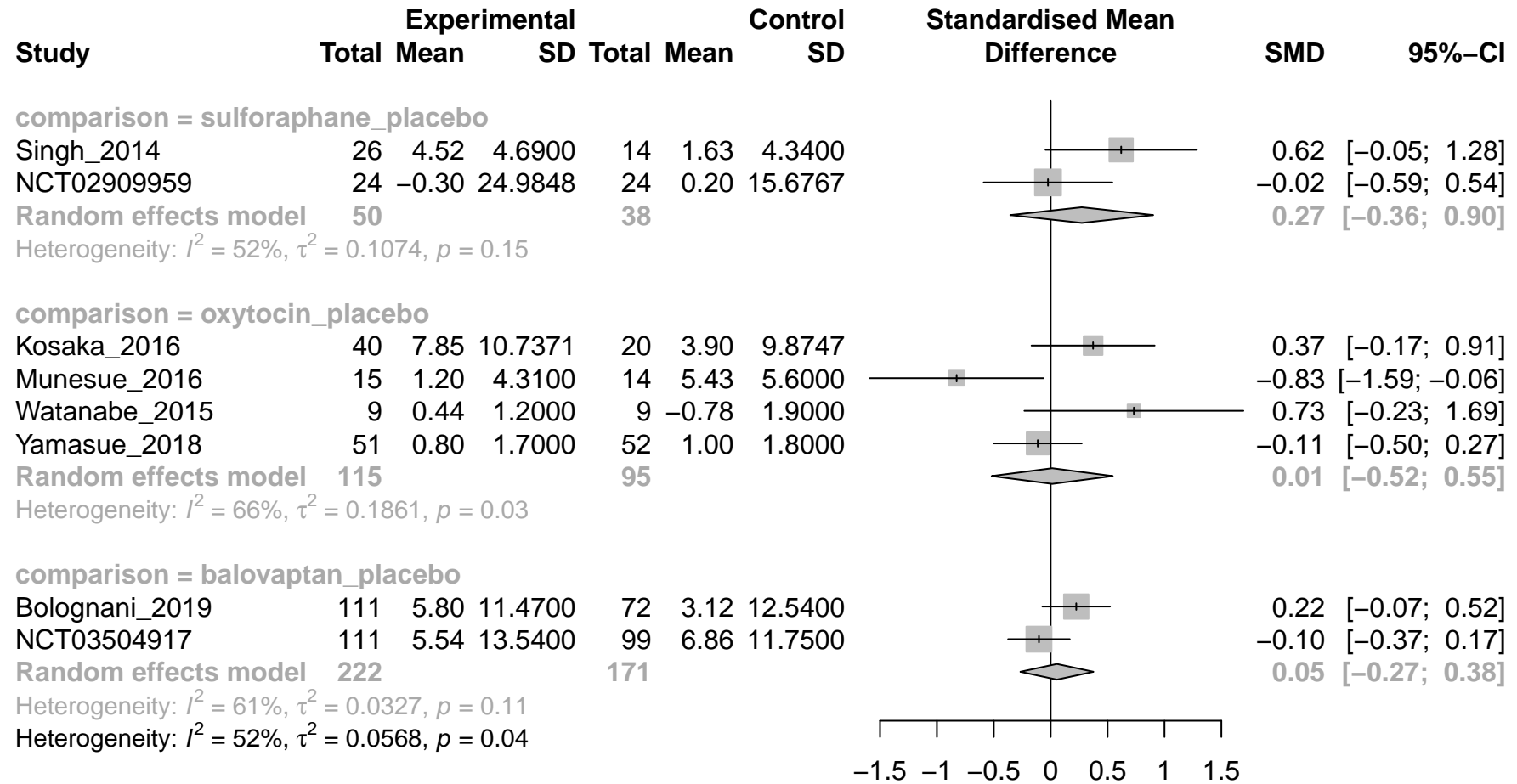

Supplement: Supplementary file 5 — Additional file 5. Fig. S3. Forest plots for pairwise meta-analysis and individual studies. [file 13229_2022_488_MOESM5_ESM.pdf]
