# Supplementary material for: Pharmacological and dietary-supplement treatments for autism spectrum disorder: a systematic review and network meta-analysis
Source: Mol Autism. 2022 Mar 4;13:10. doi: 10.1186/s13229-022-00488-4 (PMC8896153; doi:10.1186/s13229-022-00488-4)

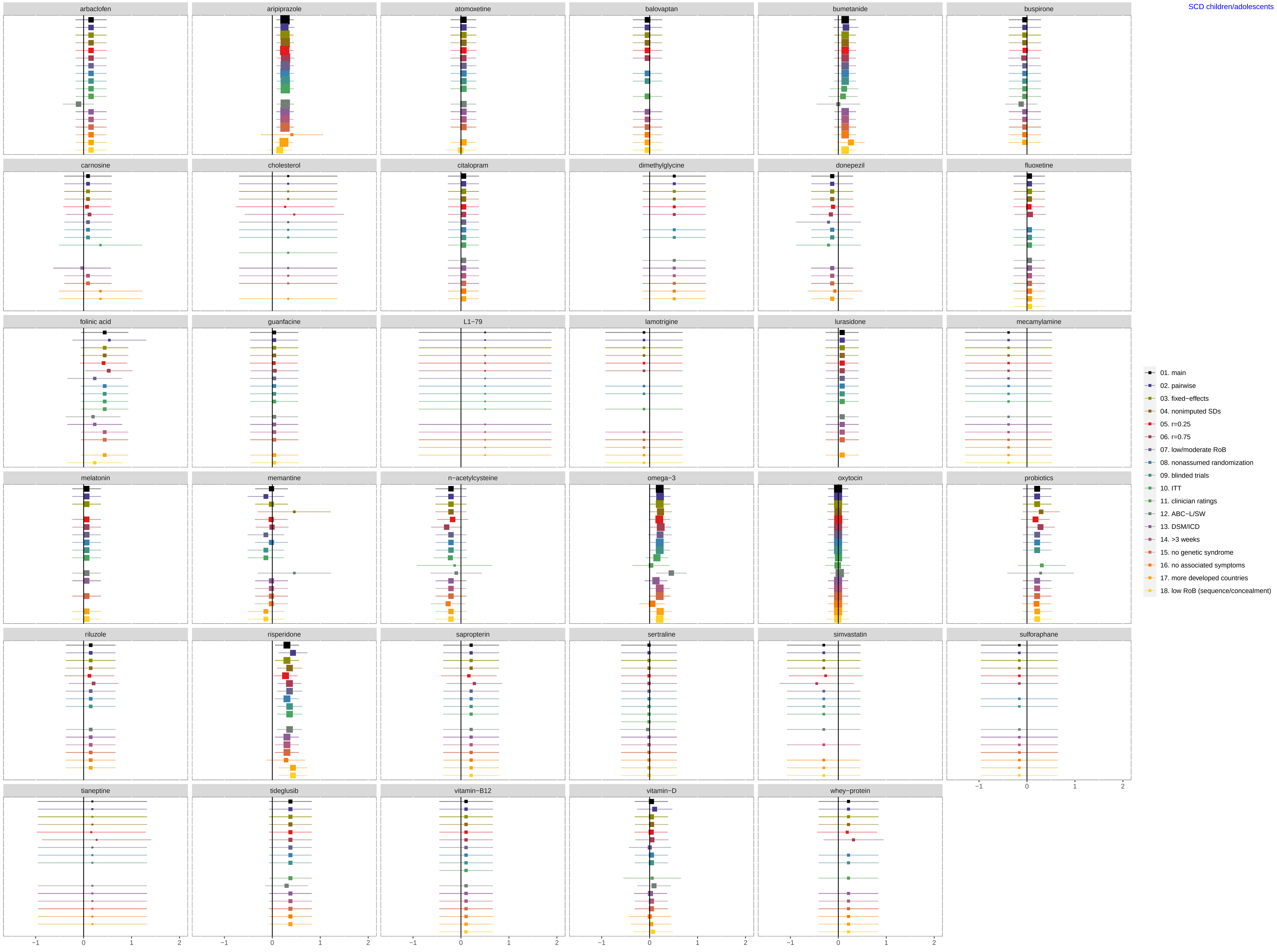
SCD children/adolescents
arbaclofen
aripiprazole
atomoxetine
balovaptan
bumetanide
buspirone
carnosine
cholesterol
citalopram
dimethylglycine
donepezil
fluoxetine
folinic acid
guanfacine
L1−79
lamotrigine
lurasidone
mecamylamine
melatonin
memantine
n−acetylcysteine
omega−3
oxytocin
probiotics
riluzole
risperidone
sapropterin
sertraline
simvastatin
sulforaphane
tianeptine
tideglusib
vitamin−B12
vitamin−D
whey−protein
−1
0
1
2
01. main
02. pairwise
03. fixed−effects
04. nonimputed SDs
05. r=0.25
06. r=0.75
07. low/moderate RoB
08. nonassumed randomization
09. blinded trials
10. ITT
11. clinician ratings
12. ABC−L/SW
13. DSM/ICD
14. >3 weeks
15. no genetic syndrome
16. no associated symptoms
17. more developed countries
18. low RoB (sequence/concealment)

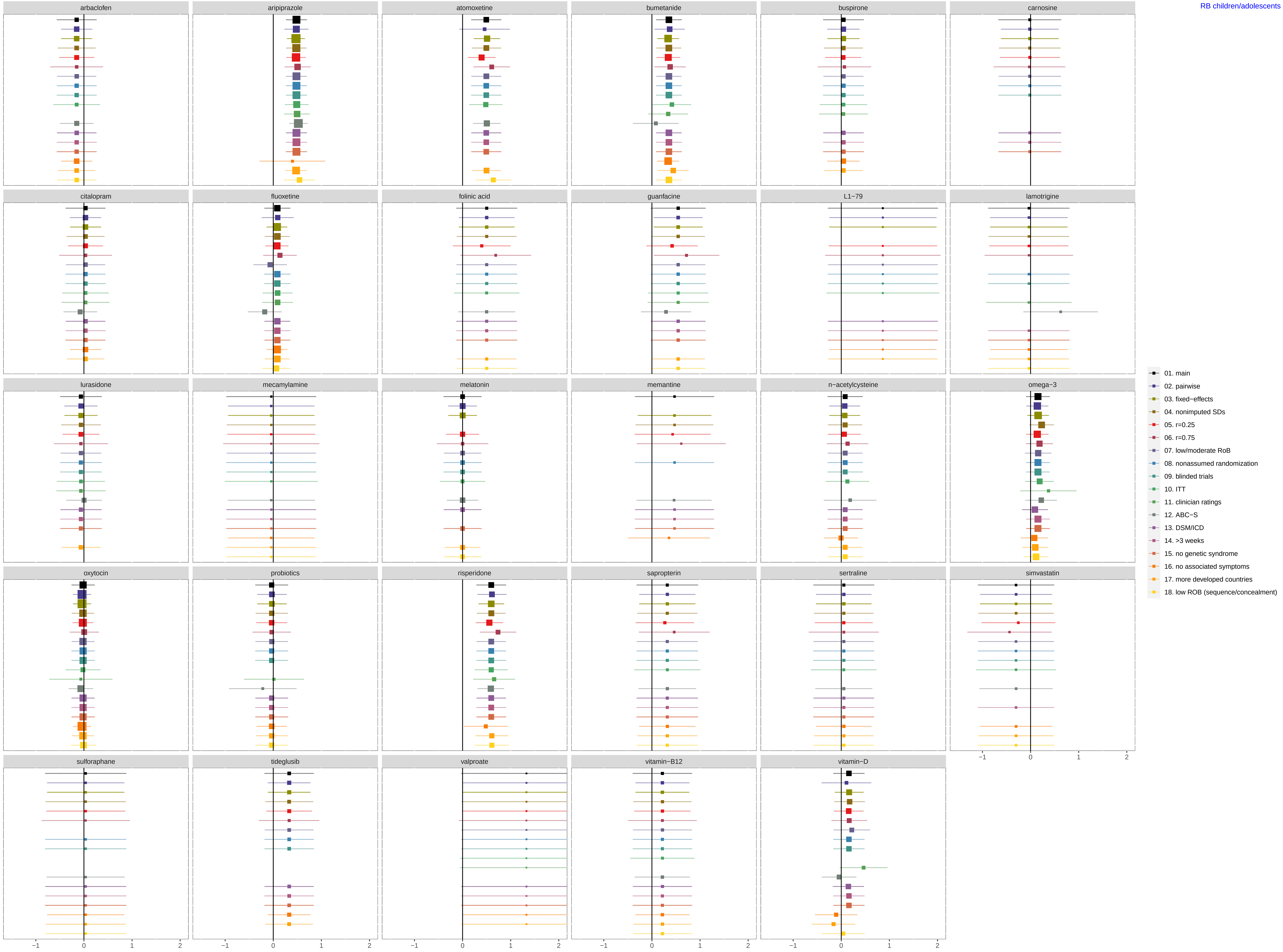
RB children/adolescents
arbaclofen
aripiprazole
atomoxetine
bumetanide
buspirone
carnosine
citalopram
fluoxetine
folinic acid
guanfacine
L1−79
lamotrigine
lurasidone
mecamylamine
melatonin
memantine
n−acetylcysteine
omega−3
oxytocin
probiotics
risperidone
sapropterin
sertraline
simvastatin
sulforaphane
tideglusib
valproate
vitamin−B12
vitamin−D
01. main
02. pairwise
03. fixed−effects
04. nonimputed SDs
05. r=0.25
06. r=0.75
07. low/moderate RoB
08. nonassumed randomization
09. blinded trials
10. ITT
11. clinician ratings
12. ABC−S
13. DSM/ICD
14. >3 weeks
15. no genetic syndrome
16. no associated symptoms
17. more developed countries
18. low ROB (sequence/concealment)
−1
0
1
2

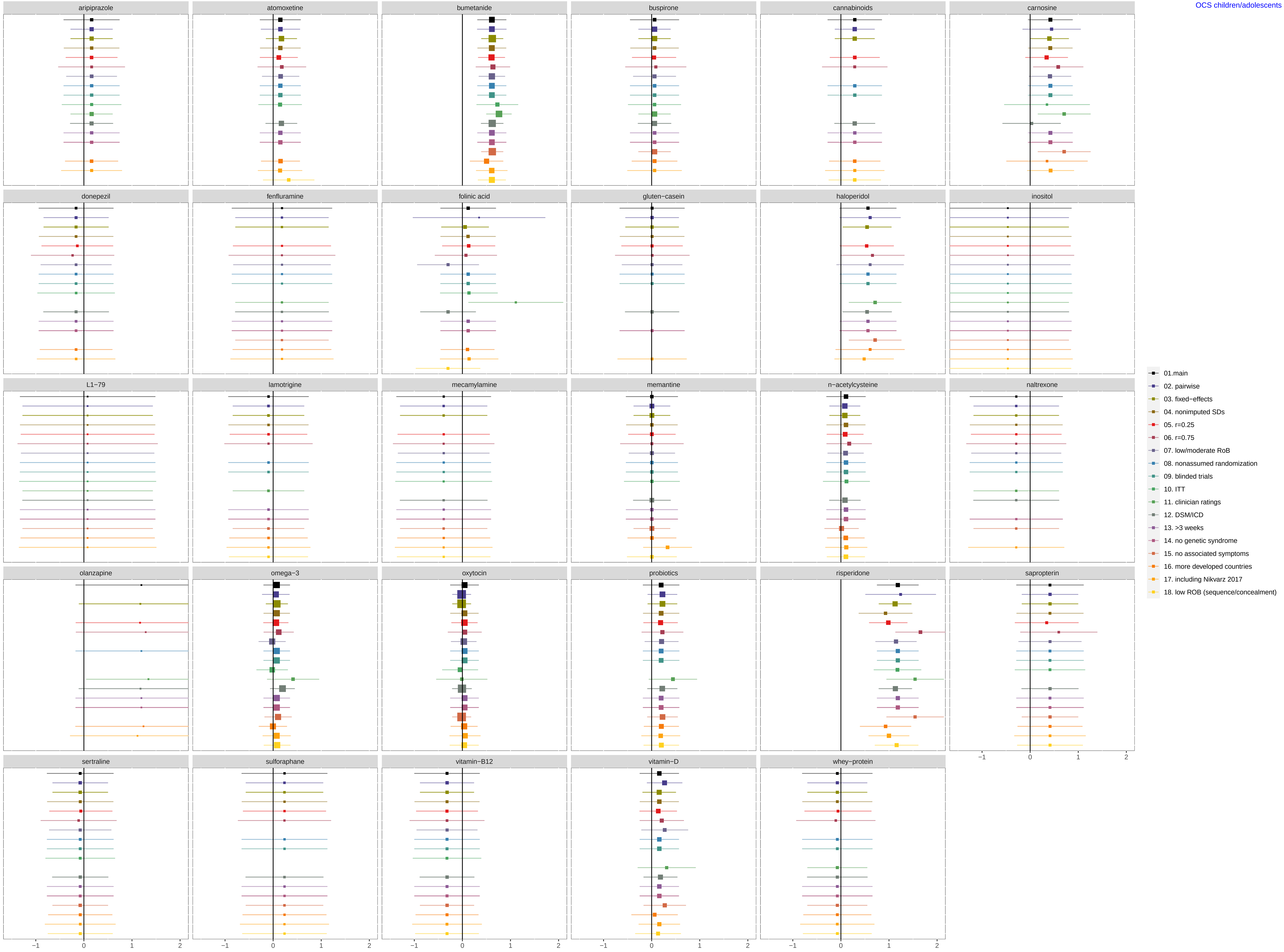
OCS children/adolescents
aripiprazole
atomoxetine
bumetanide
buspirone
cannabinoids
carnosine
donepezil
fenfluramine
folinic acid
gluten−casein
haloperidol
inositol
L1−79
lamotrigine
mecamylamine
memantine
n−acetylcysteine
naltrexone
olanzapine
omega−3
oxytocin
probiotics
risperidone
sapropterin
sertraline
sulforaphane
vitamin−B12
vitamin−D
whey−protein
−1
0
1
2
01.main
02. pairwise
03. fixed−effects
04. nonimputed SDs
05. r=0.25
06. r=0.75
07. low/moderate RoB
08. nonassumed randomization
09. blinded trials
10. ITT
11. clinician ratings
12. DSM/ICD
13. >3 weeks
14. no genetic syndrome
15. no associated symptoms
16. more developed countries
17. including Nikvarz 2017
18. low ROB (sequence/concealment)

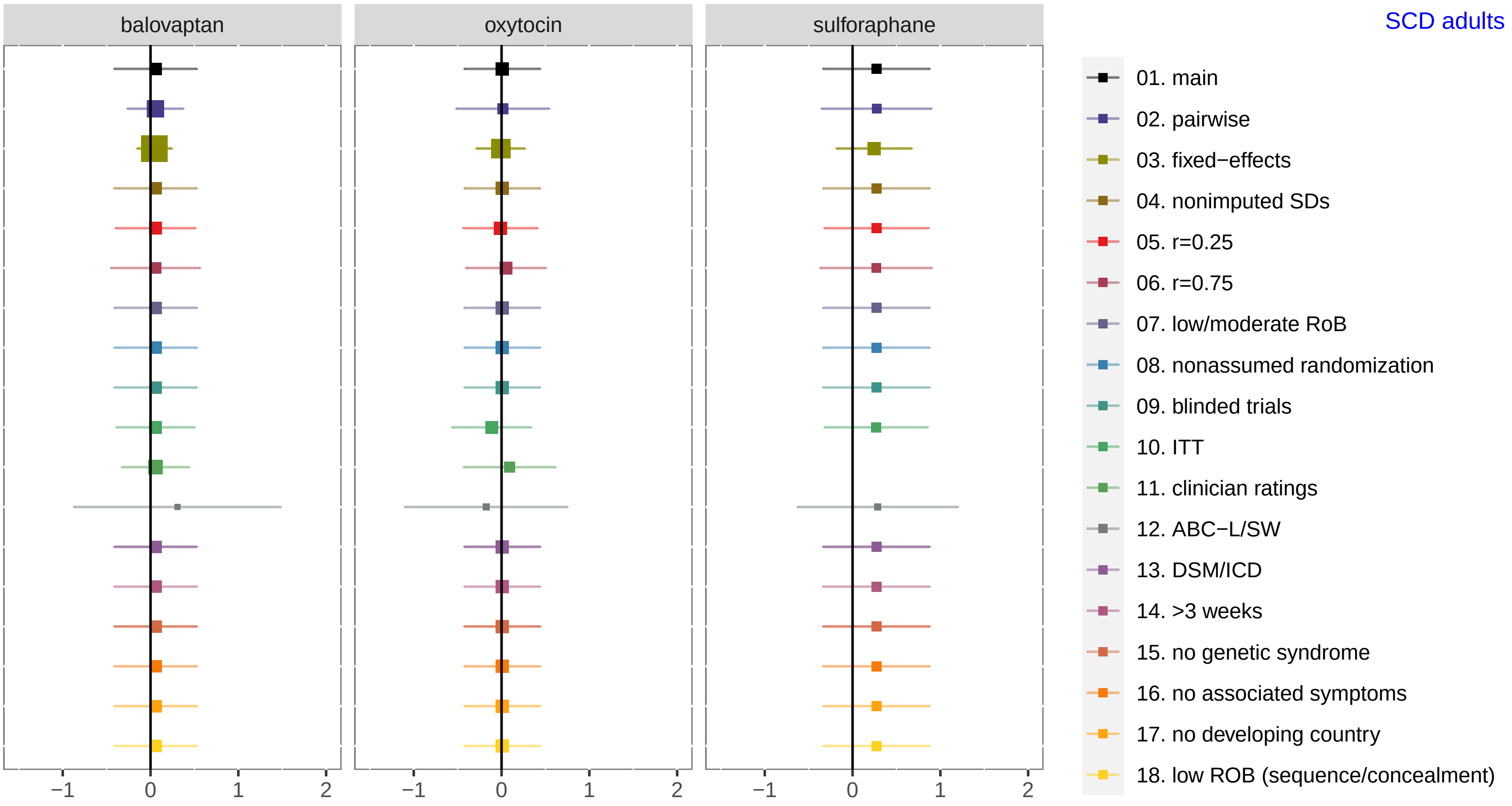

balovaptan
oxytocin
sulforaphane
SCD adults
−1
0
1
2
01. main
02. pairwise
03. fixed−effects
04. nonimputed SDs
05. r=0.25
06. r=0.75
07. low/moderate RoB
08. nonassumed randomization
09. blinded trials
10. ITT
11. clinician ratings
12. ABC−L/SW
13. DSM/ICD
14. >3 weeks
15. no genetic syndrome
16. no associated symptoms
17. no developing country
18. low ROB (sequence/concealment)

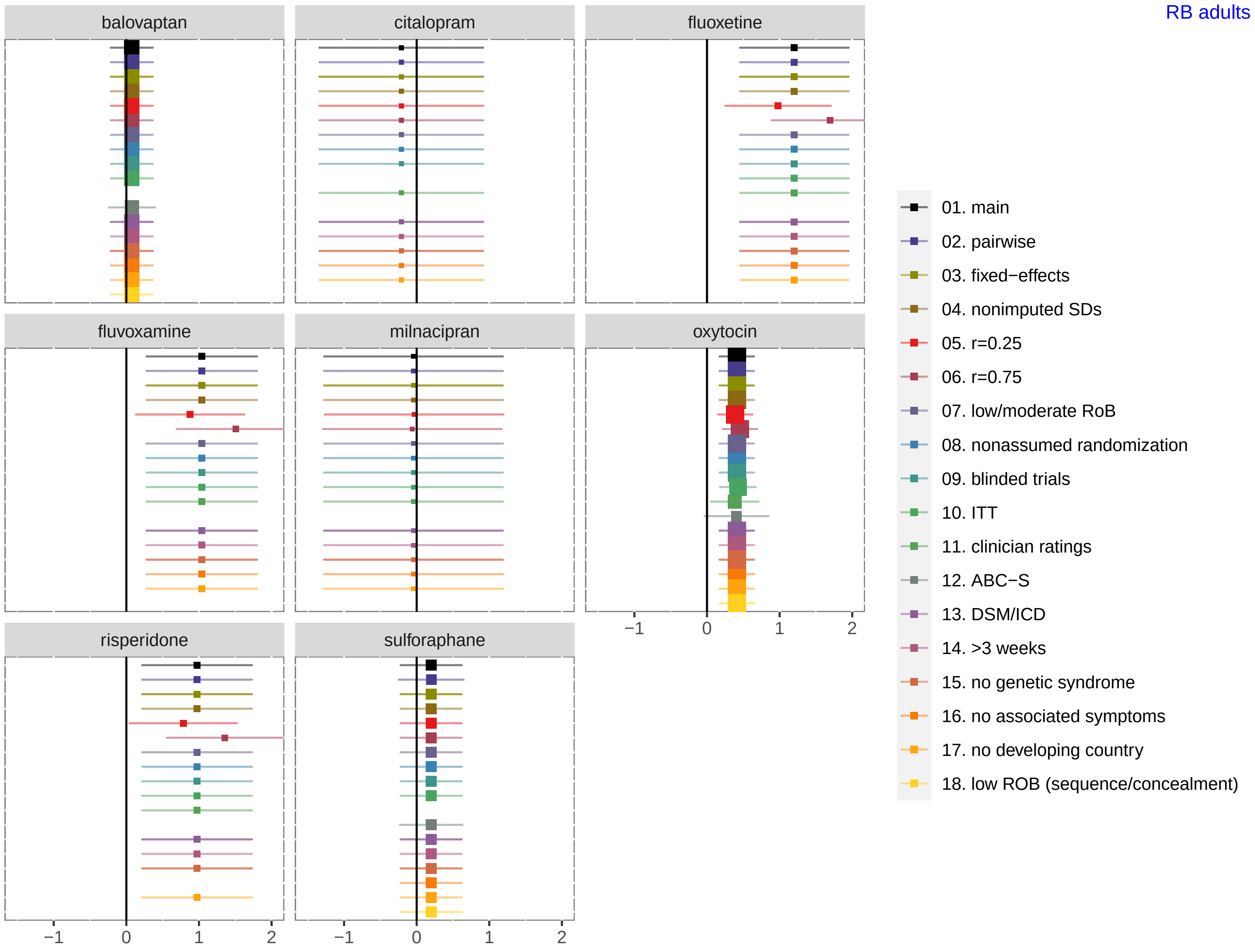

RB adults
balovaptan
citalopram
fluoxetine
fluvoxamine
milnacipran
oxytocin
risperidone
sulforaphane
−1
0
1
2
01. main
02. pairwise
03. fixed−effects
04. nonimputed SDs
05. r=0.25
06. r=0.75
07. low/moderate RoB
08. nonassumed randomization
09. blinded trials
10. ITT
11. clinician ratings
12. ABC−S
13. DSM/ICD
14. >3 weeks
15. no genetic syndrome
16. no associated symptoms
17. no developing country
18. low ROB (sequence/concealment)

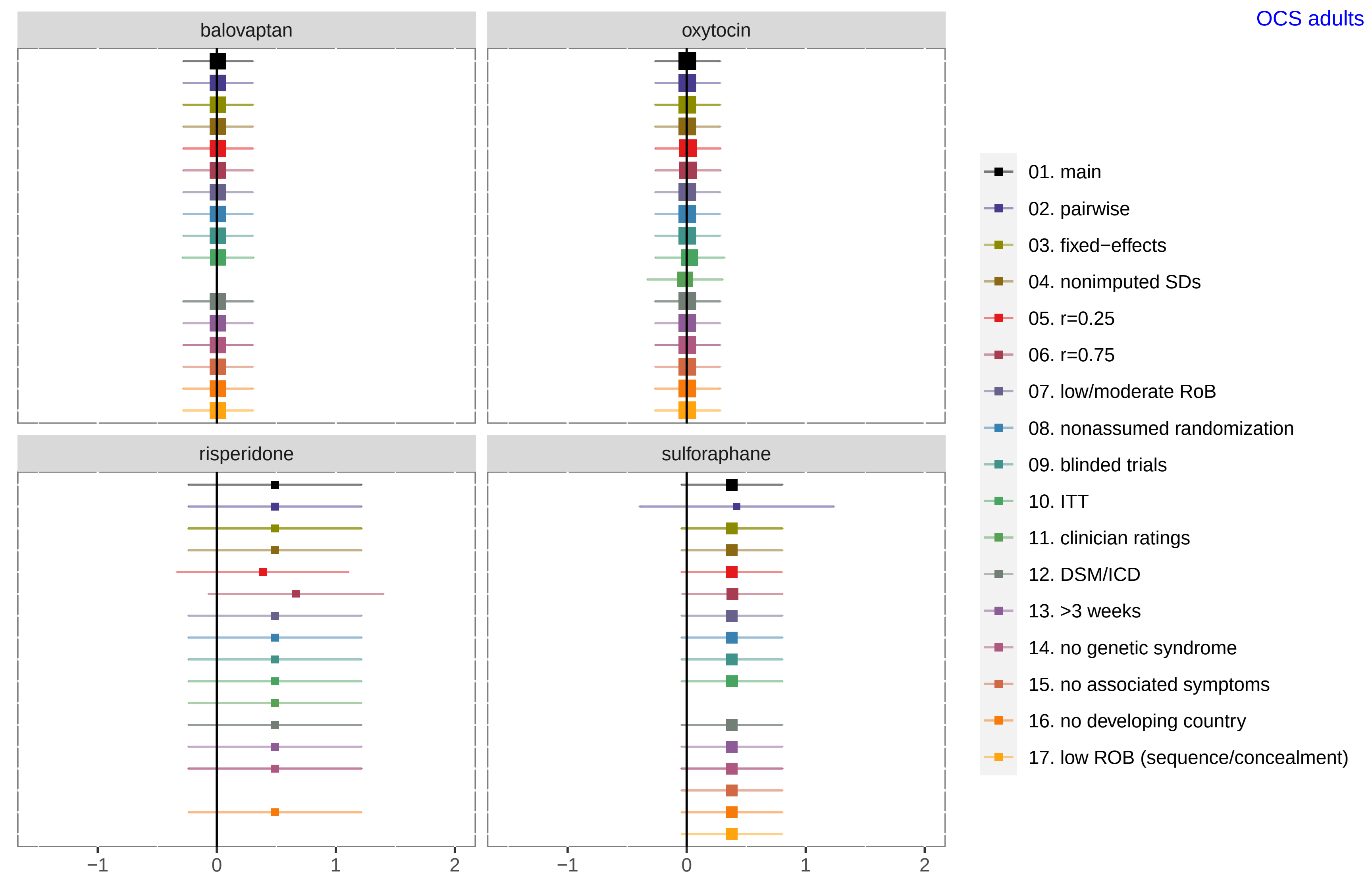

OCS adults
balovaptan
oxytocin
risperidone
sulforaphane
−1
0
1
2
01. main
02. pairwise
03. fixed−effects
04. nonimputed SDs
05. r=0.25
06. r=0.75
07. low/moderate RoB
08. nonassumed randomization
09. blinded trials
10. ITT
11. clinician ratings
12. DSM/ICD
13. >3 weeks
14. no genetic syndrome
15. no associated symptoms
16. no developing country
17. low ROB (sequence/concealment)

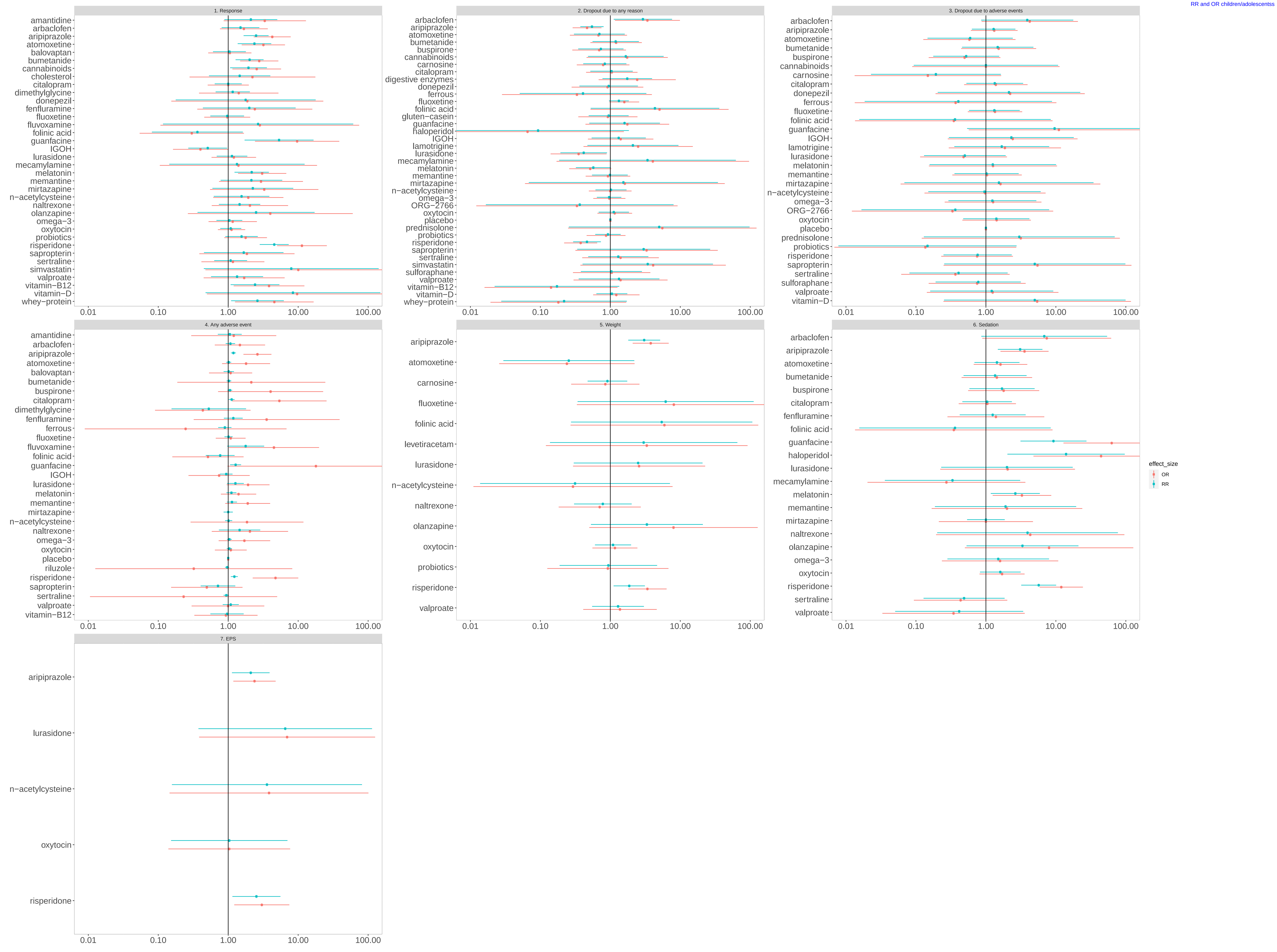
RR and OR children/adolescents
1. Response
amantidine
arbaclofen
aripiprazole
atomoxetine
balovaptan
bumetanide
cannabinoids
cholesterol
citalopram
dimethylglycine
donepezil
fenfluramine
fluoxetine
fluvoxamine
folinic acid
guanfacine
IGOH
lurasidone
mecamylamine
melatonin
memantine
mirtazapine
n−acetylcysteine
naltrexone
olanzapine
omega−3
oxytocin
probiotics
risperidone
sapropterin
sertraline
simvastatin
valproate
vitamin−B12
vitamin−D
whey−protein
2. Dropout due to any reason
arbaclofen
aripiprazole
atomoxetine
bumetanide
buspirone
cannabinoids
carnosine
citalopram
digestive enzymes
donepezil
ferrous
fluoxetine
folinic acid
gluten−casein
guanfacine
haloperidol
IGOH
lamotrigine
lurasidone
mecamylamine
melatonin
memantine
mirtazapine
n−acetylcysteine
omega−3
ORG−2766
oxytocin
placebo
prednisolone
probiotics
risperidone
sapropterin
sertraline
simvastatin
sulforaphane
valproate
vitamin−B12
vitamin−D
whey−protein
3. Dropout due to adverse events
arbaclofen
aripiprazole
atomoxetine
bumetanide
buspirone
cannabinoids
carnosine
citalopram
donepezil
ferrous
fluoxetine
folinic acid
guanfacine
IGOH
lamotrigine
lurasidone
melatonin
memantine
mirtazapine
n−acetylcysteine
omega−3
ORG−2766
oxytocin
placebo
prednisolone
probiotics
risperidone
sapropterin
sertraline
sulforaphane
valproate
vitamin−D
4. Any adverse event
amantidine
arbaclofen
aripiprazole
atomoxetine
balovaptan
bumetanide
buspirone
citalopram
dimethylglycine
fenfluramine
ferrous
fluoxetine
fluvoxamine
folinic acid
guanfacine
IGOH
lurasidone
melatonin
memantine
mirtazapine
n−acetylcysteine
naltrexone
omega−3
oxytocin
placebo
riluzole
risperidone
sapropterin
sertraline
valproate
vitamin−B12
5. Weight
aripiprazole
atomoxetine
carnosine
fluoxetine
folinic acid
levetiracetam
lurasidone
n−acetylcysteine
naltrexone
olanzapine
oxytocin
probiotics
risperidone
valproate
6. Sedation
arbaclofen
aripiprazole
atomoxetine
bumetanide
buspirone
citalopram
fenfluramine
folinic acid
guanfacine
haloperidol
lurasidone
mecamylamine
melatonin
memantine
mirtazapine
naltrexone
olanzapine
omega−3
oxytocin
risperidone
sertraline
valproate
7. EPS
aripiprazole
lurasidone
n−acetylcysteine
oxytocin
risperidone
0.01
0.10
1.00
10.00
100.00
effect_size
OR
RR

RR and OR adults

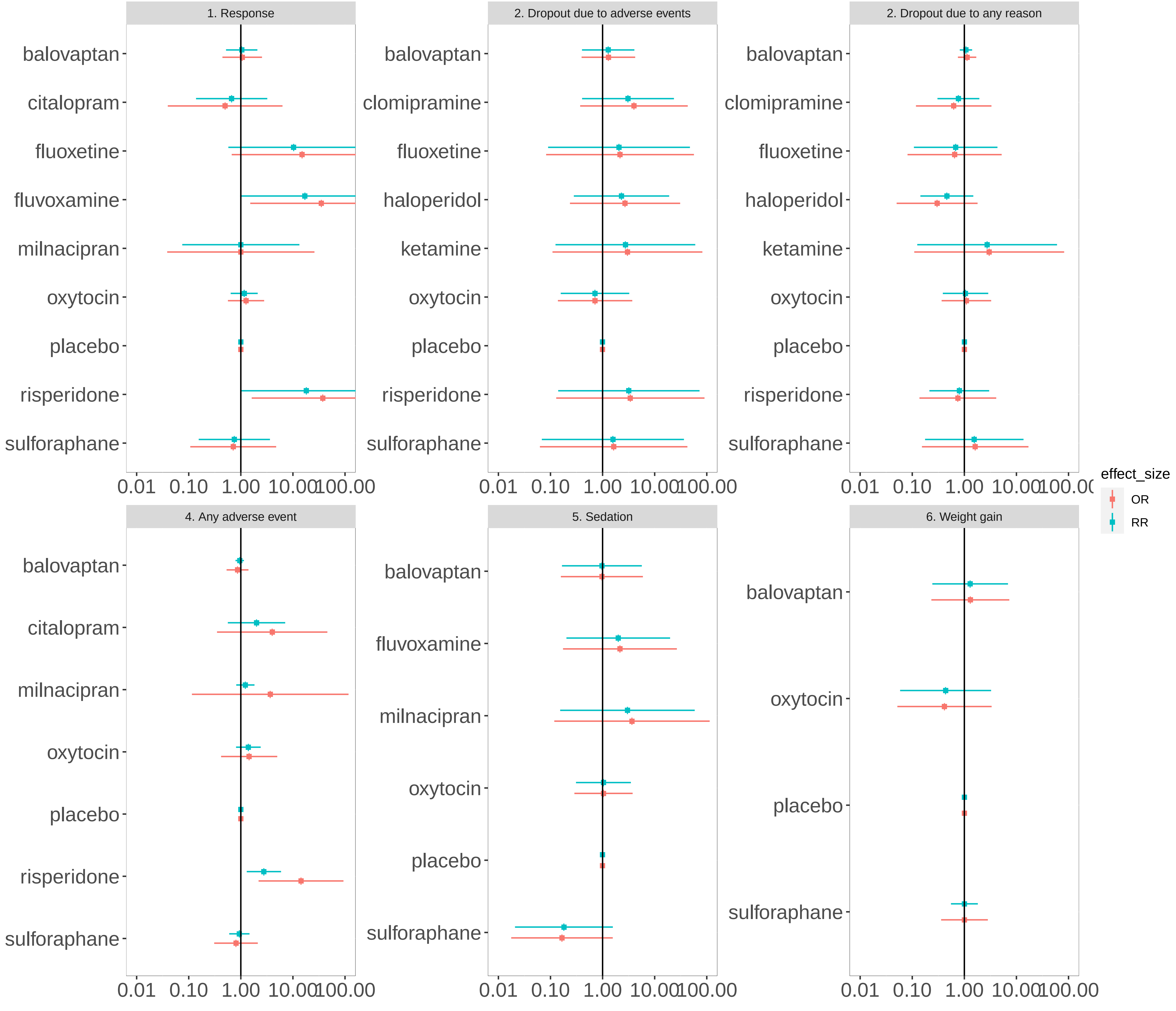

Supplement: Supplementary file 6 — Additional file 6. Fig. S4. Sensitivity analyses. [file 13229_2022_488_MOESM6_ESM.pdf]
